# Asymmetric [3 + 2] photocycloadditions of cyclopropylamines with electron-rich and electron-neutral olefins†

**DOI:** 10.1039/d1sc07044d

**Published:** 2022-03-03

**Authors:** Yating Dai, Shuangshuang Liang, Guangkuo Zeng, Hongchun Huang, Xiaowei Zhao, Shanshan Cao, Zhiyong Jiang

**Affiliations:** School of Chemistry and Chemical Engineering, Henan Normal University Xinxiang Henan P. R. China 453007 caoshanshan@htu.edu.cn jiangzhiyong@htu.edu.cn; International Scientific and Technological Cooperation Base of Chiral Chemistry, Henan University Kaifeng Henan P. R. China 475004 rosamary0530@sina.com

## Abstract

Radical addition to olefins is a common and useful chemical transformation. In the context of offering enantioenriched three-dimensional molecules *via* such a highly reactive process, chiral hydrogen-bonding (H-bonding) catalysis has been widely used to provide enantiocontrol. The current strategies for operating H-bonding induction are confined to following that are prevalent in ionic-type manifolds. Here, we report a novel protocol towards electron-rich olefins based on converting these species from acting as H-bonding donors to acceptors. It facilitates the first development of asymmetric [3 + 2] photocycloadditions with cyclopropylamines. The method is also effective for electron-neutral olefins, in which the successful construction of all-carbon quaternary stereocentres from 1,1-diaryl ethylenes that feature two structurally similar aryl substituents demonstrates the versatility of this new chiral H-bonding catalytic strategy. Furthermore, the importance of the obtained six kinds of products in pharmaceuticals and asymmetric catalysis underscores the practicability of this work.

## Abstract 2

Radical addition to olefins is a common and useful chemical transformation.

## Graphical abstract

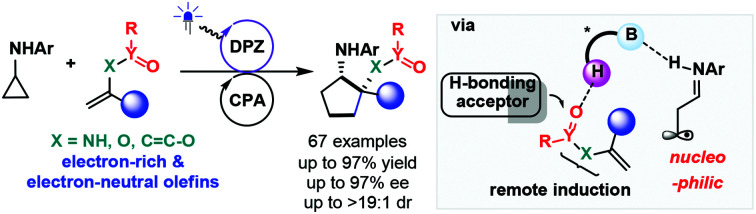

## Introduction

**Fig. 1:**
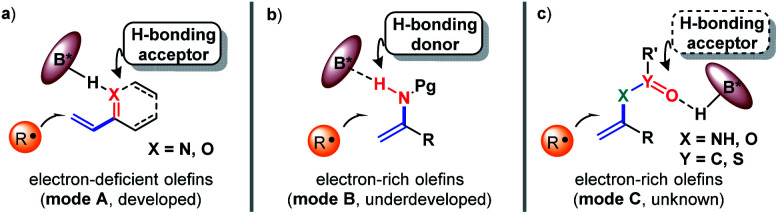
Chiral H-bonding catalytic strategies towards radical addition to olefins.

The exploration of new and efficient enantioselective catalytic strategies represents a fundamental driving force for advancement of chiral chemistry.^1,2^ In this regard, an attractive task theoretically exists in asymmetric H-bonding catalytic chemical transformations based on radical addition to olefins. As a pioneering study, in 2005, the Bach group disclosed the practicality of chiral H-bonding catalysis for enantioselective Giese-type addition of α-amino radicals to amide-activated olefins.^3^ Since then, this working pattern, that is, carbonyls or analogues (*i.e.*, imine-containing azaaryls) near the olefins acting as H-bond acceptors (mode A, Fig. 1a), which is a classic induction strategy involving chiral H-bonding catalysis to provide enantiofacial control in ionic-type conjugate addition reactions, has been extensively used for asymmetric radical additions^4–7^ and [2 + 2] photocycloadditions^8–13^ of electron-deficient olefins. Another common strategy in ground-state reactions involves the use of the base moiety of a chiral catalyst to provide a H-bond acceptor; this is applicable to electron-rich olefins and was recently adopted by Studer and coworkers to provide tandem electrophilic radical addition and asymmetric Minisci-type reactions (mode B, Fig. 1b).^14^ To increase the thermodynamic stability of electron-rich olefins, the protecting groups of heteroatom substituents (*e.g.*, O and N) are usually electron-withdrawing groups, and the highly electronegative atom can serve as a H-bond acceptor. Due to the opposite induction effect to olefins compared to mode B, this putative H-bonding interaction with a chiral catalyst would constitute a new promising application of electron-rich olefins in asymmetric radical chemistry (mode C, Fig. 1c). However, given that these electron-withdrawing groups are connected with the olefins indirectly, remote induction would result in a remarkably weaker influence on olefins than the traditional interaction patterns (*i.e.*, mode A and mode B). Together with the rather low energies of H-bonding interactions,^15^ the ability of this unprecedented H-bonding catalytic strategy to improve chemical transformations and provide enantiocontrol in the highly reactive radical addition process remains elusive.

**Fig. 2:**
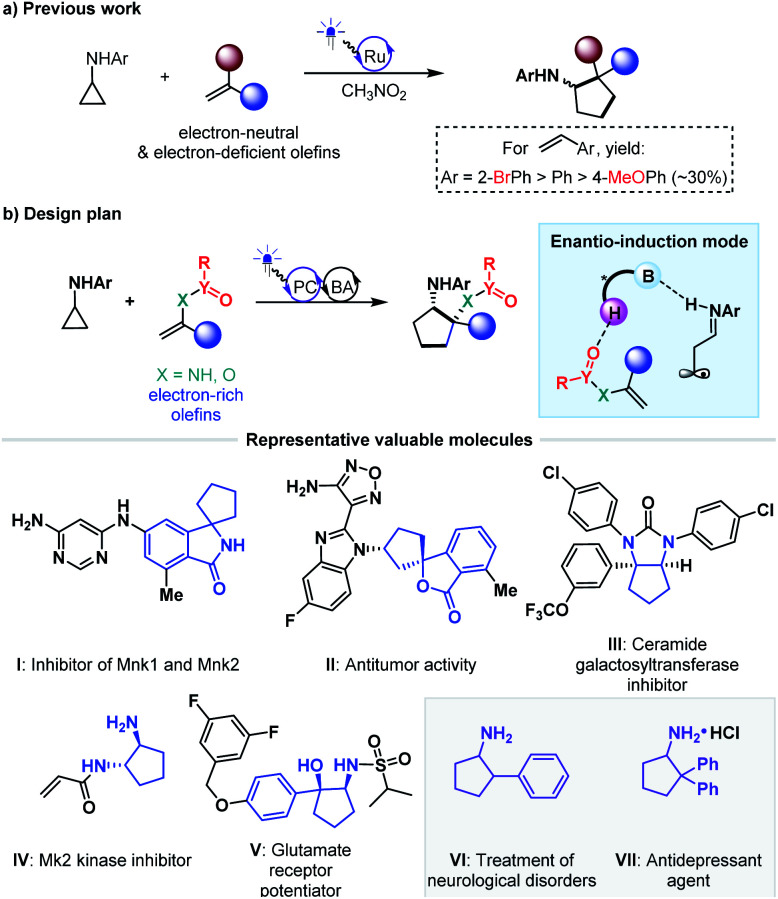
[3 + 2] photocycloadditions of cyclopropylanilines with olefins.

Cycloaddition as a powerful tool to construct valuable cycloalkane derivatives has long been appreciated as one of the most important synthetic applications of olefins.^16^ In recent years, the robust ability to allow use of simple and readily accessible feedstocks and mild reaction conditions and attain good functional group tolerance has attracted increasing attention of chemists to develop diverse photocycloaddition reactions.^9,17,18^ Among them, by utilizing cyclopropylanilines as the reducing species to offer the distonic radical cation intermediates, Zheng and co-workers developed seminal redox-neutral [3 + 2] cycloadditions with olefins, providing a direct and fully atom-economic approach to access biologically important cyclopentylamines (Fig. 2a).^19^ This elegant work then inspired several useful manifolds with electron-neutral and electron-withdrawing olefins as the reaction partners, and some of them have been accomplished in an enantioselective manner.^20–23^ However, the applicability of this tandem twice radical-addition method to electron-rich olefins has not been disclosed to date. Considering the demonstrated high reactivity of radical addition to imines,^6^ we reasoned that the mismatched energy level between the LUMO of the electron-rich olefins and the SOMO of the nucleophilic distonic radical cations might be decisive for this defect. The results disclosed by Zheng that introducing electron-donating groups on the aromatic rings of aryl ethenes deteriorated yields could corroborate this speculation.^19^

As an extension of our research interest in cooperative photoredox and chiral H-bonding catalysis,^24^ we sought to pursue asymmetric [3 + 2] photocycloadditions of cyclopropylanilines with a range of electron-rich olefins (Fig. 2b). In addition to the wide occurrence of the product cyclopentylamines in a range of bioactive molecules (*e.g.*, molecules I–V)^25–33^ and chiral catalysts, the scientific significance of this work includes exploring the feasibility of the desired remote H-bonding induction strategy, since introducing strong electron-withdrawing substituents on the heteroatoms of olefins would decrease the LUMO energy of C–C double bonds.

## Results and discussion

Given that isoinolin-1-one featuring a spiro-five-membered ring is the main structural framework of many bioactive compounds,^31–33^ we began our research by selecting *N*-cyclopropyl-3-methoxyaniline 1a and 3-methylene-isoindolin-1-one 2a as the model substrates (Table 1). The transformation was first evaluated in the presence of 0.5 mol% DPZ^34^ as the photosensitizer in THF at 25 °C and under irradiation with a 3 W blue LED (entry 1). Although electron-withdrawing carbonyl occupied the nitrogen atom, racemic product 3a was obtained in only 11% yield with 6 : 1 dr, indicating rather poor reactivity. We then used 10 mol% diphenyl phosphate (C0) as the catalyst, and the yield of 3a was increased to 57% (entry 2). This suggests that the H-bonding catalyst activated the reaction. The presence of H-bonding interactions between the catalyst and substrate was also manifested by the different diastereoselectivity. To further improve the reactivity, distinct protective groups, such as methyl (2b), *tert*-butyloxy carbonyl (Boc, 2c), tosyl (2d) and 4-(trifluoromethyl)benzenesulfonyl (2e), were introduced on the amide of 2a to decrease the electron density of the nitrogen atom (entries 3–6). Product 3e was obtained in 89% yield with >19 : 1 dr, representing the best result (entry 6). Notably, in the absence of C0, 3e was obtained in 72% yield with 1 : 1 dr (footnote *e*). A series of SPINOL-based chiral phosphoric acids (CPAs) C1–C6 were then tested to determine enantioselectivities (entries 7–12). It was found that catalyst C3 provided enantioenriched product 3e in 83% yield with 81% ee and >19 : 1 dr (entry 9). BINOL-derived CPA C1′, with the same substituent groups at the 3,3′-positions as C3, presented 42% ee, indicating the limited utility of this chiral framework (entry 13). Lowering the reaction temperature improved enantioselectivities (entries 14–17), and a 93% ee was achieved at −30 °C, which was the best outcome (entry 16). The poor yield prompted us to increase the amount of DPZ and reaction concentrations, and prolong the reaction time. Finally, 3e was obtained in 96% yield with 94% ee and >19 : 1 dr when the reaction was carried out in the presence of 1.0 mol% DPZ for 64 h (entry 18). Further investigations showed that other photosensitizers and solvents did not present better results (entries 1–4, Table S1 in the ESI†). The absence of C3 led to production of 3e in 73% yield with 1 : 2 dr (entry 5, Table S1†). Other control experiments indicated the importance of DPZ, visible light and an oxygen-free environment in the success of the reaction (entries 6–8, Table S1†).

**Table 1:** Optimization of the reaction conditions^a^

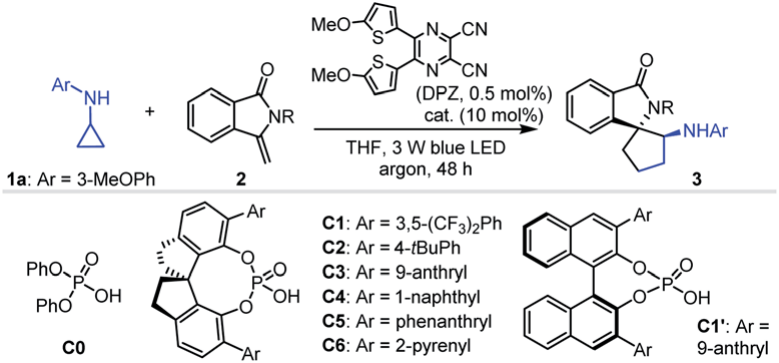						
Entry	2 (R)	Cat.	3	Yield^b^ (%)	Ee^c^ (%)	Dr^d^
1	2a (H)	None	3a	11	N.A.	6 : 1
2	2a (H)	C0	3a	57	N.A.	5 : 1
3	2b (Me)	C0	3b	65	N.A.	2 : 1
4	2c (Boc)	C0	3c	80	N.A.	>19 : 1
5	2d (tosyl)	C0	3d	85	N.A.	>19 : 1
6^e^	2e (4-CF_3_PhSO_2_)	C0	3e	89	N.A.	>19 : 1
7	2e (4-CF_3_PhSO_2_)	C1	3e	87	4	>19 : 1
8	2e (4-CF_3_PhSO_2_)	C2	3e	90	51	>19 : 1
9	2e (4-CF_3_PhSO_2_)	C3	3e	83	81	>19 : 1
10	2e (4-CF_3_PhSO_2_)	C4	3e	85	51	>19 : 1
11	2e (4-CF_3_PhSO_2_)	C5	3e	88	49	>19 : 1
12	2e (4-CF_3_PhSO_2_)	C6	3e	87	63	>19 : 1
13	2e (4-CF_3_PhSO_2_)	C1’	3e	83	42	>19 : 1
14	2e (4-CF_3_PhSO_2_)	C3	3e	80	87	>19 : 1
15	2e (4-CF_3_PhSO_2_)	C3	3e	68	92	>19 : 1
16	2e (4-CF_3_PhSO_2_)	C3	3e	57	92	>19 : 1
17	2e (4-CF_3_PhSO_2_)	C3	3e	42	93	>19 : 1
18^f^	2e (4-CF_3_PhSO_2_)	C3	3e	96	94	>19 : 1

a Reactions were performed with 1a (0.06 mmol), 2 (0.05 mmol), DPZ (2.5 × 10^−4^ mmol) and catalyst (0.005 mmol) in THF (3.0 mL) for 48 h. Entries 1–13, *T* = 25 °C; entry 14, *T* = 0 °C; entry 15, *T* = −20 °C; entry 16, *T* = −30 °C; entries 17–18, *T* = −40 °C.

b Yields of isolated products.

c Enantiomeric excesses (ees) were determined by HPLC analysis with a chiral stationary phase.

d Determined by ^1^H NMR analysis of the crude reaction mixture.

e In the absence of C0, the yield of 3e = 72%, and the dr = 1 : 1.

f The reaction was conducted with 1.0 mol% DPZ in THF (1.0 mL) for 64 h. N.A. = not available.

With the optimal reaction conditions in hand, the scope of asymmetric [3 + 2] cycloadditions of *N*-cyclopropyl arylamines 1 with a range of electron-rich exocyclic terminal olefins was evaluated (Table 2). Several arylamines 1 were first tested in reactions with 3-methylene-isoindolin-1-one 2e, and the corresponding products 3e–h were obtained in 80 to 95% yields with 90–94% ees. A variety of 3-methylene-isoindolin-1-ones 2 featuring different substituents on the aromatic ring were then examined. Regardless of their electronic properties or substitution patterns, all substrates reacted efficiently and produced enantioenriched adducts 3i–u in 75 to 97% yields with 90–97% ees. Since only moderate enantioselectivities were obtained for 3-methylene-isoindolin-1-ones 2 that contain substituents at the 5-position (3q–t), *N*-cyclopropyl 2,5-dimethoxyaniline (1c) instead of 1a was used. Subsequently, methylenephthalide 4, an electron-rich exocyclic terminal olefin that has never been used in asymmetric synthesis, was tested in a reaction with 1a. In the presence of 20 mol% C1 in Et_2_O solution at –55 °C, pharmaceutically important product 5 was obtained in 65% yield with 76% ee.

**Table 2:** Substrate scope of electron-rich exocyclic terminal olefins^a^

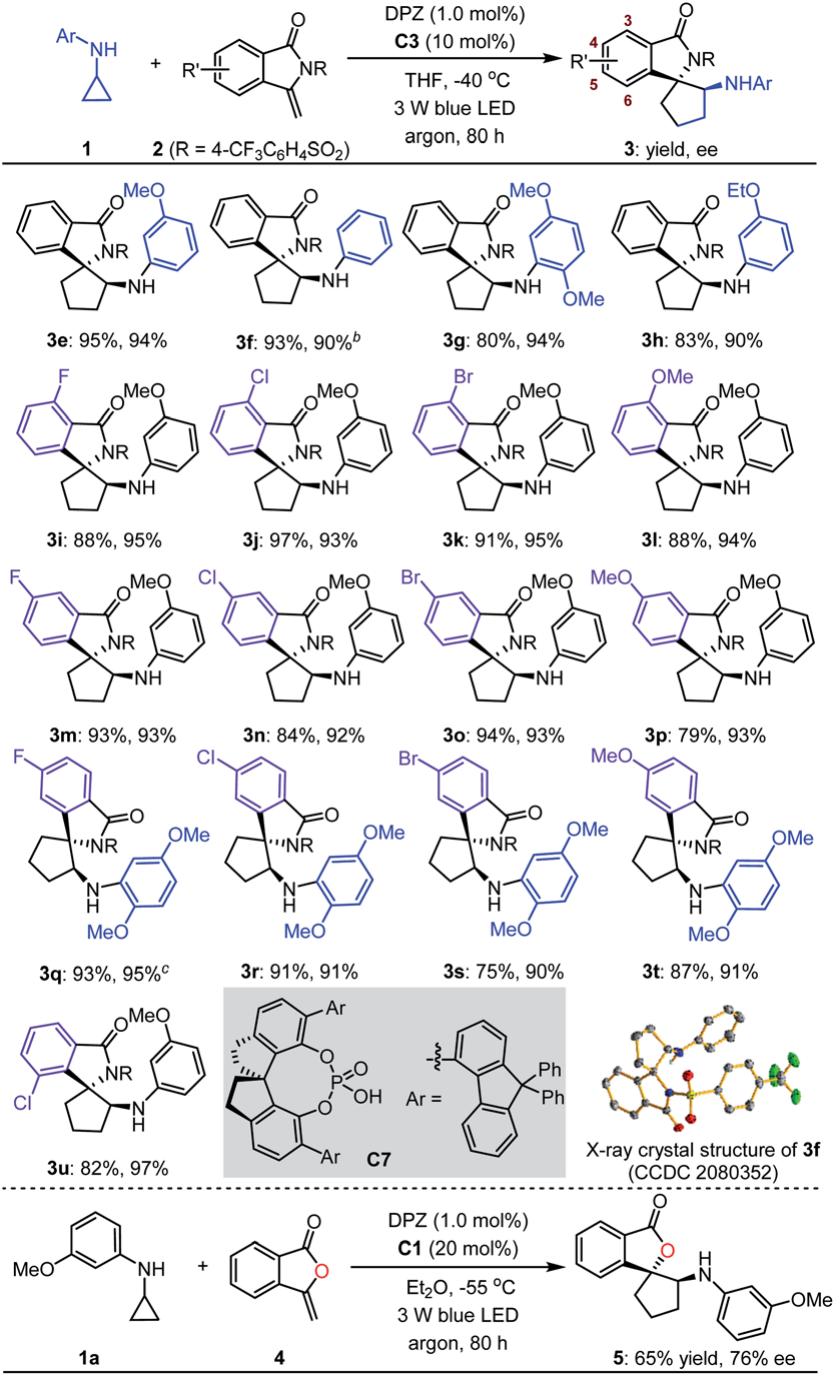

a 0.1 mmol scale. All product drs were determined to be >19 : 1 by ^1^H NMR analysis of crude reaction mixtures.

b C7 (20 mol%) was used instead of C3 and *T* = –60 °C.

c *T* = −5 °C.

Next, we exploited the dual catalysis platform to evaluate [3 + 2] cycloadditions of *N*-cyclopropyl arylamines with various electron-rich linear terminal olefins, which would provide direct and general access to highly important derivatives of both cyclopentane-1,2-diamine and α-amino cyclopentanol (*e.g.*, molecules III–V, Fig. 2) in an enantioselective fashion. With respect to the first class of target products, we began our investigation by examining *N*-protecting groups for cyclopropyl amine, *N*-activating groups for α-phenyl ethenamine and reaction parameters (for more details, see Tables S2 and S3 in the ESI†). As a result, enantioenriched 1-phenyl *trans*-cyclopentane-1,2-diamine 7a was obtained in 77% yield with 95% ee and >19 : 1 dr (Table 3). Subsequent evaluations revealed that a variety of 1-aryl *N*-2-chloro-benzoyl ethenamines 6 were well tolerated in reactions with 1c, leading to the corresponding products 7b–m in 66–83% yields with 85–95% ees and 2 : 1 to >19 : 1 drs. The high yields, enantioselectivities and diastereoselectivities achieved with fused aromatic (7l) and heteroaromatic (7m) rings at the α-positions of olefins underscore the generality of this catalytic system. We also attempted reactions with α-alkyl-substituted terminal olefins, but these transformations did not proceed. We next synthesised α-amino cyclopentanol derivatives by using O-protected α-branched ethenols. As a representative example, the use of 20 mol% CPA C1 as the chiral catalyst in a reaction of 1a with 8 furnished product 9 in 60% yield with 80% ee and 7 : 1 dr.

**Table 3:** Substrate scope of electron-rich linear terminal olefins^a^

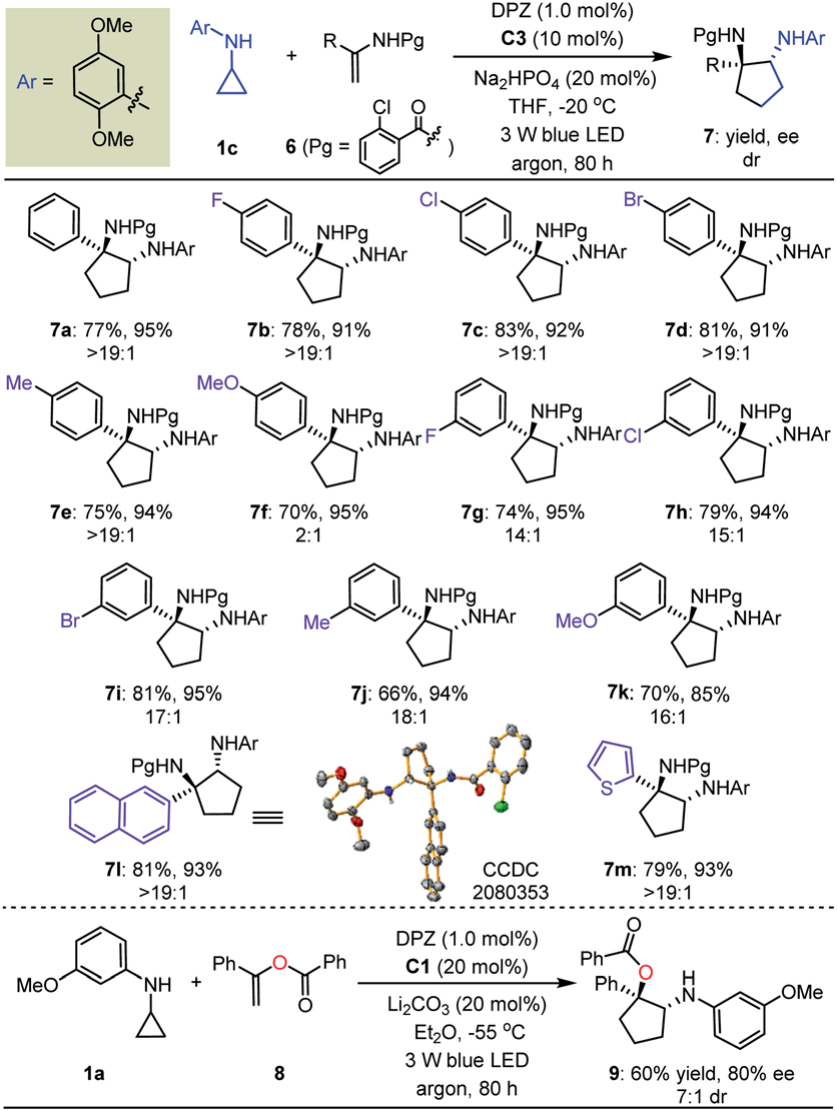

a 0.1 mmol scale. All product drs were determined by ^1^H NMR analysis of the crude reaction mixture.

Building on these encouraging results, we then explored the reactions of *N*-cyclopropyl arylamines with electron-neutral olefins to extend the application of remote H-bonding induction. In previous work on catalytic asymmetric radical-based reactions of electron-neutral olefins, the interaction of the chiral catalyst with another reaction partner to offer enantioface differentiation constituted the only strategy.^35–45^ It is worth mentioning that since no successful examples were reported in the elegant contribution of Ooi,^20^ arylethylenes and 1,1-diarylethylenes were selected for examination, thereby establishing expedient and modular access to biologically important α-aryl and α,α-diaryl cyclopentylamines (*e.g.*, compounds VI and VII, Fig. 2b)^46–48^ in an enantioselective fashion. Given the highly similar steric effects, stereocontrol of transformations of 1,1-diarylethylenes might constitute a more formidable challenge.

Initially, we tested the reaction between 1a and styrene using 0.5 mol% DPZ in THF solution at 25 °C with irradiation with a 3 W blue LED. However, no reaction was detected. Catalyst C0 also failed to promote the transformation. We then introduced a variety of ester groups onto the aromatic rings of olefins to activate the olefins and assemble H-bond acceptors (Tables S4 and S5 in the ESI†). These reactions proceeded, but enantioselectivities and diastereoselectivities were very poor. We then carefully tested various acyloxy and sulfonyloxy derivatives as substituents instead of esters and distinct N-protecting groups of cyclopropyl amines, and we modified reaction parameters (Tables S6–S8 in the ESI†). To our delight, in the presence of 2.0 mol% DPZ, 10 mol% CPA C3, KCl and 18-crown-6 as additives in 1,2-dimethoxyethane (DME) solution at –50 °C, the reaction of *N*-cyclopropyl arylamine 1aa with 2-vinylphenyl trifluoromethanesulfonate afforded product 11a in 80% yield with 84% ee and >19 : 1 dr (Table 4). By maintaining the *ortho*-OTf (Tf = trifluoromethylsulfonyl) substituent, olefins featuring different electron-withdrawing and electron-donating groups at different positions of aromatic rings were competent, resulting in products 11b–i in 64 to 80% yields with 75–85% ees and >19 : 1 drs.

**Table 4:** Substrate scope of arylethylenes^a^

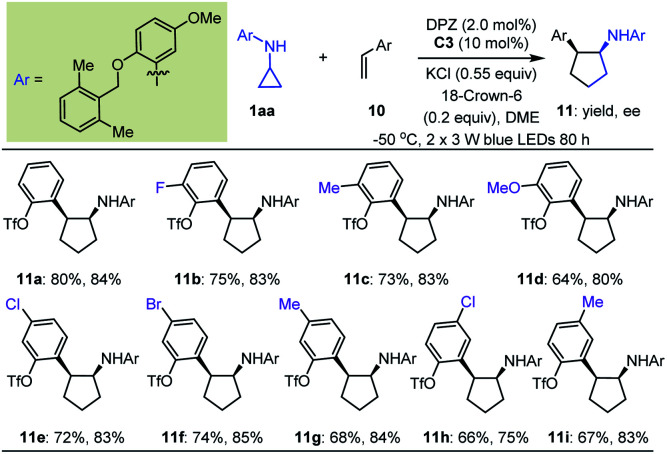

a Reactions were conducted with 1aa (0.17 mmol), 10 (0.1 mmol), DPZ (0.002 mmol), C3 (0.01 mmol), KCl (0.055 mmol) and 18-crown-6 (0.02 mmol) in 2.0 mL DME. All product drs were determined to be >19 : 1 by ^19^F NMR analysis of crude reaction mixtures.

A detailed investigation of the optimal reaction conditions for 1,1-diarylethylenes was also carried out (Table S9 in the ESI†). Finally, *N*-cyclopropyl arylamine 1bb was determined to be a suitable reaction partner for olefins (Table 5). Moreover, the use of OTf as a directing group for olefins provided products 13a–c with satisfactory results. Among them, *ortho*-OTf (13a) presented the highest diastereoselectivity, but the transformation was slightly sluggish, and *para*-OTf (13c) gave the best yield and enantioselectivity. Accordingly, a variety of 1,1-diarylethylenes 12 containing *para*-OTf on an aromatic ring were first tested. It was found that olefins bearing different electron-withdrawing or electron-donating groups on the *ortho*-position of other aromatic rings furnished products 13d–g in 67 to 87% yields with 93–94% ees and >19 : 1 drs. When these substituents were on the *meta*-position (13h–j), excellent enantioselectivity was maintained, but diastereoselectivities were slightly decreased; notably, the N-protecting group of the cyclopropyl amine was 2,6-dimethoxyphenyl in these cases. Virtually no diastereoselectivity was obtained for *para*-substituents (13k–l). To our delight, fused aromatic (13m) and heteroaromatic (13n) rings were well tolerated. We then investigated substituent effects for aryl rings containing a *para*-OTf substituent. It was found that a series of corresponding products 13o–t were achieved in 60% to 85% yields with 89% to 93% ees and 8 : 1 to >19 : 1 drs under slightly modified conditions. These inspiring results also prompted us to evaluate halogen-substituted 1,1-diarylethylenes, given their electron-withdrawing properties and ability to act as hydrogen-bond acceptors. The results indicated that *ortho*-substitution (13t–w) afforded higher diastereoselectivity than *para*-substitution (13x). Finally, we used this protocol to synthesize enantioenriched cyclopentylamines with two identical phenyl substituents on the α-position owing to the known bioactive importance of these compounds (*e.g.*, VII, Fig. 2b).^46^ Transformation of 1bb with α-phenylstyrene generated product 13y in 36% yield and 88% ee. The poor yield propelled us to test an olefin containing two *para*-OTf-substituted phenyl substituents at the α-position. Adduct 13z was obtained in 80% yield with 95% ee, indicating the importance of OTf in accelerating the reaction and improving the enantioselectivity.

**Table 5:** Substrate scope of 1,1-diarylethylenes^a^

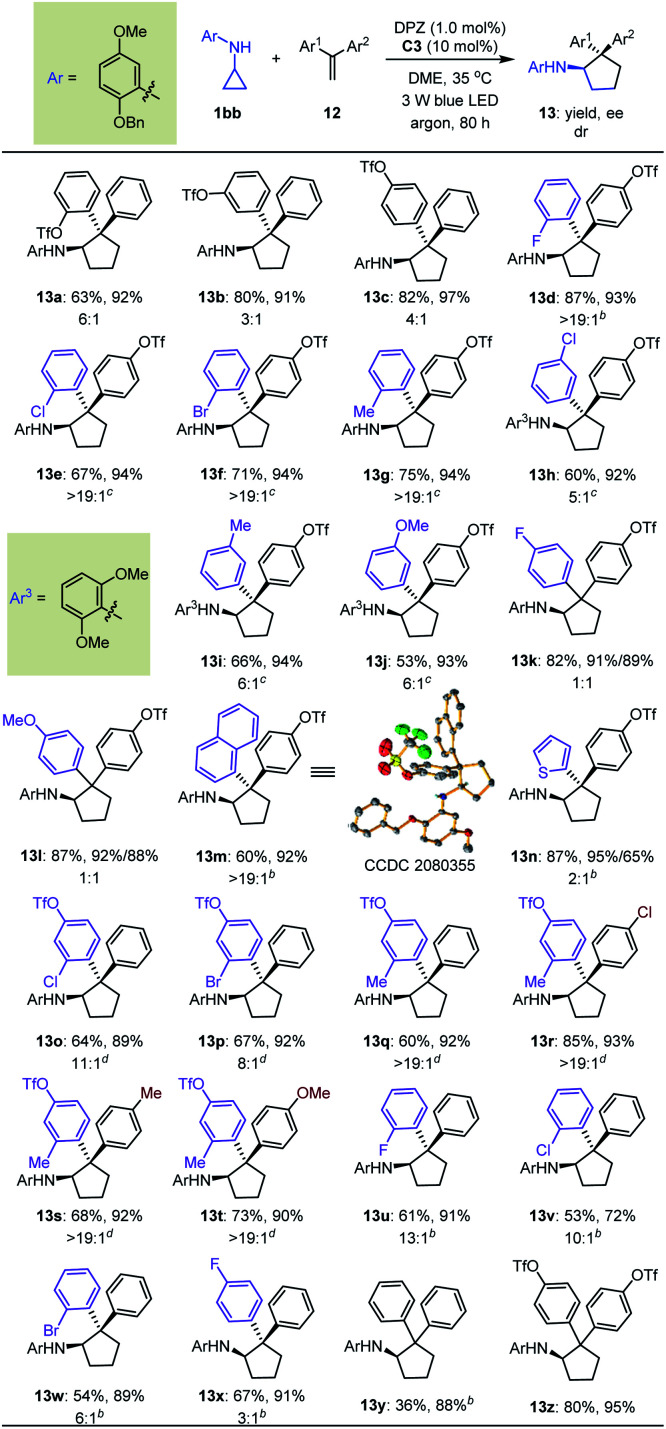

a Reactions were conducted with 1bb (0.17 mmol), 12 (0.1 mmol), DPZ (0.002 mmol), and C3 (0.01 mmol) in 1.0 mL DME. All product drs were determined by ^19^F NMR and ^1^H NMR analyses of the crude reaction mixture.

b 2.0 mol% DPZ was used.

c *T* = 25 °C.

d 2.0 mol% DPZ was used at 25 °C.

**Fig. 3:**
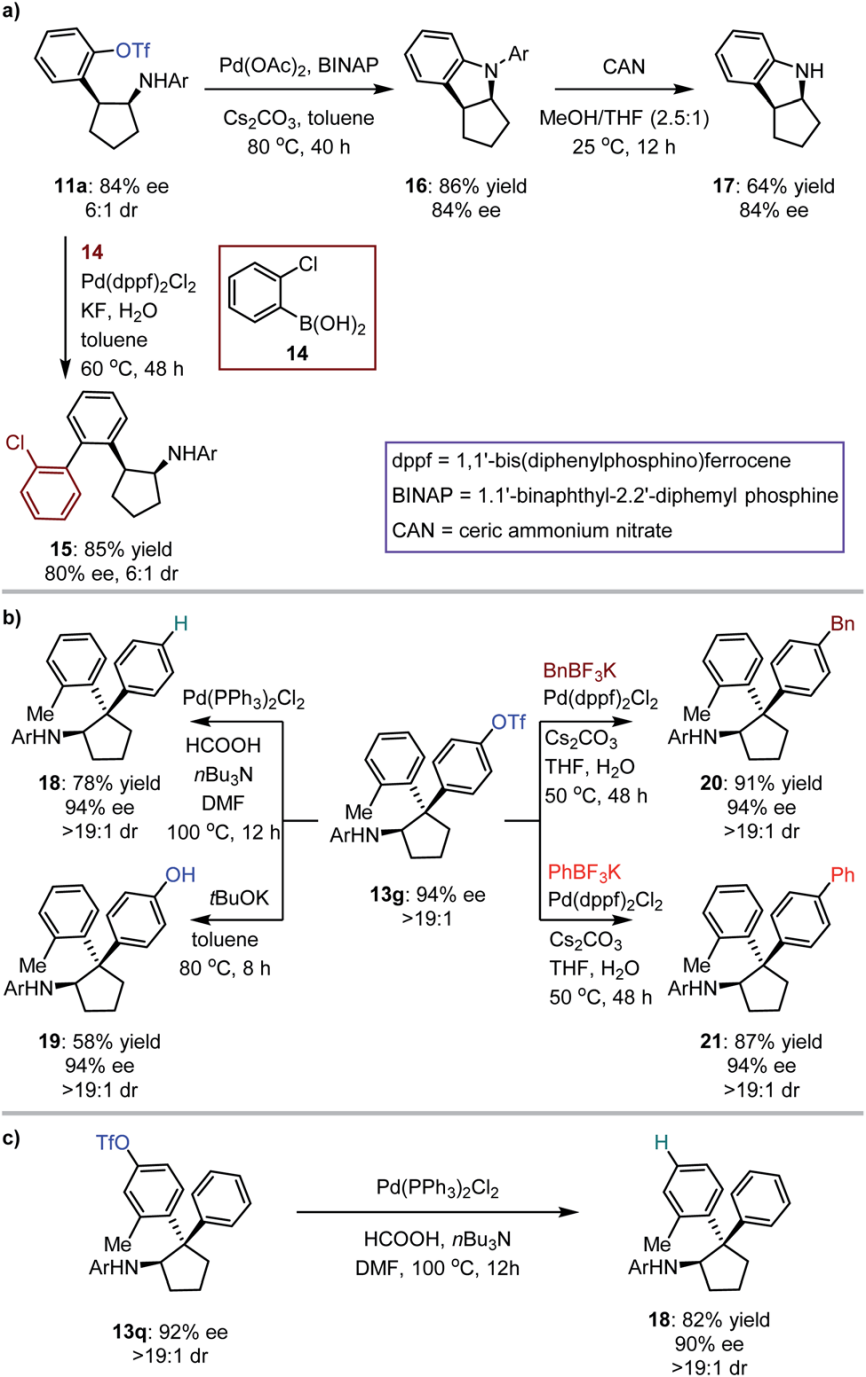
Synthetic applications.

The synthetic importance of [3 + 2] photocycloaddition products (*i.e.*, 3, 5, 7 and 9) derived from electron-rich olefins was highlighted by the structural features that serve as core frameworks of numerous bioactive molecules, *e.g.*, compounds I–V, as depicted in Fig. 2b. Although adducts generated from the electron-neutral olefins (*i.e.*, 11 and 13) also feature substantial significance (*e.g.*, compounds VI–VII, Fig. 2b), we were still interested in performing several chemical transformations with preloaded OTf on the aromatic rings to prove that introduction of this auxiliary group did not restrict the application of the method but enriched it (Fig. 3). Cyclopentylamine 11a was first selected to react with (2-chlorophenyl)boronic acid 14 by using Pd(dppf)_2_Cl_2_ as the catalyst, and Suzuki coupling product 15 was readily achieved in 85% yield with 80% ee (Fig. 3a). Furthermore, its *cis*-configuration inspired us to test the viability of intramolecular cross coupling. In the presence of Pd(OAc)_2_ and BINAP, transformation of 11a provided the biologically important indoline derivative^49,50^16 in 86% yield with 84% ee. By treatment with CAN, the *N*-aryl group of 16 was excised to produce compound 17 without compromising the ee. Subsequently, 13g, a representative cyclopentylamine that bears an all-carbon quaternary stereocentre featuring two distinct aryls, was treated with Pd(PPh_3_)Cl_2_ and HCOOH, and the OTf substituent was removed effectively, resulting in product 18 in 78% yield (Fig. 3b). Inarguably, this method could be used to synthesize OTf-free cyclopentylamine derivatives from [3 + 2] photocycloaddition adducts 11 and 13. Additionally, removal of the trifluoromethanesulfonyl group (Tf) of 13g with *t*BuOK furnished phenolic hydroxyl-containing product 19 in 58% yield with 94% ee. In the presence of Pd(dppf)_2_Cl_2_, reactions between 13g and potassium benzyltrifluoroborate or potassium phenyltrifluoroborate generated the corresponding coupling products 20 and 21 with satisfactory results. The cleavage of OTf was also carried out for adduct 13q, leading to product 18 in 82% yield with 90% ee (Fig. 3c). This indicates the absolute configurations of 13o–13t, and, more importantly, shows that the difference in steric hindrance of the aromatic rings is crucial for enantiofacial differentiation at this quaternary stereocentre.

We subsequently investigated a plausible mechanism for this [3 + 2] photocycloaddition reaction. First, Stern–Volmer experiments revealed that the transformation was most likely triggered by reductive quenching of *DPZ with *N*-aryl cyclopropylamines (see the ESI†). The quantum yield (*Φ*) for the transformation providing 3a was determined to be 0.284, indicating that a chain propagation mechanism is probably not involved. Moreover, the relationship between the ee of CPA C3 and the ee of product 3a was evaluated, and a linear correlation was identified, suggesting that a single molecule of C3 was involved in the stereocentre-forming step. Given the existence of several H-bond donors on electron-rich olefins (*i.e.*, 2, 4, 6 and 8), the operation of chiral Brønsted acid C3 as a bifunctional catalyst is conceivable. Hence, we were more interested in exploring the role of the versatile OTf group in electron-neutral olefins (*i.e.*, 10 and 12) for stereocontrol by using chiral catalysts to determine whether the desired mode C (Fig. 1) is practicable.

**Scheme 1:**
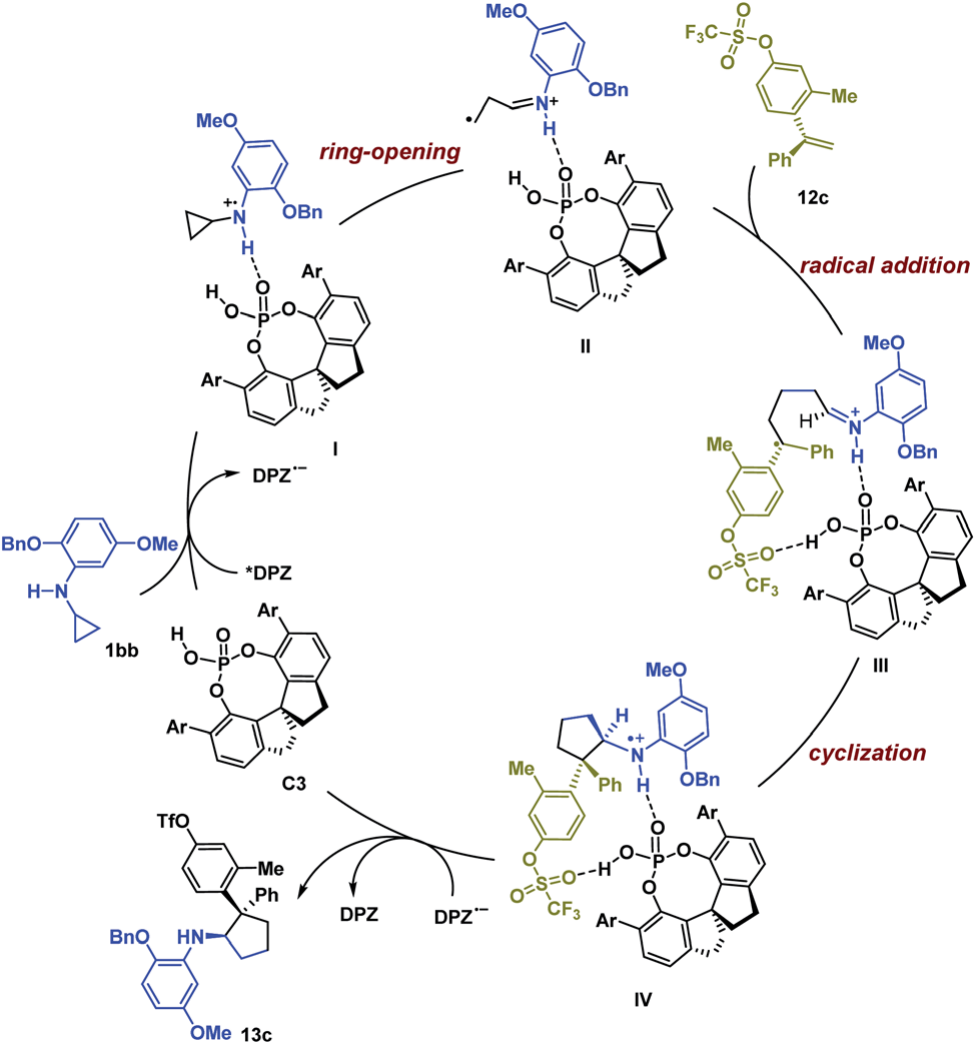
Plausible reaction pathway.

Based on the experimental results and previous work,^6,19^ a possible reaction pathway was developed by selecting the transformation of 1bb with 12c as a representative example (Scheme 1). Initially, catalyst C3 and 1bb might form a non-covalent complex with a H-bonding interaction between the N–H of 1bb and the P=O of C3. After photoexcitation, *DPZ serves as an oxidant and is reduced by the amine moiety of 1bb, and then the resulting radical cation I undergoes ring-opening to form intermediate II. During this step, the chiral information of C3 remains. With the help of another H-bonding interaction between the OTf of 12c and the P–OH group of C3, the subsequent intermolecular radical addition proceeds and leads to radical intermediate III. Given the formation of stereogenic centres, we suggest that the subsequent cyclization *via* radical addition to generate radical cation IV is responsible for determining enantio- and diastereoselectivities. Finally, the oxidizing amine radical cation IV accepts an electron from DPZ˙^−^ to give product 13c.

**Fig. 4:**
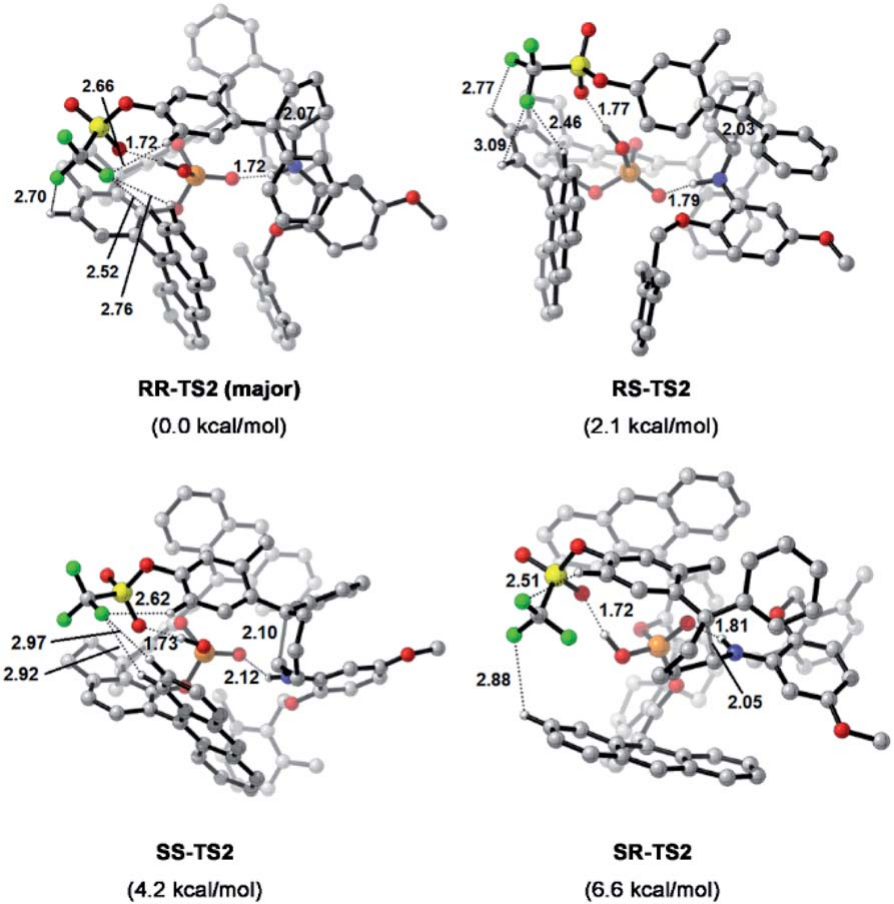
Calculated transition states and relative free energies of transition states. Energies are given in kcal mol^−1^. Bond lengths are given in Å.

To understand the precise mechanism of enantioinduction, the stereochemically relevant steps in the reaction pathway were explored in detail. The study focused on the origin of selectivity for radical addition and cyclization processes. The starting point was chosen to be the radical II/olefin 12c complex (Fig. S13 in the ESI†). All structures of interest were first submitted to a thorough conformational search. The geometries were optimized in the gas phase using Gaussian 16 at the B3LYP-D3/6-31G** level.^51^ It was found that radical addition (II–12c → III) has low barriers of 9.4 to 12.5 kcal mol^−1^ for the four diasteromeric addition transition states (TSs), and the resulting radical cation intermediates III are all 14–22.5 kcal mol^−1^ downhill from complex II–12c. For the subsequent radical cyclization process establishing the stereogenic centres, four possible TSs were also considered: *RR*-TS2, *RS*-TS2, *SR*-TS2, and *SS*-TS2, each of which leads to a stereoisomer of cyclopentane radical cation intermediate IV, and their energy barriers are between 9.2 and 15.0 kcal mol^−1^. During the stepwise radical addition and cyclization processes, it was observed that CPA C3 acted as a bifunctional catalyst with double H-bonding interactions to promote the formation of the two new C–C bonds. To achieve more reliable results, further single-point energy corrections were performed for the four cyclization transition states with the SMD (tetrahydrofuran)-M062X/6-31+G(d,p) level based on the optimized structures.^50^ The lowest-energy pathway was calculated to be that involving *RR*-TS2 (major TS2), which was 2.1 kcal mol^−1^ lower in energy than *RS*-TS2 and 4.2 kcal mol^−1^ lower than *SS*-TS2. The *SR*-TS2 pathway was significantly higher in energy and thus was excluded from further analysis (6.6 kcal mol^−1^ higher in energy than *RR*-TS2). These results are consistent with the experimentally observed enantio- and diastereoselectivities (Fig. 4).

**Fig. 5:**
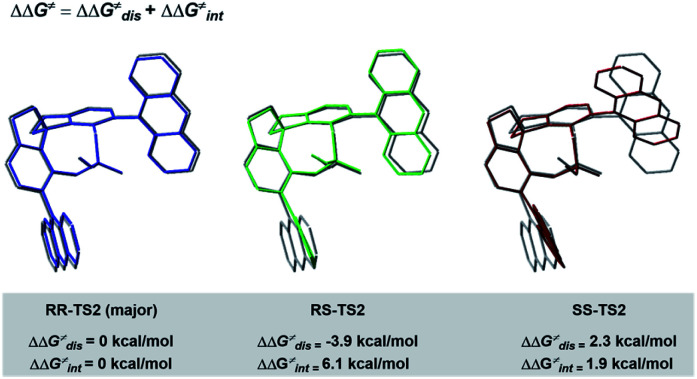
Distortion/interaction analyses of transition states.

To better comprehend the origin of the energy gap favouring formation of the *RR* enantiomer, we carried out a distortion/interaction activation strain analysis^52,53^ by considering C3 and radical intermediates as two fragments. As shown in Fig. 5, although the distortion energy of *RS*-TS2 was lower than that of *RR*-TS2, the interaction energy of *RR*-TS2 was more stabilizing, and this outweighs the energetic penalty for distortion. In addition, both interaction and distortion terms favour the major *RR*-TS2 transition state over *SS*-TS2. The computed results reveal that the interaction favours the major enantiomer *RR*-TS2 by 6.1 and 1.9 kcal mol^−1^ compared to *RS*-TS2 and *SS*-TS2, respectively. Thus, the interaction plays a dominant role in favouring *RR*-TS2. Furthermore, these interaction energy contributions to ΔΔ*G* were probed by NCI analysis^51^ (see Fig. S14 in the ESI†). Important noncovalent interactions between the catalyst and substrates are encircled in Fig. 4 (dotted line). The H-bonds O–H⋯O between the phosphate hydroxyl and the S=O oxygen of OTf and N–H⋯O between the substrate amidogen and P=O oxygen of the phosphate in *RR*-TS2 (1.72 and 1.72 Å) are stronger than those in *RS*-TS2 (1.77 and 1.79 Å) and *SS*-TS2 (1.73 and 2.12 Å). In addition, multiple C–H⋯F interactions (2.52, 2.76, 2.70, and 2.66 Å) between the CF_3_ of OTf and the anthryl of CPA were also found in the favoured *RR*-TS2, and these were weaker in the other transition states *RS*-TS2 (2.46, 2.77, and 3.09 Å) and *SS*-TS2 (2.62, 2.92, and 2.97 Å). Thus, the OTf substituent on the olefin plays a significant role in facilitating sufficient enantiocontrol by contributing to H-bonds of different strengths and multiple C–H⋯F bonds for interactions between the catalyst and substrate.

## Conclusions

In summary, we have developed redox-neutral, asymmetric [3 + 2] photocycloaddition reactions of *N*-aryl cyclopropylanilines with a variety of electron-rich and electron-neutral olefins *via* cooperative visible-light-driven photoredox and chiral H-bond catalysis. This transition metal-free method provides modular and efficient access to six kinds of biologically important enantioenriched cyclopentylamines with high yields, ees and drs in a fully atom-economical manner. The success is due to the introduction of suitable electron-withdrawing groups at specific positions of the olefins, which lowers the LUMO energy of the olefins to facilitate radical addition and embeds a H-bond acceptor to allow the chiral H-bonding catalyst to provide sufficient enantiocontrol of the subsequent asymmetric radical addition. In addition to accomplishing challenging [3 + 2] photocycloadditions of electron-rich olefins with cyclopropylanilines, this newly developed catalytic method in which olefins accept remote H-bonding by the chiral catalyst is compatible with electron-neutral 1,1-diarylethylenes; although two aryl groups are structurally similar, highly enantioselective constructions of elusive all-carbon quaternary stereocentres were accomplished. The preassembled functional groups that present H-bond donors can be conveniently removed, and, more importantly, can undergo simple chemical transformations to offer valuable derivatives, underscoring the promising utility of the methodology. Theoretical studies *via* DFT calculations demonstrated the mechanism of this unprecedented chiral H-bonding catalytic strategy. We anticipate that this work will inspire the pursuit of chiral H-bonding catalysis to achieve other important but formidable asymmetric radical-based chemical transformations.

## Data availability

General information, optimization of reaction conditions, general procedures, mechanistic studies, synthetic applications, characterization data, X-ray of products, and NMR spectra.

## Author contributions

Y. D., S. L., G. Z. and H. H. synthesized and characterized the compounds; Y. D., S. L. and G. Z. collected and analysed the spectroscopic data; S. C. performed DFT calculations; Z. J., X. Z. and S. C. wrote the manuscript. All authors discussed the results and commented on the manuscript. Z. J. directed the project.

## Conflicts of interest

There are no conflicts to declare.

## Supplementary Material

SC-013-D1SC07044D-s001

SC-013-D1SC07044D-s002
