# Supplementary material for: Asymmetric [3 + 2] photocycloadditions of cyclopropylamines with electron-rich and electron-neutral olefins
Source: Chem Sci. 2022 Mar 3;13(13):3787–95. doi: 10.1039/d1sc07044d (PMC8966714; doi:10.1039/d1sc07044d)
Supplement: SC-013-D1SC07044D-s001 [file SC-013-D1SC07044D-s001.pdf]

Electronic Supplementary Material (ESI) for Chemical Science.
This journal is © The Royal Society of Chemistry 2022

Supporting Information

Asymmetric [3 + 2] photocycloadditions of cyclopropylamines with electron-rich and electron-neutral olefins

Yating Dai,[1] Shuangshuang Liang,[2] Guangkuo Zeng,[2] Hongchun Huang,[2] Xiaowei Zhao,[2,*] Shanshan Cao,[1,*] and Zhiyong Jiang[1,2,*]

[†]*School of Chemistry and Chemical Engineering, Henan Normal University, Xinxiang, Henan, P. R. China 453007*

[‡]*International Scientific and Technological Cooperation Base of Chiral Chemistry, Henan University, Kaifeng, Henan, P. R. China 475004*

Table of Contents

1. General information

S3-S4

2. Optimization of reaction conditions

S5-S14

3. General experimental procedures

S15-S18

4. Mechanism studies

S19-S94

5. Synthetic applications

S95-S97

6. Determination of the absolute configurations

S98-S136

7. Characterization of adducts

S137-S214

8. Copies of NMR spectra

S215-S314

9. Other attempted substrates

S315

1. General information

General procedures and methods

Experiments involving moisture and/or air sensitive components were performed under a positive pressure of argon in oven-dried glassware equipped with a rubber septum inlet. Dried solvents and liquid reagents were transferred by oven-dried syringes or hypodermic syringe cooled to ambient temperature in a desiccator. Reactions mixtures were stirred in 10 mL sample vial with Teflon-coated magnetic stirring bars unless otherwise stated. Moisture in non-volatile reagents/compounds was removed in high *vacuo* by means of an oil pump and subsequent purging with nitrogen. Solvents were removed *in vacuo* under ~30 mmHg and heated with a water bath at 30–35 ^{o}C using rotary evaporator with aspirator. The condenser was cooled with running water at 0 ^{o}C.

All experiments were monitored by analytical thin layer chromatography (TLC). TLC was performed on pre-coated plates, 60 F_{254}. After elution, plate was visualized under UV illumination at 254 nm for UV active material. Further visualization was achieved by staining $Ce(SO_4)_2$ and anisaldehyde solution. For those using the aqueous stains, the TLC plates were heated on a hot plate.

Columns for flash chromatography (FC) contained *silica gel* 200–300 mesh. Columns were packed as slurry of *silica gel* in petroleum ether and equilibrated solution using the appropriate solvent system. The elution was assisted by applying pressure of about 2 atm with an air pump.

Instrumentations

Proton nuclear magnetic resonance (^{1}H NMR) and carbon NMR (^{13}C NMR) were recorded in $CDCl_3$ otherwise stated. Chemical shifts are reported in parts per million (ppm), using the residual solvent signal as an internal standard: $CDCl_3$ (^{1}H NMR: δ 7.26, singlet; ^{13}C NMR: δ 77.0, triplet). Multiplicities were given as: *s* (singlet), *d* (doublet), *t* (triplet), *q* (quartet), *quintet*, *m* (multiplets), *dd* (doublet of doublets), *dt* (doublet of triplets), and *br* (broad). Coupling constants (*J*) were recorded in Hertz (Hz). The number of proton atoms (*n*) for a given resonance was indicated by *n*H. The number of carbon atoms (*n*) for a given resonance was indicated by *n*C. HRMS (Analyzer: TOF) was reported in units of mass of charge ratio (m/z). Mass samples were dissolved in CH_3CN (HPLC Grade) unless otherwise stated.

Optical rotations were recorded on a polarimeter with a sodium lamp of wavelength 589 nm and reported as follows; 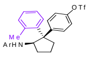 (c = g/100 mL, solvent). Melting points were determined on a melting point apparatus.

Enantiomeric excesses were determined by chiral High Performance Liquid Chromatography (HPLC) analysis. UV detection was monitored at 254 nm and 210 nm at the same time. HPLC samples were dissolved in HPLC grade isopropanol (IPA) unless otherwise stated.

Materials

All commercial reagents were purchased with the highest purity grade. They were used without further purification unless specified. All solvents used, mainly petroleum ether (PE) and ethyl acetate (EtOAc) were distilled. Anhydrous dichloromethane (DCM) and CH_3CN were freshly distilled from CaH_2 and stored under N_2 atmosphere. THF, CPME, MTBE, and Et_2O were freshly distilled from sodium/benzophenone before use. All compounds synthesized were stored in a −20 °C freezer and light-sensitive compounds were protected with aluminium foil.

2. Optimization of reaction conditions

Table S1. Control experiments for the reaction between 1e and 2a[a]

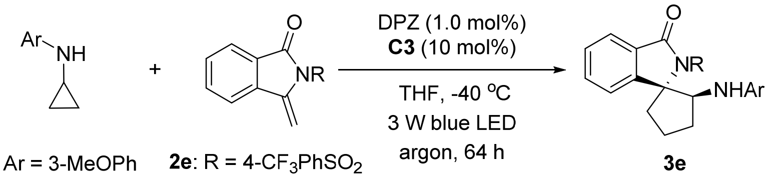

1a: Ar = 3-MeOPh **2e**: R = 4-CF$_3$PhSO$_2$ **3e**

entry	variation from the standard conditions	yield (%)[b]	ee (%)[c]	dr[d]
1	Rose Bengal instead of DPZ	75	93	>19:1
2	[Ru(bpy)$_3$]Cl$_2$ instead of DPZ	73	92	>19:1
3	toluene instead of THF	30	80	>19:1
4	CH_2Cl_2 instead of THF	45	90	>19:1
5	no **C3**	73	N.A.	1:2
6	no DPZ	0[e]	N.A.	N.A.
7	no light	0[e]	N.A.	N.A.
8	under air	0[f]	N.A.	N.A.

[a]Reactions were performed with **1e** (0.05 mmol) and **2a** (0.06 mmol). [b]Yields of isolated product **3e**. [c]Enantiomeric excesses were determined by HPLC analysis on a chiral stationary phase. [d]Determined by ^{1}H NMR analysis of the crude reaction mixture. [e]No reaction was observed. [f]**2a** was consumed, but **3e** was not obtained. bpy = 2,2'-bipyridine.

Table S2. Optimization of the reaction conditions with respect to linear electron-rich terminal olefins–part I[a]

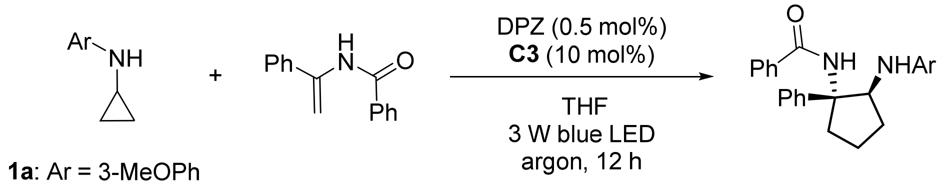

entry	*T* (°C)	conv. (%)[b]	ee (%)[c]	dr[d]
1	25	40	50	3:1
2	0	30	63	3.3:1
3	–15	20	69	4:1
4	–30	10	77	4:1
5	–50	<5	N.D.	N.D.

[a]Reactions were performed with **1a** (0.06 mmol), olefin (0.05 mmol), DPZ (0.5 mol%) in 3.0 mL THF. [b]Determined by TLC analysis of the UV (254 nm) and $Ce(SO_4)_2$ solution-staining signals between olefin and product. [c]Enantiomeric excesses were determined by HPLC analysis on a chiral stationary phase. [d]Determined by ^{1}H-NMR analysis of the crude reaction mixture.

Note: We used *N*-cyclopropyl-3-ethoxyaniline instead of *N*-cyclopropyl-3-methoxyaniline **1a** to react with several *N*-benzoyl *α*-aryl ethenamines using 0.5 mol% DPZ, 10 mol% SPINOL CPA **C3** in 3.0 mL THF as the solvent and at –40 °C. The reactions worked for 60 hours and the results are summarized as below:

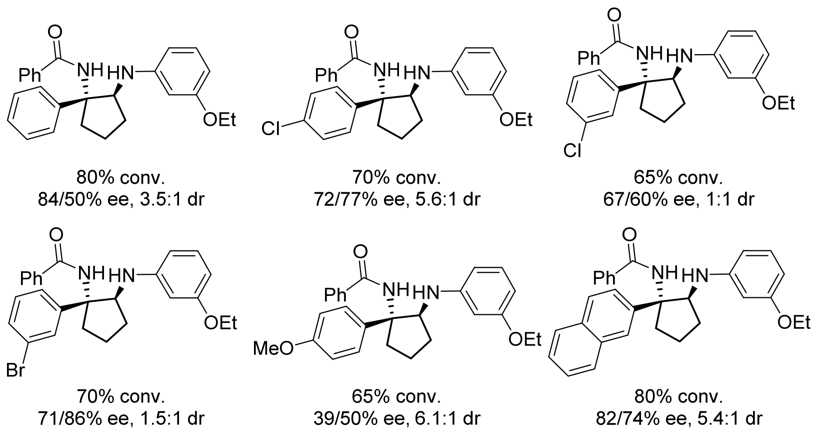

Comments: Given the unsatisfactory results obtained, we had to modify both *N*-protective groups of two substrates and further investigate reaction parameters to attain better results.

Table S3. Optimization of the reaction conditions with respect to *N*-protective *α*-phenyl ethenamine–part II[a]

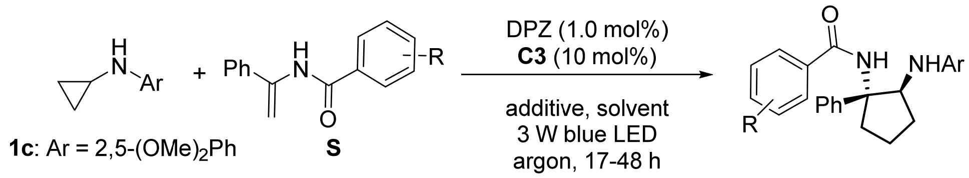

entry	**1c:S**	R	additive (mol%)	solvent (mL)	*T* (°C)	conv. (%)[b]	ee (%)[c]	dr[d]
1	1.2:1	H	--	THF (3.0)	25	25	77/50	1:1
2	1.2:1	H	--	THF (3.0)	–10	15	84/59	1.2:1
3	1.2:1	H	--	THF (3.0)	–20	trace	N.D.	N.D.
4	1.2:1	H	--	THF (3.0)	–30	N.R.	N.A.	N.A.
5	1:1.5	H	--	THF (3.0)	–10	25	85/59	1.5:1
6	1:2	H	--	THF (3.0)	–10	50	86/59	1.6:1
7	1:3	H	--	THF (3.0)	–10	50	86/59	1.6:1
8	1:2	H	--	Et_2O (3.0)	–10	30	88/79	1:2
9	1:2	H	--	CPME (3.0)	–10	10	87/77	1:1
10	1:2	H	--	MTBE (3.0)	–10	35	87/77	1:1
11	1:2	H	--	CH_2Cl_2 (3.0)	–10	10	80/64	1:1
12	1:2	H	--	toluene (3.0)	–10	N.R.	N.D.	N.D.
13	1:2	H	--	DME (3.0)	–10	50	76/25	1:3
14	1:2	H	TBAB (20)	THF (3.0)	–10	55	84/59	2.2:1
15	1:2	H	NaH_2PO_4 (20)	THF (3.0)	–10	45	86/61	1.6:1
16	1:2	H	Na_2HPO_4 (20)	THF (3.0)	–10	60	90/70	1.5:1
17	1:2	H	Na_2HPO_4 (20)	THF (3.0)	–20	50	91/72	2.3:1
18	1:2	H	Na_2HPO_4 (20)	THF (3.0)	–30	40	93/73	3.4:1
19	1:2	H	Na_2HPO_4 (20)	THF (3.0)	–40	30	91/75	3.2:1
20	1:2	H	Na_2HPO_4 (40)	THF (3.0)	–20	60	93/70	2.2:1
21	1:2	H	Na_2HPO_4 (60)	THF (3.0)	–20	60	93/75	1.9:1
22	1:2	H	Na_2HPO_4 (80)	THF (3.0)	–20	50	93/79	2.3:1
23[f]	1:2	H	Na_2HPO_4 (20)	THF (3.0)	–20	50	92/70	2.3:1
24	1:2	H	Na_2HPO_4 (20)	THF (1.0)	–20	70	96/80	3.5:1
25	1:2	H	Na_2HPO_4 (20)	THF (2.0)	–20	60	96/81	2.7:1
26	1:2	H	Na_2HPO_4 (20)	THF (5.0)	–20	25	95/75	1.7:1
27	1:2	2-naphthyl	Na_2HPO_4 (20)	THF (1.0)	–20	60	95/78	8:1
28	1:2	2-Cl	Na_2HPO_4 (20)	THF (1.0)	–20	70	95	>19:1

[a]0.05 mmol scale. [b]Determined by TLC analysis of the UV (254 nm) and $Ce(SO_4)_2$ solution-staining signals between olefin and product. [c]Enantiomeric excesses were determined by HPLC analysis on a chiral stationary phase. [d]Determined by ^{1}H-NMR analysis of the crude reaction mixture. [f]20 mol% **C3** was used. DME = 1,2-dimethoxyethane.

Table S4. Optimization of the reaction conditions with respect to arylethylenes–part I[a]

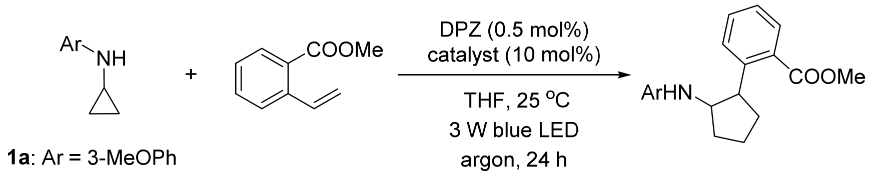

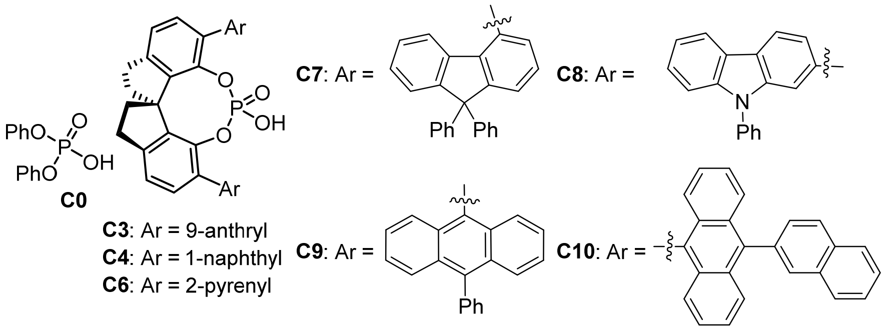

entry	catalyst	conv. (%)[b]	ee (%)[c]	dr[d]
1	--	50	--	1:1.5
2	**C0**	20	--	2:1
3	**C3**	30	30/25	2:1
4	**C4**	30	13/5	2:1
5	**C7**	20	–10/0	2:1
6	**C8**	20	2/2	2:1
7	**C6**	30	5/2	1:2
8	**C9**	30	7/5	1:2
9	**C10**	20	12/6	1:2
10[e]	**C3**	15	57/43	1:1

[a]Reactions were performed with **1a** (0.085 mmol), olefin (0.05 mmol), DPZ (0.5 mol%) in 1.0 mL THF at 25 °C. [b]Determined by TLC analysis of the UV (254 nm) and $Ce(SO_4)_2$ solution-staining signals between olefin and product. [c]Enantiomeric excesses were determined by HPLC analysis on a chiral stationary phase. [d]Determined by ^{1}H-NMR analysis of the crude reaction mixture. [e]T = –40 °C.

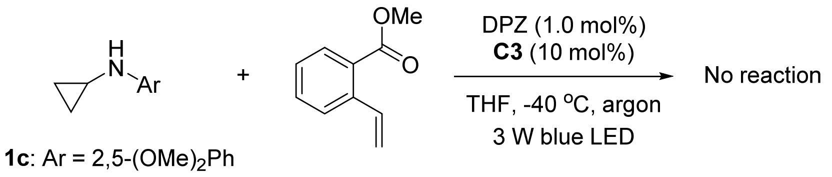

Table S5. Optimization of the reaction conditions with respect to arylethylenes–part II[a]

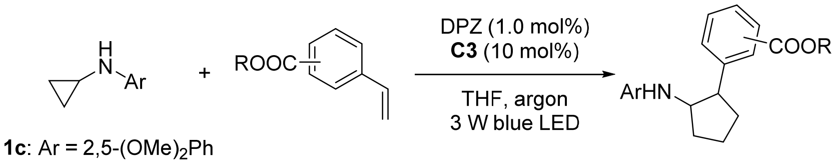

entry	COOR	*T* (°C)	*t* (h)	conv. (%)[b]	ee (%)[c]	dr[d]
1	3-COOMe	–40	24	10	26/47	1:1.6
2	4-COOMe	25	17	50	23/14	1:1
3	4-COOMe	–30	17	35	77/50	1:1
4	4-COOMe	–40	17	25	68/70	1:1
5	4-COOMe	–50	17	20	71/82	1:1
6	4-COO*t*Bu	–50	17	10	68/72	1:1
7	4-COOPh	–50	17	30	71/82	1:1
8	4-COOBn	–50	17	15	62/42	3:1

[a]Reactions were performed with **1c** (0.085 mmol), olefin (0.05 mmol), DPZ (0.5 mol%) in 1.0 mL THF. [b]Determined by TLC analysis of the UV (254 nm) and $Ce(SO_4)_2$ solution-staining signals between olefin and product. [c]Enantiomeric excesses were determined by HPLC analysis on a chiral stationary phase. [d]Determined by ^{1}H-NMR analysis of the crude reaction mixture.

Table S6. Optimization of the reaction conditions with respect to arylethylenes–part III[a]

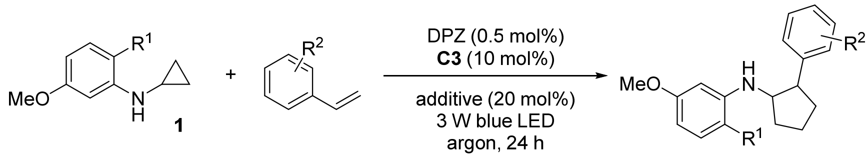

entry	R1	R2	additive	solvent (mL)	*T* (°C)	conv. (%)[b]	ee (%)[c]	dr[d]
1	H	2-OCOPh	--	THF (1.0)	25	25	24/9	2:1
2	H	2-OCOBn	--	THF (1.0)	25	30	28/25	2:1
3	H	2-OCO^{t}Bu	--	THF (1.0)	25	20	11/5	2:1
4	H	3-OCOMe	--	THF (1.0)	25	30	20/15	1:1
5	H	4-OCOMe	--	THF (1.0)	25	50	12/13	1.2:1
6	H	2-OCOMe	--	THF (1.0)	10	30	33/23	2:1
7	H	2-OCOMe	--	THF (1.0)	–20	20	40/25	2:1
8	H	2-OCOMe	--	THF (1.0)	–30	15	44/35	2:1
9	H	2-OCOMe	--	THF (1.0)	–40	10	46/37	3:1
10	OMe	2-OCOMe	--	THF (1.0)	–40	20	56/39	3:1
11	OMe	2-OCOMe	--	DCM (1.0)	–40	10	7/3	1:1
12	OMe	2-OCOMe	--	toluene (1.0)	–40	10	39/37	2:1
13	OMe	2-OCOMe	--	CH_3CN (1.0)	–40	10	17/19	2:1
14	OMe	2-OCOMe	--	CPME (1.0)	–40	10	12/20	2:1
15	OMe	2-OTf	--	THF (1.0)	–40	40	72	>19:1
16	OMe	3-OTf	--	THF (1.0)	–40	50	74/52	1.6:1
17	OMe	4-OTf	--	THF (1.0)	–40	50	17/13	3:1
18	OMe	2-OMs	--	THF (1.0)	–40	30	63/66	4:1
19	OMe	2-OSO_2Cy	--	THF (1.0)	–40	N.R.	N.A.	N.A.
20	OMe	2-OSO_2Bn	--	THF (1.0)	–40	30	70/68	4:1
21	OMe	2-OTs	--	THF (1.0)	–40	30	65	>19:1
22	OMe	2-OSO_2Ph(4-CF_3)	--	THF (1.0)	–40	30	66/35	10:1
23	OMe	2-OSO_2Ph(4-OMe)	--	THF (1.0)	–40	40	70/53	9:1
24	OMe	2-OSO_2Ph(3-OMe)	--	THF (1.0)	–40	50	70	>19:1
25	OMe	2-OSO_2Ph(2-OMe)	--	THF (1.0)	–40	20	66/83	4:1
26	OMe	2-OSO_2Ph(2-CF_3)	--	THF (1.0)	–40	5	31/25	12:1
27	OMe	2-OTf	Li_2CO_3	THF (1.0)	–40	40	3/3	1.5:1
28	OMe	2-OTf	Na_2CO_3	THF (1.0)	–40	40	49/30	1.5:1
29	OMe	2-OTf	K_2CO_3	THF (1.0)	–40	60	0/0	1.5:1
30	OMe	2-OTf	Cs_2CO_3	THF (1.0)	–40	35	0/13	3.5:1
31	OMe	2-OTf	NaF	THF (1.0)	–40	20	75	>19:1
32	OMe	2-OTf	NaCl	THF (1.0)	–40	35	50	>19:1
33	OMe	2-OTf	NaBr	THF (1.0)	–40	35	72	>19:1
34	OMe	2-OTf	NaH_2PO_4	THF (1.0)	–40	30	74	>19:1
35	OMe	2-OTf	Na_3PO_4	THF (1.0)	–40	50	2/0	4:1
36	OMe	2-OTf	$NaPF_6$	THF (1.0)	–40	N.R.	N.A.	N.A.
37	OMe	2-OTf	NaOAc	THF (1.0)	–40	30	1/0	3.9:1
38	OMe	2-OTf	LiF	THF (1.0)	–40	20	73	>19:1

39	OMe	2-OTf	KF	THF (1.0)	–40	40	12/4	4.9:1
40	OMe	2-OTf	MgF_2	THF (1.0)	–40	30	76	>19:1
41	OMe	2-OTf	CaF_2	THF (1.0)	–40	20	76/69	3.9:1
42	OMe	2-OTf	ZnF_2	THF (1.0)	–40	30	76	>19:1
43	OMe	2-OTf	KCl	THF (1.0)	–40	40	76	>19:1
44	OMe	2-OTf	KBr	THF (1.0)	–40	25	74	>19:1
45	OMe	2-OTf	K_2HPO_4	THF (1.0)	–40	30	61/11	15.7/1
46	OMe	2-OTf	KH_2PO_4	THF (1.0)	–40	20	75	>19:1
47	OMe	2-OTf	K_3PO_4	THF (1.0)	–40	30	76	>19:1
48	OMe	2-OTf	KPF_6	THF (1.0)	–40	30	53/14	13.3:1
49[f]	OMe	2-OTf	KCl, 18-crown-6	THF (1.0)	–40	50	76	>19:1
50	OMe	2-OTf	18-crown-6	THF (1.0)	–40	30	75	>19:1

[a]Reactions were performed with **1** (0.085 mmol) and olefin (0.05 mmol). [b]Determined by TLC analysis of the UV (254 nm) and $Ce(SO_4)_2$ solution-staining signals between olefin and product. [c]Enantiomeric excesses were determined by HPLC analysis on a chiral stationary phase. [d]Determined by ^{1}H-NMR or ^{19}F-NMR analysis of the crude reaction mixture. [f]After careful investigation, the amount of KCl was determined to 0.55 equivalents. For 18-crown-6, 20 mol% was used.

Comments: The use of 0.55 equiv. of KCl and 20 mol% 18-crown-6 as additives could afford corresponding product in 76% ee and >19:1 dr. Among them, KCl is responsible for improving the enantioselectivity, and 18-crown-6 can slightly increase the chemical conversion, likely due to the ability to improve the solubility of KCl in the reaction system. Given only moderate enantioselectivity obtained, the *N*-protecting group of cyclopropyl amine has to be further modified.

Table S7. Optimization of the reaction conditions with respect to arylethylenes–part IV[a]

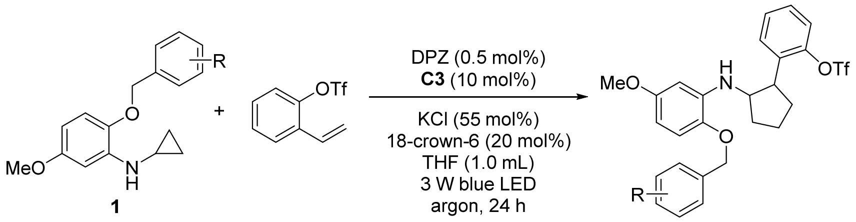

entry	R	*T* (°C)	conv. (%)[b]	ee (%)[c]	dr[d]
1	H	–40	40	80	>19:1
2	H	–50	20	85	>19:1
3	2-Me	–50	20	86	>19:1
4	2-Et	–50	20	88	>19:1
5	2-OMe	–50	20	78	>19:1
6	2-Cl	–50	20	81	>19:1
7	2,6-Me_2	–50	20	93	>19:1
8	3-Me	–50	20	82	>19:1
9	3-Cl	–50	50	70	>19:1
10	3-OMe	–50	20	66/83	11:1
11	4-Me	–50	10	31/25	16:1
12	4-Cl	–50	20	83	>19:1
13	4-F	–50	10	78	>19:1
14	4-CF_3	–50	20	75/60	9:1

[a]Reactions were performed with **1** (0.085 mmol) and olefin (0.05 mmol). [b]Determined by TLC analysis of the UV (254 nm) and $Ce(SO_4)_2$ solution-staining signals between olefin and product. [c]Enantiomeric excesses were determined by HPLC analysis on a chiral stationary phase. [d]Determined by ^{19}F-NMR analysis of the crude reaction mixture.

Comments: While excellent enantioselectivity was achieved (entry 7), the reaction was sluggish, and the conversion could not be increased through prolonging the reaction time.

Table S8. Optimization of the reaction conditions with respect to arylethylenes–part V[a]

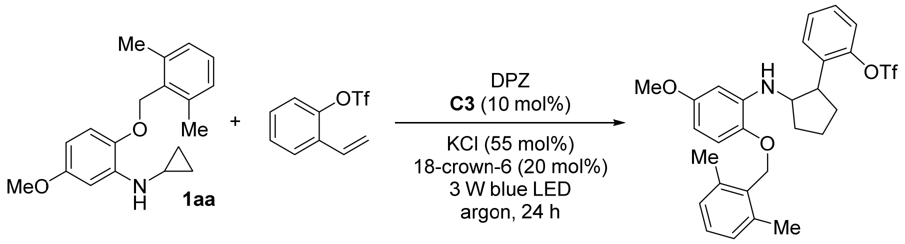

entry	mol% of DPZ	solvent (mL)	*T* (°C)	conv. (%)[b]	ee (%)[c]	dr[d]
1	0.5	THF (0.8)	–50	50	74	>19:1
2[e]	0.5	THF (1.0)	–50	40	78	>19:1
3[f]	0.5	THF (1.0)	–50	50	46	>19:1
4	0.5	DCM (1.0)	–50	20	30	>19:1
5	0.5	toluene (1.0)	–50	20	54	>19:1
6	0.5	MTBE (1.0)	–50	N.R.	N.A.	N.A.
7	0.5	CPME (1.0)	–50	N.R.	N.A.	N.A.
8	0.5	1,4-dioxane (1.0)	10	20	67	>19:1
9	0.5	mesitylene (1.0)	–50	10	64	>19:1
10	0.5	DME (1.0)	–50	40	90	>19:1
11	1.0	THF (1.0)	–50	20	88	>19:1
12	1.0	DME (1.0)	–50	50	87	>19:1
13	2.0	DME (1.0)	–50	60	85	>19:1
14[e]	2.0	DME (1.0)	–50	80	85	>19:1

[a]Reactions were performed with **1aa** (0.085 mmol) and olefin (0.05 mmol). [b]Determined by TLC analysis of the UV (254 nm) and $Ce(SO_4)_2$ solution-staining signals between olefin and product. [c]Enantiomeric excesses were determined by HPLC analysis on a chiral stationary phase. [d]Determined by ^{19}F-NMR analysis of the crude reaction mixture. [e]2 x 3W blue LEDs. [f]3 x 3W blue LEDs.

Table S9. Optimization of the reaction conditions with respect to 1,1-diaryl ethylenes[a]

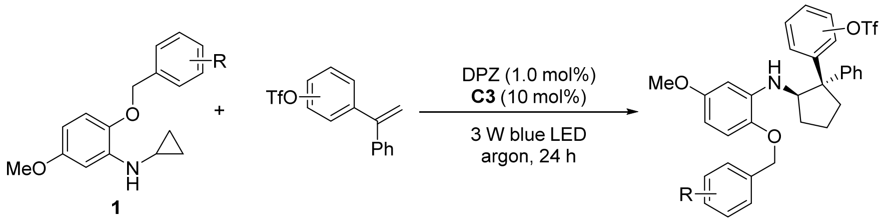

entry	R	position of OTf	solvent (mL)	*T* (°C)	conv. (%)[b]	ee (%)[c]	dr[d]
1[f]	2,6-Me2	*ortho*	DME (2.0)	25	5	N.A.	2.6:1
2	2,6-Me2	*ortho*	DME (2.0)	–50	<5	N.D.	N.D.
3	2,6-Me2	*ortho*	DME (2.0)	–10	5	97	>19:1
4	2,6-Me2	*ortho*	DME (2.0)	25	20	95	>19:1
5	2,6-Me2	*ortho*	THF (2.0)	25	15	92/35	5:1
6	2,6-Me2	*ortho*	toluene (2.0)	25	20	88	N.A.
7	2,6-Me2	*ortho*	DCM (2.0)	25	20	91	N.A.
8	2,6-Me2	*ortho*	$CHCl_3$ (2.0)	25	N.R.	N.A.	N.A.
9	2,6-Me2	*ortho*	Et_2O (2.0)	25	10	91	>19:1
10	2,6-Me2	*ortho*	MeCN (2.0)	25	20	87	>19:1
11	2,6-Me2	*ortho*	CPME (2.0)	25	20	87	9:1
12	2,6-Me2	*ortho*	MTBE (2.0)	25	20	91	>19:1
14	2,6-Me2	*ortho*	DME (2.0)	30	25	94	>19:1
15	2,6-Me2	*ortho*	DME (1.0)	35	30	93/11	12:1
16	2,6-Me2	*ortho*	DME (1.0)	40	40	93/5	4:1
17	2,6-Me2	*ortho*	DME (0.5)	35	50	93/57	12:1
18	H	*ortho*	DME (0.5)	35	30	91/76	6:1
19	H	*meta*	DME (0.5)	35	70	90/85	3:1
20	H	*para*	DME (0.5)	35	70	93/87	4:1

[a]Reactions were performed with **1** (0.085 mmol) and olefin (0.05 mmol). [b]Determined by TLC analysis of the UV (254 nm) and $Ce(SO_4)_2$ solution-staining signals between olefin and product. [c]Enantiomeric excesses were determined by HPLC analysis on a chiral stationary phase. [d]Determined by ^{19}F-NMR analysis of the crude reaction mixture. [e]In the absence of **C3**.

Comments: For the olefins with *meta*-OTf and *para*-OTf on the aromatic ring (entries 19–20), when using *N*-protecting cyclopropyl amine **1aa** (entries 1–17) to react with them, both enantioselectivity and diastereoselectivity of products were deteriorated slightly. Meanwhile, although the diastereoselectivity of using the *ortho*-OTf olefin (entry 17) is better than that of other olefins (entries 19–20), the reaction is remarkably slower than the latter two chemical transformations.

3. General experimental procedures

(1)

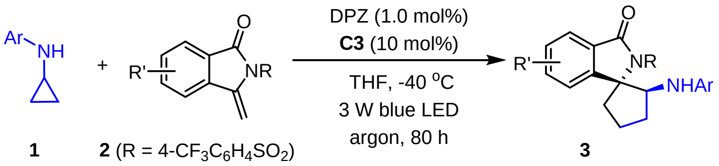

70.0 μL (0.001 mmol, 0.01 equiv.) of DPZ solution (1.0 mg of DPZ in 200 μL of toluene) was added into a 10 mL Schlenk tube, and then solvent was removed in *vacuo*.

For **3e–3u**: **1** (0.12 mmol, 1.2 equiv.), **2** (0.10 mmol, 1.0 equiv), **C3** (0.01 mmol, 0.1 equiv.) and THF (2.0 mL) were sequentially added, degassed three times by freeze-pump-thaw method. The reaction mixture was stirred under an argon atmosphere at −40 °C. Then irradiated by a 3 W blue LED (λ = 450−455 nm) for another 80 h. The reaction mixture was directly loaded onto a short *silica gel* column, followed by gradient elution with petroleum ether/ethyl acetate (50/1–3/1 ratio). Removing the solvent in *vacuo*, afforded products **3e−3u**.

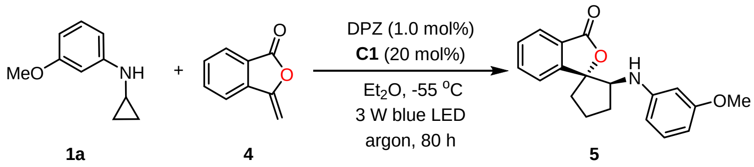

For **5**: **1a** (0.12 mmol, 1.2 equiv.), **4** (0.10 mmol, 1.0 equiv), **C1** (0.02 mmol, 0.2 equiv.) and Et_2O (6.0 mL) were sequentially added, degassed three times by freeze-pump-thaw method. The reaction mixture was stirred under an argon atmosphere at −55 °C. Then irradiated by a 3 W blue LED (λ = 450−455 nm) for another 80 h. The reaction mixture was directly loaded onto a short *silica gel* column, followed by gradient elution with petroleum ether/ethyl acetate (50/1–3/1 ratio). Removing the solvent in *vacuo*, afforded products **5**.

(2)

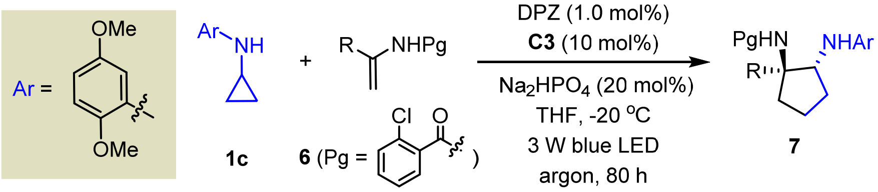

70.0 μL (0.001 mmol, 0.01 equiv.) of DPZ solution (1.0 mg of DPZ in 200 μL of toluene) was added into a 10 mL Schlenk tube, and then solvent was removed in *vacuo*.

For **7a–7m**: **1c** (0.10 mmol, 1.0 equiv.), **6** (0.20 mmol, 2.0 equiv), **C3** (0.01 mmol, 0.1 equiv.), NaH_2PO_4 (0.02 mmol, 0.2 equiv.) and THF (2.0 mL) were sequentially added, degassed three times by freeze-pump-thaw method. The reaction mixture was stirred under an argon atmosphere at −20 °C. Then irradiated by a 3 W blue LED (λ = 450−455 nm) for another 80 h. The reaction mixture was directly loaded onto a short *silica gel* column, followed by gradient elution with petroleum ether/ethyl acetate (50/1–3/1 ratio). Removing the solvent in *vacuo*, afforded products **7a−7m**.

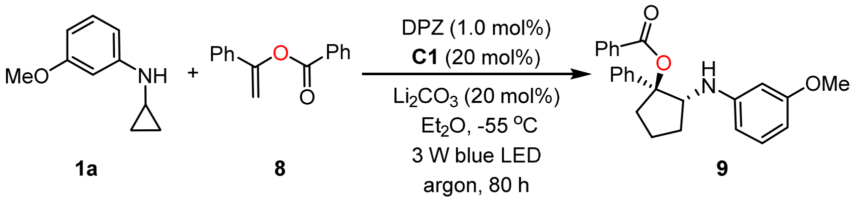

For **9**: **1a** (0.12 mmol, 1.2 equiv.), **8** (0.10 mmol, 1.0 equiv), **C1** (0.02 mmol, 0.2 equiv.), Li_2CO_3 (0.02 mmol, 0.2 equiv.) and Et_2O (4.0 mL) were sequentially added, degassed three times by freeze-pump-thaw method. The reaction mixture was stirred under an argon atmosphere at −55 °C. Then irradiated by a 3 W blue LED (λ = 450−455 nm) for another 80 h. The reaction mixture was directly loaded onto a short *silica gel* column, followed by gradient elution with petroleum ether/ethyl acetate (50/1–3/1 ratio). Removing the solvent in *vacuo*, afforded products **9**.

(3)

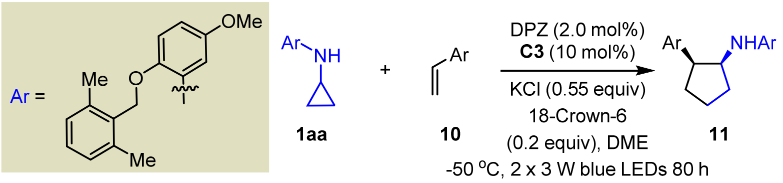

140.0 μL (0.002 mmol, 0.02 equiv.) of DPZ solution (1.0 mg of DPZ in 200 μL of toluene) was added into a 10 mL Schlenk tube, and then solvent was removed in *vacuo*.

For **11a–11i**: **1aa** (0.17 mmol, 1.7 equiv.), **10** (0.10 mmol, 1.0 equiv), **C3** (0.01 mmol, 0.1 equiv.), KCl (0.055 mmol, 0.55 equiv.), 18-Crown-6 (0.02 mmol, 0.2 equiv.) and DME (2.0 mL) were sequentially added, degassed three times by freeze-pump-thaw method. The reaction mixture was stirred under an argon atmosphere at −50 °C. Then irradiated by a 2 × 3 W blue LED (λ = 450−455 nm) for another 80 h. The reaction mixture was directly loaded onto a short *silica gel* column, followed by gradient elution with petroleum ether/ethyl acetate

(100/1–30/1 ratio). Removing the solvent in *vacuo*, afforded products **11a−11i**.

(4)

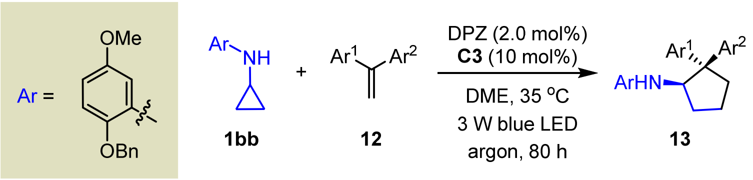

70.0 μL (0.001 mmol, 0.01 equiv.) of DPZ solution (1.0 mg of DPZ in 200 μL of toluene) was added into a 10 mL Schlenk tube, and then solvent was removed in *vacuo*.

For **13a–c, 13k, 13l, 13z**: **1bb** (0.17 mmol, 1.7 equiv.), **10** (0.10 mmol, 1.0 equiv), **C3** (0.01 mmol, 0.1 equiv.) and DME (1.0 mL) were sequentially added, degassed three times by freeze-pump-thaw method. The reaction mixture was stirred under an argon atmosphere at 35 °C. Then irradiated by a 3 W blue LED (λ = 450−455 nm) for another 80 h. The reaction mixture was directly loaded onto a short *silica gel* column, followed by gradient elution with petroleum ether/ethyl acetate (100/1–30/1 ratio). Removing the solvent in *vacuo*, afforded products **13a–c, 13k, 13l, 13z**.

140.0 μL (0.002 mmol, 0.02 equiv.) of DPZ solution (1.0 mg of DPZ in 200 μL of toluene) was added into a 10 mL Schlenk tube, and then solvent was removed in *vacuo*.

For **13d, 13m, 13n, 13u–y**: **1bb** (0.17 mmol, 1.7 equiv.), **10** (0.10 mmol, 1.0 equiv), **C3** (0.01 mmol, 0.1 equiv.) and DME (1.0 mL) were sequentially added, degassed three times by freeze-pump-thaw method. The reaction mixture was stirred under an argon atmosphere at 35 °C. Then irradiated by a 3 W blue LED (λ = 450−455 nm) for another 80 h. The reaction mixture was directly loaded onto a short *silica gel* column, followed by gradient elution with petroleum ether/ethyl acetate (100/1–30/1 ratio). Removing the solvent in *vacuo*, afforded products **13d, 13m, 13n, 13u–y**.

70.0 μL (0.001 mmol, 0.01 equiv.) of DPZ solution (1.0 mg of DPZ in 200 μL of toluene) was added into a 10 mL Schlenk tube, and then solvent was removed in *vacuo*.

For **13e–j**: **1bb** (0.17 mmol, 1.7 equiv.), **10** (0.10 mmol, 1.0 equiv), **C3** (0.01 mmol, 0.1 equiv.) and DME (1.0 mL) were sequentially added, degassed three times by freeze-pump-thaw method. The reaction mixture was stirred under an argon atmosphere at 25 °C. Then irradiated by a 3 W blue LED (λ = 450−455 nm) for another 80 h. The reaction mixture was directly

loaded onto a short *silica gel* column, followed by gradient elution with petroleum ether/ethyl acetate (100/1–30/1 ratio). Removing the solvent in *vacuo*, afforded products **13e–j**.

140.0 μL (0.002 mmol, 0.02 equiv.) of DPZ solution (1.0 mg of DPZ in 200 μL of toluene) was added into a 10 mL Schlenk tube, and then solvent was removed in *vacuo*.

For **13o–t**: **1bb** (0.17 mmol, 1.7 equiv.), **10** (0.10 mmol, 1.0 equiv), **C3** (0.01 mmol, 0.1 equiv.) and DME (1.0 mL) were sequentially added, degassed three times by freeze-pump-thaw method. The reaction mixture was stirred under an argon atmosphere at 25 ^{o}C. Then irradiated by a 3 W blue LED (λ = 450−455 nm) for another 80 h. The reaction mixture was directly loaded onto a short *silica gel* column, followed by gradient elution with petroleum ether/ethyl acetate (100/1–30/1 ratio). Removing the solvent in *vacuo*, afforded products **13o–t**.

4. Mechanism studies

(1) Linear effect experiments

These reactions were conducted according to the general procedure (see Section 3.1 in SI). Chiral HPLC analyses gave linear effect data (see page S209−212 for the detailed HPLC results).

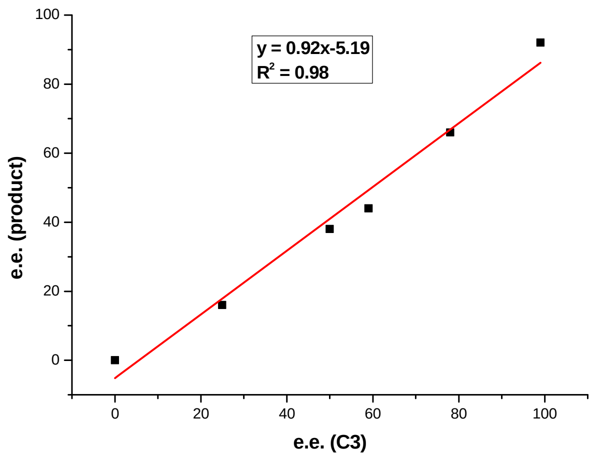

Fig. S1. Relationship between ee values of **C3** and **3e.**

Comments: From the results that determine a linear correlation between ee of CPA **C3** and ee of **3e** (Fig. S1), it could conclude that only a single molecule of the chiral phosphate is involved in the C−C bond-forming step.

(2) Emission quenching experiments

The Luminescence Quenching Experiments of DPZ (excitation wavelength = 448 nm)

Emission intensities were recorded on a spectrofluorometer. **DPZ** solution was excited at 448 nm and the emission intensity at 525 nm was observed. A solution of **DPZ** (1.0×10^{-5} M) was added to the appropriate amount of quencher in 3.0 mL volumetric flask under N_2. The solution was transferred to a 3.0 mL quartz cell and the emission spectrum of the sample was collected.

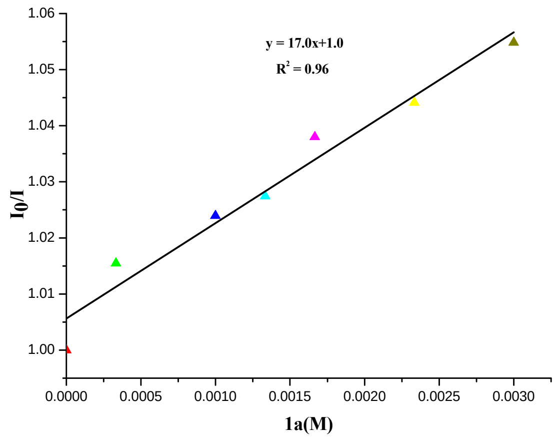

Fig. S2. Stern–Volmer quenching experiment of DPZ and **1a**.

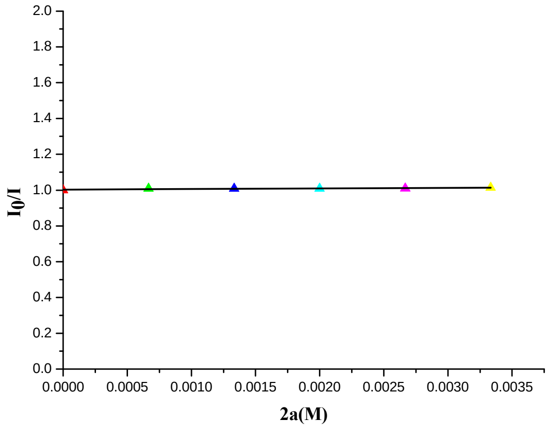

Fig. S3. Stern–Volmer quenching experiment of DPZ and **2e**. No quenching observed.

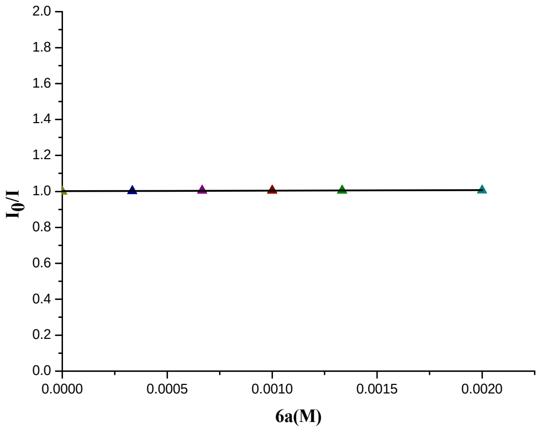

Fig. S4. Stern–Volmer quenching experiment of DPZ and **6a**. No quenching observed.

(3) Cyclic voltammetry measurement

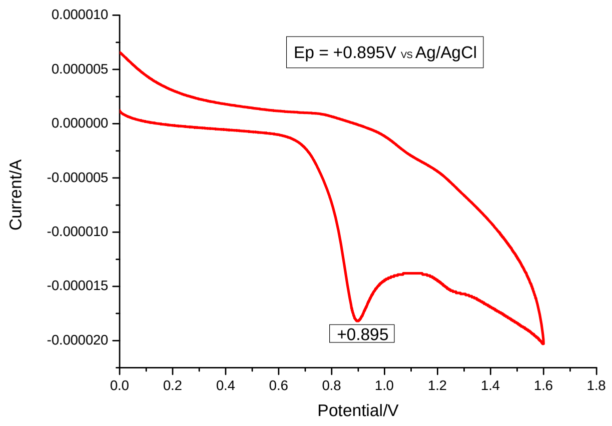

Fig. S5. Cyclic voltammogram of **1a** in MeCN.

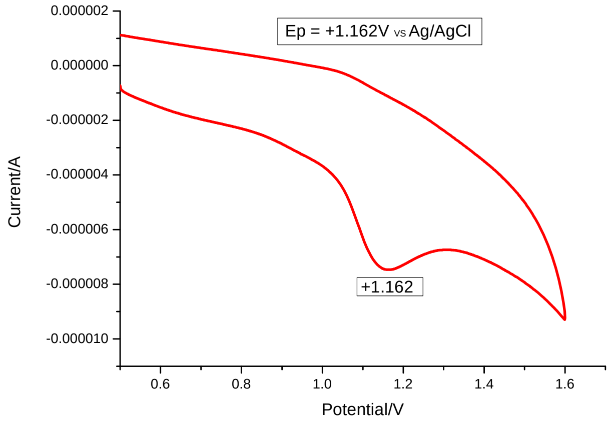

Fig. S6. Cyclic voltammogram of **2e** in MeCN.

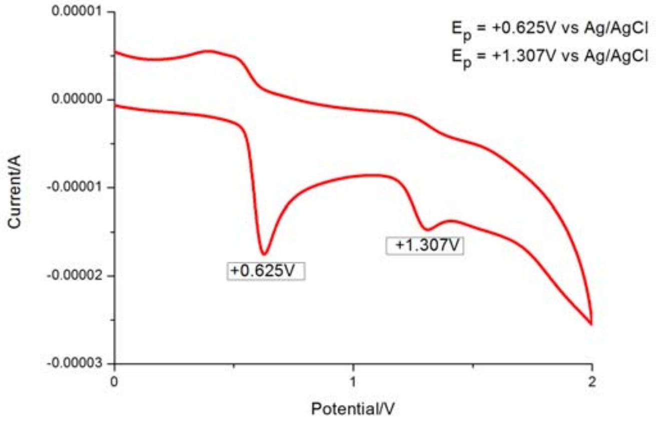

Fig. S7. Cyclic voltammogram of **1aa** in MeCN.

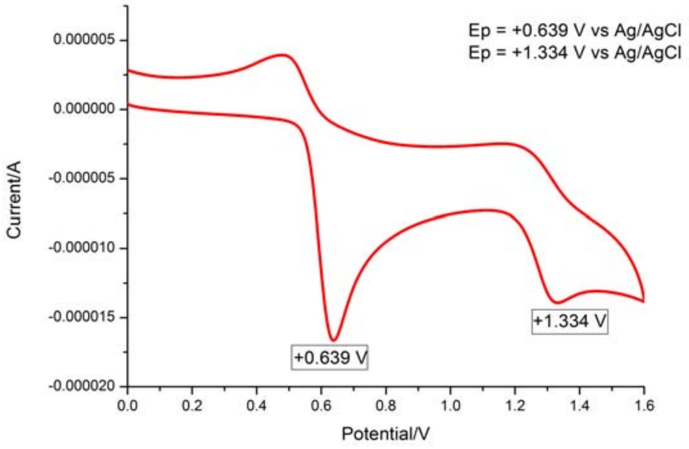

Fig. S8. Cyclic voltammogram of **4a** in MeCN.

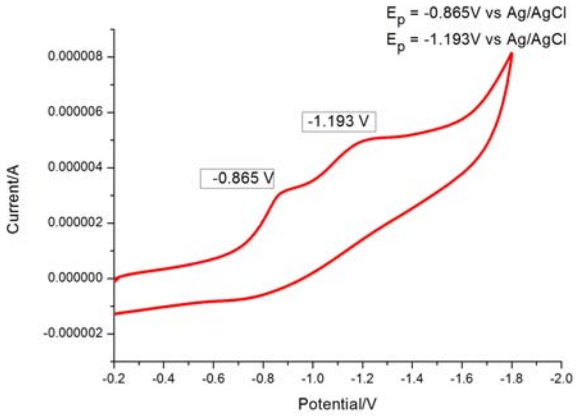

Fig. S9. Cyclic voltammogram of **5a** in MeCN.

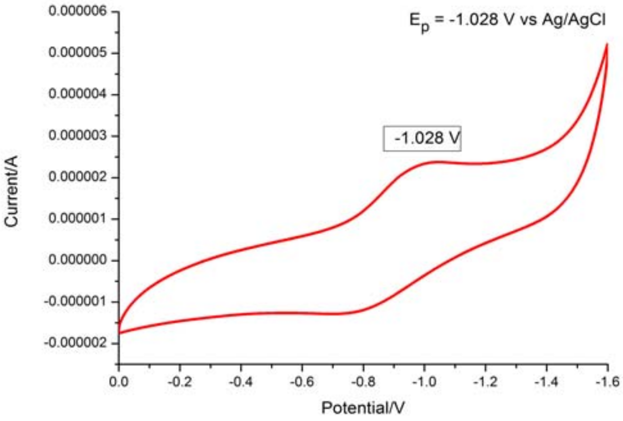

Fig. S10. Cyclic voltammogram of **10a** in MeCN.

(4) Quantum yield measurement (Blue LED)

Determination of the light intensity at 445 nm:

The photon flux of the spectrophotometer was determined by standard ferrioxalate actinometry. A 0.15 M solution of ferrioxalate was prepared by dissolving 2.21 g of potassium ferrioxalate hydrate in 30 mL of 0.05 M H_2SO_4. A buffered solution of phenanthroline was prepared by dissolving 50 mg of phenanthroline and 11.25 g of sodium acetate in 50 mL of 0.5M H_2SO_4. Both solutions were stored in the dark. To determine the photon flux of the spectrophotometer, 6.0 mL of the ferrioxalate solution was placed in a cuvette and irradiated for 360.0 seconds at λ = 445 nm with an emission slit width at 10.0 nm. After irradiation, 3.0 mL irradiated solution of the ferrous ions and 0.6 mL of the phenanthroline solution was added to the cuvette. The solution was then allowed to rest for 1 h to allow the ferrous ions to completely coordinate to the phenanthroline. The absorbance of the solution was measured at 510 nm. A non-irradiated sample was also prepared and the absorbance at 510 nm measured. Conversion was calculated using eq 1.

$$\text{mol Fe}^{2+} = \frac{\text{H}_1 \times \text{H}_3 \times \text{? H(510 甐)}}{10^3 \times \text{H}_2 \times \text{H} \times \text{H(510 甐)}} \quad (1)$$

$$= \frac{6 \times 3.6 \times 0.959}{10^3 \times 3 \times 1 \times 11100} = 6.2277 \times 10^{-7} \text{ 甐HI}$$

Where V_1 is the irradiated volume (6 mL), V_2 is the aliquot of the irradiated solution taken for the determination of the ferrous ions (3 mL), V_3 is the final volume after complexation with phenanthroline (3.6 mL), l is the optical path-length of the irradiation cell (1 cm), ΔA(510nm) is the optical difference in absorbance between the irradiated solution and that taken in the dark, ε(510nm) is that of the complex $Fe(phen)_3^{2+}$ (11100 L mol^{-1} cm^{-1}). The photon flux can be calculated using eq 2.

$$\text{photon flux} = \frac{\text{甐HF甐}^{2+}}{\text{H} \cdot \text{H} \cdot \text{H}} \quad (2)$$

$$= \frac{6.2277 \times 10^{-6} \text{ 甐HF}}{0.92 \times 360 \text{ H} \times 0.9968}$$

$$= 1.8864 \times 10^{-9} \text{ 甐甐甐BED/H}$$

Where ϕ is the quantum yield for the feeerrioxalate actinometer (0.92 at λ = 445nm), t is the time (360.0 s), and f is the fraction of light absorbed at λ = 445nm (0.9968, *vide infra*). The

photon flux was calculated (average of three experiments) to be 1.8864 $\times 10^{-9}$ einstein s^{-1}.

Determination of fraction of light absorbed at 445 nm for the ferrioxalate solution:

The absorbance of the above ferrioxalate solution at 445 nm was measured to be 2.50. The fraction of light absorbed (f) by this solution was calculated using eq 3, where A is the measured absorbance at 445 nm.

$$f = 1 - 10^{-H} \quad (3)$$

$$= 1 - 10^{-2.50} = 0.9968$$

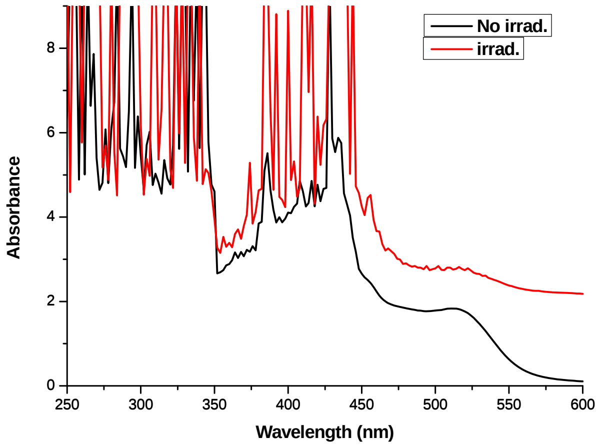

Fig. S11. Absorption spectra of the irradiation experiment and non-irradiation experiment

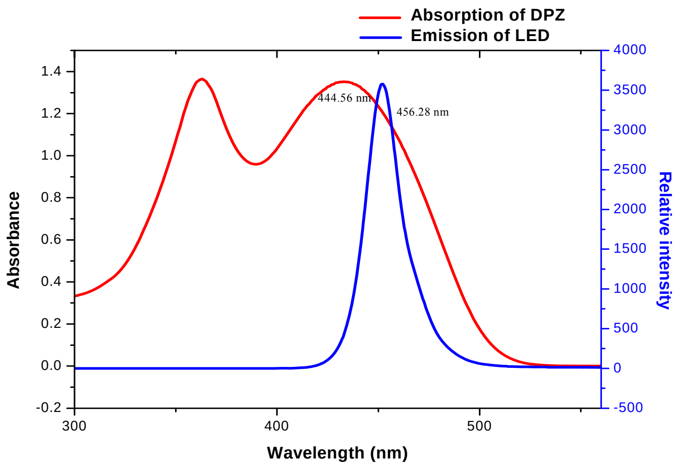

Fig. S12. Absorption spectra of 0.005 M solution of DPZ in THF

Determination of the reaction quantum yield

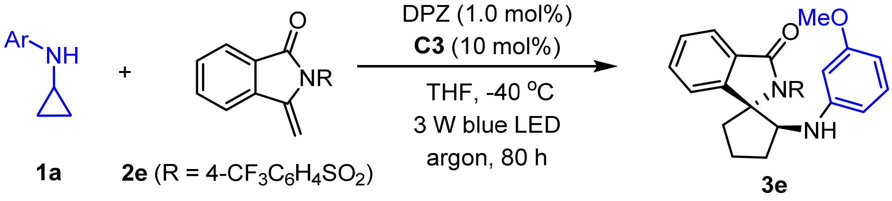

The reaction mixture was stirred and irradiated by 3W Blue LED (λmax = 445 nm) for 12 h (43200 s). The yield of product was determined from the isolated compounds following chromatographic purification. The yield of **3e** was determined to be 22% (0.022×10^{-3} mol of **3e**). The reaction quantum yield (Φ) was determined using eq 4 where the photon flux is 1.8864×10^{-9} einstein s^{-1} (determined by actinometry as described above), t is the reaction time (43200 s) and f is the fraction of incident light absorbed by the catalyst, determined using eq 3. An absorption spectrum of DPZ (0.005 M) gave an absorbance value of 1.296 at 445 nm (figure S2), indicating that the fraction of light absorbed by the photocatalyst (f) is 0.949.

$$F = \frac{\text{[illegible]}}{\text{[illegible]} \times H \times H} \qquad (4)$$

$$12\ h: F = \frac{0.022 \times 10^{-3}\ \text{[illegible]}}{(1.8864 \times 10^{-9}\ \text{einstein}\ s^{-1}) \times 43200\ H \times 0.949} = 0.284$$

The reaction quantum yield (Φ) was calculated to be 0.284.

(5) Computation Details

All the calculations in this work were performed on the basis of density functional theory (DFT) in the Gaussian G16 package (Revision B.01).[1] Geometry optimizations and frequencies were calculated with the B3LYP,[2] dispersion-corrected with the D3 version of Grimme's dispersion with Becke-Johnson damping (B3LYP-D3(BJ))[3] and a basis set of 6-31G(d, p).[4] All of the optimized geometries had been characterized as minima (zero imaginary frequencies) or transition state structures (a single imaginary frequency) at the same level of theory. Intrinsic reaction coordinate (IRC)[5] calculations were also carried out to inspect whether each of the transition structures actually connected the proposed reactant and product. For the four key transition states, the solvent tetrahydrofuran (Ɛ = 7.43) was evaluated by single-point energy calculation with SMD solvation model[6] using M06-2X

functional method[7] on the basis sets of 6-31+G(d, p). Noncovalent interactions (NCI) analysis[8] was employed to gain more insights into the important noncovalent interactions that are present in the key transition states. The key 3D structures were prepared using the CYL view visualization program.[9]

5.1 The radical addition and cyclization processes of substrate olefin 12c

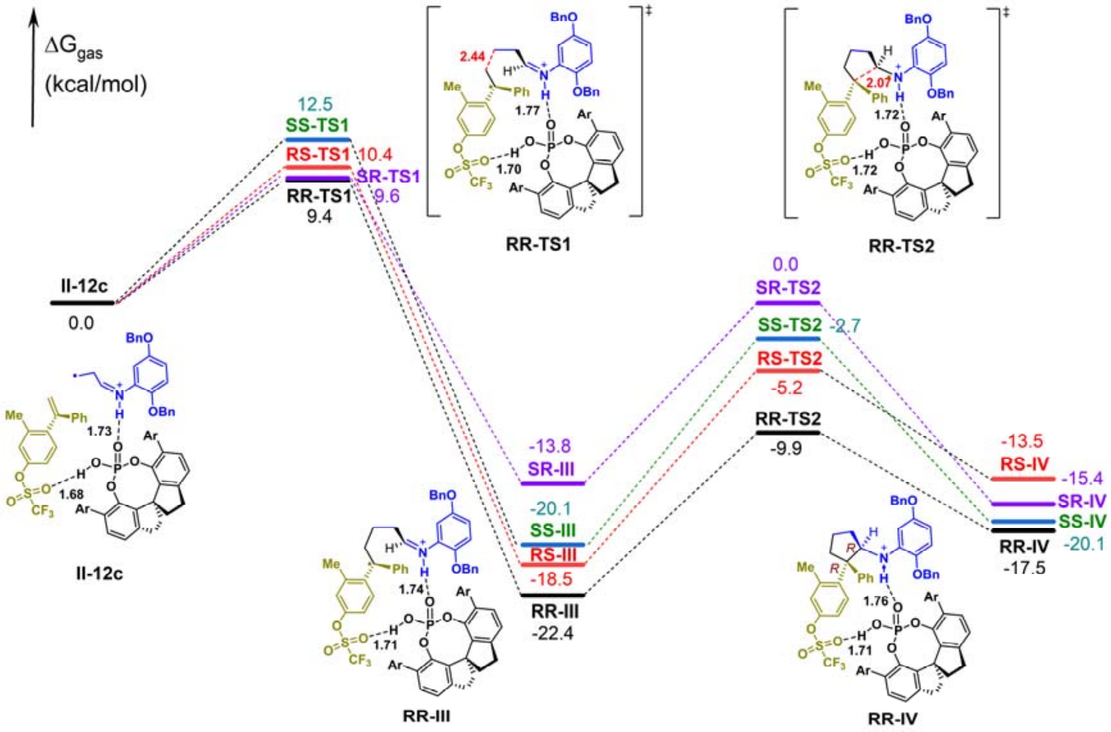

Fig. S13. Free energy profiles for the radical addition and cyclization processes calculated with CPA catalyst. For clarify, only the structures of RR-pathway are shown. Computational method: B3LYP-D3/6-31G(d,p) level of theory in gas phase.

5.2 NCI analysis for the two transition states

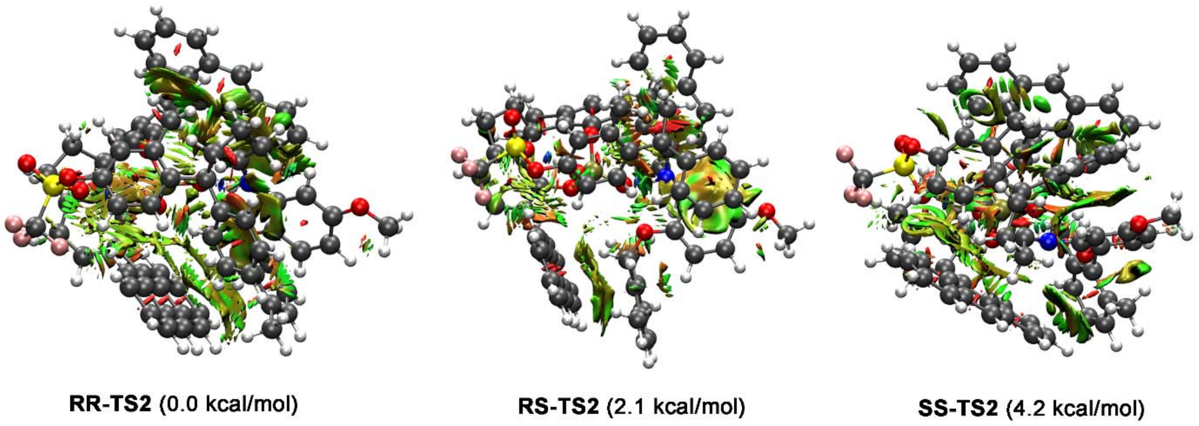

Fig. S14. Transition states for the major **RR-TS2**, minor **RS-TS2** and **SS-TS2** enantiomers featuring multiple noncovalent interactions (hydrogen bonds, C-H···F, C-H···π and π···π

interactions) (blue, strongly attractive; green, weakly attractive; red, strongly repulsive). Computational method: SMD//M062X/6-31+G(d,p)//B3LYP-D3/6-31G(d,p) level of theory in solvent tetrahydrofuran.

5.3 NPA charges on olefins

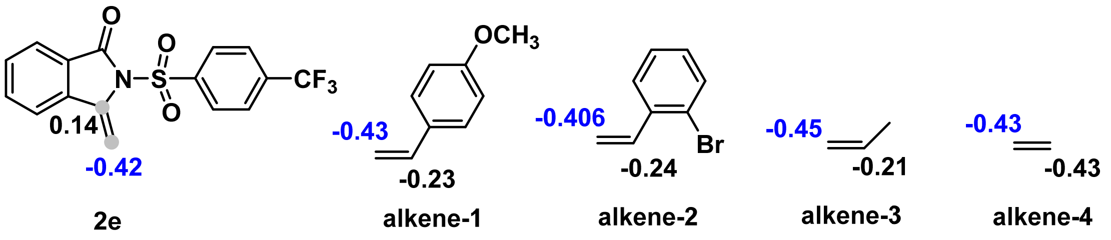

Fig. S15. Computed skeletons of five types of olefins.

Natural population analysis (NPA) charge analysis showed that the charge on the terminal carbon in **2e** (–0.42) is similar with the charge on the terminal carbon (–0.43) in electron-rich **alkene-1**, of which the poor reactivity with cyclopropylanilines as the reaction partners has been shown by Zheng and co-workers (ref. 19 in the manuscript).

5.4 The cascade radical addition and cyclization process of the reaction of 2d

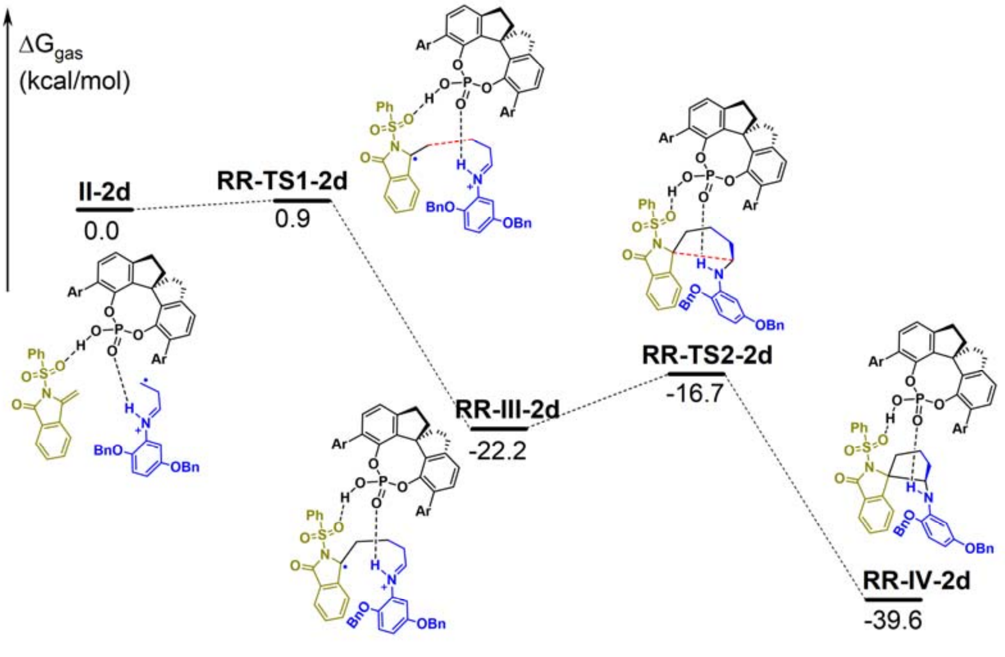

Fig. S16. Free energy profiles for the radical addition and cyclization processes of the substrate olefin **2d**.

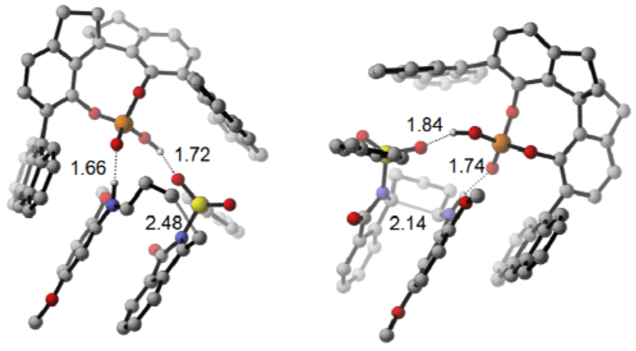

RR-TS1-2d **RR-TS2-2d**

Fig. S17. Computed structures of the two key transition states.

To get more information about the mode of sterecontrol of the electron-rich olefins bearing heteroatom-containing and electron-withdrawing as protect groups, the radical addition and cyclization process of the substrate **2d** was investigated by DFT calculations at B3LYP-D3/6-31G(d,p) level of theory. The free energy profile for the pathway leading to favored stereoisomer product is shown in Figure S16. The energy barriers of radical addition and cyclization processes were 0.9 and 5.5 kcal/mol. DFT calculations indicate that the H-bonds O-H···O between the phosphate hydroxyl and the S=O oxygen of OTf and N-H···O between the substrate amidogen and P=O oxygen of the phosphate were present in both two transition states **RR-TS1-2d** and **RR-TS2-2d** (Fig. S17), which plays a significant role in facilitating sufficient enantiocontrol.

5.5 Reference

1. Frisch, M. J.; Trucks, G. W.; Schlegel, H. B.; Scuseria, G. E.; Robb, M. A.; Cheeseman, J. R.; Scalmani, G.; Barone, V.; Mennucci, B.; Petersson, G. A.; Nakatsuji, H.; Caricato, M.; Li, X.; Hratchian, H. P.; Izmaylov, A. F.; Bloino, J.; Zheng, G.; Sonnenberg, J. L.; Hada, M.; Ehara, M.; Toyota, K.; Fukuda, R.; Hasegawa, J.; Ishida, M.; Nakajima, T.; Honda, Y.; Kitao, O.; Nakai, H.; Vreven, T.; Montgomery, J. A., Jr.; Peralta, J. E.; Ogliaro, F.; Bearpark, M.; Heyd, J. J.; Brothers, E.; Kudin, K. N.; Staroverov, V. N.; Kobayashi, R.; Normand, J.; Raghavachari, K.; Rendell, A.; Burant, J. C.; Iyengar, S. S.; Tomasi, J.; Cossi, M.; Rega, N.; Millam, N. J.; Klene, M.; Knox, J. E.; Cross, J. B.; Bakken, V.; Adamo, C.; Jaramillo, J.; Gomperts, R.; Stratmann, R. E.; Yazyev, O.; Austin, A. J.; Cammi, R.; Pomelli, C.; Ochterski, J. W.; Martin, R. L.; Morokuma, K.; Zakrzewski, V. G.; Voth, G. A.; Salvador, P.; Dannenberg, J. J.; Dapprich, S.; Daniels, A. D.; Farkas, Ö.; Foresman, J. B.; Ortiz, J. V.; Cioslowski, J.; Fox, D. J. *Gaussian 16, Revision B.01*; Gaussian, Inc.: Wallingford, CT, 2016.

2. (a) Becke, A. D. *Phys. Rev. A* **1988**, *38*, 3098–3100. (b) Becke, A. D. *J. Chem. Phys.* **1993**, *98*, 5648-5652. (c) Lee, C.; Yang, W.; Parr, R. G. *Phys. Rev. B* **1988**, *37*, 785–789.
3. Grimme, S.; Antony, J.; Ehrlich, S.; Krieg, H. *J. Chem. Phys.* **2010**, *132*, 154104.
4. Hehre, W. J.; Ditchfield, R.; Pople, J. A. *J. Chem. Phys.* **1972**, *56*, 2257–2261.
5. (a) Fukui, K. A. Formulation of the Reaction Coordinate. Formulation of the reaction coordinate. *J. Phys. Chem.* **1970**, *74*, 4161–4163. (b) Fukui, K. The Path of Chemical Reactions-The IRC Approach. *Acc. Chem. Res.* **1981**, *14*, 363–368.
6. Marenich, A. V.; Cramer, C. J.; Truhlar, D. G. *J. Phys. Chem. B* **2009**, *113*, 6378–6396.
7. (a) Zhao, Y.; Truhlar, D. G. *Theor. Chem. Acc.* **2008**, *120*, 215–241; (b) Zhao, Y.; Truhlar, D. G. *Acc. Chem. Res.* **2008**, *41*, 157–167.
8. Contreras-García, J.; Johnson, E. R.; Keinan, S.; Chaudret, R.; Piquemal, J.-P.; Beratan, D. N.; Yang, W. *J. Chem. Theory Comput.* **2011**, *7*, 625−632.
9. Legault, C. Y. *CYLview*, 1.0b; Université de Sherbrooke: Canada, **2009**; http://www.cylview.org.

5.6 Cartesian coordination and energies for the all the calculated species were performed at the B3LYP-D3/6-31G(d,p) level of theory in gas phase.

II

Sum of electronic and thermal Free Energies= -4858.932174

C	-2.42288800	-3.14924300	-2.11543100
C	-3.93502600	-3.10764200	-2.50770500
C	-2.52078900	-3.08326600	-0.60383300
C	-1.59963600	-2.16861800	-2.93147700
H	-4.26349500	-2.06617300	-2.53552600
H	-4.11548000	-3.54638400	-3.49179500
C	-4.65581400	-3.85390300	-1.36242900
C	-1.42178700	-4.27485400	-4.05516500
C	-3.74996300	-3.60698100	-0.17707600
C	-1.60403000	-2.66758600	0.34928400
C	-1.11581200	-2.79522700	-4.09039900
C	-1.31486100	-0.82520300	-2.72815800
H	-5.66881000	-3.47741900	-1.19090900
H	-4.74634800	-4.92683800	-1.57274200
H	-2.26202500	-4.51577600	-4.71833500
H	-0.57491600	-4.88555300	-4.38179000
C	-4.00015600	-3.80722700	1.17645700
C	-1.81187200	-2.87026200	1.72264300

O	-0.45197500	-1.96009500	-0.06095200
C	-0.43928500	-2.06592500	-5.06236500
C	-0.63821500	-0.05800300	-3.68710300
O	-1.67043400	-0.22214800	-1.50463200
H	-4.94656900	-4.22328900	1.50810300
C	-3.01810400	-3.47411500	2.11052900
P	-0.61781400	-0.36624300	-0.28394800
H	-0.07469200	-2.54901800	-5.96351100
C	-0.22956500	-0.70175500	-4.86644400
H	-3.18790200	-3.65398700	3.16651500
O	-1.42997300	0.24538100	0.91609200
O	0.70912600	0.25744000	-0.47547200
H	0.28386500	-0.11607500	-5.62114200
H	-2.39076800	0.02509400	0.92816700
C	-1.79080300	-4.50360700	-2.57517700
H	-0.88204100	-4.68387300	-1.99190400
H	-2.46606500	-5.34735900	-2.41599400
C	-1.60485600	0.44904700	5.70921200
C	-0.35536900	-0.09894100	5.61741800
C	-0.05970600	-1.09589100	4.63906900
C	-1.08204600	-1.49233800	3.70007600
C	-2.36482700	-0.86958300	3.81405100
C	-2.62217200	0.04464800	4.80035100
C	1.19838000	-1.69895300	4.58278200
C	-0.78240200	-2.46598200	2.71853100
C	0.48992700	-3.08274400	2.68609700
C	1.49367200	-2.69147200	3.64655600
C	2.76162700	-3.35220500	3.63669400
H	3.49791500	-3.06136600	4.38105400
C	3.03316500	-4.34492600	2.73578800
C	2.05245600	-4.72562900	1.77726500
C	0.82550900	-4.11941700	1.75721100
H	1.96031200	-1.39798300	5.29647500
H	-1.82506300	1.19079900	6.47036300
H	0.43622500	0.20606800	6.29485200
H	-3.14580200	-1.13791700	3.11402700
H	-3.61477900	0.47632200	4.88658500
H	3.99302500	-4.84878100	2.74145300
H	2.28713900	-5.50388800	1.05931300
H	0.08102200	-4.44203700	1.04008500
C	3.66202600	2.80228100	-3.53582800
C	2.62861800	3.69649600	-3.45480200
C	1.26739600	3.25389500	-3.42562900
C	0.98891600	1.83781800	-3.48574100

C	2.10345600	0.94094300	-3.52489900
C	3.39129300	1.40553100	-3.56272700
C	0.20724200	4.16116300	-3.35553700
C	-0.35244800	1.39042000	-3.49730900
C	-1.41696500	2.31893700	-3.41539300
C	-1.12384200	3.73139500	-3.34557300
C	-2.20402900	4.66508900	-3.27997200
H	-1.96689700	5.72382000	-3.22988100
C	-3.50508600	4.23960100	-3.28655600
C	-3.79916400	2.84833200	-3.36120500
C	-2.79134900	1.92315300	-3.42957300
H	0.41979900	5.22705800	-3.32793400
H	4.68911300	3.15093400	-3.56913200
H	2.82085200	4.76563200	-3.43213500
H	1.91693300	-0.12424000	-3.51771700
H	4.21566400	0.70201900	-3.59401500
H	-4.31654800	4.95839500	-3.23745300
H	-4.83127000	2.51536300	-3.36072200
H	-3.03991600	0.87261300	-3.49271800
C	2.84903900	4.51388700	1.29008300
C	2.22788300	2.89883700	-0.35897300
C	2.74767200	4.28569400	-0.19104400
H	3.44681800	3.85167000	1.90500500
H	2.18706000	5.21184600	1.78532200
H	1.15529300	2.74303700	-0.36773500
H	3.69812700	4.41519800	-0.71708000
H	2.02607700	4.96951500	-0.64046600
N	2.90008400	1.79431600	-0.39794600
H	2.29860600	0.95246400	-0.48877000
C	4.27855600	1.49409200	-0.43412900
C	5.31566500	2.39649600	-0.20635300
C	4.55628200	0.14638900	-0.75314000
C	6.64294800	1.97331800	-0.34582900
H	5.14996500	3.41621700	0.10405400
C	5.87753100	-0.25034400	-0.92482500
C	6.92234700	0.65248300	-0.72410500
H	6.07988000	-1.27400400	-1.21572800
H	7.94277100	0.31761000	-0.85746100
O	7.57881200	2.92309000	-0.09771500
C	8.95242400	2.56354500	-0.21654900
H	9.19626500	2.25353100	-1.23929300
H	9.51691800	3.46106400	0.03354000
H	9.21576600	1.76069600	0.48181400
O	3.48885700	-0.68428700	-0.92765900

C	3.31556300	-1.73436000	0.08419100
H	3.70212100	-1.36590300	1.03526600
H	2.23449000	-1.83928800	0.17159000
C	3.95733800	-3.02696200	-0.33351000
C	5.11929900	-3.50796100	0.30304200
C	3.34836300	-3.76289400	-1.37180000
C	5.65399700	-4.73344700	-0.11206100
C	3.91348200	-4.97933000	-1.76179900
C	5.05855300	-5.46588200	-1.13506900
H	6.54524200	-5.11518900	0.37724000
H	3.44809200	-5.54982000	-2.56035600
H	5.48672600	-6.41447600	-1.44380000
C	2.08668300	-3.26858100	-2.03874200
H	1.24426600	-3.26493700	-1.33881300
H	2.19446300	-2.24250600	-2.39983900
H	1.81906100	-3.90329800	-2.88660200
C	5.78772800	-2.76606300	1.43960500
H	5.10353600	-2.62323300	2.28096700
H	6.64826900	-3.33028100	1.80664700
H	6.14491600	-1.77678500	1.14045300
C	-2.73917800	2.77855300	2.94314000
C	-1.75544600	3.54043500	2.30396900
C	-1.84189400	3.77060900	0.91746900
C	-2.89120900	3.18929800	0.19413800
C	-3.84323700	2.44085700	0.86601000
C	-3.80631100	2.23159400	2.23699600
H	-2.65499100	2.60439700	4.00789100
H	-2.97306000	3.32420700	-0.87666600
H	-4.57130900	1.64238500	2.72701500
C	-0.60407600	4.06077100	3.11324500
C	-0.63104600	5.32314400	3.56627900
H	-1.48351000	5.96514200	3.37369100
H	0.18443200	5.75405300	4.13743900
C	0.52917500	3.12656900	3.34364900
C	1.49479400	3.35369500	4.34065500
C	0.66865100	1.98614400	2.53707700
C	2.57414300	2.48856700	4.49657900
H	1.39281100	4.20007500	5.01129600
C	1.74531600	1.11624600	2.69711800
H	-0.08096000	1.76369800	1.79304600
C	2.70950400	1.36771800	3.67031800
H	3.30600400	2.68127400	5.27512100
H	1.80950000	0.23418400	2.06950500
H	3.54404200	0.68651600	3.80478700

C	-0.84101800	4.63741900	0.19940500
H	-1.21528900	5.66328200	0.10346500
H	0.09585500	4.69256100	0.75317700
H	-0.65238700	4.26919900	-0.81040500
O	-4.91918500	1.90498700	0.09675600
S	-4.93951900	0.30924400	-0.21683900
O	-4.96515100	0.08687600	-1.64978600
O	-3.99962400	-0.39696800	0.66049400
C	-6.63085900	-0.08760000	0.46773600
F	-6.82819300	-1.39389900	0.30617700
F	-7.55058300	0.60320500	-0.18754200
F	-6.65565900	0.21846800	1.76187500

RR-TS1

Sum of electronic and thermal Free Energies=-4858.917167

C	2.64579700	-1.24078500	3.49112400
C	4.09843100	-0.74474500	3.76929300
C	2.82986900	-1.87358700	2.12338000
C	1.59205100	-0.18761300	3.78741600
H	4.24641100	0.21120300	3.25697400
H	4.28883500	-0.59270200	4.83432200
C	5.00137000	-1.82406000	3.12798400
C	1.70051100	-1.56605600	5.74131100
C	4.14387800	-2.34919200	1.99749600
C	1.92793000	-2.11009700	1.09710700
C	1.10664900	-0.33722200	5.09529200
C	1.10365100	0.85033200	3.00381900
H	5.94930200	-1.41352200	2.76722400
H	5.24858700	-2.61901500	3.84216600
H	2.50776200	-1.28915000	6.43129800
H	0.96810200	-2.13551000	6.32063400
C	4.50063600	-3.15631000	0.92318500
C	2.24183400	-2.94684600	0.01420700
O	0.68513100	-1.44018900	1.09791800
C	0.18813700	0.56539400	5.62036600
C	0.18242400	1.78254000	3.50194400
O	1.52828300	0.93935800	1.66343100
H	5.51247900	-3.53633800	0.82809600
C	3.53909200	-3.48068900	-0.03535700
P	0.69842500	0.09032100	0.56198200
H	-0.18531400	0.44974600	6.63304000
C	-0.25215800	1.62380700	4.82810200
H	3.79190900	-4.14032600	-0.85852000
O	1.65988200	0.16230100	-0.68431000

O	-0.67858500	0.55563800	0.29579300
H	-0.96220200	2.34213600	5.22356000
H	2.62707600	0.12821000	-0.50575300
C	2.24342900	-2.34402600	4.52817300
H	1.44238700	-2.95168000	4.09419400
H	3.07587000	-3.00914500	4.76738300
C	1.80737000	-2.40861800	-5.13143900
C	0.71494500	-3.13354100	-4.74336200
C	0.50097300	-3.46187100	-3.37058300
C	1.43984400	-2.99718700	-2.37580500
C	2.55549500	-2.22425300	-2.82553600
C	2.73919000	-1.95737200	-4.15630800
C	-0.60153400	-4.22174700	-2.97413100
C	1.23097000	-3.32031100	-1.01474200
C	0.10647500	-4.08810100	-0.62769900
C	-0.82196200	-4.54685200	-1.63418300
C	-1.93809900	-5.34877900	-1.24035700
H	-2.61077800	-5.70480800	-2.01559400
C	-2.14664400	-5.67561600	0.07100900
C	-1.23647000	-5.22509300	1.06785500
C	-0.14540300	-4.47063700	0.72916200
H	-1.29998400	-4.57506500	-3.72816700
H	1.96946100	-2.17662300	-6.17904700
H	-0.00876700	-3.48204300	-5.47404300
H	3.26421700	-1.85741200	-2.09630600
H	3.61192200	-1.40056100	-4.47838200
H	-3.00141100	-6.27581600	0.36181400
H	-1.41375800	-5.48753000	2.10519100
H	0.55225700	-4.16465800	1.49835900
C	-4.46143100	3.12390800	1.68937500
C	-3.65200200	4.19869200	1.43844100
C	-2.25602600	4.16329500	1.75273800
C	-1.70796200	2.97617200	2.36459200
C	-2.58960000	1.87415700	2.59527000
C	-3.91681500	1.94125000	2.26717300
C	-1.42116400	5.25710700	1.50810400
C	-0.34015200	2.93695900	2.71461500
C	0.49794200	4.04092900	2.44199400
C	-0.06218400	5.22630600	1.83519600
C	0.78671400	6.34900400	1.59018100
H	0.35011100	7.23647900	1.14126600
C	2.11630000	6.31124000	1.91169700
C	2.67373700	5.14201800	2.50370100
C	1.89051900	4.04845400	2.76576200

H	-1.84125700	6.15973500	1.07180000
H	-5.51801100	3.16386500	1.44630300
H	-4.05813000	5.11158500	1.01081100
H	-2.18584500	0.97228600	3.03356500
H	-4.55975800	1.08540500	2.44250800
H	2.75076300	7.17074900	1.72158600
H	3.72810400	5.12550900	2.76080300
H	2.32328800	3.17250300	3.23318900
C	-1.93825700	4.86478000	-2.13771600
C	-2.33769300	2.58688200	-1.21909900
C	-2.93171000	3.77149800	-1.89805300
H	-1.28067900	5.12869000	-1.31913000
H	-2.25387800	5.68317100	-2.77529800
H	-1.28354000	2.59258900	-0.97423400
H	-3.39805900	3.44103900	-2.83401300
H	-3.76074600	4.12801200	-1.26251000
N	-2.92888700	1.49929600	-0.85263100
H	-2.29222200	0.82627900	-0.38556400
C	-4.26316100	1.05332800	-0.92154500
C	-5.32381700	1.77132600	-1.46768000
C	-4.47599000	-0.21541000	-0.33329200
C	-6.61729400	1.23989700	-1.42595100
H	-5.19699800	2.73643300	-1.92983200
C	-5.77271400	-0.71301400	-0.26399500
C	-6.83973100	0.00132600	-0.81280000
H	-5.95439000	-1.64686000	0.24868200
H	-7.83719300	-0.41307600	-0.74401900
O	-7.57898100	2.01316100	-1.99459900
C	-8.92164500	1.53999600	-1.96798500
H	-9.28258200	1.42023400	-0.93964200
H	-9.51580700	2.30077000	-2.47316900
H	-9.01988400	0.58754200	-2.50215400
O	-3.37068200	-0.81786300	0.17250000
C	-3.20721300	-2.25820000	0.04635500
H	-3.57647900	-2.56126900	-0.93375600
H	-2.12402300	-2.38881900	0.06161100
C	-3.85385400	-3.02930600	1.16876300
C	-4.88954200	-3.95205700	0.91342100
C	-3.36724700	-2.84253100	2.48015500
C	-5.44758800	-4.65371200	1.98866600
C	-3.95393900	-3.55732200	3.52770800
C	-4.99073600	-4.45539600	3.28802700
H	-6.24559500	-5.36536100	1.79881500
H	-3.58560900	-3.41141400	4.53907600

H	-5.43726200	-5.00489200	4.11087100
C	-2.19211900	-1.93710200	2.75783900
H	-2.07462300	-1.76303000	3.83055700
H	-1.26407300	-2.38553200	2.38770300
H	-2.28712800	-0.97537500	2.25391600
C	-5.38539000	-4.25187200	-0.48451900
H	-5.76774100	-3.36725900	-1.00295900
H	-4.58607900	-4.66434500	-1.10726100
H	-6.19242900	-4.98703800	-0.45202700
C	2.27767600	1.59592200	-3.69797800
C	1.46695600	2.48600100	-2.97350300
C	2.00440300	3.11672500	-1.82685300
C	3.31429500	2.81983200	-1.43813100
C	4.07167600	1.92457300	-2.17267400
C	3.58367400	1.30880200	-3.31327400
H	1.86656200	1.10220200	-4.57130800
H	3.73152400	3.27036500	-0.54413500
H	4.20491200	0.62347100	-3.87329300
C	0.07395700	2.70664600	-3.47271000
C	-0.36398000	3.96956700	-3.76772300
H	0.31591800	4.81096300	-3.71409600
H	-1.25789200	4.12723800	-4.35440600
C	-0.81466700	1.52915100	-3.62360700
C	-2.03004200	1.59777800	-4.34050500
C	-0.52625400	0.32767000	-2.95060200
C	-2.93935200	0.54651200	-4.31827300
H	-2.26707800	2.47924700	-4.92603900
C	-1.44354900	-0.71956500	-2.91743800
H	0.40505800	0.22598200	-2.41641300
C	-2.66032500	-0.61304700	-3.58765300
H	-3.86855200	0.63006600	-4.87325800
H	-1.20296700	-1.61635200	-2.36061500
H	-3.37912200	-1.42589500	-3.55658700
C	1.22554400	4.04691100	-0.93997100
H	1.85420800	4.41055600	-0.12926100
H	0.83901200	4.91272200	-1.47679200
H	0.37772400	3.53709900	-0.47684300
O	5.39504600	1.64176900	-1.73773900
S	5.58726800	0.48801000	-0.59955200
O	6.51177000	0.96002100	0.40895800
O	4.29006200	-0.08266900	-0.23041800
C	6.46495600	-0.79413300	-1.64117400
F	6.75710600	-1.83308900	-0.86717000
F	7.56974900	-0.26726400	-2.14399300

F	5.65280400	-1.17995700	-2.62843500

RR-III

Sum of electronic and thermal Free Energies= -4858.967942

C	2.99730000	-0.70879000	3.49986300
C	4.45732500	-0.16107200	3.54309800
C	3.05901900	-1.55292700	2.23851900
C	1.95347000	0.36902900	3.73955000
H	4.53145600	0.69711300	2.86760500
H	4.74814600	0.16768300	4.54358400
C	5.31767500	-1.32562800	3.00085400
C	2.30877400	-0.68025800	5.86272000
C	4.36466400	-2.03673800	2.06558500
C	2.06828900	-1.95469700	1.35366800
C	1.61793900	0.42529900	5.10119100
C	1.35620200	1.26592400	2.86191500
H	6.21368400	-0.97362300	2.48079900
H	5.65479500	-1.98820300	3.80727200
H	3.17890300	-0.29108500	6.40662300
H	1.65760900	-1.15506400	6.60218700
C	4.63374900	-3.01156400	1.11185600
C	2.29913100	-2.94812600	0.38795100
O	0.81497200	-1.30528500	1.37760300
C	0.73851400	1.39031500	5.57876600
C	0.46896200	2.25544300	3.31042700
O	1.63466100	1.14848800	1.48513400
H	5.63810400	-3.40276200	0.98725200
C	3.59467100	-3.48504100	0.31067100
P	0.71876000	0.12575800	0.62394200
H	0.48041500	1.43194000	6.63230600
C	0.18469300	2.30399700	4.68475700
H	3.78277500	-4.26606000	-0.41805000
O	1.52455800	0.02981800	-0.72667500
O	-0.69448100	0.52332300	0.45912800
H	-0.49832500	3.06863100	5.03904600
H	2.50571000	0.00038600	-0.66065200
C	2.73579900	-1.63685800	4.73461100
H	1.90822000	-2.31149400	4.49188200
H	3.60523100	-2.24962800	4.98151900
C	1.58434800	-3.21211700	-4.74333000
C	0.52571700	-3.87717400	-4.19026300
C	0.38685300	-3.98915800	-2.77336000

C	1.36336100	-3.36335700	-1.91182600
C	2.44272700	-2.66200900	-2.53571600
C	2.55642800	-2.60719900	-3.89970800
C	-0.67272900	-4.70160000	-2.20823900
C	1.22814700	-3.47256900	-0.50700200
C	0.13548900	-4.17926200	0.05045700
C	-0.82077800	-4.81633600	-0.82482100
C	-1.89958900	-5.56244300	-0.25744700
H	-2.58645800	-6.06165000	-0.93469400
C	-2.06307400	-5.65128600	1.09713300
C	-1.13053000	-5.01722900	1.96502000
C	-0.06227300	-4.32395700	1.46095500
H	-1.39344300	-5.18672700	-2.86144800
H	1.69033200	-3.14611400	-5.82123600
H	-0.22649100	-4.34453600	-4.81873500
H	3.18390200	-2.18243400	-1.91218700
H	3.40680800	-2.10674200	-4.34975900
H	-2.89869500	-6.19921500	1.51803800
H	-1.26902200	-5.09240700	3.03825800
H	0.65295300	-3.87700600	2.13960700
C	-4.35332900	3.16597500	1.69964200
C	-3.61943000	4.23539000	1.26374500
C	-2.20457700	4.29748800	1.47377000
C	-1.55676400	3.21871700	2.18211100
C	-2.36325900	2.11889200	2.61150600
C	-3.71112300	2.08665000	2.37213900
C	-1.44250500	5.38279000	1.03159200
C	-0.16717000	3.27629300	2.42618800
C	0.59287400	4.37422700	1.96693400
C	-0.06486900	5.45038400	1.26225200
C	0.70856600	6.57110600	0.82967200
H	0.20071600	7.37688400	0.30755100
C	2.05382400	6.63522500	1.07226100
C	2.70630600	5.57430700	1.76305400
C	1.99978500	4.48350800	2.19757900
H	-1.93491300	6.20579800	0.51947700
H	-5.42439200	3.13086400	1.53145400
H	-4.10168900	5.07061800	0.76253200
H	-1.88286600	1.29621600	3.12289800
H	-4.29719000	1.23223800	2.69500700
H	2.62920500	7.49443700	0.74336600
H	3.77268900	5.63905300	1.95437800
H	2.50361600	3.68908500	2.73479400
C	-1.75354400	4.38909400	-2.75574800

C	-2.30199400	2.49430400	-1.19357600
C	-2.84807500	3.53306600	-2.10617400
H	-1.16737100	4.84015900	-1.95311300
H	-2.24025900	5.21853100	-3.27825100
H	-1.27922900	2.56952100	-0.84551500
H	-3.49636400	3.05727000	-2.84659000
H	-3.48825100	4.19262400	-1.50618600
N	-2.90414200	1.44326200	-0.74115500
H	-2.27975200	0.82550100	-0.19144600
C	-4.22428300	0.97131200	-0.88441800
C	-5.29481000	1.75573800	-1.30400300
C	-4.42181400	-0.37350600	-0.48991300
C	-6.57921000	1.20743500	-1.37067100
H	-5.18070900	2.79838500	-1.55328200
C	-5.70901300	-0.89828100	-0.53935400
C	-6.78092300	-0.12151500	-0.98560000
H	-5.89197500	-1.90368200	-0.19463500
H	-7.76905200	-0.56261100	-1.00788600
O	-7.55223100	2.05555200	-1.80064400
C	-8.88664300	1.56467900	-1.85751300
H	-9.24207100	1.25667300	-0.86688000
H	-9.49498900	2.39366500	-2.21803100
H	-8.97331500	0.72136700	-2.55314400
O	-3.30545400	-1.01572200	-0.07899900
C	-3.28383600	-2.45190200	0.08927700
H	-3.68413700	-2.90855500	-0.81770000
H	-2.21686100	-2.67102600	0.13642000
C	-3.98254000	-2.94563100	1.33333100
C	-5.00810700	-3.91018500	1.24859800
C	-3.54136000	-2.48178300	2.59128000
C	-5.61408800	-4.35948800	2.42782200
C	-4.17432700	-2.95129600	3.74535200
C	-5.21014300	-3.87831700	3.66874300
H	-6.40615100	-5.09981400	2.36617600
H	-3.83931000	-2.59166300	4.71396000
H	-5.69218200	-4.23440600	4.57384100
C	-2.36921600	-1.54116800	2.71597300
H	-1.45259800	-2.00628700	2.33913800
H	-2.50613200	-0.63003800	2.13549600
H	-2.20004300	-1.27232800	3.76207100
C	-5.43934600	-4.53008400	-0.06308400
H	-4.61429400	-5.07290400	-0.53375100
H	-6.25133200	-5.24132800	0.10191200
H	-5.79308000	-3.79836900	-0.79708600

C	1.84812400	1.08326800	-3.77833300
C	1.15177800	2.12499800	-3.12301400
C	1.87898500	2.93945100	-2.20875100
C	3.21847500	2.64682500	-1.94764100
C	3.84474500	1.59528300	-2.59439300
C	3.18677500	0.81200500	-3.52914500
H	1.31662500	0.46837400	-4.49434800
H	3.76940200	3.22707000	-1.21547700
H	3.70520500	0.00859000	-4.03333400
C	-0.27626300	2.30635700	-3.42844700
C	-0.80948100	3.68232900	-3.76866500
H	0.02973700	4.35569000	-3.95886400
H	-1.33896800	3.61096000	-4.72581900
C	-1.13664500	1.14924400	-3.57723400
C	-2.36891100	1.20756900	-4.28421700
C	-0.82934200	-0.07994300	-2.93112500
C	-3.24485200	0.13029100	-4.30561100
H	-2.63668800	2.10192100	-4.83398600
C	-1.71343600	-1.14788900	-2.95067900
H	0.08950800	-0.16959000	-2.37143600
C	-2.93142900	-1.05271300	-3.63003700
H	-4.17727600	0.21197700	-4.85542600
H	-1.45239300	-2.05800900	-2.42442800
H	-3.62576700	-1.88677400	-3.63605200
C	1.27132600	4.06501800	-1.42193400
H	1.96933300	4.42087400	-0.66608600
H	1.00902000	4.92065900	-2.04971400
H	0.36960100	3.75196100	-0.89644000
O	5.19889900	1.31560200	-2.27114700
S	5.45972100	0.32757800	-0.99445700
O	6.37891400	0.95185800	-0.06528500
O	4.19073000	-0.24071600	-0.53563000
C	6.36635300	-1.03479700	-1.89999300
F	6.72744400	-1.95626100	-1.01423600
F	7.43131600	-0.52814000	-2.49994900
F	5.54331400	-1.57164400	-2.80256700

RR-TS2

B3LYP-D3/6-31G(d,p):

Sum of electronic and thermal Free Energies=-4858.947998

SMD//M062X/6-31+G(d,p):

Sum of electronic and thermal Free Energies= -4856.912969

C	3.86073200	-0.45431500	2.89963800

C	5.25079000	0.10817800	2.47346800
C	3.56874300	-1.37844100	1.73115800
C	2.89188100	0.61526700	3.37496800
H	5.09537100	0.92333100	1.75971900
H	5.81766700	0.50102000	3.32090100
C	5.94283300	-1.08139900	1.76604600
C	3.87269600	-0.30149700	5.35803300
C	4.77560500	-1.86585400	1.20723100
C	2.37031000	-1.84675200	1.21325200
C	2.94366000	0.73406300	4.77232800
C	2.04191500	1.44910700	2.65729000
H	6.62944800	-0.75541700	0.97884300
H	6.52789600	-1.68447600	2.47091700
H	4.84302500	0.14664400	5.60759000
H	3.48298500	-0.74971200	6.27639200
C	4.77271700	-2.89821200	0.27674900
C	2.32868400	-2.91973600	0.30926700
O	1.16749400	-1.17940700	1.53221600
C	2.18242500	1.68968300	5.43495800
C	1.26568800	2.43011200	3.29454900
O	1.95685000	1.27199700	1.25833200
H	5.70354400	-3.28286600	-0.12697400
C	3.55386900	-3.44258500	-0.13285600
P	0.88326100	0.18862500	0.70388600
H	2.22040500	1.77655700	6.51627900
C	1.36143300	2.53426500	4.69127200
H	3.53478600	-4.27715900	-0.82575700
O	1.35126900	-0.04049700	-0.78350800
O	-0.53071400	0.59478700	0.82215500
H	0.76628400	3.29349300	5.18747600
H	2.32062600	-0.04630000	-0.94406500
C	4.00313500	-1.31149000	4.20442100
H	3.17094400	-2.02197200	4.24418400
H	4.93268000	-1.88428800	4.22182500
C	-0.24196000	-3.51771500	-4.16072600
C	-0.89731500	-4.30041100	-3.24943500
C	-0.48689100	-4.33813000	-1.88125800
C	0.64209400	-3.54085400	-1.46172300
C	1.30287300	-2.74813000	-2.45110200
C	0.87343700	-2.73467000	-3.75131700
C	-1.15129700	-5.13164700	-0.94353800
C	1.05454100	-3.57272200	-0.11039700
C	0.33056700	-4.33240100	0.84101700
C	-0.79338700	-5.12954600	0.40692800

C	-1.52049700	-5.89647000	1.36763400
H	-2.36781200	-6.48108100	1.02476000
C	-1.17877400	-5.87373900	2.69049200
C	-0.06244600	-5.10433300	3.12154500
C	0.67454600	-4.37228900	2.22911500
H	-1.96620600	-5.76961200	-1.27363100
H	-0.56511700	-3.49339800	-5.19632600
H	-1.74431500	-4.91098800	-3.54977600
H	2.15067400	-2.14939000	-2.15204600
H	1.39256600	-2.12753400	-4.48442700
H	-1.75482900	-6.43894900	3.41464300
H	0.21104400	-5.10559100	4.17188100
H	1.53317800	-3.81011500	2.57416600
C	-3.83196600	2.97057800	2.52112100
C	-3.26196800	4.09749900	1.99528000
C	-1.84282200	4.27383700	1.99330300
C	-1.00917100	3.24732300	2.57476500
C	-1.65042800	2.09130500	3.12027200
C	-3.01193100	1.95332000	3.08795900
C	-1.24726200	5.41267600	1.44371700
C	0.39269000	3.41149800	2.58255100
C	0.98164600	4.57054000	2.03119800
C	0.13977900	5.58746200	1.44566200
C	0.74859300	6.74294900	0.86741500
H	0.10437000	7.50184400	0.43246300
C	2.10876700	6.89445600	0.86043000
C	2.94129900	5.89692500	1.44351200
C	2.39641300	4.77667400	2.01372800
H	-1.87810100	6.18690200	1.01363800
H	-4.90983200	2.84605300	2.50960200
H	-3.87972000	4.88890800	1.57960500
H	-1.03414500	1.31007400	3.54586500
H	-3.47398400	1.06041700	3.49618000
H	2.55918800	7.77712700	0.41817300
H	4.01806500	6.03334800	1.44065300
H	3.03803400	4.02674400	2.46081800
C	-2.27647800	4.74665300	-2.54837400
C	-2.19685000	2.78539800	-1.08456300
C	-3.10186300	3.86055900	-1.62670400
H	-1.59004700	5.36097600	-1.95774200
H	-2.91404200	5.43687300	-3.10659100
H	-1.31614800	3.13499200	-0.56541300
H	-3.90806700	3.40766600	-2.20492700
H	-3.55118200	4.42539000	-0.80310600

N	-2.68389600	1.66142200	-0.46810200
H	-1.95135300	1.09500400	-0.00768500
C	-3.94141500	1.10794200	-0.44513000
C	-5.13706500	1.81533700	-0.65983100
C	-4.01974300	-0.28705100	-0.09712800
C	-6.36722800	1.17498400	-0.60725900
H	-5.14436500	2.87822300	-0.82345000
C	-5.26646500	-0.90746400	-0.03198200
C	-6.43186700	-0.19844800	-0.29718600
H	-5.33068700	-1.94088500	0.26687800
H	-7.38339800	-0.71005800	-0.23249000
O	-7.44941000	1.95960300	-0.83944200
C	-8.74577700	1.37696600	-0.74192300
H	-8.88141200	0.57749700	-1.47956000
H	-8.93279300	0.98322900	0.26382200
H	-9.44904600	2.18239100	-0.95080100
O	-2.84315200	-0.87696400	0.13941100
C	-2.69057300	-2.32008700	0.17546200
H	-3.05258500	-2.71026400	-0.77501000
H	-1.60667500	-2.43373700	0.20276800
C	-3.33385500	-3.01824800	1.34899500
C	-4.28048400	-4.04278300	1.13179000
C	-2.92715800	-2.68898400	2.65806200
C	-4.83435200	-4.69900000	2.23631600
C	-3.50709700	-3.36492500	3.73548000
C	-4.45784000	-4.36014400	3.53170700
H	-5.56496900	-5.48556700	2.07260500
H	-3.19670000	-3.11010300	4.74447300
H	-4.89831600	-4.87617300	4.37919900
C	-1.84955500	-1.66786600	2.91480500
H	-2.02983800	-0.72585700	2.39762800
H	-1.75562300	-1.47081900	3.98598100
H	-0.88074600	-2.03336000	2.56134000
C	-4.70234100	-4.48333100	-0.25356600
H	-5.43236900	-5.29282600	-0.18838500
H	-5.15972800	-3.67825200	-0.83941000
H	-3.85299900	-4.84942600	-0.83638900
C	0.62953200	1.07487100	-3.81274500
C	0.18557700	2.03804900	-2.88352600
C	1.17245000	2.74430100	-2.14498600
C	2.52125400	2.42966500	-2.32753900
C	2.90169300	1.44983100	-3.22748000
C	1.97369900	0.76590900	-3.99251900
H	-0.09608800	0.55831700	-4.42565100

H	3.27868500	2.94964600	-1.75171800
H	2.29243300	0.02786000	-4.71728000
C	-1.28549100	2.39755300	-2.90190500
C	-1.49041100	3.81122800	-3.49362900
H	-0.51718400	4.24326600	-3.72946600
H	-2.01114800	3.71611400	-4.44981200
C	-2.20547600	1.30763300	-3.33082000
C	-3.37271000	1.54610700	-4.07761400
C	-1.97362500	-0.01362300	-2.88686800
C	-4.25390100	0.51150400	-4.39099100
H	-3.60146400	2.54332800	-4.43418200
C	-2.83833200	-1.04758300	-3.22301200
H	-1.10745100	-0.22795400	-2.27096600
C	-3.98771600	-0.79094400	-3.97430600
H	-5.14387700	0.72770200	-4.97319500
H	-2.60460400	-2.05693300	-2.90381300
H	-4.66646400	-1.59779000	-4.23133500
C	0.87739900	3.78919900	-1.10886500
H	0.11117400	4.49752900	-1.41447200
H	0.55625900	3.32046300	-0.17641200
H	1.77376000	4.36168800	-0.86884300
O	4.28260000	1.17043900	-3.38765300
S	4.98366100	0.19025400	-2.28296200
O	6.28682500	0.71777800	-1.94270200
O	4.01248500	-0.16356000	-1.24650300
C	5.22958200	-1.32240600	-3.35772300
F	5.98610800	-0.99965700	-4.39443400
F	4.03727900	-1.75076700	-3.77846300
F	5.81612000	-2.26508900	-2.63031700

RR-IV

Sum of electronic and thermal Free Energies=-4858.960020

C	4.13638800	-2.24864900	-1.75135900
C	5.15133700	-2.73963400	-0.67281800
C	4.03512800	-0.77611800	-1.38179000
C	2.93941400	-3.17458500	-1.88950300
H	4.61422600	-2.90939300	0.26582800
H	5.64087000	-3.67387600	-0.95745300
C	6.12705400	-1.55525600	-0.50438100
C	4.50082800	-3.85964200	-3.57154800
C	5.25049200	-0.36237800	-0.81254100
C	3.03328300	0.16432000	-1.59028800
C	3.18989100	-4.14120600	-2.87697900
C	1.72874500	-3.20611200	-1.20936300

H	6.54806400	-1.50121100	0.50284700
H	6.96784100	-1.62311500	-1.20555900
H	5.29108100	-4.52430500	-3.20005400
H	4.44342500	-4.00706600	-4.65376300
C	5.50045300	0.98408500	-0.57867000
C	3.25684800	1.53558100	-1.37156800
O	1.75686500	-0.25910000	-2.00544100
C	2.27170500	-5.15241000	-3.13358800
C	0.76835200	-4.19945700	-1.46215400
O	1.44630400	-2.17434500	-0.29011800
H	6.44754600	1.30474300	-0.15741800
C	4.51472800	1.92196500	-0.88465100
P	0.71767300	-0.85620600	-0.90809700
H	2.47000300	-5.90071300	-3.89455700
C	1.07654100	-5.18044200	-2.41727400
H	4.69983400	2.97899400	-0.72568600
O	0.71722400	0.11656200	0.34972300
O	-0.60613200	-1.07040200	-1.51845200
H	0.34147100	-5.95398200	-2.61245500
H	1.54756800	0.13456100	0.86717100
C	4.76937300	-2.39561800	-3.17626400
H	4.24007800	-1.72569400	-3.86172700
H	5.82651400	-2.12273400	-3.18957700
C	0.53149000	4.98015600	1.38693800
C	0.26062300	5.26416800	0.07600300
C	0.80957100	4.47194600	-0.98049400
C	1.68948000	3.37346300	-0.65141900
C	1.93812200	3.11289600	0.73316000
C	1.37203000	3.87914700	1.71647300
C	0.50633100	4.73328600	-2.31802900
C	2.24620000	2.58795800	-1.68679300
C	1.93354700	2.86912200	-3.03790200
C	1.04370000	3.96219800	-3.35165200
C	0.74006200	4.24663800	-4.71806100
H	0.05350900	5.05956800	-4.93137400
C	1.29530900	3.51269100	-5.72979500
C	2.18593200	2.44415000	-5.42725000
C	2.49229500	2.13138100	-4.12908000
H	-0.16889100	5.54813800	-2.56212400
H	0.11346600	5.59094200	2.18013100
H	-0.37946600	6.09994800	-0.19102700
H	2.58942100	2.29277700	0.99428300
H	1.56830600	3.64984200	2.75791800
H	1.06255500	3.74090900	-6.76483200

H	2.62835400	1.87350500	-6.23764500
H	3.18032500	1.32262600	-3.91634400
C	-4.09789800	-3.48943400	-3.00907500
C	-4.19416000	-3.85496200	-1.69042500
C	-3.02202900	-4.09251700	-0.91124400
C	-1.72634200	-3.94835500	-1.52962500
C	-1.67788400	-3.50554000	-2.88860600
C	-2.82389800	-3.29238000	-3.60716400
C	-3.10614000	-4.50015100	0.42327500
C	-0.56284400	-4.25000400	-0.79025500
C	-0.66273900	-4.69803700	0.54572100
C	-1.96223300	-4.81427100	1.16355900
C	-2.04881700	-5.26539200	2.51566100
H	-3.03312300	-5.35782900	2.96622200
C	-0.92187900	-5.58486700	3.22687700
C	0.35944400	-5.47783100	2.61815000
C	0.48382300	-5.05512200	1.31957900
H	-4.08411100	-4.60712400	0.88559500
H	-4.99331800	-3.35919200	-3.60879800
H	-5.16296200	-3.98946100	-1.21902100
H	-0.71167400	-3.33969500	-3.34593500
H	-2.76182200	-2.97331100	-4.64260200
H	-1.00118300	-5.93124600	4.25226300
H	1.24401800	-5.74612700	3.18678900
H	1.46274700	-4.99102600	0.85917600
C	-4.14279700	-1.07262000	4.87690000
C	-3.49138300	-0.06885700	2.83731900
C	-3.99527200	-1.41904300	3.38883700
H	-5.03270100	-0.44974300	5.02375400
H	-4.25540100	-1.95789700	5.50712800
H	-4.36359200	0.59373600	2.90631300
H	-4.94363700	-1.72973200	2.94862800
H	-3.28014700	-2.22603400	3.23973600
N	-3.01011600	0.06012600	1.47011300
H	-2.12296500	0.53269500	1.33389100
C	-3.72653400	-0.12737900	0.34459300
C	-4.95621600	-0.79522100	0.28693100
C	-3.16883800	0.42340400	-0.88089200
C	-5.57624100	-1.00681400	-0.93611100
H	-5.42940300	-1.18346500	1.17434900
C	-3.78833800	0.15542200	-2.10781900
C	-4.97516600	-0.54758300	-2.14414900
H	-3.35946500	0.53441700	-3.02097000
H	-5.45073600	-0.73182700	-3.09707800

O	-6.74502200	-1.67348000	-0.89515600
C	-7.50381100	-1.84322800	-2.09517200
H	-6.96567900	-2.46247500	-2.81897900
H	-8.41787800	-2.35166700	-1.79233000
H	-7.75309600	-0.87640500	-2.54362900
O	-2.12454000	1.21137800	-0.68470900
C	-1.37646400	1.78257900	-1.80368600
H	-0.56264900	2.29430200	-1.29331700
H	-0.96818600	0.94310400	-2.35607500
C	-2.16052800	2.72071200	-2.67853700
C	-2.87444000	3.79736600	-2.11741000
C	-2.09581500	2.54643500	-4.07676500
C	-3.52006100	4.69554100	-2.97226200
C	-2.75295600	3.46741600	-4.89877900
C	-3.45973900	4.53585700	-4.35435500
H	-4.07005200	5.52940300	-2.54582100
H	-2.70058200	3.34468200	-5.97631900
H	-3.96174100	5.24426000	-5.00596500
C	-1.32634500	1.40651800	-4.70637600
H	-1.40730100	1.45068200	-5.79415700
H	-0.26296800	1.44155900	-4.45704000
H	-1.69373000	0.42396700	-4.38538300
C	-2.95925600	3.99744400	-0.62469500
H	-1.99589100	3.83200200	-0.13775300
H	-3.28484300	5.01421300	-0.39160900
H	-3.67292400	3.31018600	-0.15864400
C	-0.13916500	1.21830600	4.42897700
C	-0.94345000	0.29363500	3.72949000
C	-0.30270700	-0.83102200	3.15490900
C	1.08620500	-0.96288000	3.29970400
C	1.83035600	-0.02148200	3.97977300
C	1.23884400	1.09047300	4.55620100
H	-0.61298500	2.07089000	4.89752300
H	1.58326900	-1.82026400	2.85996800
H	1.83026700	1.82176600	5.09324900
C	-2.46949700	0.51327300	3.89791400
C	-2.86357600	-0.28666700	5.19474200
H	-2.06050700	-0.99476000	5.41087200
H	-2.94466900	0.36292400	6.06761000
C	-2.79982600	2.01569600	3.93260800
C	-3.74732700	2.54002200	4.81824100
C	-2.25706100	2.87260000	2.96126000
C	-4.13542700	3.87936800	4.74775300
H	-4.20264000	1.90542900	5.57026700

C	-2.64749300	4.20798200	2.88593800
H	-1.49625900	2.51191800	2.27574800
C	-3.58909000	4.71895500	3.77966700
H	-4.86735500	4.26170200	5.45218800
H	-2.20626300	4.84607700	2.12905600
H	-3.89035700	5.75995300	3.72354800
C	-0.94201900	-1.97849800	2.41996800
H	-1.31666900	-2.72918600	3.12117600
H	-1.75213000	-1.69551200	1.75730200
H	-0.19235400	-2.47271200	1.80667600
O	3.22889200	-0.21429900	4.10132200
S	4.13088900	0.03028600	2.75998800
O	5.21478400	-0.92959600	2.75807300
O	3.25349700	0.21001200	1.60723300
C	4.83406800	1.70807800	3.21058400
F	5.62320100	2.13224800	2.23204600
F	5.51869000	1.59543100	4.33845700
F	3.82147500	2.56262600	3.37531300

RS-TS1

Sum of electronic and thermal Free Energies = -4858.915567

C	2.61965700	-0.68439600	3.63770800
C	4.11123200	-0.25339200	3.78059100
C	2.69787800	-1.53353900	2.38224500
C	1.63863500	0.46136500	3.80935800
H	4.29610200	0.59725800	3.11680600
H	4.36004700	0.05138200	4.79983600
C	4.91185700	-1.48393700	3.29133800
C	1.76265200	-0.61366100	5.94502200
C	3.96502500	-2.13175300	2.30433000
C	1.73471300	-1.87342600	1.44408400
C	1.20202600	0.53447500	5.14118700
C	1.17003300	1.39590900	2.89471600
H	5.85933600	-1.20417700	2.82095100
H	5.15285400	-2.16294200	4.11813300
H	2.61529700	-0.28222000	6.55134200
H	1.03077500	-1.04542800	6.63358700
C	4.20806700	-3.14975000	1.39020100
C	1.92764000	-2.93068400	0.54203900
O	0.55031300	-1.10603400	1.34849500
C	0.34775100	1.54974700	5.55548100
C	0.31673700	2.44034200	3.28025200
O	1.55832600	1.26888100	1.54467600
H	5.18283800	-3.62260300	1.33565900

C	3.17721800	-3.56776300	0.54589800
P	0.67308800	0.31786400	0.57889700
H	0.01016300	1.60432400	6.58565300
C	-0.07494500	2.49887600	4.62805000
H	3.33343300	-4.39370500	-0.13999200
O	1.62306900	0.14511300	-0.66752500
O	-0.66552800	0.83487500	0.22654100
H	-0.73183600	3.30580100	4.93433000
H	2.57991800	-0.00454300	-0.50011500
C	2.20597100	-1.59485200	4.84512200
H	1.35379800	-2.21024600	4.53783200
H	3.01109300	-2.26636000	5.15034800
C	0.83495800	-3.11936300	-4.53512300
C	-0.15893400	-3.82682300	-3.91752700
C	-0.18944700	-3.96409400	-2.49577300
C	0.83899500	-3.32794800	-1.70417900
C	1.85621200	-2.59606000	-2.39275600
C	1.85658400	-2.50419400	-3.75896000
C	-1.18013300	-4.71480700	-1.85790700
C	0.81815600	-3.46414600	-0.30039600
C	-0.19388500	-4.22335800	0.33378700
C	-1.20385200	-4.87092500	-0.46903900
C	-2.18610700	-5.68768900	0.16928500
H	-2.93871300	-6.16525200	-0.44960100
C	-2.18216300	-5.86636700	1.52424500
C	-1.19503100	-5.22400900	2.32080000
C	-0.23541400	-4.43122100	1.74906800
H	-1.93457600	-5.21537300	-2.45914400
H	0.85136600	-3.02587900	-5.61604000
H	-0.94332100	-4.30418600	-4.49748900
H	2.63441800	-2.11874300	-1.81390700
H	2.64554900	-1.95649000	-4.26036800
H	-2.93665000	-6.48358800	1.99761000
H	-1.20145300	-5.37042600	3.39627900
H	0.52324700	-3.97122900	2.37029200
C	-4.28614700	3.90106000	1.43164600
C	-3.41215300	4.88727300	1.05482100
C	-2.01969700	4.79317600	1.36372400
C	-1.53454500	3.63179500	2.07334400
C	-2.48163400	2.61982300	2.42929500
C	-3.81119000	2.75486600	2.12816700
C	-1.11159600	5.76882800	0.94186700
C	-0.15661800	3.51943000	2.36732500
C	0.75328200	4.50362800	1.91907100

C	0.26070100	5.64325400	1.18035800
C	1.18774600	6.63139500	0.72704700
H	0.80325400	7.48244000	0.17231200
C	2.52474200	6.51946600	0.99618300
C	3.01125800	5.40894700	1.74310900
C	2.15588200	4.43789900	2.19135300
H	-1.47909100	6.63947100	0.40517300
H	-5.34206500	3.98446800	1.19476700
H	-3.76227100	5.76545800	0.51964100
H	-2.12847700	1.72456300	2.92398100
H	-4.50640500	1.97005300	2.40242500
H	3.21812700	7.28014900	0.65243300
H	4.07133700	5.33982700	1.96565700
H	2.53821400	3.60587100	2.76939700
C	-1.29111400	4.53973400	-2.48048100
C	-2.73704900	2.77332700	-1.48276100
C	-1.39238200	3.39388800	-1.52714700
H	-2.11784300	4.74006400	-3.15376300
H	-0.70464500	5.40012600	-2.18518600
H	-3.57675800	3.29438900	-1.93125000
H	-1.15746000	3.72523000	-0.51021600
H	-0.65722700	2.60536700	-1.72319600
N	-2.98712800	1.65451800	-0.88804900
H	-2.20268400	1.18510200	-0.38797800
C	-4.26893700	1.06485800	-0.75072800
C	-5.34655900	1.44243900	-1.54707400
C	-4.41932800	0.10996000	0.27024300
C	-6.60911300	0.89138000	-1.30917800
H	-5.24024800	2.13362200	-2.37361700
C	-5.69315200	-0.39686800	0.52642000
C	-6.78338600	-0.01736800	-0.25406200
H	-5.82056400	-1.10239700	1.33685300
H	-7.75696400	-0.43838700	-0.03970700
O	-7.59236100	1.30440400	-2.14745500
C	-8.90214300	0.77572300	-1.96106500
H	-9.30390500	1.04290700	-0.97685300
H	-9.51820800	1.22743600	-2.73781400
H	-8.91173500	-0.31440600	-2.07580600
O	-3.31852600	-0.24365100	0.99916900
C	-2.74738800	-1.54993300	0.63946700
H	-2.52622800	-1.54165700	-0.42846600
H	-1.80344500	-1.57684800	1.17691700
C	-3.66204400	-2.67384100	1.03177900
C	-4.44213100	-3.34614300	0.07192700

C	-3.78503300	-2.98568900	2.40056600
C	-5.34769600	-4.32360100	0.50052100
C	-4.69879500	-3.96646000	2.79404200
C	-5.48132200	-4.63016200	1.85071100
H	-5.95509600	-4.84253900	-0.23531500
H	-4.79805300	-4.20836000	3.84821400
H	-6.19182000	-5.38684700	2.16919000
C	-2.95152800	-2.26819400	3.43424300
H	-3.21267400	-2.59804000	4.44231500
H	-1.88439400	-2.46487200	3.28127900
H	-3.09314900	-1.18507100	3.37654700
C	-4.34186700	-3.03167300	-1.40211000
H	-3.32376000	-3.17466300	-1.77651500
H	-5.00113600	-3.68528700	-1.97831500
H	-4.63217000	-1.99787900	-1.61468000
C	2.46975800	1.04910500	-4.04918200
C	1.81802400	2.09732200	-3.37542500
C	2.47561700	2.72500800	-2.29085800
C	3.73853800	2.25884100	-1.90928100
C	4.32687000	1.20086700	-2.57752400
C	3.72323300	0.58670800	-3.66403900
H	1.96719900	0.57737600	-4.88619200
H	4.24953700	2.70779000	-1.06425800
H	4.21893200	-0.22374900	-4.18308700
C	0.45896100	2.47617400	-3.87052500
C	0.19980400	3.76461100	-4.24692300
H	0.99207800	4.50392600	-4.23723800
H	-0.67331900	4.02712700	-4.82599900
C	-0.60753200	1.44975900	-3.87610900
C	-1.86094900	1.69267000	-4.48163100
C	-0.45860100	0.25026800	-3.15409800
C	-2.90981200	0.78637300	-4.35981200
H	-2.01602200	2.58830500	-5.07187300
C	-1.51281300	-0.65353700	-3.03025300
H	0.47646000	0.03082500	-2.66045000
C	-2.74467500	-0.39281100	-3.62783400
H	-3.85737100	0.99532000	-4.84736100
H	-1.35657100	-1.56701800	-2.46892900
H	-3.56291800	-1.09801500	-3.53429400
C	1.87403500	3.80871700	-1.43608300
H	1.30143300	3.36790400	-0.61473600
H	2.65425300	4.41698000	-0.97739400
H	1.21438000	4.46784300	-1.98998300
O	5.59262300	0.73897400	-2.12903600

S	5.59886500	-0.30898200	-0.87589900
O	6.70549800	0.01362800	-0.00079800
O	4.24152900	-0.47593100	-0.35536500
C	6.03809400	-1.87005100	-1.81362900
F	6.20638100	-2.85896800	-0.94398800
F	7.15454000	-1.66155200	-2.49405800
F	5.04238900	-2.16792600	-2.65044000

RS-III

Sum of electronic and thermal Free Energies = -4858.964177

C	2.71881300	-0.57905600	3.57988100
C	4.20778200	-0.12840200	3.67647900
C	2.77491500	-1.45090600	2.33862400
C	1.72554400	0.55510800	3.75389700
H	4.36607200	0.70992100	2.99061000
H	4.47851800	0.19982900	4.68279100
C	5.00955000	-1.36026000	3.19236000
C	1.91113700	-0.47951400	5.90451200
C	4.04596900	-2.03814100	2.24231600
C	1.79260700	-1.81821300	1.43071600
C	1.31238100	0.64277200	5.09197900
C	1.22336000	1.46459000	2.83177100
H	5.94259700	-1.08003100	2.69405000
H	5.27833900	-2.01976100	4.02633300
H	2.77136100	-0.12158600	6.48456100
H	1.20297200	-0.91081300	6.61771200
C	4.27792800	-3.07170200	1.34305000
C	1.97394000	-2.89505900	0.54921800
O	0.60174300	-1.06037500	1.34210600
C	0.44350900	1.64662300	5.50394600
C	0.35075400	2.49369900	3.21333200
O	1.59554200	1.32384500	1.47941700
H	5.25532900	-3.53677700	1.27495100
C	3.23067600	-3.51824200	0.53446800
P	0.70761300	0.34942400	0.53987500
H	0.12325400	1.71265500	6.53896600
C	-0.01882800	2.56713800	4.56705900
H	3.37742500	-4.35931900	-0.13492000
O	1.65440100	0.14809300	-0.70638000
O	-0.63661000	0.84937000	0.18449400
H	-0.69120400	3.36238400	4.87007100
H	2.61509300	0.03188100	-0.53604300
C	2.34688800	-1.47339900	4.81317800
H	1.49843900	-2.10813900	4.53649900

H	3.16937400	-2.12614100	5.11271900
C	0.79128500	-3.37932700	-4.48875300
C	-0.17987700	-4.06537600	-3.81362400
C	-0.18788100	-4.11424400	-2.38584200
C	0.84031500	-3.41359000	-1.65020100
C	1.82968700	-2.70109600	-2.39759600
C	1.80879200	-2.69497900	-3.76709600
C	-1.15668700	-4.84042400	-1.68883100
C	0.84848500	-3.47316600	-0.24070900
C	-0.14033100	-4.21133700	0.45342300
C	-1.15588800	-4.91508100	-0.29320100
C	-2.11570400	-5.70756000	0.40689800
H	-2.87326200	-6.22690000	-0.17092600
C	-2.08406000	-5.81298700	1.76900500
C	-1.08948300	-5.11820700	2.51007200
C	-0.15118200	-4.34607400	1.87812100
H	-1.91137400	-5.38806600	-2.24718500
H	0.79288500	-3.35483400	-5.57353000
H	-0.96231000	-4.59321800	-4.35096700
H	2.60182800	-2.16838200	-1.85988800
H	2.57883300	-2.16661500	-4.31726800
H	-2.82162600	-6.41240100	2.28950900
H	-1.07292700	-5.20716800	3.59169400
H	0.61345400	-3.84616400	2.45956200
C	-4.34415500	3.81163700	1.51619200
C	-3.51604000	4.82951600	1.12364400
C	-2.10837500	4.76625000	1.36537400
C	-1.56358300	3.61346400	2.04382800
C	-2.46543200	2.56966000	2.42214700
C	-3.80821300	2.66690200	2.16991600
C	-1.24192900	5.76712500	0.91114300
C	-0.17439600	3.54322800	2.29402500
C	0.69197000	4.55012300	1.81223100
C	0.14208600	5.67512000	1.09192000
C	1.02484900	6.68881100	0.60726400
H	0.59791800	7.52754300	0.06491200
C	2.37212700	6.61797600	0.83577800
C	2.91461500	5.52492400	1.56993500
C	2.10333000	4.52744400	2.04217600
H	-1.65331700	6.63471500	0.40133600
H	-5.41082200	3.86804600	1.32393100
H	-3.91307900	5.70559700	0.61843300
H	-2.06645100	1.67930500	2.89016600
H	-4.46804900	1.85497800	2.45261900

H	3.03139300	7.39849600	0.47008400
H	3.98234700	5.48945000	1.76188100
H	2.52804200	3.70764100	2.60795700
C	-1.13301100	4.33546300	-2.80535800
C	-2.73886700	2.69430500	-1.68209900
C	-1.45293600	3.41601500	-1.61143000
H	-2.06888400	4.65872700	-3.27367800
H	-0.67648400	5.24195600	-2.40191100
H	-3.55444000	3.13350900	-2.24716000
H	-1.55210500	4.04719700	-0.71979400
H	-0.65435400	2.71205900	-1.37716800
N	-3.00617000	1.64336000	-0.97427500
H	-2.24248800	1.22982900	-0.40459600
C	-4.28074800	1.04196000	-0.82402600
C	-5.37882000	1.40814000	-1.59728300
C	-4.40620500	0.09270000	0.20630600
C	-6.62922400	0.83924000	-1.33930900
H	-5.30613700	2.11376600	-2.41485500
C	-5.66925000	-0.42739500	0.48694300
C	-6.77615500	-0.06865300	-0.27979800
H	-5.77589100	-1.12587100	1.30626800
H	-7.74151300	-0.49817600	-0.04611300
O	-7.63183600	1.24207500	-2.16039600
C	-8.93379700	0.70570500	-1.94548000
H	-9.31691000	0.97299600	-0.95384000
H	-9.56890300	1.15106800	-2.71053300
H	-8.93904400	-0.38484000	-2.05688900
O	-3.28936400	-0.23305100	0.92431100
C	-2.71870500	-1.54978500	0.61090500
H	-2.54543600	-1.59873700	-0.46416800
H	-1.75167600	-1.53723100	1.10658700
C	-3.60084500	-2.65856000	1.10833000
C	-4.40197300	-3.40432400	0.22308900
C	-3.67260700	-2.87930100	2.49832800
C	-5.27898700	-4.36116000	0.74698700
C	-4.55835700	-3.84259100	2.98771400
C	-5.36311000	-4.57718800	2.11859900
H	-5.90322600	-4.93581500	0.06889500
H	-4.61849800	-4.01428700	4.05850100
H	-6.05206900	-5.31872300	2.51139800
C	-2.81634500	-2.08248100	3.45236200
H	-3.02505800	-2.36050500	4.48791700
H	-1.75122600	-2.25751400	3.26400000
H	-2.99191500	-1.00861300	3.33964500

C	-4.35317700	-3.18979300	-1.27128500
H	-3.34925600	-3.36517400	-1.66981800
H	-5.03570400	-3.87568900	-1.77883000
H	-4.64427600	-2.17034400	-1.54273400
C	2.22622500	0.86282500	-3.90765200
C	1.64422700	2.00317400	-3.30746700
C	2.44166000	2.77040600	-2.40354100
C	3.73139000	2.32761300	-2.10631200
C	4.23844900	1.17713800	-2.68790700
C	3.51355700	0.43906500	-3.61042800
H	1.63895100	0.29692600	-4.62044800
H	4.33792200	2.86572400	-1.38617200
H	3.94671100	-0.44031400	-4.06934200
C	0.25406900	2.31834300	-3.63590000
C	-0.16965900	3.74091100	-3.88504500
H	0.71294500	4.37271000	-3.97480200
H	-0.65353300	3.79451700	-4.86687500
C	-0.76387000	1.29559500	-3.70008700
C	-2.03605900	1.56684100	-4.28025500
C	-0.60401400	0.03279600	-3.07216600
C	-3.06414000	0.62984500	-4.25063800
H	-2.20523100	2.50505900	-4.79508300
C	-1.64219800	-0.88740200	-3.03219400
H	0.32715900	-0.20435800	-2.57952800
C	-2.87943400	-0.60133300	-3.61907800
H	-4.01343800	0.86121300	-4.72411700
H	-1.47985100	-1.83828200	-2.53935000
H	-3.68382300	-1.32757900	-3.58728500
C	1.96136800	3.98369800	-1.64691900
H	1.05428000	3.77846000	-1.07866300
H	2.71321500	4.29847600	-0.92416100
H	1.76044200	4.83971700	-2.29515400
O	5.53459400	0.74689000	-2.30850700
S	5.63646100	-0.19738000	-0.97521000
O	6.77726300	0.21890100	-0.18772400
O	4.31237200	-0.35416500	-0.37141200
C	6.07644500	-1.81375900	-1.81445500
F	6.38438300	-2.70787700	-0.88255700
F	7.11053800	-1.61354100	-2.61671500
F	5.02647800	-2.23751300	-2.52047600

RS-TS2

B3LYP-D3/6-31G(d,p):

Sum of electronic and thermal Free Energies = -4858.940436

SMD//M062X/6-31+G(d,p):

Sum of electronic and thermal Free Energies = -4856.909512

C	4.43492600	-2.18645100	-1.56383100
C	5.04458900	-3.09514800	-0.45353900
C	4.32937700	-0.87003700	-0.81219100
C	3.27196700	-2.81992000	-2.31273600
H	4.23827400	-3.44039300	0.20148400
H	5.54655800	-3.97392400	-0.86524300
C	5.99401300	-2.15409800	0.32786000
C	5.21021300	-3.21191200	-3.66469700
C	5.34979700	-0.79636600	0.14796700
C	3.50493700	0.22979400	-1.00247300
C	3.73143400	-3.45060200	-3.47965300
C	1.91595000	-2.87374500	-2.00807800
H	6.08195200	-2.42816300	1.38348700
H	7.00806200	-2.17044400	-0.08948300
H	5.78749400	-4.09059400	-3.34948200
H	5.47958300	-3.00993800	-4.70524600
C	5.62417200	0.40668800	0.79021300
C	3.78474300	1.47546600	-0.41766100
O	2.29073500	0.07177400	-1.70527900
C	2.86017900	-4.15531200	-4.30187500
C	1.00852500	-3.57394100	-2.82008200
O	1.46430500	-2.22081600	-0.84518800
H	6.41240600	0.47136300	1.53336300
C	4.87805000	1.54029700	0.46256200
P	1.10428400	-0.63907700	-0.86029500
H	3.21992500	-4.64034800	-5.20382600
C	1.51100700	-4.21871000	-3.96047900
H	5.11290500	2.49503300	0.92029700
O	1.25481100	-0.25140200	0.66591300
O	-0.23212800	-0.27408300	-1.37386900
H	0.81302100	-4.76228200	-4.58842400
H	2.02381400	-0.56726600	1.18321800
C	5.46378900	-2.01301000	-2.73497100
H	5.23189200	-1.08335000	-3.26467900
H	6.49191500	-1.94368300	-2.37379900
C	1.06381200	4.92218800	2.33962300
C	1.05411700	5.38935800	1.05293400
C	1.67464100	4.65539100	-0.00510600
C	2.34094300	3.41003200	0.29535100
C	2.29783600	2.94675100	1.64781500
C	1.68906900	3.67728000	2.63556200

C	1.62727700	5.10950900	-1.32313500
C	2.98796900	2.69253000	-0.73721700
C	2.93731000	3.17037900	-2.07022400
C	2.22674300	4.39326400	-2.36044900
C	2.13997200	4.85178500	-3.71013900
H	1.58729100	5.76546500	-3.90393600
C	2.73034900	4.15638100	-4.72913700
C	3.46396700	2.97047300	-4.44475600
C	3.57058400	2.50044400	-3.16304600
H	1.09215000	6.02658600	-1.55061300
H	0.59801400	5.49346500	3.13631400
H	0.57291500	6.33047100	0.80521400
H	2.75469900	1.99590300	1.88117900
H	1.68468300	3.30092200	3.65364100
H	2.65725500	4.51108600	-5.75215600
H	3.95118400	2.43812400	-5.25553000
H	4.14909000	1.60633400	-2.96868400
C	-3.20815900	-0.81075100	-3.99316200
C	-3.66662100	-1.87366900	-3.26331400
C	-2.76826600	-2.87318100	-2.77937700
C	-1.35571500	-2.73352500	-3.04786600
C	-0.92772300	-1.62240700	-3.83737100
C	-1.82227100	-0.69390500	-4.29542400
C	-3.22420000	-3.98210000	-2.05987600
C	-0.45335800	-3.68720300	-2.53055000
C	-0.92614200	-4.80485000	-1.80653600
C	-2.34528700	-4.95765300	-1.57976900
C	-2.81068600	-6.08942700	-0.84250100
H	-3.88076800	-6.20096400	-0.69090900
C	-1.93380400	-7.01447800	-0.34205200
C	-0.53393500	-6.85726000	-0.54992300
C	-0.04744100	-5.79064200	-1.25863200
H	-4.29108500	-4.09640000	-1.88198900
H	-3.90121900	-0.05774700	-4.35470400
H	-4.72561700	-1.98129000	-3.04760700
H	0.12750500	-1.51656200	-4.05785400
H	-1.47329100	0.14679400	-4.88565000
H	-2.30026700	-7.87191200	0.21322300
H	0.15092400	-7.59514200	-0.14461300
H	1.01910400	-5.68055100	-1.41585600
C	-1.82808800	-3.67310900	1.85835800
C	-2.73394100	-1.77144100	0.59675700
C	-1.51703500	-2.63856400	0.75166700
H	-1.98036500	-4.66914300	1.43852900

H	-0.98526900	-3.73803400	2.55071100
H	-3.67058500	-2.27998800	0.39238300
H	-1.25525500	-3.10659800	-0.19350600
H	-0.67215100	-2.01058500	1.03823000
N	-2.61037500	-0.57652200	-0.03870300
H	-1.66083700	-0.25674200	-0.28762100
C	-3.63984600	0.29597900	-0.32920500
C	-4.96232000	-0.11287500	-0.50782200
C	-3.30287000	1.68442900	-0.36615400
C	-5.96662900	0.83116500	-0.68431700
H	-5.22944000	-1.15980200	-0.50481700
C	-4.31823800	2.62049700	-0.54513000
C	-5.64041700	2.20116800	-0.69549900
H	-4.08210900	3.67417200	-0.58707000
H	-6.40922100	2.94942300	-0.83813100
O	-7.22025400	0.33558900	-0.85026400
C	-8.29906200	1.25625400	-0.96136600
H	-8.19703400	1.89159200	-1.84872100
H	-9.19986200	0.65045900	-1.05540900
H	-8.37544700	1.88731800	-0.06760400
O	-1.99560500	1.93858800	-0.16216100
C	-1.51612200	3.27302300	0.11436900
H	-2.01495500	3.62339500	1.01988000
H	-0.46411000	3.10313100	0.35002400
C	-1.65720800	4.26250400	-1.01622600
C	-2.16951100	5.54923500	-0.74447100
C	-1.20950300	3.92694400	-2.31019600
C	-2.24384200	6.48217800	-1.78380600
C	-1.31957200	4.88200300	-3.32666500
C	-1.83069800	6.15021200	-3.07149000
H	-2.63320600	7.47471000	-1.57778800
H	-0.97347300	4.62581100	-4.32313700
H	-1.90071900	6.88191100	-3.87046500
C	-0.59503100	2.58825400	-2.62399900
H	0.10124900	2.26470300	-1.84967500
H	-1.34191200	1.79465800	-2.70564200
H	-0.04229300	2.63692200	-3.56469800
C	-2.63123600	5.95755800	0.63793400
H	-2.97689600	6.99340700	0.63338000
H	-3.46243300	5.33885100	0.99798400
H	-1.82898600	5.87844500	1.37856600
C	-1.32977800	0.09807200	2.63338100
C	-1.91926500	-1.02493600	3.23733900
C	-1.31341600	-1.53530500	4.41737500

C	-0.07226600	-1.02226700	4.81757300
C	0.52525100	0.00523200	4.10433500
C	-0.11367700	0.62510200	3.04724400
H	-1.79225800	0.55040700	1.77036100
H	0.42604300	-1.42681600	5.69114400
H	0.34593500	1.45072000	2.52338500
C	-3.12648800	-1.67104600	2.59068900
C	-3.10696400	-3.20159100	2.58622000
H	-3.19328300	-3.62670800	3.58397200
H	-3.97364200	-3.57184400	2.03601800
C	-4.40885000	-0.94350600	2.65255700
C	-5.63834400	-1.58588700	2.36927700
C	-4.45196800	0.44396400	2.92066100
C	-6.83040900	-0.87680200	2.32758700
H	-5.66435600	-2.65167600	2.17904100
C	-5.64602300	1.15314900	2.86535100
H	-3.54269800	0.96544000	3.18631700
C	-6.84103400	0.50112500	2.56190000
H	-7.75563800	-1.39696300	2.10372900
H	-5.64334800	2.21923100	3.06747000
H	-7.77392800	1.05408100	2.52420900
C	-1.93833100	-2.56944700	5.32750200
H	-3.02491800	-2.47075900	5.36935000
H	-1.69740700	-3.59359900	5.02424400
H	-1.55744800	-2.43997700	6.34296000
O	1.82159500	0.46354500	4.47575700
S	3.05020100	-0.53298200	4.04854100
O	3.12486600	-1.68522000	4.92743400
O	3.08671000	-0.71379500	2.59488600
C	4.41623500	0.66933100	4.49124000
F	5.56304900	0.08818100	4.15011800
F	4.38627400	0.90530400	5.79325000
F	4.26110100	1.80052300	3.81285400

RS-IV

Sum of electronic and thermal Free Energies=-4858.953653

C	4.13638800	-2.24864900	-1.75135900
C	5.15133700	-2.73963400	-0.67281800
C	4.03512800	-0.77611800	-1.38179000
C	2.93941400	-3.17458500	-1.88950300
H	4.61422600	-2.90939300	0.26582800
H	5.64087000	-3.67387600	-0.95745300
C	6.12705400	-1.55525600	-0.50438100
C	4.50082800	-3.85964200	-3.57154800

C	5.25049200	-0.36237800	-0.81254100
C	3.03328300	0.16432000	-1.59028800
C	3.18989100	-4.14120600	-2.87697900
C	1.72874500	-3.20611200	-1.20936300
H	6.54806400	-1.50121100	0.50284700
H	6.96784100	-1.62311500	-1.20555900
H	5.29108100	-4.52430500	-3.20005400
H	4.44342500	-4.00706600	-4.65376300
C	5.50045300	0.98408500	-0.57867000
C	3.25684800	1.53558100	-1.37156800
O	1.75686500	-0.25910000	-2.00544100
C	2.27170500	-5.15241000	-3.13358800
C	0.76835200	-4.19945700	-1.46215400
O	1.44630400	-2.17434500	-0.29011800
H	6.44754600	1.30474300	-0.15741800
C	4.51472800	1.92196500	-0.88465100
P	0.71767300	-0.85620600	-0.90809700
H	2.47000300	-5.90071300	-3.89455700
C	1.07654100	-5.18044200	-2.41727400
H	4.69983400	2.97899400	-0.72568600
O	0.71722400	0.11656200	0.34972300
O	-0.60613200	-1.07040200	-1.51845200
H	0.34147100	-5.95398200	-2.61245500
H	1.54756800	0.13456100	0.86717100
C	4.76937300	-2.39561800	-3.17626400
H	4.24007800	-1.72569400	-3.86172700
H	5.82651400	-2.12273400	-3.18957700
C	0.53149000	4.98015600	1.38693800
C	0.26062300	5.26416800	0.07600300
C	0.80957100	4.47194600	-0.98049400
C	1.68948000	3.37346300	-0.65141900
C	1.93812200	3.11289600	0.73316000
C	1.37203000	3.87914700	1.71647300
C	0.50633100	4.73328600	-2.31802900
C	2.24620000	2.58795800	-1.68679300
C	1.93354700	2.86912200	-3.03790200
C	1.04370000	3.96219800	-3.35165200
C	0.74006200	4.24663800	-4.71806100
H	0.05350900	5.05956800	-4.93137400
C	1.29530900	3.51269100	-5.72979500
C	2.18593200	2.44415000	-5.42725000
C	2.49229500	2.13138100	-4.12908000
H	-0.16889100	5.54813800	-2.56212400
H	0.11346600	5.59094200	2.18013100

H	-0.37946600	6.09994800	-0.19102700
H	2.58942100	2.29277700	0.99428300
H	1.56830600	3.64984200	2.75791800
H	1.06255500	3.74090900	-6.76483200
H	2.62835400	1.87350500	-6.23764500
H	3.18032500	1.32262600	-3.91634400
C	-4.09789800	-3.48943400	-3.00907500
C	-4.19416000	-3.85496200	-1.69042500
C	-3.02202900	-4.09251700	-0.91124400
C	-1.72634200	-3.94835500	-1.52962500
C	-1.67788400	-3.50554000	-2.88860600
C	-2.82389800	-3.29238000	-3.60716400
C	-3.10614000	-4.50015100	0.42327500
C	-0.56284400	-4.25000400	-0.79025500
C	-0.66273900	-4.69803700	0.54572100
C	-1.96223300	-4.81427100	1.16355900
C	-2.04881700	-5.26539200	2.51566100
H	-3.03312300	-5.35782900	2.96622200
C	-0.92187900	-5.58486700	3.22687700
C	0.35944400	-5.47783100	2.61815000
C	0.48382300	-5.05512200	1.31957900
H	-4.08411100	-4.60712400	0.88559500
H	-4.99331800	-3.35919200	-3.60879800
H	-5.16296200	-3.98946100	-1.21902100
H	-0.71167400	-3.33969500	-3.34593500
H	-2.76182200	-2.97331100	-4.64260200
H	-1.00118300	-5.93124600	4.25226300
H	1.24401800	-5.74612700	3.18678900
H	1.46274700	-4.99102600	0.85917600
C	-4.14279700	-1.07262000	4.87690000
C	-3.49138300	-0.06885700	2.83731900
C	-3.99527200	-1.41904300	3.38883700
H	-5.03270100	-0.44974300	5.02375400
H	-4.25540100	-1.95789700	5.50712800
H	-4.36359200	0.59373600	2.90631300
H	-4.94363700	-1.72973200	2.94862800
H	-3.28014700	-2.22603400	3.23973600
N	-3.01011600	0.06012600	1.47011300
H	-2.12296500	0.53269500	1.33389100
C	-3.72653400	-0.12737900	0.34459300
C	-4.95621600	-0.79522100	0.28693100
C	-3.16883800	0.42340400	-0.88089200
C	-5.57624100	-1.00681400	-0.93611100
H	-5.42940300	-1.18346500	1.17434900

C	-3.78833800	0.15542200	-2.10781900
C	-4.97516600	-0.54758300	-2.14414900
H	-3.35946500	0.53441700	-3.02097000
H	-5.45073600	-0.73182700	-3.09707800
O	-6.74502200	-1.67348000	-0.89515600
C	-7.50381100	-1.84322800	-2.09517200
H	-6.96567900	-2.46247500	-2.81897900
H	-8.41787800	-2.35166700	-1.79233000
H	-7.75309600	-0.87640500	-2.54362900
O	-2.12454000	1.21137800	-0.68470900
C	-1.37646400	1.78257900	-1.80368600
H	-0.56264900	2.29430200	-1.29331700
H	-0.96818600	0.94310400	-2.35607500
C	-2.16052800	2.72071200	-2.67853700
C	-2.87444000	3.79736600	-2.11741000
C	-2.09581500	2.54643500	-4.07676500
C	-3.52006100	4.69554100	-2.97226200
C	-2.75295600	3.46741600	-4.89877900
C	-3.45973900	4.53585700	-4.35435500
H	-4.07005200	5.52940300	-2.54582100
H	-2.70058200	3.34468200	-5.97631900
H	-3.96174100	5.24426000	-5.00596500
C	-1.32634500	1.40651800	-4.70637600
H	-1.40730100	1.45068200	-5.79415700
H	-0.26296800	1.44155900	-4.45704000
H	-1.69373000	0.42396700	-4.38538300
C	-2.95925600	3.99744400	-0.62469500
H	-1.99589100	3.83200200	-0.13775300
H	-3.28484300	5.01421300	-0.39160900
H	-3.67292400	3.31018600	-0.15864400
C	-0.13916500	1.21830600	4.42897700
C	-0.94345000	0.29363500	3.72949000
C	-0.30270700	-0.83102200	3.15490900
C	1.08620500	-0.96288000	3.29970400
C	1.83035600	-0.02148200	3.97977300
C	1.23884400	1.09047300	4.55620100
H	-0.61298500	2.07089000	4.89752300
H	1.58326900	-1.82026400	2.85996800
H	1.83026700	1.82176600	5.09324900
C	-2.46949700	0.51327300	3.89791400
C	-2.86357600	-0.28666700	5.19474200
H	-2.06050700	-0.99476000	5.41087200
H	-2.94466900	0.36292400	6.06761000
C	-2.79982600	2.01569600	3.93260800

C	-3.74732700	2.54002200	4.81824100
C	-2.25706100	2.87260000	2.96126000
C	-4.13542700	3.87936800	4.74775300
H	-4.20264000	1.90542900	5.57026700
C	-2.64749300	4.20798200	2.88593800
H	-1.49625900	2.51191800	2.27574800
C	-3.58909000	4.71895500	3.77966700
H	-4.86735500	4.26170200	5.45218800
H	-2.20626300	4.84607700	2.12905600
H	-3.89035700	5.75995300	3.72354800
C	-0.94201900	-1.97849800	2.41996800
H	-1.31666900	-2.72918600	3.12117600
H	-1.75213000	-1.69551200	1.75730200
H	-0.19235400	-2.47271200	1.80667600
O	3.22889200	-0.21429900	4.10132200
S	4.13088900	0.03028600	2.75998800
O	5.21478400	-0.92959600	2.75807300
O	3.25349700	0.21001200	1.60723300
C	4.83406800	1.70807800	3.21058400
F	5.62320100	2.13224800	2.23204600
F	5.51869000	1.59543100	4.33845700
F	3.82147500	2.56262600	3.37531300

SS-TS1

Sum of electronic and thermal Free Energies=-4858.912181

C	0.53809800	-4.92618200	-2.44002800
C	-0.12165400	-2.69140800	-2.85077300
C	-0.47622100	-3.76939700	-0.41981700
H	1.50193700	-4.74545000	-1.95328000
H	0.22130700	-5.94218500	-2.19391200
C	0.66154500	-4.66379900	-3.95602800
C	-0.48453000	-3.85315200	-1.93931000
C	-2.26841200	-5.26185400	-0.98070400
C	0.43138800	-3.17246400	-4.04829500
C	-0.28154400	-1.32204500	-2.69519800
C	-1.39364100	-4.68881700	0.11078200
C	0.33186600	-3.05110800	0.45493100
H	1.63265500	-4.96261100	-4.36076700
H	-0.10109300	-5.21481400	-4.52054000
H	-2.01613400	-6.31150500	-1.17476100
H	-3.32911700	-5.22927700	-0.71428200
C	0.71923900	-2.30414300	-5.09646300
C	-0.00976900	-0.41711600	-3.73250100
O	-0.70725600	-0.81560100	-1.45941200

C	-1.40056700	-4.96910800	1.47373900
C	0.36904500	-3.31694100	1.83295000
O	1.11715800	-1.99432600	-0.05907900
H	1.13838700	-2.67897600	-6.02499400
C	0.47267500	-0.93880400	-4.94195300
P	0.36719800	-0.58441300	-0.27070400
H	-2.09820700	-5.69248500	1.88376200
C	-0.49521000	-4.31807600	2.31080800
H	0.68157200	-0.25154600	-5.75486500
O	1.50607200	0.33218400	-0.84545400
O	-0.37592600	-0.02026200	0.88548000
H	-0.46710300	-4.55731300	3.36798100
H	2.39898800	-0.05039300	-1.00595100
C	-1.93893100	-4.36824900	-2.19721000
H	-2.62245000	-3.51621200	-2.22952900
H	-2.02302700	-4.89023200	-3.15291100
C	3.09292400	3.72023200	-3.55481200
C	1.82471400	4.21061600	-3.39545400
C	0.69282300	3.33744300	-3.39405500
C	0.89908400	1.91749000	-3.55277100
C	2.24269200	1.45014800	-3.69830400
C	3.29964900	2.32028000	-3.71207600
C	-0.60579400	3.83135000	-3.23820700
C	-0.21437900	1.04738700	-3.54963400
C	-1.52163600	1.56136300	-3.39193600
C	-1.71478800	2.98224400	-3.22404800
C	-3.04089700	3.49150700	-3.06037800
H	-3.16971900	4.56391800	-2.94160600
C	-4.12489500	2.65731300	-3.07560300
C	-3.94088600	1.26075600	-3.27536600
C	-2.68623200	0.73276200	-3.42852400
H	-0.75680100	4.90209700	-3.13475000
H	3.94378900	4.39386500	-3.56648300
H	1.65291200	5.27742900	-3.28326300
H	2.42110300	0.38688200	-3.79298400
H	4.30573200	1.93995700	-3.84047800
H	-5.12575300	3.05364400	-2.94407500
H	-4.81012100	0.61299100	-3.31988200
H	-2.56170700	-0.32968500	-3.59947900
C	-0.18371100	-0.55604500	6.19448400
C	1.16232000	-0.60787200	5.95815800
C	1.68681900	-1.28071700	4.81130500
C	0.77915800	-1.91006700	3.88218200
C	-0.61889400	-1.80572700	4.15871100

C	-1.08299400	-1.16888500	5.27883500
C	3.06112100	-1.34179500	4.57679000
C	1.29367500	-2.60203400	2.75824600
C	2.69215400	-2.67437600	2.54891800
C	3.58532400	-2.01594000	3.47305700
C	4.99611400	-2.07846900	3.25079000
H	5.65007600	-1.57235700	3.95571100
C	5.51239600	-2.76599400	2.18876200
C	4.63935500	-3.45312200	1.29891200
C	3.28300300	-3.41149200	1.47447600
H	3.74060400	-0.86741300	5.28007500
H	-0.56934300	-0.04687700	7.07147100
H	1.86399500	-0.14329700	6.64477300
H	-1.31760200	-2.24203600	3.46350400
H	-2.15181100	-1.11709200	5.46351800
H	6.58272400	-2.79932600	2.01927400
H	5.06057900	-4.01111700	0.47031100
H	2.64127800	-3.95989900	0.79672700
C	-0.22511100	5.21001300	-0.24860700
C	-1.20654400	2.96242200	0.26187500
C	-1.43733900	4.42710500	0.14221500
H	0.47500000	4.76996500	-0.94512300
H	-0.31984200	6.28920400	-0.27227200
H	-0.18762600	2.58804600	0.27404400
H	-2.26263200	4.58161600	-0.57300300
H	-1.82227900	4.77584900	1.11313200
N	-2.10464800	2.03325000	0.35244300
H	-1.70067800	1.07870600	0.46614500
C	-3.51689500	2.06329700	0.32381100
C	-4.29174300	3.21314900	0.46184400
C	-4.13301600	0.80668500	0.10465000
C	-5.68015800	3.14036400	0.31251400
H	-3.86653500	4.17508900	0.69752400
C	-5.51435600	0.74879000	-0.05042500
C	-6.28619700	1.90857500	0.03721400
H	-5.99150700	-0.20872200	-0.21654500
H	-7.35917800	1.83333000	-0.08357100
O	-6.34011000	4.32029000	0.45297700
C	-7.75960100	4.31049800	0.34615900
H	-8.21314300	3.66809100	1.11009500
H	-8.07768400	5.34051700	0.50392300
H	-8.08366600	3.97904600	-0.64775000
O	-3.29877500	-0.25717300	0.09570800
C	-3.59536800	-1.41189900	-0.71262800

H	-3.88667100	-1.06062700	-1.70090100
H	-2.62084300	-1.88932400	-0.80070400
C	-4.59573900	-2.38992900	-0.14855000
C	-5.56451800	-2.95244200	-1.00629500
C	-4.48499500	-2.82672900	1.18616800
C	-6.40951800	-3.95215800	-0.51186600
C	-5.34819700	-3.82613200	1.64521400
C	-6.30386100	-4.39009600	0.80493700
H	-7.15424500	-4.38921000	-1.17021700
H	-5.26187900	-4.16559900	2.67333300
H	-6.96398400	-5.16846700	1.17435000
C	-3.46561100	-2.24164100	2.12944600
H	-2.49143000	-2.11041100	1.65455000
H	-3.77223900	-1.25349900	2.48802600
H	-3.34266900	-2.89368300	2.99683600
C	-5.71236500	-2.51799500	-2.44871500
H	-4.81463700	-2.72453400	-3.04202600
H	-6.54244400	-3.04602300	-2.92241200
H	-5.91595600	-1.44427900	-2.53462900
C	2.70852800	3.09451200	0.44096800
C	2.32970400	3.06538900	1.79335700
C	3.00147100	2.17534800	2.66903200
C	3.97672300	1.32081500	2.14217400
C	4.31703300	1.38849200	0.79856300
C	3.71465200	2.28413600	-0.06993300
H	2.20771400	3.77148900	-0.23848700
H	4.49802900	0.61903100	2.78067900
H	3.97762500	2.34290700	-1.11511300
C	1.27774700	4.01124400	2.25420000
C	1.26469700	5.28070100	1.75492000
H	2.10237500	5.66388600	1.18497500
H	0.53526800	6.00742800	2.08697100
C	0.15292100	3.54047600	3.09396800
C	-0.60634800	4.43752200	3.87152600
C	-0.25680000	2.19718600	3.04766500
C	-1.75933000	4.01671400	4.52662000
H	-0.28044800	5.46679300	3.97686400
C	-1.42094400	1.78152700	3.68907400
H	0.30364700	1.47508100	2.46738400
C	-2.18227400	2.68774000	4.42490700
H	-2.32626500	4.72485100	5.12300400
H	-1.72109000	0.74485800	3.60382600
H	-3.08806700	2.36338300	4.92750300
C	2.75031400	2.16275800	4.15597000

H	3.61219500	1.74616000	4.68136200
H	2.57214000	3.17279200	4.53265400
H	1.87719400	1.56073100	4.41839000
O	5.40098100	0.55380800	0.39711500
S	5.34162000	-0.39223100	-0.91490800
O	6.07920500	-1.60518100	-0.64477900
O	3.99306700	-0.41849400	-1.49096100
C	6.40734300	0.61443000	-2.08826900
F	6.18034100	0.16767500	-3.32201200
F	7.67861700	0.44990500	-1.76384600
F	6.08734400	1.90738200	-2.01729900

SS-III

Sum of electronic and thermal Free Energies= -4858.961725

C	0.45565500	3.49333900	-2.48501100
C	-0.55375600	4.48306500	-3.15569500
C	0.07539100	2.20156300	-3.19054200
C	0.45068600	3.66947100	-0.97361300
H	-1.51734200	4.40013800	-2.64270800
H	-0.22205100	5.52114000	-3.08315700
C	-0.68904200	3.97407100	-4.60571700
C	2.24419900	5.03731800	-1.79044600
C	-0.47933800	2.48553500	-4.44875000
C	0.22848000	0.87445500	-2.81476900
C	1.36912000	4.66838700	-0.61408500
C	-0.34625100	3.10484700	0.01591200
H	-1.65784600	4.21539300	-5.05220000
H	0.07868100	4.41166800	-5.25620600
H	1.98993500	6.03297100	-2.17395600
H	3.30445200	5.05606000	-1.52145400
C	-0.78145900	1.46084100	-5.33975300
C	-0.05922000	-0.18440900	-3.68913200
O	0.66807400	0.57355300	-1.51837700
C	1.38039500	5.18018100	0.67921900
C	-0.36483700	3.59323900	1.33269200
O	-1.13478800	1.97707500	-0.30902200
H	-1.20132200	1.68326000	-6.31575200
C	-0.54644600	0.13742800	-4.96487500
P	-0.38646700	0.55101200	-0.28795600
H	2.07431400	5.96823500	0.95418100
C	0.49018900	4.67081200	1.62390800
H	-0.76616000	-0.67156800	-5.65332200
O	-1.53203200	-0.45492300	-0.66858200
O	0.38444500	0.19597000	0.93015300

H	0.47160900	5.08244500	2.62679300
H	-2.41710200	-0.11007200	-0.92829700
C	1.91696300	3.93502000	-2.82168500
H	2.58858300	3.08402100	-2.68003700
H	2.01751400	4.26528800	-3.85794000
C	-3.18590500	-4.23308300	-2.92029600
C	-1.92167400	-4.70244500	-2.68551500
C	-0.78465500	-3.84398600	-2.80429900
C	-0.98196100	-2.45857900	-3.15971700
C	-2.32262400	-2.00868200	-3.37213900
C	-3.38379500	-2.86752100	-3.27161000
C	0.51123700	-4.32071700	-2.58917500
C	0.13701700	-1.60423300	-3.28251000
C	1.44206500	-2.10024800	-3.06065700
C	1.62684400	-3.48765600	-2.70581500
C	2.95153800	-3.98404800	-2.49965300
H	3.07522900	-5.03344100	-2.24576300
C	4.04127200	-3.16918700	-2.63880700
C	3.86505000	-1.80650600	-3.00681400
C	2.61210900	-1.29210300	-3.21334700
H	0.65558300	-5.36931600	-2.34185400
H	-4.04044700	-4.89780500	-2.84350600
H	-1.75628300	-5.74351600	-2.42296100
H	-2.49564200	-0.96817700	-3.61376700
H	-4.38715600	-2.50366200	-3.45615600
H	5.04063400	-3.55735000	-2.47641800
H	4.73731000	-1.17632600	-3.14144000
H	2.49404300	-0.25957400	-3.51921600
C	0.35263100	1.59780500	6.07123400
C	-0.99803900	1.57061800	5.85910000
C	-1.56363400	2.03624500	4.63177100
C	-0.69301000	2.52994300	3.59160000
C	0.71313600	2.50532700	3.84752000
C	1.21633700	2.07610000	5.04712700
C	-2.94401700	2.02478300	4.42504300
C	-1.25136700	3.02135400	2.38541500
C	-2.65516700	3.03094000	2.20561500
C	-3.51029200	2.50517900	3.24370000
C	-4.92698300	2.50112700	3.05049800
H	-5.55207000	2.09504900	3.84106400
C	-5.48346500	3.00604200	1.90901300
C	-4.64809900	3.56875600	0.90298700
C	-3.28762100	3.58174500	1.04678300
H	-3.59438300	1.64894800	5.21056100

H	0.76915200	1.25081000	7.01105100
H	-1.67267000	1.20310600	6.62684000
H	1.38745500	2.83959000	3.07553900
H	2.28947700	2.08632400	5.21347100
H	-6.55787500	2.99037800	1.76397000
H	-5.10164200	3.98527200	0.01097700
H	-2.67446600	4.03260800	0.27686300
C	0.01426600	-5.02798500	0.76021600
C	1.19608800	-2.80001100	0.73209400
C	1.35006300	-4.27533800	0.84761700
H	-0.52491100	-4.65110000	-0.11159600
H	0.23076200	-6.08081200	0.55234800
H	0.19751900	-2.37907200	0.67403600
H	2.01333300	-4.60633000	0.03980500
H	1.86807700	-4.49581700	1.78987900
N	2.13077700	-1.90550200	0.67218800
H	1.76668000	-0.93020100	0.65133300
C	3.53945400	-1.98031200	0.62448800
C	4.28553000	-3.14912100	0.75639600
C	4.18006800	-0.74737100	0.35504600
C	5.67001200	-3.11381900	0.56456200
H	3.83851900	-4.10154600	0.98978900
C	5.55704000	-0.72666000	0.16063400
C	6.30184700	-1.90337000	0.25147000
H	6.04795100	0.21702700	-0.04379200
H	7.37249100	-1.86020700	0.09866300
O	6.30129300	-4.31044000	0.69209700
C	7.71709400	-4.34161500	0.54365300
H	8.21055500	-3.70743500	1.28931300
H	8.01026800	-5.37923800	0.69971400
H	8.02104900	-4.02604800	-0.46165500
O	3.38124600	0.34984300	0.34597000
C	3.52288500	1.31892900	-0.72075600
H	3.66911400	0.77291500	-1.65098700
H	2.53779800	1.78208800	-0.75798300
C	4.57612200	2.37465400	-0.50571100
C	5.49207100	2.67557100	-1.53489500
C	4.56368000	3.13237700	0.68281800
C	6.37905800	3.74392200	-1.36093600
C	5.46463500	4.19169700	0.82204100
C	6.36604800	4.50113400	-0.19303300
H	7.08354800	3.98153700	-2.15233700
H	5.45298000	4.77886000	1.73572200
H	7.05754500	5.32932400	-0.07431900

C	3.60748300	2.81369900	1.80278000
H	2.61466800	2.55851900	1.42823900
H	3.94989500	1.95153300	2.38407200
H	3.51199400	3.66642800	2.47875900
C	5.54357700	1.88310500	-2.82291200
H	4.61263500	1.95360500	-3.39650900
H	6.34789600	2.24885800	-3.46445100
H	5.72934000	0.81977900	-2.63570100
C	-2.76269200	-3.08485400	0.82698200
C	-2.22085700	-2.78632500	2.10525100
C	-2.85093000	-1.74075400	2.85771900
C	-3.85937600	-0.98410800	2.25355800
C	-4.31109900	-1.29323900	0.97862400
C	-3.80141600	-2.36705400	0.26185300
H	-2.35143400	-3.89343100	0.23928400
H	-4.33287800	-0.17372800	2.79401000
H	-4.16900500	-2.62766300	-0.71868100
C	-1.11060300	-3.58332800	2.59366600
C	-0.89134300	-4.96637000	2.02060700
H	-1.84901100	-5.42935100	1.76625000
H	-0.43937900	-5.60567900	2.78277100
C	-0.06862100	-3.05637900	3.46618300
C	0.69615800	-3.89908800	4.31155000
C	0.30956900	-1.69355200	3.40070100
C	1.78125200	-3.40984000	5.02888700
H	0.42031800	-4.94071700	4.43050100
C	1.41250600	-1.21705800	4.09610900
H	-0.21859700	-1.01966500	2.73881100
C	2.15697100	-2.06778600	4.91584900
H	2.33686700	-4.07807800	5.67953500
H	1.68914800	-0.17652500	3.98221200
H	3.01710800	-1.69251100	5.46100900
C	-2.56824900	-1.48503000	4.31824600
H	-3.46165000	-1.07909900	4.79841400
H	-2.28862500	-2.40653700	4.83250300
H	-1.75807000	-0.77000500	4.47452100
O	-5.41152100	-0.52875700	0.49578000
S	-5.34716600	0.25687600	-0.92156800
O	-6.05142000	1.51276100	-0.79378400
O	-4.00350700	0.17650300	-1.50543900
C	-6.45220500	-0.85077400	-1.96220100
F	-6.20065400	-0.57999200	-3.24135100
F	-7.71587000	-0.58981900	-1.67381200
F	-6.19176000	-2.13656700	-1.72074300

SS-TS2

B3LYP-D3/6-31G(d,p):

Sum of electronic and thermal Free Energies =-4858.9471

SMD//M062X/6-31+G(d,p):

Sum of electronic and thermal Free Energies = -4856.906278

C	-3.46281200	2.30641900	2.07118100
C	-4.90891700	1.73097200	2.22476700
C	-3.35933000	2.39039900	0.56089300
C	-2.47844800	1.54477700	2.94305200
H	-4.87019200	0.64479700	2.10019600
H	-5.33583900	1.94366000	3.20742000
C	-5.70104900	2.35874400	1.05792000
C	-3.16498900	3.45327000	4.22018600
C	-4.63598600	2.54547500	0.00234400
C	-2.25219300	2.36973100	-0.27355100
C	-2.40951500	2.14427600	4.21089000
C	-1.75212500	0.38252400	2.71366700
H	-6.51629500	1.71698100	0.71006000
H	-6.14974500	3.31821500	1.34384100
H	-4.12093200	3.34902700	4.74840900
H	-2.61159400	4.25176800	4.72348500
C	-4.78355300	2.80085600	-1.35634800
C	-2.36422100	2.58630500	-1.65916500
O	-0.99512300	2.06706800	0.28346900
C	-1.70182100	1.54213100	5.24529400
C	-1.06184700	-0.27351800	3.74231000
O	-1.66936600	-0.14350000	1.40733700
H	-5.76784100	2.95037800	-1.78932200
C	-3.64952000	2.85213300	-2.16449600
P	-0.56342900	0.51680900	0.42473100
H	-1.65405400	2.00475800	6.22618300
C	-1.06395300	0.32534500	5.01313500
H	-3.75052900	3.06088400	-3.22361100
O	-0.86743000	-0.20801400	-0.94361000
O	0.86171700	0.41334500	0.81421900
H	-0.53973800	-0.17737100	5.81836700
H	-1.82042600	-0.38818500	-1.09923300
C	-3.37997800	3.72619700	2.71806400
H	-2.50922200	4.24659700	2.30532000
H	-4.26446600	4.33015600	2.50354800
C	-1.36521300	-0.12849800	-5.96372500
C	-0.26388000	0.65735200	-5.75482500

C	-0.17566700	1.52566100	-4.62247900
C	-1.27465000	1.58718900	-3.68713500
C	-2.38703400	0.71781500	-3.92760900
C	-2.43760800	-0.09523100	-5.03026600
C	0.96210500	2.30600700	-4.40057100
C	-1.20496200	2.48247900	-2.58694300
C	-0.05334300	3.28144300	-2.39303900
C	1.06310100	3.15884900	-3.30142400
C	2.23889600	3.94030500	-3.07850000
H	3.07104500	3.82523700	-3.76718700
C	2.30485500	4.82306500	-2.03747900
C	1.18937700	4.98564900	-1.16928400
C	0.05247300	4.24697000	-1.34404900
H	1.78491200	2.25979400	-5.10964600
H	-1.42623100	-0.77128300	-6.83593300
H	0.56424900	0.64552300	-6.45801600
H	-3.20632900	0.70178000	-3.22218500
H	-3.30839500	-0.72304600	-5.19438400
H	3.19672400	5.41783000	-1.87372800
H	1.24843300	5.70083800	-0.35885300
H	-0.78912400	4.39904600	-0.68156700
C	3.70060600	-2.06569000	4.59112000
C	2.93836200	-3.16975100	4.32081800
C	1.56761400	-3.04364100	3.93473900
C	0.97601500	-1.72916400	3.86235000
C	1.82393400	-0.60153400	4.10392400
C	3.13807300	-0.76338700	4.45811200
C	0.78424000	-4.16329400	3.64704000
C	-0.39952800	-1.59507100	3.56752500
C	-1.17972800	-2.73706000	3.26284200
C	-0.56492500	-4.04300800	3.30039900
C	-1.35821400	-5.19610500	3.01179100
H	-0.87981300	-6.17055500	3.04961200
C	-2.68847400	-5.08174900	2.70823500
C	-3.30256800	-3.79754500	2.68095900
C	-2.57547500	-2.66898900	2.95505800
H	1.23009800	-5.15263400	3.70548000
H	4.73726400	-2.17524500	4.89334800
H	3.36112400	-4.16731600	4.39406200
H	1.40637000	0.39128700	4.00023600
H	3.75872900	0.10830200	4.64208800
H	-3.28090400	-5.96610200	2.49666900
H	-4.35605800	-3.70796800	2.43968600
H	-3.07101800	-1.70768600	2.94286800

C	2.02387900	-2.14399400	-4.70206700
C	1.92220300	-0.73124200	-2.65864700
C	2.50714600	-0.84658400	-4.05391000
H	0.99278500	-2.03057800	-5.04329600
H	2.61579400	-2.38565100	-5.58936100
H	0.86779100	-0.47900000	-2.63631200
H	2.16890400	0.02334400	-4.62064900
H	3.59474900	-0.80380500	-4.03752500
N	2.57559600	-0.00167800	-1.68910400
H	1.98544900	0.46039000	-0.99101900
C	3.89919200	-0.03471700	-1.30909300
C	4.84346200	-0.92210700	-1.84145800
C	4.27259800	0.81822500	-0.21341100
C	6.09174700	-1.06523200	-1.25393400
H	4.59326100	-1.60352900	-2.63524800
C	5.53556300	0.66034900	0.35623100
C	6.43738400	-0.27467000	-0.14102600
H	5.83861400	1.27635100	1.19055900
H	7.40776600	-0.36984200	0.32835100
O	6.89323200	-2.00909700	-1.80498000
C	8.12041100	-2.31880200	-1.15229300
H	7.94603400	-2.64883700	-0.12145100
H	8.56460800	-3.13243000	-1.72477600
H	8.80425400	-1.46246800	-1.15361300
O	3.35642600	1.73529000	0.15160900
C	3.28987100	2.19707100	1.53359300
H	2.53024200	1.57695000	2.00354600
H	4.24629600	2.00466400	2.02377000
C	2.96072100	3.66611000	1.60476500
C	3.83845700	4.61156200	1.03098100
C	1.82881000	4.09756000	2.32520200
C	3.56989300	5.97376500	1.18416400
C	1.59027700	5.47166400	2.45440700
C	2.45257100	6.40721700	1.89411500
H	4.24936900	6.69915300	0.74649900
H	0.71896300	5.80355800	3.01123600
H	2.25779500	7.46885500	2.00993000
C	0.88370900	3.13775100	3.00699600
H	-0.03235500	3.64917700	3.30737900
H	0.60372700	2.30684100	2.36194000
H	1.32632400	2.71744100	3.91823400
C	5.05940400	4.19128800	0.25124200
H	4.78266500	3.66354500	-0.66537700
H	5.65987400	5.06160400	-0.02245100

H	5.70354500	3.52109700	0.82985300
C	-0.69279300	-2.44055600	-2.83498200
C	0.26312800	-2.96488000	-1.93888800
C	-0.22265300	-3.69377300	-0.81388300
C	-1.59426700	-3.64445300	-0.51992900
C	-2.47435900	-3.02161800	-1.38334300
C	-2.05629100	-2.48347100	-2.59000000
H	-0.37674000	-1.92902800	-3.73202600
H	-1.97252300	-4.12522900	0.37360200
H	-2.75970600	-2.04655800	-3.28308900
C	1.72534700	-2.77797600	-2.24964900
C	2.15403300	-3.25495700	-3.64487000
H	1.57915200	-4.14030300	-3.93292800
H	3.20482000	-3.54844900	-3.61436500
C	2.67837300	-2.98455200	-1.13140400
C	3.79462300	-3.84173800	-1.21304600
C	2.48876300	-2.26774500	0.06792200
C	4.68700400	-3.95749600	-0.15202900
H	3.95886300	-4.44833700	-2.09527400
C	3.40305200	-2.34849600	1.10611800
H	1.63604400	-1.61011500	0.17497100
C	4.51173800	-3.19171800	1.00205100
H	5.52721700	-4.63932900	-0.23439200
H	3.25206200	-1.74465500	1.99012100
H	5.21705600	-3.26201400	1.82368300
C	0.59784800	-4.61384200	0.06275500
H	-0.04977600	-5.40848700	0.43853500
H	1.42117200	-5.07101100	-0.48480800
H	1.02380600	-4.10580900	0.92913800
O	-3.85166600	-2.97938700	-1.02796300
S	-4.47147900	-1.53601500	-0.58067600
O	-5.07649500	-1.64350800	0.73256100
O	-3.54360900	-0.45510200	-0.91440600
C	-5.83251600	-1.40098900	-1.85238000
F	-5.27839800	-1.34823700	-3.06546700
F	-6.49032200	-0.27069400	-1.61029400
F	-6.64598800	-2.44042200	-1.76547400

SS-IV

Sum of electronic and thermal Free Energies = -4858.958892

C	0.24434900	4.11369500	1.57519900
C	1.34453300	5.05757600	2.16248700
C	0.20297700	3.04761500	2.66505800
C	0.51605900	3.80919200	0.11138100

H	2.32740500	4.64078400	1.91992000
H	1.28949800	6.06437200	1.74174000
C	1.11737600	5.00862700	3.68872400
C	-1.02406500	5.63898400	0.09948700
C	0.59100200	3.60579800	3.89551600
C	-0.15620800	1.70694100	2.63944900
C	-0.09825600	4.76165300	-0.71344300
C	1.35218700	2.86628500	-0.46791400
H	2.03047400	5.19900700	4.25985000
H	0.38151200	5.75760700	4.00744900
H	-0.60211700	6.64503800	0.21445900
H	-2.00530800	5.75391000	-0.36902100
C	0.50551700	2.86964100	5.07281100
C	-0.29176900	0.94646700	3.81092600
O	-0.38851000	1.06023400	1.42191600
C	0.20119600	4.81077100	-2.07308600
C	1.73977900	2.93154800	-1.81256500
O	1.82778700	1.81463000	0.32271500
H	0.80446400	3.30820600	6.01975600
C	0.03205500	1.55515500	5.02943700
P	0.84121500	0.54103500	0.50257600
H	-0.27679300	5.54450100	-2.71423400
C	1.14520000	3.92569900	-2.60350500
H	-0.06805300	0.97900800	5.94335300
O	1.72553900	-0.32543700	1.48800100
O	0.26152700	-0.06485300	-0.71561200
H	1.43246900	3.99657500	-3.64743300
H	2.62569900	-0.47636000	1.12223600
C	-1.10045300	4.90618200	1.45998200
H	-1.93343100	4.20028300	1.45576600
H	-1.25139900	5.58150000	2.30517100
C	1.73230300	-3.80879600	4.26471700
C	0.40628600	-4.00378100	3.98919800
C	-0.47949600	-2.89440200	3.82167300
C	0.04647600	-1.55274200	3.93355700
C	1.43793700	-1.39887500	4.22637100
C	2.25071900	-2.48751000	4.39134700
C	-1.83746800	-3.08186500	3.54652800
C	-0.81043200	-0.45107000	3.73186300
C	-2.18055700	-0.65570800	3.44212300
C	-2.70246500	-1.99904700	3.36128900
C	-4.09376900	-2.18863100	3.09496700
H	-4.47917000	-3.20334900	3.06275900
C	-4.92969900	-1.12384800	2.89831000

C	-4.41987300	0.20228600	2.98044900
C	-3.09468800	0.42663900	3.25158100
H	-2.23075900	-4.09360700	3.48811900
H	2.39420800	-4.65899100	4.39447600
H	-0.00046400	-5.00710100	3.90056700
H	1.84304100	-0.39777400	4.29905000
H	3.30350300	-2.34779100	4.61094400
H	-5.98261400	-1.28393300	2.69216400
H	-5.09654700	1.04188600	2.85541200
H	-2.72384300	1.44020900	3.34618200
C	2.26336100	-1.05479500	-5.13234400
C	3.51044400	-0.83266300	-4.61573800
C	3.73534200	0.20289900	-3.65764200
C	2.62303400	1.01668400	-3.22396600
C	1.33272800	0.72466000	-3.76412300
C	1.16264300	-0.26549300	-4.69553000
C	5.00992800	0.44727200	-3.14488000
C	2.84593800	2.05614200	-2.29813300
C	4.14750400	2.30373100	-1.79595300
C	5.24418500	1.47367600	-2.22656800
C	6.55022200	1.70915700	-1.70088500
H	7.35703800	1.06027200	-2.02478300
C	6.77703900	2.71621100	-0.80266300
C	5.70440600	3.55520700	-0.39365600
C	4.43675400	3.35905100	-0.87537700
H	5.83973600	-0.17747900	-3.46132200
H	2.10598000	-1.83118200	-5.87398200
H	4.36081500	-1.42677100	-4.93869800
H	0.48732500	1.30578600	-3.42247700
H	0.17871600	-0.46140200	-5.10591300
H	7.77359500	2.88526000	-0.40772100
H	5.89640900	4.36516000	0.30330100
H	3.63826500	4.02108400	-0.56418500
C	-2.00131300	-5.55169600	0.27384700
C	-2.06918200	-3.20892300	0.04236300
C	-2.99400100	-4.39112600	0.33856900
H	-1.31016800	-5.50789300	1.12377500
H	-2.48793200	-6.52951500	0.29190100
H	-1.44359700	-3.10519000	0.92732700
H	-3.51952700	-4.28458100	1.28815500
H	-3.72720400	-4.54400800	-0.45332700
N	-2.61664200	-1.86489600	-0.13124500
H	-1.90678300	-1.13080500	-0.10271300
C	-3.86361600	-1.41553900	-0.35018800

C	-5.01856700	-2.20631800	-0.50626800
C	-4.02803000	0.03458800	-0.38123600
C	-6.26682900	-1.62695900	-0.64411200
H	-4.97172300	-3.27871800	-0.51404700
C	-5.30234100	0.59709000	-0.51389300
C	-6.41573300	-0.21128900	-0.64234400
H	-5.40506000	1.67121400	-0.55714800
H	-7.39073300	0.24307100	-0.75877700
O	-7.30037500	-2.48248000	-0.77551800
C	-8.62218300	-1.96733700	-0.94571200
H	-8.69864900	-1.36368100	-1.85618900
H	-9.26626000	-2.84084000	-1.03534900
H	-8.93260500	-1.37479200	-0.07839500
O	-2.89805000	0.72303200	-0.28292600
C	-2.89324300	2.11496300	0.15074900
H	-3.39975300	2.13578300	1.11391300
H	-1.83362800	2.28460700	0.31423300
C	-3.44779000	3.13168700	-0.81015400
C	-4.39239800	4.07773000	-0.35665900
C	-2.92860700	3.20390700	-2.11712300
C	-4.80578600	5.08816900	-1.23185700
C	-3.36675900	4.22702000	-2.96232500
C	-4.29662200	5.16617700	-2.52549400
H	-5.52970400	5.82137300	-0.88968700
H	-2.96609600	4.28651100	-3.96978000
H	-4.62440600	5.95858700	-3.19085400
C	-1.93214400	2.19238300	-2.61413500
H	-1.13037200	2.00639900	-1.89577900
H	-2.41302300	1.22812800	-2.79320000
H	-1.47983700	2.52992700	-3.54846300
C	-4.95973700	4.04934600	1.04643600
H	-4.19413900	4.23658400	1.80722000
H	-5.72545700	4.81891100	1.16211100
H	-5.42409900	3.08659300	1.28917600
C	0.87914100	-3.19065100	0.23625800
C	0.32721700	-3.36490200	-1.03908800
C	1.21450600	-3.44621900	-2.14371200
C	2.58110000	-3.22727900	-1.93227300
C	3.07302500	-3.04344900	-0.65110800
C	2.24560600	-3.05993900	0.45432400
H	0.26161900	-3.17538700	1.12241400
H	3.27008300	-3.24199700	-2.76835200
H	2.63274500	-2.96655200	1.45976000
C	-1.16741700	-3.70894100	-1.18195700

C	-1.25722900	-5.27831300	-1.04323000
H	-0.26066200	-5.71981800	-1.05470200
H	-1.80444000	-5.71402900	-1.88015200
C	-1.84560400	-3.12745100	-2.42714600
C	-2.91541600	-3.77373500	-3.05762400
C	-1.50703400	-1.83557400	-2.85977100
C	-3.61708800	-3.16646700	-4.10129300
H	-3.22233900	-4.76577400	-2.74877100
C	-2.20213700	-1.23492000	-3.90763300
H	-0.69547700	-1.29877300	-2.37936000
C	-3.26138100	-1.89280400	-4.53431900
H	-4.43799300	-3.69712000	-4.57325000
H	-1.91169500	-0.24498900	-4.24008100
H	-3.79711700	-1.41892700	-5.35057000
C	0.79743600	-3.82872700	-3.54538400
H	1.66044100	-4.21459100	-4.09243400
H	0.02445000	-4.59943600	-3.54670300
H	0.40243800	-2.97674900	-4.10104800
O	4.48131900	-2.95035800	-0.49482400
S	5.15586800	-1.53732900	-0.04985000
O	6.32720700	-1.32020300	-0.86968700
O	4.12788600	-0.51356200	0.14911000
C	5.76265000	-2.01527400	1.65189200
F	6.48277100	-1.00712400	2.12805200
F	6.49598600	-3.11533800	1.57919900
F	4.70703700	-2.22453000	2.44934500

SR-TS1

Sum of electronic and thermal Free Energies = -4858.916863

C	-0.74026100	-0.65998800	-4.09332400
C	0.21130700	-1.00240900	-5.28611300
C	-0.23611500	0.73349600	-3.73422600
C	-0.80033400	-1.82604800	-3.12126100
H	1.16665300	-1.35413500	-4.88325300
H	-0.19659300	-1.78993000	-5.92339000
C	0.41286300	0.34189400	-6.01067300
C	-2.65963400	-2.11394400	-4.60701200
C	0.31945700	1.33519900	-4.87649700
C	-0.27035400	1.46017300	-2.54697000
C	-1.80655300	-2.71940100	-3.51742900
C	0.01111200	-2.15958900	-2.04514600
H	1.36825000	0.40322300	-6.53951500
H	-0.37446400	0.51803800	-6.75416700
H	-2.46033300	-2.59347700	-5.57327700

H	-3.72772900	-2.22850000	-4.40229700
C	0.73268700	2.66186600	-4.85668900
C	0.14897300	2.80017700	-2.49032800
O	-0.70551200	0.83261400	-1.36387900
C	-1.90433000	-3.97289700	-2.92258500
C	-0.06851700	-3.40657500	-1.40378300
O	0.91665400	-1.18727700	-1.56793600
H	1.14944700	3.12510200	-5.74550100
C	0.62182600	3.38906600	-3.67389500
P	0.33104100	-0.10277200	-0.52946400
H	-2.66263100	-4.67861100	-3.24656400
C	-1.01923000	-4.31613600	-1.90075800
H	0.94021600	4.42539900	-3.63955000
O	1.59575500	0.79226800	-0.24389900
O	-0.38785100	-0.63420800	0.65275100
H	-1.07440300	-5.29820600	-1.44429100
H	2.44880300	0.54144900	-0.66227200
C	-2.21690200	-0.63472500	-4.60498300
H	-2.82885300	-0.07135800	-3.89947100
H	-2.30503300	-0.15340900	-5.58137400
C	3.82322500	4.92034900	0.45712400
C	2.64064700	5.34117100	1.00193000
C	1.38906600	4.93546200	0.44330400
C	1.37915400	4.04177100	-0.69234800
C	2.63984200	3.61536600	-1.21562800
C	3.81731000	4.05336400	-0.67226700
C	0.17733500	5.39321200	0.96972800
C	0.14568400	3.62411600	-1.24489300
C	-1.07011600	4.08284100	-0.69066900
C	-1.04817000	4.99445600	0.43090500
C	-2.28798300	5.45330300	0.97932500
H	-2.25583600	6.14121800	1.81934900
C	-3.48616100	5.03742000	0.46160300
C	-3.51052100	4.14765600	-0.65059100
C	-2.34515700	3.69576000	-1.21081900
H	0.18976900	6.08095700	1.81135600
H	4.76839500	5.24808200	0.87781800
H	2.63027200	6.00896600	1.85840600
H	2.65605600	2.93429000	-2.05609000
H	4.75718600	3.73622700	-1.10555100
H	-4.42181700	5.38010600	0.89147700
H	-4.46467800	3.83383300	-1.05900200
H	-2.37612800	3.03930600	-2.07059500
C	-0.89167300	-5.09437800	3.43444500

C	0.46664700	-5.06138400	3.27944400
C	1.06143500	-4.61981500	2.05727100
C	0.21476000	-4.18506100	0.97162100
C	-1.19673300	-4.20014400	1.19832600
C	-1.73152100	-4.65537900	2.37419000
C	2.44789700	-4.61718700	1.89146500
C	0.80137400	-3.78240100	-0.25439600
C	2.20693300	-3.81030900	-0.41258600
C	3.04043900	-4.22496200	0.69105400
C	4.46052200	-4.25070000	0.52571300
H	5.06984200	-4.56810900	1.36778000
C	5.03842500	-3.90207000	-0.66285400
C	4.21871100	-3.53319700	-1.76711000
C	2.85656100	-3.49055000	-1.64637800
H	3.07947800	-4.94978700	2.71078900
H	-1.33105000	-5.44143500	4.36370600
H	1.12413500	-5.38437300	4.08137600
H	-1.85594000	-3.84723000	0.42145200
H	-2.80931700	-4.66176000	2.50522600
H	6.11649600	-3.91438900	-0.77883400
H	4.68632700	-3.28077100	-2.71143500
H	2.25340600	-3.22836900	-2.50617300
C	-0.33379300	2.68290900	3.62376200
C	-1.65314300	2.14419900	1.83351300
C	-0.22265500	2.14497400	2.22444600
H	-0.94524900	2.12249100	4.32130600
H	-0.28103800	3.75707300	3.77189200
H	-2.16954000	3.09164700	1.74632100
H	0.19540000	1.14183600	2.18975200
H	0.35961300	2.80439300	1.58290400
N	-2.36908300	1.05564100	1.74664200
H	-1.86954800	0.16004000	1.65286400
C	-3.78075000	1.00130400	1.66001000
C	-4.57263800	2.10111000	1.97903200
C	-4.36434300	-0.21119000	1.25137800
C	-5.96078900	2.02136200	1.84022000
H	-4.14951800	3.02997000	2.33707000
C	-5.75102800	-0.29013600	1.15918600
C	-6.55370400	0.81528700	1.43831600
H	-6.19579300	-1.22495500	0.84185700
H	-7.62816500	0.72636000	1.34584800
O	-6.63819400	3.16157800	2.13338000
C	-8.06022400	3.13798600	2.05005000
H	-8.48784700	2.40558800	2.74428100

H	-8.39158600	4.13765300	2.32925000
H	-8.39639000	2.91425100	1.03083800
O	-3.55721100	-1.27811400	0.95074100
C	-3.21901500	-1.37730800	-0.47263400
H	-2.65146500	-0.49179900	-0.74803300
H	-2.54283100	-2.22592000	-0.51985800
C	-4.41173000	-1.57221200	-1.36806000
C	-4.91079200	-0.50492300	-2.14192900
C	-5.02998700	-2.83949000	-1.42345300
C	-5.97465300	-0.74301900	-3.02046800
C	-6.09722100	-3.03967300	-2.30238500
C	-6.55871200	-2.00256200	-3.11095400
H	-6.35207400	0.07363900	-3.62885700
H	-6.57097500	-4.01581700	-2.34930800
H	-7.38233500	-2.17284400	-3.79732500
C	-4.57030900	-3.97034900	-0.53509400
H	-3.60964300	-4.37406400	-0.86929700
H	-4.44157400	-3.63577400	0.49772200
H	-5.29287100	-4.78939100	-0.54530900
C	-4.37730700	0.90294900	-2.01158100
H	-4.72873700	1.36476600	-1.08316700
H	-3.28735100	0.94724300	-1.98966400
H	-4.72243300	1.52339100	-2.84273400
C	3.06172600	1.39775000	2.56789400
C	2.62139300	0.36888900	3.42172600
C	3.19370800	-0.92237000	3.28253300
C	4.12449700	-1.13700400	2.25956700
C	4.52267900	-0.09165500	1.44114300
C	4.02761100	1.19510100	1.59371700
H	2.62476500	2.38517300	2.65051100
H	4.56332700	-2.11514500	2.10907400
H	4.33779600	2.01126500	0.95870600
C	1.60723200	0.68890000	4.45228700
C	1.54386800	1.95015300	4.98132100
H	2.32790600	2.67615900	4.80097900
H	0.85211400	2.18146300	5.78066500
C	0.49726300	-0.24221400	4.75198600
C	-0.10118500	-0.28042300	6.02342600
C	-0.09013500	-0.98266400	3.71182600
C	-1.26907600	-1.00879900	6.23708200
H	0.36087200	0.25193600	6.84876200
C	-1.28006800	-1.67539000	3.91671000
H	0.35716900	-0.99054300	2.72591100
C	-1.87354100	-1.69213800	5.17973900

H	-1.71041800	-1.03731500	7.22840500
H	-1.73489800	-2.19892500	3.08519500
H	-2.79530400	-2.24195400	5.34210200
C	2.88961800	-2.06422100	4.22139600
H	3.71353100	-2.78125000	4.21554600
H	2.74801800	-1.71288000	5.24540300
H	1.98037900	-2.59664900	3.93253000
O	5.53968100	-0.40055000	0.49514500
S	5.39283800	-0.02790500	-1.07636400
O	5.97791400	-1.09025100	-1.86286700
O	4.06139700	0.51250100	-1.36662800
C	6.59973900	1.40790500	-1.16391500
F	6.34329200	2.08458100	-2.28196700
F	7.83358300	0.93370400	-1.18394900
F	6.44195600	2.21117600	-0.11049200

SR-III

Sum of electronic and thermal Free Energies = -4858.95422

C	0.41713900	4.07517900	-1.05457000
C	-0.62963300	5.23738100	-1.09671800
C	-0.13799700	3.17589900	-2.15118900
C	0.66231700	3.63815200	0.38299600
H	-1.51220600	4.93384500	-0.52434500
H	-0.24150800	6.15741300	-0.65480200
C	-0.99459500	5.37073000	-2.58852300
C	2.35322100	5.26391800	-0.11484500
C	-0.84563700	3.95145600	-3.08456300
C	-0.02656300	1.80786900	-2.37012900
C	1.67004600	4.43523800	0.94708500
C	0.00110300	2.72255400	1.19239100
H	-2.00293200	5.76342600	-2.74753900
H	-0.30460600	6.04535100	-3.11061500
H	2.06890500	6.32016300	-0.03074000
H	3.44193700	5.21506500	-0.03692700
C	-1.33165500	3.38535600	-4.25783400
C	-0.51548400	1.19920200	-3.53772200
O	0.57271300	1.00516700	-1.38731600
C	1.91154900	4.39934300	2.31610700
C	0.21154700	2.65783600	2.58065200
O	-0.90365600	1.82050300	0.58684500
H	-1.86524600	3.99040800	-4.98413100
C	-1.14268400	2.02337500	-4.48618100
P	-0.28930500	0.50824600	-0.11203000
H	2.67485600	5.03230200	2.75707000

C	1.15717300	3.54749000	3.12100700
H	-1.51823900	1.56702200	-5.39564400
O	-1.57950400	-0.21042200	-0.66336400
O	0.60012500	-0.34289600	0.71998700
H	1.31204100	3.54023400	4.19403800
H	-2.44737900	0.24234800	-0.57103500
C	1.82596700	4.64121500	-1.42423200
H	2.47589100	3.81529300	-1.72195000
H	1.78166300	5.34648900	-2.25699600
C	-4.02208800	-2.54831800	-4.09742600
C	-2.81593800	-3.14526000	-4.34559100
C	-1.59645900	-2.40548200	-4.25134500
C	-1.64284800	-1.00871900	-3.88680000
C	-2.92545400	-0.43380900	-3.62036300
C	-4.07217400	-1.17356500	-3.73163500
C	-0.36032000	-3.01623200	-4.47978300
C	-0.43935700	-0.26933800	-3.78912500
C	0.80526600	-0.91030200	-3.98941500
C	0.83899200	-2.31586900	-4.32588800
C	2.10107600	-2.97543000	-4.44658600
H	2.10691900	-4.03573600	-4.68325300
C	3.27344900	-2.28981400	-4.27624400
C	3.24471900	-0.89166000	-4.01250600
C	2.05550000	-0.22575100	-3.87997400
H	-0.32955900	-4.06852100	-4.74950000
H	-4.94285400	-3.11674900	-4.18036200
H	-2.76217500	-4.19270500	-4.62828300
H	-2.98481900	0.60269100	-3.31595800
H	-5.03049800	-0.70821100	-3.53796400
H	4.22702000	-2.79972600	-4.36670100
H	4.18181400	-0.35142400	-3.93131000
H	2.04771300	0.84019400	-3.69232300
C	1.50993600	-0.96356400	6.07553000
C	0.14235500	-0.94649900	6.04897200
C	-0.57070600	-0.05696200	5.18715700
C	0.16468600	0.81700100	4.30388100
C	1.59108600	0.73477500	4.34240700
C	2.24078100	-0.10452500	5.20960400
C	-1.96611500	-0.00263800	5.20355800
C	-0.53687800	1.72087100	3.46631800
C	-1.94875000	1.79346200	3.53160000
C	-2.67041200	0.90415000	4.41118400
C	-4.09636500	0.98898600	4.47798400
H	-4.62013000	0.31075600	5.14632100

C	-4.78309500	1.91120100	3.73897700
C	-4.07264300	2.82119000	2.90576700
C	-2.70986900	2.76111100	2.80327200
H	-2.51430400	-0.66004300	5.87301600
H	2.03868000	-1.63245000	6.74645600
H	-0.43261400	-1.59884500	6.69975100
H	2.16206400	1.35485500	3.67122500
H	3.32629800	-0.12974700	5.22016700
H	-5.86486800	1.96717500	3.79034600
H	-4.62460800	3.56560100	2.34491300
H	-2.19055400	3.47756700	2.17969700
C	-0.50056700	-4.57239400	-1.36792400
C	1.60730300	-3.21754000	-0.87001700
C	0.15515900	-3.19567900	-1.14777500
H	-1.25963600	-4.43740700	-2.14080400
H	0.23477300	-5.26087900	-1.79879100
H	2.21305800	-4.03343500	-1.25842700
H	-0.37282400	-2.58324500	-0.41827500
H	0.08545700	-2.64438300	-2.09285000
N	2.23956000	-2.24510200	-0.30459500
H	1.68281000	-1.42937200	0.05493700
C	3.65702100	-2.16387100	-0.19468800
C	4.42761900	-3.28985900	0.05776400
C	4.24511000	-0.90284800	-0.41099400
C	5.82351600	-3.18533300	0.07254900
H	3.96499800	-4.24212900	0.28690000
C	5.63550400	-0.81042100	-0.40060800
C	6.42139800	-1.94196000	-0.16676800
H	6.10984000	0.15083700	-0.55642700
H	7.49826900	-1.83435500	-0.15518600
O	6.49374800	-4.33704100	0.33786600
C	7.91524500	-4.28616100	0.39253200
H	8.26047900	-3.60582000	1.18006500
H	8.24153400	-5.30014700	0.62165000
H	8.34149000	-3.97600000	-0.56897900
O	3.38336800	0.12556900	-0.60714100
C	3.82782600	1.28050700	-1.31688500
H	4.40104200	0.94290500	-2.18441400
H	2.89809200	1.71812600	-1.68759800
C	4.62248300	2.32188300	-0.55408700
C	5.33260200	3.25135000	-1.34879900
C	4.62297000	2.44386400	0.84824000
C	6.05052800	4.27834300	-0.73148100
C	5.36212500	3.48400200	1.42849800

C	6.07370600	4.39399400	0.65564100
H	6.59373400	4.98891800	-1.34719600
H	5.36696900	3.57621500	2.51052700
H	6.63701100	5.19170200	1.12924400
C	3.86741300	1.51810200	1.76969500
H	2.82595300	1.38876600	1.47191900
H	4.31655300	0.52095600	1.80907300
H	3.89205600	1.92811400	2.78051900
C	5.31888000	3.17104100	-2.85875600
H	4.30160700	3.19476800	-3.26673300
H	5.86461100	4.01161800	-3.29182500
H	5.78947200	2.25131400	-3.22767800
C	-3.08786300	-3.08880100	-0.08617000
C	-2.40498200	-3.38282800	1.12223200
C	-2.87542100	-2.74842900	2.31460900
C	-3.85706100	-1.75761000	2.21307300
C	-4.42910600	-1.45256500	0.98608500
C	-4.09230400	-2.14237200	-0.17041200
H	-2.80196500	-3.58020200	-1.00499900
H	-4.20306500	-1.23462500	3.09598400
H	-4.56040100	-1.93241000	-1.12022700
C	-1.27190000	-4.29097000	1.06655900
C	-1.17211200	-5.22972000	-0.11038500
H	-2.16389900	-5.59105400	-0.39205200
H	-0.60129400	-6.11469000	0.17156100
C	-0.04969400	-4.08808400	1.82791600
C	0.93358100	-5.10556600	1.94129100
C	0.29148200	-2.81202600	2.33338200
C	2.17064000	-4.85796700	2.52735100
H	0.71598000	-6.11008400	1.59782900
C	1.53810000	-2.56127200	2.88792600
H	-0.39054500	-1.98465600	2.20197300
C	2.48641900	-3.58066600	2.99730300
H	2.89037600	-5.66621900	2.61748400
H	1.77253000	-1.55694800	3.21258200
H	3.46033600	-3.38035000	3.43179900
C	-2.42088300	-3.16096600	3.69385500
H	-3.24601900	-3.05007900	4.40221100
H	-2.09414700	-4.20236900	3.70529500
H	-1.58893100	-2.55426700	4.05930000
O	-5.45616000	-0.46704500	0.98690100
S	-5.37198800	0.82742800	0.01012300
O	-5.90005900	1.97618500	0.71162800
O	-4.08842300	0.86964200	-0.69760600

C	-6.67768100	0.36255200	-1.25887900
F	-6.47322800	1.11538200	-2.33846800
F	-7.87494500	0.59901900	-0.75053300
F	-6.57663300	-0.92583300	-1.59073000

SR-TS2

B3LYP-D3/6-31G(d,p):

Sum of electronic and thermal Free Energies =-4858.932184

SMD//M062X/6-31+G(d,p):

Sum of electronic and thermal Free Energies = -4856.90246

C	0.98856100	3.85619800	2.71163000
C	2.26644600	4.63097200	3.17351100
C	1.17485200	2.54846800	3.46905500
C	0.83167900	3.95493300	1.20415500
H	3.11374900	4.30165900	2.56328400
H	2.16034500	5.71094100	3.04831000
C	2.47373900	4.19117100	4.63616600
C	-0.51312000	5.71518000	2.11429000
C	1.93086800	2.78094700	4.63033800
C	0.70497100	1.26226100	3.22956000
C	0.09289300	5.09956500	0.87384600
C	1.37504200	3.18768400	0.18360000
H	3.52096500	4.23361400	4.94906200
H	1.90926300	4.82748300	5.32931300
H	-0.00558500	6.65180500	2.37560500
H	-1.57144500	5.95577900	1.97610400
C	2.12260600	1.77502700	5.57062400
C	0.86159900	0.22928200	4.16478700
O	0.07808500	0.96154600	2.00732900
C	0.00446300	5.52240600	-0.44822700
C	1.34779800	3.60346200	-1.15801000
O	1.95730700	1.95005100	0.51563400
H	2.70246800	1.96119800	6.46920600
C	1.56401200	0.51819900	5.34538500
P	0.96732400	0.68616800	0.69408200
H	-0.55581600	6.41520400	-0.70743700
C	0.66887600	4.80129700	-1.44107400
H	1.69004700	-0.27391600	6.07547300
O	1.98708800	-0.44347800	1.12677400
O	0.06425400	0.40702700	-0.45194000
H	0.65638900	5.15632700	-2.46568000
H	2.75969600	-0.49017900	0.51769700
C	-0.28221600	4.63105800	3.19145200

H	-1.13032500	3.94067300	3.22385100
H	-0.15703800	5.04018100	4.19629300
C	2.93409400	-4.47723200	3.68757500
C	1.58908200	-4.68489100	3.82899200
C	0.68098400	-3.58527300	3.91792500
C	1.20285400	-2.23945900	3.89862400
C	2.61633500	-2.07122700	3.75436300
C	3.45016600	-3.15109100	3.63831500
C	-0.70144100	-3.78384100	3.97529000
C	0.31231100	-1.14231200	3.97030700
C	-1.08583500	-1.35738300	3.94353300
C	-1.59574000	-2.71013400	3.94079800
C	-3.00832000	-2.92198700	3.88027800
H	-3.37532300	-3.94477800	3.88299100
C	-3.87919800	-1.86805800	3.81475200
C	-3.38177800	-0.53398900	3.83431400
C	-2.03684900	-0.28849000	3.91494800
H	-1.09154900	-4.79806000	4.00512200
H	3.61266400	-5.32086600	3.61028600
H	1.18316200	-5.69190700	3.86085700
H	3.01565300	-1.06672400	3.70098100
H	4.51556100	-2.99764100	3.49930500
H	-4.94712400	-2.04431500	3.73856700
H	-4.08004300	0.29593000	3.79383100
H	-1.67855500	0.73229900	3.94860200
C	-0.02468400	1.43127900	-5.67755100
C	1.31018700	1.17102000	-5.53047200
C	2.03435000	1.63966700	-4.39062600
C	1.33714300	2.37561500	-3.36113800
C	-0.06154000	2.60335100	-3.55119400
C	-0.71493400	2.16353100	-4.67199700
C	3.40348800	1.39855200	-4.25696700
C	2.04809600	2.84058700	-2.22863900
C	3.44474700	2.62618000	-2.13119800
C	4.12640800	1.88397100	-3.16544600
C	5.53424000	1.66960600	-3.06334700
H	6.02579600	1.09625400	-3.84295500
C	6.24682600	2.16552300	-2.00725700
C	5.58669300	2.92113900	-1.00008600
C	4.23749600	3.14519400	-1.06093400
H	3.92248300	0.83737700	-5.02941100
H	-0.55898900	1.08741600	-6.55733700
H	1.85383500	0.61511900	-6.28884600
H	-0.59790800	3.15272700	-2.78932200

H	-1.77377400	2.36795200	-4.79761800
H	7.31446400	1.98798400	-1.93618700
H	6.16397400	3.33218700	-0.17791200
H	3.76097200	3.74426700	-0.29563000
C	-0.19218500	-4.48270400	0.55878200
C	-1.63379800	-2.63684400	-0.00875400
C	-0.32421300	-2.94600000	0.65962100
H	0.85672400	-4.76505400	0.46281600
H	-0.56302800	-4.94986100	1.47355200
H	-2.42819300	-3.29411600	0.31861100
H	0.50264800	-2.42465500	0.17784100
H	-0.35581100	-2.60627100	1.69358500
N	-2.10409900	-1.37540700	-0.20461800
H	-1.42814000	-0.61738000	-0.40066700
C	-3.46030100	-1.06859700	-0.16260100
C	-4.37108400	-1.85695000	0.55504100
C	-3.93030400	0.08760700	-0.84810000
C	-5.72301000	-1.53817100	0.58542600
H	-4.03626300	-2.67910200	1.17056000
C	-5.29271200	0.38608800	-0.82509400
C	-6.18820300	-0.41711400	-0.12258600
H	-5.66219100	1.26316500	-1.33647200
H	-7.23637700	-0.14797000	-0.11438700
O	-6.50253100	-2.35116400	1.34878100
C	-7.89121300	-2.04990700	1.45101100
H	-8.05388200	-1.05759400	1.88752100
H	-8.38484000	-2.10416600	0.47373100
H	-8.31243800	-2.80846500	2.10993900
O	-2.98539100	0.80586600	-1.48158400
C	-3.34422400	2.04918500	-2.10966600
H	-2.40311100	2.36722300	-2.55342100
H	-4.03666600	1.82580200	-2.92535900
C	-3.90745700	3.11998500	-1.19704100
C	-3.41457600	3.30978700	0.10919700
C	-4.90733500	3.97368400	-1.71275800
C	-3.95534100	4.33961500	0.88831800
C	-5.41379600	4.99623300	-0.90545100
C	-4.94766900	5.17783800	0.39306300
H	-3.58382800	4.47802300	1.89973700
H	-6.18030800	5.65383600	-1.30415200
H	-5.35213200	5.97278000	1.01171800
C	-5.43984100	3.82584500	-3.12136300
H	-5.91886200	2.85264600	-3.28732000
H	-6.18878600	4.59297000	-3.32781300

H	-4.65158900	3.92754100	-3.87552600
C	-2.33432900	2.44847000	0.70891400
H	-1.54803700	2.20359400	-0.00336200
H	-1.87501100	2.95889400	1.55310100
H	-2.72824600	1.49920800	1.08773200
C	1.20983900	-4.18862600	-2.12021100
C	0.10877600	-3.30926300	-2.18775000
C	0.34399000	-1.98209100	-2.63562000
C	1.67213200	-1.53773900	-2.75930700
C	2.72130100	-2.41905100	-2.56332600
C	2.51238600	-3.77003700	-2.32459000
H	1.05181900	-5.23129200	-1.88447500
H	1.87478600	-0.50351900	-3.00558300
H	3.34692100	-4.45560000	-2.25522100
C	-1.20564500	-3.79825900	-1.64420500
C	-1.04284700	-4.95863100	-0.65397700
H	-0.61400600	-5.83539100	-1.14839000
H	-2.03290800	-5.28266500	-0.32561600
C	-2.39084500	-3.81059100	-2.52876200
C	-2.24982500	-3.63518300	-3.91974600
C	-3.68907800	-4.03109600	-2.02319500
C	-3.35499400	-3.66433300	-4.76279600
H	-1.25903300	-3.49604200	-4.33579400
C	-4.79501000	-4.04496600	-2.86416100
H	-3.84428000	-4.17717800	-0.96133800
C	-4.63450400	-3.85959300	-4.23901700
H	-3.21739600	-3.53945400	-5.83196300
H	-5.78383100	-4.20147200	-2.44565600
H	-5.49757300	-3.87508200	-4.89652600
C	-0.73757700	-1.00616500	-3.01172400
H	-0.51080200	-0.01418800	-2.62717700
H	-0.78251800	-0.93095900	-4.10376000
H	-1.72502800	-1.30081100	-2.66893800
O	4.06795300	-1.97939900	-2.65562500
S	4.78478600	-1.35012500	-1.33476600
O	6.15792600	-1.10292000	-1.70521500
O	3.93038400	-0.32649900	-0.72816300
C	4.77658000	-2.76964100	-0.09492800
F	3.54412900	-2.94335600	0.38569500
F	5.59330500	-2.42518900	0.89496700
F	5.20454600	-3.88798500	-0.67064700

SR-IV

Sum of electronic and thermal Free Energies = -4858.956759

C	-1.17609900	-4.18003900	-1.81832400
C	-2.46196900	-4.77651200	-2.47598500
C	-1.64936000	-4.01829200	-0.38199900
C	-0.61029700	-3.05048400	-2.65873200
H	-3.14187700	-3.95294800	-2.71693900
H	-2.24352800	-5.31440400	-3.40133700
C	-3.08203600	-5.66237900	-1.37608200
C	0.56672300	-5.02074800	-3.33419100
C	-2.65247600	-4.96306100	-0.10629500
C	-1.20013300	-3.20544000	0.64981300
C	0.29397500	-3.55759300	-3.60421400
C	-0.90837000	-1.69521300	-2.66162200
H	-4.16943800	-5.74448700	-1.46138800
H	-2.68304600	-6.68385200	-1.41384700
H	0.04654200	-5.65643200	-4.06124200
H	1.62968100	-5.27217600	-3.39892400
C	-3.12231000	-5.14575600	1.18922100
C	-1.59275300	-3.41861900	1.97956100
O	-0.35521600	-2.11102300	0.36546500
C	0.80768100	-2.72870300	-4.59747900
C	-0.42085200	-0.83057900	-3.65673500
O	-1.72352500	-1.19921200	-1.63832800
H	-3.90070400	-5.87301000	1.39801000
C	-2.55918600	-4.40204700	2.22780900
P	-1.03488700	-0.76723100	-0.23571500
H	1.48419900	-3.11940500	-5.35148300
C	0.42742600	-1.38547000	-4.63044600
H	-2.86664800	-4.57380000	3.25396200
O	-2.31458900	-0.52407600	0.65477200
O	-0.02965100	0.31290900	-0.33574700
H	0.79320800	-0.73869100	-5.42046500
H	-2.98137100	0.02728700	0.18192300
C	-0.00242100	-5.20973000	-1.91133500
H	0.75878600	-4.94302300	-1.16981500
H	-0.32884100	-6.23091500	-1.70321800
C	-2.74723100	0.28900500	5.44914000
C	-1.49368200	-0.15053200	5.77828800
C	-0.84286100	-1.16381400	5.00755400
C	-1.52749900	-1.72464700	3.86552800
C	-2.83612400	-1.23515200	3.55948600
C	-3.42506600	-0.26549800	4.32637700
C	0.43132800	-1.62655500	5.34334300
C	-0.90218900	-2.74191000	3.11595100
C	0.37092200	-3.23300600	3.49108000

C	1.05217800	-2.65029700	4.62201000
C	2.32913400	-3.16497700	5.00542300
H	2.83106600	-2.71396100	5.85665400
C	2.89411100	-4.21981600	4.33962400
C	2.21599400	-4.81168300	3.23669000
C	1.00288000	-4.32955800	2.82237900
H	0.94059700	-1.19979200	6.20343400
H	-3.23628700	1.04907200	6.05036800
H	-0.97464700	0.25085100	6.64400800
H	-3.35302800	-1.64496200	2.70185000
H	-4.42220700	0.08303600	4.08240500
H	3.85498900	-4.61449700	4.65455300
H	2.67096900	-5.64810700	2.71642300
H	0.49846000	-4.79230700	1.98323900
C	2.06828700	3.72414300	-3.79217000
C	0.74010500	4.02036000	-3.93535300
C	-0.25569500	2.99742400	-3.88395900
C	0.15087900	1.62130000	-3.71889500
C	1.54640700	1.36545400	-3.54034300
C	2.47135800	2.37835600	-3.57520000
C	-1.61371500	3.30630900	-3.97944100
C	-0.82771600	0.59893500	-3.73117600
C	-2.19788100	0.92383600	-3.88672100
C	-2.59392200	2.30912800	-3.97610200
C	-3.97732500	2.63527600	-4.10327700
H	-4.25926700	3.68289700	-4.14601000
C	-4.92744300	1.65371100	-4.18086700
C	-4.53880200	0.28766400	-4.14540000
C	-3.22307300	-0.06459400	-4.00267800
H	-1.91581300	4.34506000	-4.08565500
H	2.81421500	4.51122100	-3.82964900
H	0.41406900	5.04582100	-4.08351100
H	1.86785800	0.34772900	-3.37057900
H	3.52257200	2.15104500	-3.43748100
H	-5.97603200	1.91417200	-4.27147400
H	-5.29625100	-0.48449600	-4.23467900
H	-2.94950600	-1.11175200	-3.99286800
C	1.25008300	1.63861500	4.35915400
C	2.15027700	1.96601500	2.11602800
C	1.58229500	0.88372400	3.05949400
H	0.29398100	1.30515900	4.75967700
H	2.00151200	1.44782200	5.13005800
H	3.16640000	2.20993700	2.43026600
H	0.67788500	0.45918900	2.61292900

H	2.28834100	0.06444900	3.20041000
N	2.24390800	1.42486500	0.77201900
H	1.45636900	0.83808900	0.45378700
C	3.34222000	1.43177900	-0.00893900
C	4.36253000	2.39505200	0.07917400
C	3.49885300	0.33698200	-0.95279100
C	5.49272100	2.29772100	-0.71764300
H	4.25283700	3.25773500	0.72009300
C	4.65964200	0.24398100	-1.72930000
C	5.64797600	1.20697400	-1.62136200
H	4.79218900	-0.59517800	-2.39789500
H	6.53930300	1.11937300	-2.22850900
O	6.39955900	3.28520500	-0.58360700
C	7.57271300	3.27819700	-1.39875500
H	7.31671400	3.32147500	-2.46284400
H	8.12520300	4.17505000	-1.12256800
H	8.18983900	2.39564000	-1.19938100
O	2.51706900	-0.54834600	-0.92387200
C	2.61284100	-1.84057400	-1.56394100
H	1.62490400	-2.26138400	-1.37929900
H	2.71093100	-1.69027400	-2.63953800
C	3.70309300	-2.72120700	-1.00208300
C	3.88405400	-2.83009700	0.39338300
C	4.49042900	-3.48449100	-1.88965100
C	4.85664700	-3.71224100	0.87604500
C	5.45068800	-4.35561800	-1.36574400
C	5.63509600	-4.47164400	0.00851900
H	4.99330600	-3.80071500	1.94931800
H	6.05590900	-4.94683600	-2.04629600
H	6.38263800	-5.15370300	0.40126900
C	4.32394400	-3.39859800	-3.39084000
H	3.31398400	-3.66809300	-3.71245600
H	4.51619500	-2.38941500	-3.77728600
H	5.02294800	-4.07187600	-3.89097700
C	3.09496700	-2.01253400	1.38689500
H	2.03135400	-1.96418500	1.14584600
H	3.19694300	-2.43281400	2.38618500
H	3.46637000	-0.98445200	1.42496400
C	-1.25026000	2.71318500	2.62385100
C	-0.16193600	3.04697500	1.80170400
C	-0.40661400	3.25792100	0.42373000
C	-1.72520400	3.22832600	-0.05085800
C	-2.77067100	2.97026800	0.81742200
C	-2.55854100	2.66858200	2.15201400

H	-1.10075000	2.50109100	3.67270100
H	-1.92783400	3.42479900	-1.09847200
H	-3.38463400	2.43891300	2.81242500
C	1.24013900	3.22867700	2.41363100
C	1.23840300	3.13371700	3.97409800
H	0.41128500	3.70332700	4.40174000
H	2.15104300	3.61509000	4.33061400
C	1.85282300	4.59530100	2.06888500
C	1.05031600	5.68036100	1.69145400
C	3.21972200	4.84288700	2.27060600
C	1.59956000	6.94215900	1.46628000
H	-0.01603400	5.54057900	1.56499200
C	3.77568200	6.09971500	2.03747100
H	3.87027200	4.05908100	2.64498400
C	2.96744900	7.15726200	1.62287600
H	0.94992300	7.75990700	1.17020300
H	4.83929500	6.25114300	2.19244200
H	3.39534700	8.13768300	1.44134500
C	0.65831300	3.53399500	-0.60554600
H	0.74328400	2.67216900	-1.26882900
H	0.38207900	4.39440200	-1.21838800
H	1.63514700	3.74093800	-0.18160000
O	-4.10748300	3.15044600	0.36406700
S	-4.89240200	1.97655800	-0.43090800
O	-5.85131700	2.60877700	-1.30686400
O	-3.94826500	0.95594500	-0.89670300
C	-5.84279200	1.18705400	0.97285200
F	-4.97414800	0.52065400	1.74615600
F	-6.72390600	0.33937800	0.45936500
F	-6.45545900	2.11724500	1.69006600

(6) Control Experiments

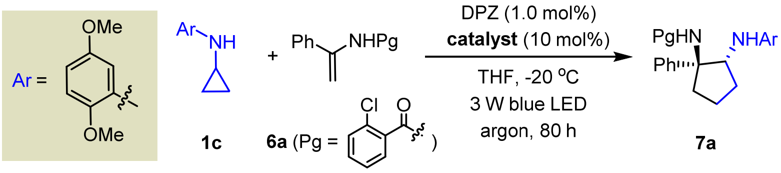

C3 as the catalyst, with Na_2HPO_4: **7a**: 77% yield, 95% ee (0.1 mmol scale)
C3 as the catalyst, no Na_2HPO_4: **7a**: 37% yeld, 93% ee (0.1 mmol scale)
C3Na as the catalyst, no Na_2HPO_4: **7a**: 68% yeld, 0% ee (0.1 mmol scale)

As the above equation shown, the reaction of **1c** with **6a** could afford product **7a** in 77% yield with 95% ee (also see Table 3 in the manuscript). We then attempted it under the established reaction conditions but without Na_2HPO_4. It was found that **7a** was obtained in 37% yield with 93% ee. The reaction was also evaluated with sodium salt of CPA **C3** (i.e., **C3Na**), leading to 7a in

68% yield with 0% ee. The results indicate that Na_2HPO_4 can promote the transformation but roughly does not affect the enantiocontrol effect of catalyst. Furthermore, the generation of **C3Na** when **C3** in the presence of Na_2HPO_4 might be excluded.

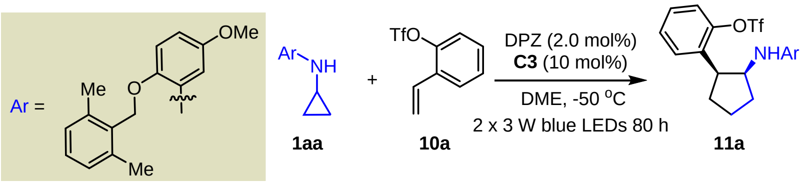

With KCl (0.55 equiv), 18-Crown-6 (0.2 equiv): **11a**: 84% yield, 80% ee, >19:1 dr
No KCl (0.55 equiv), 18-Crown-6 (0.2 equiv): **11a**: 62% yield, 80% ee, >19:1 dr

As the above equation shown,, the reaction of **1aa** with **10a** could afford product **11a** in 84% yield with 80% ee and >19:1 dr (also see Table 4 in the manuscript). We then attempted it under the established reaction conditions but without additives (i.e., KCl and 18-Crown-6). It was found that **11a** was obtained in 62% yield with 80% ee and >19:1 dr. The results indicate that the additives are useful to the acceleration of chemical transformations.

5. Synthetic applications

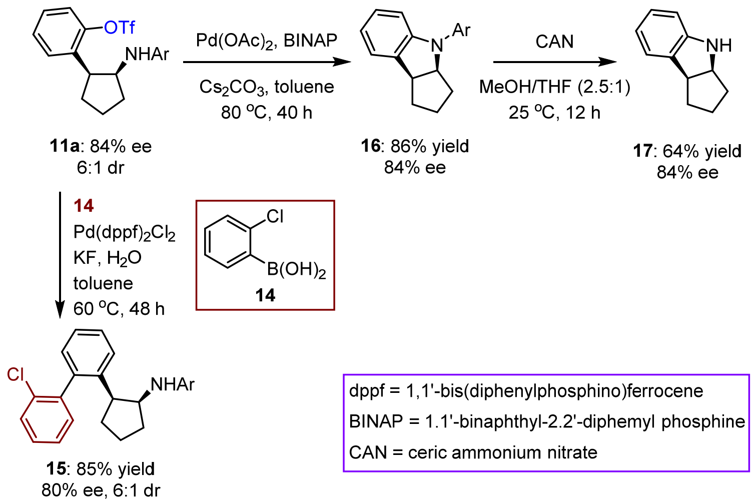

15: To a mixture of **11a** (61.1 mg, 0.1 mmol, 1.0 equiv.), KF (17.4 mg, 0.3 mmol, 3.0 equiv.), **14** (17.2 mg, 0.11 mmol, 1.1 equiv.), Pd(dppf)$_2$Cl$_2$ (7.31 mg, 0.01 mmol, 10 mol %) was added 3.0 mL toluene at room temperature under argon atmosphere. H_2O (0.3 mL) was added subsequently. The reaction mixture was warmed to 60 °C and stirred for 48 h. The reaction was quenched with sat. NaCl water solution and extracted with EA (3 × 4 mL). The combined organic phase was washed with brine, dried over Na_2SO_4, evaporated to give the crude products. The residue was purified by flash chromatography (*silica gel*, 30:1 hexanes/EtOAc) to give product **15.**

16: To a mixture of **11a** (54.9 mg, 0.1 mmol, 1.0 equiv.), Cs_2CO_3 (97.7mg, 0.3 mmol, 3.0 equiv.), BINAP (12.4 mg, 0.02 mmol, 20 mol%) and Pd(OAc)$_2$ (2.24 mg, 0.01 mmol, 10 mol%) was added 3.0 mL toluene at room temperature under argon atmosphere. The reaction mixture was warmed to 80 °C and stirred for 40 h. The reaction was quenched with sat. NaCl water solution and extracted with EA (3 × 4 mL). The combined organic phase was washed with brine, dried over Na_2SO_4, evaporated to give the crude products. The residue was purified by flash chromatography (*silica gel*, 30:1 hexanes/EtOAc) to give product **16**.

17: To a solution of CAN (438.4 mg, 0.8 mmol, 2.0 equiv.) in MeOH (1.0 mL) under an atmosphere of N_2 at 0 °C was added a solution of **16** (159.6 mg, 0.4 mmol, 1.0 equiv.) in MeOH/THF (v/v = 2:1, 4 mL) through a pressure-equalizing addition funnel over 30 min. The mixture was allowed to stir at 0 °C for an additional 30 min upon until 1.0 M HCl (2 mL) was

added. The resulting solution was allowed to warm to 25 °C and stirred for 12 h. After removal of solvent by evaporation, the resulting mixture was washed with 5.0 mL CH_2Cl_2 for three times. The combined organic portion was then washed with 1.0 M aqueous HCl (3 x 5 mL). The combined aqueous layers were neutralized with solid Na_2CO_3 until pH ≈ 7. The aqueous layer was washed with CH_2Cl_2 (4 x 5 mL). The combined organic phase was washed with brine, dried over Na_2SO_4, evaporated to give the crude product. The residue was purified by flash chromatography (*silica gel*, 10:1 hexanes/EtOAc) to give product **17**.

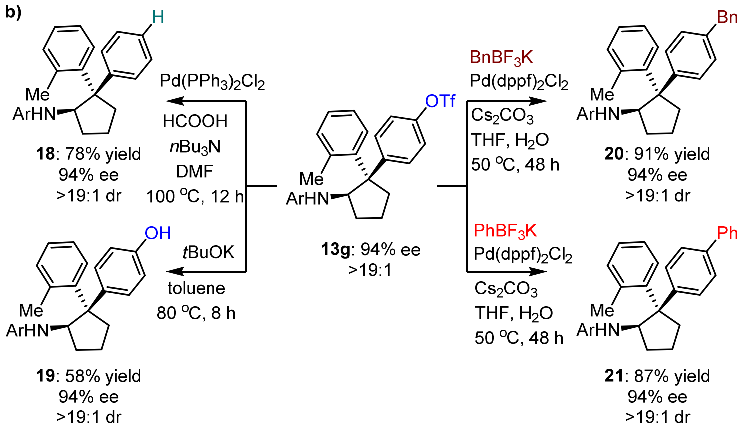

18: To a solution of **13g** (61.1 mg, 0.1 mmol, 1.0 equiv.) in DMF (3 mL) and formic acid (7.7 μL, 0.4 mmol, 4.0 equiv.) were added $Pd(PPh_3)_2Cl_2$ (3.5 mg, 0.005 mmol, 5 mol%) and tributylamine (142.9 μL, 0.6 mmol, 6.0 equiv.) at room temperature. After this mixture was stirred for 12 h at 100 °C under argon, the reaction was quenched with water (3 mL) and the resulting mixture was extracted with Et_2O (3 mL) for three times. The combined organic layers were washed with brine (3 mL), dried with Na_2SO_4 and concentrated. The resulting residue was purified by flash column chromatography (hexane/AcOEt, 30:1) on *silica gel* to give product **18**.

19: To a solution of **13g** (61.1 mg, 0.1 mmol, 1.0 equiv.) in toluene (3 mL) was added *t*-BuOK (33.6 mg, 0.3 mmol, 3.0 equiv.). The suspension was heated at 80 °C (oil bath temp) during 8 h. The crude mixture was quenched with ethyl acetate (3 mL) and a saturated aqueous solution of ammonium chloride (3 mL). The aqueous layer was extracted with ethyl acetate (2 × 10 mL). Organic layers were then combined, dried over $MgSO_4$, evaporated to give the crude product. The residue was purified by flash chromatography (*silica gel*, 5:1 hexanes/EtOAc) to afford product **19**.

20: To a mixture of **13g** (61.1 mg, 0.1 mmol, 1.0 equiv.), Cs_2CO_3 (97.7 mg, 0.3 mmol, 3.0 equiv.), $BnBF_3K$ (19.8 mg, 0.1 mmol, 1.0 equiv.), $Pd(dppf)_2Cl_2$ (7.31 mg, 0.01 mmol, 10 mol %) was added 3.0 mL THF at room temperature under argon atmosphere. H_2O (0.3 mL) was added subsequently. The reaction mixture was warmed to 50 ^oC and stirred 48 h. The reaction was quenched with sat. NaCl water solution and extracted with EA (3 × 4 mL). The combined organic phase was washed with brine, dried over Na_2SO_4, evaporated to give the crude products. The residue was purified by flash chromatography (*silica gel*, 30:1 hexanes/EtOAc) to give **20**.

21: To a mixture of **13g** (61.1 mg, 0.1 mmol, 1.0 equiv.), Cs_2CO_3 (97.7 mg, 0.3 mmol, 3.0 equiv.), $PhBF_3K$ (18.4 mg, 0.1 mmol, 1.0 equiv.), $Pd(dppf)_2Cl_2$ (7.31 mg, 0.01 mmol, 10 mol %) was added 3.0 mL THF at room temperature under argon atmosphere. H_2O (0.3 mL) was added subsequently. The reaction mixture was warmed to 50 ^oC and stirred 48 h. The reaction was quenched with sat. NaCl water solution and extracted with EA (3 × 4 mL). The combined organic phase was washed with brine, dried over Na_2SO_4, evaporated to give the crude products. The residue was purified by flash chromatography (*silica gel*, 30:1 hexanes/EtOAc) to give **21**.

6. Determination of the absolute configurations

(1) Absolute configurations of **3** and **5** are determined by *X*-ray structure analysis of the product **3f.**

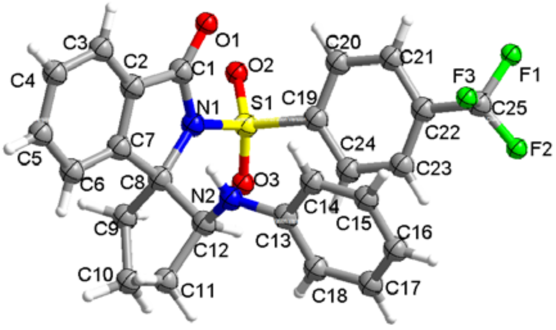

Fig. S18. Absolute configuration of **3f** (CCDC 2080352).

Displacement ellipsoids are drawn at the 30% probability level.

(Solvent: ethyl acetate/hexane = 1:10*)*

Table S10 Crystal data and structure refinement for 3f.

Identification code	**3f**
Empirical formula	$C_{25}H_{21}F_3N_2O_3S$
Formula weight	486.50
Temperature/K	293(2)
Crystal system	monoclinic
Space group	$P2_1/c$
a/Å	15.2479(2)
b/Å	19.58181(19)
c/Å	16.7407(3)
α/°	90
β/°	116.0951(19)
γ/°	90
Volume/Å^3	4488.94(12)
Z	8
ρ_{calc}g/cm^3	1.440
μ/mm^{-1}	1.779
F(000)	2016.0
Crystal size/mm^3	0.15 × 0.12 × 0.1
Radiation	CuKα (λ = 1.54184)
2Θ range for data collection/°	7.414 to 134.158
Index ranges	$-13 \le h \le 18, -23 \le k \le 23, -19 \le l \le 20$
Reflections collected	36582
Independent reflections	8004 [R_{int} = 0.0313, R_{sigma} = 0.0211]

Data/restraints/parameters	8004/0/613
Goodness-of-fit on F^2	1.039
Final R indexes [I>=2σ (I)]	R_1 = 0.0474, wR_2 = 0.1294
Final R indexes [all data]	R_1 = 0.0557, wR_2 = 0.1377
Largest diff. peak/hole / e $Å^{-3}$	0.34/-0.31

Table S11 Fractional Atomic Coordinates (×10^4) and Equivalent Isotropic Displacement Parameters (Å^2×10^3) for 3f. U_{eq} is defined as 1/3 of of the trace of the orthogonalised U_{IJ} tensor.

Atom	*x*	*y*	*z*	U(eq)
C1	491.7(15)	6955.4(10)	2739.4(14)	44.9(4)
C2	750.3(15)	7142.7(10)	3665.3(14)	44.9(4)
C3	1505.0(18)	7559.7(11)	4223.2(17)	56.2(5)
C4	1580(2)	7692.5(12)	5057.3(18)	65.6(6)
C5	914(2)	7414.1(13)	5319.6(18)	68.5(7)
C6	161(2)	7000.1(12)	4761.1(17)	61.5(6)
C7	83.2(16)	6856.7(10)	3920.8(15)	47.2(5)
C8	-659.1(15)	6423.7(10)	3189.0(14)	46.1(4)
C9	-1714.8(17)	6590.2(13)	3017.3(18)	61.7(6)
C10	-1982(2)	6049.7(17)	3532(2)	80.4(8)
C11	-1121(2)	5571.6(13)	3967.3(18)	64.5(6)
C12	-547.6(15)	5652.0(10)	3416.6(15)	48.2(5)
C13	764.4(17)	4777.3(11)	3767.5(14)	49.9(5)
C14	1746.9(19)	4661.2(13)	4005.3(17)	63.4(6)
C15	2091(3)	4010.5(16)	4002(2)	86.6(9)
C16	1467(3)	3465.2(15)	3749(2)	91.9(10)
C17	494(3)	3572.4(13)	3502(2)	79.0(8)
C18	136(2)	4219.5(12)	3512.0(16)	61.9(6)
C19	-178.1(15)	5620.1(11)	1349.1(13)	46.7(4)
C20	589.0(19)	5776.7(13)	1156(2)	66.3(7)
C21	1187.3(19)	5260.1(14)	1134(2)	74.8(8)
C22	1020.6(18)	4593.6(13)	1303.7(16)	60.3(6)
C23	233(2)	4439.8(13)	1459.4(18)	66.6(6)
C24	-378.2(19)	4952.8(12)	1474.2(16)	59.6(6)
C25	1724(2)	4045.5(18)	1345(2)	84.3(9)
F1	2089.7(17)	4150.6(12)	760.2(16)	115.7(7)
F2	1341.5(18)	3440.2(10)	1161.4(18)	124.2(8)
F3	2488.3(17)	4029.6(16)	2121.0(16)	156.4(13)
N1	-385.7(12)	6584.3(9)	2447.4(11)	45.6(4)
N2	454.7(14)	5441.6(9)	3794.3(14)	58.5(5)
O1	923.3(11)	7084.0(8)	2302.3(10)	55.4(4)

O2	-942.4(13)	6823.6(9)	840.0(11)	64.0(4)
O3	-1828.6(11)	5985.7(9)	1312.0(12)	64.3(4)
S1	-936.4(4)	6283.4(3)	1406.9(3)	48.65(15)
C1'	5446.1(15)	6856.6(10)	2357.4(15)	46.7(5)
C2'	5685.1(15)	7135.7(10)	3241.7(15)	48.4(5)
C3'	6427.4(18)	7588.7(12)	3731.0(18)	62.7(6)
C4'	6502(2)	7803.6(14)	4541.8(19)	72.1(7)
C5'	5843(2)	7571.2(13)	4843.0(18)	68.5(7)
C6'	5104.1(19)	7119.8(12)	4352.3(16)	59.4(6)
C7'	5032.4(15)	6892.6(10)	3541.6(14)	45.3(4)
C8'	4299.0(14)	6409.7(10)	2870.2(13)	42.7(4)
C9'	3235.5(15)	6605.0(11)	2643.0(16)	52.9(5)
C10'	3011.5(19)	6231.5(15)	3339.2(18)	67.9(7)
C11'	3829.8(17)	5709.2(13)	3802.9(16)	57.8(5)
C12'	4373.9(14)	5670.5(10)	3219.7(14)	44.8(4)
C13'	5579.4(16)	4725.5(10)	3743.4(13)	47.0(5)
C14'	4864.6(18)	4226.7(11)	3553.7(16)	56.7(5)
C15'	5125(2)	3543.0(12)	3668.6(18)	70.6(7)
C16'	6073(3)	3345.8(13)	3980(2)	82.3(9)
C17'	6785(2)	3832.9(14)	4167(2)	76.8(8)
C18'	6544.2(18)	4520.7(12)	4046.0(16)	59.5(6)
C19'	4929.3(16)	5394.3(11)	1249.0(14)	48.3(5)
C20'	5712.0(19)	5532.1(12)	1064.5(19)	63.2(6)
C21'	6386.9(19)	5025.0(13)	1196(2)	67.3(7)
C22'	6281.7(16)	4392.2(12)	1508.9(16)	55.7(5)
C23'	5472.7(18)	4252.7(12)	1652.2(17)	58.5(6)
C24'	4794.2(17)	4753.8(11)	1520.3(15)	53.7(5)
C25'	7064(2)	3862.0(15)	1737(2)	71.1(7)
F1'	7639.9(16)	3854.2(16)	2604.3(15)	139.4(10)
F2'	7648.1(14)	3975.2(9)	1351.1(17)	105.7(7)
F3'	6725.0(13)	3241.1(8)	1502.0(14)	89.1(5)
N1'	4588.9(12)	6470.7(8)	2121.5(11)	44.2(4)
N2'	5357.9(13)	5418.6(9)	3617.5(13)	51.7(4)
O1'	5878.4(12)	6935.2(8)	1908.1(12)	59.2(4)
O2'	4057.9(14)	6522.9(9)	486.4(11)	67.7(5)
O3'	3223.4(11)	5747.5(9)	1093.3(11)	61.1(4)
S1'	4093.8(4)	6055.7(3)	1149.7(3)	49.46(15)

Table S12 Anisotropic Displacement Parameters (Å^2×10^3) for 3f. The Anisotropic displacement factor exponent takes the form: -2π^2[h^{2}a*^{2}U$_{11}$+2hka*b*U$_{12}$+…].

Atom	U_{11}	U_{22}	U_{33}	U_{23}	U_{13}	U_{12}
C1	47.3(11)	38.8(9)	52.7(11)	8.5(8)	25.8(9)	3.7(8)
C2	50.3(11)	36.9(9)	51.7(11)	3.2(8)	26.4(9)	2.6(8)
C3	61.4(13)	42.7(11)	67.9(14)	-3.3(10)	31.5(11)	-6.1(9)
C4	76.1(16)	51.9(13)	66.8(15)	-16.7(11)	29.5(13)	-8.6(11)
C5	93.1(19)	57.8(14)	61.9(15)	-16.4(12)	40.9(14)	-1.9(13)
C6	80.2(16)	56.5(13)	64.9(14)	-9.1(11)	47.6(13)	-5.3(12)
C7	54.5(12)	38.6(10)	56.1(12)	0.1(9)	31.2(10)	3.9(8)
C8	49.5(11)	46.4(10)	51.2(11)	2.4(9)	30.3(9)	0.4(8)
C9	53.0(13)	68.1(14)	75.9(16)	1.7(12)	39.4(12)	3.7(11)
C10	68.4(17)	96(2)	100(2)	11.7(17)	57.7(17)	-2.3(15)
C11	76.7(16)	64.7(14)	64.3(15)	2.1(12)	42.1(13)	-15.6(12)
C12	49.6(11)	46.0(11)	51.5(11)	0.4(9)	24.5(9)	-7.6(9)
C13	61.6(13)	42.1(10)	45.6(11)	5.1(8)	23.1(10)	-2.6(9)
C14	65.9(15)	53.4(13)	68.8(15)	4.1(11)	27.8(12)	-0.3(11)
C15	88(2)	70.8(18)	104(2)	10.9(17)	44.9(19)	20.4(16)
C16	128(3)	51.9(15)	105(2)	7.1(15)	59(2)	17.3(17)
C17	121(3)	44.2(13)	75.9(18)	-3.6(12)	47.4(18)	-16.7(15)
C18	77.0(16)	49.6(12)	59.8(14)	-0.3(10)	30.6(12)	-11.7(11)
C19	47.7(11)	49.4(11)	42.7(10)	-3.7(8)	19.8(9)	-3.3(9)
C20	67.7(15)	53.7(13)	92.3(19)	-12.6(12)	48.8(14)	-14.6(11)
C21	57.1(14)	71.7(16)	107(2)	-29.8(16)	46.6(15)	-15.7(12)
C22	57.1(13)	61.4(14)	52.9(13)	-18.1(11)	15.5(10)	1.1(11)
C23	89.0(18)	52.9(13)	66.2(15)	-0.3(11)	41.7(14)	0.4(12)
C24	72.3(15)	53.5(12)	65.6(14)	-4.4(11)	41.7(12)	-8.5(11)
C25	74.8(18)	93(2)	72.5(18)	-25.3(16)	20.9(15)	19.0(16)
F1	117.8(16)	116.9(16)	133.0(18)	-30.8(14)	73.9(15)	21.0(13)
F2	130.3(18)	70.3(12)	176(2)	-21.0(13)	71.4(17)	17.7(12)
F3	109.8(17)	203(3)	97.5(16)	-41.5(17)	-8.0(13)	83.7(18)
N1	45.3(9)	48.9(9)	46.7(9)	3.0(7)	24.1(7)	-1.0(7)
N2	54.2(11)	40.9(9)	73.4(13)	-2.6(9)	21.8(9)	-6.7(8)
O1	58.1(9)	61.6(9)	55.8(9)	8.3(7)	33.6(7)	-5.7(7)
O2	75.0(11)	61.1(10)	49.9(9)	12.3(7)	22.0(8)	8.0(8)
O3	44.3(8)	78.2(11)	65.8(10)	-4.8(8)	20.0(7)	-4.1(7)
S1	44.9(3)	52.9(3)	45.6(3)	3.3(2)	17.4(2)	1.3(2)
C1'	49.2(11)	36.8(9)	58.6(12)	5.0(9)	27.8(10)	2.7(8)
C2'	47.7(11)	38.7(10)	59.0(12)	-1.6(9)	23.6(10)	0.8(8)
C3'	55.7(13)	52.6(13)	78.5(17)	-8.6(12)	28.4(12)	-9.8(10)
C4'	66.8(15)	59.4(14)	75.8(17)	-17.5(13)	18.3(13)	-11.9(12)
C5'	81.2(17)	61.1(14)	56.1(14)	-13.7(11)	23.7(13)	-3.1(13)

C6'	68.9(15)	56.2(13)	57.1(13)	-4.1(10)	31.4(12)	-1.8(11)
C7'	47.3(11)	37.0(9)	50.6(11)	0.9(8)	20.6(9)	3.7(8)
C8'	45.6(10)	40.5(10)	44.8(10)	0.6(8)	22.4(9)	1.0(8)
C9'	47.6(11)	51.0(11)	61.1(13)	1.3(10)	24.9(10)	7.0(9)
C10'	55.9(14)	90.4(19)	66.2(15)	6.5(13)	34.8(12)	8.2(13)
C11'	59.5(13)	61.8(13)	61.1(14)	6.2(11)	34.8(11)	-4.0(11)
C12'	44.1(10)	40.9(10)	50.0(11)	1.9(8)	21.2(9)	-2.3(8)
C13'	54.7(12)	40.8(10)	44.4(11)	4.5(8)	20.6(9)	0.2(9)
C14'	63.9(14)	47.4(11)	55.1(13)	3.5(10)	22.7(11)	-5.8(10)
C15'	96(2)	44.4(12)	67.5(16)	0.2(11)	32.2(15)	-12.0(13)
C16'	112(2)	42.9(13)	82.7(19)	9.4(13)	34.4(18)	14.7(14)
C17'	82.2(19)	58.0(15)	82.6(19)	10.6(13)	29.3(15)	20.6(14)
C18'	60.3(13)	52.2(12)	62.5(14)	7.0(10)	23.8(11)	5.2(10)
C19'	54.2(12)	46.3(11)	48.3(11)	-8.2(9)	26.1(10)	-4.6(9)
C20'	76.1(16)	46.6(12)	85.3(17)	-7.8(11)	52.3(14)	-9.7(11)
C21'	64.5(15)	57.7(13)	99(2)	-21.7(13)	53.1(15)	-16.0(11)
C22'	51.3(12)	52.4(12)	62.1(13)	-16.3(10)	23.9(10)	-6.4(10)
C23'	64.2(14)	47.5(11)	68.8(15)	-2.1(10)	33.9(12)	-3.0(10)
C24'	56.4(12)	52.2(12)	60.5(13)	-4.0(10)	33.0(11)	-5.5(10)
C25'	56.6(14)	72.1(16)	79.7(18)	-18.9(14)	25.4(13)	-2.2(12)
F1'	92.5(14)	193(3)	93.0(15)	-30.0(15)	4.2(11)	56.7(15)
F2'	89.0(12)	74.6(11)	186(2)	-16.9(12)	89.9(15)	1.7(9)
F3'	87.0(11)	54.7(8)	129.1(15)	3.9(9)	50.6(11)	8.8(8)
N1'	50.3(9)	39.3(8)	46.1(9)	0.7(7)	24.2(8)	-0.3(7)
N2'	45.1(9)	38.2(8)	67.2(11)	4.0(8)	20.4(8)	-2.7(7)
O1'	66.0(10)	54.0(9)	71.8(10)	1.0(8)	43.3(9)	-5.5(7)
O2'	85.2(12)	67.1(10)	49.3(9)	8.9(8)	28.2(8)	8.1(9)
O3'	48.4(8)	68.6(10)	61.9(10)	-11.1(8)	20.3(7)	-4.3(7)
S1'	51.8(3)	49.9(3)	45.5(3)	-0.4(2)	20.3(2)	1.5(2)

Table S13 Bond Lengths for 3f.

Atom	Atom	Length/Å	Atom	Atom	Length/Å
C1	C2	1.468(3)	C1'	C2'	1.466(3)
C1	N1	1.408(3)	C1'	N1'	1.408(3)
C1	O1	1.206(2)	C1'	O1'	1.208(3)
C2	C3	1.385(3)	C2'	C3'	1.387(3)
C2	C7	1.383(3)	C2'	C7'	1.381(3)
C3	C4	1.375(4)	C3'	C4'	1.377(4)
C4	C5	1.383(4)	C4'	C5'	1.385(4)
C5	C6	1.380(4)	C5'	C6'	1.383(4)

C6	C7	1.387(3)	C6'	C7'	1.386(3)
C7	C8	1.510(3)	C7'	C8'	1.517(3)
C8	C9	1.540(3)	C8'	C9'	1.542(3)
C8	C12	1.549(3)	C8'	C12'	1.547(3)
C8	N1	1.506(2)	C8'	N1'	1.506(2)
C9	C10	1.529(4)	C9'	C10'	1.536(3)
C10	C11	1.513(4)	C10'	C11'	1.534(4)
C11	C12	1.532(3)	C11'	C12'	1.536(3)
C12	N2	1.434(3)	C12'	N2'	1.435(3)
C13	C14	1.389(3)	C13'	C14'	1.392(3)
C13	C18	1.391(3)	C13'	C18'	1.388(3)
C13	N2	1.391(3)	C13'	N2'	1.392(3)
C14	C15	1.379(4)	C14'	C15'	1.385(3)
C15	C16	1.368(5)	C15'	C16'	1.359(4)
C16	C17	1.369(5)	C16'	C17'	1.374(4)
C17	C18	1.383(4)	C17'	C18'	1.387(3)
C19	C20	1.378(3)	C19'	C20'	1.385(3)
C19	C24	1.379(3)	C19'	C24'	1.380(3)
C19	S1	1.770(2)	C19'	S1'	1.772(2)
C20	C21	1.373(4)	C20'	C21'	1.376(4)
C21	C22	1.383(4)	C21'	C22'	1.382(4)
C22	C23	1.369(4)	C22'	C23'	1.383(3)
C22	C25	1.497(4)	C22'	C25'	1.499(4)
C23	C24	1.378(4)	C23'	C24'	1.372(3)
C25	F1	1.339(4)	C25'	F1'	1.325(4)
C25	F2	1.297(4)	C25'	F2'	1.329(3)
C25	F3	1.309(4)	C25'	F3'	1.312(3)
N1	S1	1.6733(18)	N1'	S1'	1.6723(17)
O2	S1	1.4184(17)	O2'	S1'	1.4211(17)
O3	S1	1.4233(17)	O3'	S1'	1.4229(17)

Table S14 Bond Angles for 3f.

Atom	Atom	Atom	Angle/°	Atom	Atom	Atom	Angle/°
N1	C1	C2	105.57(16)	N1'	C1'	C2'	105.73(17)
O1	C1	C2	128.6(2)	O1'	C1'	C2'	128.3(2)
O1	C1	N1	125.9(2)	O1'	C1'	N1'	126.0(2)
C3	C2	C1	128.09(19)	C3'	C2'	C1'	127.6(2)
C7	C2	C1	109.44(18)	C7'	C2'	C1'	109.87(18)
C7	C2	C3	122.4(2)	C7'	C2'	C3'	122.5(2)
C4	C3	C2	118.0(2)	C4'	C3'	C2'	118.0(2)

C3	C4	C5	120.2(2)	C3'	C4'	C5'	120.0(2)
C6	C5	C4	121.6(2)	C6'	C5'	C4'	121.7(2)
C5	C6	C7	118.7(2)	C5'	C6'	C7'	118.7(2)
C2	C7	C6	119.0(2)	C2'	C7'	C6'	119.1(2)
C2	C7	C8	111.54(18)	C2'	C7'	C8'	111.07(18)
C6	C7	C8	129.5(2)	C6'	C7'	C8'	129.8(2)
C7	C8	C9	112.41(18)	C7'	C8'	C9'	112.38(17)
C7	C8	C12	112.71(17)	C7'	C8'	C12'	113.29(16)
C9	C8	C12	104.19(17)	C9'	C8'	C12'	102.71(16)
N1	C8	C7	99.84(16)	N1'	C8'	C7'	100.17(15)
N1	C8	C9	116.57(18)	N1'	C8'	C9'	115.90(17)
N1	C8	C12	111.48(16)	N1'	C8'	C12'	112.90(15)
C10	C9	C8	105.3(2)	C10'	C9'	C8'	104.80(18)
C11	C10	C9	108.0(2)	C11'	C10'	C9'	107.37(18)
C10	C11	C12	104.5(2)	C10'	C11'	C12'	104.93(18)
C11	C12	C8	102.61(17)	C11'	C12'	C8'	102.92(16)
N2	C12	C8	111.67(16)	N2'	C12'	C8'	112.67(16)
N2	C12	C11	119.0(2)	N2'	C12'	C11'	117.96(18)
C14	C13	C18	118.0(2)	C18'	C13'	C14'	118.6(2)
C14	C13	N2	118.8(2)	C18'	C13'	N2'	119.0(2)
C18	C13	N2	123.1(2)	N2'	C13'	C14'	122.4(2)
C15	C14	C13	121.0(3)	C15'	C14'	C13'	119.9(2)
C16	C15	C14	120.6(3)	C16'	C15'	C14'	121.3(3)
C15	C16	C17	119.2(3)	C15'	C16'	C17'	119.4(2)
C16	C17	C18	121.2(3)	C16'	C17'	C18'	120.6(3)
C17	C18	C13	120.1(3)	C17'	C18'	C13'	120.2(2)
C20	C19	C24	120.9(2)	C20'	C19'	S1'	119.42(17)
C20	C19	S1	119.47(17)	C24'	C19'	C20'	121.2(2)
C24	C19	S1	119.58(17)	C24'	C19'	S1'	119.33(17)
C21	C20	C19	119.0(2)	C21'	C20'	C19'	118.8(2)
C20	C21	C22	120.4(2)	C20'	C21'	C22'	120.3(2)
C21	C22	C25	119.6(3)	C21'	C22'	C23'	120.3(2)
C23	C22	C21	120.2(2)	C21'	C22'	C25'	120.4(2)
C23	C22	C25	120.2(3)	C23'	C22'	C25'	119.3(2)
C22	C23	C24	120.0(2)	C24'	C23'	C22'	119.9(2)
C23	C24	C19	119.4(2)	C23'	C24'	C19'	119.5(2)
F1	C25	C22	112.1(3)	F1'	C25'	C22'	110.9(2)
F2	C25	C22	113.9(3)	F1'	C25'	F2'	105.8(3)
F2	C25	F1	104.8(2)	F2'	C25'	C22'	112.9(3)
F2	C25	F3	109.0(3)	F3'	C25'	C22'	113.7(2)

F3	C25	C22	111.7(2)	F3'	C25'	F1'	107.6(3)
F3	C25	F1	104.7(3)	F3'	C25'	F2'	105.5(2)
C1	N1	C8	112.77(16)	C1'	N1'	C8'	112.67(16)
C1	N1	S1	120.55(14)	C1'	N1'	S1'	119.99(14)
C8	N1	S1	126.18(14)	C8'	N1'	S1'	126.80(13)
C13	N2	C12	124.45(19)	C13'	N2'	C12'	122.68(17)
N1	S1	C19	105.94(9)	N1'	S1'	C19'	105.40(9)
O2	S1	C19	108.83(10)	O2'	S1'	C19'	108.83(11)
O2	S1	N1	106.65(10)	O2'	S1'	N1'	107.05(10)
O2	S1	O3	120.41(11)	O2'	S1'	O3'	120.46(11)
O3	S1	C19	107.99(10)	O3'	S1'	C19'	107.90(10)
O3	S1	N1	106.12(9)	O3'	S1'	N1'	106.22(9)

Table S15 Torsion Angles for 3f.

A	B	C	D	Angle/°	A	B	C	D	Angle/°
C1	C2	C3	C4	176.7(2)	C1'	C2'	C3'	C4'	177.9(2)
C1	C2	C7	C6	-176.6(2)	C1'	C2'	C7'	C6'	-177.16(19)
C1	C2	C7	C8	2.5(2)	C1'	C2'	C7'	C8'	1.0(2)
C1	N1	S1	C19	69.81(17)	C1'	N1'	S1'	C19'	68.42(16)
C1	N1	S1	O2	-46.02(18)	C1'	N1'	S1'	O2'	-47.34(18)
C1	N1	S1	O3	-175.55(15)	C1'	N1'	S1'	O3'	-177.25(15)
C2	C1	N1	C8	-8.4(2)	C2'	C1'	N1'	C8'	-6.8(2)
C2	C1	N1	S1	179.23(13)	C2'	C1'	N1'	S1'	-178.93(13)
C2	C3	C4	C5	-0.1(4)	C2'	C3'	C4'	C5'	-0.5(4)
C2	C7	C8	C9	-131.22(19)	C2'	C7'	C8'	C9'	-128.36(19)
C2	C7	C8	C12	111.40(19)	C2'	C7'	C8'	C12'	115.77(19)
C2	C7	C8	N1	-7.0(2)	C2'	C7'	C8'	N1'	-4.7(2)
C3	C2	C7	C6	1.0(3)	C3'	C2'	C7'	C6'	1.5(3)
C3	C2	C7	C8	-179.83(19)	C3'	C2'	C7'	C8'	179.7(2)
C3	C4	C5	C6	-0.1(4)	C3'	C4'	C5'	C6'	0.5(4)
C4	C5	C6	C7	0.7(4)	C4'	C5'	C6'	C7'	0.5(4)
C5	C6	C7	C2	-1.2(3)	C5'	C6'	C7'	C2'	-1.5(3)
C5	C6	C7	C8	179.9(2)	C5'	C6'	C7'	C8'	-179.3(2)
C6	C7	C8	C9	47.8(3)	C6'	C7'	C8'	C9'	49.6(3)
C6	C7	C8	C12	-69.6(3)	C6'	C7'	C8'	C12'	-66.3(3)
C6	C7	C8	N1	172.0(2)	C6'	C7'	C8'	N1'	173.2(2)
C7	C2	C3	C4	-0.4(3)	C7'	C2'	C3'	C4'	-0.5(4)
C7	C8	C9	C10	-97.8(2)	C7'	C8'	C9'	C10'	-88.4(2)
C7	C8	C12	C11	84.1(2)	C7'	C8'	C12'	C11'	79.6(2)
C7	C8	C12	N2	-44.4(2)	C7'	C8'	C12'	N2'	-48.5(2)

C7	C8	N1	C1	9.4(2)	C7'	C8'	N1'	C1'	7.1(2)
C7	C8	N1	S1	-178.74(14)	C7'	C8'	N1'	S1'	178.57(13)
C8	C9	C10	C11	-1.6(3)	C8'	C9'	C10'	C11'	-12.9(3)
C8	C12	N2	C13	-155.5(2)	C8'	C12'	N2'	C13'	-160.99(19)
C8	N1	S1	C19	-101.45(17)	C8'	N1'	S1'	C19'	-102.49(17)
C8	N1	S1	O2	142.72(17)	C8'	N1'	S1'	O2'	141.74(16)
C8	N1	S1	O3	13.19(19)	C8'	N1'	S1'	O3'	11.84(19)
C9	C8	C12	C11	-38.0(2)	C9'	C8'	C12'	C11'	-41.9(2)
C9	C8	C12	N2	-166.59(19)	C9'	C8'	C12'	N2'	-169.97(18)
C9	C8	N1	C1	130.7(2)	C9'	C8'	N1'	C1'	128.23(18)
C9	C8	N1	S1	-57.4(2)	C9'	C8'	N1'	S1'	-60.3(2)
C9	C10	C11	C12	-22.3(3)	C9'	C10'	C11'	C12'	-13.1(3)
C10	C11	C12	C8	37.0(3)	C10'	C11'	C12'	C8'	33.9(2)
C10	C11	C12	N2	160.8(2)	C10'	C11'	C12'	N2'	158.6(2)
C11	C12	N2	C13	85.3(3)	C11'	C12'	N2'	C13'	79.3(3)
C12	C8	C9	C10	24.5(3)	C12'	C8'	C9'	C10'	33.7(2)
C12	C8	N1	C1	-109.87(19)	C12'	C8'	N1'	C1'	-113.69(18)
C12	C8	N1	S1	62.0(2)	C12'	C8'	N1'	S1'	57.8(2)
C13	C14	C15	C16	1.1(5)	C13'	C14'	C15'	C16'	1.1(4)
C14	C13	C18	C17	0.1(4)	C14'	C13'	C18'	C17'	-0.9(4)
C14	C13	N2	C12	168.0(2)	C14'	C13'	N2'	C12'	-4.9(3)
C14	C15	C16	C17	-0.3(5)	C14'	C15'	C16'	C17'	-1.3(5)
C15	C16	C17	C18	-0.6(5)	C15'	C16'	C17'	C18'	0.4(5)
C16	C17	C18	C13	0.7(4)	C16'	C17'	C18'	C13'	0.8(4)
C18	C13	C14	C15	-1.0(4)	C18'	C13'	C14'	C15'	0.0(3)
C18	C13	N2	C12	-11.9(4)	C18'	C13'	N2'	C12'	173.3(2)
C19	C20	C21	C22	0.0(4)	C19'	C20'	C21'	C22'	-0.2(4)
C20	C19	C24	C23	4.0(4)	C20'	C19'	C24'	C23'	3.2(4)
C20	C19	S1	N1	-86.0(2)	C20'	C19'	S1'	N1'	-85.0(2)
C20	C19	S1	O2	28.3(2)	C20'	C19'	S1'	O2'	29.5(2)
C20	C19	S1	O3	160.6(2)	C20'	C19'	S1'	O3'	161.79(19)
C20	C21	C22	C23	2.7(4)	C20'	C21'	C22'	C23'	3.1(4)
C20	C21	C22	C25	-175.4(3)	C20'	C21'	C22'	C25'	-174.0(2)
C21	C22	C23	C24	-2.0(4)	C21'	C22'	C23'	C24'	-2.9(4)
C21	C22	C25	F1	-35.7(4)	C21'	C22'	C25'	F1'	98.1(3)
C21	C22	C25	F2	-154.5(3)	C21'	C22'	C25'	F2'	-20.4(4)
C21	C22	C25	F3	81.5(4)	C21'	C22'	C25'	F3'	-140.6(3)
C22	C23	C24	C19	-1.3(4)	C22'	C23'	C24'	C19'	-0.3(4)
C23	C22	C25	F1	146.2(3)	C23'	C22'	C25'	F1'	-79.0(3)
C23	C22	C25	F2	27.4(4)	C23'	C22'	C25'	F2'	162.5(2)

C23	C22	C25	F3	-96.6(4)	C23'	C22'	C25'	F3'	42.3(4)
C24	C19	C20	C21	-3.3(4)	C24'	C19'	C20'	C21'	-3.0(4)
C24	C19	S1	N1	95.71(19)	C24'	C19'	S1'	N1'	94.11(19)
C24	C19	S1	O2	-149.95(19)	C24'	C19'	S1'	O2'	-151.36(18)
C24	C19	S1	O3	-17.6(2)	C24'	C19'	S1'	O3'	-19.1(2)
C25	C22	C23	C24	176.0(2)	C25'	C22'	C23'	C24'	174.3(2)
N1	C1	C2	C3	-173.9(2)	N1'	C1'	C2'	C3'	-175.1(2)
N1	C1	C2	C7	3.5(2)	N1'	C1'	C2'	C7'	3.5(2)
N1	C8	C9	C10	147.8(2)	N1'	C8'	C9'	C10'	157.27(19)
N1	C8	C12	C11	-164.56(18)	N1'	C8'	C12'	C11'	-167.42(17)
N1	C8	C12	N2	66.9(2)	N1'	C8'	C12'	N2'	64.5(2)
N2	C13	C14	C15	179.0(3)	N2'	C13'	C14'	C15'	178.2(2)
N2	C13	C18	C17	-179.9(2)	N2'	C13'	C18'	C17'	-179.2(2)
O1	C1	C2	C3	6.1(4)	O1'	C1'	C2'	C3'	4.6(4)
O1	C1	C2	C7	-176.5(2)	O1'	C1'	C2'	C7'	-176.8(2)
O1	C1	N1	C8	171.59(19)	O1'	C1'	N1'	C8'	173.46(19)
O1	C1	N1	S1	-0.8(3)	O1'	C1'	N1'	S1'	1.3(3)
S1	C19	C20	C21	178.4(2)	S1'	C19'	C20'	C21'	176.1(2)
S1	C19	C24	C23	-177.77(19)	S1'	C19'	C24'	C23'	-175.89(18)

Table S16 Hydrogen Atom Coordinates (Å×10^4) and Isotropic Displacement Parameters (Å^2×10^3) for 3f.

**Atom**	***x***	***y***	***z***	**U(eq)**
H3	1948	7744	4039	67
H4	2080	7970	5446	79
H5	975	7508	5886	82
H6	-285	6821	4945	74
H9A	-1753	7045	3230	74
H9B	-2151	6566	2387	74
H10A	-2128	6266	3981	97
H10B	-2554	5797	3131	97
H11A	-1339	5104	3949	77
H11B	-723	5700	4582	77
H12	-898	5399	2860	58
H14	2179	5028	4169	76
H15	2753	3942	4173	104
H16	1700	3027	3745	110
H17	67	3203	3323	95
H18	-526	4282	3348	74
H20	700	6225	1042	80

H21	1707	5359	1004	90
H23	111	3989	1555	80
H24	-921	4850	1568	72
H2	894	5747	4055	70
H3'	6862	7743	3518	75
H4'	6996	8105	4887	87
H5'	5899	7723	5390	82
H6'	4664	6972	4562	71
H9'A	3172	7095	2684	63
H9'B	2796	6457	2047	63
H10C	2988	6554	3769	81
H10D	2385	6002	3054	81
H11C	3561	5267	3838	69
H11D	4264	5859	4400	69
H12'	3992	5377	2710	54
H14'	4213	4352	3350	68
H15'	4641	3213	3530	85
H16'	6238	2885	4065	99
H17'	7434	3700	4377	92
H18'	7031	4846	4168	71
H20'	5780	5959	856	76
H21'	6916	5109	1073	81
H23'	5389	3820	1838	70
H24'	4247	4663	1613	64
H2'	5830	5708	3785	62

(2) Absolute configurations of **7** and **9** are determined by *X*-ray structure analysis of the product **7l.**

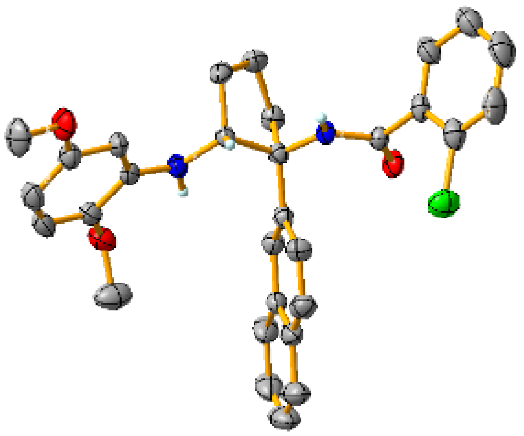

Fig. S19. Absolute configuration of **7l** (CCDC 2080353).

Displacement ellipsoids are drawn at the 30% probability level.

(Solvent: THF)

Table S17 Crystal data and structure refinement for 7l.

Identification code	**7l**
Empirical formula	$C_{30}H_{29}ClN_2O_3$
Formula weight	501.00
Temperature/K	293(2)
Crystal system	orthorhombic
Space group	$P2_12_12_1$
a/Å	12.3663(3)
b/Å	16.7129(3)
c/Å	25.3786(5)
α/°	90
β/°	90
γ/°	90
Volume/Å^3	5245.20(18)
Z	8
ρ_{calc}g/cm^3	1.269
μ/mm^{-1}	1.558
F(000)	2112.0
Crystal size/mm^3	0.16 × 0.09 × 0.06
Radiation	CuKα (λ = 1.54184)
2Θ range for data collection/°	6.966 to 134.156
Index ranges	$-12 \leq h \leq 14$, $-19 \leq k \leq 12$, $-30 \leq l \leq 30$
Reflections collected	19920
Independent reflections	9353 [R_{int} = 0.0420, R_{sigma} = 0.0626]
Data/restraints/parameters	9353/4/677
Goodness-of-fit on F^2	1.022
Final R indexes [I>=2σ (I)]	R_1 = 0.0576, wR_2 = 0.1329
Final R indexes [all data]	R_1 = 0.0870, wR_2 = 0.1549
Largest diff. peak/hole / e Å^{-3}	0.20/-0.19
Flack parameter	-0.013(19)

Table S18 Fractional Atomic Coordinates (×10^4) and Equivalent Isotropic Displacement Parameters (Å^2×10^3) for 7l. U_{eq} is defined as 1/3 of of the trace of the orthogonalised U_{IJ} tensor.

**Atom**	***x***	***y***	***z***	**U(eq)**
C1	4282(6)	7083(5)	3517(4)	103(2)
C2	3745(7)	7620(6)	3812(4)	122(3)
C3	4268(7)	8098(6)	4178(4)	115(3)
C4	5364(6)	8019(5)	4247(3)	96(2)

C5	5959(5)	7467(4)	3953(2)	68.9(15)
C6	5421(5)	6992(4)	3579(3)	77.8(17)
C7	6018(5)	6430(4)	3283(3)	81.0(18)
C8	7106(5)	6354(3)	3356(2)	68.3(15)
C9	7666(4)	6829(3)	3730(2)	54.8(12)
C10	7097(5)	7372(3)	4012(2)	63.8(14)
C11	8886(4)	6699(3)	3798.5(19)	51.6(11)
C12	9150(4)	5910(3)	4097.8(19)	53.9(12)
C13	10355(5)	6006(3)	4247(2)	66.6(14)
C14	10594(5)	6911(4)	4230(3)	78.1(17)
C15	9502(5)	7316(3)	4126(2)	62.8(13)
C16	9385(4)	7190(3)	2912(2)	57.3(12)
C17	10074(5)	7036(3)	2435(2)	66.5(15)
C18	9668(7)	6797(4)	1957(3)	86.3(19)
C19	10319(9)	6711(5)	1519(3)	110(3)
C20	11404(10)	6883(6)	1559(4)	121(3)
C21	11842(8)	7105(5)	2023(4)	112(3)
C22	11188(6)	7195(4)	2462(3)	90(2)
C23	8268(4)	5112(3)	4796.7(19)	58.7(13)
C24	7419(5)	5076(4)	5167(2)	68.1(15)
C25	7156(6)	4367(5)	5404(3)	89(2)
C26	7718(6)	3675(5)	5292(3)	89(2)
C27	8579(6)	3708(4)	4944(2)	78.3(17)
C28	8854(5)	4427(3)	4699(2)	66.0(14)
C29	5822(7)	5861(6)	5131(4)	128(3)
C30	8933(8)	2300(4)	5003(3)	119(3)
Cl1	8330.4(19)	6543.3(14)	1906.2(9)	107.1(7)
Cl1A	11831(19)	7248(15)	2965(8)	107.1(7)
N1	9406(4)	6620(2)	3280.9(16)	52.1(10)
N2	8471(4)	5841(3)	4559.4(18)	64.6(12)
O1	8873(3)	7817(2)	2965.0(15)	73.0(11)
O2	6902(4)	5797(3)	5282.7(19)	90.6(13)
O3	9228(4)	3068(3)	4807(2)	98.7(15)
C1'	3186(5)	5470(5)	4642(2)	88(2)
C2'	2966(7)	5449(6)	5172(3)	107(3)
C3'	2352(8)	4844(8)	5385(3)	120(3)
C4'	1940(7)	4252(6)	5075(3)	104(3)
C5'	2137(5)	4251(4)	4523(2)	75.1(17)
C6'	2770(5)	4869(4)	4302(2)	67.7(15)
C7'	2905(4)	4891(4)	3754(2)	63.3(14)

C8'	2461(4)	4333(3)	3428(2)	56.7(12)
C9'	1862(5)	3707(3)	3660(2)	69.2(15)
C10'	1707(6)	3665(4)	4181(3)	84.1(18)
C11'	2513(4)	4388(3)	2826(2)	56.8(12)
C12'	3278(5)	3769(3)	2555(2)	65.3(14)
C13'	3371(6)	4091(4)	1993(2)	85.5(19)
C14'	3157(7)	4996(4)	2023(3)	95(2)
C15'	2949(5)	5176(3)	2601(2)	69.1(15)
C16'	574(4)	4694(3)	2717(2)	56.8(12)
C17'	-488(5)	4454(3)	2473(2)	65.7(14)
C18'	-685(5)	4409(4)	1938(2)	77.0(16)
C19'	-1698(7)	4222(4)	1740(3)	97(2)
C20'	-2534(6)	4088(5)	2084(4)	105(2)
C21'	-2364(6)	4133(5)	2615(4)	101(2)
C22'	-1348(6)	4312(4)	2811(3)	89.3(19)
C23'	4646(4)	3124(4)	3142(2)	67.0(14)
C24'	5372(5)	3293(4)	3548(2)	76.8(17)
C25'	5733(7)	2704(5)	3876(3)	101(2)
C26'	5388(7)	1915(5)	3809(3)	101(2)
C27'	4673(6)	1740(4)	3425(3)	90(2)
C28'	4318(5)	2336(4)	3084(3)	79.6(18)
C29'	6163(10)	4356(6)	4032(3)	160(5)
C30'	4400(8)	399(5)	3723(4)	125(3)
Cl	-1267(8)	3994(6)	3471(3)	92.0(7)
Cl1'	338(2)	4588.4(14)	1470.3(8)	92.0(7)
N1'	1436(4)	4241(2)	2606.1(17)	58.4(11)
N2'	4333(4)	3741(3)	2819(2)	77.4(14)
O1'	625(3)	5277(2)	3012.9(15)	70.8(10)
O2'	5663(4)	4094(3)	3578.2(18)	97.5(15)
O3'	4270(5)	979(3)	3331(3)	128(2)

Table S19 Anisotropic Displacement Parameters (Å^2×10^3) for 7l. The Anisotropic displacement factor exponent takes the form: -2π^2[h^{2}a*^{2}U$_{11}$+2hka*b*U$_{12}$+…].

Atom	U_{11}	U_{22}	U_{33}	U_{23}	U_{13}	U_{12}
C1	66(4)	100(5)	143(7)	31(5)	-5(5)	0(4)
C2	65(5)	131(8)	171(10)	43(7)	19(6)	21(5)
C3	94(6)	131(7)	119(7)	31(6)	41(5)	52(6)
C4	88(5)	117(5)	82(4)	17(4)	26(4)	43(5)
C5	66(3)	78(4)	63(3)	20(3)	13(3)	13(3)
C6	62(4)	80(4)	92(4)	25(4)	4(3)	6(3)

C7	70(4)	75(4)	98(5)	3(3)	-15(4)	-10(3)
C8	66(3)	58(3)	80(4)	-7(3)	-4(3)	-1(3)
C9	60(3)	51(3)	54(3)	7(2)	3(2)	4(2)
C10	71(3)	63(3)	57(3)	4(3)	0(3)	6(3)
C11	55(3)	46(2)	54(3)	3(2)	4(2)	1(2)
C12	59(3)	53(3)	50(3)	7(2)	6(2)	4(2)
C13	61(3)	76(3)	63(3)	8(3)	4(3)	20(3)
C14	64(3)	91(4)	79(4)	7(3)	-13(3)	-8(3)
C15	76(4)	52(3)	61(3)	-5(2)	-1(3)	-2(3)
C16	62(3)	50(3)	60(3)	5(2)	0(3)	0(3)
C17	92(4)	53(3)	55(3)	13(2)	9(3)	5(3)
C18	112(5)	69(4)	78(4)	8(3)	11(4)	6(4)
C19	162(9)	104(6)	63(4)	-3(4)	12(5)	-2(6)
C20	147(9)	128(7)	88(6)	5(5)	47(6)	16(7)
C21	104(6)	111(6)	123(7)	27(5)	35(6)	1(5)
C22	87(5)	91(5)	92(5)	17(4)	30(4)	5(4)
C23	56(3)	74(3)	47(3)	12(2)	-3(2)	-4(3)
C24	59(3)	82(4)	64(3)	2(3)	3(3)	-2(3)
C25	79(4)	107(5)	81(4)	17(4)	18(4)	-24(4)
C26	96(5)	85(5)	87(5)	25(4)	4(4)	-28(4)
C27	86(5)	71(4)	79(4)	19(3)	-5(4)	-7(3)
C28	66(3)	68(3)	64(3)	22(3)	5(3)	-3(3)
C29	90(6)	155(8)	139(7)	-30(6)	-4(5)	30(6)
C30	166(9)	70(5)	121(6)	28(4)	7(6)	-18(5)
Cl1	99.5(14)	121.5(16)	100.4(14)	-19.4(12)	-15.1(12)	-4.9(13)
Cl1A	99.5(14)	121.5(16)	100.4(14)	-19.4(12)	-15.1(12)	-4.9(13)
N1	61(3)	42(2)	53(2)	3.5(18)	7(2)	8(2)
N2	67(3)	68(3)	59(3)	12(2)	14(2)	10(3)
O1	89(3)	52(2)	77(3)	15.9(18)	10(2)	16(2)
O2	67(3)	103(3)	102(3)	-1(3)	18(2)	-2(3)
O3	119(4)	64(2)	113(4)	29(2)	5(3)	-1(3)
C1'	70(4)	123(6)	72(4)	-32(4)	-12(3)	2(4)
C2'	88(5)	165(8)	68(4)	-37(5)	-16(4)	18(5)
C3'	110(7)	196(10)	54(4)	-10(5)	4(4)	47(7)
C4'	105(6)	146(7)	60(4)	24(4)	9(4)	30(5)
C5'	68(4)	97(5)	60(3)	10(3)	2(3)	23(4)
C6'	55(3)	95(4)	53(3)	-8(3)	-4(3)	12(3)
C7'	56(3)	74(3)	59(3)	-6(3)	1(2)	-8(3)
C8'	60(3)	52(3)	58(3)	-4(2)	-2(2)	0(2)
C9'	80(4)	60(3)	68(3)	4(3)	-2(3)	-7(3)

C10'	94(5)	77(4)	81(4)	18(3)	14(4)	2(4)
C11'	61(3)	55(3)	55(3)	-10(2)	-3(2)	-1(2)
C12'	59(3)	65(3)	71(3)	-17(3)	-5(3)	2(3)
C13'	84(4)	107(5)	65(4)	-26(3)	7(3)	3(4)
C14'	130(6)	88(4)	67(4)	3(3)	26(4)	-7(4)
C15'	84(4)	65(3)	59(3)	-1(3)	8(3)	-9(3)
C16'	66(3)	47(3)	57(3)	-5(2)	-5(3)	8(3)
C17'	69(3)	58(3)	70(3)	-12(3)	-4(3)	12(3)
C18'	83(4)	78(4)	70(4)	4(3)	-18(3)	5(3)
C19'	97(5)	106(5)	88(5)	7(4)	-33(4)	0(5)
C20'	74(5)	123(6)	119(7)	2(5)	-20(5)	2(5)
C21'	73(5)	115(6)	116(6)	-11(5)	11(4)	5(4)
C22'	77(4)	100(5)	91(5)	-19(4)	1(4)	11(4)
C23'	47(3)	86(4)	68(3)	-17(3)	3(3)	6(3)
C24'	62(3)	98(5)	70(4)	-15(3)	1(3)	-16(3)
C25'	90(5)	117(6)	95(5)	3(5)	-26(4)	-11(5)
C26'	97(5)	99(5)	108(6)	6(4)	-27(5)	2(5)
C27'	77(4)	76(4)	116(6)	-16(4)	-5(4)	7(4)
C28'	68(4)	76(4)	95(5)	-24(3)	-19(3)	12(3)
C29'	222(13)	143(8)	116(7)	1(6)	-59(8)	-75(9)
C30'	116(7)	89(5)	169(9)	-7(6)	38(6)	7(5)
Cl	108.4(16)	104.0(15)	63.5(10)	11.0(10)	-5.3(11)	-8.6(13)
Cl1'	108.4(16)	104.0(15)	63.5(10)	11.0(10)	-5.3(11)	-8.6(13)
N1'	67(3)	50(2)	58(2)	-14.4(19)	-7(2)	5(2)
N2'	60(3)	74(3)	98(4)	-13(3)	-12(3)	-5(3)
O1'	86(3)	56(2)	71(2)	-18.7(18)	-7(2)	15(2)
O2'	97(3)	110(4)	85(3)	-13(3)	-11(3)	-40(3)
O3'	133(5)	78(3)	173(6)	-8(4)	-48(4)	10(3)

Table S20 Bond Lengths for 7l.

Atom	Atom	Length/Å	Atom	Atom	Length/Å
C1	C2	1.345(12)	C1'	C2'	1.373(10)
C1	C6	1.426(9)	C1'	C6'	1.419(8)
C2	C3	1.384(12)	C2'	C3'	1.375(12)
C3	C4	1.374(10)	C3'	C4'	1.363(12)
C4	C5	1.396(9)	C4'	C5'	1.422(8)
C5	C6	1.405(9)	C5'	C6'	1.412(9)
C5	C10	1.426(8)	C5'	C10'	1.411(9)
C6	C7	1.411(9)	C6'	C7'	1.403(7)
C7	C8	1.364(8)	C7'	C8'	1.362(7)

C8	C9	1.419(7)	C8'	C9'	1.411(7)
C9	C10	1.354(7)	C8'	C11'	1.532(7)
C9	C11	1.534(7)	C9'	C10'	1.338(8)
C11	C12	1.556(6)	C11'	C12'	1.560(7)
C11	C15	1.527(7)	C11'	C15'	1.533(7)
C11	N1	1.469(6)	C11'	N1'	1.464(7)
C12	C13	1.546(7)	C12'	C13'	1.529(8)
C12	N2	1.446(6)	C12'	N2'	1.468(7)
C13	C14	1.543(8)	C13'	C14'	1.536(9)
C14	C15	1.533(8)	C14'	C15'	1.519(8)
C16	C17	1.504(8)	C16'	C17'	1.507(8)
C16	N1	1.336(6)	C16'	N1'	1.338(6)
C16	O1	1.232(6)	C16'	O1'	1.232(5)
C17	C18	1.371(9)	C17'	C18'	1.379(8)
C17	C22	1.404(9)	C17'	C22'	1.388(9)
C18	C19	1.381(10)	C18'	C19'	1.386(9)
C18	Cl1	1.712(8)	C18'	Cl1'	1.761(7)
C19	C20	1.376(12)	C19'	C20'	1.373(11)
C20	C21	1.348(12)	C20'	C21'	1.364(11)
C21	C22	1.385(10)	C21'	C22'	1.384(10)
C22	Cl1A	1.506(18)	C22'	Cl	1.759(10)
C23	C24	1.412(7)	C23'	C24'	1.395(8)
C23	C28	1.377(7)	C23'	C28'	1.387(8)
C23	N2	1.382(7)	C23'	N2'	1.374(8)
C24	C25	1.367(9)	C24'	C25'	1.364(9)
C24	O2	1.396(8)	C24'	O2'	1.388(7)
C25	C26	1.379(10)	C25'	C26'	1.395(10)
C26	C27	1.386(9)	C26'	C27'	1.349(10)
C27	C28	1.394(7)	C27'	C28'	1.389(9)
C27	O3	1.382(8)	C27'	O3'	1.386(8)
C29	O2	1.393(9)	C29'	O2'	1.378(8)
C30	O3	1.423(7)	C30'	O3'	1.400(10)

Table S21 Bond Angles for 7l.

Atom	Atom	Atom	Angle/°	Atom	Atom	Atom	Angle/°
C2	C1	C6	119.9(9)	C2'	C1'	C6'	120.3(8)
C1	C2	C3	121.9(8)	C1'	C2'	C3'	120.9(8)
C4	C3	C2	119.5(8)	C4'	C3'	C2'	120.9(7)
C3	C4	C5	120.9(8)	C3'	C4'	C5'	120.4(8)
C4	C5	C6	119.1(6)	C6'	C5'	C4'	119.0(7)

C4	C5	C10	122.5(7)	C10'	C5'	C4'	122.8(7)
C6	C5	C10	118.4(6)	C10'	C5'	C6'	118.2(5)
C5	C6	C1	118.8(7)	C5'	C6'	C1'	118.5(6)
C5	C6	C7	119.3(6)	C7'	C6'	C1'	122.8(6)
C7	C6	C1	122.0(7)	C7'	C6'	C5'	118.6(5)
C8	C7	C6	120.4(6)	C8'	C7'	C6'	122.5(6)
C7	C8	C9	121.4(6)	C7'	C8'	C9'	117.7(5)
C8	C9	C11	118.5(5)	C7'	C8'	C11'	123.2(5)
C10	C9	C8	118.5(5)	C9'	C8'	C11'	118.9(5)
C10	C9	C11	123.1(5)	C10'	C9'	C8'	121.8(6)
C9	C10	C5	122.1(6)	C9'	C10'	C5'	121.1(6)
C9	C11	C12	112.4(4)	C8'	C11'	C12'	115.2(4)
C15	C11	C9	117.2(4)	C8'	C11'	C15'	115.9(4)
C15	C11	C12	101.6(4)	C15'	C11'	C12'	101.1(4)
N1	C11	C9	110.0(4)	N1'	C11'	C8'	109.3(4)
N1	C11	C12	105.5(4)	N1'	C11'	C12'	105.8(4)
N1	C11	C15	109.2(4)	N1'	C11'	C15'	108.8(5)
C13	C12	C11	103.6(4)	C13'	C12'	C11'	102.9(5)
N2	C12	C11	110.0(4)	N2'	C12'	C11'	111.1(4)
N2	C12	C13	111.7(4)	N2'	C12'	C13'	111.7(5)
C14	C13	C12	106.2(4)	C12'	C13'	C14'	106.6(5)
C15	C14	C13	105.6(5)	C15'	C14'	C13'	105.8(5)
C11	C15	C14	103.6(4)	C14'	C15'	C11'	104.4(5)
N1	C16	C17	115.5(4)	N1'	C16'	C17'	117.2(4)
O1	C16	C17	121.6(5)	O1'	C16'	C17'	120.4(5)
O1	C16	N1	122.8(5)	O1'	C16'	N1'	122.4(5)
C18	C17	C16	123.6(6)	C18'	C17'	C16'	125.0(6)
C18	C17	C22	117.3(6)	C18'	C17'	C22'	117.7(6)
C22	C17	C16	118.9(6)	C22'	C17'	C16'	117.3(5)
C17	C18	C19	121.9(8)	C17'	C18'	C19'	121.9(7)
C17	C18	Cl1	119.6(6)	C17'	C18'	Cl1'	121.8(5)
C19	C18	Cl1	118.4(7)	C19'	C18'	Cl1'	116.3(5)
C20	C19	C18	119.2(8)	C20'	C19'	C18'	119.1(7)
C21	C20	C19	120.9(9)	C21'	C20'	C19'	120.2(7)
C20	C21	C22	119.9(9)	C20'	C21'	C22'	120.5(8)
C17	C22	Cl1A	124.8(12)	C17'	C22'	Cl	126.7(6)
C21	C22	C17	120.8(8)	C21'	C22'	C17'	120.6(7)
C21	C22	Cl1A	112.4(12)	C21'	C22'	Cl	109.2(7)
C28	C23	C24	118.4(5)	C28'	C23'	C24'	117.4(6)
C28	C23	N2	124.0(5)	N2'	C23'	C24'	118.1(6)

N2	C23	C24	117.6(5)	N2'	C23'	C28'	124.5(6)
C25	C24	C23	120.5(6)	C25'	C24'	C23'	120.9(6)
C25	C24	O2	123.1(6)	C25'	C24'	O2'	125.3(6)
O2	C24	C23	116.3(5)	O2'	C24'	C23'	113.8(6)
C24	C25	C26	121.0(6)	C24'	C25'	C26'	120.6(7)
C25	C26	C27	119.1(6)	C27'	C26'	C25'	119.5(7)
C26	C27	C28	120.4(6)	C26'	C27'	C28'	120.1(7)
O3	C27	C26	125.2(6)	C26'	C27'	O3'	124.0(7)
O3	C27	C28	114.5(6)	O3'	C27'	C28'	115.9(7)
C23	C28	C27	120.6(5)	C23'	C28'	C27'	121.5(6)
C16	N1	C11	123.6(4)	C16'	N1'	C11'	123.3(4)
C23	N2	C12	122.0(5)	C23'	N2'	C12'	123.2(5)
C29	O2	C24	116.6(6)	C29'	O2'	C24'	118.0(6)
C27	O3	C30	117.5(6)	C27'	O3'	C30'	118.2(7)

Table S22 Hydrogen Bonds for 7l.

D	H	A	d(D-H)/Å	d(H-A)/Å	d(D-A)/Å	D-H-A/°
N1	H1A	O1'[1]	0.82(4)	1.98(4)	2.786(5)	173(4)
N2	H2A	O2	0.84(3)	2.27(5)	2.673(6)	109(4)
N1'	H1'A	O1[2]	0.85(3)	1.97(3)	2.812(5)	167(6)

[1]1+X,+Y,+Z; [2]1-X,-1/2+Y,1/2-Z

Table S23 Torsion Angles for 7l.

A	B	C	D	Angle/°	A	B	C	D	Angle/°
C1	C2	C3	C4	-0.7(14)	C1'	C2'	C3'	C4'	-0.3(13)
C1	C6	C7	C8	-179.4(6)	C1'	C6'	C7'	C8'	-176.9(6)
C2	C1	C6	C5	0.3(11)	C2'	C1'	C6'	C5'	-0.6(10)
C2	C1	C6	C7	179.2(7)	C2'	C1'	C6'	C7'	175.5(6)
C2	C3	C4	C5	0.3(12)	C2'	C3'	C4'	C5'	-0.2(13)
C3	C4	C5	C6	0.4(10)	C3'	C4'	C5'	C6'	0.3(10)
C3	C4	C5	C10	179.7(6)	C3'	C4'	C5'	C10'	-178.2(7)
C4	C5	C6	C1	-0.6(9)	C4'	C5'	C6'	C1'	0.1(9)
C4	C5	C6	C7	-179.6(6)	C4'	C5'	C6'	C7'	-176.2(6)
C4	C5	C10	C9	179.2(6)	C4'	C5'	C10'	C9'	176.4(6)
C5	C6	C7	C8	-0.5(10)	C5'	C6'	C7'	C8'	-0.8(9)
C6	C1	C2	C3	0.4(13)	C6'	C1'	C2'	C3'	0.7(12)
C6	C5	C10	C9	-1.5(8)	C6'	C5'	C10'	C9'	-2.2(10)
C6	C7	C8	C9	0.3(10)	C6'	C7'	C8'	C9'	-1.2(9)
C7	C8	C9	C10	-0.7(9)	C6'	C7'	C8'	C11'	174.3(5)

C7	C8	C9	C11	178.8(5)	C7'	C8'	C9'	C10'	1.6(9)
C8	C9	C10	C5	1.3(8)	C7'	C8'	C11'	C12'	108.7(6)
C8	C9	C11	C12	-73.7(6)	C7'	C8'	C11'	C15'	-9.0(8)
C8	C9	C11	C15	169.0(5)	C7'	C8'	C11'	N1'	-132.3(5)
C8	C9	C11	N1	43.6(6)	C8'	C9'	C10'	C5'	0.2(10)
C9	C11	C12	C13	-166.0(4)	C8'	C11'	C12'	C13'	-167.0(5)
C9	C11	C12	N2	-46.5(6)	C8'	C11'	C12'	N2'	-47.3(6)
C9	C11	C15	C14	166.5(4)	C8'	C11'	C15'	C14'	167.2(5)
C9	C11	N1	C16	60.2(6)	C8'	C11'	N1'	C16'	60.2(6)
C10	C5	C6	C1	180.0(6)	C9'	C8'	C11'	C12'	-75.8(6)
C10	C5	C6	C7	1.1(9)	C9'	C8'	C11'	C15'	166.6(5)
C10	C9	C11	C12	105.7(6)	C9'	C8'	C11'	N1'	43.2(6)
C10	C9	C11	C15	-11.5(7)	C10'	C5'	C6'	C1'	178.7(6)
C10	C9	C11	N1	-137.0(5)	C10'	C5'	C6'	C7'	2.5(9)
C11	C9	C10	C5	-178.2(5)	C11'	C8'	C9'	C10'	-174.2(6)
C11	C12	C13	C14	21.3(5)	C11'	C12'	C13'	C14'	25.6(7)
C11	C12	N2	C23	158.2(5)	C11'	C12'	N2'	C23'	105.4(6)
C12	C11	C15	C14	43.5(5)	C12'	C11'	C15'	C14'	42.0(6)
C12	C11	N1	C16	-178.3(5)	C12'	C11'	N1'	C16'	-175.2(5)
C12	C13	C14	C15	5.4(6)	C12'	C13'	C14'	C15'	0.1(8)
C13	C12	N2	C23	-87.3(6)	C13'	C12'	N2'	C23'	-140.3(6)
C13	C14	C15	C11	-30.6(6)	C13'	C14'	C15'	C11'	-26.5(8)
C15	C11	C12	C13	-39.9(5)	C15'	C11'	C12'	C13'	-41.3(5)
C15	C11	C12	N2	79.7(5)	C15'	C11'	C12'	N2'	78.4(6)
C15	C11	N1	C16	-69.7(6)	C15'	C11'	N1'	C16'	-67.3(6)
C16	C17	C18	C19	175.8(6)	C16'	C17'	C18'	C19'	-176.8(6)
C16	C17	C18	Cl1	-7.9(8)	C16'	C17'	C18'	Cl1'	3.2(8)
C16	C17	C22	C21	-176.2(6)	C16'	C17'	C22'	C21'	176.3(6)
C16	C17	C22	Cl1A	21.2(15)	C16'	C17'	C22'	Cl	-27.0(10)
C17	C16	N1	C11	173.0(5)	C17'	C16'	N1'	C11'	-178.4(5)
C17	C18	C19	C20	-1.1(12)	C17'	C18'	C19'	C20'	1.0(11)
C18	C17	C22	C21	-0.4(10)	C18'	C17'	C22'	C21'	-0.2(10)
C18	C17	C22	Cl1A	-163.0(13)	C18'	C17'	C22'	Cl	156.6(6)
C18	C19	C20	C21	2.3(14)	C18'	C19'	C20'	C21'	-0.6(12)
C19	C20	C21	C22	-2.6(14)	C19'	C20'	C21'	C22'	-0.2(13)
C20	C21	C22	C17	1.6(12)	C20'	C21'	C22'	C17'	0.6(12)
C20	C21	C22	Cl1A	166.2(13)	C20'	C21'	C22'	Cl	-159.8(8)
C22	C17	C18	C19	0.2(10)	C22'	C17'	C18'	C19'	-0.6(10)
C22	C17	C18	Cl1	176.5(5)	C22'	C17'	C18'	Cl1'	179.3(5)
C23	C24	C25	C26	0.7(10)	C23'	C24'	C25'	C26'	0.5(12)

C23	C24	O2	C29	114.1(7)	C23'	C24'	O2'	C29'	164.9(7)
C24	C23	C28	C27	2.9(9)	C24'	C23'	C28'	C27'	1.7(9)
C24	C23	N2	C12	-166.3(5)	C24'	C23'	N2'	C12'	-150.8(5)
C24	C25	C26	C27	1.6(11)	C24'	C25'	C26'	C27'	-1.5(13)
C25	C24	O2	C29	-67.6(9)	C25'	C24'	O2'	C29'	-14.7(11)
C25	C26	C27	C28	-1.8(11)	C25'	C26'	C27'	C28'	2.6(13)
C25	C26	C27	O3	178.2(7)	C25'	C26'	C27'	O3'	-179.1(8)
C26	C27	C28	C23	-0.5(10)	C26'	C27'	C28'	C23'	-2.8(11)
C26	C27	O3	C30	5.8(10)	C26'	C27'	O3'	C30'	15.7(12)
C28	C23	C24	C25	-3.0(9)	C28'	C23'	C24'	C25'	-0.6(10)
C28	C23	C24	O2	175.4(5)	C28'	C23'	C24'	O2'	179.8(5)
C28	C23	N2	C12	14.8(9)	C28'	C23'	N2'	C12'	31.4(9)
C28	C27	O3	C30	-174.2(6)	C28'	C27'	O3'	C30'	-166.0(7)
Cl1	C18	C19	C20	-177.5(7)	Cl1'	C18'	C19'	C20'	-179.0(6)
N1	C11	C12	C13	74.1(5)	N1'	C11'	C12'	C13'	72.1(5)
N1	C11	C12	N2	-166.4(4)	N1'	C11'	C12'	N2'	-168.2(5)
N1	C11	C15	C14	-67.7(5)	N1'	C11'	C15'	C14'	-69.1(6)
N1	C16	C17	C18	103.8(7)	N1'	C16'	C17'	C18'	-60.5(8)
N1	C16	C17	C22	-80.7(7)	N1'	C16'	C17'	C22'	123.3(6)
N2	C12	C13	C14	-97.0(5)	N2'	C12'	C13'	C14'	-93.6(6)
N2	C23	C24	C25	178.0(6)	N2'	C23'	C24'	C25'	-178.5(6)
N2	C23	C24	O2	-3.7(8)	N2'	C23'	C24'	O2'	1.9(8)
N2	C23	C28	C27	-178.2(6)	N2'	C23'	C28'	C27'	179.5(6)
O1	C16	C17	C18	-79.0(8)	O1'	C16'	C17'	C18'	120.7(6)
O1	C16	C17	C22	96.6(7)	O1'	C16'	C17'	C22'	-55.5(8)
O1	C16	N1	C11	-4.2(8)	O1'	C16'	N1'	C11'	0.4(8)
O2	C24	C25	C26	-177.5(6)	O2'	C24'	C25'	C26'	-179.9(7)
O3	C27	C28	C23	179.5(5)	O3'	C27'	C28'	C23'	178.8(6)

Table S24 Hydrogen Atom Coordinates (Å×10^4) and Isotropic Displacement Parameters (Å^2×10^3) for 7l.

Atom	*x*	*y*	*z*	U(eq)
H1	3911	6773	3272	123
H2	3001	7673	3769	147
H3	3880	8469	4375	138
H4	5716	8337	4494	115
H7	5668	6109	3036	97
H8	7488	5982	3156	82
H10	7462	7693	4253	77
H12	9050	5447	3866	65

H13A	10488	5794	4597	80
H13B	10811	5722	3998	80
H14A	10896	7091	4562	94
H14B	11103	7034	3951	94
H15A	9128	7429	4453	75
H15B	9596	7811	3931	75
H18	8932	6690	1928	104
H19	10027	6540	1201	132
H20	11841	6845	1262	145
H21	12581	7199	2048	135
H22	11491	7361	2779	108
H25	6589	4352	5645	107
H26	7521	3193	5449	107
H28	9437	4444	4468	79
H29A	5765	5785	4757	192
H29B	5555	6382	5222	192
H29C	5404	5460	5310	192
H30A	9378	1899	4840	179
H30B	8188	2197	4922	179
H30C	9035	2287	5377	179
H1A	9710(30)	6210(20)	3195(16)	29(11)
H2A	7970(30)	6180(20)	4600(20)	52(15)
H1'	3610	5879	4505	106
H2'	3237	5849	5390	129
H3'	2215	4840	5745	144
H4'	1528	3847	5225	124
H7'	3314	5302	3607	76
H9'	1567	3313	3445	83
H10'	1310	3243	4321	101
H12'	2944	3238	2553	78
H13C	2843	3835	1765	103
H13D	4087	3989	1853	103
H14C	3779	5293	1897	114
H14D	2534	5139	1811	114
H15C	2422	5601	2639	83
H15D	3612	5332	2778	83
H18'	-121	4508	1705	92
H19'	-1809	4187	1378	116
H20'	-3219	3967	1956	127
H21'	-2934	4044	2846	122

H22'	-1240	4336	3174	107
H25'	6212	2829	4146	121
H26'	5650	1514	4028	122
H28'	3850	2202	2811	96
H29D	6168	4930	4038	241
H29E	6893	4161	4041	241
H29F	5776	4159	4333	241
H30D	4115	-102	3602	187
H30E	4021	562	4035	187
H30F	5155	338	3802	187
H1'A	1400(60)	3770(20)	2470(20)	100(20)
H2'A	4560(60)	4200(30)	2920(30)	120(30)

Table S25 Atomic Occupancy for 7l.

**Atom**	***Occupancy***	**Atom**	***Occupancy***	**Atom**	***Occupancy***
H18	0.087(3)	H22	0.913(3)	Cl1	0.913(3)
Cl1A	0.087(3)	H18'	0.197(3)	H22'	0.803(3)
Cl	0.197(3)	Cl1'	0.803(3)		

(3) Absolute configurations of **11**, **15**, **16** and **17** are determined by *X*-ray structure analysis of the product **15.**

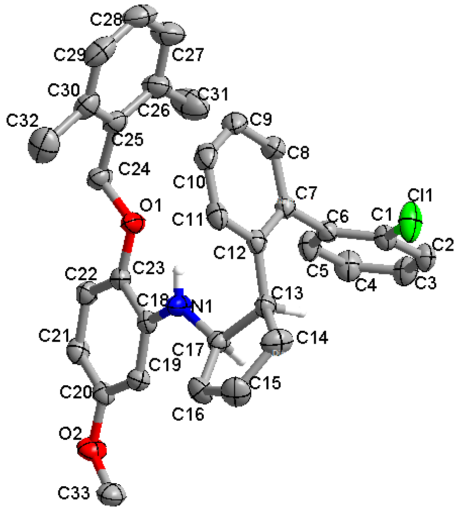

Fig. S20. Absolute configuration of **15** (CCDC 2080354).

Displacement ellipsoids are drawn at the 30% probability level.

(Solvent: diethyl ether/hexane = 5:1)

Table S26 Crystal data and structure refinement for 20210476.

Identification code	20210476
Empirical formula	$C_{33}H_{34}ClNO_2$
Formula weight	512.06
Temperature/K	293(2)

Crystal system	monoclinic
Space group	$P2_1$
a/Å	11.1324(7)
b/Å	7.5493(6)
c/Å	16.7445(13)
α/°	90
β/°	90.526(7)
γ/°	90
Volume/Å^3	1407.18(18)
Z	2
ρ_{calc}g/cm^3	1.209
μ/mm^{-1}	1.423
F(000)	544.0
Crystal size/mm^3	0.13 × 0.11 × 0.1
Radiation	CuKα (λ = 1.54184)
2Θ range for data collection/°	7.942 to 145.108
Index ranges	-13 ≤ h ≤ 12, -9 ≤ k ≤ 8, -18 ≤ l ≤ 20
Reflections collected	12005
Independent reflections	4982 [R_{int} = 0.0351, R_{sigma} = 0.0427]
Data/restraints/parameters	4982/6/350
Goodness-of-fit on F^2	1.022
Final R indexes [I>=2σ (I)]	R_1 = 0.0505, wR_2 = 0.1279
Final R indexes [all data]	R_1 = 0.0629, wR_2 = 0.1415
Largest diff. peak/hole / e Å^{-3}	0.12/-0.26
Flack parameter	0.018(14)

Table S27 Fractional Atomic Coordinates (×10^4) and Equivalent Isotropic Displacement Parameters (Å^2×10^3) for 20210476. U_{eq} is defined as 1/3 of of the trace of the orthogonalised U_{IJ} tensor.

Atom	*x*	*y*	*z*	U(eq)
C1	6889(3)	3880(6)	2353(2)	60.5(9)
C2	7668(4)	5291(6)	2234(3)	66.5(10)
C3	7226(4)	6970(6)	2297(3)	73.0(12)
C4	6047(5)	7266(7)	2467(3)	81.4(13)
C5	5287(4)	5852(6)	2586(3)	74.1(12)
C6	5687(3)	4106(6)	2531(2)	56.2(8)
C7	4872(3)	2589(5)	2701(2)	55.8(9)
C8	4710(4)	2060(7)	3486(3)	73.6(12)
C9	3956(4)	680(8)	3683(3)	81.5(14)
C10	3327(4)	-151(7)	3101(3)	78.8(13)
C11	3467(4)	336(7)	2311(3)	70.6(11)

C12	4241(3)	1702(6)	2092(2)	54.5(8)
C13	4330(3)	2298(6)	1232(2)	61.5(10)
C14	4530(40)	1060(40)	527(17)	86(4)
C15	3610(40)	1340(100)	-120(30)	86(4)
C16	2860(20)	2920(30)	162(14)	75(3)
C17	3298(3)	3555(6)	949(2)	57.0(9)
C18	1816(3)	5561(5)	1587(2)	51.3(8)
C19	1544(3)	6553(6)	903(2)	55.0(8)
C20	788(3)	8011(5)	943(2)	57.1(9)
C21	310(4)	8530(6)	1661(3)	64.4(10)
C22	585(4)	7554(6)	2347(2)	62.4(10)
C23	1316(3)	6105(6)	2316(2)	55.5(9)
C24	1035(4)	5335(7)	3686(3)	71.8(11)
C25	1440(4)	3981(7)	4284(2)	69.9(11)
C26	2352(5)	4407(9)	4831(3)	88.3(15)
C27	2666(7)	3190(11)	5408(3)	110(2)
C28	2120(7)	1597(11)	5454(4)	108(2)
C29	1254(6)	1113(9)	4905(4)	101(2)
C30	900(5)	2318(8)	4308(3)	81.4(14)
C31	2998(8)	6155(12)	4796(5)	136(3)
C32	-43(6)	1816(12)	3702(4)	115(2)
C33	805(5)	8378(8)	-473(3)	83.4(14)
Cl1	7453.0(11)	1743.4(19)	2245.6(12)	103.1(5)
N1	2510(3)	4069(6)	1583(2)	70.1(10)
O1	1662(3)	5074(5)	2963.6(16)	72.3(8)
O2	455(3)	9002(5)	291.2(19)	78.9(9)
C14A	4260(30)	680(20)	661(12)	86(4)
C15A	3730(30)	1530(60)	-70(20)	86(4)
C16A	2607(13)	2290(20)	339(9)	75(3)

Table S28 Anisotropic Displacement Parameters (Å^2×10^3) for 20210476. The Anisotropic displacement factor exponent takes the form: $-2\pi^2[h^2a^{*2}U_{11}+2hka^*b^*U_{12}+\ldots]$.

Atom	U_{11}	U_{22}	U_{33}	U_{23}	U_{13}	U_{12}
C1	57(2)	53(2)	72(2)	-1.2(18)	-0.8(16)	1.0(17)
C2	55(2)	63(3)	82(3)	-4(2)	0.9(17)	-8.4(18)
C3	73(3)	53(3)	93(3)	-1(2)	3(2)	-13(2)
C4	82(3)	51(3)	112(4)	0(2)	3(3)	2(2)
C5	58(2)	62(3)	102(3)	-1(2)	4(2)	6(2)
C6	54.2(18)	57(2)	57.0(19)	1.6(16)	-4.6(14)	-1.1(16)
C7	44.5(17)	56(2)	66(2)	6.7(17)	0.3(14)	2.3(15)

C8	66(2)	88(4)	66(2)	11(2)	-5.7(18)	-4(2)
C9	72(3)	95(4)	78(3)	35(3)	6(2)	0(3)
C10	60(2)	74(3)	102(3)	25(3)	2(2)	-10(2)
C11	50.4(19)	61(3)	100(3)	4(2)	-9.6(19)	-6.2(17)
C12	43.9(15)	51(2)	68(2)	2.4(17)	-1.2(14)	6.7(16)
C13	50.2(18)	72(3)	62(2)	-3.8(18)	1.7(15)	7.0(17)
C14	115(14)	71(10)	73(7)	-4(7)	15(5)	26(7)
C15	95(6)	86(10)	78(4)	-34(5)	-4(5)	-5(6)
C16	72(6)	77(10)	77(7)	-7(5)	-15(5)	-2(5)
C17	58.6(19)	58(2)	54.4(19)	2.6(17)	5.6(15)	5.9(17)
C18	45.3(16)	53(2)	55.8(19)	0.0(15)	-0.8(13)	2.3(14)
C19	59.2(18)	53(2)	52.5(18)	-1.8(16)	-1.2(14)	4.0(17)
C20	54.4(19)	48(2)	69(2)	7.8(16)	-5.7(16)	0.5(15)
C21	60(2)	53(2)	80(3)	1.4(19)	5.1(18)	8.5(18)
C22	55(2)	70(3)	62(2)	-4.6(19)	6.9(16)	7.7(18)
C23	45.2(16)	65(3)	56(2)	3.6(16)	1.8(14)	1.6(15)
C24	77(3)	83(3)	55(2)	0(2)	11.4(18)	9(2)
C25	75(3)	83(3)	52(2)	3(2)	12.2(17)	4(2)
C26	102(4)	98(4)	65(3)	6(2)	-9(2)	-8(3)
C27	136(6)	127(6)	67(3)	18(3)	-13(3)	0(5)
C28	140(5)	114(5)	69(3)	30(3)	18(3)	16(5)
C29	119(5)	86(4)	99(4)	9(3)	47(4)	-2(3)
C30	85(3)	88(4)	71(3)	-8(2)	24(2)	-3(3)
C31	158(7)	120(7)	129(6)	9(5)	-49(5)	-30(5)
C32	112(4)	108(5)	125(5)	-29(4)	11(4)	-16(4)
C33	94(3)	87(4)	70(3)	20(2)	-2(2)	14(3)
Cl1	64.0(6)	57.8(6)	187.9(16)	-3.0(9)	16.0(7)	5.0(6)
N1	78(2)	79(3)	53.5(19)	12.7(18)	10.7(15)	27(2)
O1	69.4(17)	95(2)	52.5(15)	11.9(14)	9.0(12)	24.8(16)
O2	94(2)	66(2)	75.8(19)	15.9(16)	-3.1(15)	20.0(17)
C14A	115(14)	71(10)	73(7)	-4(7)	15(5)	26(7)
C15A	95(6)	86(10)	78(4)	-34(5)	-4(5)	-5(6)
C16A	72(6)	77(10)	77(7)	-7(5)	-15(5)	-2(5)

Table S29 Bond Lengths for 20210476.

Atom	Atom	Length/Å	Atom	Atom	Length/Å
C1	C2	1.389(6)	C18	C19	1.400(5)
C1	C6	1.384(5)	C18	C23	1.407(5)
C1	Cl1	1.740(4)	C18	N1	1.366(5)
C2	C3	1.364(7)	C19	C20	1.388(6)

C3	C4	1.364(7)	C20	C21	1.377(6)
C4	C5	1.378(7)	C20	O2	1.371(5)
C5	C6	1.394(6)	C21	C22	1.396(6)
C6	C7	1.490(5)	C22	C23	1.365(6)
C7	C8	1.389(6)	C23	O1	1.386(5)
C7	C12	1.403(5)	C24	C25	1.497(7)
C8	C9	1.380(7)	C24	O1	1.416(5)
C9	C10	1.350(8)	C25	C26	1.398(7)
C10	C11	1.382(7)	C25	C30	1.393(8)
C11	C12	1.395(6)	C26	C27	1.376(9)
C12	C13	1.512(6)	C26	C31	1.504(10)
C13	C14	1.52(2)	C27	C28	1.350(11)
C13	C17	1.561(5)	C28	C29	1.375(10)
C13	C14A	1.555(16)	C29	C30	1.405(9)
C14	C15	1.49(2)	C30	C32	1.503(8)
C15	C16	1.53(5)	C33	O2	1.422(6)
C16	C17	1.48(2)	C14A	C15A	1.50(2)
C17	N1	1.437(5)	C15A	C16A	1.54(3)
C17	C16A	1.592(15)			

Table S30 Bond Angles for 20210476.

Atom	Atom	Atom	Angle/°	Atom	Atom	Atom	Angle/°
C2	C1	Cl1	118.0(3)	N1	C17	C16A	110.0(6)
C6	C1	C2	122.8(4)	C19	C18	C23	118.0(3)
C6	C1	Cl1	119.1(3)	N1	C18	C19	123.7(3)
C3	C2	C1	118.4(4)	N1	C18	C23	118.2(3)
C4	C3	C2	121.1(4)	C20	C19	C18	120.8(3)
C3	C4	C5	119.8(5)	C21	C20	C19	120.5(4)
C4	C5	C6	121.7(4)	O2	C20	C19	123.6(4)
C1	C6	C5	116.2(4)	O2	C20	C21	115.9(4)
C1	C6	C7	122.5(4)	C20	C21	C22	119.0(4)
C5	C6	C7	121.2(4)	C23	C22	C21	121.2(4)
C8	C7	C6	119.1(4)	C22	C23	C18	120.5(3)
C8	C7	C12	118.8(4)	C22	C23	O1	125.5(3)
C12	C7	C6	122.0(3)	O1	C23	C18	113.9(3)
C9	C8	C7	121.9(5)	O1	C24	C25	109.2(4)
C10	C9	C8	119.4(4)	C26	C25	C24	119.5(5)
C9	C10	C11	120.3(4)	C30	C25	C24	120.5(4)
C10	C11	C12	121.6(4)	C30	C25	C26	120.0(5)
C7	C12	C13	120.8(4)	C25	C26	C31	121.4(6)

C11	C12	C7	117.9(4)	C27	C26	C25	118.9(6)
C11	C12	C13	121.1(4)	C27	C26	C31	119.7(6)
C12	C13	C14	124.6(14)	C28	C27	C26	121.6(6)
C12	C13	C17	114.6(3)	C27	C28	C29	120.7(6)
C12	C13	C14A	110.4(8)	C28	C29	C30	119.6(6)
C14	C13	C17	104.3(19)	C25	C30	C29	119.1(5)
C14A	C13	C17	104.9(12)	C25	C30	C32	120.5(6)
C15	C14	C13	112(4)	C29	C30	C32	120.4(6)
C14	C15	C16	105(4)	C18	N1	C17	125.1(4)
C17	C16	C15	111(2)	C23	O1	C24	117.1(3)
C13	C17	C16A	100.3(6)	C20	O2	C33	117.5(4)
C16	C17	C13	107.8(10)	C15A	C14A	C13	101(2)
N1	C17	C13	113.1(3)	C14A	C15A	C16A	96(2)
N1	C17	C16	123.4(10)	C15A	C16A	C17	97.0(18)

Table S31 Torsion Angles for 20210476.

A	B	C	D	Angle/°	A	B	C	D	Angle/°
C1	C2	C3	C4	-0.5(7)	C17	C13	C14A	C15A	28.0(17)
C1	C6	C7	C8	93.2(5)	C18	C19	C20	C21	1.3(6)
C1	C6	C7	C12	-88.3(5)	C18	C19	C20	O2	-177.8(4)
C2	C1	C6	C5	-0.1(6)	C18	C23	O1	C24	-169.0(4)
C2	C1	C6	C7	-176.6(4)	C19	C18	C23	C22	0.3(6)
C2	C3	C4	C5	0.7(8)	C19	C18	C23	O1	-178.3(3)
C3	C4	C5	C6	-0.6(8)	C19	C18	N1	C17	16.8(7)
C4	C5	C6	C1	0.3(7)	C19	C20	C21	C22	-0.8(6)
C4	C5	C6	C7	176.9(5)	C19	C20	O2	C33	6.9(6)
C5	C6	C7	C8	-83.2(5)	C20	C21	C22	C23	0.1(7)
C5	C6	C7	C12	95.3(5)	C21	C20	O2	C33	-172.2(4)
C6	C1	C2	C3	0.1(6)	C21	C22	C23	C18	0.2(6)
C6	C7	C8	C9	179.2(4)	C21	C22	C23	O1	178.6(4)
C6	C7	C12	C11	-177.9(4)	C22	C23	O1	C24	12.5(6)
C6	C7	C12	C13	-2.2(5)	C23	C18	C19	C20	-1.0(6)
C7	C8	C9	C10	-2.1(8)	C23	C18	N1	C17	-165.1(4)
C7	C12	C13	C14	134(2)	C24	C25	C26	C27	176.1(5)
C7	C12	C13	C17	-95.8(4)	C24	C25	C26	C31	-4.3(9)
C7	C12	C13	C14A	146.0(13)	C24	C25	C30	C29	-176.5(4)
C8	C7	C12	C11	0.7(6)	C24	C25	C30	C32	3.8(7)
C8	C7	C12	C13	176.3(4)	C25	C24	O1	C23	173.9(4)
C8	C9	C10	C11	2.2(8)	C25	C26	C27	C28	0.5(11)
C9	C10	C11	C12	-0.9(8)	C26	C25	C30	C29	2.4(7)

C10	C11	C12	C7	-0.5(6)	C26	C25	C30	C32	-177.3(5)
C10	C11	C12	C13	-176.2(4)	C26	C27	C28	C29	2.2(11)
C11	C12	C13	C14	-51(2)	C27	C28	C29	C30	-2.5(10)
C11	C12	C13	C17	79.7(5)	C28	C29	C30	C25	0.2(8)
C11	C12	C13	C14A	-38.4(13)	C28	C29	C30	C32	179.9(6)
C12	C7	C8	C9	0.6(7)	C30	C25	C26	C27	-2.8(9)
C12	C13	C14	C15	128(3)	C30	C25	C26	C31	176.8(6)
C12	C13	C17	C16	-133.9(10)	C31	C26	C27	C28	-179.1(8)
C12	C13	C17	N1	6.0(5)	Cl1	C1	C2	C3	178.4(4)
C12	C13	C17	C16A	-111.1(7)	Cl1	C1	C6	C5	-178.3(3)
C12	C13	C14A	C15A	151.9(14)	Cl1	C1	C6	C7	5.1(5)
C13	C14	C15	C16	5(5)	N1	C17	C16A	C15A	-162.7(16)
C13	C17	N1	C18	156.4(4)	N1	C18	C19	C20	177.0(4)
C13	C17	C16A	C15A	-43.3(17)	N1	C18	C23	C22	-177.9(4)
C13	C14A	C15A	C16A	-56(3)	N1	C18	C23	O1	3.6(5)
C14	C13	C17	C16	5.6(14)	O1	C24	C25	C26	94.7(6)
C14	C13	C17	N1	145.5(13)	O1	C24	C25	C30	-86.3(5)
C14	C15	C16	C17	-1(5)	O2	C20	C21	C22	178.3(4)
C15	C16	C17	C13	-3(3)	C14A	C13	C17	N1	127.2(8)
C15	C16	C17	N1	-138(3)	C14A	C13	C17	C16A	10.1(8)
C16	C17	N1	C18	-70.8(13)	C14A	C15A	C16A	C17	62(2)
C17	C13	C14	C15	-7(3)	C16A	C17	N1	C18	-92.4(8)

Table S32 Hydrogen Atom Coordinates (Å×10^4) and Isotropic Displacement Parameters (Å^2×10^3) for 20210476.

Atom	*x*	*y*	*z*	U(eq)
H2	8471	5096	2114	80
H3	7737	7929	2222	88
H4	5757	8418	2502	98
H5	4487	6067	2707	89
H8	5121	2653	3892	88
H9	3882	325	4212	98
H10	2797	-1055	3231	95
H11	3036	-262	1917	85
H13	5043	3060	1231	74
H13A	5102	2896	1157	74
H14A	5320	1269	312	103
H14B	4491	-153	710	103
H15A	3106	294	-180	104
H15B	3990	1591	-623	104

H16A	2922	3870	-226	90
H16B	2028	2576	200	90
H17	3719	4657	820	68
H17A	3620	4597	675	68
H19	1875	6232	416	66
H21	-189	9516	1689	77
H22	264	7897	2834	75
H24A	178	5221	3591	86
H24B	1193	6515	3890	86
H27	3268	3472	5775	132
H28	2332	817	5862	129
H29	904	-5	4929	121
H31A	3095	6500	4248	204
H31B	3773	6043	5047	204
H31C	2538	7038	5069	204
H32A	-706	2625	3735	172
H32B	-319	634	3807	172
H32C	292	1867	3176	172
H33A	460	9122	-880	125
H33B	527	7185	-545	125
H33C	1665	8407	-511	125
H1	2560(40)	3640(60)	2020(30)	53(11)
H14C	5044	183	558	103
H14D	3737	-242	869	103
H15C	3524	671	-483	104
H15D	4240	2443	-288	104
H16C	2143	1384	609	90
H16D	2094	2941	-28	90

Table S33 Atomic Occupancy for 20210476.

**Atom**	***Occupancy***	**Atom**	***Occupancy***	**Atom**	***Occupancy***
H13	0.408(19)	H13A	0.592(19)	C14	0.408(19)
H14A	0.408(19)	H14B	0.408(19)	C15	0.408(19)
H15A	0.408(19)	H15B	0.408(19)	C16	0.408(19)
H16A	0.408(19)	H16B	0.408(19)	H17	0.408(19)
H17A	0.592(19)	C14A	0.592(19)	H14C	0.592(19)
H14D	0.592(19)	C15A	0.592(19)	H15C	0.592(19)
H15D	0.592(19)	C16A	0.592(19)	H16C	0.592(19)
H16D	0.592(19)				

(4) Absolute configurations of **13**, **18**, **19**, **20** and **21** are determined by *X*-ray structure analysis of the product **13m.**

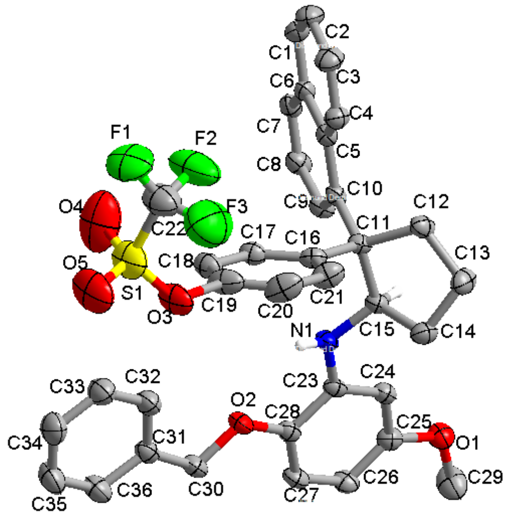

Fig. S21. Absolute configuration of **13m** (CCDC 2080355).

Displacement ellipsoids are drawn at the 30% probability level.

(Solvent: diethyl ether/hexane = 1:1)

Table S34 Crystal data and structure refinement for 13m.

Identification code	**13m**
Empirical formula	$C_{36}H_{32}F_3NO_5S$
Formula weight	647.68
Temperature/K	293(2)
Crystal system	monoclinic
Space group	C2
a/Å	29.3277(9)
b/Å	7.49483(19)
c/Å	18.2663(6)
α/°	90
β/°	124.382(5)
γ/°	90
Volume/Å^3	3313.6(2)
Z	4
ρ_{calc}g/cm^3	1.298
μ/mm^{-1}	1.378
F(000)	1352.0
Crystal size/mm^3	0.16 × 0.1 × 0.07
Radiation	CuKα (λ = 1.54184)
2Θ range for data collection/°	7.304 to 141.744

Index ranges	$-35 \le h \le 27$, $-7 \le k \le 9$, $-22 \le l \le 22$
Reflections collected	15762
Independent reflections	5649 [R_{int} = 0.0287, R_{sigma} = 0.0295]
Data/restraints/parameters	5649/89/446
Goodness-of-fit on F^2	1.043
Final R indexes [I>=2σ (I)]	R_1 = 0.0518, wR_2 = 0.1436
Final R indexes [all data]	R_1 = 0.0580, wR_2 = 0.1521
Largest diff. peak/hole / e $Å^{-3}$	0.25/-0.28
Flack parameter	-0.006(15)

Table S35 Fractional Atomic Coordinates (×10[4]) and Equivalent Isotropic Displacement Parameters ($Å^2$×10[3]) for 13m. U_{eq} is defined as 1/3 of of the trace of the orthogonalised U_{IJ} tensor.

Atom	*x*	*y*	*z*	U(eq)
S1	8549.0(10)	3574(7)	10124.1(17)	142.0(14)
S1A	8448(3)	5103(17)	9846(5)	142.0(14)
F1	8349(4)	5621(16)	11061(6)	186(4)
F2	7681(4)	5050(20)	9798(8)	226(6)
F3	8222(7)	6623(18)	9815(10)	250(6)
F1A	8428(12)	4260(40)	11203(16)	186(4)
F2A	7731(11)	3580(60)	10040(20)	226(6)
F3A	8478(16)	2110(30)	10410(20)	250(6)
O1	5709.0(12)	-2066(4)	2670.2(18)	71.0(8)
O2	7502.2(9)	-1740(4)	6150.8(17)	61.2(7)
O3	8362(4)	3495(19)	9186(8)	110(4)
O4	8262(6)	2000(20)	10308(9)	240(7)
O5	9115(3)	3777(19)	10679(6)	182(5)
O3A	8253(12)	4130(60)	9020(20)	110(4)
O4A	8483(13)	6980(30)	10260(20)	240(7)
O5A	9072(8)	4780(40)	10371(18)	182(5)
N1	6612.3(12)	265(4)	5594(2)	54.1(7)
C1	5203.9(17)	1551(7)	7592(3)	68.3(11)
C2	5328(2)	3220(8)	7915(3)	79.6(13)
C3	5644(2)	4335(7)	7747(3)	77.3(12)
C4	5819.9(17)	3741(6)	7236(3)	62.5(9)
C5	5679.4(13)	2019(5)	6850(2)	50.0(7)
C6	5372.0(13)	879(5)	7055(2)	54.3(8)
C7	5248.3(13)	-866(5)	6736(3)	59.2(9)
C8	5405.9(15)	-1493(6)	6218(3)	62.1(9)
C9	5692.1(13)	-383(5)	5980(2)	54.2(8)
C10	5834.6(12)	1335(5)	6280(2)	46.9(7)

C11	6158.4(13)	2513(5)	6039(2)	49.0(7)
C12	5834.8(17)	4267(6)	5559(3)	63.0(9)
C13	5876(2)	4623(6)	4772(3)	75.4(12)
C14	6283.9(17)	3259(6)	4851(3)	65.0(10)
C15	6205.5(13)	1662(5)	5293(2)	49.8(7)
C16	6736.5(14)	2857(6)	6867(2)	56.5(9)
C17	6994.3(15)	1561(6)	7519(3)	61.9(9)
C18	7519.1(17)	1777(8)	8284(3)	79.5(13)
C19	7780.9(18)	3389(10)	8386(3)	94.8(19)
C20	7552(2)	4684(10)	7768(4)	99.3(19)
C21	7026(2)	4442(7)	7005(3)	79.1(13)
C22	8230(5)	5300(20)	10302(9)	140(4)
C23	6617.8(12)	-818(5)	4978(2)	48.2(7)
C24	6181.0(13)	-917(5)	4098(2)	52.2(8)
C25	6185.8(15)	-2090(5)	3512(2)	57.5(8)
C26	6644.7(16)	-3116(6)	3792(3)	62.9(9)
C27	7090.9(15)	-3010(6)	4669(3)	60.8(9)
C28	7084.8(13)	-1917(5)	5271(2)	51.2(8)
C29	5691(3)	-3137(9)	2014(3)	102.4(19)
C30	7982.8(14)	-2768(6)	6468(3)	61.6(10)
C31	8401.6(15)	-2396(6)	7430(3)	60.4(9)
C32	8318.1(17)	-1221(7)	7922(3)	76.2(12)
C33	8718(2)	-978(9)	8812(3)	91.0(16)
C34	9205(2)	-1874(10)	9224(3)	98.5(17)
C35	9297(2)	-3044(9)	8736(4)	99.3(18)
C36	8901.0(18)	-3290(8)	7861(3)	81.9(13)
C22A	8287(12)	3770(30)	10457(19)	140(4)

Table S36 Anisotropic Displacement Parameters (Å^2×10^3) for 13m. The Anisotropic displacement factor exponent takes the form: -2π^2[h^{2}a*^{2}U$_{11}$+2hka*b*U$_{12}$+…].

Atom	U_{11}	U_{22}	U_{33}	U_{23}	U_{13}	U_{12}
S1	84.0(12)	209(4)	76.1(13)	-36.7(18)	10.5(10)	-3(2)
S1A	84.0(12)	209(4)	76.1(13)	-36.7(18)	10.5(10)	-3(2)
F1	182(5)	253(11)	121(4)	-85(7)	85(4)	-26(8)
F2	122(4)	357(15)	174(7)	-104(10)	70(5)	21(8)
F3	278(12)	216(10)	285(13)	15(9)	177(10)	29(9)
F1A	182(5)	253(11)	121(4)	-85(7)	85(4)	-26(8)
F2A	122(4)	357(15)	174(7)	-104(10)	70(5)	21(8)
F3A	278(12)	216(10)	285(13)	15(9)	177(10)	29(9)
O1	70.8(16)	67.2(19)	65.2(15)	-13.4(14)	32.5(13)	1.8(14)

O2	47.4(11)	65.3(18)	69.5(14)	-5.0(13)	32.2(11)	9.4(11)
O3	51(5)	159(12)	87(5)	-39(6)	18(4)	-11(4)
O4	201(11)	234(11)	152(7)	-9(8)	20(7)	-78(10)
O5	93(3)	254(13)	112(7)	-63(7)	5(4)	-1(6)
O3A	51(5)	159(12)	87(5)	-39(6)	18(4)	-11(4)
O4A	201(11)	234(11)	152(7)	-9(8)	20(7)	-78(10)
O5A	93(3)	254(13)	112(7)	-63(7)	5(4)	-1(6)
N1	46.0(14)	60(2)	57.1(15)	-4.8(14)	29.5(13)	7.8(13)
C1	67(2)	85(3)	68(2)	16(2)	47.9(18)	11(2)
C2	94(3)	93(4)	78(3)	2(2)	65(2)	13(3)
C3	99(3)	74(3)	76(2)	-10(2)	59(2)	3(2)
C4	73(2)	57(2)	71(2)	0.2(18)	48.6(18)	0.5(18)
C5	46.7(15)	52(2)	51.7(15)	4.6(14)	28.0(13)	6.8(13)
C6	46.0(15)	62(2)	59.2(18)	13.7(16)	31.9(14)	9.4(15)
C7	48.4(16)	56(2)	74(2)	15.2(18)	34.9(16)	4.1(15)
C8	55.1(18)	49(2)	85(2)	0.2(18)	41.4(18)	-2.1(15)
C9	50.1(16)	48(2)	70(2)	-4.7(16)	37.0(16)	-0.4(14)
C10	38.3(13)	46.6(19)	54.9(15)	1.1(14)	25.9(12)	3.0(12)
C11	48.5(15)	46(2)	60.8(17)	-4.5(15)	36.1(14)	-1.2(14)
C12	72(2)	50(2)	84(2)	6.9(19)	54(2)	9.4(18)
C13	108(3)	54(3)	84(3)	12(2)	66(3)	15(2)
C14	76(2)	65(3)	74(2)	-1.3(19)	53.9(19)	-3.6(19)
C15	47.9(15)	50(2)	57.3(16)	-2.1(15)	33.1(13)	3.9(13)
C16	54.8(17)	62(2)	63.9(19)	-13.5(17)	40.5(16)	-10.1(16)
C17	53.4(18)	66(3)	67(2)	-11.0(19)	33.8(16)	-5.5(17)
C18	58(2)	104(4)	67(2)	-16(2)	29.6(18)	2(2)
C19	58(2)	149(6)	77(3)	-49(3)	37(2)	-34(3)
C20	96(3)	117(5)	94(3)	-40(3)	59(3)	-62(4)
C21	86(3)	76(3)	84(3)	-21(2)	54(2)	-34(2)
C22	116(5)	175(9)	121(6)	-33(7)	62(5)	10(6)
C23	46.9(15)	45.4(19)	62.9(17)	-1.1(15)	37.4(14)	-2.7(13)
C24	47.7(15)	49(2)	65.2(18)	-0.6(15)	35.2(15)	2.1(14)
C25	61.7(19)	51(2)	65.7(19)	-5.4(17)	39.5(16)	-5.3(16)
C26	69(2)	54(2)	68(2)	-12.4(18)	41.0(18)	2.4(18)
C27	58.3(18)	52(2)	79(2)	-3.4(18)	43.4(18)	8.3(16)
C28	47.9(15)	49(2)	62.8(18)	-1.1(15)	34.8(14)	-1.5(13)
C29	100(4)	96(4)	78(3)	-30(3)	30(3)	9(3)
C30	50.3(17)	62(3)	76(2)	0.7(18)	37.4(17)	9.3(16)
C31	51.9(17)	59(2)	75(2)	6.0(18)	38.3(17)	2.7(16)
C32	61(2)	90(4)	76(2)	2(2)	37.5(19)	11(2)

C33	81(3)	114(5)	75(3)	-4(3)	42(2)	12(3)
C34	75(3)	121(5)	73(3)	6(3)	26(2)	9(3)
C35	72(3)	105(5)	91(3)	5(3)	28(3)	28(3)
C36	60(2)	83(3)	88(3)	-7(3)	34(2)	12(2)
C22A	116(5)	175(9)	121(6)	-33(7)	62(5)	10(6)

Table S37 Bond Lengths for 13m.

Atom	Atom	Length/Å	Atom	Atom	Length/Å
S1	O3	1.476(14)	C7	C8	1.350(6)
S1	O4	1.591(14)	C8	C9	1.413(5)
S1	O5	1.380(9)	C9	C10	1.369(5)
S1	C22	1.731(13)	C10	C11	1.532(4)
S1A	O3A	1.47(4)	C11	C12	1.568(5)
S1A	O4A	1.574(17)	C11	C15	1.580(4)
S1A	O5A	1.534(18)	C11	C16	1.527(5)
S1A	C22A	1.75(2)	C12	C13	1.533(5)
F1	C22	1.248(14)	C13	C14	1.517(6)
F2	C22	1.343(16)	C14	C15	1.532(5)
F3	C22	1.322(19)	C16	C17	1.385(6)
F1A	C22A	1.24(3)	C16	C21	1.398(6)
F2A	C22A	1.36(4)	C17	C18	1.385(6)
F3A	C22A	1.389(18)	C18	C19	1.387(9)
O1	C25	1.376(5)	C19	C20	1.345(9)
O1	C29	1.418(6)	C20	C21	1.387(7)
O2	C28	1.370(4)	C23	C24	1.380(5)
O2	C30	1.412(4)	C23	C28	1.419(5)
O3	C19	1.496(10)	C24	C25	1.390(5)
O3A	C19	1.33(4)	C25	C26	1.374(5)
N1	C15	1.443(4)	C26	C27	1.386(5)
N1	C23	1.395(4)	C27	C28	1.379(5)
C1	C2	1.343(7)	C30	C31	1.495(6)
C1	C6	1.415(5)	C31	C32	1.376(6)
C2	C3	1.404(7)	C31	C36	1.383(6)
C3	C4	1.371(5)	C32	C33	1.378(6)
C4	C5	1.416(6)	C33	C34	1.358(8)
C5	C6	1.437(5)	C34	C35	1.381(8)
C5	C10	1.446(5)	C35	C36	1.358(7)
C6	C7	1.394(6)			

Table S38 Bond Angles for 13m.

Atom	Atom	Atom	Angle/°	Atom	Atom	Atom	Angle/°
O3	S1	O4	108.8(8)	C17	C16	C11	119.6(3)
O3	S1	C22	111.2(6)	C17	C16	C21	117.4(4)
O4	S1	C22	96.3(9)	C21	C16	C11	123.0(4)
O5	S1	O3	111.1(7)	C16	C17	C18	122.8(5)
O5	S1	O4	119.1(8)	C17	C18	C19	116.9(5)
O5	S1	C22	109.4(7)	O3A	C19	C18	135(2)
O3A	S1A	O4A	145(2)	O3A	C19	C20	102.3(19)
O3A	S1A	O5A	101.4(17)	C18	C19	O3	114.6(8)
O3A	S1A	C22A	106(2)	C20	C19	O3	122.4(8)
O4A	S1A	C22A	99.9(15)	C20	C19	C18	122.7(4)
O5A	S1A	O4A	96.3(13)	C19	C20	C21	119.6(5)
O5A	S1A	C22A	99.6(13)	C20	C21	C16	120.6(5)
C25	O1	C29	117.8(3)	F1	C22	S1	120.7(12)
C28	O2	C30	116.9(3)	F1	C22	F2	105.2(11)
S1	O3	C19	127.4(10)	F1	C22	F3	119.3(15)
C19	O3A	S1A	136(3)	F2	C22	S1	109.6(10)
C23	N1	C15	119.8(3)	F3	C22	S1	104.0(10)
C2	C1	C6	121.4(4)	F3	C22	F2	94.8(13)
C1	C2	C3	120.5(4)	N1	C23	C28	118.8(3)
C4	C3	C2	120.3(5)	C24	C23	N1	123.0(3)
C3	C4	C5	121.3(4)	C24	C23	C28	118.1(3)
C4	C5	C6	117.4(3)	C23	C24	C25	121.6(3)
C4	C5	C10	124.1(3)	O1	C25	C24	114.0(3)
C6	C5	C10	118.4(3)	C26	C25	O1	125.9(3)
C1	C6	C5	119.0(4)	C26	C25	C24	120.1(3)
C7	C6	C1	120.9(4)	C25	C26	C27	119.0(3)
C7	C6	C5	120.1(3)	C28	C27	C26	121.9(3)
C8	C7	C6	120.5(3)	O2	C28	C23	114.8(3)
C7	C8	C9	120.6(4)	O2	C28	C27	126.0(3)
C10	C9	C8	122.1(3)	C27	C28	C23	119.2(3)
C5	C10	C11	120.1(3)	O2	C30	C31	110.4(3)
C9	C10	C5	118.2(3)	C32	C31	C30	123.8(3)
C9	C10	C11	121.7(3)	C32	C31	C36	117.4(4)
C10	C11	C12	111.5(3)	C36	C31	C30	118.8(4)
C10	C11	C15	112.7(3)	C31	C32	C33	120.6(4)
C12	C11	C15	100.2(3)	C34	C33	C32	121.1(5)
C16	C11	C10	109.6(3)	C33	C34	C35	119.0(5)
C16	C11	C12	113.2(3)	C36	C35	C34	119.9(5)
C16	C11	C15	109.5(2)	C35	C36	C31	122.1(5)

C13	C12	C11	109.1(3)	F1A	C22A	S1A	119(2)
C14	C13	C12	105.6(3)	F1A	C22A	F2A	101(2)
C13	C14	C15	103.4(3)	F1A	C22A	F3A	116(3)
N1	C15	C11	114.8(3)	F2A	C22A	S1A	112(3)
N1	C15	C14	115.5(3)	F2A	C22A	F3A	106(3)
C14	C15	C11	104.5(3)	F3A	C22A	S1A	102.4(17)

Table S39 Torsion Angles for 13m.

A	B	C	D	Angle/°	A	B	C	D	Angle/°
S1	O3	C19	C18	-69.9(12)	C10	C5	C6	C1	-178.2(3)
S1	O3	C19	C20	116.7(12)	C10	C5	C6	C7	2.5(5)
S1A	O3A	C19	C18	-82(4)	C10	C11	C12	C13	-136.7(4)
S1A	O3A	C19	C20	96(4)	C10	C11	C15	N1	-77.8(4)
O1	C25	C26	C27	-178.9(4)	C10	C11	C15	C14	154.6(3)
O2	C30	C31	C32	0.5(6)	C10	C11	C16	C17	32.4(4)
O2	C30	C31	C36	179.7(4)	C10	C11	C16	C21	-147.7(3)
O3	S1	C22	F1	-177.5(13)	C11	C12	C13	C14	-7.6(5)
O3	S1	C22	F2	60.2(15)	C11	C16	C17	C18	179.6(3)
O3	S1	C22	F3	-40.2(14)	C11	C16	C21	C20	-179.8(4)
O3	C19	C20	C21	174.7(7)	C12	C11	C15	N1	163.6(3)
O4	S1	O3	C19	50.8(15)	C12	C11	C15	C14	36.0(3)
O4	S1	C22	F1	69.6(14)	C12	C11	C16	C17	157.6(3)
O4	S1	C22	F2	-52.8(12)	C12	C11	C16	C21	-22.5(4)
O4	S1	C22	F3	-153.1(11)	C12	C13	C14	C15	30.5(5)
O5	S1	O3	C19	-176.2(11)	C13	C14	C15	N1	-169.4(3)
O5	S1	C22	F1	-54.4(15)	C13	C14	C15	C11	-42.3(4)
O5	S1	C22	F2	-176.7(11)	C15	N1	C23	C24	-15.5(5)
O5	S1	C22	F3	83.0(12)	C15	N1	C23	C28	166.7(3)
O3A	S1A	C22A	F1A	177(2)	C15	C11	C12	C13	-17.3(4)
O3A	S1A	C22A	F2A	-65(2)	C15	C11	C16	C17	-91.6(4)
O3A	S1A	C22A	F3A	48(3)	C15	C11	C16	C21	88.3(4)
O3A	C19	C20	C21	-176.3(18)	C16	C11	C12	C13	99.2(4)
O4A	S1A	O3A	C19	-84(6)	C16	C11	C15	N1	44.4(4)
O4A	S1A	C22A	F1A	-26(3)	C16	C11	C15	C14	-83.2(3)
O4A	S1A	C22A	F2A	92(2)	C16	C17	C18	C19	1.1(6)
O4A	S1A	C22A	F3A	-155(2)	C17	C16	C21	C20	0.1(6)
O5A	S1A	O3A	C19	156(4)	C17	C18	C19	O3	-175.3(7)
O5A	S1A	C22A	F1A	72(3)	C17	C18	C19	O3A	175(2)
O5A	S1A	C22A	F2A	-170(2)	C17	C18	C19	C20	-1.9(7)
O5A	S1A	C22A	F3A	-57(3)	C18	C19	C20	C21	1.8(8)

N1	C23	C24	C25	-176.0(3)	C19	C20	C21	C16	-0.8(8)
N1	C23	C28	O2	-2.4(4)	C21	C16	C17	C18	-0.3(6)
N1	C23	C28	C27	179.1(3)	C22	S1	O3	C19	-54.0(15)
C1	C2	C3	C4	1.2(7)	C23	N1	C15	C11	167.5(3)
C1	C6	C7	C8	179.4(4)	C23	N1	C15	C14	-70.7(4)
C2	C1	C6	C5	-0.4(6)	C23	C24	C25	O1	177.6(3)
C2	C1	C6	C7	178.9(4)	C23	C24	C25	C26	-3.6(6)
C2	C3	C4	C5	1.5(7)	C24	C23	C28	O2	179.8(3)
C3	C4	C5	C6	-3.5(5)	C24	C23	C28	C27	1.2(5)
C3	C4	C5	C10	177.7(4)	C24	C25	C26	C27	2.4(6)
C4	C5	C6	C1	2.9(5)	C25	C26	C27	C28	0.6(6)
C4	C5	C6	C7	-176.4(3)	C26	C27	C28	O2	179.2(4)
C4	C5	C10	C9	177.2(3)	C26	C27	C28	C23	-2.4(6)
C4	C5	C10	C11	-1.7(5)	C28	O2	C30	C31	178.7(3)
C5	C6	C7	C8	-1.3(5)	C28	C23	C24	C25	1.7(5)
C5	C10	C11	C12	-58.9(4)	C29	O1	C25	C24	176.1(4)
C5	C10	C11	C15	-170.6(3)	C29	O1	C25	C26	-2.7(7)
C5	C10	C11	C16	67.2(4)	C30	O2	C28	C23	-178.2(3)
C6	C1	C2	C3	-1.7(7)	C30	O2	C28	C27	0.2(5)
C6	C5	C10	C9	-1.5(4)	C30	C31	C32	C33	178.3(5)
C6	C5	C10	C11	179.5(3)	C30	C31	C36	C35	-178.8(5)
C6	C7	C8	C9	-0.9(6)	C31	C32	C33	C34	0.6(9)
C7	C8	C9	C10	1.9(6)	C32	C31	C36	C35	0.5(8)
C8	C9	C10	C5	-0.6(5)	C32	C33	C34	C35	0.1(10)
C8	C9	C10	C11	178.3(3)	C33	C34	C35	C36	-0.5(11)
C9	C10	C11	C12	122.2(3)	C34	C35	C36	C31	0.3(10)
C9	C10	C11	C15	10.4(4)	C36	C31	C32	C33	-0.9(7)
C9	C10	C11	C16	-111.7(4)	C22A	S1A	O3A	C19	53(4)

Table S40 Hydrogen Atom Coordinates (Å×10^4) and Isotropic Displacement Parameters (Å^2×10^3) for 13m.

**Atom**	***x***	***y***	***z***	**U(eq)**
H1	6923(18)	510(60)	6080(30)	58(11)
H1A	5003	820	7724	82
H2	5205	3641	8253	95
H3	5734	5481	7983	93
H4	6036	4484	7141	75
H7	5056	-1605	6881	71
H8	5325	-2665	6016	74
H9	5787	-834	5609	65

H12A	5450	4141	5351	76
H12B	5991	5262	5970	76
H13A	5519	4480	4216	90
H13B	6008	5825	4801	90
H14A	6659	3707	5215	78
H14B	6199	2940	4272	78
H15	5846	1133	4851	60
H17	6807	500	7439	74
H18	7688	882	8710	95
H20	7744	5735	7853	119
H21	6866	5342	6581	95
H24	5876	-182	3892	63
H26	6656	-3870	3398	75
H27	7403	-3695	4858	73
H29A	5348	-2939	1452	154
H29B	5720	-4372	2174	154
H29C	5993	-2824	1972	154
H30A	8137	-2485	6132	74
H30B	7888	-4026	6387	74
H32	7989	-585	7652	91
H33	8653	-187	9136	109
H34	9472	-1704	9826	118
H35	9629	-3661	9007	119
H36	8968	-4085	7540	98

Table S41 Atomic Occupancy for 13m.

**Atom**	***Occupancy***	**Atom**	***Occupancy***	**Atom**	***Occupancy***
S1	0.718(4)	S1A	0.282(4)	F1	0.718(4)
F2	0.718(4)	F3	0.718(4)	F1A	0.282(4)
F2A	0.282(4)	F3A	0.282(4)	O3	0.718(4)
O4	0.718(4)	O5	0.718(4)	O3A	0.282(4)
O4A	0.282(4)	O5A	0.282(4)	C22	0.718(4)
C22A	0.282(4)				

7. Characterization of adducts

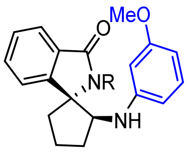

3e: white solid; Mp 76.5 - 77.7 °C; 49.0 mg, 95% yield; 94% ee; dr > 19:1; $[\alpha]_D^{22}$ +104.8 (*c* 1.0, $CHCl_3$); ^1H NMR (300 MHz, $CDCl_3$) δ 8.15 (d, *J* = 8.2 Hz, 2H), 7.80 (d, *J* = 7.7 Hz, 1H), 7.74 (t, *J* = 7.5 Hz, 1H), 7.86 – 7.44 (m, 4H), 6.99 (t, *J* = 8.1 Hz, 1H), 6.27 (d, *J* = 8.1 Hz, 1H), 6.01 (d, *J* = 8.1 Hz, 1H), 5.95 (s, 1H), 5.16 (dd, *J* = 10.7, 7.8 Hz, 1H), 3.70 (s, 3H), 3.21 – 3.07 (m, 1H), 2.76 – 2.61 (m, 1H), 2.40 – 2.22 (m, 2H), 2.16 – 2.06 (m, 1H), 1.86 – 1.74 (m, 1H); ^{13}C NMR (75 MHz, $CDCl_3$) δ 166.7, 160.8, 148.4, 147.8, 142.5, 135.1 (q, *J* = 33.2 Hz), 134.7, 130.1, 129.4, 129.1, 126.7 (q, *J* = 273.6 Hz), 125.8 (q, *J* = 3.5 Hz), 125.2, 122.6, 106.6, 103.6, 99.8, 79.1, 59.8, 55.1, 34.8, 31.0, 19.9; ^{19}F NMR (376 MHz, $CDCl_3$) δ –63.3; HRMS (ESI) m/z 539.1224 (M+Na$^+$), calc. for $C_{26}H_{23}F_3N_2O_4SNa^+$ 539.1223.

The ee was determined by HPLC analysis: CHIRALPAK IA (4.6 mm i.d. x 250 mm); Hexane/2-propanol = 80/20; flow rate: 1.0 mL/min; 25 °C; 210 nm; retention time: 14.0 min (minor) and 26.9 min (major).

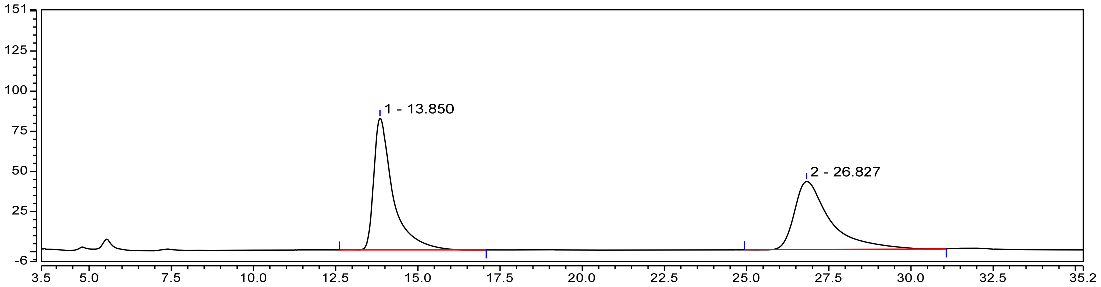

Entry	Retention Time	Area	Height	%Area
1	13.850	55.4073	82.06	50.95
2	26.827	53.3420	42.46	49.05

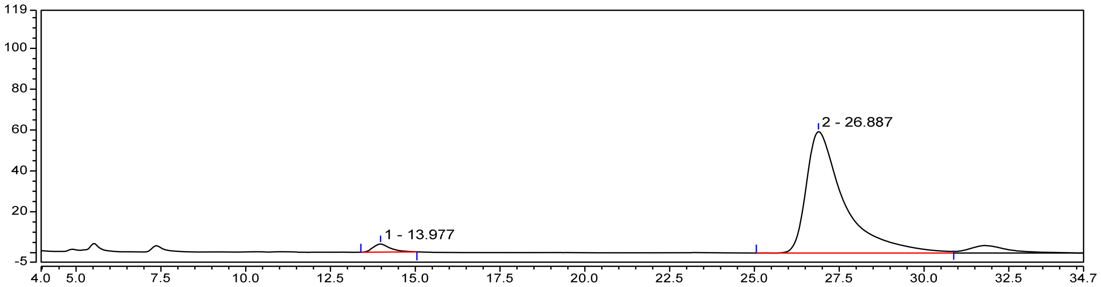

Entry	Retention Time	Area	Height	%Area
1	13.977	2.3475	3.95	2.95
2	26.887	77.2458	59.41	97.05

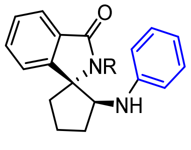

R = 4-$CF_3C_6H_4SO_2$

3f: white solid; Mp 93.5 - 94.3 °C; 45.2 mg, 93% yield; 90% ee; dr > 19:1; $[\alpha]_D^{22}$ –38.5 (*c* 1.0, $CHCl_3$); ^{1}H NMR (300 MHz, $CDCl_3$) δ 8.12 (d, *J* = 8.2 Hz, 2H), 7.83 – 7.67 (m, 2H), 7.60 – 7.38 (m, 4H), 7.09 (t, *J* = 7.8 Hz, 2H), 6.71 (t, *J* = 7.3 Hz, 1H), 6.41 (d, *J* = 7.9 Hz, 2H), 5.20 (dd, *J* = 10.9, 7.7 Hz, 1H), 3.21 – 3.06 (m, 1H), 2.74 – 2.62 (m, 1H), 2.40 – 2.20 (m, 2H), 2.18 – 2.04 (m, 1H), 1.87 – 1.72 (m, 1H); ^{13}C NMR (75 MHz, $CDCl_3$) δ 166.6, 148.4, 146.3, 142.4, 135.1 (q, *J* = 33.2 Hz), 134.7, 129.4, 129.3, 129.0, 128.2, 125.7 (q, *J* = 3.6 Hz, 1H), 125.7, 125.6, 125.1, 123.0 (q, *J* = 273.2 Hz), 122.6, 118.4, 113.6, 79.1, 59.6, 34.8, 30.9, 19.8; ^{19}F NMR (376 MHz, $CDCl_3$) δ –63.4; HRMS (ESI) m/z 509.1115 (M+Na$^+$), calc. for $C_{25}H_{21}F_3N_2O_3SNa^+$ 509.1117.

The ee was determined by HPLC analysis: CHIRAL INB (4.6 mm i.d. x 250 mm); Hexane/2-propanol = 85/15; flow rate: 1.0 mL/min; 25 °C; 254 nm; retention time: 14.4 min (major) and 18.7 min (minor).

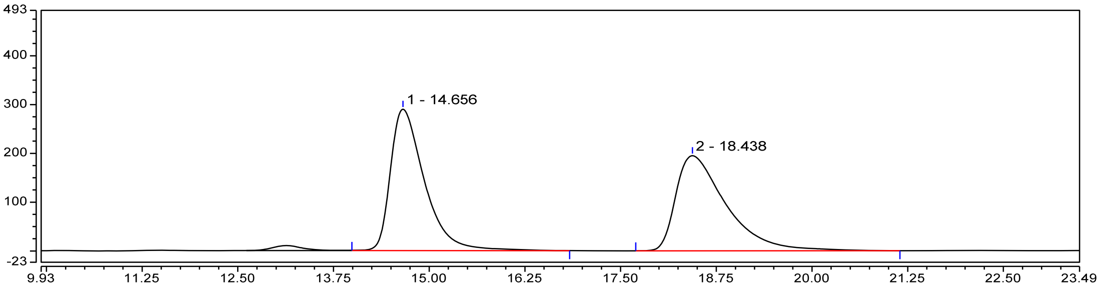

Entry	Retention Time	Area	Height	%Area
1	14.656	149.3852	289.67	50.17
2	18.438	148.3553	194.88	49.83

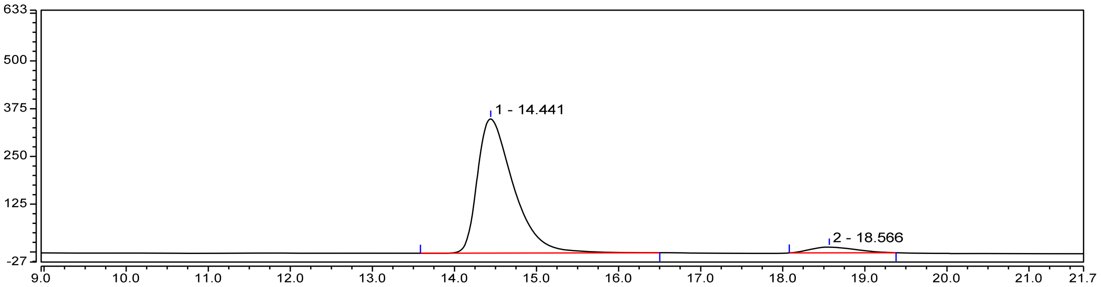

Entry	Retention Time	Area	Height	%Area
1	14.441	178.5643	351.18	94.89
2	18.566	9.6174	15.21	5.11

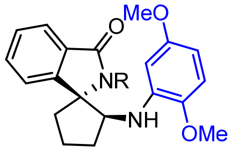

R = 4-$CF_3C_6H_4SO_2$

3g: white solid; Mp 75.0 – 76.1 °C; 43.7 mg, 80% yield; 94% ee; dr > 19:1; $[\alpha]_D^{22}$ +41.9 (*c* 1.0, $CHCl_3$); ^1H NMR (300 MHz, Chloroform-*d*) δ 8.15 (d, *J* = 8.3 Hz, 2H), 7.78 (d, *J* = 7.6 Hz, 1H), 7.72 (td, *J* = 7.6, 1.2 Hz, 1H), 7.58 – 7.47 (m, 4H), 6.45 (d, *J* = 8.6 Hz, 1H), 6.31 (d, *J* = 2.8 Hz, 1H), 6.11 (dd, *J* = 8.6, 2.8 Hz, 1H), 5.13 – 4.99 (m, 1H), 3.75 (s, 3H), 3.39 (d, *J* = 9.4 Hz, 1H), 3.31 (s, 3H), 3.20 – 3.08 (m, 1H), 2.74 – 2.61 (m, 1H), 2.41 – 2.24 (m, 2H), 2.17 – 2.06 (m, 1H), 1.90 – 1.76 (m, 1H); ^{13}C NMR (75 MHz, $CDCl_3$) δ 166.7, 154.8, 148.6, 142.5, 141.2, 137.6, δ 134.9 (q, *J* = 33.0 Hz), 134.5, 129.2, 128.9, 128.0, δ 125.6 (q, *J* = 3.5 Hz), 124.8, 122.7, 110.7, 99.9, 98.9, 79.2, 59.9, 56.0, 55.5, 35.1, 31.5, 20.2; ^{19}F NMR (376 MHz, CD_2Cl_2) δ –63.6; HRMS (ESI) m/z 547.1505 (M+H$^+$), calc. for $C_{27}H_{26}F_3N_2O_5S^+$ 547.1509.

The ee was determined by HPLC analysis: CHIRAL MQ (x 2) (4.6 mm i.d. x 250 mm); Hexane/2-propanol = 50/50; flow rate: 1.0 mL/min; 25 °C; 254 nm; retention time: 9.8 min (minor) and 17.2 min (major).

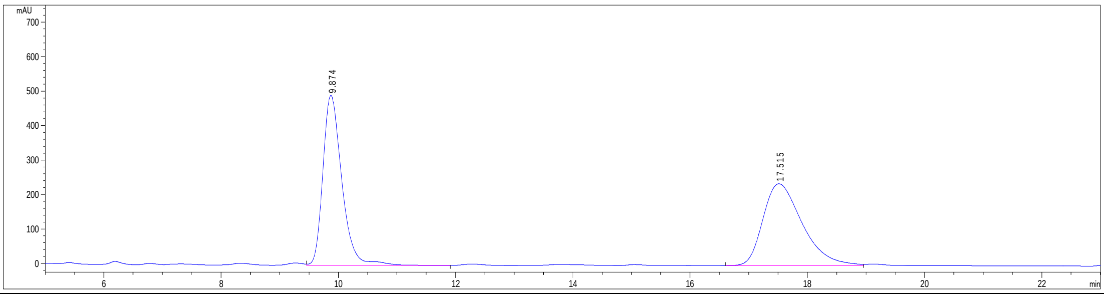

Entry	Retention Time	Area	Height	%Area
1	9.874	11067.4	492.2	50.043
2	17.515	11048.5	237.3	49.957

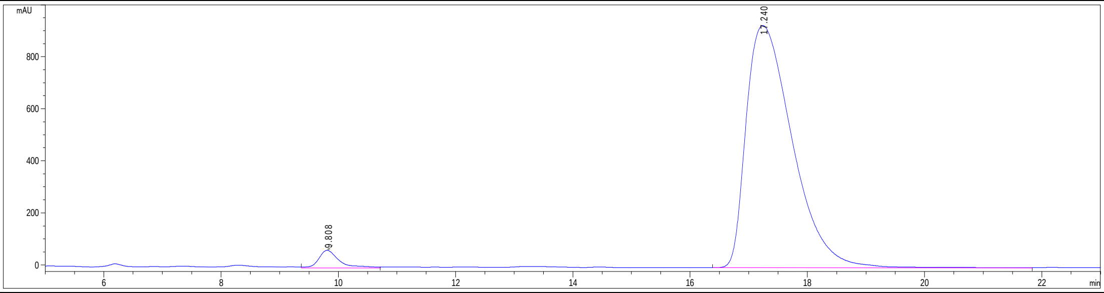

Entry	Retention Time	Area	Height	%Area
1	9.808	1666.1	67.8	3.158
2	17.24	51092.4	931.2	96.842

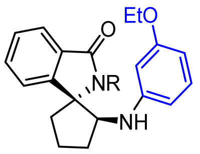

R = 4-$CF_3C_6H_4SO_2$

3h: white solid; Mp 76.3 – 77.2; 44.0 mg, 83% yield; 90% ee; dr > 19:1; $[\alpha]_D^{22}$ +23.2 (*c* 1.0, $CHCl_3$); ^{1}H NMR (300 MHz, Chloroform-*d*) δ 8.14 (d, *J* = 8.2 Hz, 2H), 7.87 – 7.78 (m, 1H), 7.73 (t, *J* = 7.5 Hz, 1H), 7.53 (dt, *J* = 10.6, 6.5 Hz, 4H), 6.98 (t, *J* = 8.1 Hz, 1H), 6.26 (dd, *J* = 8.2, 2.2 Hz, 1H), 6.07 – 5.91 (m, 2H), 5.15 (dd, *J* = 11.0, 7.6 Hz, 1H), 3.93 (q, *J* = 7.0 Hz, 2H), 3.24 – 3.06 (m, 1H), 2.74 – 2.65 (m, 1H), 2.40 – 2.21 (m, 2H), 2.18 – 2.06 (m, 1H), 1.92 – 1.72 (m, 1H), 1.35 (t, *J* = 7.0 Hz, 3H); ^{13}C NMR (75 MHz, $CDCl_3$) δ 166.6, 160.2, 148.4, 147.8, 142.4, δ 135.1 (q, *J* = 32.9 Hz), 134.7, 130.0, 129.4, 129.0, 128.2, δ 125.7 (q, *J* = 3.7 Hz), 125.1, δ 123.1 (q, *J* = 273.2 Hz), 122.6, 106.4, 104.0, 100.3, 79.0, 63.2, 59.6, 34.7, 30.8, 19.8, 14.8; ^{19}F NMR (376 MHz, $CDCl_3$) δ –63.3; HRMS (ESI) m/z 531.1555 (M+H$^+$), calc. for $C_{27}H_{26}F_3N_2O_4S^+$ 531.1560.

The ee was determined by HPLC analysis: CHIRALPAK IC (4.6 mm i.d. x 250 mm); Hexane/2-propanol = 70/30; flow rate: 1.0 mL/min; 25 °C; 210 nm; retention time: 12.2 min (minor) and 14.7 min (major).

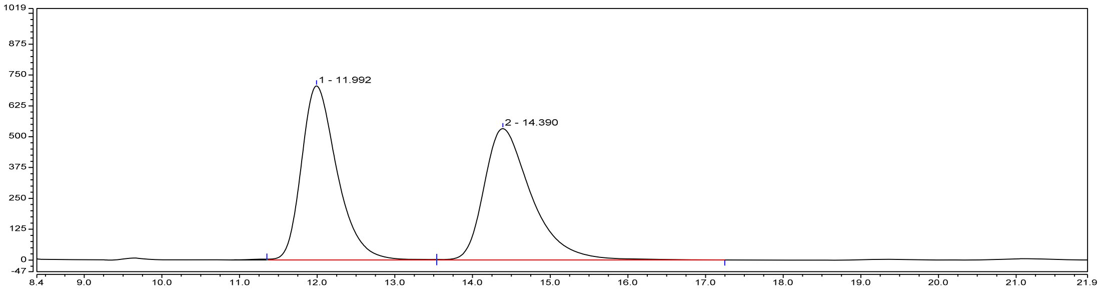

Entry	Retention Time	Area	Height	%Area
1	11.992	374.3872	704.87	49.93
2	14.390	375.4092	531.92	50.07

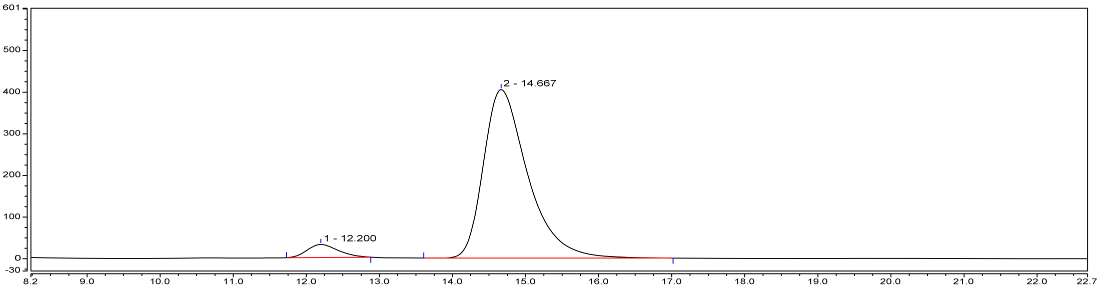

Entry	Retention Time	Area	Height	%Area
1	12.200	15.3530	31.20	5.13
2	14.667	283.8473	404.57	94.87

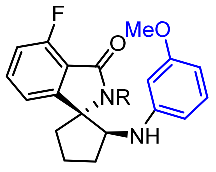

R = 4-$CF_3C_6H_4SO_2$

3i: white solid; Mp 94.7 - 95.7 °C; 47.0 mg, 88% yield; 95% ee; dr > 19:1; $[\alpha]_D^{22}$ +43.5 (*c* 1.0, $CHCl_3$); ^{1}H NMR (300 MHz, $CDCl_3$) δ 8.16 (d, *J* = 8.3 Hz, 2H), 7.71 (td, *J* = 8.0, 4.9 Hz, 1H), 7.54 (d, *J* = 8.3 Hz, 2H), 7.31 (d, *J* = 7.8 Hz, 1H), 7.15 (t, *J* = 8.6 Hz, 1H), 7.00 (t, *J* = 8.1 Hz, 1H), 6.29 (d, *J* = 8.1 Hz, 1H), 6.08 – 5.94 (m, 2H), 5.17 (dd, *J* = 10.9, 7.7 Hz, 1H), 3.71 (s, 3H), 3.16 – 3.05 (m, 1H), 2.68 (dt, *J* = 12.9, 6.4 Hz, 1H), 2.29 (dt, *J* = 15.1, 10.6 Hz, 2H), 2.09 (d, *J* = 11.4 Hz, 1H), 1.81 (dd, *J* = 21.4, 11.1 Hz, 1H); ^{13}C NMR (75 MHz, $CDCl_3$) δ163.4 (d, *J* = 2.6 Hz), 160.9, 159.5 (d, *J* = 265.8 Hz), 151.1, 142.2, 136.7 (d, *J* = 8.2 Hz), 135.3 (q, *J* = 33.1 Hz), 130.2, 129.3, 125.8 (q, *J* = 3.7 Hz), 123.1 (q, *J* = 273.2 Hz), 118.6 (d, *J* = 4.4 Hz), 116.6 (d, *J* = 18.7 Hz), 116.1 (d, *J* = 12.5 Hz), 106.8, 104.0, 100.0, 78.8, 60.4, 55.2, 35.4, 31.3, 20.1; ^{19}F NMR (376 MHz, CD_2Cl_2) δ –63.6, –115.8; HRMS (ESI) m/z 557.1127 ($M+Na^+$), calc. for $C_{26}H_{22}F_4N_2O_4SNa^+$ 557.1129.

The ee was determined by HPLC analysis: CHIRALPAK IA (4.6 mm i.d. x 250 mm); Hexane/2-propanol = 80/20; flow rate: 1.0 mL/min; 25 °C; 254 nm; retention time: 15.8 min (minor) and 25.5 min (major).

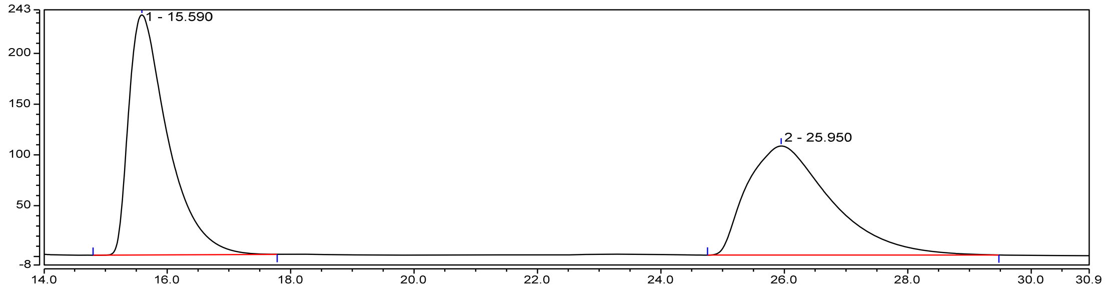

Entry	Retention Time	Area	Height	%Area
1	15.590	173.7018	236.63	50.58
2	25.950	169.6993	107.49	49.42

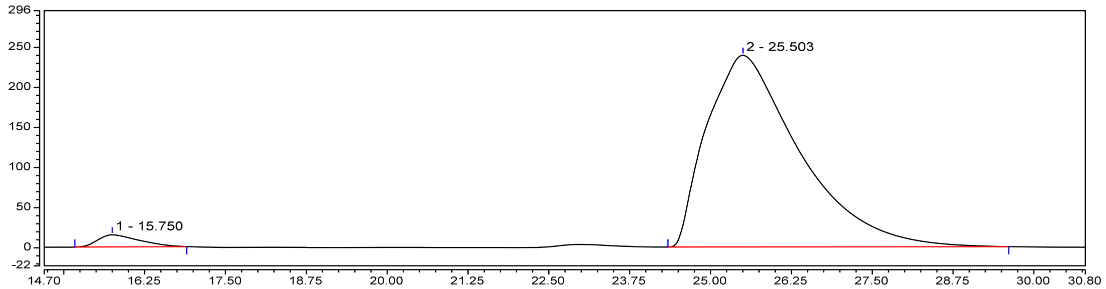

Entry	Retention Time	Area	Height	%Area
1	15.750	11.2075	15.10	2.75
2	25.503	397.0699	238.95	97.25

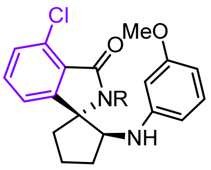

R = 4-$CF_3C_6H_4SO_2$

3j: white solid; Mp 131.5 - 132.7 °C; 53.4 mg, 97% yield; 93% ee; dr > 19:1; $[\alpha]_D^{22}$ +37.7 (*c* 1.0, $CHCl_3$); ^{1}H NMR (300 MHz, $CDCl_3$) δ 8.17 (d, *J* = 8.2 Hz, 2H), 7.67 7.51 (m, 3H), 7.44 (t, *J* = 8.3 Hz, 2H), 6.99 (t, *J* = 8.1 Hz, 1H), 6.28 (d, *J* = 8.3 Hz, 1H), 6.05 (d, *J* = 8.1 Hz, 1H), 5.99 (s, 1H), 5.24 – 5.12 (m, 1H), 3.71 (s, 3H), 3.18 – 3.04 (m, 1H), 2.74 – 2.64 (m, 1H), 2.40 – 2.21 (m, 2H), 2.14 – 2.03 (m, 1H), 1.87 – 1.73 (m, 1H); ^{13}C NMR (75 MHz, $CDCl_3$) δ 164.1, 160.8, 151.1, 147.6, 142.2, 135.2 (q, *J* = 33.5 Hz), 135.0, 133.3, 131.0, 130.1, 129.2, 125.8 (q, *J* = 3.6 Hz), 124.4, 123.0 (q, *J* = 273.2 Hz), 121.0, 106.7, 104.0, 100.0, 77.6, 60.4, 55.1, 35.5, 31.5, 20.2; ^{19}F NMR (376 MHz, CD_2Cl_2) δ –63.6; HRMS (ESI) m/z 573.0832 (M+Na^+), calc. for $C_{26}H_{22}ClF_3N_2O_4SNa^+$ 573.0833.

The ee was determined by HPLC analysis: CHIRALPAK IA (4.6 mm i.d. x 250 mm); Hexane/2-propanol = 80/20; flow rate: 1.0 mL/min; 25 °C; 254 nm; retention time: 16.7 min (minor) and 26.9 min (major).

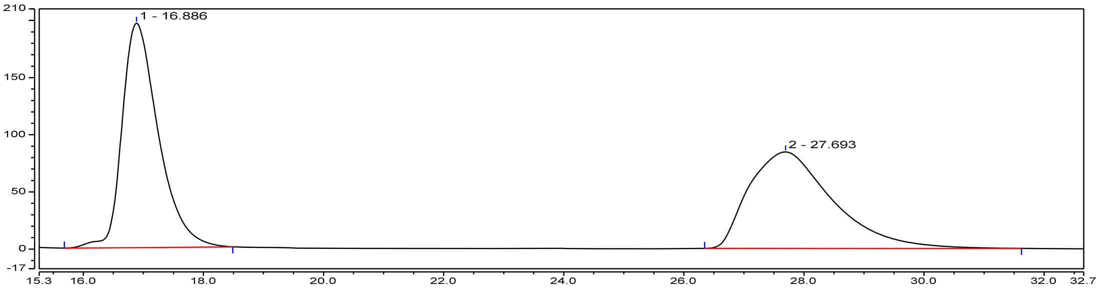

Entry	Retention Time	Area	Height	%Area
1	16.886	137.6585	196.19	50.28
2	27.693	136.1017	84.44	49.72

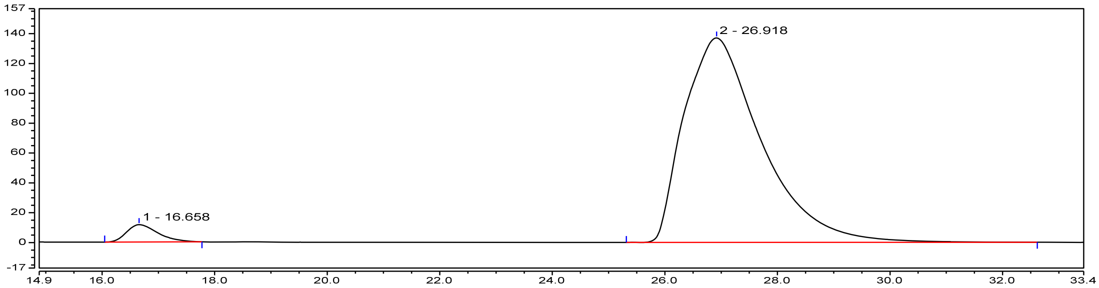

Entry	Retention Time	Area	Height	%Area
1	16.658	7.4436	11.63	3.31
2	26.918	217.5616	136.99	96.69

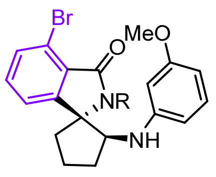

R = 4-$CF_3C_6H_4SO_2$

3k: white solid; Mp 78.1 - 79.7 °C; 54.0 mg, 91% yield; 95% ee; dr > 19:1; $[\alpha]_D^{22}$ +19.3 (*c* 1.0, $CHCl_3$); ^{1}H NMR (300 MHz, $CDCl_3$) δ 8.17 (d, *J* = 8.3 Hz, 2H), 7.66 (d, *J* = 7.6 Hz, 1H), 7.59 – 7.45 (m, 4H), 7.00 (t, *J* = 8.1 Hz, 1H), 6.29 (dd, *J* = 8.2, 2.1 Hz, 1H), 6.09 – 5.96 (m, 2H), 5.18 (dd, *J* = 11.0, 7.6 Hz, 1H), 3.71 (s, 3H), 3.16 – 3.03 (m, 1H), 2.69 (dt, *J* = 13.2, 6.6 Hz, 1H), 2.39 – 2.20 (m, 2H), 2.16 – 2.02 (m, 1H), 1.85 – 1.70 (m, 1H); ^{13}C NMR (75 MHz, $CDCl_3$) δ 160.8, 151.3, 142.2, 135.2 (q, *J* = 33.1 Hz), 135.0, 134.4, 130.1, 129.2, 125.9 (q, *J* = 3.6 Hz), 123.1 (q, *J* = 273.3 Hz), 121.6, 120.8, 106.8, 104.0, 100.1 (q, *J* = 12.6 Hz), 77.3, 60.5, 55.1, 35.5, 31.4, 20.2; ^{19}F NMR (376 MHz, CD_2Cl_2) δ –63.6; HRMS (ESI) m/z 617.0325 (M+Na$^+$), calc. for $C_{26}H_{22}BrF_3N_2O_4SNa^+$ 617.0328.

The ee was determined by HPLC analysis: CHIRALPAK IA (4.6 mm i.d. x 250 mm); Hexane/2-propanol = 80/20; flow rate: 1.0 mL/min; 25 °C; 254 nm; retention time: 19.9 min (minor) and 29.1 min (major).

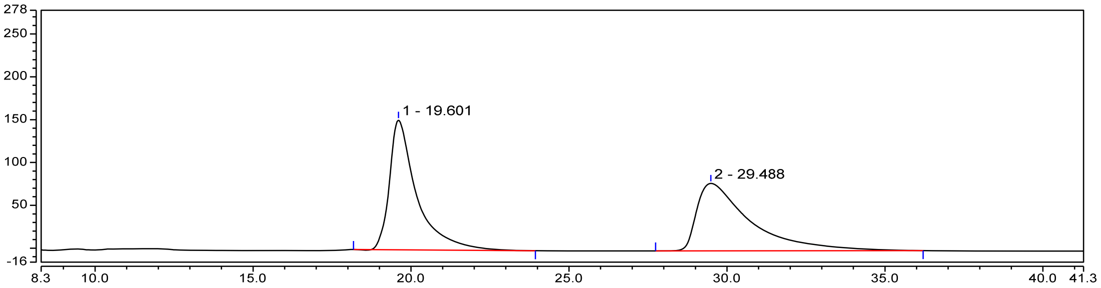

Entry	Retention Time	Area	Height	%Area
1	19.601	151.6313	151.25	49.48
2	29.488	154.8043	78.81	50.52

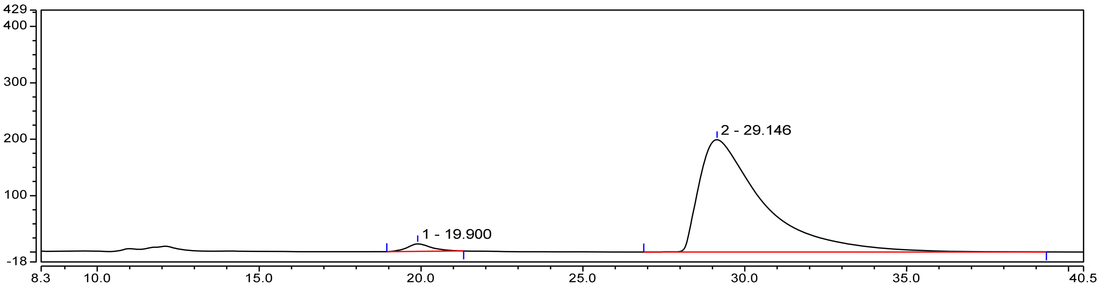

Entry	Retention Time	Area	Height	%Area
1	19.900	11.6405	13.31	2.50
2	29.146	453.6229	198.79	97.50

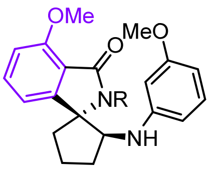

R = 4-$CF_3C_6H_4SO_2$

3l: white solid; Mp 129.5 - 130.7 °C; 48.0 mg, 88% yield; 94% ee; dr > 19:1; $[\alpha]_D^{22}$ +25.0 (*c* 1.0, $CHCl_3$); ^{1}H NMR (300 MHz, $CDCl_3$) δ 8.12 (d, *J* = 8.3 Hz, 2H), 7.65 (t, *J* = 8.0 Hz, 1H), 7.48 (d, *J* = 8.3 Hz, 2H), 7.10 6.90 (m, 3H), 6.27 (dd, *J* = 8.2, 1.7 Hz, 1H), 6.04 (d, *J* = 8.0 Hz, 1H), 5.98 (s, 1H), 5.14 (dd, *J* = 10.9, 7.7 Hz, 1H), 3.92 (s, 3H), 3.71 (s, 3H), 3.20 – 3.05 (m, 1H), 2.70 – 2.59 (m, 6.3 Hz, 1H), 2.36 – 2.19 (m, 2H), 2.12 – 2.01 (m, 1H), 1.86 – 1.72 (m, 1H); ^{13}C NMR (75 MHz, $CDCl_3$) δ 165.1, 160.9, 158.6, 151.3, 148.0, 142.6, 136.4, 134.8 (q, *J* = 32.8 Hz), 130.1, 129.2, 125.7 (q, *J* = 3.7 Hz), 123.1 (q, *J* = 272.9 Hz), 115.5, 114.2, 111.2, 106.5, 103.5, 99.8, 77.8, 60.0, 56.1, 55.1, 35.2, 31.0, 19.9; ^{19}F NMR (376 MHz, $CDCl_3$) δ –63.4; HRMS (ESI) m/z 569.1328 (M+Na$^+$), calc. for $C_{27}H_{25}F_3N_2O_5SNa^+$ 569.1328.

The ee was determined by HPLC analysis: CHIRALPAK IF (4.6 mm i.d. x 250 mm); Hexane/2-propanol = 60/40; flow rate: 1.0 mL/min; 25 °C; 254 nm; retention time: 7.3 min (minor) and 16.7 min (major).

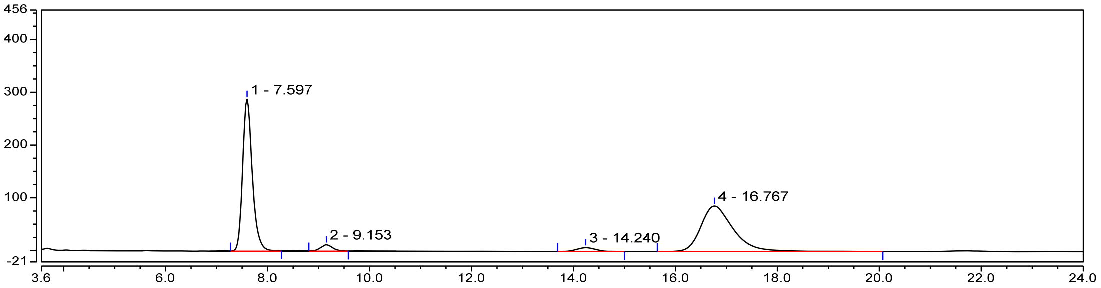

Entry	Retention Time	Area	Height	%Area
1	7.597	64.0107	287.97	47.58
2	9.153	3.4743	12.28	2.58
3	14.240	3.1777	7.46	2.36
4	16.767	63.8573	86.27	47.47

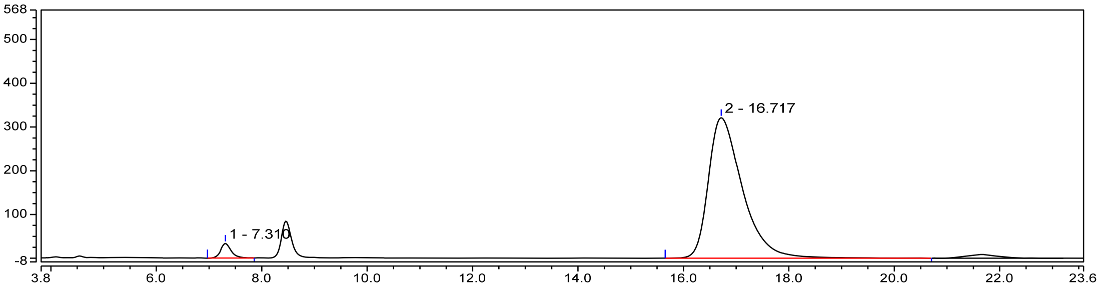

Entry	Retention Time	Area	Height	%Area
1	7.310	7.7192	33.16	3.11
2	16.717	240.1969	320.95	96.89

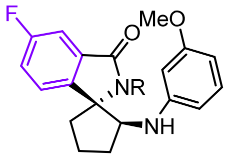

R = 4-$CF_3C_6H_4SO_2$

3m: white solid; Mp 58.1 - 59.3 °C; 49.7 mg, 93% yield; 93% ee; dr > 19:1; $[\alpha]_D^{22}$ +66.6 (*c* 1.0, $CHCl_3$); ^{1}H NMR (300 MHz, $CDCl_3$) δ 8.15 (d, *J* = 8.3 Hz, 2H), 7.59 – 7.47 (m, 3H), 7.47 – 7.37 (m, 2H), 6.99 (t, *J* = 8.1 Hz, 1H), 6.28 (dd, *J* = 8.2, 1.7 Hz, 1H), 6.02 (d, *J* = 8.1 Hz, 1H), 5.96 (s, 1H), 5.14 (dd, *J* = 10.8, 7.7 Hz, 1H), 3.71 (s, 3H), 3.21 – 3.05 (m, 1H), 2.76 – 2.65 (m, 1H), 2.37 – 2.18 (m, 2H), 2.15 – 2.04 (m, 1H), 1.83 – 1.69 (m, 1H); ^{13}C NMR (75 MHz, $CDCl_3$) δ 165.6 (d, *J* = 3.6 Hz), 163.0 (d, *J* = 251.4 Hz), 147.6, 144.0 (d, *J* = 2.6 Hz), 142.2, 135.3 (q, *J* = 33.4 Hz), 130.4 (d, *J* = 8.7 Hz), 130.2, 129.1, 125.8 (q, *J* = 3.5 Hz), 124.5 (d, *J* = 8.2 Hz), 123.0 (q, *J* = 273.2 Hz), 122.4 (d, *J* = 23.6 Hz), 111.6 (d, *J* = 23.5 Hz), 106.6, 103.8, 99.8, 79.1, 59.8, 55.1, 34.8, 31.1, 19.9; ^{19}F NMR (376 MHz, CD_2Cl_2) δ –63.6, –111.6; HRMS (ESI) m/z 557.1127 ($M+Na^+$), calc. for $C_{26}H_{22}F_4N_2O_4SNa^+$ 557.1129.

The ee was determined by HPLC analysis: CHIRALPAK IA (4.6 mm i.d. x 250 mm); Hexane/2-propanol = 80/20; flow rate: 1.0 mL/min; 25 °C; 254 nm; retention time: 11.7 min (minor) and 24.6 min (major).

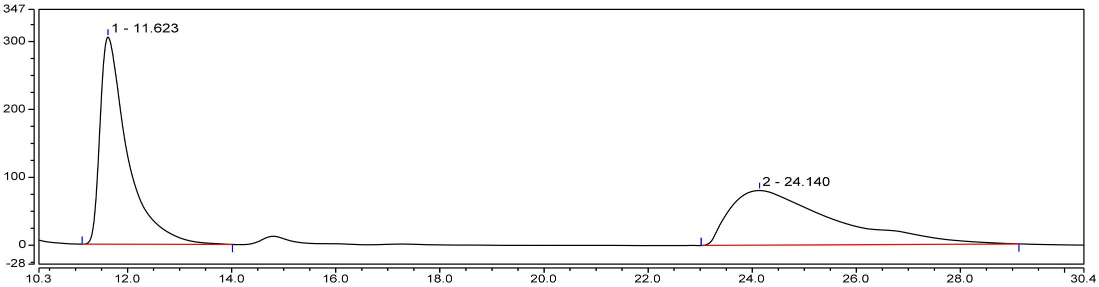

Entry	Retention Time	Area	Height	%Area
1	11.623	185.4204	304.99	49.90
2	24.140	186.1569	80.59	50.10

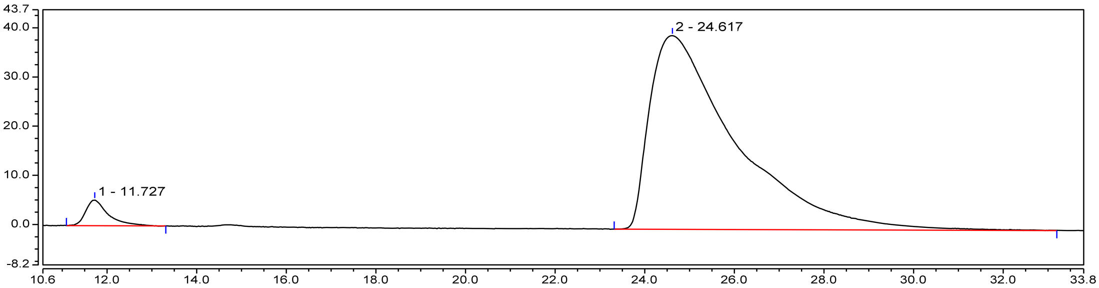

Entry	Retention Time	Area	Height	%Area
1	11.727	3.0399	5.22	3.36
2	24.617	87.5161	39.38	96.64

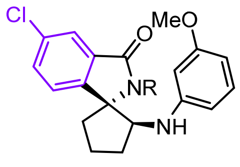

R = 4-$CF_3C_6H_4SO_2$

3n: white solid; Mp 131.5 - 132.7 °C; 46.2 mg, 84% yield; 92% ee; dr > 19:1; $[\alpha]_D^{22}$ +88.1 (*c* 1.0, $CHCl_3$); ^{1}H NMR (300 MHz, $CDCl_3$) δ 8.14 (d, *J* = 8.2 Hz, 2H), 7.73 (s, 1H), 7.70 – 7.63 (m, 1H), 7.53 (d, *J* = 8.4 Hz, 2H), 7.46 (d, *J* = 8.2 Hz, 1H), 6.99 (t, *J* = 8.1 Hz, 1H), 6.28 (d, *J* = 8.2 Hz, 1H), 6.03 (d, *J* = 8.0 Hz, 1H), 5.96 (s, 1H), 5.15 (dd, *J* = 10.6, 7.8 Hz, 1H), 3.71 (s, 3H), 3.20 – 3.06 (m, 1H), 2.74 – 2.65 (m, 1H), 2.28 – 2.18 (m, 2H), 2.16 – 2.04 (m, 1H), 1.83 – 1.69 (m, 1H); ^{13}C NMR (75 MHz, $CDCl_3$) δ 165.3, 160.8, 147.6, 146.5, 142.1, 135.8, 135.3 (q, *J* = 33.3 Hz), 134.7, 130.1, 129.9, 129.1, 125.8 (q, *J* = 3.6 Hz), 125.0, 124.0, 123.0 (q, *J* = 273.2 Hz), 106.6, 103.8, 99.8, 79.1, 59.9, 55.1, 34.8, 31.1, 19.9; ^{19}F NMR (376 MHz, CD_2Cl_2) δ –63.6; HRMS (ESI) m/z 573.0832 (M+Na$^+$), calc. for $C_{26}H_{22}ClF_3N_2O_4SNa^+$ 573.0833.

The ee was determined by HPLC analysis: CHIRALPAK IA (4.6 mm i.d. x 250 mm); Hexane/2-propanol = 80/20; flow rate: 1.0 mL/min; 25 °C; 254 nm; retention time: 11.8 min (minor) and 26.8 min (major).

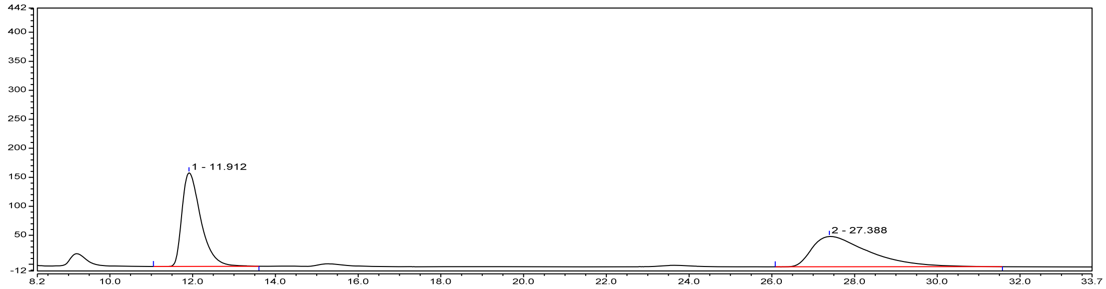

Entry	Retention Time	Area	Height	%Area
1	11.912	85.2078	161.44	50.47
2	27.388	83.6139	52.49	49.53

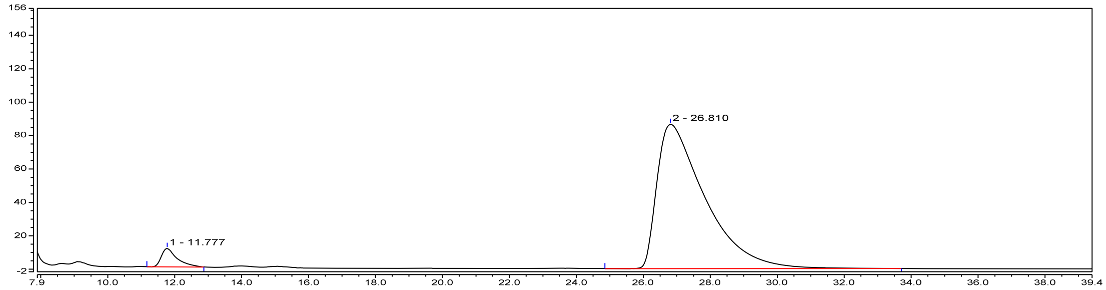

Entry	Retention Time	Area	Height	%Area
1	11.777	5.9420	11.09	3.98
2	26.810	143.4316	86.43	96.02

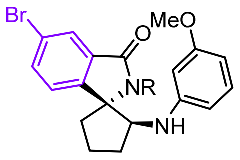

R = 4-$CF_3C_6H_4SO_2$

3o: white solid; Mp 147.5 - 148.7 °C; 55.8 mg, 94% yield; 93% ee; dr > 19:1; $[\alpha]_D^{22}$ +93.9 (*c* 1.0, $CHCl_3$); ^{1}H NMR (300 MHz, $CDCl_3$) δ 8.15 (d, *J* = 8.2 Hz, 2H), 7.90 (s, 1H), 7.82 (d, *J* = 8.2 Hz, 1H), 7.53 (d, *J* = 8.3 Hz, 2H), 7.40 (d, *J* = 8.3 Hz, 1H), 6.99 (t, *J* = 8.1 Hz, 1H), 6.29 (d, *J* = 8.0 Hz, 1H), 6.03 (d, *J* = 8.3 Hz, 1H), 5.97 (s, 1H), 5.15 (dd, *J* = 10.5, 7.9 Hz, 1H), 3.71 (s, 3H), 3.17 – 3.04 (m, 1H), 2.73 – 2.63 (m, 1H), 2.40 – 2.19 (m, 2H), 2.16 – 2.02 (m, 1H), 1.82 – 1.70 (m, 1H); ^{13}C NMR (75 MHz, $CDCl_3$) δ 165.2, 160.9, 147.6, 147.1, 142.2, 137.5, 135.3 (q, *J* = 33.2 Hz), 130.2, 130.1, 129.1, 128.1, 125.8 (q, *J* = 3.7 Hz), 124.2, 123.5, 123.0 (q, *J* = 273.3 Hz), 106.6, 103.9, 99.9, 79.2, 60.0, 55.1, 34.9, 31.2, 20.0; ^{19}F NMR (376 MHz, CD_2Cl_2) δ –63.6; HRMS (ESI) m/z 617.0327 (M+Na^+), calc. for $C_{26}H_{22}BrF_3N_2O_4SNa^+$ 617.0328.

The ee was determined by HPLC analysis: CHIRALPAK IF (4.6 mm i.d. x 250 mm); Hexane/2-propanol = 60/40; flow rate: 1.0 mL/min; 25 °C; 254 nm; Retention Time: 9.7 min (minor) and 19.3 min (major).

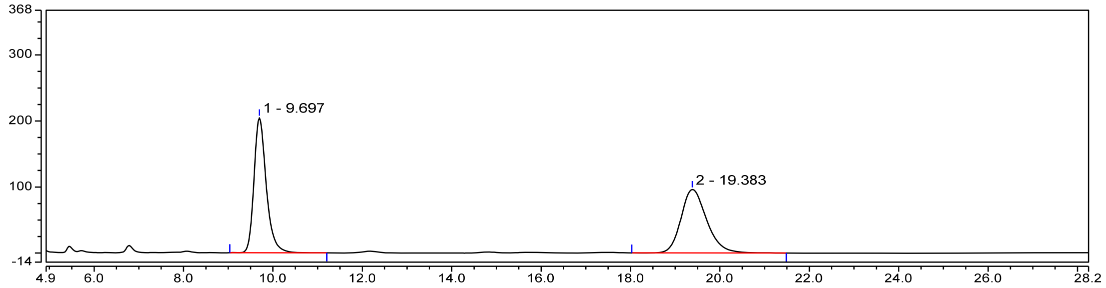

Entry	Retention Time	Area	Height	%Area
1	9.697	65.2896	204.26	50.44
2	19.383	64.1469	96.14	49.56

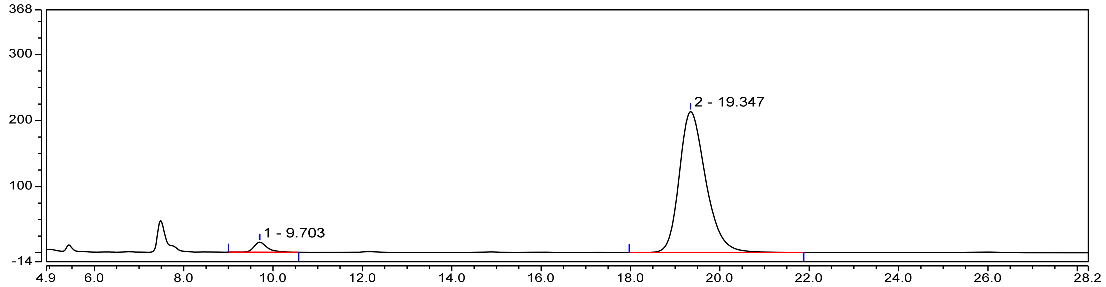

Entry	Retention Time	Area	Height	%Area
1	9.703	5.3039	15.13	3.50
2	19.347	146.3268	213.37	96.50

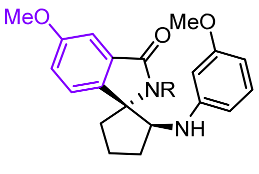

R = 4-$CF_3C_6H_4SO_2$

3p: white solid; Mp 80.6 - 81.8 °C; 43.1 mg, 79% yield; 93% ee; dr > 19:1; $[\alpha]_D^{22}$ +116.3 (*c* 1.0, $CHCl_3$); ^1H NMR (300 MHz, $CDCl_3$) δ 8.17 (d, *J* = 7.9 Hz, 2H), 7.53 (d, *J* = 7.8 Hz, 2H), 7.44 (d, *J* = 8.3 Hz, 1H), 7.36 – 7.25 (m, 2H), 7.04 (t, *J* = 7.9 Hz, 1H), 6.31 (d, *J* = 8.0 Hz, 1H), 6.06 (d, *J* = 7.9 Hz, 1H), 5.99 (s, 1H), 5.15 (t, *J* = 9.0 Hz, 1H), 3.88 (s, 3H), 3.75 (s, 3H), 3.27 – 3.11 (m, 1H), 2.76 – 2.66 (m, 1H), 2.42 – 2.21 (m, 2H), 2.19 – 2.08 (m, 1H), 1.86 – 1.70 (m, 1H); ^{13}C NMR (75 MHz, $CDCl_3$) δ 166.7, 160.8, 160.6, 147.8, 142.4, 140.5, 135.0 (q, *J* = 33.1 Hz), 130.1, 129.5, 129.0, 125.7 (q, *J* = 3.6 Hz), 123.6, 123.4, 123.0 (q, *J* = 273.4 Hz), 106.9, 106.4, 103.4, 99.6, 78.8, 59.3, 55.7, 55.0, 34.5, 30.7, 19.6; ^{19}F NMR (376 MHz, $CDCl_3$) δ –63.3; HRMS (ESI) m/z 569.1329 (M+Na$^+$), calc. for $C_{27}H_{25}F_3N_2O_5SNa^+$ 569.1328.

The ee was determined by HPLC analysis: CHIRALPAK IA (4.6 mm i.d. x 250 mm); Hexane/2-propanol = 80/20; flow rate: 1.0 mL/min; 25 °C; 254 nm; retention time: 16.6 min (minor) and 26.2 min (major).

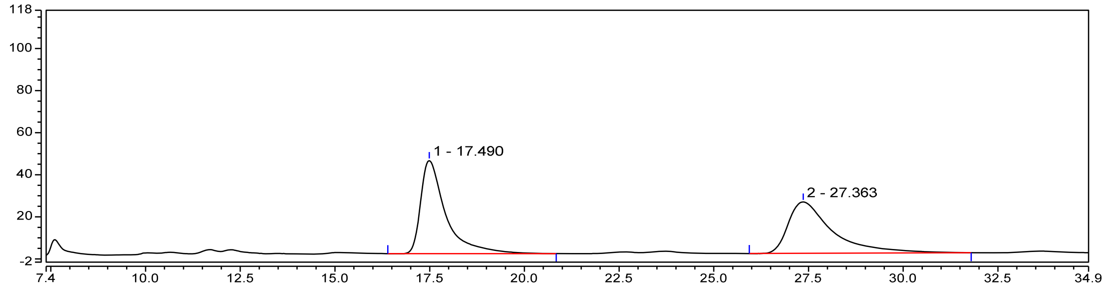

Entry	Retention Time	Area	Height	%Area
1	17.490	34.0310	44.24	50.65
2	27.363	33.1595	24.43	49.35

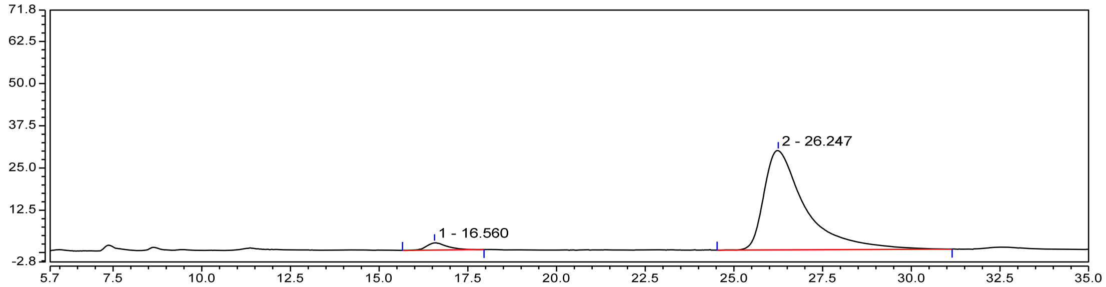

Entry	Retention Time	Area	Height	%Area
1	16.560	1.4178	2.14	3.44
2	26.247	39.7706	29.39	96.56

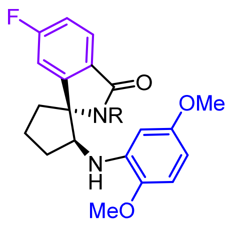

R = 4-$CF_3C_6H_4SO_2$

3q: white solid; Mp 71.6-72.9 °C; 52.4 mg, 93% yield; 95% ee; dr > 19:1; $[\alpha]_D^{22}$ +28.1 (*c* 1.0, $CHCl_3$); ^{1}H NMR (300 MHz, Chloroform-*d*) δ 8.16 (d, *J* = 8.2 Hz, 2H), 7.74 (dd, *J* = 8.4, 5.0 Hz, 1H), 7.55 (d, *J* = 8.3 Hz, 2H), 7.20 (td, *J* = 8.6, 6.3 Hz, 2H), 6.47 (d, *J* = 8.7 Hz, 1H), 6.29 (d, *J* = 2.7 Hz, 1H), 6.14 (dd, *J* = 8.7, 2.8 Hz, 1H), 5.09 (t, *J* = 9.1 Hz, 1H), 3.73 (s, 3H), 3.40 (s, 3H), 3.11 (t, *J* = 12.3 Hz, 1H), 2.70 (d, *J* = 11.1 Hz, 1H), 2.42 – 2.20 (m, 2H), 2.18 – 2.03 (m, 1H), 1.82 (d, *J* = 10.7 Hz, 1H); ^{13}C NMR (75 MHz, $CDCl_3$) δ 166.5 (d, *J* = 256.4 Hz), 165.5, 154.7, δ 151.0 (d, *J* = 9.4 Hz), δ 141.8 (d, *J* = 83.3 Hz), δ 135.1 (q, *J* = 33.1 Hz), 129.0, δ 127.1 (d, *J* = 10.2 Hz), δ 125.7 (q, *J* = 3.7 Hz), δ 124.1 (d, *J* = 2.2 Hz), δ 123.0 (q, *J* = 273.2 Hz), 117.4, 117.1, 110.6, 110.2, 100.8, 99.4, δ 78.9 (d, *J* = 2.7 Hz), 60.5, 55.9, 55.6, 35.5, 31.7, 20.5; ^{19}F NMR (376 MHz, CD_2Cl_2) δ –63.6, –102.0; HRMS (ESI) m/z 565.1411 ($M+H^+$), calc. for $C_{27}H_{25}F_4N_2O_5S^+$ 565.1415.

The ee was determined by HPLC analysis: CHIRAL MQ (x 2) (4.6 mm i.d. x 250 mm); Hexane/2-propanol = 80/20; flow rate: 1.0 mL/min; 25 °C; 210 nm; retention time: 15.2 min (minor) and 25.3 min (major).

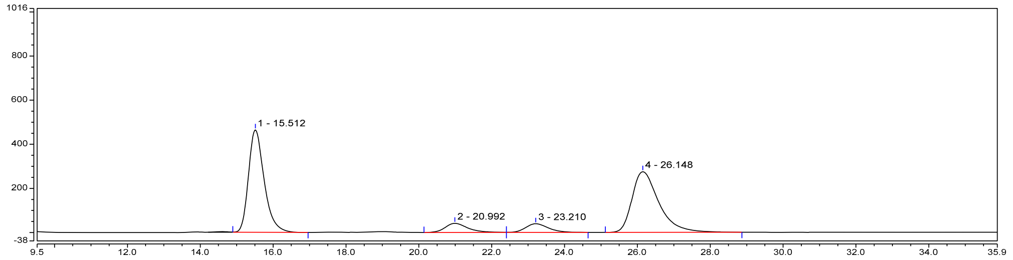

Entry	Retention Time	Area	Height	%Area
1	15.512	232.2256	464.48	44.22
2	20.992	30.2680	41.61	5.76
3	23.210	28.1793	39.38	5.37
4	26.148	234.5174	275.13	44.65

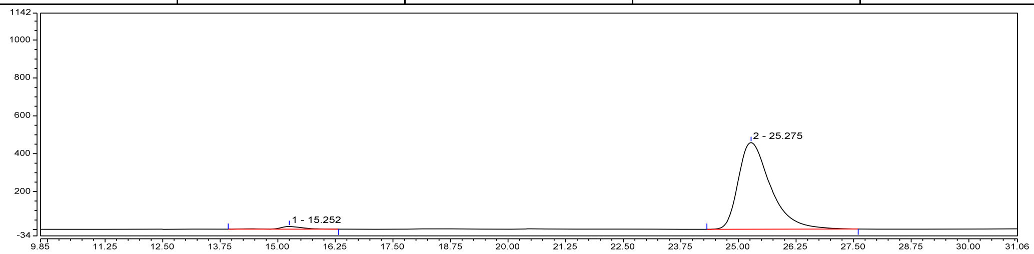

Entry	Retention Time	Area	Height	%Area
1	15.252	8.6932	15.02	2.29
2	25.275	370.9612	457.61	97.71

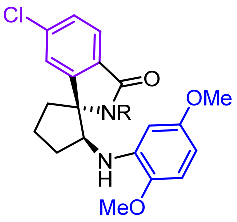

R = 4-$CF_3C_6H_4SO_2$

3r: white solid; Mp 74.5-75.4 °C; 52.8 mg, 91% yield; 91% ee; dr > 19:1; $[\alpha]_D^{22}$ +108.0 (*c* 1.0, $CHCl_3$); ^{1}H NMR (300 MHz, Chloroform-*d*) δ 8.17 (d, *J* = 8.2 Hz, 2H), 7.64 (d, *J* = 8.1 Hz, 1H), 7.57 (d, *J* = 8.3 Hz, 2H), 7.54 – 7.49 (m, 1H), 7.45 (dd, *J* = 8.2, 1.6 Hz, 1H), 6.45 (d, *J* = 8.7 Hz, 1H), 6.28 (d, *J* = 2.8 Hz, 1H), 6.12 (dd, *J* = 8.7, 2.8 Hz, 1H), 5.08 (dd, *J* = 10.5, 7.5 Hz, 1H), 3.73 (s, 3H), 3.40 (s, 3H), 3.16 – 3.02 (m, 1H), 2.80 – 2.63 (m, 1H), 2.43 – 2.23 (m, 2H), 2.20 – 2.03 (m, 1H), 1.95 – 1.73 (m, 1H); ^{13}C NMR (75 MHz, $CDCl_3$) δ 165.6, 154.6, 150.0, 142.2, 140.9, δ 135.1 (q, *J* = 33.0 Hz), 129.8, 129.0, 126.4, 125.8, 125.8, 125.7, 125.7, 123.2, δ 123.0 (q, *J* = 273.1 Hz), 110.5, 100.8, 99.3, 79.1, 60.6, 55.9, 55.5, 35.7, 31.9, 20.7; ^{19}F NMR (376 MHz, $CDCl_3$) δ –63.3; HRMS (ESI) m/z 581.1114 (M+H$^+$), calc. for $C_{27}H_{25}ClF_3N_2O_5S^+$ 581.1119.

The ee was determined by HPLC analysis: CHIRAL MQ (x 2) (4.6 mm i.d. x 250 mm); Hexane/2-propanol = 80/20; flow rate: 1.0 mL/min; 25 °C; 210 nm; retention time: 15.3 min (minor) and 26.2 min (major).

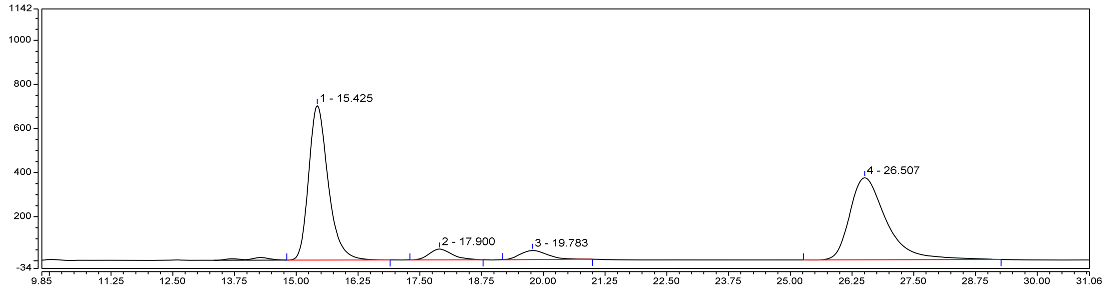

Entry	Retention Time	Area	Height	%Area
1	15.425	320.8035	700.38	46.32
2	17.900	26.3091	49.03	3.80
3	19.783	26.4540	41.36	3.82
4	26.507	319.0217	372.28	46.06

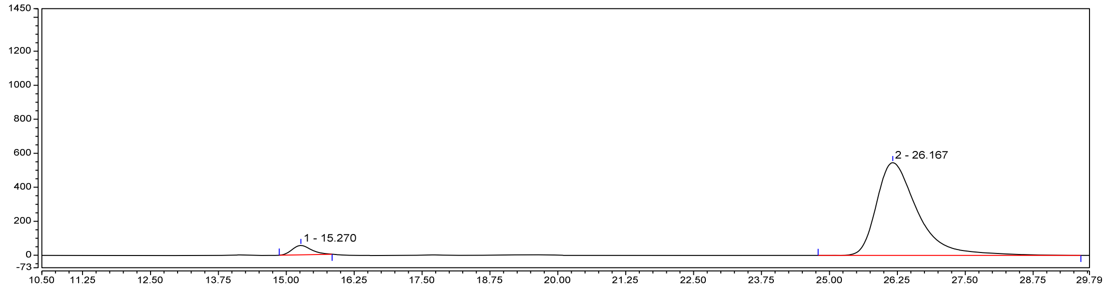

Entry	Retention Time	Area	Height	%Area
1	15.270	8.6932	22.3982	4.48
2	26.167	370.9612	477.4711	95.52

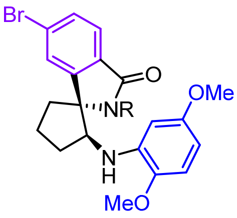

R = 4-$CF_3C_6H_4SO_2$

3s: white solid; Mp 72.6-73.3 °C; 46.8 mg, 75% yield; 90% ee; dr > 19:1; $[\alpha]_D^{22}$ +46.3 (*c* 1.0, $CHCl_3$); ^{1}H NMR (300 MHz, Chloroform-*d*) δ 8.18 (d, *J* = 8.2 Hz, 2H), 7.68 (d, *J* = 1.4 Hz, 1H), 7.64 – 7.53 (m, 4H), 6.44 (d, *J* = 8.7 Hz, 1H), 6.27 (d, *J* = 2.8 Hz, 1H), 6.12 (dd, *J* = 8.7, 2.8 Hz, 1H), 5.15 – 5.0 (m, 1H), 3.73 (s, 3H), 3.41 (s, 3H), 3.16 – 3.02 (m, 1H), 2.74 – 2.62 (m, 1H), 2.43 – 2.21 (m, 2H), 2.18 – 2.06 (m, 1H), 1.91 – 1.76 (m, 1H); ^{13}C NMR (75 MHz, $CDCl_3$) δ 165.7, 154.6, 150.1, 142.2, 141.1, 136.8, 135.1 (q, *J* = 33.0 Hz), 132.6, 129.4, 129.0, 126.8, 126.1, 125.8, 125.7 (q, *J* = 3.6 Hz), 123.0 (q, *J* = 273.2 Hz), 110.4, 100.6, 99.2, 79.1, 60.6, 55.9, 55.5, 35.8, 32.0, 20.8; ^{19}F NMR (565 MHz, $CDCl_3$) δ –63.3; HRMS (ESI) m/z 625.0608 (M+H$^+$), calc. for $C_{27}H_{25}BrF_3N_2O_5S^+$ 625.0614.

The ee was determined by HPLC analysis: CHIRAL MQ (x 2) (4.6 mm i.d. x 250 mm); Hexane/2-propanol = 80/20; flow rate: 1.0 mL/min; 25 °C; 210 nm; retention time: 15.2 min (minor) and 26.1 min (major).

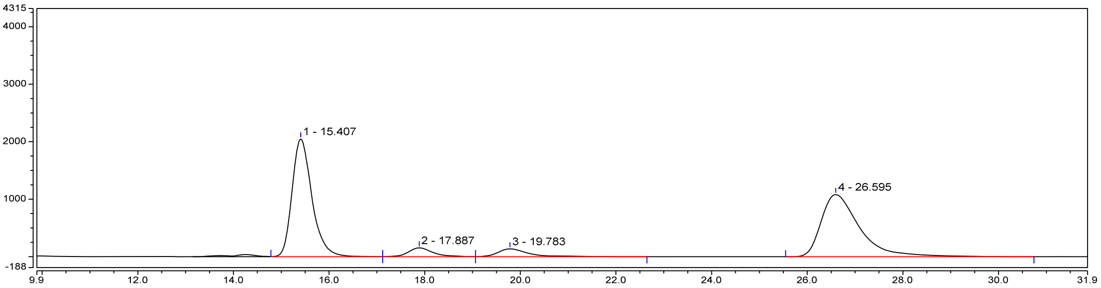

Entry	Retention Time	Area	Height	%Area
1	15.407	986.7145	2046.76	45.08
2	17.887	91.7346	153.58	4.19
3	19.783	103.2292	136.53	4.72
4	26.595	1007.3232	1081.41	46.02

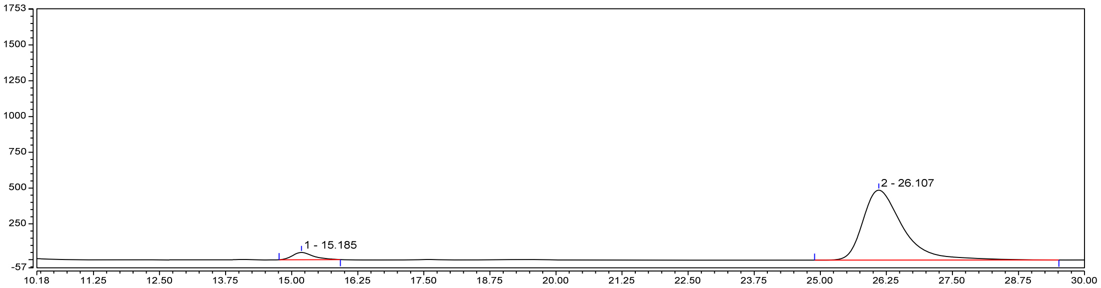

Entry	Retention Time	Area	Height	%Area
1	15.185	22.9570	50.35	5.08
2	26.107	428.8944	488.89	94.92

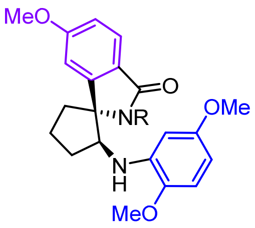

R = 4-$CF_3C_6H_4SO_2$

3t: white solid; Mp 68.1 - 69.4 °C; 50.1 mg, 87% yield; 91% ee; dr > 19:1; $[\alpha]_D^{22}$ +101.6 (*c* 1.0, $CHCl_3$); ^{1}H NMR (300 MHz, Methylene Chloride-d_2) δ 8.14 (d, *J* = 8.2 Hz, 2H), 7.62 (d, *J* = 8.4 Hz, 1H), 7.57 (d, *J* = 8.3 Hz, 2H), 7.00 (dd, *J* = 8.5, 2.1 Hz, 1H), 6.96 (d, *J* = 2.1 Hz, 1H), 6.45 (d, *J* = 8.7 Hz, 1H), 6.28 (d, *J* = 2.8 Hz, 1H), 6.08 (dd, *J* = 8.7, 2.8 Hz, 1H), 5.09 – 4.93 (m, 1H), 3.90 (s, 3H), 3.71 (s, 3H), 3.57 (s, 1H), 3.35 (s, 3H), 3.11 – 2.98 (m, 1H), 2.69 – 2.56 (m, 1H), 2.39 – 2.22 (m, 2H), 2.15 – 2.01 (m, 1H), 1.93 – 1.73 (m, 1H); ^{13}C NMR (75 MHz, CD_2Cl_2) δ 165.7, 164.5, 154.4, 150.6, 142.5, 140.7, 137.2, 134.1 (q, *J* = 32.9 Hz), 128.4, 125.7, 125.2 (q, *J* = 3.7 Hz), 122.8 (q, *J* = 273.1 Hz), 119.8, 115.0, 110.2, 107.4, 99.5, 98.4, 78.5, 60.2, 55.5, 55.5, 54.9, 35.0, 31.3, 20.1; ^{19}F NMR (376 MHz, CD_2Cl_2) δ –63.6; HRMS (ESI) m/z 577.1609 (M+H$^+$), calc. for $C_{28}H_{28}F_3N_2O_6S^+$ 577.1615.

The ee was determined by HPLC analysis: Lux Cellulose-1 (4.6 mm i.d. x 250 mm); Hexane/2-propanol = 50/50 flow rate: 1.0 mL/min; 25 °C; 210 nm; retention time: 7.5 min (major) and 9.9 min (minor).

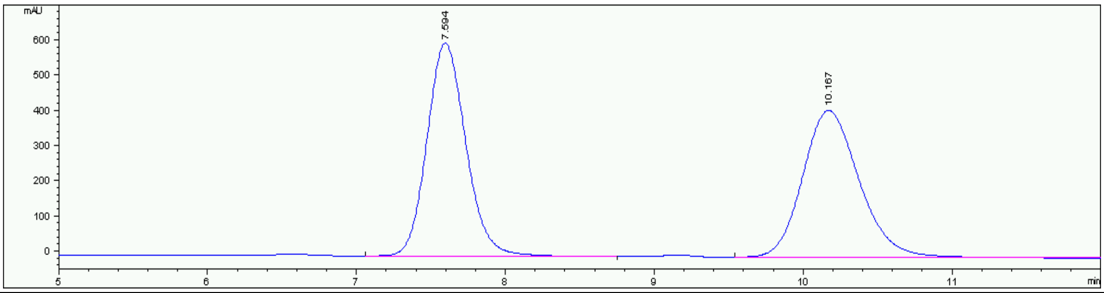

Entry	Retention Time	Area	Height	%Area
1	7.594	11216.3	607.4	50.188
2	10.167	11132.5	418.2	49.812

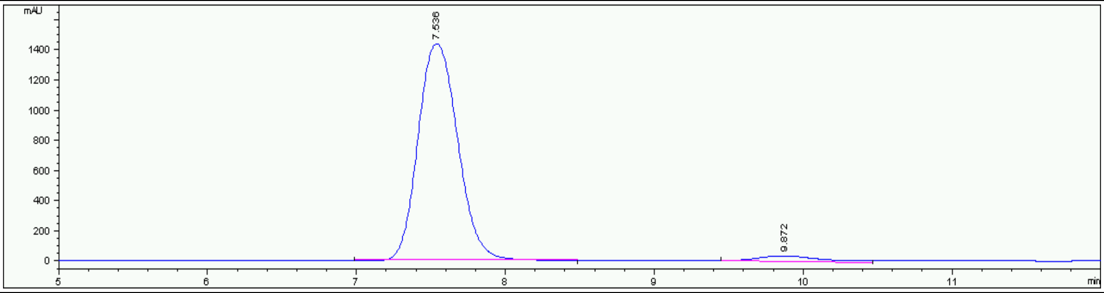

Entry	Retention Time	Area	Height	%Area
1	7.536	26411.8	1434	95.370
2	9.872	1282.2	40.3	4.630

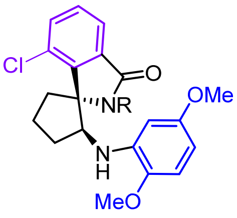

3u: white solid; Mp 73.5 - 74.9 °C; 41.8 mg, 82% yield; 97% ee; dr > 19:1; $[\alpha]_D^{22}$ +20.9 (*c* 1.0, $CHCl_3$); ^1H NMR (300 MHz, Chloroform-*d*) δ 8.17 (d, *J* = 8.2 Hz, 2H), 7.68 – 7.53 (m, 3H), 7.52 – 7.37 (m, 2H), 6.47 (d, *J* = 8.6 Hz, 1H), 6.31 (d, *J* = 2.8 Hz, 1H), 6.13 (dd, *J* = 8.7, 2.8 Hz, 1H), 5.15 – 5.01 (m, 1H), 3.74 (s, 3H), 3.38 (s, 3H), 3.17 – 3.02 (m, 1H), 2.75 – 2.57 (m, 1H), 2.43 – 2.22 (m, 2H), 2.13 – 2.04 (m, 1H), 1.90 – 1.76 (m, 1H); ^{13}C NMR (75 MHz, $CDCl_3$) δ 164.2, 154.7, 151.3, 142.2, 141.2, 137.4, 135.3, 134.8, 132.9, 130.8, 129.2, 125.8 (q, *J* = 3.5 Hz), 124.1, 123.0 (q, *J* = 273.1 Hz), 121.1, 110.7, 100.4, 99.3, 77.8, 60.9, 55.9, 55.6, 35.7, 32.1, 20.6; ^{19}F NMR (376 MHz, CD_2Cl_2) δ –63.6; HRMS (ESI) m/z 581.1111 ($M+H^+$), calc. for $C_{28}H_{28}F_3N_2O_6S^+$ 581.1119.

The ee was determined by HPLC analysis: Lux i-Amylose-1 (4.6 mm i.d. x 250 mm); Hexane/2-propanol = 75/25 flow rate: 1.0 mL/min; 25 °C; 210 nm; retention time: 12.3 min (major) and 14.7 min (minor).

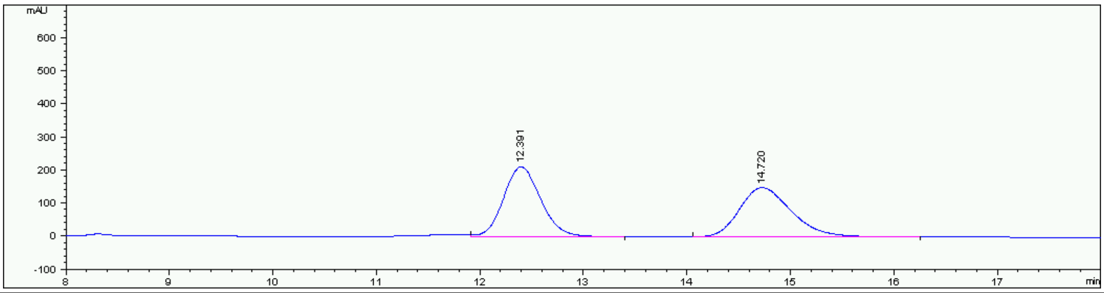

Entry	Retention Time	Area	Height	%Area
1	12.391	5362.2	211.9	50.639
2	14.72	5226.7	149.4	49.361

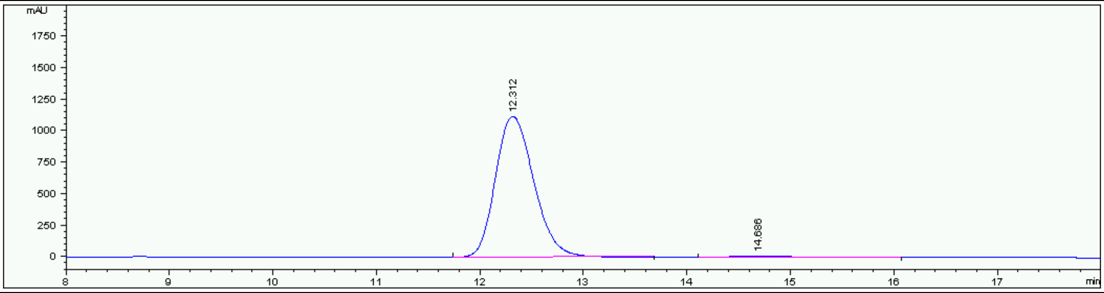

Entry	Retention Time	Area	Height	%Area
1	12.312	28900	1117.4	98.416
2	14.686	465.2	13.5	1.584

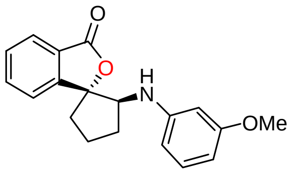

5: white solid; Mp 86.5 - 87.4 °C; 20.1 mg, 65% yield, 76% ee; dr > 19:1; $[\alpha]_D^{22}$ -35.6 (*c* 1.0, $CHCl_3$); ^{1}H NMR (300 MHz, $CDCl_3$) δ 7.81 (d, *J* = 7.2 Hz, 1H), 7.52 – 7.37 (m, 3H), 6.84 (t, *J* = 8.1 Hz, 1H), 6.21 – 6.04 (m, 1H), 5.83 (d, *J* = 7.9 Hz, 1H), 5.72 (s, 1H), 4.09 (t, *J* = 6.5 Hz, 1H), 3.58 (s, 3H), 2.73 – 2.54 (m, 1H), 2.37 – 2.18 (m, 2H), 2.15 – 1.99 (m, 2H), 1.84 – 1.68 (m, 1H). ^{13}C NMR (75 MHz, $CDCl_3$) δ 169.9 (s, 1H), 160.5 (s, 2H), 149.9 (s, 2H), 148.2 (s, 2H), 133.9 (s, 4H), 129.7 (s, 4H), 129.2 (s, 3H), 126.6 (s, 1H), 125.4 (s, 4H), 123.5 (s, 4H), 106.1 (s, 4H), 103.7 (s, 4H), 98.8 (s, 4H), 95.9 (s, 2H), 63.3 (s, 4H), 55.0 (s, 4H), 37.5 (s, 4H), 33.8 (s, 4H), 21.6 (s, 3H). HRMS (ESI) m/z 332.1255 (M+Na$^+$), calc. for $C_{19}H_{19}NO_3Na^+$ 332.1257.

The ee was determined by HPLC analysis: CHIRAL AD-H (4.0 mm i.d. x 100 mm); Hexane/2-propanol = 90/10; flow rate: 1.0 mL/min; 25 °C; 230 nm; retention time: 20.7 min (major) and 23.0 min (minor).

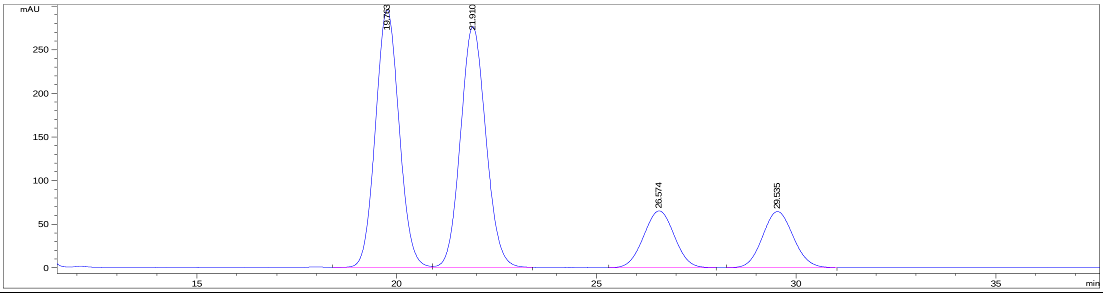

Entry	Retention Time	Area	Height	%Area
1	19.763	12159.1	295.3	38.879
2	21.91	12183.6	276.2	38.958
3	26.574	3477.4	65.1	11.119
4	29.535	3454	64.4	11.044

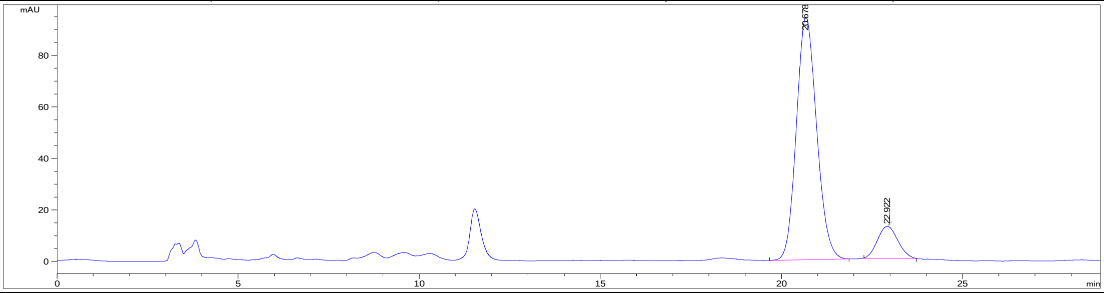

Entry	Retention Time	Area	Height	%Area
1	20.678	3636.9	93.9	87.768
2	22.922	506.9	12.8	12.232

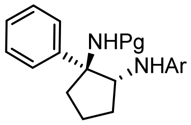

PG = 2-ClC$_6$H$_4$CO
Ar = 2,5-(OMe)$_2$Ph

7a: white solid; Mp 127.2 - 128.9 °C; 34.6 mg, 77% yield; 95% ee; dr > 19:1; $[\alpha]_D^{22}$ −14.8 (*c* 1.0, CHCl$_3$); ^{1}H NMR (300 MHz, Methylene Chloride-*d*$_2$) δ 7.54 – 7.46 (m, 3H), 7.46 – 7.40 (m, 2H), 7.40 – 7.33 (m, 3H), 7.33 – 7.27 (m, 1H), 6.99 (s, 1H), 6.59 (d, *J* = 8.7 Hz, 1H), 6.29 (d, *J* = 2.8 Hz, 1H), 6.10 (dd, *J* = 8.7, 2.8 Hz, 1H), 4.19 – 4.07 (m, 1H), 3.80 (d, *J* = 9.7 Hz, 1H), 3.68 (s, 3H), 3.60 (s, 3H), 3.05 – 2.90 (m, 1H), 2.60 – 2.46 (m, 1H), 2.22 – 2.11 (m, 1H), 2.10 – 1.94 (m, 2H), 1.44 – 1.32 (m, 1H); ^{13}C NMR (75 MHz, CD$_2$Cl$_2$) δ 165.0, 154.3, 141.2, 140.0, 137.7, 135.3, 130.6, 130.0, 129.6, 129.2, 127.7, 126.9, 126.6, 126.6, 110.1, 98.7, 98.0, 67.2, 61.2, 55.6, 54.9, 34.6, 28.1, 19.4; HRMS (ESI) m/z 451.1777 (M+H$^+$), calc. for $C_{26}H_{28}ClN_2O_3^+$ 451.1783.

The ee was determined by HPLC analysis: CHIRALPAK IF (4.6 mm i.d. x 250 mm); Hexane/2-propanol = 70/30; flow rate: 1.0 mL/min; 25 °C; 210 nm; retention time: 14.4 min (major) and 16.0 min (minor).

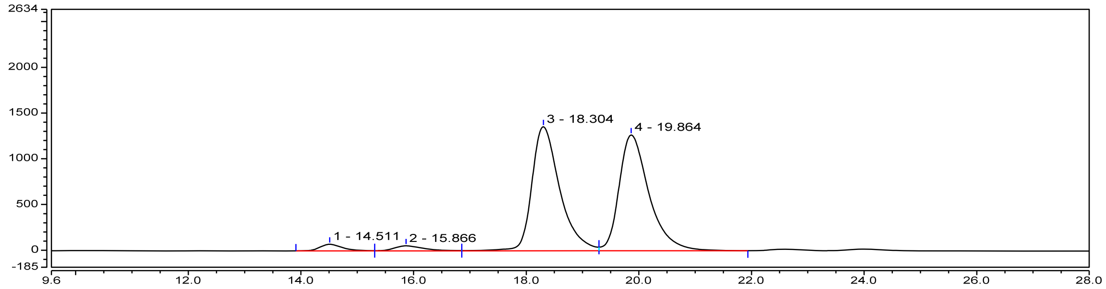

Entry	Retention Time	Area	Height	%Area
1	14.511	33.9225	72.50	2.08
2	15.866	33.6626	55.53	2.07
3	18.304	780.1998	1356.51	47.94
4	19.864	779.5285	1265.88	47.90

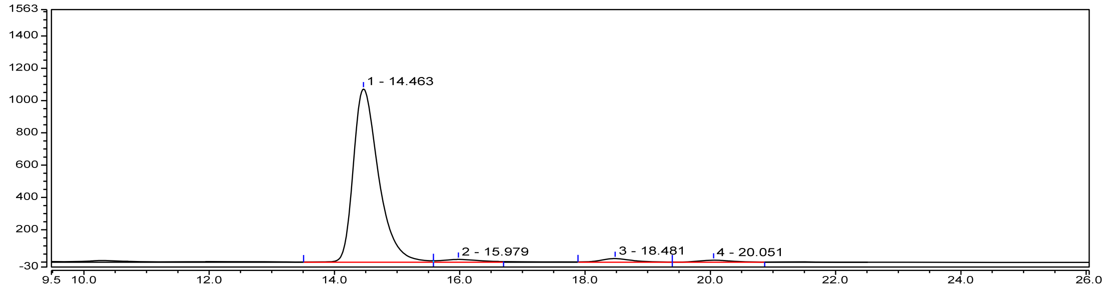

Entry	Retention Time	Area	Height	%Area
1	14.463	501.1897	1072.43	93.54
2	15.979	12.5353	17.54	2.34
3	18.481	13.6520	22.72	2.55
4	20.051	8.4155	12.91	1.57

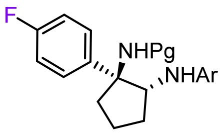

PG = 2-ClC_6H_4CO
Ar = 2,5-$(OMe)_2$Ph

7b: white solid; Mp 95.1 - 96.7 °C; 36.5 mg, 78% yield; 91% ee; dr > 19:1; $[\alpha]_D^{22}$ +28.4 (*c* 1.0, $CHCl_3$); ^{1}H NMR (300 MHz, Methylene Chloride-d_2) δ 7.50 – 7.42 (m, 3H), 7.38 – 7.25 (m, 3H), 7.14 – 6.98 (m, 3H), 6.59 (d, *J* = 8.7 Hz, 1H), 6.29 (d, *J* = 2.8 Hz, 1H), 6.11 (dd, *J* = 8.7, 2.8 Hz, 1H), 4.12 (dd, *J* = 9.5, 6.3 Hz, 1H), 3.67 (s, 3H), 3.61 (s, 3H), 2.99 – 2.87 (m, 1H), 2.56 – 2.43 (m, 1H), 2.23 – 2.10 (m, 1H), 2.08 – 1.93 (m, 2H), 1.38 – 1.30 (m, 1H); ^{13}C NMR (75 MHz, CD_2Cl_2) δ 165.1, δ 161.6 (d, *J* = 245.4 Hz), 154.3, 141.3, 137.4, 135.8 (d, *J* = 3.2 Hz), 135.1, 130.7, 130.0, 129.6, 129.2, 128.4 (d, *J* = 8.1 Hz), 126.6, 114.3 (d, *J* = 21.2 Hz), 110.0, 99.0, 98.3, 66.9, 61.3, 55.5, 54.9, 34.7, 28.1, 19.2; ^{19}F NMR (565 MHz, $CDCl_3$) δ –116.1; HRMS (ESI) m/z 469.1685 (M+H$^+$), calc. for $C_{26}H_{27}ClFN_2O_3^+$ 469.1689.

The ee was determined by HPLC analysis: CHIRALPAK IF (4.6 mm i.d. x 250 mm); Hexane/2-propanol = 60/40; flow rate: 1.0 mL/min; 25 °C; 210 nm; retention time: 9.4 min (major) and 11.7 min (minor).

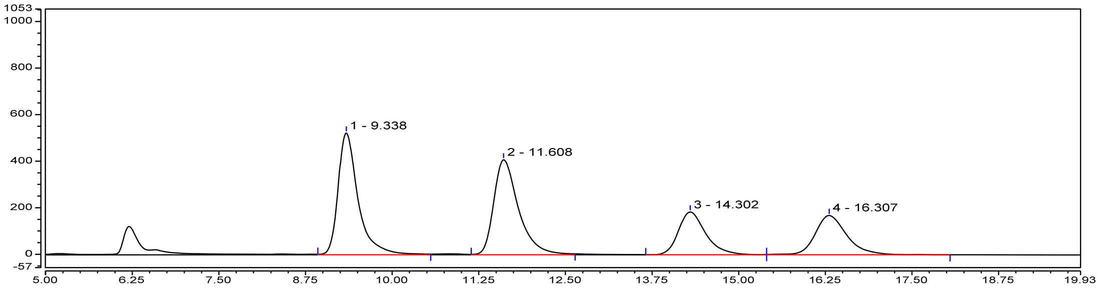

Entry	Retention Time	Area	Height	%Area
1	9.338	176.1650	523.38	33.90
2	11.608	169.6291	408.16	32.64
3	14.302	84.5760	183.87	16.28
4	16.307	89.2707	169.04	17.18

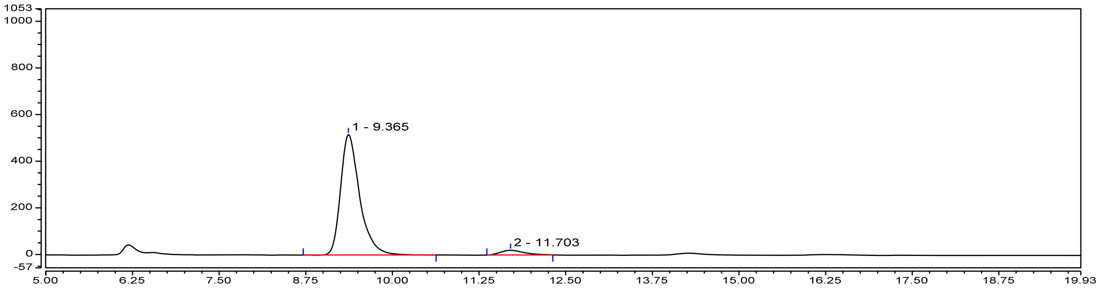

Entry	Retention Time	Area	Height	%Area
1	9.365	164.5292	527.31	95.36
2	11.703	8.0115	13.7	4.64

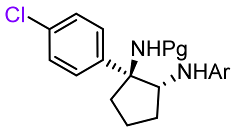

PG = 2-ClC$_6$H$_4$CO
Ar = 2,5-(OMe)$_2$Ph

7c: white solid; Mp 79.6-80.2 °C; 40.2 mg, 83% yield; 92% ee; dr > 19:1; $[\alpha]_D^{22}$ +9.7 (*c* 1.0, $CHCl_3$); ^{1}H NMR (300 MHz, Methylene Chloride-*d*$_2$) δ 7.49 (dd, *J* = 7.1, 1.8 Hz, 1H), 7.46 – 7.32 (m, 6H), 7.32 – 7.25 (m, 1H), 6.96 (s, 1H), 6.59 (d, *J* = 8.6 Hz, 1H), 6.27 (d, *J* = 2.8 Hz, 1H), 6.11 (dd, *J* = 8.7, 2.8 Hz, 1H), 4.20 – 4.05 (m, 1H), 3.80 – 3.59 (m, 7H), 3.00 – 2.85 (m, 1H), 2.54 – 2.42 (m, 1H), 2.24 – 2.11 (m, 1H), 2.09 – 1.89 (m, 2H), 1.44 – 1.29 (m, 1H); ^{13}C NMR (75 MHz, CD_2Cl_2) δ 165.0, 154.4, 141.3, 138.8, 137.6, 135.1, 132.7, 130.8, 130.1, 129.6, 129.3, 128.3, 127.7, 126.6, 110.2, 99.1, 98.3, 67.1, 61.4, 55.6, 55.0, 34.7, 28.4, 19.3; HRMS (ESI) m/z 485.1389 (M+H$^+$), calc. for $C_{26}H_{27}Cl_2N_2O_3^+$ 485.1393.

The ee was determined by HPLC analysis: CHIRALPAK IG (4.6 mm i.d. x 250 mm); Hexane/2-propanol = 50/50; flow rate: 1.0 mL/min; 25 °C; 210 nm; retention time: 9.9 min (major) and 14.9 min (minor).

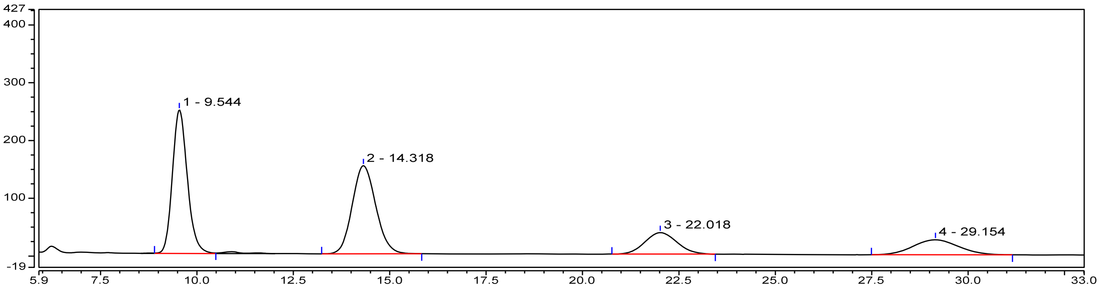

Entry	Retention Time	Area	Height	%Area
1	9.544	111.9148	248.48	38.36
2	14.318	108.1226	152.40	37.06
3	22.018	35.9756	37.24	12.33
4	29.154	35.7082	25.98	12.24

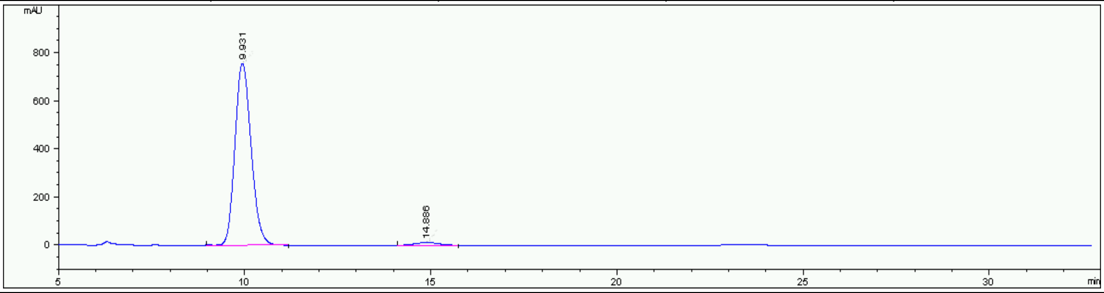

Entry	Retention Time	Area	Height	%Area
1	9.931	23254.6	760.6	95.869
2	14.886	1002.2	16.7	4.131

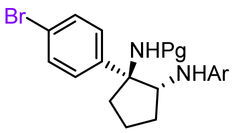

7d: white solid; Mp 75.2 - 76.6 °C; 42.8 mg, 81% yield; 91% ee; dr > 19:1; $[\alpha]_D^{22}$ +18.1 (*c* 1.0, $CHCl_3$); ^{1}H NMR (300 MHz, Methylene Chloride-d_2) δ 7.55 – 7.46 (m, 3H), 7.40 – 7.32 (m, 4H), 7.31 – 7.24 (m, 1H), 7.05 (s, 1H), 6.60 (d, *J* = 8.7 Hz, 1H), 6.29 (d, *J* = 2.8 Hz, 1H), 6.12 (dd, *J* = 8.7, 2.8 Hz, 1H), 4.11 (dd, *J* = 9.7, 6.3 Hz, 1H), 3.67 (s, 3H), 3.61 (s, 3H), 2.99 – 2.87 (m, 1H), 2.53 – 2.41 (m, 1H), 2.22 – 2.10 (m, 1H), 2.05 – 1.90 (m, 2H), 1.37 – 1.27 (m, 1H); ^{13}C NMR (75 MHz, CD_2Cl_2) δ 165.0, 154.2, 141.2, 139.2, 135.0, 130.7, 130.7, 130.0, 129.6, 129.2, 128.6, 126.6, 120.9, 110.1, 99.3, 98.4, 67.0, 61.4, 55.5, 54.9, 34.7, 28.1, 19.2; HRMS (ESI) m/z 529.0886 (M+H$^+$), calc. for $C_{26}H_{27}BrClN_2O_3^+$ 529.0888.

The ee was determined by HPLC analysis: CHIRALPAK IF (4.6 mm i.d. x 250 mm); Hexane/2-propanol = 50/50; flow rate: 1.0 mL/min; 25 °C; 210 nm; retention time: 8.5 min (major) and 11.6 min (minor).

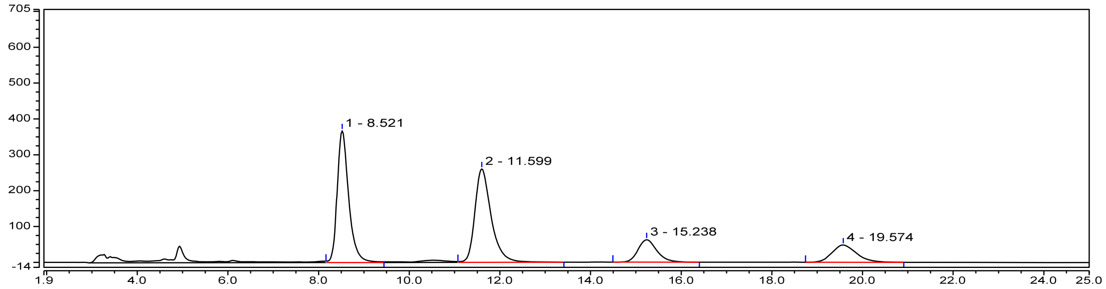

Entry	Retention Time	Area	Height	%Area
1	8.521	108.4658	367.56	39.13
2	11.599	108.3417	260.56	39.08
3	15.238	30.1985	62.54	10.89
4	19.574	30.2029	48.08	10.90

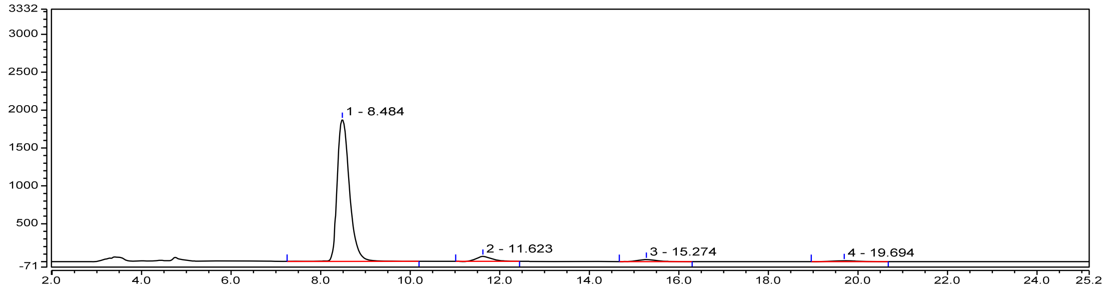

Entry	Retention Time	Area	Height	%Area
1	8.484	592.1098	1871.82	92.72
2	11.623	26.3369	65.11	4.12
3	15.274	13.0332	26.66	2.04
4	19.694	7.1301	11.48	1.12

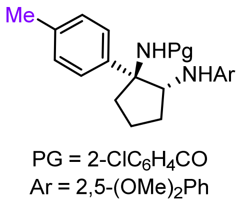

7e: white solid; Mp 74.8 - 75.9 °C; 34.8 mg, 75% yield; 94% ee; dr > 19:1; $[\alpha]_D^{22}$ +20.1 (*c* 1.0, $CHCl_3$); ^{1}H NMR (300 MHz, Methylene Chloride-d_2) δ 7.49 (dd, *J* = 7.5, 1.8 Hz, 1H), 7.39 – 7.32 (m, 4H), 7.29 (dd, *J* = 7.1, 2.4 Hz, 1H), 7.22 (d, *J* = 8.0 Hz, 2H), 6.90 (s, 1H), 6.58 (d, *J* = 8.6 Hz, 1H), 6.27 (d, *J* = 2.8 Hz, 1H), 6.08 (dd, *J* = 8.6, 2.8 Hz, 1H), 4.15 – 4.03 (m, 1H), 3.82 (d, *J* = 9.8 Hz, 1H), 3.67 (s, 3H), 3.60 (s, 3H), 3.00 – 2.89 (m, 1H), 2.54 – 2.43 (m, 1H), 2.37 (s, 3H), 2.20 – 2.10 (m, 1H), 2.07 – 1.94 (m, 2H), 1.35 – 1.25 (m, 1H); ^{13}C NMR (75 MHz, CD_2Cl_2) δ 165.0, 154.4, 141.2, 137.8, 137.0, 136.7, 135.4, 130.6, 130.0, 129.6, 129.2, 128.4, 126.6, 126.5, 110.2, 98.7, 98.0, 66.9, 61.2, 55.6, 54.9, 34.6, 28.0, 20.3, 19.3; HRMS (ESI) m/z 465.1937 (M+H$^+$), calc. for $C_{27}H_{30}ClN_2O_3^+$ 465.1939.

The ee was determined by HPLC analysis: CHIRALPAK IF (4.6 mm i.d. x 250 mm); Hexane/2-propanol = 60/40; flow rate: 1.0 mL/min; 25 °C; 210 nm; retention time: 12.0 min (major) and 15.4 min (minor).

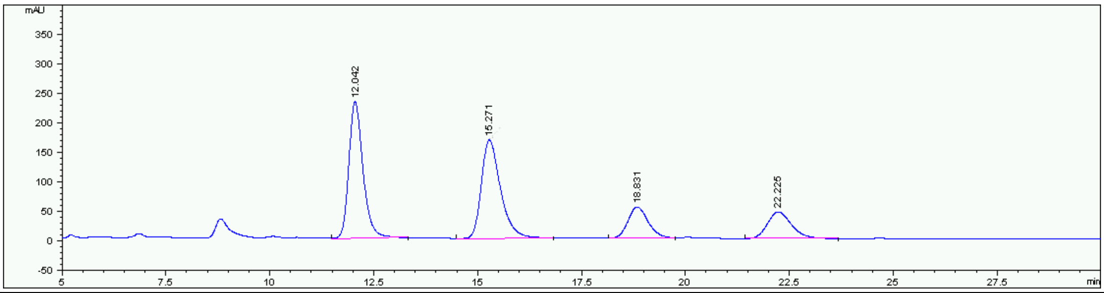

Entry	Retention Time	Area	Height	%Area
1	12.042	5599.4	233.2	37.738
2	15.271	5590.5	168.6	37.678
3	18.831	1806.6	52.9	12.176
4	22.225	1841	45.3	12.408

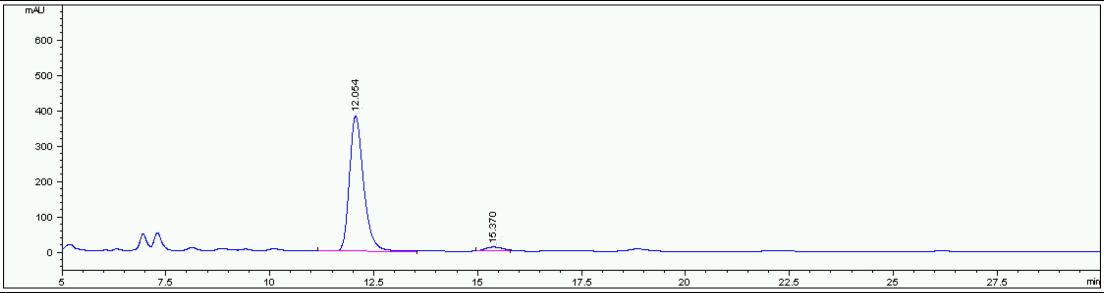

Entry	Retention Time	Area	Height	%Area
1	12.054	9189.7	382.5	96.851
2	15.37	298.8	10.5	3.149

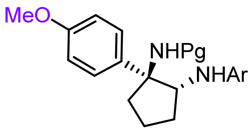

7f: white solid; Mp 76.5 - 77.7 °C; 33.6 mg, 70% yield; 85% ee; dr > 19:1; $[\alpha]_D^{22}$ +104.8 (*c* 1.0, $CHCl_3$); ^{1}H NMR (300 MHz, Chloroform-d) δ 7.48 (dd, J = 7.0, 2.1 Hz, 1H), 7.26 – 7.12 (m, 4H), 7.04 – 6.90 (m, 3H), 6.77 (dd, J = 7.8, 2.2 Hz, 1H), 6.51 (d, J = 8.6 Hz, 1H), 6.26 (d, J = 2.8 Hz, 1H), 6.04 (dd, J = 8.7, 2.8 Hz, 1H), 4.15 – 4.04 (m, 1H), 3.70 (s, 3H), 3.62 (s, 3H), 3.54 (s, 3H), 3.02 – 2.88 (m, 1H), 2.53 – 2.38 (m, 1H), 2.13 – 2.01 (m, 1H), 1.99 – 1.85 (m, 2H), 1.40 – 1.24 (m, 1H); ^{13}C NMR (75 MHz, CD2Cl2) δ 165.4, 154.8, 141.7, 138.3, 137.4, 137.2, 135.8, 131.0, 130.5, 130.0, 129.7, 128.9, 127.0, 126.9, 110.6, 99.1, 98.4, 67.4, 61.6, 56.1, 55.4, 35.1, 28.4, 20.7, 19.8; HRMS (ESI) m/z 481.1885 (M+H$^+$), calc. for $C_{27}H_{30}ClN_2O_4^+$ 481.1889.

The ee was determined by HPLC analysis: CHIRALPAK IG (4.6 mm i.d. x 250 mm) x 2; Hexane/2-propanol = 95/5; flow rate: 1.0 mL/min; 25 °C; 210 nm; retention time: 41.5 min (minor) and 44.1 min (major).

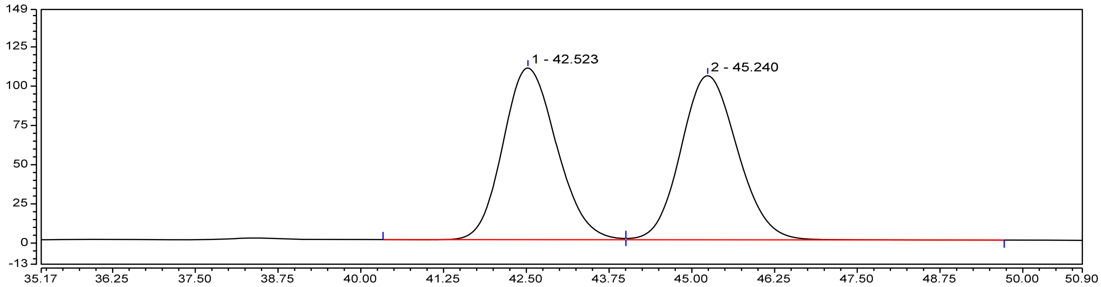

Entry	Retention Time	Area	Height	%Area
1	42.523	103.0916	109.43	49.86
2	45.240	103.6894	104.59	50.14

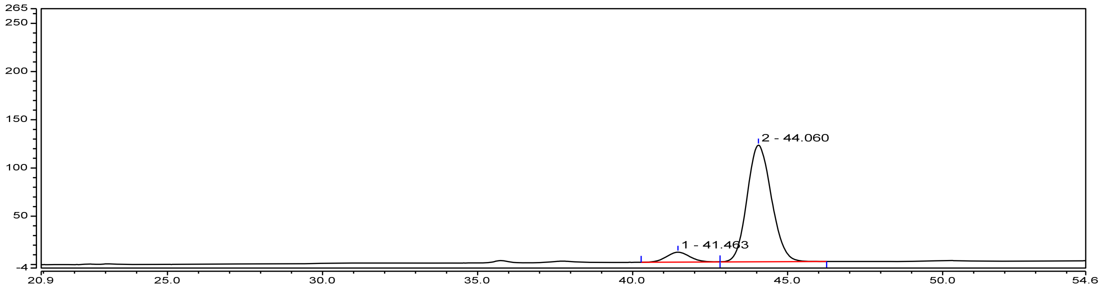

Entry	Retention Time	Area	Height	%Area
1	41.463	9.0922	10.32	7.62
2	44.060	110.2388	120.97	92.38

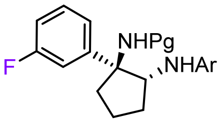

7g: white solid; Mp 65.8 - 66.9 °C; 34.6 mg, 74% yield; 95% ee; dr = 14:1; $[\alpha]_D^{22}$ +11.6 (*c* 1.0, $CHCl_3$); ^{1}H NMR (300 MHz, Methylene Chloride-d_2) δ 7.52 – 7.46 (m, 1H), 7.42 – 7.33 (m, 3H), 7.32 – 7.25 (m,

2H), 7.20 (dt, J = 10.9, 2.2 Hz, 1H), 7.09 – 6.99 (m, 2H), 6.61 (d, J = 8.7 Hz, 1H), 6.28 (d, J = 2.8 Hz, 1H), 6.11 (dd, J = 8.6, 2.8 Hz, 1H), 4.20 4.06 (m, 1H), 3.79 (d, J = 10.3 Hz, 1H), 3.68 (s, 3H), 3.63 (s, 3H), 3.02 – 2.89 (m, 1H), 2.56 – 2.43 (m, 1H), 2.24 – 2.12 (m, 1H), 2.08 – 1.94 (m, 2H), 1.43 – 1.33 (m, 1H); ^{13}C NMR (75 MHz, CD_2Cl_2) δ 165.1, 162.4 (d, J = 244.6 Hz), 154.3, 143.0 (d, J = 6.6 Hz), 141.2, 137.5, 134.9, 130.8, 130.0, 129.6, 129.3, 129.1 (d, J = 8.3 Hz), 126.6, 122.4 (d, J = 2.8 Hz), 114.0, 113.8 (d, J = 3.2 Hz), 113.5, 110.1, 99.0, 98.2, 67.2 (d, J = 1.6 Hz), 61.4, 55.5, 54.9, 34.7, 28.2, 19.3; ^{19}F NMR (376 MHz, CD_2Cl_2) δ –113.8; HRMS (ESI) m/z 469.1686 (M+H$^+$), calc. for $C_{26}H_{27}ClFN_2O_3^+$ 469.1689.

The ee was determined by HPLC analysis: CHIRALPAK IG (4.6 mm i.d. x 250 mm); Hexane/2-propanol = 60/40; flow rate: 1.0 mL/min; 25 °C; 210 nm; retention time: 11.0 min (major) and 14.9 min (minor).

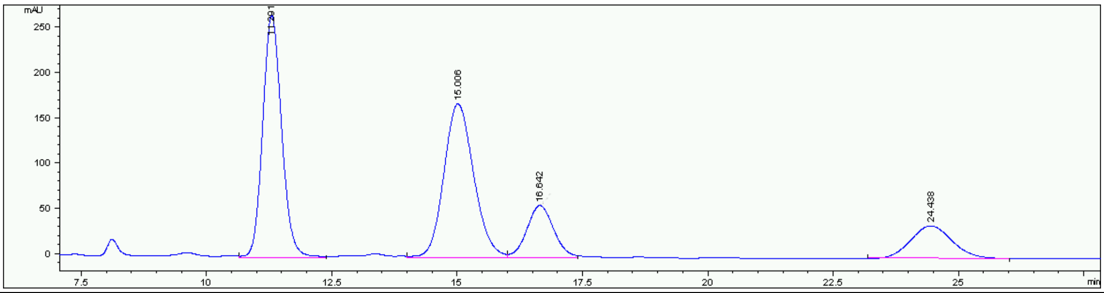

Entry	Retention Time	Area	Height	%Area
1	11.291	7107.4	267.7	38.332
2	15.006	7127.3	170.7	38.439
3	16.642	2216.2	58.4	11.952
4	24.438	2090.9	36.1	11.277

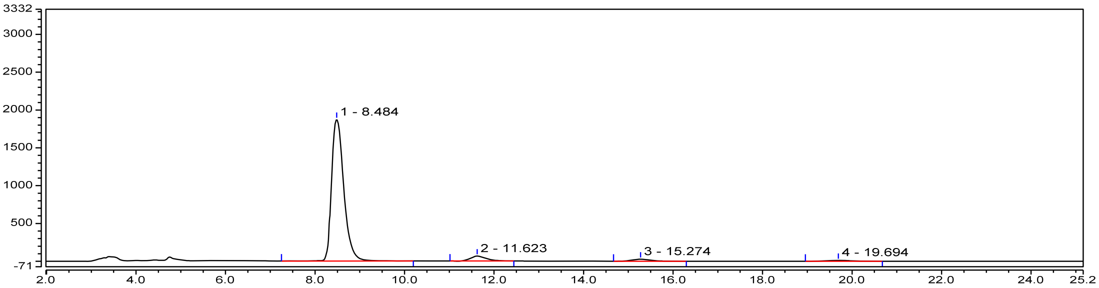

Entry	Retention Time	Area	Height	%Area
1	11.048	32569.6	1114	91.090
2	14.93	861	18.7	2.408
3	16.678	1937.6	50.2	5.419
4	24.332	387.2	6.5	1.083

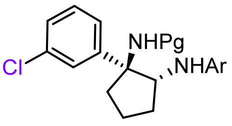

PG = 2-ClC$_6$H$_4$CO
Ar = 2,5-(OMe)$_2$Ph

7h: white solid; Mp 67.9 - 68.9 °C; 38.2 mg, 79% yield; 94% ee; dr = 15:1; $[\alpha]_D^{22}$ −13.1 (*c* 1.0, CHCl$_3$); ^{1}H NMR (300 MHz, Methylene Chloride-*d*$_2$) δ 7.53 – 7.46 (m, 2H), 7.41 – 7.33 (m, 4H), 7.32 – 7.25 (m, 2H), 6.96 (s, 1H), 6.60 (d, *J* = 8.7 Hz, 1H), 6.27 (d, *J* = 2.8 Hz, 1H), 6.10 (dd, *J* = 8.6, 2.9 Hz, 1H), 4.19 – 4.06 (m, 1H), 3.77 (d, *J* = 10.5 Hz, 1H), 3.67 (s, 3H), 3.64 (s, 2.80H), 3.36 (s, 0.20H), 3.02 – 2.89 (m, 1H), 2.54 – 2.40 (m, 1H), 2.24 – 2.11 (m 1H), 2.08 – 1.94 (m, 2H), 1.41 – 1.29 (m, 1H); ^{13}C NMR (75 MHz, CD$_2$Cl$_2$) δ 165.1, 154.4, 142.4, 141.2, 137.5, 135.0, 133.8, 130.8, 130.1, 129.7, 129.3, 129.0, 127.0, 127.0, 126.7, 125.0, 110.2, 99.1, 98.2, 67.2, 61.4, 55.7, 55.0, 34.7, 28.3, 19.3; HRMS (ESI) m/z 485.1389 (M+H$^+$), calc. for $C_{26}H_{27}Cl_2N_2O_3^+$ 485.1393.

The ee was determined by HPLC analysis: CHIRAL MQ (x 2) (4.6 mm i.d. x 250 mm); Hexane/2-propanol = 70/30; flow rate: 1.0 mL/min; 25 °C; 210 nm; retention time: 8.4 min (minor) and 9.2 min (major).

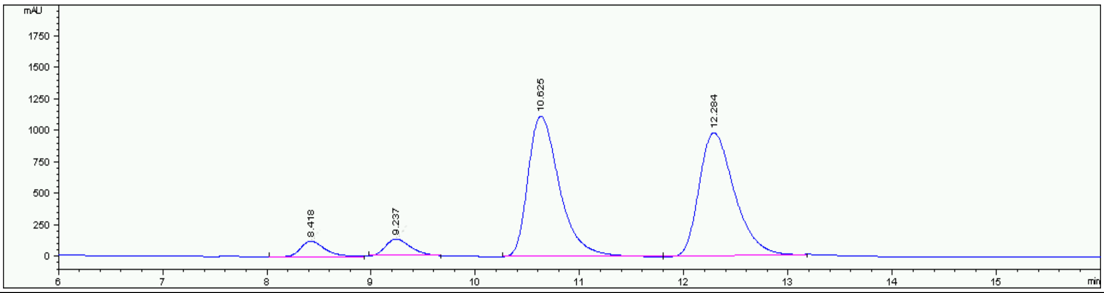

Entry	Retention Time	Area	Height	%Area
1	8.418	2240.2	127.8	4.360
2	9.237	2180.1	131.9	4.243
3	10.625	23570.2	1116.1	45.876
4	12.284	23388	981.4	45.521

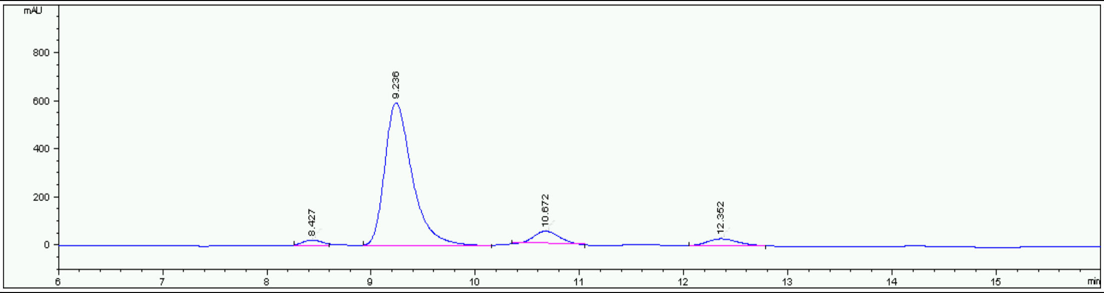

Entry	Retention Time	Area	Height	%Area
1	8.427	351.2	25.4	2.728
2	9.236	11082.6	596.4	86.097
3	10.672	860.1	51.4	6.682
4	12.352	578.4	28.6	4.493

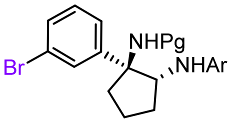

PG = 2-ClC$_6$H$_4$CO
Ar = 2,5-(OMe)$_2$Ph

7i: white solid; Mp 89.7 - 90.3 °C; 42.8 mg, 81% yield; 95% ee; dr = 17:1; $[\alpha]_D^{22}$ +17.2 (*c* 1.0, $CHCl_3$); ^{1}H NMR (300 MHz, Methylene Chloride-d_2) δ 7.67 – 7.61 (m, 1H), 7.52 – 7.44 (m, 2H), 7.43 – 7.33 (m, 3H), 7.33 – 7.24 (m, 2H), 6.97 (s, 1H), 6.60 (d, J = 8.6 Hz, 1H), 6.26 (d, J = 2.8 Hz, 1H), 6.10 (dd, J = 8.6, 2.8 Hz, 1H), 4.18 – 4.04 (m, 1H), 3.78 (d, J = 10.0 Hz, 1H), 3.67 (s, 3H), 3.65 (s, 2.83H), 3.36 (s, 0.17H), 3.02 – 2.86 (m, 1H), 2.56 – 2.34 (m, 1H), 2.23 – 2.11 (m, 1H), 2.09 – 1.92 (m, 2H), 1.39 – 1.27 (m, 1H); ^{13}C NMR (75 MHz, CD_2Cl_2) δ 165.1, 154.3, 142.6, 141.2, 137.4, 135.0, 130.8, 130.0, 129.9, 129.9, 129.6, 129.2, 129.2, 126.6, 125.4, 122.0, 110.1, 99.0, 98.1, 67.1, 61.3, 55.6, 54.9, 34.6, 28.2, 19.3; HRMS (ESI) m/z 529.0883 (M+H$^+$), calc. for $C_{26}H_{27}BrClN_2O_3^+$ 529.0888.

The ee was determined by HPLC analysis: CHIRAL MQ (x 2) (4.6 mm i.d. x 250 mm); Hexane/2-propanol = 70/30; flow rate: 1.0 mL/min; 25 °C; 210 nm; retention time: 8.9 min (minor) and 9.7 min (major).

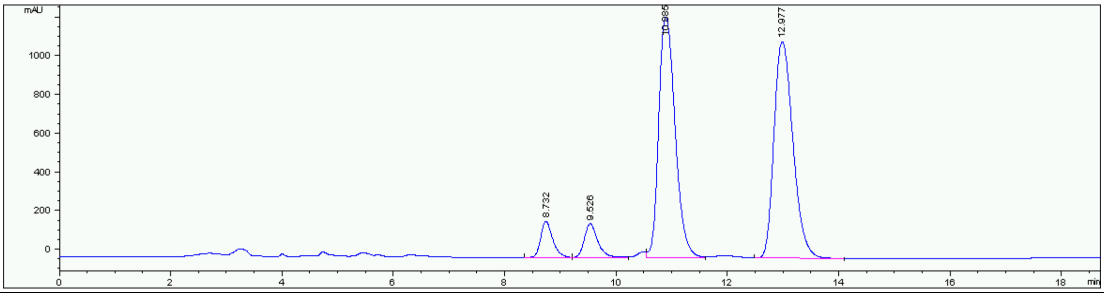

Entry	Retention Time	Area	Height	%Area
1	8.732	2905.4	189	4.977
2	9.526	3076.3	177.7	5.270
3	10.885	25720	1246.2	44.061
4	12.977	26671.6	1121	45.691

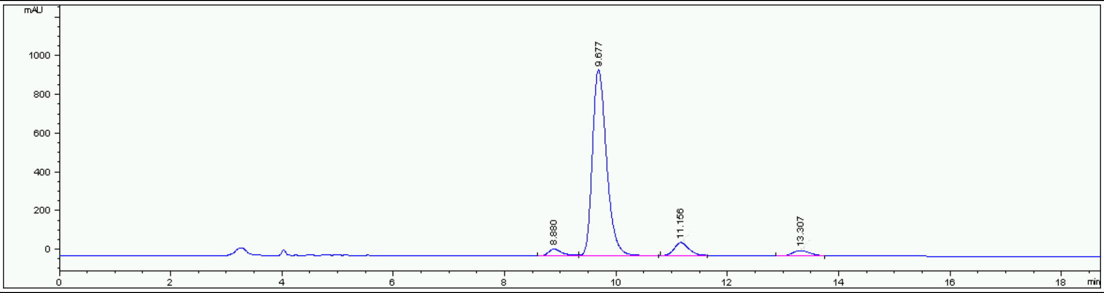

Entry	Retention Time	Area	Height	%Area
1	8.88	615.7	35.5	3.130
2	9.677	17062.4	961.2	86.753
3	11.156	1336.8	69.2	6.797
4	13.307	653	29.2	3.320

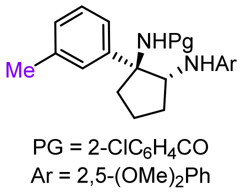

7j: white solid; Mp 151.7 - 152.3 °C; 30.6 mg, 66% yield; 94% ee; dr = 18:1; $[\alpha]_D^{22}$ –9.2 (*c* 1.0, $CHCl_3$); ^1H NMR (300 MHz, Methylene Chloride-d_2) δ 7.54 – 7.45(m, 1H), 7.38 – 7.32 (m, 2H), 7.32 – 7.22 (m, 4H), 7.21 – 7.10 (m, 1H), 6.91 (s, 1H), 6.58 (d, *J* = 8.6 Hz, 1H), 6.27 (d, *J* = 2.8 Hz, 1H), 6.08 (dd, *J* = 8.6, 2.9 Hz, 1H), 4.09 (td, *J* = 9.8, 6.2 Hz, 1H), 3.84 (d, *J* = 9.9 Hz, 1H), 3.67 (s, 3H), 3.60 (s, 2.84H), 3.30 (s, 0.16H), 3.05 – 2.89 (m, 1H), 2.56 – 2.43 (m, 1H), 2.39 (s, 3H), 2.21 – 2.11 (m, 1H), 2.09 – 1.94 (m, 2H), 1.38 – 1.28 (m, 1H); ^{13}C NMR (75 MHz, CD_2Cl_2) δ 165.0, 154.4, 141.2, 140.0, 137.8, 137.5, 135.4, 130.6, 130.1, 129.6, 129.2, 127.7, 127.6, 127.4, 126.6, 123.6, 110.2, 98.7, 98.0, 67.0, 61.1, 55.6, 54.9, 34.6, 28.0, 21.0, 19.4; HRMS (ESI) m/z 465.1936 (M+H$^+$), calc. for $C_{27}H_{30}ClN_2O_3^+$ 465.1939.

The ee was determined by HPLC analysis: CHIRAL INB (x 2) (4.6 mm i.d. x 250 mm); Hexane/2-propanol = 80/20; flow rate: 1.0 mL/min; 25 °C; 210 nm; retention time: 19.1 min (minor) and 20.4 min (major).

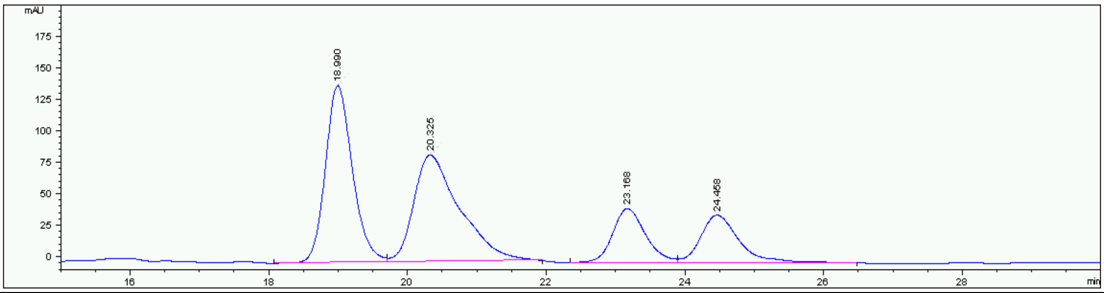

Entry	Retention Time	Area	Height	%Area
1	18.99	3798.4	140.2	35.825
2	20.325	3860.3	84.3	36.409
3	23.168	1448.4	43.2	13.661
4	24.458	1495.5	38	14.105

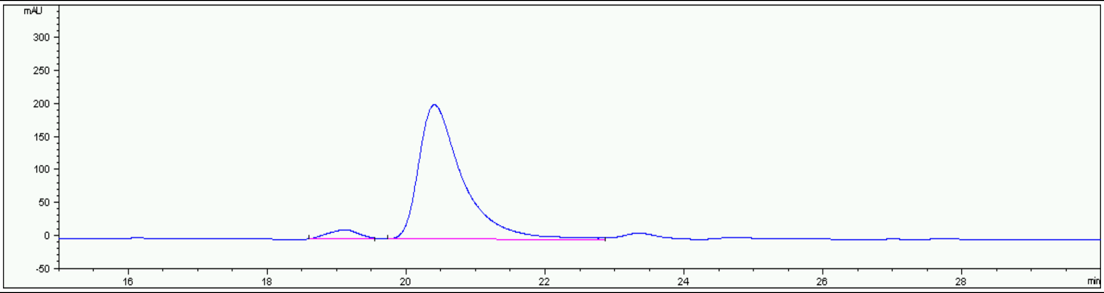

Entry	Retention Time	Area	Height	%Area
1	19.111	466.2	14.6	4.902
2	20.402	9045.8	204.5	95.098

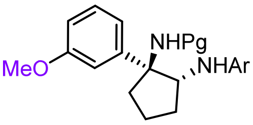

PG = 2-ClC$_6$H$_4$CO
Ar = 2,5-(OMe)$_2$Ph

7k: white solid; Mp 145.0 - 146.6 °C; 33.6 mg, 70% yield; 85% ee; dr = 16:1; $[\alpha]_D^{22}$ −12.3 (*c* 1.0, CHCl$_3$); ^{1}H NMR (300 MHz, Chloroform-*d*) δ 7.55 (dd, *J* = 7.0, 2.1 Hz, 1H), 7.34 – 7.21 (m, 4H), 7.12 – 6.97 (m, 3H), 6.84 (dd, *J* = 7.7, 2.2 Hz, 1H), 6.58 (d, *J* = 8.6 Hz, 1H), 6.33 (d, *J* = 2.8 Hz, 1H), 6.11 (dd, *J* = 8.7, 2.8 Hz, 1H), 4.15 (dd, *J* = 10.2, 6.2 Hz, 1H), 3.85 – 3.72 (m, 4H), 3.69 (s, 3H), 3.61 (s, 2.82H), 3.33 (s, 0.18H), 3.10 – 2.95 (m, 1H), 2.59 – 2.44 (m, 1H), 2.18 – 2.08 (m, 1H), 2.06 – 1.94 (m, 2H), 1.46 – 1.30 (m, 1H); ^{13}C NMR (75 MHz, CDCl$_3$) δ 165.5, 159.5, 154.7, 141.7, 141.6, 135.2, 131.1, 130.5, 130.2, 130.1, 129.1, 127.0, 119.3, 112.9, 112.9, 110.6, 98.6, 67.6, 61.5, 56.0, 55.5, 55.1, 35.0, 28.2, 19.8; HRMS (ESI) m/z 481.1884 (M+H$^+$), calc. for $C_{27}H_{30}ClN_2O_4^+$ 481.1889.

The ee was determined by HPLC analysis: CHIRAL MQ (x 2) (4.6 mm i.d. x 250 mm); Hexane/2-propanol = 60/40; flow rate: 1.0 mL/min; 25 °C; 210 nm; retention time: 9.9 min (minor) and 11.8 min (major).

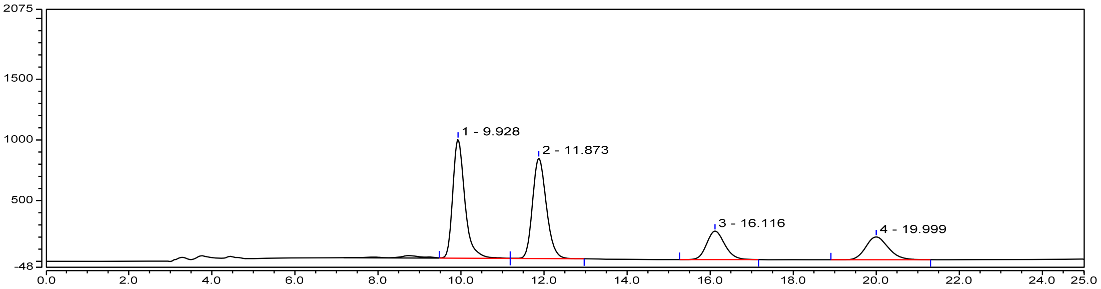

Entry	Retention Time	Area	Height	%Area
1	9.928	332.6943	979.17	37.52
2	11.873	317.8988	825.87	35.85
3	16.116	118.2555	233.89	13.34
4	19.999	117.8002	187.98	13.29

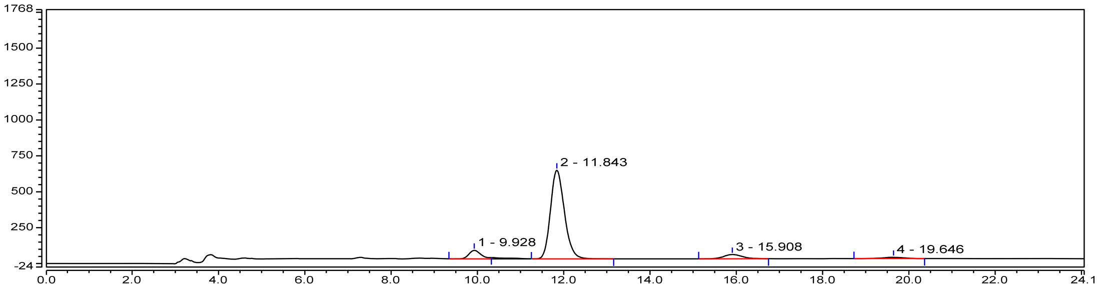

Entry	Retention Time	Area	Height	%Area
1	9.928	19.1229	59.40	7.07
2	11.843	231.4137	617.64	85.61
3	15.908	14.7764	30.12	5.47
4	19.646	4.9919	8.75	1.85

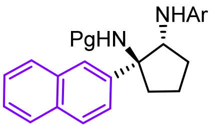

PG = 2-ClC_6H_4CO
Ar = 2,5-$(OMe)_2Ph$

7l: white solid; Mp 177.7 - 178.2 °C; 40.5 mg, 81% yield; 93% ee; dr > 19:1; $[\alpha]_D^{22}$ +11.5 (*c* 1.0, $CHCl_3$); ^1H NMR (300 MHz, Methylene Chloride-d_2) δ 7.96 (d, *J* = 2.0 Hz, 1H), 7.92 – 7.82 (m, 3H), 7.62 (dd, *J* = 8.7, 2.0 Hz, 1H), 7.56 – 7.48(m, 3H), 7.40 – 7.24 (m, 3H), 7.06 (s, 1H), 6.55 (d, *J* = 8.6 Hz, 1H), 6.32 (d, *J* = 2.8 Hz, 1H), 6.09 (dd, *J* = 8.6, 2.8 Hz, 1H), 4.23 (m, 1H), 3.86 (s, 1H), 3.68 (s, 3H), 3.47 (s, 3H), 3.20 – 3.06 (m, 1H), 2.69 – 2.55 (m, 1H), 2.24 – 2.05 (m, 3H), 1.50 – 1.37 (m, 1H); ^{13}C NMR (75 MHz, $CDCl_3$) δ 165.5, 154.7, 141.6, 138.1, 137.6, 135.2, 133.1, 132.7, 131.2, 130.6, 130.2, 130.1, 128.3, 127.8, 127.4, 127.0, 126.3, 126.0, 125.9, 125.0, 110.5, 99.3, 98.6, 67.9, 61.8, 55.9, 55.5, 34.9, 28.6, 19.9; HRMS (ESI) m/z 501.1935 ($M+H^+$), calc. for $C_{30}H_{30}ClN_2O_3^+$ 501.1939.

The ee was determined by HPLC analysis: CHIRALPAK IF (4.6 mm i.d. x 250 mm); Hexane/2-propanol = 60/40; flow rate: 1.0 mL/min; 25 °C; 210 nm; retention time: 14.8 min (major) and 17.8 min (minor).

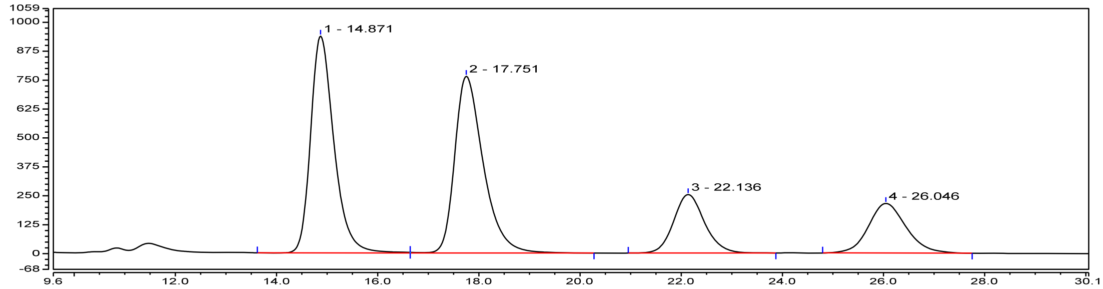

Entry	Retention Time	Area	Height	%Area
1	14.871	501.4384	936.70	36.74
2	17.751	499.8335	763.70	36.63
3	22.136	179.4012	252.80	13.15
4	26.046	184.0268	214.23	13.48

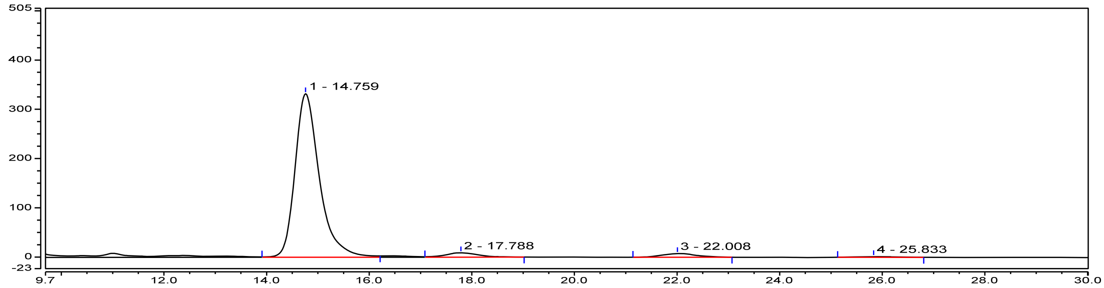

Entry	Retention Time	Area	Height	%Area
1	14.759	182.8219	331.95	93.18
2	17.788	6.4814	9.01	3.30
3	22.008	5.6387	7.56	2.87
4	25.833	1.2655	1.64	0.64

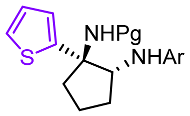

PG = 2-ClC$_6$H$_4$CO
Ar = 2,5-(OMe)$_2$Ph

7m: white solid; Mp 56.1 - 57.0 °C; 36.0 mg, 79% yield; 93% ee; dr > 19:1; $[\alpha]_D^{22}$ –10.4 (*c* 1.0, CHCl$_3$); ^{1}H NMR (300 MHz, Methylene Chloride-*d*$_2$) δ 7.54 – 7.49 (m, 1H), 7.37 – 7.32 (m, 2H), 7.32 – 7.27 (m, 2H), 7.10 (dd, *J* = 3.6, 1.3 Hz, 1H), 7.05 (dd, *J* = 5.0, 3.6 Hz, 1H), 6.90 (s, 1H), 6.61 (d, *J* = 8.6 Hz, 1H), 6.28 (d, *J* = 2.7 Hz, 1H), 6.14 – 6.06 (m, 1H), 4.19 – 4.08 (m, 2H), 3.66 (s, 3H), 3.65 (s, 3H), 2.97 –2.86 (m, 1H), 2.60 – 2.50 (m, 1H), 2.26 – 2.16 (m, 1H), 2.05 – 1.96 (m, 2H), 1.49 – 1.38 (m, 1H); ^{13}C NMR (75 MHz, CD$_2$Cl$_2$) δ 165.2, 154.4, 144.9, 141.3, 137.8, 135.2, 130.7, 130.0, 129.6, 129.2, 126.6, 126.4, 124.8, 124.2, 110.3, 99.0, 98.1, 65.9, 61.3, 55.7, 54.9, 36.6, 27.8, 18.8; HRMS (ESI) m/z 457.1340 (M+H$^+$), calc. for $C_{24}H_{26}ClN_2O_3S^+$ 457.1347.

The ee was determined by HPLC analysis: CHIRALPAK IF (4.6 mm i.d. x 250 mm); Hexane/2-propanol = 60/40; flow rate: 1.0 mL/min; 25 °C; 210 nm; retention time: 14.8 min (major) and 17.8 min (minor).

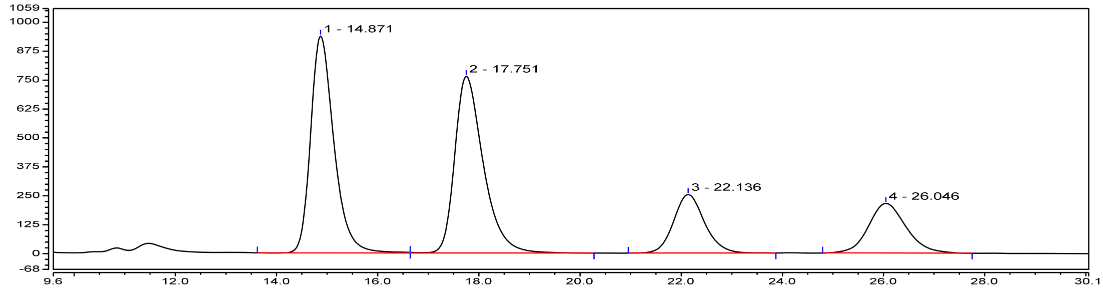

Entry	Retention Time	Area	Height	%Area
1	14.871	501.4384	936.70	36.74
2	17.751	499.8335	763.70	36.63
3	22.136	179.4012	252.80	13.15
4	26.046	184.0268	214.23	13.48

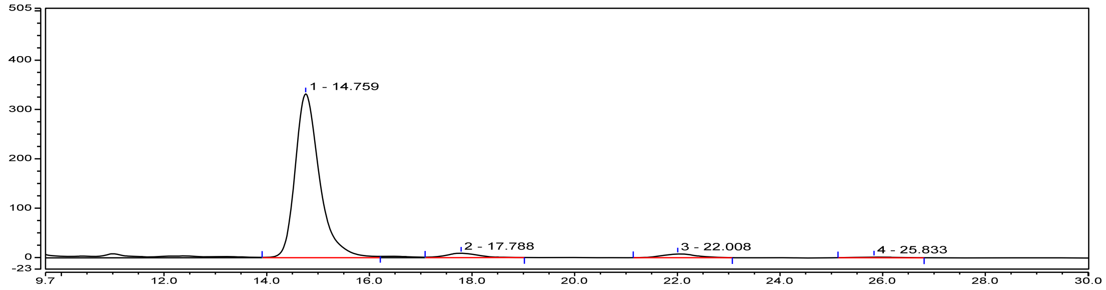

Entry	Retention Time	Area	Height	%Area
1	14.759	182.8219	331.95	93.18
2	17.788	6.4814	9.01	3.30
3	22.008	5.6387	7.56	2.87
4	25.833	1.2655	1.64	0.64

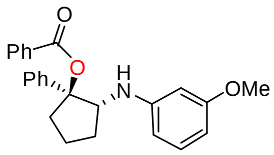

9: yellow solid; Mp 83.2 - 83.9 °C; 23.2 mg, 60% yield; 80% ee; dr = 7:1; $[\alpha]_D^{22}$ +15.3 (*c* 1.0, $CHCl_3$); ^1H NMR (300 MHz, $CDCl_3$) δ 7.99 (dd, *J* = 23.6, 7.6 Hz, 2H), 7.54 (t, *J* = 7.4 Hz, 1H), 7.40 (d, *J* = 7.6 Hz, 2H), 7.38 – 7.25 (m, 5H), 7.07 (dt, *J* = 34.4, 8.1 Hz, 1H), 6.45 4.01 (m, 3H), 4.47 (dd, *J* = 9.9, 6.5 Hz, 1H), 3.75 (d, *J* = 35.2 Hz, 3H), 3.17 – 3.03 (m, 1H), 2.57 – 2.42 (m, 1H), 2.20 – 1.82 (m, 3H), 1.58 – 1.39 (m, 1H); ^{13}C NMR (75 MHz, $CDCl_3$) δ 165.5, 160.8, 148.6, 139.0, 132.8, 130.6, 129.9, 129.8, 128.2, 128.2, 127.8, 126.4, 107.0, 102.4, 100.0, 91.8, 62.3, 55.1, 34.1, 28.5, 19.2; HRMS (ESI) m/z 410.1725 ($M+Na^+$), calc. for $C_{25}H_{25}NO_3Na$ 410.1727.

The ee was determined by HPLC analysis: CHIRAL AD-H (4.0 mm i.d. x 100 mm); Hexane/2-propanol = 95/5; flow rate: 1.0 mL/min; 25 °C; 230 nm; retention time: 9.8 min (major) and 14.1 min (minor).

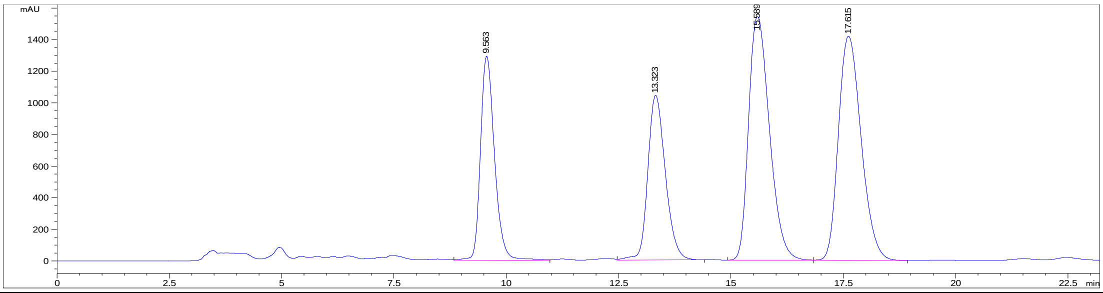

Entry	Retention Time	Area	Height	%Area
1	9. 563	28044. 9	1292. 7	17. 973
2	13. 323	28254. 2	1041. 2	18. 107
3	15. 589	49777. 9	1541	31. 900
4	17. 615	49964. 4	1418. 8	32. 020

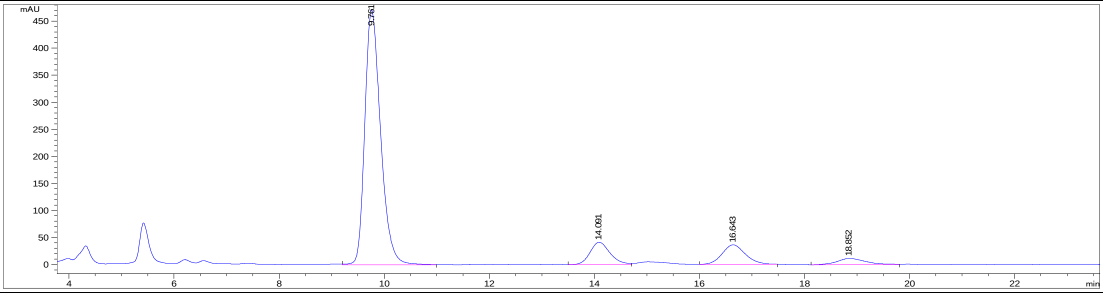

Entry	Retention Time	Area	Height	%Area
1	9. 761	9844. 6	471. 3	78. 807
2	14. 091	1090. 4	41. 2	8. 729
3	16. 643	1119. 8	36. 2	8. 964
4	18. 852	437. 2	11. 5	3. 500

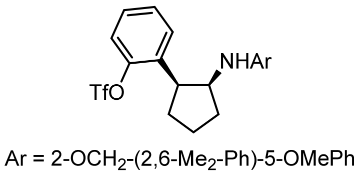

11a: yellow oil; 43.9 mg, 80% yield; 84% ee; dr > 19:1; $[\alpha]_D^{22}$ –24.5 (*c* 1.0, $CHCl_3$); ^1H NMR (300 MHz, Chloroform-*d*) δ 7.24 – 7.18 (m, 2H), 7.13 – 7.07 (m, 4H), 6.91 – 6.84 (m, 1H), 6.73 – 6.67 (m, 1H), 6.12 (d, *J* = 7.4 Hz, 2H), 4.83 (s, 2H), 4.02 –3.96 (m, 1H), 3.71 (s, 3H), 3.61 – 3.55 (m, 1H), 2.26 (s, 6H), 2.22 – 2.17 (m, 1H), 2.06 – 1.99 (m, 2H), 1.93 – 1.70 (m, 3H); ^{13}C NMR (75 MHz, $CDCl_3$) δ 154.8, 148.1, 140.9, 138.2, 133.3, 133.2, 129.6, 128.3, 128.2, 128.1, 128.0, 121.0, 118.5 (q, *J* = 320.1 Hz), 111.5, 99.6, 98.5, 65.6, 55.5, 42.8, 33.2, 29.6, 22.7, 19.5; ^{19}F NMR (376 MHz, Chloroform-*d*) δ –73.9; HRMS (ESI) m/z 550.1866 (M+H$^+$), calc. for $C_{28}H_{31}F_3NO_5S^+$ 550.1870.

The ee was determined by HPLC analysis: CHIRALPAK IG (x 2) (4.6 mm i.d. x 250 mm); Hexane/2-propanol = 97/3; flow rate: 1.0 mL/min; 25 °C; 210 nm; retention time: 11.4 min (major) and 13.0 min (minor).

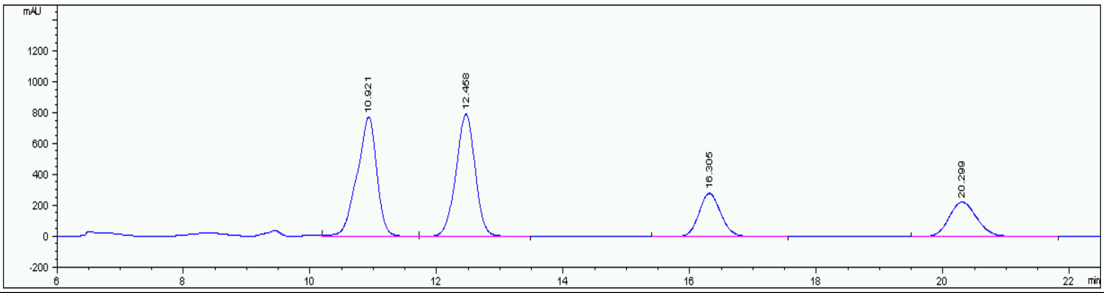

Entry	Retention Time	Area	Height	%Area
1	10.921	16915.8	772.7	35.634
2	12.458	16742	792.3	35.268
3	16.305	6866.8	281.4	14.465
4	20.299	6946.6	224.3	14.633

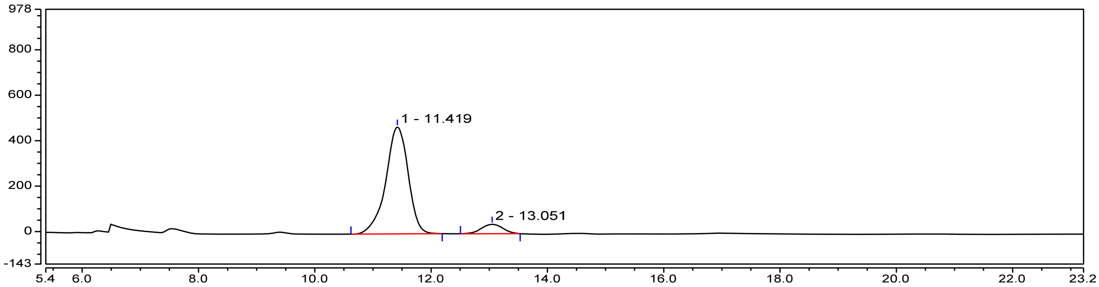

Entry	Retention Time	Area	Height	%Area
1	11.419	210.1115	469.58	92.23
2	13.051	17.6986	40.99	7.77

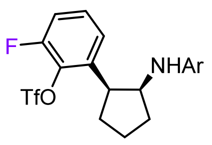

Ar = 2-OCH_2-(2,6-Me_2-Ph)-5-OMePh

11b: yellow oil; 42.5 mg, 75% yield; 83% ee; dr > 19:1; $[\alpha]_D^{22}$ –26.8 (*c* 1.0, $CHCl_3$); ^1H NMR (300 MHz, Chloroform-*d*) δ 7.22 (dd, *J* = 8.2, 6.7 Hz, 1H), 7.10 (d, *J* = 7.5 Hz, 2H), 6.96 – 6.85 (m, 2H), 6.81 – 6.72 (m, 1H), 6.70 (d, *J* = 8.5 Hz, 1H), 6.15 – 6.02 (m, 2H), 4.84 (s, 2H), 4.06 – 3.99 (m, 1H), 3.94 (s, 1H), 3.71 (s, 3H), 3.64 – 3.54 (m, 1H), 2.28 (s, 6H), 2.24 – 2.16 (m, 1H), 2.09 – 2.01 (m, 1H), 2.00 – 1.86 (m, 2H), 1.84 – 1.69 (m, 2H); ^{13}C NMR (75 MHz, $CDCl_3$) δ 154.8, 153.5 (d, *J* = 252.4 Hz), 140.6, 138.5, 138.2, 136.7 (d, *J* = 0.9 Hz), 135.3 (d, *J* = 13.4 Hz), 133.4, 128.4 (d, *J* = 7.9 Hz), 128.3, 128.1, 124.4 (d, *J* = 3.4 Hz), 118.5 (q, *J* = 320.5 Hz), 115.2 (d, *J* = 18.7 Hz), 111.4, 99.0, 97.9, 65.5, 55.9, 55.4, 42.9 (d, *J* = 1.8 Hz), 33.6, 30.1, 22.7, 19.5; ^{19}F NMR (376 MHz, Chloroform-*d*) δ –73.0, –126.3; HRMS (ESI) m/z 568.1771 (M+H$^+$), calc. for $C_{28}H_{30}F_4NO_5S^+$ 568.1775.

The ee was determined by HPLC analysis: CHIRALPAK IG (x 2) (4.6 mm i.d. x 250 mm); Hexane/2-propanol = 98/2; flow rate: 1.0 mL/min; 25 °C; 210 nm; retention time: 12.1 min (major) and 13.2 min (minor).

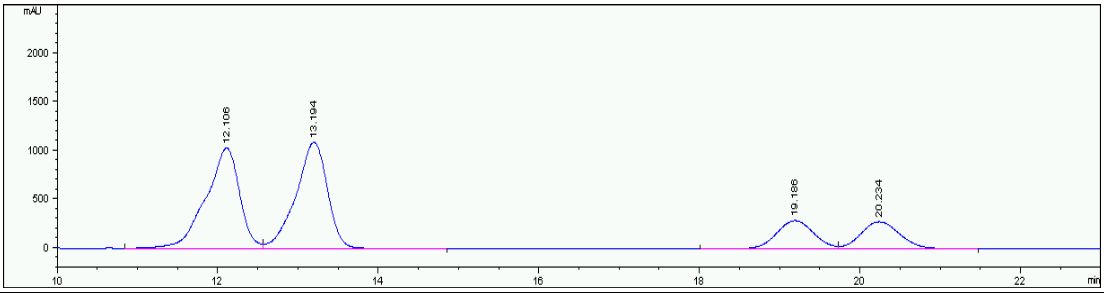

Entry	Retention Time	Area	Height	%Area
1	12.106	30622.1	1037.1	38.388
2	13.194	30258.5	1096.7	37.932
3	19.186	9452.6	291.9	11.850
4	20.234	9437.8	279.8	11.831

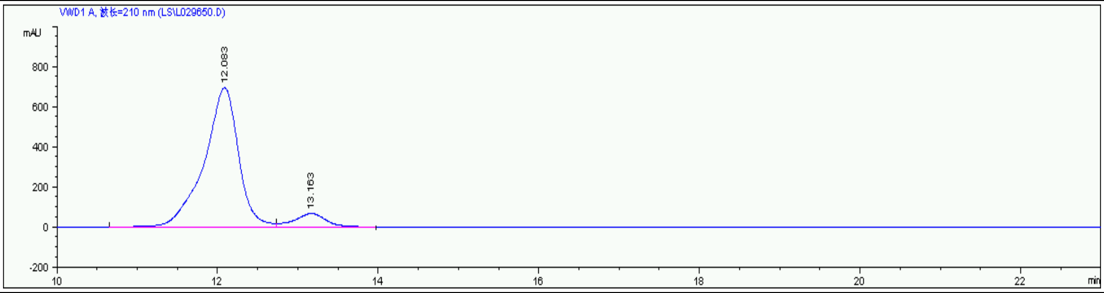

Entry	Retention Time	Area	Height	%Area
1	12.083	20795.2	696.1	91.443
2	13.163	1945.9	68.6	8.557

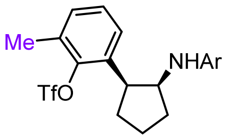

Ar = 2-OCH$_2$-(2,6-Me$_2$-Ph)-5-OMePh

11c: yellow oil; 41.1 mg, 73% yield; 83% ee; dr > 19:1; $[\alpha]_D^{22}$ –23.5 (*c* 1.0, $CHCl_3$); ^{1}H NMR (300 MHz, Chloroform-*d*) δ 7.24 – 7.18 (m, 1H), 7.09 (d, *J* = 7.6 Hz, 2H), 7.06 – 7.01 (m, 1H), 6.96 – 6.90 (m, 1H), 6.76 – 6.66 (m, 2H), 6.10 (d, *J* = 7.4 Hz, 2H), 4.83 (d, *J* = 1.7 Hz, 2H), 4.00 – 3.93 (m, 1H), 3.71 (s, 3H), 3.68 – 3.58 (m, 1H), 2.28 (d, *J* = 6.3 Hz, 9H), 2.16 (dt, *J* = 9.0, 5.2 Hz, 1H), 2.07 – 1.98 (m, 2H), 1.93 – 1.72 (m, 3H); ^{13}C NMR (75 MHz, $CDCl_3$) δ 154.8, 146.3, 141.0, 138.1, 133.9, 133.3, 131.4, 130.6, 128.3, 128.1, 127.7, 127.2, 118.6 (q, *J* = 319.9 Hz), 111.4, 99.4, 98.6, 65.6, 56.2, 55.4, 43.0, 33.2, 30.0, 22.7, 19.5, 17.3; ^{19}F NMR (376 MHz, Chloroform-*d*) δ –73.4; HRMS (ESI) m/z 564.2023 (M+H$^+$), calc. for $C_{29}H_{33}F_3NO_5S^+$ 564.2026.

The ee was determined by HPLC analysis: CHIRALPAK IG (x 2) (4.6 mm i.d. x 250 mm); Hexane/2-propanol = 95/5; flow rate: 1.0 mL/min; 25 °C; 210 nm; retention time: 9.2 min (major) and 10.5 min (minor).

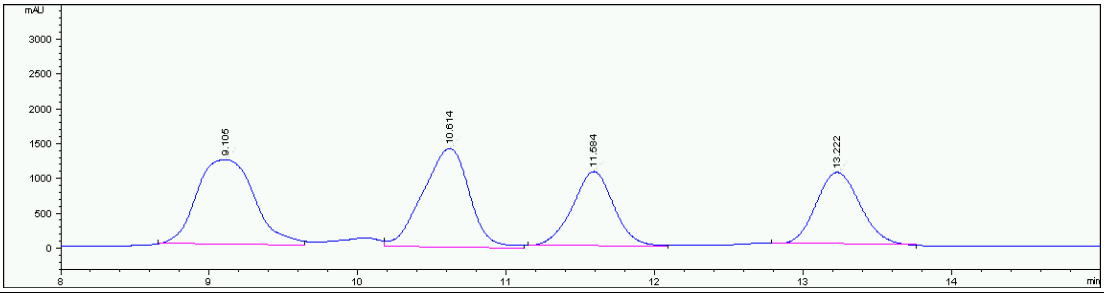

Entry	Retention Time	Area	Height	%Area
1	9.105	32824.8	1219.4	31.046
2	10.614	30914.3	1417.6	29.239
3	11.584	21120.2	1074.7	19.976
4	13.222	20870.6	1026.4	19.740

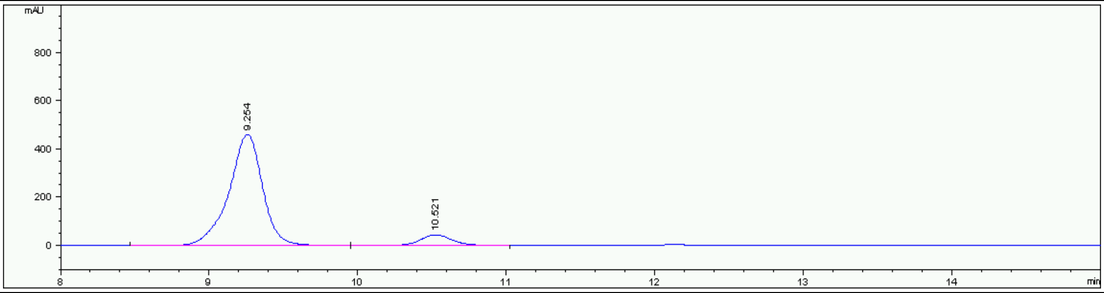

Entry	Retention Time	Area	Height	%Area
1	9.254	7385.3	463.2	90.881
2	10.521	741.1	46	9.119

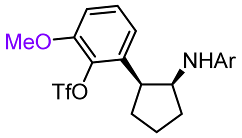

Ar = 2-OCH$_2$-(2,6-Me$_2$-Ph)-5-OMePh

11d: yellow oil; 37.0 mg, 64% yield; 80% ee; dr > 19:1; $[\alpha]_D^{22}$ –49.2 (*c* 1.0, $CHCl_3$); ^{1}H NMR (300 MHz, Chloroform-*d*) δ 7.30 – 7.22 (m, 1H), 7.14 (d, *J* = 7.5 Hz, 2H), 6.82 – 6.68 (m, 4H), 6.11 (d, *J* = 7.3 Hz, 2H), 4.87 (s, 2H), 4.11 – 3.96 (m, 2H), 3.85 (s, 3H), 3.75 (s, 3H), 3.60 (q, *J* = 8.5 Hz, 1H), 2.32 (s, 6H), 2.21 (d, *J* = 8.8 Hz, 1H), 2.09 – 1.98 (m, 2H), 1.96 – 1.67 (m, 3H); ^{13}C NMR (75 MHz, $CDCl_3$) δ 154.8, 150.8, 140.7, 138.8, 138.2, 137.5, 134.7, 133.5, 128.2, 128.1, 128.0, 120.4, 118.7 (q, *J* = 320.6 Hz), 111.5, 111.1, 98.7, 97.6, 65.5, 55.9, 55.7, 55.4, 43.0, 33.4, 29.5, 22.6, 19.6; ^{19}F NMR (376 MHz, Chloroform-*d*) δ -72.8; HRMS (ESI) m/z 580.1981 (M+H$^+$), calc. for $C_{29}H_{33}F_3NO_6S^+$ 580.1975.

The ee was determined by HPLC analysis: CHIRAL INB (x 2) (4.6 mm i.d. x 250 mm); Hexane/2-propanol = 95/5; flow rate: 1.0 mL/min; 25 °C; 210 nm; retention time: 17.0 min (major) and 20.8 min (minor).

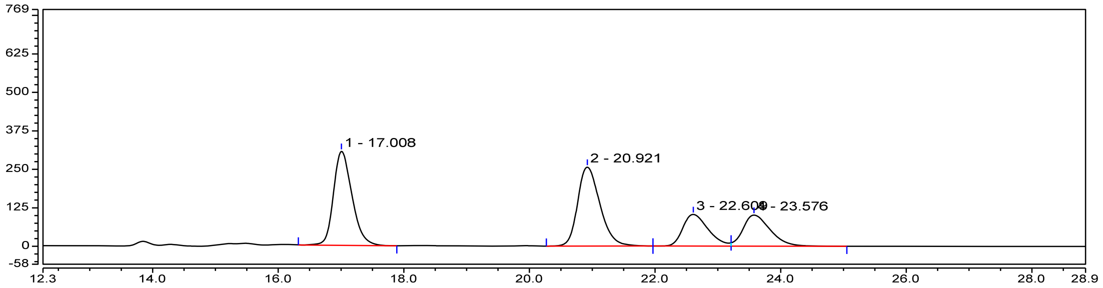

Entry	Retention Time	Area	Height	%Area
1	17.008	105.3286	305.67	34.00
2	20.921	105.6075	256.68	34.09
3	22.609	48.1638	102.92	15.55
4	23.576	50.6478	101.07	16.35

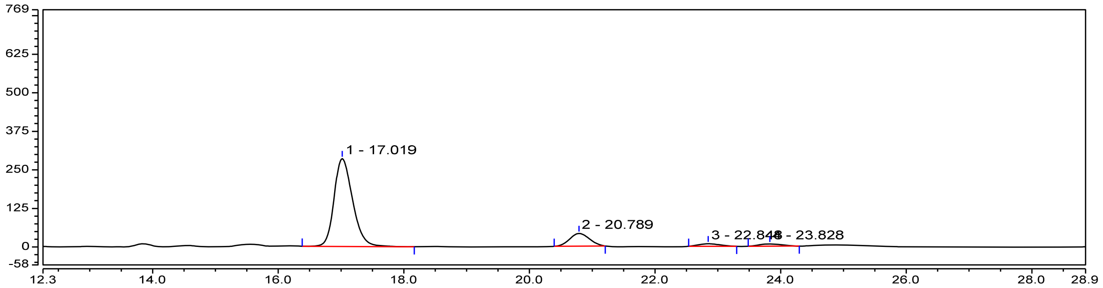

Entry	Retention Time	Area	Height	%Area
1	17.019	97.4785	284.54	82.17
2	20.789	15.0304	41.18	12.67
3	22.848	3.2060	8.14	2.70
4	23.828	2.9205	7.43	2.46

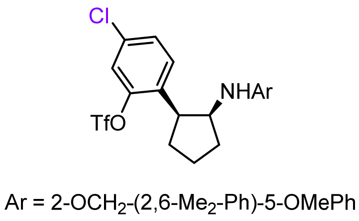

11e: yellow oil; 40.8 mg, 72% yield; 83% ee; dr > 19:1; $[\alpha]_D^{22}$ –29.5 (*c* 1.0, $CHCl_3$); ^{1}H NMR (300 MHz, Chloroform-*d*) δ 7.35 – 7.27 (m, 1H), 7.22 – 7.09 (m, 4H), 6.88 (dd, *J* = 8.4, 2.1 Hz, 1H), 6.76 (d, *J* = 8.5 Hz, 1H), 6.22 – 6.00 (m, 2H), 4.92 (s, 2H), 4.00 (d, *J* = 23.3 Hz, 2H), 3.77 (s, 3H), 3.56 (q, *J* = 7.6, 7.1 Hz, 1H), 2.33 (s, 6H), 2.28 – 2.19 (m, 1H), 2.10 – 1.75 (m, 5H). ^{13}C NMR (75 MHz, $CDCl_3$) δ 154.9, 147.8, 140.8, 138.5, 138.1, 133.3, 133.0 132.4, 130.4, 128.4, 128.2, 121.5, 118.5 (q, *J* = 320.2 Hz), 111.4, 99.0, 98.0, 65.6, 55.8, 55.5, 42.6, 33.4, 29.7, 22.5, 19.4; ^{19}F NMR (376 MHz, Chloroform-d) δ –73.7; HRMS (ESI) m/z 586.1772 (M+H$^+$), calc. for $C_{28}H_{30}ClF_3NO_5S^+$ 586.1775.

The ee was determined by HPLC analysis: CHIRALCEL OD-H (x 2) (4.6 mm i.d. x 250 mm); Hexane/2-propanol = 98/2; flow rate: 1.0 mL/min; 25 °C; 210 nm; retention time: 11.0 min (minor) and 11.6 min (major).

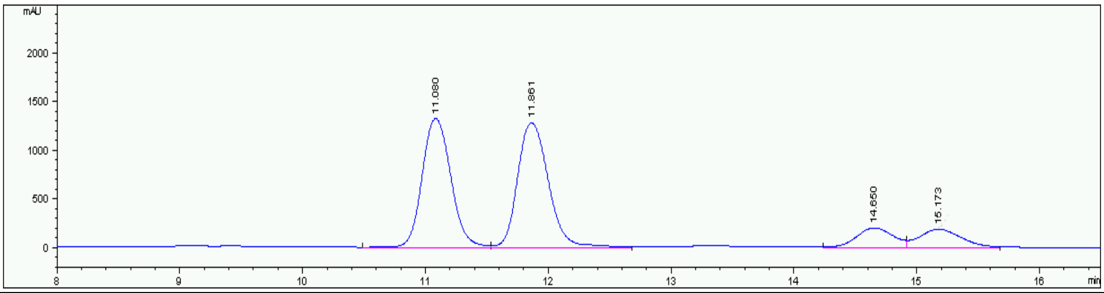

Entry	Retention Time	Area	Height	%Area
1	11.08	21971.1	1332.2	40.904
2	11.861	22690.9	1287.5	42.244
3	14.65	4426	207.1	8.240
4	15.173	4625.6	192.7	8.612

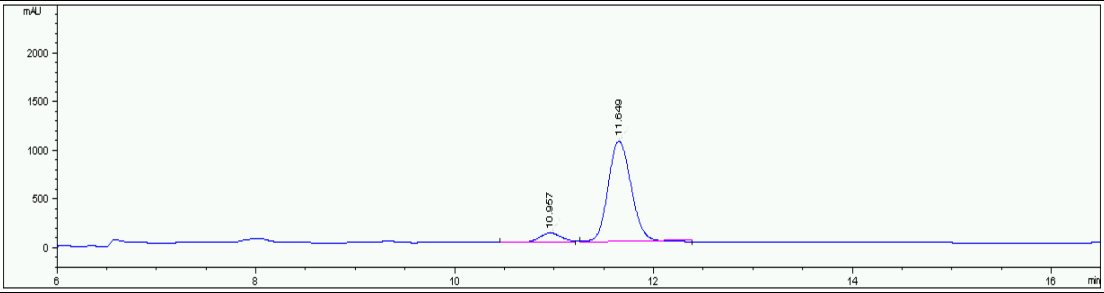

Entry	Retention Time	Area	Height	%Area
1	10.957	1830.5	103.4	9.858
2	11.649	16737.3	1036	90.142

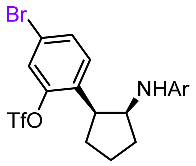

11f: yellow oil; 46.4 mg, 74% yield; 85% ee; dr > 19:1; $[\alpha]_D^{22}$ –56.5 (*c* 1.0, $CHCl_3$); ^1H NMR (300 MHz, Chloroform-*d*) δ 7.35 – 7.25(m, 2H), 7.19 (d, *J* = 7.5 Hz, 2H), 7.10 – 6.99 (m, 2H), 6.76 (d, *J* = 8.5 Hz, 1H), 6.24 – 6.06 (m, 2H), 4.92 (s, 2H), 4.09 – 4.07 (m, 1H), 3.97 (s, 1H), 3.76 (s, 3H), 3.55 (dt, *J* = 10.1, 7.0 Hz, 1H), 2.34 (s, 6H), 2.27 – 2.15 (m, 1H), 2.08 – 1.72 (m, 5H); ^{13}C NMR (75 MHz, Chloroform-*d*) δ 154.8, 147.8, 140.8, 138.4, 138.1, 133.3, 132.9, 131.1, 130.7, 128.4, 128.2, 124.3, 120.4, 118.5 (q, *J* = 320.3 Hz), 111.4, 99.0, 98.0, 65.6, 55.8, 55.5, 42.7, 33.3, 29.6, 22.4, 19.4; ^{19}F NMR (376 MHz, Chloroform-*d*) δ –73.7; HRMS (ESI) m/z 628.0970 $(M+H^+)$, calc. for $C_{28}H_{30}BrF_3NO_5S^+$ 628.0975.

The ee was determined by HPLC analysis: CHIRALCEL OD-H (x 2) (4.6 mm i.d. x 250 mm); Hexane/2-propanol = 97/3; flow rate: 1.0 mL/min; 25 °C; 210 nm; retention time: 15.6min (minor) and 16.7 min (major).

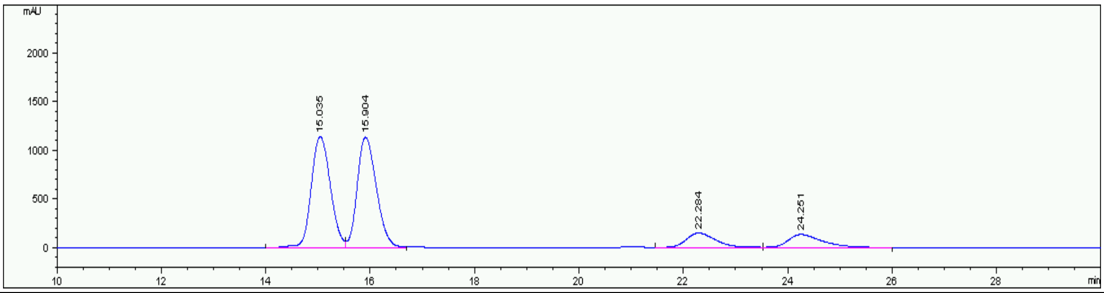

Entry	Retention Time	Area	Height	%Area
1	15.035	29400.1	1144	40.831
2	15.904	29266.4	1140.4	40.645
3	22.284	6521.2	154.3	9.057
4	24.251	6817.5	139.5	9.468

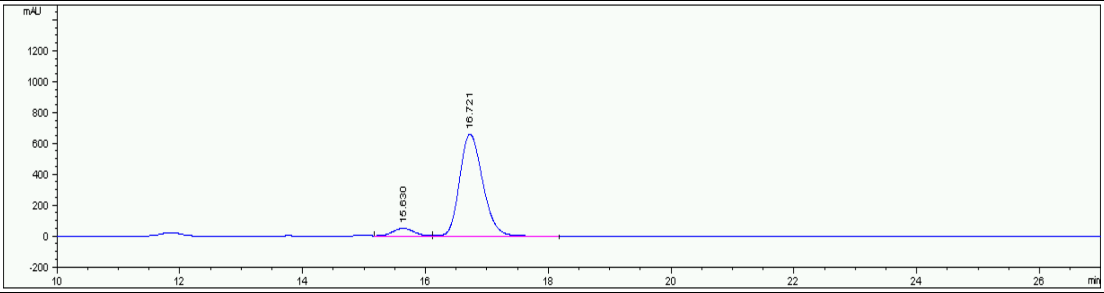

Entry	Retention Time	Area	Height	%Area
1	15.63	1392.7	53.4	7.367
2	16.721	17512.7	663.2	92.633

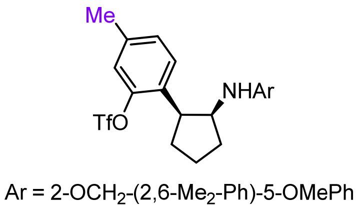

11g: yellow oil; 38.3 mg, 68% yield; 84% ee; dr > 19:1; $[\alpha]_D^{22}$ –7.8 (*c* 1.0, $CHCl_3$); ^{1}H NMR (300 MHz, Chloroform-*d*) δ 7.22 (dd, *J* = 8.3, 6.8 Hz, 1H), 7.10 (d, *J* = 7.5 Hz, 2H), 7.02 (d, *J* = 8.0 Hz, 1H), 6.96 – 6.90 (m, 1H), 6.72 – 6.62 (m, 2H), 6.13 – 6.03 (m 2H), 4.84 (s, 2H), 4.03 – 3.87 (m, 2H), 3.71 (s, 3H), 3.51 (q, *J* = 8.3 Hz, 1H), 2.32– 3.22 (m, 9H), 2.16 (d, *J* = 7.1 Hz, 1H), 2.03 – 1.93 (m, 2H), 1.90 – 1.67 (m, 3H); ^{13}C NMR (75 MHz, $CDCl_3$) δ 154.8, 147.9, 141.0, 138.4, 138.1, 133.3, 129.8, 129.3, 128.8, 128.3, 128.1, 121.6, 118.5 (q, *J* = 320.1 Hz), 111.6, 98.7, 65.6, 56.3, 55.5, 42.65, 32.9, 29.4, 22.5, 20.8, 19.4; ^{19}F NMR (376 MHz, Chloroform-d) δ –74.0; HRMS (ESI) m/z 564.2023 (M+H$^+$), calc. for $C_{29}H_{33}F_3NO_5S^+$ 564.2026.

The ee was determined by HPLC analysis: CHIRAL INB (x 2) (4.6 mm i.d. x 250 mm); Hexane/2-propanol = 97/3; flow rate: 1.0 mL/min; 25 °C; 210 nm; retention time: 14.2 min (minor) and 14.9 min (major).

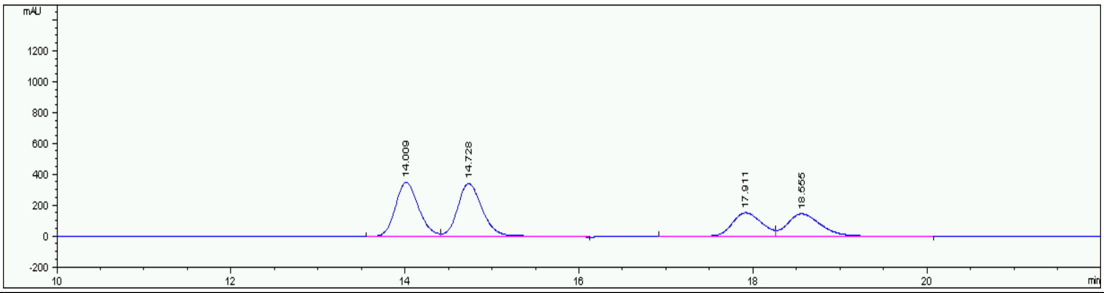

Entry	Retention Time	Area	Height	%Area
1	14.009	6671.4	353.6	30.516
2	14.728	7061.7	346.7	32.302
3	17.911	3896.7	156.1	17.824
4	18.555	4232	149.2	19.358

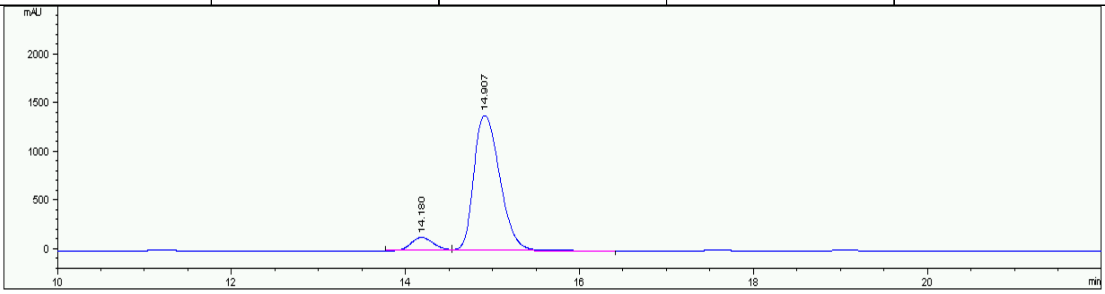

Entry	Retention Time	Area	Height	%Area
1	14.18	2624.4	139.8	8.207
2	14.907	29352.7	1394.3	91.793

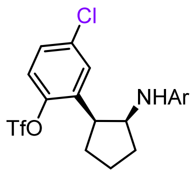

Ar = 2-OCH$_2$-(2,6-Me$_2$-Ph)-5-OMePh

11h: yellow oil; 38.9 mg, 66% yield; 75% ee; dr > 19:1; $[\alpha]_D^{22}$ –28.8 (*c* 1.0, $CHCl_3$); ^{1}H NMR (300 MHz, Chloroform-*d*) δ 7.30 (d, *J* = 2.7 Hz, 1H), 7.25 – 7.20 (m, 1H), 7.16 – 7.08 (m, 3H), 7.03 (d, *J* = 8.8 Hz, 1H), 6.74 (d, *J* = 8.5 Hz, 1H), 6.17 – 6.02 (m, 2H), 4.92 (s, 2H), 4.14 (s, 1H), 3.98 (s, 1H), 3.73 (s, 3H), 3.57 (dt, *J* = 9.6, 7.0 Hz, 1H), 2.35 (s, 6H), 2.31 – 2.21 (m, 1H), 2.16 – 2.07 (m, 1H), 2.00 – 1.87 (m, 2H), 1.82 – 1.68 (m, 2H); ^{13}C NMR (75 MHz, $CDCl_3$) δ 154.9, 146.4, 140.5, 138.6, 138.1, 136.1, 133.7, 133.3, 130.1, 128.4, 128.2, 127.9, 122.0, 118.5 (q, *J* = 320.2 Hz), 112.2, 99.4, 97.9, 66.0, 56.0, 55.4, 42.7, 33.7, 30.2, 22.5, 19.5; ^{19}F NMR (376 MHz, Chloroform-*d*) δ -72.9; HRMS (ESI) m/z 590.0771 (M+H$^+$), calc. for $C_{28}H_{30}ClF_3NO_5S^+$ 590.0777.

The ee was determined by HPLC analysis: CHIRAL MD (x 2) (4.6 mm i.d. x 250 mm); Hexane/2-propanol = 98/2; flow rate: 1.0 mL/min; 25 °C; 210 nm; retention time: 7.5 min (major) and 8.6 min (minor).

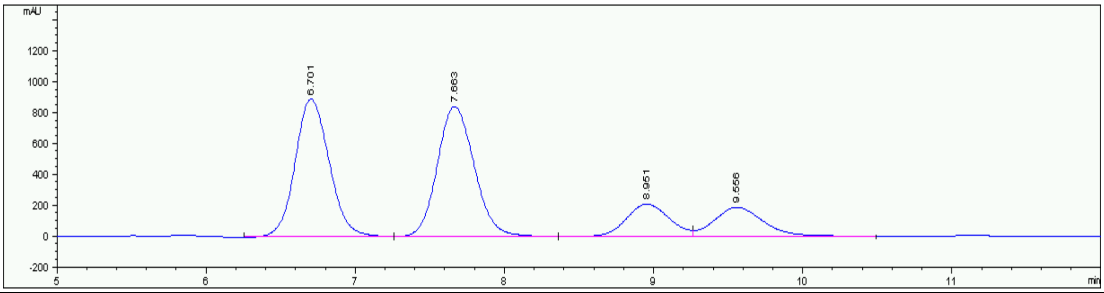

Entry	Retention Time	Area	Height	%Area
1	6.701	14299	894.6	37.430
2	7.663	14769.6	847.3	38.662
3	8.951	4439.7	213.8	11.622
4	9.556	4693.3	193.6	12.286

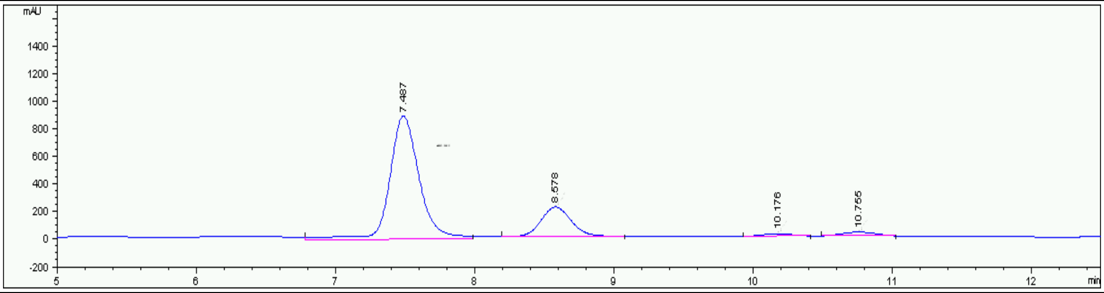

Entry	Retention Time	Area	Height	%Area
1	7.487	13465.1	895.9	77.646
2	8.578	3214.9	214	18.539
3	10.176	242.4	16.2	1.398
4	10.755	419.1	26.5	2.417

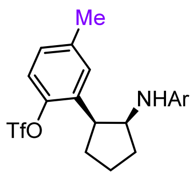

Ar = 2-OCH$_2$-(2,6-Me$_2$-Ph)-5-OMePh

11i: yellow oil; 37.7 mg, 67% yield; 83% ee; dr > 19:1; $[\alpha]_D^{22}$ +4.3 (*c* 1.0, $CHCl_3$); ^{1}H NMR (300 MHz, Chloroform-*d*) δ 7.21 – 7.16 (m, 1H), 7.10 – 7.04 (m, 3H), 6.96 – 6.85 (m, 2H), 6.71 (d, *J* = 8.6 Hz, 1H), 6.23 – 6.07 (m, 2H), 4.83 (s, 2H), 4.06 – 3.98 (m, 1H), 3.73 – 3.68 (m, 3H), 3.58 – 3.49 (m, 1H), 2.25 (d, *J* = 2.0 Hz, 6H), 2.22 – 2.16 (m, 1H), 2.04 (s, 5H), 1.97 – 1.89 (m, 1H), 1.86 – 1.71 (m, 2H); ^{13}C NMR (75 MHz, $CDCl_3$) δ 154.8, 146.2, 140.7, 138.7, 138.1, 138.1, 133.2, 133.0, 130.3 128.5, 128.3, 128.2, 120.6, 111.6, 98.7, 97.9, 65.7, 55.6, 55.4, 42.7, 33.4, 29.6, 22.6, 20.8, 19.4; ^{19}F NMR (376 MHz, Chloroform-*d*) δ –73.9; HRMS (ESI) m/z 564.2021 (M+H$^+$), calc. for $C_{29}H_{33}F_3NO_5S^+$ 564.2026.

The ee was determined by HPLC analysis: CHIRALCEL OD-H (x 2) (4.6 mm i.d. x 250 mm); Hexane/2-propanol = 98/2; flow rate: 1.0 mL/min; 25 °C; 210 nm; retention time: 14.7 min (major) and 17.1 min (minor).

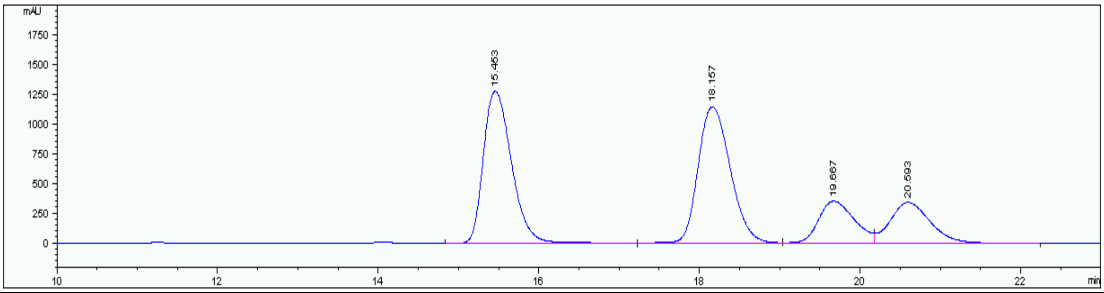

Entry	Retention Time	Area	Height	%Area
1	15.453	31637.8	1277.4	36.201
2	18.157	32292.1	1148.6	36.949
3	19.667	11203	355.7	12.819
4	20.593	12262.8	345.9	14.031

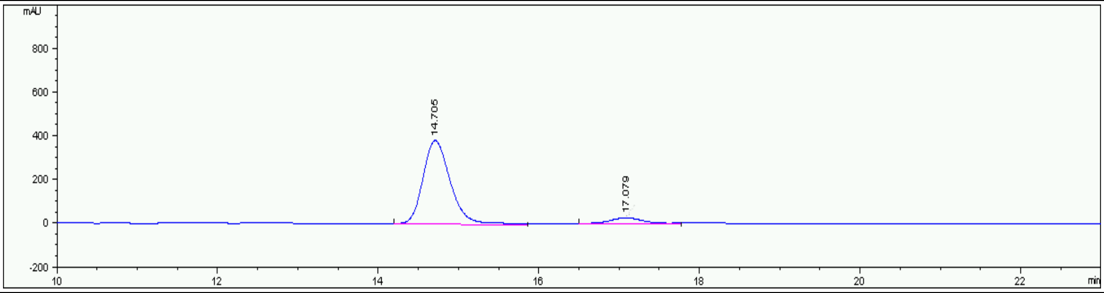

Entry	Retention Time	Area	Height	%Area
1	14.705	9396.1	386.2	91.335
2	17.079	891.4	28.6	8.665

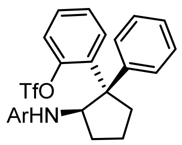

Ar = 2-OBn-5-OMePh

13a: white solid; Mp 80.6 - 81.8 °C; 37.6 mg, 63% yield; 92% ee; dr = 6:1; $[\alpha]_D^{22}$ –16.3 (*c* 1.0, $CHCl_3$); ^{1}H NMR (300 MHz, Chloroform-*d*) δ 7.63 (dd, *J* = 6.1, 3.5 Hz, 1H), 7.33 – 7.27 (m, 5H), 7.26 – 7.23 (m, 1H), 7.15 – 6.95 (m, 7H), 6.72 (d, *J* = 8.6 Hz, 1H), 6.41 (d, *J* = 2.8 Hz, 1H), 6.13 (dd, *J* = 8.7, 2.8 Hz, 1H), 4.89 – 4.73 (m, 2H), 4.33 – 4.19 (m, 1H), 4.08 (d, *J* = 10.1 Hz, 1H), 3.79 (d, *J* = 3.0 Hz, 3H), 3.20 – 3.07 (m, 1H), 2.35 – 2.23 (m, 1H), 2.15 – 1.98 (m, 2H), 1.93 – 1.82 (m, 1H), 1.32 – 1.20 (m, 1H); ^{13}C NMR (75 MHz, $CDCl_3$) δ 155.1, 149.1, 141.4, 140.8, 140.8, 138.3, 136.8, 129.0, 128.3, 128.2, 128.1, 127.9, 127.8, 127.7, 127.5, 126.8, 120.6, δ 118.0 (q, *J* = 319.8 Hz), 111.8, 98.2, 97.8, 71.0, 58.2, 55.5, 54.1, 36.2, 28.0, 19.6; ^{19}F NMR (376 MHz, Chloroform-*d*) δ –74.4, –74.8; HRMS (ESI) m/z 598.1864 (M+H$^+$), calc. for $C_{32}H_{31}NO_5S^+$ 598.1870.

The ee was determined by HPLC analysis: CHIRALPAK IF (4.6 mm i.d. x 250 mm); Hexane/2-propanol = 98/2; flow rate: 1.0 mL/min; 25 °C; 210 nm; retention time: 7.1 min (major) and 7.7 min (minor).

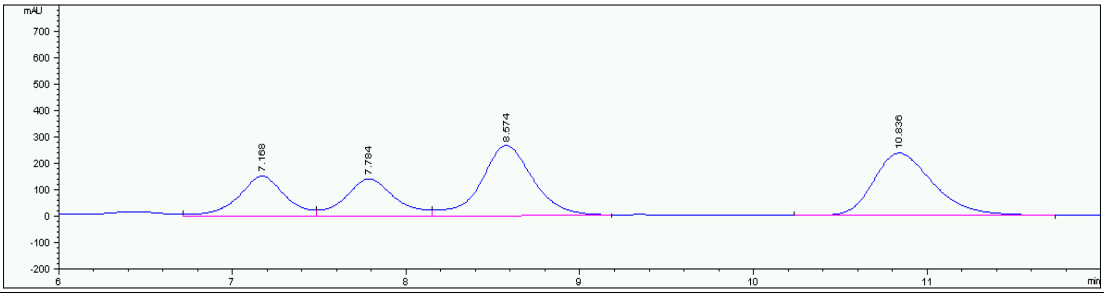

Entry	Retention Time	Area	Height	%Area
1	7.168	2849.1	152.2	16.791
2	7.784	2806.2	141.7	16.538
3	8.574	5653.7	267.7	33.319
4	10.836	5659.1	236	33.352

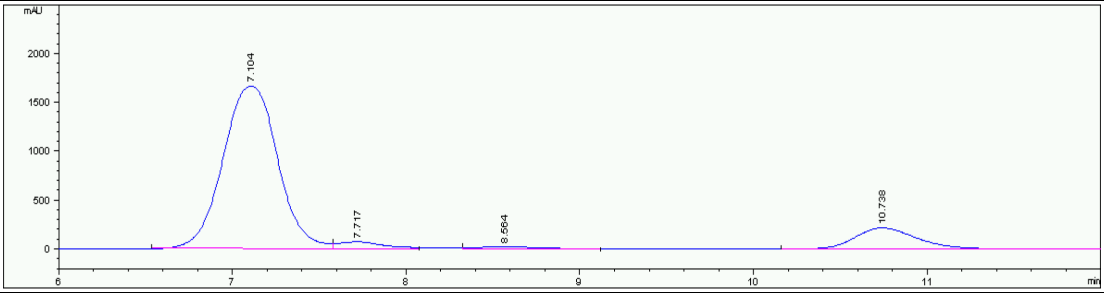

Entry	Retention Time	Area	Height	%Area
1	7.104	36407.2	1662.4	83.193
2	7.717	1443.5	75.6	3.299
3	8.564	585.5	21.2	1.338

4	10.738	5326	215.1	12.170

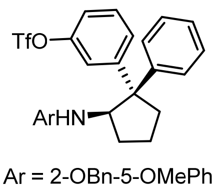

13b: yellow oil; 47.8 mg, 80% yield; 91% ee; dr = 3:1; $[\alpha]_D^{22}$ –39.0 (*c* 1.0, $CHCl_3$); ^1H NMR (300 MHz, Chloroform-*d*) δ 7.54 – 7.41 (m, 7H), 7.39 – 7.28 (m, 4H), 7.26 – 7.05 (m, 3H), 6.89 (dd, *J* = 8.6, 2.2 Hz, 1H), 6.64 – 6.55 (m, 1H), 6.32 (dd, *J* = 8.7, 2.6 Hz, 1H), 5.07 – 4.90 (m, 2H), 4.65 – 4.43 (m, 1H), 4.34 – 4.13 (m, 1H), 3.96 (s, 3H), 3.01 – 2.77 (m, 1H), 2.56 – 2.37 (m, 2H), 2.20 – 2.06 (m, 1H), 2.03 – 1.88 (m, 1H), 1.65 – 1.57 (m, 1H); ^{13}C NMR (75 MHz, $CDCl_3$) δ 155.1, 155.1, 151.9, 149.5, 149.4, 147.8, 147.3, 142.9, 140.8, 138.4, 137.0, 129.8, 129.5, 129.4, 128.6, 128.4, 128.3, 128.3, 128.2, 127.8, 127.7, 127.6, 127.5, 127.1, 127.0, 126.7, 126.3, 121.0, 120.5, 119.0, 118.8, 118.8 (q, *J* = 321.1 Hz),112.0, 111.9, 98.3, 98.2, 98.2, 71.0, 58.6, 57.4, 57.1, 55.4, 37.7, 29.8, 29.4, 26.9, 19.8; ^{19}F NMR (376 MHz, $CDCl_3$) δ –72.77, –72.78; HRMS (ESI) m/z 598.1863 (M+H$^+$), calc. for $C_{32}H_{31}NO_5S^+$ 598.1870.

The ee was determined by HPLC analysis: Lux Amylose-1 (4.6 mm i.d. x 250 mm); Hexane/2-propanol = 99/1; flow rate: 1.0 mL/min; 25 °C; 210 nm; retention time: 10.9 min (minor) and 11.9 min (major).

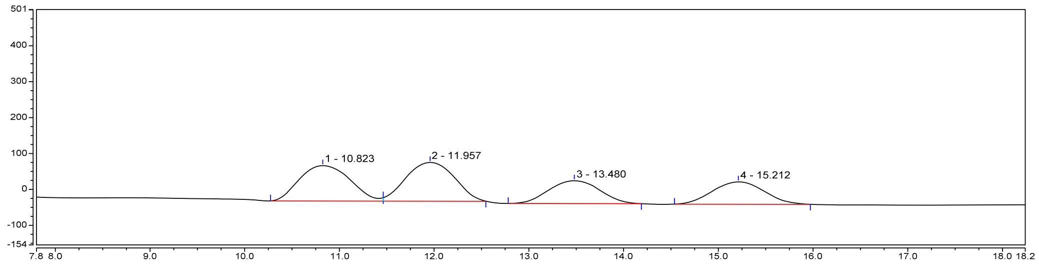

Entry	Retention Time	Area	Height	%Area
1	10.823	60.8956	98.00	29.93
2	11.957	63.7310	107.33	31.32
3	13.480	39.5368	63.35	19.43
4	15.212	39.3250	61.93	19.33

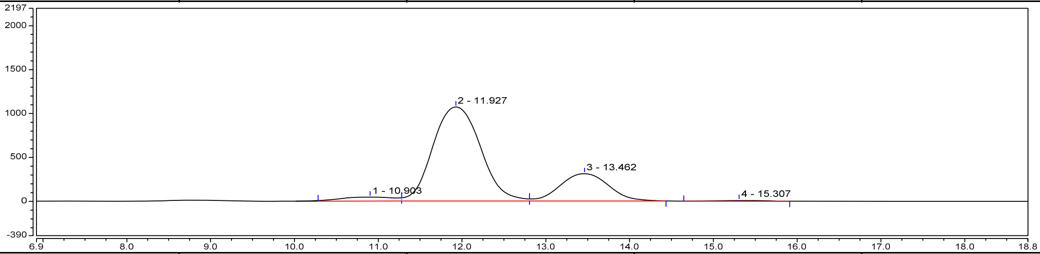

Entry	Retention Time	Area	Height	%Area
1	10.903	33.6268	45.39	3.53
2	11.927	705.2760	1070.66	74.08

3	13.462	208.0867	311.16	21.86
4	15.307	5.1017	8.68	0.54

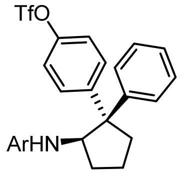

Ar = 2-OBn-5-OMePh

13c: white solid; Mp 66.5 - 67.8 °C; 49.0 mg, 82% yield; 97% ee; dr = 4:1; $[\alpha]_D^{22}$ –32.8 (*c* 1.0, $CHCl_3$); ^{1}H NMR (300 MHz, Chloroform-d) δ 7.50 – 7.45 (m, 3H), 7.44 – 7.40 (m, 1H), 7.39 – 7.34 (d, *J* = 1.8 Hz, 3H), 7.34 – 7.25 (m, 5H), 7.24 – 7.17 (m, 1H), 7.02 – 6.99 (m, 1H), 6.86 (d, *J* = 8.6 Hz, 1H), 6.53 (d, *J* = 2.7 Hz, 1H), 6.29 (dd, *J* = 8.6, 2.8 Hz, 1H), 5.00 – 4.87 (m, 2H), 4.58 – 4.41 (m, 1H), 4.34 – 4.10 (m, 1H), 3.93 (s, 3H), 2.96 – 2.77 (m, 1H), 2.49 – 2.34 (m, 2H), 2.16 – 2.02 (m, 1H), 2.00 – 1.88 (m, 1H), 1.60 – 1.48 (m, 1H); ^{13}C NMR (75 MHz, $CDCl_3$) δ 155.2, 147.9, 147.8, 145.1, 140.9, 138.5, 137.0, 130.6, 128.5, 128.5, 128.1, 127.9, 127.2, 126.3, 120.7, 118.7 (q, *J* = 320.7 Hz), 111.8, 98.3, 98.1, 71.2, 58.5, 57.2, 55.6, 37.9, 29.8, 20.0; ^{19}F NMR (376 MHz, $CDCl_3$) δ –72.9, –73.0; HRMS (ESI) m/z 598.1865 (M+H+), calc. for $C_{32}H_{31}F_3NO_5S^+$ 598.1870.

The ee was determined by HPLC analysis: CHIRALPAK IG (4.6 mm i.d. x 250 mm); Hexane/2-propanol = 95/5; flow rate: 1.0 mL/min; 25 °C; 210 nm; retention time: 6.8 min (major) and 7.4min (minor).

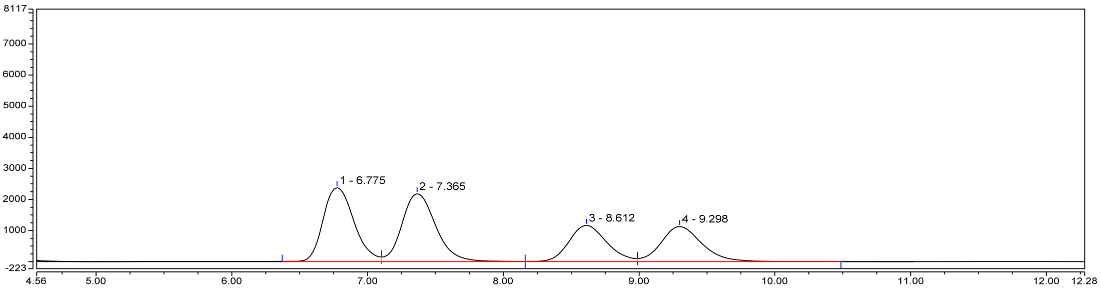

Entry	Retention Time	Area	Height	%Area
1	6.775	625.1848	2370.68	30.93
2	7.365	629.4244	2177.47	31.14
3	8.612	374.5081	1162.74	18.53
4	9.298	392.3369	1124.92	19.41

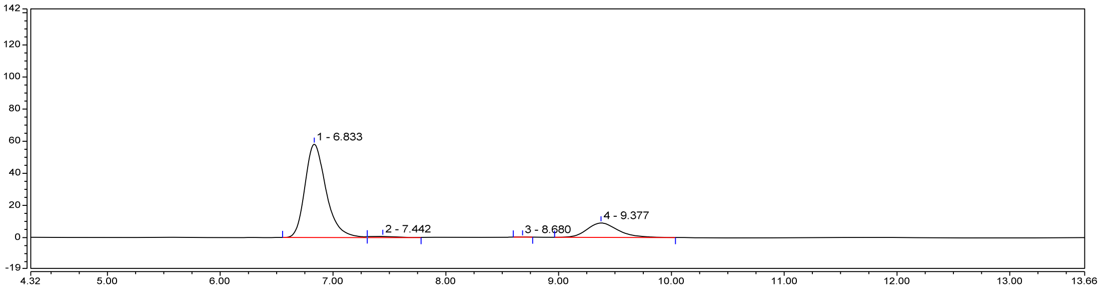

Entry	Retention Time	Area	Height	%Area
1	6.833	12.5999	58.15	80.60

2	7.442	0.1882	0.76	1.20
3	8.680	0.0070	0.07	0.04
4	9.377	2.8379	8.95	18.15

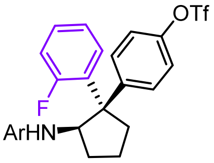

Ar = 2-OBn-5-OMePh

13d: white solid; Mp 66.8 - 68.9 °C; 53.5 mg, 87% yield; 93% ee; dr > 19:1; $[\alpha]_D^{22}$ –82.7 (*c* 1.0, $CHCl_3$); ^1H NMR (300 MHz, Chloroform-*d*) δ 7.43 (t, *J* = 7.7 Hz, 1H), 7.37 – 7.27 (m, 3H), 7.21 – 7.11 (m, 3H), 7.06 (d, *J* = 7.6 Hz, 3H), 6.92 – 6.76 (m, 3H), 6.70 (d, *J* = 8.1 Hz, 1H), 6.42 – 6.35 (m, 1H), 6.17 – 6.05 (m, 1H), 4.85 – 4.71 (m, 2H), 4.38 – 4.21 (m, 1H), 3.92 (d, *J* = 9.5 Hz, 1H), 3.76 (s, 3H), 3.04 – 2.89 (m, 1H), 2.35 –2.21 (m, 1H), 2.17 – 2.03 (m, 1H), 2.03 – 1.88 (m, 1H), 1.85 – 1.71 (m, 1H), 1.27 – 1.14 (m, 1H); ^{13}C NMR (75 MHz, $CDCl_3$) δ 161.0 (d, *J* = 248.3 Hz), 155.2, 148.0, 143.5, 141.0, 138.3, 136.9, 135.2 (d, *J* = 13.0 Hz), 129.7, 128.5, 128.2, 128.1, 127.4 (d, *J* = 4.6 Hz), 124.0 (d, *J* = 3.3 Hz), 120.7, 118.7 (q, *J* = 320.8 Hz), 116.2 (d, *J* = 22.4 Hz), 111.8, 98.3, 98.1, 71.3, 58.1, 55.6, 53.7, 36.2, 36.2, 28.7, 19.6; ^{19}F NMR (376 MHz, $CDCl_3$) δ –73.0, –109.2; HRMS (ESI) m/z 616.1771 ($M+H^+$), calc. for $C_{32}H_{30}F_4NO_5S^+$ 616.1775.

The ee was determined by HPLC analysis: CHIRALPAK IG (4.6 mm i.d. x 250 mm); Hexane/2-propanol = 90/10; flow rate: 1.0 mL/min; 25 °C; 210 nm; retention time: 7.0 min (minor) and 9.6 min (major).

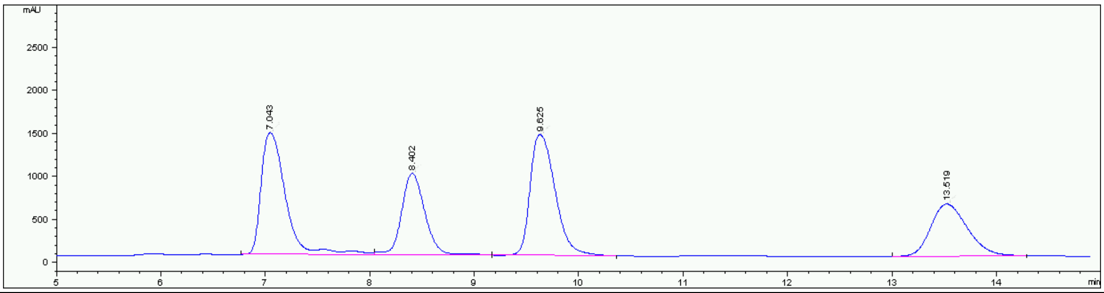

Entry	Retention Time	Area	Height	%Area
1	7.043	22946.1	1410	29.867
2	8.402	14953	948.8	19.463
3	9.625	23801.9	1411	30.980
4	13.519	15127.9	611.4	19.690

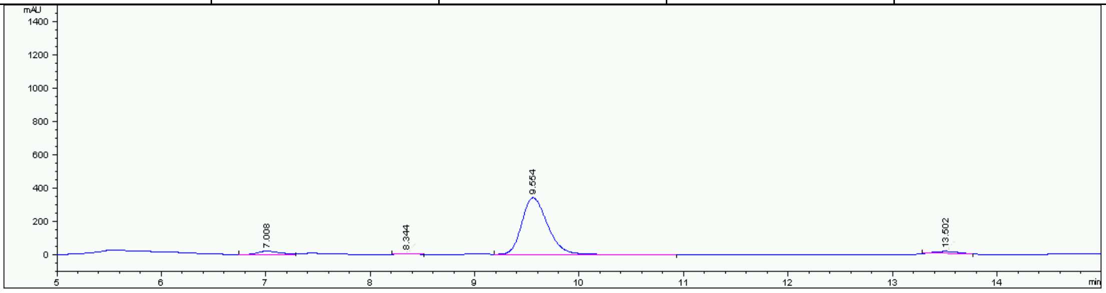

Entry	Retention Time	Area	Height	%Area
1	7.008	393.8	23.1	5.803
2	8.344	64.1	5.1	0.945
3	9.554	6080.7	343.9	89.607
4	13.502	247.4	14.5	3.646

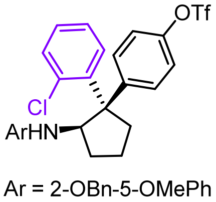

13e: yellow oil; 42.3 mg, 67% yield; 94% ee; dr > 19:1; $[\alpha]_D^{22}$ –30.0 (*c* 1.0, $CHCl_3$); ^1H NMR (300 MHz, Chloroform-*d*) δ 7.43 (d, *J* = 7.9 Hz, 1H), 7.29 (d, *J* = 6.8 Hz, 3H), 7.24 – 7.17 (m, 2H), 7.16 – 7.10 (m, 3H), 6.99 (d, *J* = 8.4 Hz, 2H), 6.78 (d, *J* = 8.4 Hz, 2H), 6.69 (d, *J* = 8.6 Hz, 1H), 6.39 (d, *J* = 2.7 Hz, 1H), 6.12 (dd, *J* = 8.6, 2.7 Hz, 1H), 4.84 – 4.67 (m, 2H), 4.28 (dd, *J* = 11.4, 6.3 Hz, 1H), 3.93 (s, 1H), 3.76 (s, 3H), 3.38 – 3.16 (m, 1H), 2.34 – 2.20 (m, 1H), 2.09 – 1.88 (m, 2H), 1.88 – 1.69 (m, 1H), 1.22 – 1.10 (m, 1H); ^{13}C NMR (75 MHz, $CDCl_3$) δ 155.1, 147.9, 145.1, 143.2, 140.9, 138.0, 136.7, 134.6, 131.4, 129.8, 128.4, 128.2, 128.1, 128.1, 127.9, 126.7, 120.6, 118.6 (q, *J* = 320.7 Hz), 111.6, 98.3, 98.1, 71.2, 58.2, 56.2, 55.5, 35.6, 28.3, 19.4; ^{19}F NMR (376 MHz, $CDCl_3$) δ –72.9; HRMS (ESI) m/z 632.1470 (M+H$^+$), calc. for $C_{32}H_{30}ClF_3NO_5S^+$ 632.1480.

The ee was determined by HPLC analysis: CHIRAL MQ (x 2) (4.6 mm i.d. x 250 mm); Hexane/2-propanol = 98/2; flow rate: 1.0 mL/min; 25 °C; 210 nm; retention time: 6.3 min (major) and 7.2min (minor).

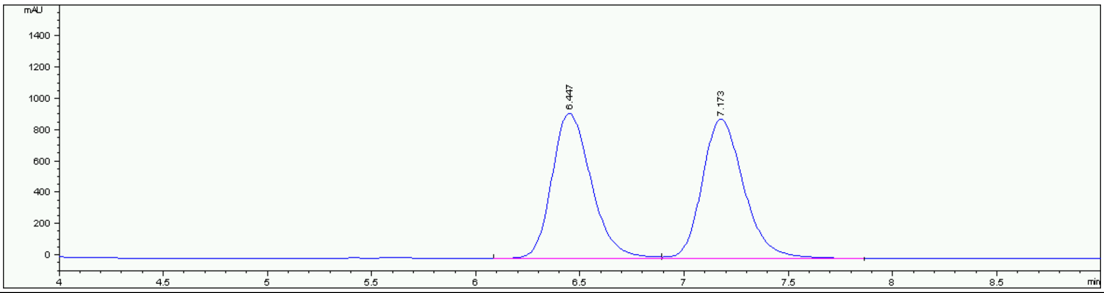

Entry	Retention Time	Area	Height	%Area
1	6.447	12288	930.1	49.696
2	7.173	12438.2	890.3	50.304

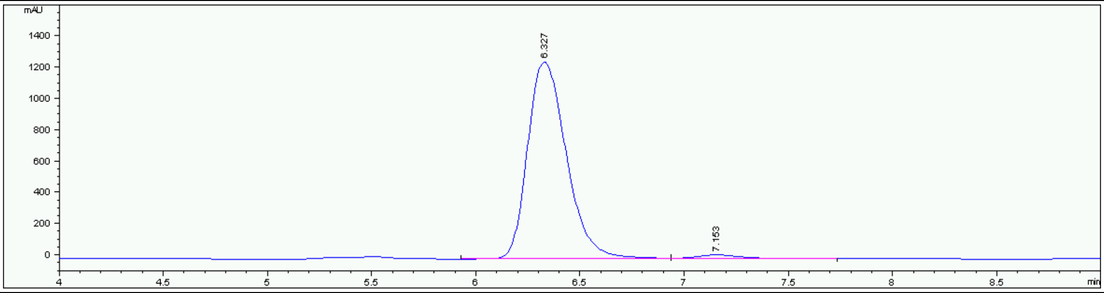

Entry	Retention Time	Area	Height	%Area

1	6.327	16451.6	1261	96.963
2	7.153	515.3	28	3.037

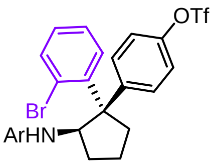

Ar = 2-OBn-5-OMePh

13f: white solid; Mp 75.4 - 77.5 °C; 47.9 mg, 71% yield; 94% ee; dr > 19:1; $[\alpha]_D^{22}$ −13.0 (*c* 1.0, $CHCl_3$); ^1H NMR (300 MHz, Chloroform-*d*) δ 7.43 (t, *J* = 6.7 Hz, 2H), 7.30 (d, *J* = 6.6 Hz, 3H), 7.22 (d, *J* = 7.9 Hz, 1H), 7.18 – 7.09 (m, 2H), 7.09 – 6.91 (m, 3H), 6.79 (d, *J* = 8.3 Hz, 2H), 6.70 (d, *J* = 8.5 Hz, 1H), 6.39 (d, *J* = 2.9 Hz, 1H), 6.12 (dd, *J* = 8.7, 2.9 Hz, 1H), 4.86 – 4.68 (m, 2H), 4.30 (dd, *J* = 11.6, 6.3 Hz, 1H), 3.93 (s, 1H), 3.77 (s, 3H), 3.50 – 3.30 (m, 1H), 2.37 – 2.21 (m, 1H), 2.11 – 1.91 (m, 2H), 1.87 – 1.71 (m, 1H), 1.26 – 1.14 (m, 1H); ^{13}C NMR (75 MHz, $CDCl_3$) δ 155.2, 148.0, 146.7, 143.2, 141.0, 138.1, 1368, 135.2, 130.2, 128.7, 128.5, 128.3, 128.2, 128.2, 127.4, 124.7, 120.6, 118.8 (q, *J* = 320.8 Hz), 111.7, 98.4, 98.2, 71.3, 58.4, 57.3, 556, 35.8, 28.4, 19.5; ^{19}F NMR (376 MHz, $CDCl_3$) δ −72.8; HRMS (ESI) m/z 676.0966 ($M+H^+$), calc. for $C_{32}H_{30}BrF_3NO_5S^+$ 676.0975.

The ee was determined by HPLC analysis: CHIRALCEL OD-H (4.6 mm i.d. x 250 mm); Hexane/2-propanol = 80/20; flow rate: 1.0 mL/min; 25 °C; 210 nm; retention time: 9.6 min (minor) and 15.5min (major).

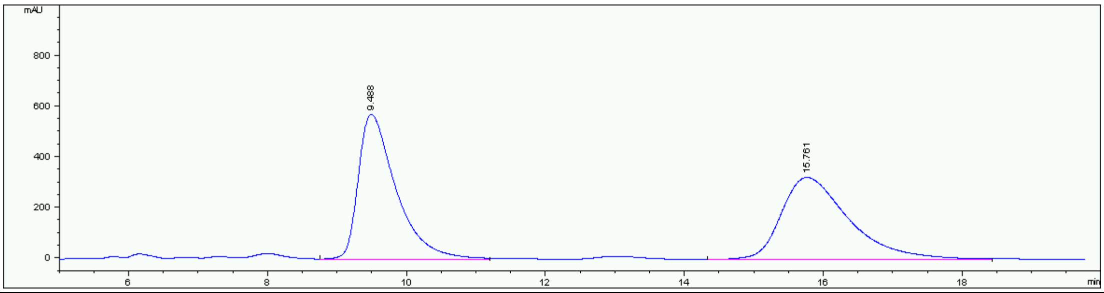

Entry	Retention Time	Area	Height	%Area
1	9.488	21906.7	573.5	49.724
2	15.761	22149.6	324.7	50.276

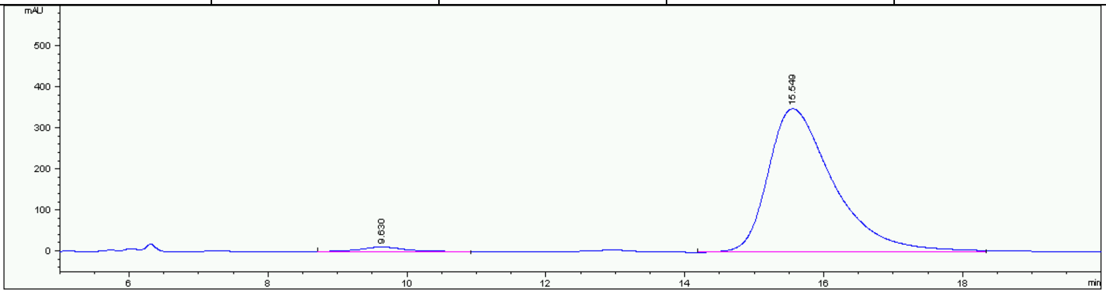

Entry	Retention Time	Area	Height	%Area
1	9.63	769.6	12.9	3.226
2	15.549	23084.4	350.5	96.774

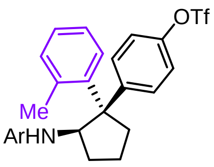

Ar = 2-OBn-5-OMePh

13g: white solid; Mp 104.4 - 105.8 °C; 45.8 mg, 75% yield; 94% ee; dr > 19:1; $[\alpha]_D^{22}$ –5.7 (*c* 1.0, $CHCl_3$); ^1H NMR (300 MHz, Chloroform-*d*) δ 7.42 (dd, *J* = 5.7, 3.7 Hz, 1H), 7.34 (d, *J* = 6.7 Hz, 3H), 7.18 (td, *J* = 5.8, 2.6 Hz, 4H), 7.12 – 6.96 (m, 3H), 6.81 (d, *J* = 8.4 Hz, 2H), 6.74 (d, *J* = 8.5 Hz, 1H), 6.46 (d, *J* = 2.8 Hz, 1H), 6.24 – 6.10 (m, 1H), 4.87 – 4.74 (m, 2H), 4.38 (dd, *J* = 11.3, 6.3 Hz, 1H), 4.06 (s, 1H), 3.82 (s, 3H), 3.00 (td, *J* = 9.4, 4.7 Hz, 1H), 2.34 (dt, *J* = 13.7, 7.3 Hz, 1H), 2.12 (td, *J* = 10.7, 9.1, 6.3 Hz, 1H), 2.06 – 1.83 (m, 2H), 1.77 (s, 3H), 1.31 – 1.24 (m, 1H); ^{13}C NMR (75 MHz, $CDCl_3$) δ 155.1, 147.9, 146.1, 144.3, 141.0, 138.0, 136.9, 136.8, 132.3, 129.9, 128.4, 128.1, 126.6, 126.3, 125.8, 120.7, 118.6 (q, *J* = 320.8 Hz, 1H), 111.6, 98.4, 98. 1, 71.2, 58.6, 56.2, 55.5, 36.2, 28.4, 21.7, 19.6; ^{19}F NMR (376 MHz, $CDCl_3$) δ –72.9; HRMS (ESI) m/z 612.2018 ($M+H^+$), calc. for $C_{33}H_{33}F_3NO_5S^+$ 612.2026.

The ee was determined by HPLC analysis: CHIRAL INB (4.6 mm i.d. x 250 mm); Hexane/2-propanol = 95/5; flow rate: 1.0 mL/min; 25 °C; 210 nm; retention time: 10.1 min (minor) and 20.8min (major).

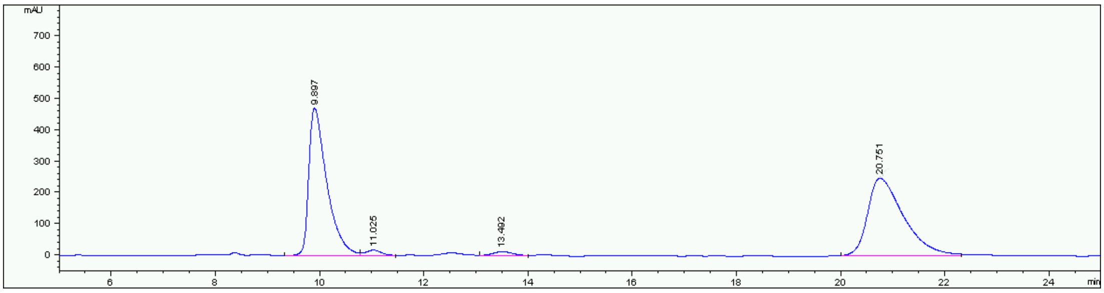

Entry	Retention Time	Area	Height	%Area
1	9.897	11564.1	472.8	47.340
2	11.025	458.3	19.5	1.876
3	13.492	454.6	14.7	1.861
4	20.751	11951	247.9	48.923

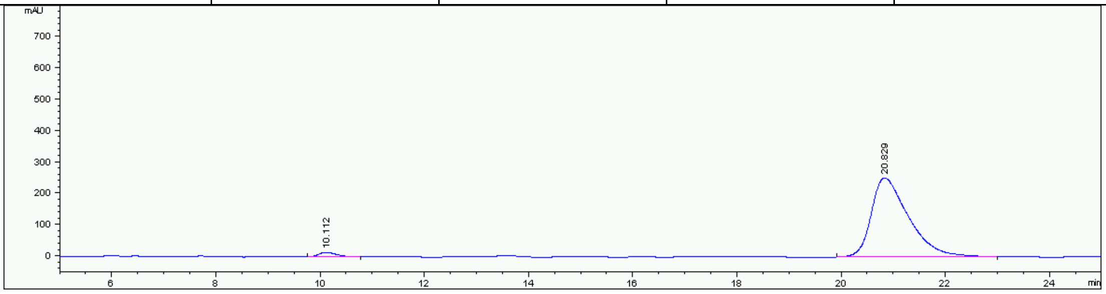

Entry	Retention Time	Area	Height	%Area
1	10.112	395.8	15.2	3.021
2	20.829	12704.1	252.5	96.979

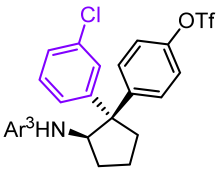

Ar = 2-OBn-5-OMePh

13h: yellow oil; 33.3 mg, 60% yield; 92% ee; dr = 5:1; $[\alpha]_D^{22}$ –3.5 (*c* 1.0, $CHCl_3$); ^{1}H NMR (300 MHz, Chloroform-*d*) δ 7.90 (d, *J* = 2.2 Hz, 1H), 7.37 – 7.27 (m, 3H), 7.25 – 7.15 (m, 4H), 6.81 (t, *J* = 8.3 Hz, 1H), 6.51 (d, *J* = 8.2 Hz, 2H), 4.77 (dd, *J* = 9.9, 6.2 Hz, 1H), 3.77 (s, 6H), 3.21 (s, 1H), 2.76 – 2.64 (m, 1H), 2.30 – 2.16 (m, 1H), 1.95 – 1.81 (m, 2H), 1.80 – 1.68 (m, 1H), 1.37 – 1.28 (m, 1H); ^{13}C NMR (75 MHz, $CDCl_3$) δ 151.4, 151.0, 148.1, 144.8, 134.1, 131.3, 129.5, 128.0, 126.6, 126.2, 126.1, 120.5, 120.4, 118.8 (q, *J* = 320.9 Hz), 104.6, 60.5, 57.1, 55.8, 38.7, 30.6, 19.4; ^{19}F NMR (376 MHz, CDCl3) δ -72.9, -72.9; ^{19}F NMR (376 MHz, $CDCl_3$) δ –72.88, -72.91; HRMS (ESI) m/z 556.1163 ($M+H^+$), calc. for $C_{26}H_{26}ClF_3NO_5S^+$ 556.1167.

The ee was determined by HPLC analysis: CHIRAL INB (x 2) (4.6 mm i.d. x 250 mm); Hexane/2-propanol = 98/2; flow rate: 1.0 mL/min; 25 °C; 210 nm; retention time: 8.9 min (major) and 13.4 min (minor).

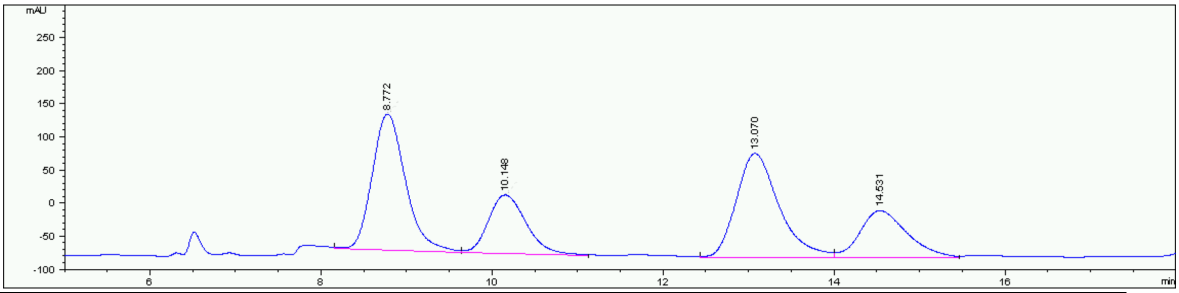

Entry	Retention Time	Area	Height	%Area
1	8.772	5649.8	206.6	33.895
2	10.148	2775.8	88.9	16.653
3	13.07	5501	157.7	33.002
4	14.531	2742	71.2	16.450

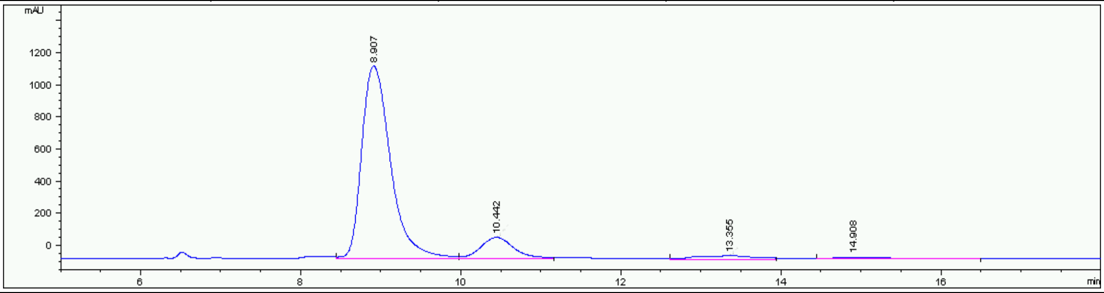

Entry	Retention Time	Area	Height	%Area
1	8.907	30151.6	1200.2	84.634
2	10.442	3831.4	132	10.755
3	13.355	1265	24.1	3.551
4	14.908	378	7.5	1.061

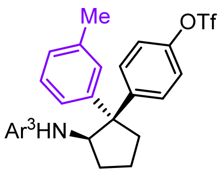

Ar = 2-OBn-5-OMePh

13i: yellow oil; 35.3 mg, 66% yield; 94% ee; dr = 6:1; $[\alpha]_D^{22}$ –14.8 (*c* 1.0, $CHCl_3$); ^{1}H NMR (300 MHz, Chloroform-*d*) δ 7.46 –7.40 (m, 1H), 7.38 – 7.27 (m, 3H), 7.23 – 7.13 (m, 3H), 7.02 (d, *J* = 7.4 Hz, 1H), 6.77 (t, *J* = 8.3 Hz, 1H), 6.49 (d, *J* = 8.3 Hz, 2H), 4.88 (dd, *J* = 9.0, 6.1 Hz, 1H), 3.75 (s, 6H), 2.77 – 2.63 (m, 1H), 2.38 – 2.24 (m, 4H), 1.97 – 1.82 (m, 2H), 1.80 – 1.65 (m, 1H), 1.44 – 1.34 (m, 1H); ^{13}C NMR (75 MHz, $CDCl_3$) δ 151.00, 148.6, 147.8, 145.8, 137.6, 131.2, 128.3, 128.1, 126.6, 124.8, 120.1, 119.9, 104.7, 60.8, 57.4, 55.8, 38.4, 30.8, 21.7, 19.6; ^{19}F NMR (376 MHz, $CDCl_3$) δ –72.92, –72.95; HRMS (ESI) m/z 536.1708 (M+H$^+$), calc. for $C_{27}H_{29}F_3NO_5S^+$ 536.1713.

The ee was determined by HPLC analysis: CHIRAL INB (x 2) (4.6 mm i.d. x 250 mm); Hexane/2-propanol = 98/2; flow rate: 1.0 mL/min; 25 °C; 210 nm; retention time: 12.4 min (major) and 14.6 min (minor).

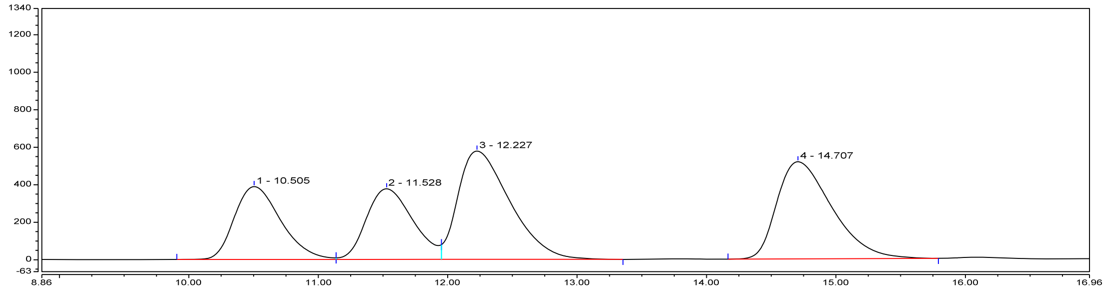

Entry	Retention Time	Area	Height	%Area
1	10.505	161.1304	161.1304	19.14
2	11.528	156.6051	156.6051	18.61
3	12.227	268.7861	268.7861	31.94
4	14.707	255.1242	255.1242	30.31

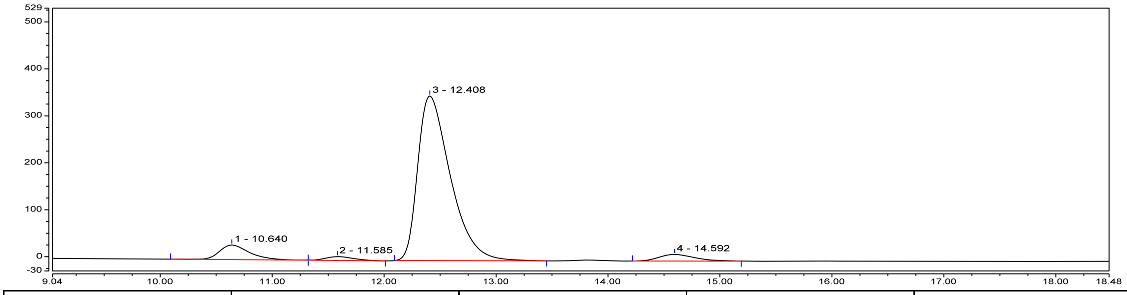

Entry	Retention Time	Area	Height	%Area
1	10.640	9.6628	31.01	7.13
2	11.585	2.3622	8.23	1.74
3	12.408	118.2839	350.33	87.33
4	14.592	5.1429	14.12	3.80

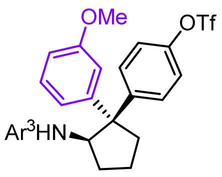

Ar = 2-OBn-5-OMePh

13j: yellow oil; 29.2 mg, 53% yield; 93% ee; dr = 6:1; $[\alpha]_D^{22}$ –9.4 (*c* 1.0, $CHCl_3$); ^{1}H NMR (300 MHz, Chloroform-*d*) δ 7.39 – 7.31 (m, 2H), 7.28 – 7.15 (m, 4H), 7.14 – 7.08 (m, 1H), 6.76 (dd, *J* = 8.0, 3.0 Hz, 2H), 6.53 – 6.47 (m, 2H), 4.94 – 4.75 (m, 1H), 3.78 – 3.72 (m, 9H), 3.34 (s, 1H), 2.76 – 2.63 (m, 1H), 2.42 – 2.26 (m, 1H), 1.98 – 1.84 (m, 2H), 1.82 – 1.68 (m, 1H), 1.43 –1.31 (m, 1H); ^{13}C NMR (75 MHz, $CDCl_3$) δ 159.4, 150.9, 150.3, 147.8, 145.5, 131.1, 129.1, 126.4, 120.3, 120.1, 119.7, 118.7 (q, *J* = 320.8 Hz), 114.0, 110.7, 104.6, 60.7, 57.5, 55.8, 55.0, 38.2, 30.8, 19.5; ^{19}F NMR (376 MHz, $CDCl_3$) δ –72.91, –72.94; HRMS (ESI) m/z 552.1656 ($M+H^+$), calc. for $C_{27}H_{29}F_3NO_6S^+$ 552.1662.

The ee was determined by HPLC analysis: CHIRAL INB (x 2) (4.6 mm i.d. x 250 mm); Hexane/2-propanol = 95/5; flow rate: 1.0 mL/min; 25 °C; 210 nm; retention time: 14.0 min (minor) and 14.8 min (major).

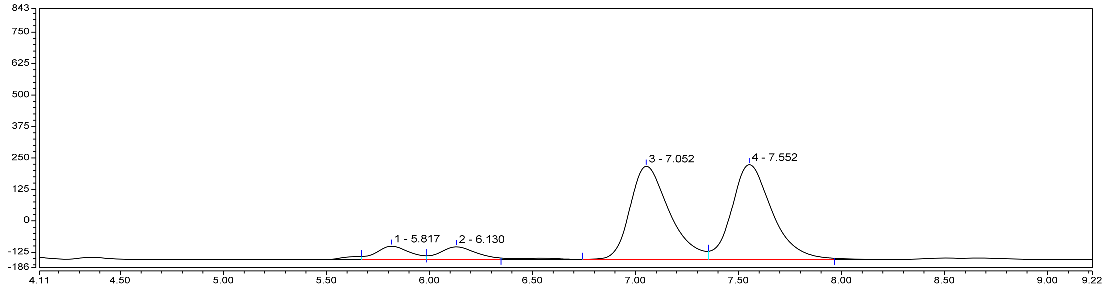

Entry	Retention Time	Area	Height	%Area
1	5.817	10.0445	53.41	5.35
2	6.130	10.3423	50.76	5.51
3	7.052	82.1490	370.68	43.74
4	7.552	85.2685	377.25	45.40

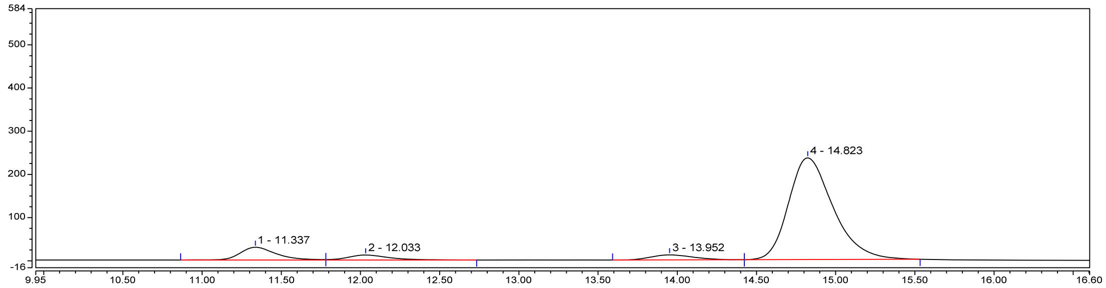

Entry	Retention Time	Area	Height	%Area
1	11.337	7.7570	29.57	8.47
2	12.033	3.4658	11.63	3.78
3	13.952	3.6223	11.74	3.96
4	14.823	76.7374	235.56	83.79

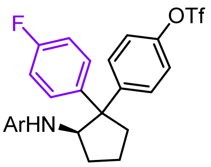

Ar = 2-OBn-5-OMePh

13k: white solid; Mp 58.6 - 59.8 °C; 50.4 mg, 82% yield; 91%/89% ee; dr = 1:1; $[\alpha]_D^{22}$ –93.5 (*c* 1.0, $CHCl_3$); ^{1}H NMR (300 MHz, Chloroform-*d*) δ 7.36 – 7.24 (m, 4H), 7.18 – 7.06 (m, 5H), 7.01 – 6.87 (m, 2H), 6.84 (d, *J* = 8.2 Hz, 1H), 6.77 –6.66 (m, 2H), 6.38 (dd, *J* = 7.2, 2.7 Hz, 1H), 6.23 – 6.08 (m, 1H), 4.86 – 4.71 (m, 2H), 4.36 – 4.23 (m, 1H), 4.07 (s, 1H), 3.76 (d, *J* = 1.4 Hz, 3H), 2.76 – 2.57 (m, 1H), 2.33 – 2.07 (m, 2H), 2.00 – 1.84 (m, 1H), 1.84 – 1.70 (m, 1H), 1.41 – 1.28 (m, 1H); ^{13}C NMR (75 MHz, $CDCl_3$) δ 161.4 (d, *J* = 246.5 Hz), 161.1 (d, *J* = 245.5 Hz), 155.1, 149.3, 147.9, 147.6, 144.6, 143.6, 143.5, 141.0 (d, *J* = 2.0 Hz), 138.9, 138.9, 138.2, 136.9, 136.8, 130.5, 130.4, 130.2, 129.1, 128.7, 128.6, 128.4, 128.4, 128.1, 128.0, 127.9, 127.6, 120.9, 120.7, 118.7 (q, *J* = 320.9 Hz), 118.6 (q, *J* = 320.7 Hz), 115.1 (d, *J* = 21.1 Hz), 115.0 (d, *J* = 21.0 Hz), 111.8, 98.5, 71.2, 71.1, 58.7, 58.5, 56.5, 56.2, 55.4, 38.2, 38.0, 29.6, 29.4, 19.8, 19.7; ^{19}F NMR (376 MHz, $CDCl_3$) δ –72.9, –73.0, –115.6, –116.4; HRMS (ESI) m/z 616.1763 (M+H$^+$), calc. for $C_{32}H_{30}F_4NO_6S^+$ 616.1775.

The ee was determined by HPLC analysis: CHIRALPAK MQ (x 2) (4.6 mm i.d. x 250 mm); Hexane/2-propanol = 99/1; flow rate: 1.0 mL/min; 25 °C; 210 nm; retention time: 8.9 min (major) and 11.5min (minor).

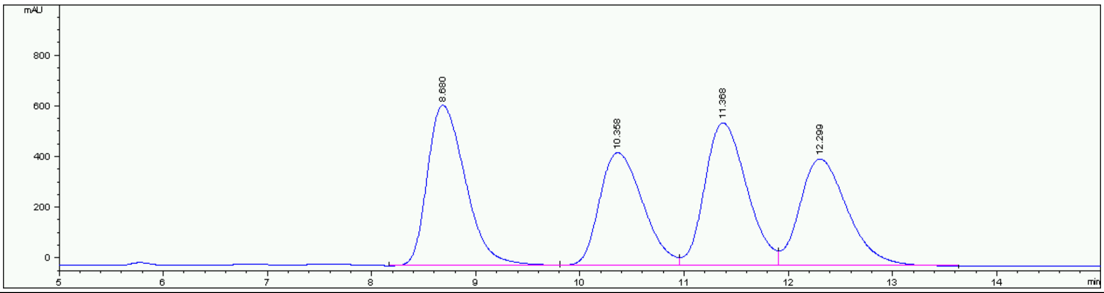

Entry	Retention Time	Area	Height	%Area
1	8.68	16134.4	633.3	27.591
2	10.358	12940.4	446.5	22.129
3	11.368	15885.1	564.5	27.164
4	12.299	13518.1	422.5	23.117

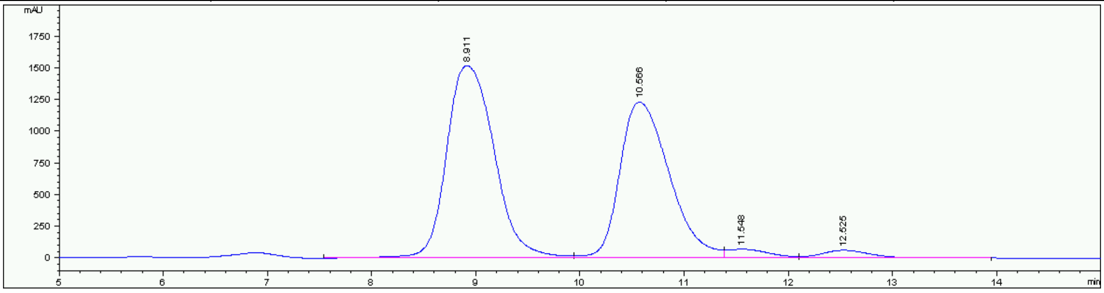

Entry	Retention Time	Area	Height	%Area
1	8.911	48620.7	1522.2	51.974
2	10.566	40817.7	1233.6	43.632
3	11.548	1988.9	70.8	2.126
4	12.525	2121.6	62.4	2.268

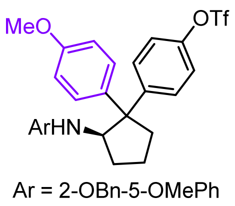

13l: white solid; Mp 57.8 - 58.7 °C; 54.5 mg, 87% yield; 92%/88% ee; dr = 1:1; $[\alpha]_D^{22}$ –49.5 (*c* 1.0, $CHCl_3$); ^{1}H NMR (300 MHz, Chloroform-*d*) δ 7.38 – 7.31 (m, 4H), 7.22 – 7.12 (m, 5H), 6.99 (d, *J* = 8.7 Hz, 1H), 6.90 (d, *J* = 8.7 Hz, 1H), 6.84 – 6.72 (m, 2H), 6.66 (d, *J* = 8.7 Hz, 1H), 6.43 (dd, *J* = 10.5, 2.8 Hz, 1H), 6.24 – 6.12 (m, 1H), 4.95 – 4.80 (m, 2H), 4.39 – 4.28 (m, 1H), 3.82 (d, *J* = 1.5 Hz, 3H), 3.79 (s, 1.4H), 3.71 (s, 1.6H), 2.82 – 2.64 (m, 1H), 2.36 – 2.13 (m, 2H), 2.06 – 1.91 (m, 1H), 1.89 – 1.74 (m, 1H), 1.51 – 1.37 (m, 1H); ^{13}C NMR (75 MHz, $CDCl_3$) δ 158.2, 157.8, 155.2, 155.1, 149.8, 147.8, 147.5, 145.2, 141.0, 140.9, 139.8, 138.4, 138.3, 137.0, 136.9, 135.2, 130.5, 129.8, 129.1, 128.4, 128.3, 128.0, 128.0, 127.8, 127.5, 120.8, 120.6, 113.7, 113.6, 112.2, 111.9, 98.6, 98.4, 71.2, 71.2, 59.0, 58.7, 56.6, 56.0, 55.5, 55.1, 55.1, 38.2, 37.8, 29.8, 29.4, 20.0, 19.8; ^{19}F NMR (376 MHz, $CDCl_3$) δ –72.9, –73.0; HRMS (ESI) m/z 628.1968 (M+H$^+$), calc. for $C_{33}H_{33}F_3NO_6S^+$ 628.1975.

The ee was determined by HPLC analysis: Lux i-Amylose-3 (4.6 mm i.d. x 250 mm) - Lux Amylose-1 (4.6 mm i.d. x 250 mm); Hexane/2-propanol = 85/15; flow rate: 1.0 mL/min; 25 °C; 210 nm; retention time: 11.2 min (major) and 11.7 min (minor).

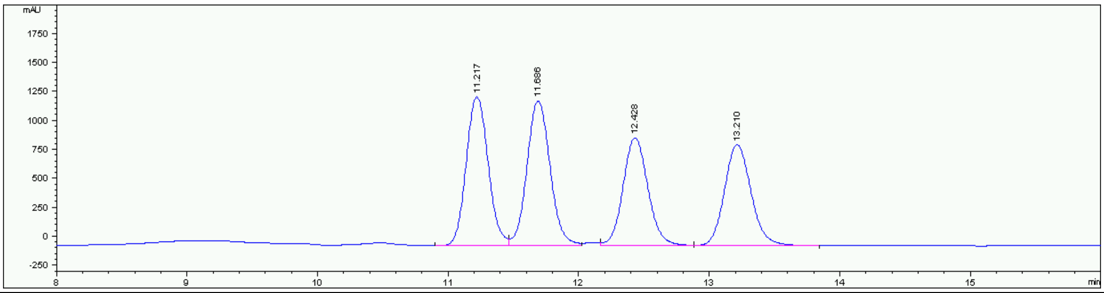

Entry	Retention Time	Area	Height	%Area
1	11.217	15108.9	1284.7	27.275
2	11.686	15490.7	1250.6	27.964
3	12.428	12407.8	932.5	22.399
4	13.21	12386.9	873.4	22.361

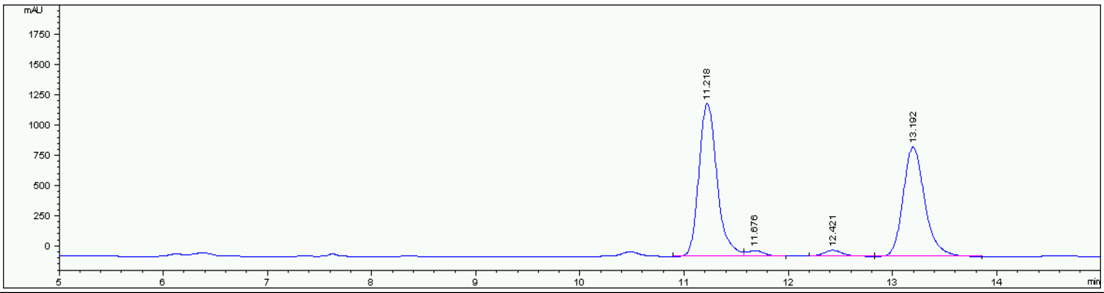

Entry	Retention Time	Area	Height	%Area
1	11.218	15455.2	1266.1	51.679
2	11.676	579.8	48.6	1.939
3	12.421	705.9	51.5	2.361
4	13.192	13165.1	907	44.022

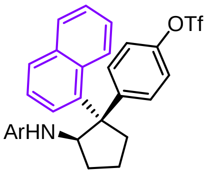

Ar = 2-OBn-5-OMePh

13m: white solid; Mp 72.6 - 73.8 °C; 38.8 mg, 60% yield; 92% ee; dr > 19:1; $[\alpha]_D^{22}$ –6.0 (*c* 1.0, $CHCl_3$); ^{1}H NMR (300 MHz, Chloroform-*d*) δ 7.64 (dd, *J* = 11.9, 8.1 Hz, 2H), 7.47 (d, *J* = 7.3 Hz, 1H), 7.30 (d, *J* = 7.7 Hz, 1H), 7.25 – 7.10 (m, 6H), 7.10 – 6.95 (m, 4H), 6.61 (d, *J* = 8.6 Hz, 1H), 6.35 (d, *J* = 2.8 Hz, 1H), 6.04 (dd, *J* = 8.6, 2.8 Hz, 1H), 4.76 – 4.61 (m, 2H), 4.49 – 4.33 (m, 1H), 3.98 – 3.84 (m, 1H), 3.70 (s, 3H), 3.19 – 3.05 (m, 1H), 2.34 – 2.21 (m, 1H), 2.13 – 1.92 (m, 2H), 1.83 – 1.70 (m, 1H), 1.34 – 1.21 (m, 1H); ^{13}C NMR (75 MHz, $CDCl_3$) δ 155.2, 148.0, 144.9, 143.4, 140.9, 138.4, 136.8, 135.0, 131.5, 128.9, 128.4, 128.1, 128.1, 127.9, 126.8, 125.3, 125.0, 124.9, 124.0, 120.9, 111.7, 98.2, 98.0, 71.2, 58.8, 56.31, 55.5, 38.3, 28.7, 20.0; ^{19}F NMR (376 MHz, $CDCl_3$) δ –73.0; HRMS (ESI) m/z 648.2023 ($M+H^+$), calc. for $C_{36}H_{33}F_3NO_5S^+$ 648.2026.

The ee was determined by HPLC analysis: CHIRAL MQ (x 2) (4.6 mm i.d. x 250 mm); Hexane/2-propanol = 98/2; flow rate: 1.0 mL/min; 25 °C; 210 nm; retention time: 7.7 min (major) and 8.8 min (minor).

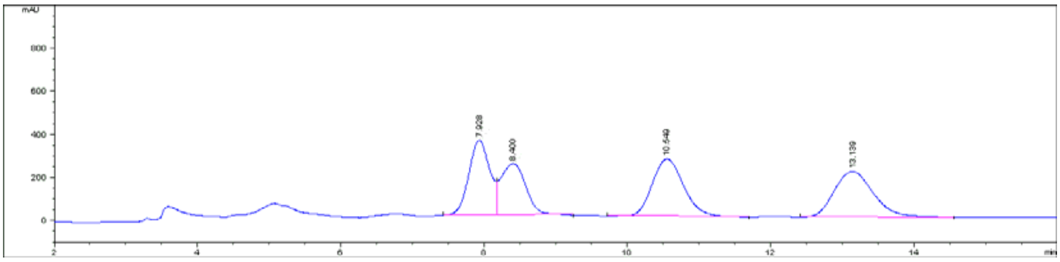

Entry	Retention Time	Area	Height	%Area

1	7.928	7150.5	347	24.650
2	8.4	5624.1	237.2	19.388
3	10.549	8114.5	266.3	27.973
4	13.139	8119.4	211.8	27.990

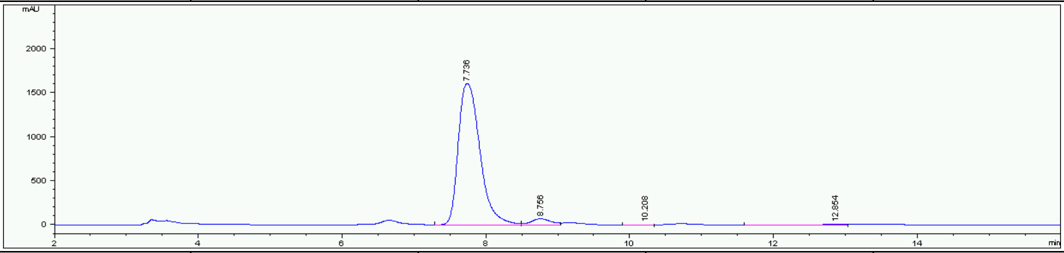

Entry	Retention Time	Area	Height	%Area
1	7.736	33973.5	1611	93.917
2	8.756	1533.1	74.8	4.238
3	10.208	147.9	7	0.409
4	12.854	519.6	9.7	1.436

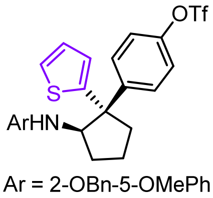

13n: yellow oil; 52.5 mg, 87% yield; 95%/65% ee; dr = 2:1; $[\alpha]_D^{22}$ –27.0 (*c* 1.0, $CHCl_3$); ^1H NMR (300 MHz, Chloroform-*d*) δ 7.49 (d, *J* = 8.5 Hz, 1H), 7.41 – 7.30 (m, 4H), 7.29 – 7.24 (m, 1H), 7.23 – 7.10 (m, 3H), 7.03 – 6.91 (m, 2H), 6.83 – 6.78 (m, 1H), 6.75 (d, *J* = 8.5 Hz, 0.5H), 6.62 (d, *J* = 3.6 Hz, 0.5H), 6.41 (dd, *J* = 9.7, 2.7 Hz, 1H), 6.26 – 6.15 (m, 1H), 5.00 – 4.81 (m, 2H), 4.65 – 4.20 (m, 2H), 3.82 (d, *J* = 1.8 Hz, 3H), 2.87 – 2.65 (m, 1H), 2.60 – 2.36 (m, 1H), 2.35 – 2.21 (m, 1H), 2.18 – 1.82 (m, 2H), 1.72 – 1.53 (m, 1H); ^{13}C NMR (75 MHz, $CDCl_3$) δ 155.2, 155.0, 151.8, 148.9, 148.0, 147.8, 147.8, 144.1, 140.8, 138.4, 138.2, 137.0, 137.0, 130.0, 129.0, 128.4, 128.3, 128.0, 127.7, 127.6, 127.5, 126.8, 126.7, 126.4, 124.6, 124.0, 123.8, 120.8, 120.8, 112.2, 112.1, 98.7, 98.6, 98.5, 98.4, 71.2, 71.1, 62.0, 59.2, 56.2, 55.5, 55.4, 54.6, 40.5, 38.0, 30.3, 29.4, 20.5, 19.4; ^{19}F NMR (376 MHz, $CDCl_3$) δ –72.9, –73.0; HRMS (ESI) m/z 604.1428 (M+H$^+$), calc. for $C_{30}H_{29}F_3NO_5S_2^+$ 604.1434.

The ee was determined by HPLC analysis: Lux Amylose-1 (4.6 mm i.d. x 250 mm) - CHIRAL MQ (x 2) (4.6 mm i.d. x 250 mm); Hexane/2-propanol = 95/5; flow rate: 1.0 mL/min; 25 °C; 210 nm; retention time: 16.7min (major) and 18.9min (minor).

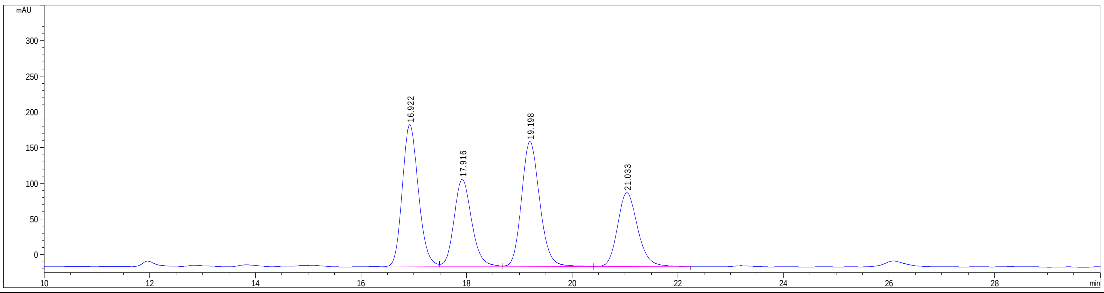

Entry	Retention Time	Area	Height	%Area
1	16.922	4240.1	199.1	30.243
2	17.916	2851.8	122.6	20.341
3	19.198	4192.6	175.3	29.904
4	21.033	2735.6	103.4	19.512

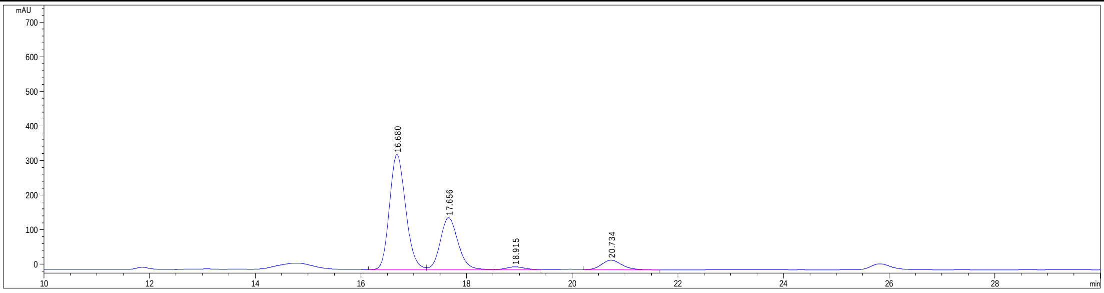

Entry	Retention Time	Area	Height	%Area
1	16.68	7063.5	332.1	61.741
2	17.656	3476.6	150	30.388
3	18.915	174.8	7.7	1.528
4	20.734	725.7	27.3	6.343

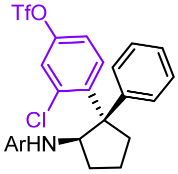

Ar = 2-OBn-5-OMePh

13o: white solid; Mp 56.7 - 57.8 °C; 40.4 mg, 64% yield; 89% ee; dr = 11:1; $[\alpha]_D^{22}$ –20.2 (*c* 1.0, $CHCl_3$); ^{1}H NMR (300 MHz, Chloroform-*d*) δ 7.61 (d, *J* = 8.8 Hz, 1H), 7.40 – 7.29 (m, 3H), 7.22 (d, *J* = 2.7 Hz, 1H), 7.20 – 7.11 (m, 4H), 7.11 – 7.03 (m, 2H), 6.97 (d, *J* = 7.5 Hz, 2H), 6.74 (d, *J* = 8.6 Hz, 1H), 6.42 (d, *J* = 2.8 Hz, 1H), 6.16 (dd, *J* = 8.6, 2.8 Hz, 1H), 4.93 – 4.75 (m, 2H), 4.34 – 4.21 (m, 1H), 4.09 (s, 1H), 3.81 (s, 3H), 3.45 – 3.30 (m, 1H), 2.30 (dt, *J* = 13.4, 7.1 Hz, 1H), 2.12 – 1.97 (m, 2H), 1.94 – 1.80 (m, 1H), 1.34 – 1.26 (m, 1H); ^{13}C NMR (75 MHz, $CDCl_3$) δ 155.2, 147.5, 147.0, 141.3, 140.9, 138.2, 136.9, 136.1, 129.8, 128.4, 128.2, 127.9, 127.8, 127.8, 126.7, 124.0, 119.2, 118.7 (q, *J* = 321.0 Hz), 112.0, 98.3, 98.2, 71.2, 58.6, 56.1, 55.5, 35.4, 28.1, 19.4; ^{19}F NMR (376 MHz, $CDCl_3$) δ –72.8, –72.9; HRMS (ESI) m/z 632.1473 (M+H$^+$), calc. for $C_{32}H_{30}ClF_3NO_5S^+$ 632.1480.

The ee was determined by HPLC analysis: CHIRALPAK IF (x 2) (4.6 mm i.d. x 250 mm); Hexane/2-propanol = 97/3; flow rate: 1.0 mL/min; 25 °C; 210 nm; retention time: 11.4 min

(major) and 12.2 min (minor).

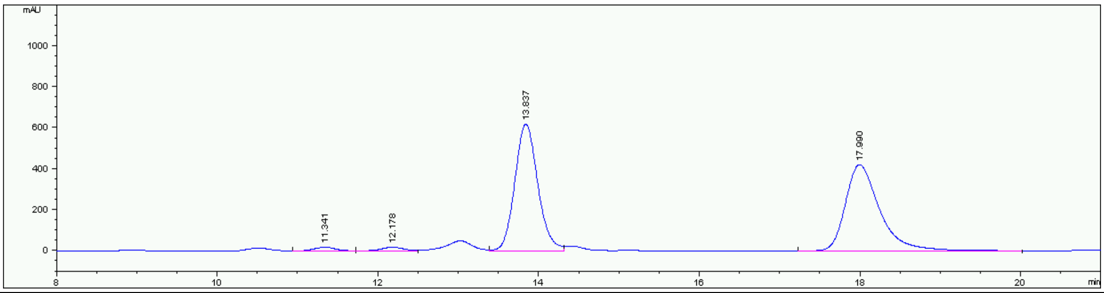

Entry	Retention Time	Area	Height	%Area
1	11.341	383.5	18.7	1.489
2	12.178	406.5	19.1	1.578
3	13.837	12374.7	619.6	48.036
4	17.99	12596.5	421.4	48.897

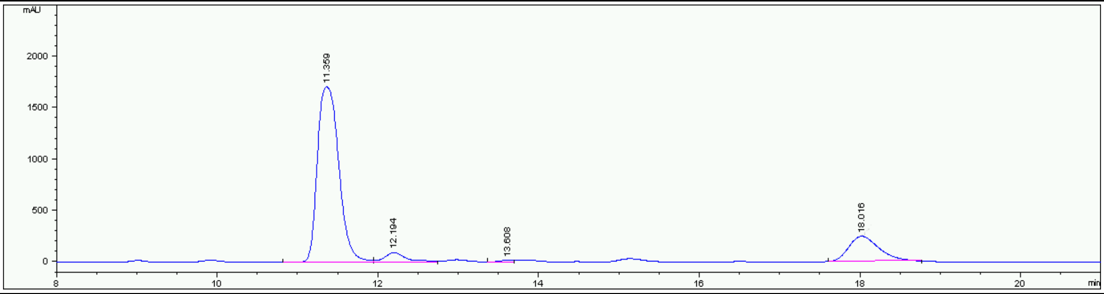

Entry	Retention Time	Area	Height	%Area
1	11.359	31415.6	1703.9	79.136
2	12.194	1760.2	92.8	4.434
3	13.608	221.5	16.9	0.558
4	18.016	6301	242.4	15.872

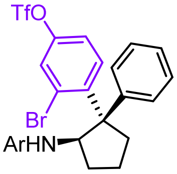

Ar = 2-OBn-5-OMePh

13p: white solid; Mp 83.1 - 84.9 °C; 45.2 mg, 67% yield; 92% ee; dr = 8:1; $[\alpha]_D^{22}$ –13.2 (*c* 1.0, $CHCl_3$); ^{1}H NMR (300 MHz, Chloroform-*d*) δ 7.58 (d, *J* = 8.9 Hz, 1H), 7.42 (d, *J* = 2.7 Hz, 1H), 7.37 – 7.28 (m, 3H), 7.21 (dd, *J* = 8.9, 2.6 Hz, 1H), 7.17 – 7.10 (m, 3H), 7.10 – 7.02 (m, 2H), 6.96 (d, *J* = 7.4 Hz, 2H), 6.73 (d, *J* = 8.6 Hz, 1H), 6.40 (d, *J* = 2.8 Hz, 1H), 6.15 (dd, *J* = 8.6, 2.8 Hz, 1H), 4.90 – 4.73 (m, 2H), 4.26 (s, 1H), 4.16 – 3.99 (m, 1H), 3.80 (s, 3H), 3.55 – 3.41 (m, 1H), 2.37 – 3.25 (m, 1H), 2.10 – 1.96 (m, 2H), 1.92 – 1.79 (m, 1H), 1.34 – 1.26 (m, 1H); ^{13}C NMR (75 MHz, $CDCl_3$) δ 155.2, 148.4, 147.3, 141.2, 140.9, 138.2, 136.8, 130.1, 128.4, 128.2, 128.2, 127.8, 127.8, 127.5, 126.6, 125.3, 119.7, 112.0, 98.3, 98.2, 71.2, 58.7, 57.0, 55.5, 35.5, 28.1, 19.4; ^{19}F NMR (376 MHz, $CDCl_3$) δ –72.8, –72.9; HRMS (ESI) m/z 676.0970 (M+H+), calc. for $C_{32}H_{30}BrF_3NO_5S^+$ 676.0975.

The ee was determined by HPLC analysis: CHIRAL INB (4.6 mm i.d. x 250 mm);

Hexane/2-propanol = 95/5; flow rate: 1.0 mL/min; 25 °C; 210 nm; retention time: 8.9 min (major) and 9.7 min (minor).

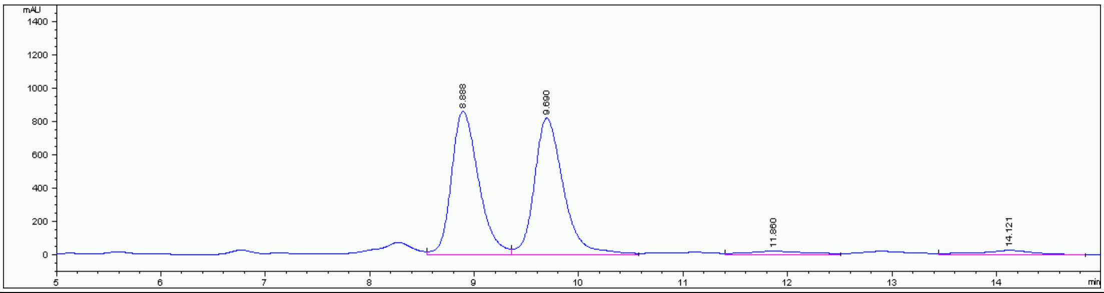

Entry	Retention Time	Area	Height	%Area
1	8.888	15609	862.4	46.175
2	9.69	15992.4	821.6	47.309
3	11.86	1020.2	23	3.018
4	14.121	1182.5	28.5	3.498

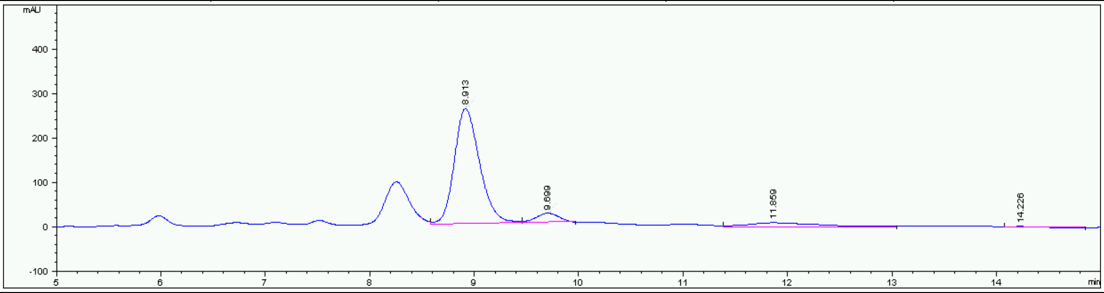

Entry	Retention Time	Area	Height	%Area
1	8.913	4413.5	260.1	81.629
2	9.699	332.7	21.3	6.154
3	11.859	611.3	10.9	11.305
4	14.226	49.3	2.5	0.912

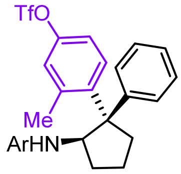

Ar = 2-OBn-5-OMePh

13q: white solid; Mp 135.4 - 136.4 °C; 36.7 mg, 60% yield; 92% ee; dr > 19:1; $[\alpha]_D^{22}$ –21.8 (*c* 1.0, $CHCl_3$); ^{1}H NMR (300 MHz, Chloroform-*d*) δ 7.51 (d, *J* = 8.8 Hz, 1H), 7.32 (dd, *J* = 5.1, 2.0 Hz, 3H), 7.19 – 7.12 (m, 3H), 7.09 – 6.94 (m, 6H), 6.74 (d, *J* = 8.6 Hz, 1H), 6.43 (d, *J* = 2.8 Hz, 1H), 6.15 (dd, *J* = 8.6, 2.8 Hz, 1H), 4.90 – 4.75 (m, 2H), 4.38 – 4.25 (m, 1H), 3.81 (s, 3H), 3.10 – 2.94 (m, 6.1 Hz, 1H), 2.38 –2.34 (m, 1H), 2.11 – 1.99 (m, 2H), 1.90 – 1.82 (m, 4H), 1.33 – 1.27 (m, 1H); ^{13}C NMR (75 MHz, $CDCl_3$) δ 155.1, 147.6, 147.6, 142.3, 140.8, 140.4, 138.3, 136.9, 128.3, 128.2, 127.9, 127.8, 127.8, 126.6, 124.2, 118.7 (q, *J* = 320.7 Hz), 117.8, 111.8, 98.1, 97.8, 71.1, 58.7, 55.8, 55.4, 35.9, 28.0, 21.8, 19.5; ^{19}F NMR (376 MHz, $CDCl_3$) δ –73.0; HRMS (ESI) m/z 612.2020 (M+H$^+$), calc. for $C_{33}H_{33}F_3NO_5S^+$ 612.2026.

The ee was determined by HPLC analysis: Lux i-Amylose-1 (4.6 mm i.d. x 250 mm);

Hexane/2-propanol = 99/1; flow rate: 1.0 mL/min; 25 °C; 210 nm; retention time: 9.1 min (major) and 10.5 min (minor).

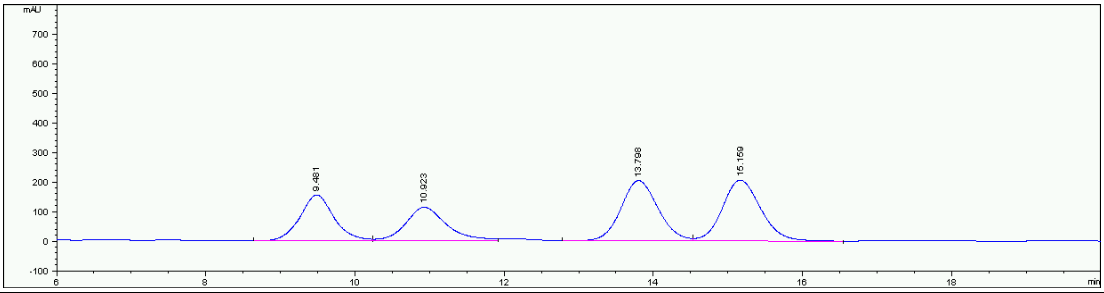

Entry	Retention Time	Area	Height	%Area
1	9.283	8161.4	248.1	21.527
2	10.569	7501.2	189	19.785
3	13.171	10965.2	306.5	28.922
4	14.874	11285.4	300	29.766

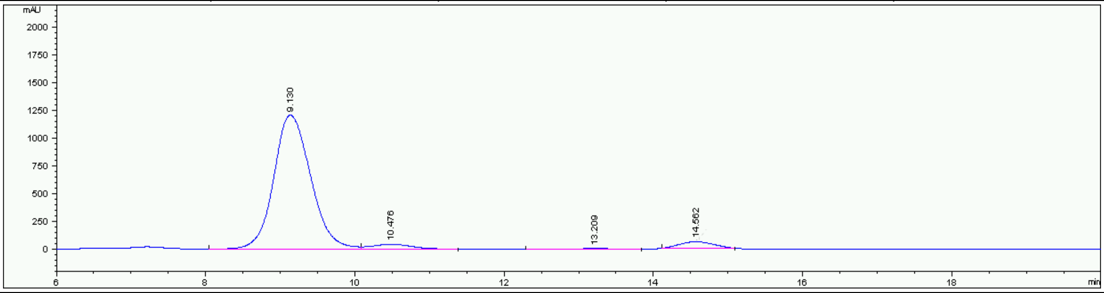

Entry	Retention Time	Area	Height	%Area
1	9.13	41728.3	1208.3	91.100
2	10.476	1741.4	45.3	3.802
3	13.209	263.1	6.5	0.574
4	14.562	2071.9	62.6	4.523

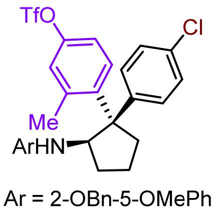

13r: white solid; Mp 79.1 - 80.2 °C; 54.8 mg, 85% yield; 93% ee; dr > 19:1; $[\alpha]_D^{22}$ –32.2 (*c* 1.0, $CHCl_3$); ^1H NMR (300 MHz, Chloroform-*d*) δ 7.49 (d, *J* = 8.8 Hz, 1H), 7.42 – 7.34 (m, 3H), 7.23 – 7.14 (m, 2H), 7.10 – 6.86 (m, 6H), 6.77 (d, *J* = 8.6 Hz, 1H), 6.45 (d, *J* = 2.8 Hz, 1H), 6.19 (dd, *J* = 8.6, 2.8 Hz, 1H), 4.94 – 4.77 (m, 2H), 4.30 (dd, *J* = 11.6, 6.3 Hz, 1H), 4.18 – 3.99 (m, 1H), 3.82 (s, 3H), 3.06 – 2.92 (m, 1H), 2.40 – 2.52 (m, 1H), 2.10 – 1.97 (m, 2H), 1.97 – 1.86 (m, 1H), 1.84 (s, 3H), 1.33 – 1.23 (m, 1H); ^{13}C NMR (75 MHz, $CDCl_3$) δ 155.2, 147.8, 147.0, 141.0, 140.2, 138.0, 136.7, 132.6, 129.3, 128.5, 128.4, 128.3, 128.1, 127.8, 124.3, 118.8 (q, *J* = 320.6 Hz), 118.0, 111.8, 98.4, 98.3, 71.2, 58.8, 55.6, 55.5, 35.9, 28.2, 21.8, 19.4; ^{19}F NMR (376 MHz, $CDCl_3$) δ –73.0; HRMS (ESI) m/z 646.1626 (M+H$^+$), calc. for $C_{33}H_{32}ClF_3NO_5S^+$ 646.1636.

The ee was determined by HPLC analysis: Lux Amylose-1 (4.6 mm i.d. x 250 mm) - CHIRAL MQ (x 2) (4.6 mm i.d. x 250 mm); Hexane/2-propanol = 95/5; flow rate: 1.0 mL/min; 25 °C; 210 nm; retention time: 10.4 min (major) and 11.1 min (minor).

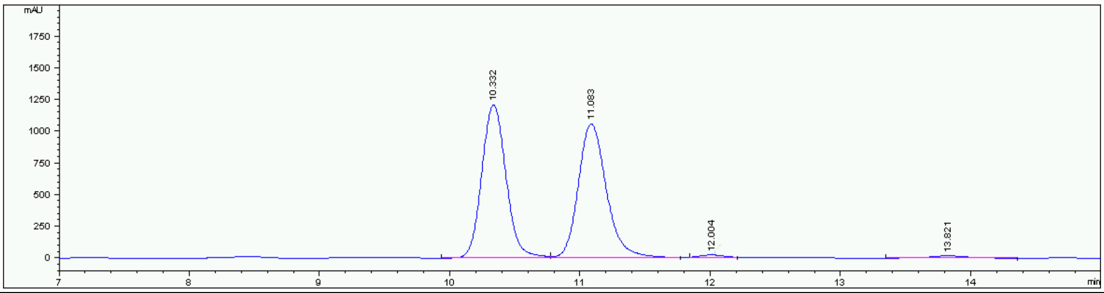

Entry	Retention Time	Area	Height	%Area
1	10.332	15735.1	1209.5	48.391
2	11.083	16039	1060.1	49.326
3	12.004	370.2	25.6	1.138
4	13.821	372.1	18.3	1.144

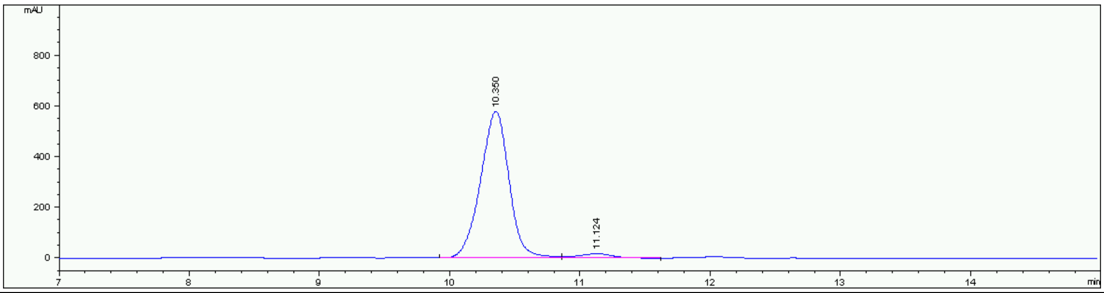

Entry	Retention Time	Area	Height	%Area
1	10.35	8873.2	580	96.295
2	11.124	341.4	18	3.705

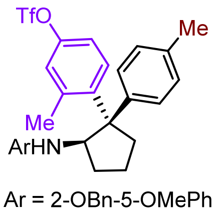

13s: white solid; Mp 71.7 - 72.8 °C; 42.5 mg, 68% yield; 92% ee; dr > 19:1; $[\alpha]_D^{22}$ –26.8 (*c* 1.0, $CHCl_3$); ^{1}H NMR (300 MHz, Chloroform-*d*) δ 7.50 (d, *J* = 8.8 Hz, 1H), 7.38 – 7.32 (m, 3H), 7.26 – 7.18 (m, 2H), 7.07 (dd, *J* = 8.7, 2.9 Hz, 1H), 6.97 (d, *J* = 2.9 Hz, 1H), 6.94 – 6.80 (m, 4H), 6.76 (d, *J* = 8.6 Hz, 1H), 6.47 (d, *J* = 2.8 Hz, 1H), 6.24 – 6.14 (m, 1H), 4.93 – 4.77 (m, 2H), 4.28 (dd, *J* = 11.4, 6.2 Hz, 1H), 3.83 (s, 3H), 3.10 – 2.98 (m, 1H), 2.34 – 2.28 (m, 1H), 2.25 (s, 3H), 2.09 – 1.97 (m, 2H), 1.90 – 1.80 (m, 4H), 1.41 –1.30 (m, 1H); ^{13}C NMR (75 MHz, $CDCl_3$) δ 155.2, 147.7, 147.6, 140.9, 140.5, 139.1, 138.1, 137.0, 136.1, 129.0, 128.3, 128.2, 127.9, 127.8, 127.8, 124.2, 118.7 (q, *J* = 320.6 Hz), 117.8, 112.1, 98.5, 98.3, 71.3, 59.0, 55.7, 55.5, 35.8, 28.1, 21.8, 20.9, 19.6; ^{19}F NMR (376 MHz, $CDCl_3$) δ –73.0; HRMS (ESI) m/z 626.2175 (M+H$^+$), calc. for $C_{34}H_{35}F_3NO_5S^+$ 626.2183.

The ee was determined by HPLC analysis: Lux i-Amylose-3 (4.6 mm i.d. x 250 mm) - CHIRAL MQ (x 2) (4.6 mm i.d. x 250 mm); Hexane/2-propanol = 98/2; flow rate: 1.0 mL/min; 25 °C; 210 nm; retention time: 15.3 min (major) and 17.0 min (minor).

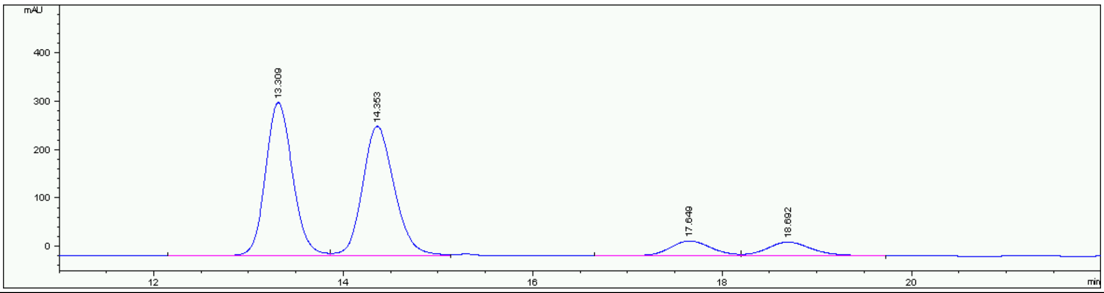

Entry	Retention Time	Area	Height	%Area
1	13.309	6335.7	317.5	43.507
2	14.353	6285.4	268.1	43.162
3	17.649	978.7	31.2	6.721
4	18.692	962.5	28.7	6.610

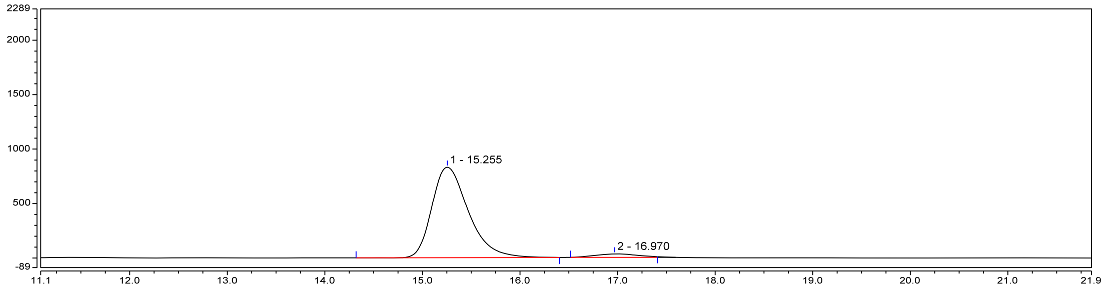

Entry	Retention Time	Area	Height	%Area
1	15.255	360.3622	831.70	95.82
2	16.970	15.7195	31.22	4.18

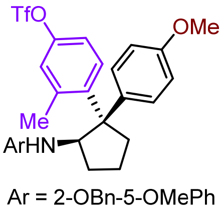

13t: yellow oil; 46.8 mg, 73% yield; 90% ee; dr > 19:1; $[\alpha]_D^{22}$ –16.3 (*c* 1.0, $CHCl_3$); ^1H NMR (300 MHz, Chloroform-*d*) δ 7.49 (d, *J* = 8.8 Hz, 1H), 7.35 – 7.29 (m, 3H), 7.21 – 7.12 (m, 2H), 7.04 (dd, *J* = 8.8, 2.8 Hz, 1H), 6.98 – 6.80 (m, 3H), 6.74 (d, *J* = 8.6 Hz, 1H), 6.59 (d, *J* = 8.4 Hz, 2H), 6.42 (d, *J* = 2.8 Hz, 1H), 6.15 (dd, *J* = 8.6, 2.8 Hz, 1H), 4.84 (q, *J* = 11.2 Hz, 2H), 4.33 – 4.20 (m, 1H), 4.19 – 4.08 (m, 1H), 3.81 (s, 3H), 3.68 (s, 3H), 3.04 – 2.89 (m, 1H), 2.35 – 2.24 (m, 1H), 2.06 – 1.93 (m, 2H), 1.90 – 1.75 (m, 4H), 1.33 – 1.24 (m, 1H); ^{13}C NMR (75 MHz, $CDCl_3$) δ 158.1, 155.3, 147.9, 147.6, 140.9, 140.5, 138.6, 137.0, 134.3, 129.0, 128.3, 128.2, 127.8, 127.6, 124.2, 118.7 (q, *J* = 320.7 Hz), 117.8, 113.7, 112.3, 98.2, 98.0, 71.3, 58.8, 55.5, 55.3, 55.1, 36.0, 28.2, 21.8, 19.5; ^{19}F NMR (565 MHz, $CDCl_3$) δ –73.0; HRMS (ESI) m/z 664.1945 (M+Na$^+$), calc. for $C_{34}H_{34}F_3NO_6SNa^+$ 664.1951.

The ee was determined by HPLC analysis: CHIRALPAK IC (4.6 mm i.d. x 250 mm) - Lux i-Cellulose-5 (4.6 mm i.d. x 250 mm); Hexane/2-propanol = 95/5; flow rate: 1.0 mL/min; 25 °C; 210 nm; retention time: 15.3 min (major) and 17min (minor).

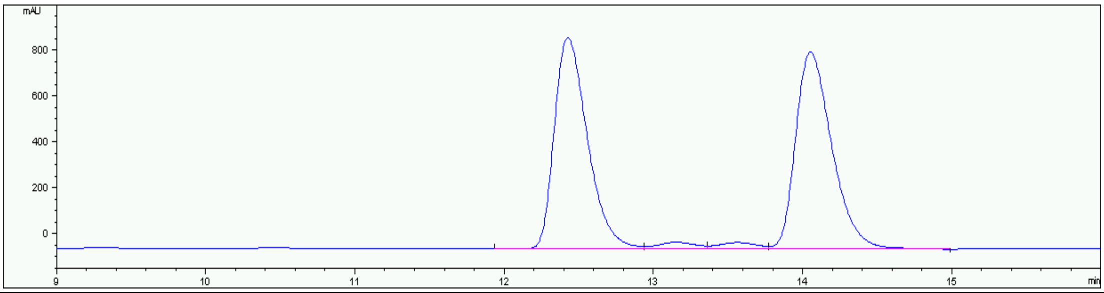

Entry	Retention Time	Area	Height	%Area
1	12.425	14209.6	920.9	47.749
2	13.149	503.8	29	1.693
3	13.56	494.2	28.2	1.661
4	14.051	14551.6	859.3	48.898

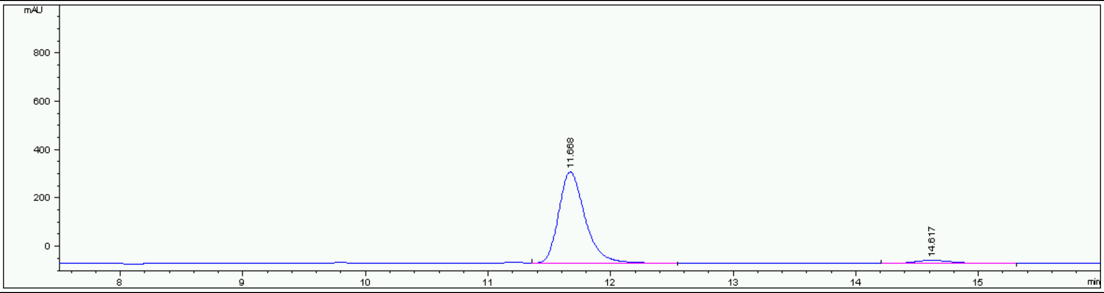

Entry	Retention Time	Area	Height	%Area
1	11.668	5740.3	378.9	95.015
2	14.617	301.2	13.2	4.985

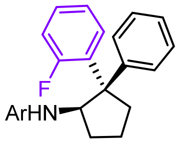

13u: white solid; Mp 80.6 - 81.8 °C; 15.4 mg, 61% yield; 91% ee; dr = 13:1; $[\alpha]_D^{22}$ –26.3 (*c* 1.0, $CHCl_3$); ^{1}H NMR (300 MHz, Methylene Chloride-*d_2*) δ 7.52 – 7.44 (m, 1H), 7.36 – 7.26 (m, 4H), 7.19 – 7.03 (m, 8H), 6.96 – 6.86 (m, 1H), 6.76 – 6.63 (m, 1H), 6.47 – 6.31 (m, 1H), 6.17 – 6.05 (m, 1H), 4.88 – 4.73 (m, 2H), 4.41 – 4.23 (m, 1H), 4.08 (d, *J* = 9.5 Hz, 1H), 3.76 (s, 3H), 3.12 – 2.92 (m, 1H), 2.34 – 2.22 (m, 1H), 2.17 – 1.93 (m, 2H), 1.88 – 1.73 (m, 1H), 1.31 – 1.23 (m, 1H); ^{13}C NMR (75 MHz, $CDCl_3$) δ 164.3, 161.0, 156.8, 144.6, 142.2, 140.1, 138.7, 137.8, 137.6, 129.9, 129.8, 129.7, 129.6, 129.4, 129.3, 129.2, 128.7, 128.0, 125.3, 125.3, 117.6, 117.3, 113.5, 99.6, 99.5, 72.5, 59.6, 56.9, 55.5, 55.4, 37.9, 37.8, 30.1, 21.2; ^{19}F NMR (565 MHz, $CDCl_3$) δ –109.1, –109.4; HRMS (ESI) m/z 468.2329 (M+H$^+$), calc. for $C_{34}H_{35}F_3NO_6S^+$ 4682333.

The ee was determined by HPLC analysis: Lux i-Amylose-3 (4.6 mm i.d. x 250 mm) -

CHIRALPAK IG (4.6 mm i.d. x 250 mm); Hexane/2-propanol = 97/3; flow rate: 1.0 mL/min; 25 °C; 210 nm; retention time: 19.2 min (minor) and 22.0 min (major).

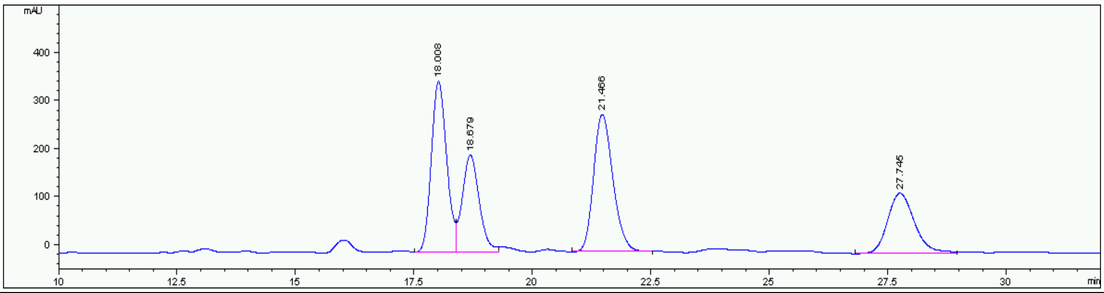

Entry	Retention Time	Area	Height	%Area
1	18.008	8040.7	356.8	30.643
2	18.679	5046.8	202.6	19.234
3	21.466	8127.4	286.2	30.974
4	27.745	5024.6	126.1	19.149

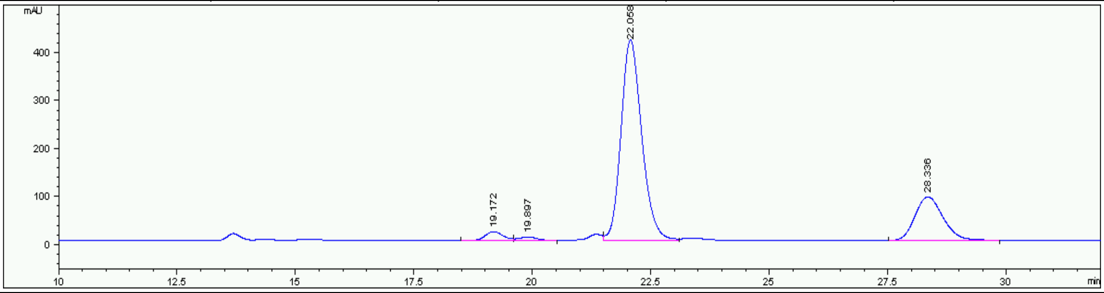

Entry	Retention Time	Area	Height	%Area
1	19.172	524.6	19.3	3.013
2	19.897	223.6	8	1.284
3	22.058	12862.7	419.4	73.877
4	28.336	3800.2	91.2	21.826

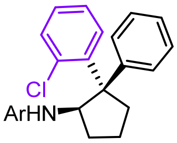

Ar = 2-OBn-5-OMePh

13v: white solid; Mp 164.7 - 166.1 °C; 25.6 mg, 53% yield; 72% ee; dr = 10:1; $[\alpha]_D^{22}$ –26.2 (*c* 1.0, $CHCl_3$); ^1H NMR (300 MHz, Methylene Chloride-d2) δ 7.51 (dd, J = 7.7, 1.7 Hz, 1H), 7.32 – 7.24 (m, 5H), 7.23 – 7.16 (m, 2H), 7.16 – 7.10 (m, 3H), 7.08 – 6.99 (m, 3H), 6.70 (d, J = 8.6 Hz, 1H), 6.41 (d, *J* = 2.8 Hz, 1H), 6.10 (dd, *J* = 8.6, 2.8 Hz, 1H), 4.88 – 4.68 (m, 2H), 4.40 – 4.26 (m, 1H), 4.07 (d, *J* = 9.9 Hz, 1H), 3.75 (s, 3H), 3.42 – 3.29 (m, 1H), 2.32 – 2.22 (m, 1H), 2.13 – 1.95 (m, 2H), 1.91 – 1.80 (m, 1H), 1.30 – 1.25 (m, 1H); ^{13}C NMR (75 MHz, CDCl3) δ 155.2, 146.1, 142.4, 140.8, 138.6, 137.0, 134.8, 131.2, 128.4, 128.3, 128.0, 127.7, 127.5, 126.5, 126.2, 112.0, 98.1, 97.7, 71.1, 58.4, 56.4, 55.5, 35.8, 28.2, 19.6; HRMS (ESI) m/z 506.1852 (M+Na$^+$), calc. for $C_{31}H_{30}ClNO_2Na^+$ 506.1857.

The ee was determined by HPLC analysis: CHIRAL MQ (x 2) (4.6 mm i.d. x 250 mm);

Hexane/2-propanol = 95/5; flow rate: 1.0 mL/min; 25 °C; 210 nm; retention time: 5.2 min (major) and 6.0 min (minor).

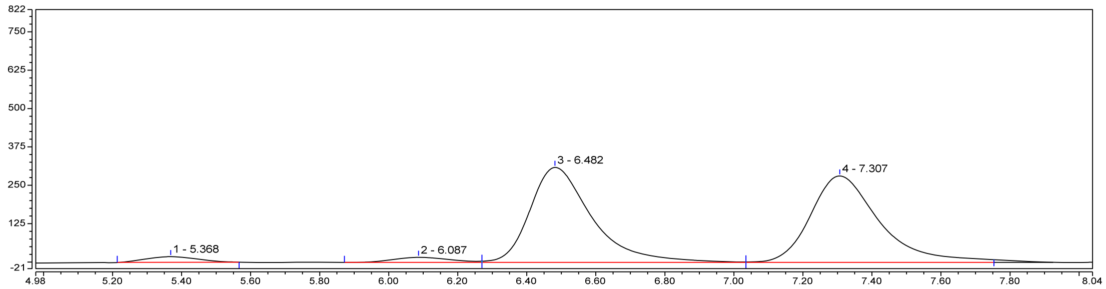

Entry	Retention Time	Area	Height	%Area
1	5.368	3.1554	18.38	2.33
2	6.087	3.0896	16.28	2.29
3	6.482	64.1915	309.21	47.49
4	7.307	64.7347	281.27	47.89

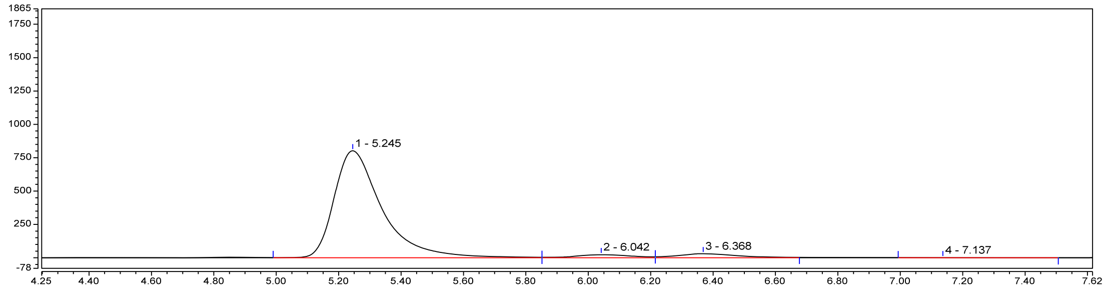

Entry	Retention Time	Area	Height	%Area
1	5.245	142.5618	801.89	92.12
2	6.042	4.7773	23.73	3.09
3	6.368	6.9584	30.83	4.50
4	7.137	0.4646	1.53	0.30

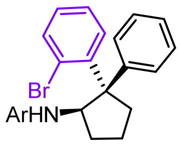

Ar = 2-OBn-5-OMePh

13w: white solid; Mp 123.6 - 124.8 °C; 28.4 mg, 54% yield; 89% ee; dr = 6:1; $[\alpha]_D^{22}$ –8.0 (*c* 1.0, $CHCl_3$); ^{1}H NMR (300 MHz, Chloroform-*d*) δ 7.66 – 7.43 (m, 2H), 7.39 – 7.24 (m, 5H), 7.19 – 7.10 (m, 4H), 7.08 – 6.99 (m, 3H), 6.72 (d, *J* = 8.6 Hz, 1H), 6.48 – 6.40 (m, 1H), 6.13 (dd, *J* = 8.6, 2.8 Hz, 1H), 4.90 – 4.75 (m, 2H), 4.42 – 4.29 (m, 1H), 4.17 (s, 1H), 3.81 (d, *J* = 1.8 Hz, 3H), 3.64 – 3.35 (m, 1H), 2.38 – 2.28 (m, 1H), 2.15 – 1.97 (m, 2H), 1.97 – 1.75 (m, 1H), 1.43 – 1.28 (m, 1H); ^{13}C NMR (75 MHz, $CDCl_3$) δ 155.2, 148.7, 147.7, 144.4, 142.4, 140.8, 140.8, 138.6, 137.2, 137.0, 135.0, 128.9, 128.8, 128.4, 128.4, 128.2, 128.1, 128.0, 127.8, 127.7, 127.6, 127.2, 127.2, 126.3, 125.9, 124.9, 112.0, 112.0, 98.2, 97.6, 71.1, 71.0, 58.6, 58.5, 57.5, 57.4, 55.5, 37.8, 36.0, 29.7, 28.3, 20.2, 19.7; HRMS (ESI) m/z 528.1526 (M+H$^+$), calc. for $C_{31}H_{31}BrNO_2^+$ 528.1533.

The ee was determined by HPLC analysis: CHIRAL INA (x 2) (4.6 mm i.d. x 250 mm); Hexane/2-propanol = 98/2; flow rate: 1.0 mL/min; 25 °C; 210 nm; retention time: 13.7 min (minor) and 15.1 min (major).

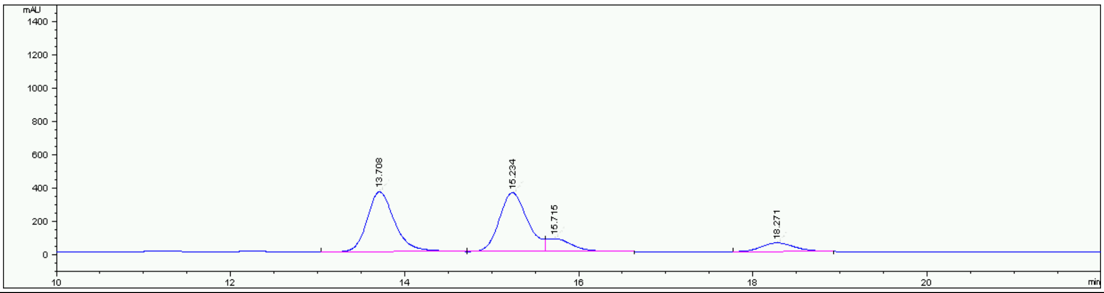

Entry	Retention Time	Area	Height	%Area
1	13.708	8053.4	361.3	42.147
2	15.234	8025.8	353.8	42.002
3	15.715	1541.1	77.9	8.065
4	18.271	1487.9	53.5	7.787

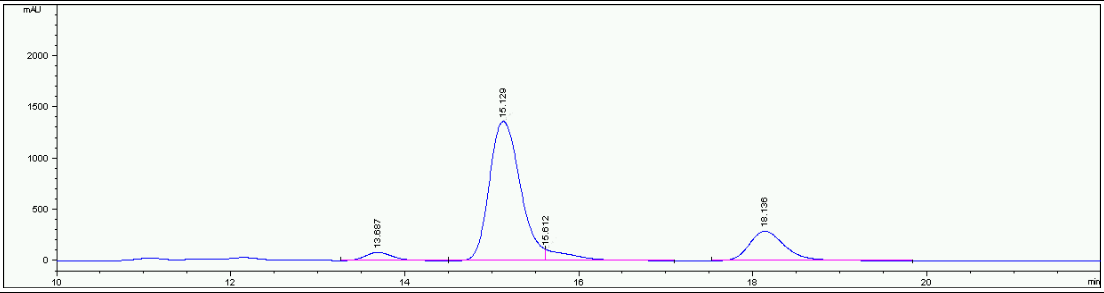

Entry	Retention Time	Area	Height	%Area
1	13.687	1932.7	82.6	4.186
2	15.129	33604.9	1359.3	72.782
3	15.612	2337.9	113.4	5.064
4	18.136	8296.2	288.4	17.968

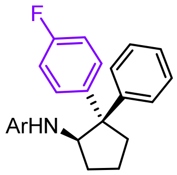

Ar = 2-OBn-5-OMePh

13x: yellow oil; 31.3 mg, 67% yield; 91% ee; dr = 3:1; $[\alpha]_D^{22}$ –8.7 (*c* 1.0, $CHCl_3$); ^{1}H NMR (300 MHz, Chloroform-*d*) δ 7.44 – 7.34 (m, 3H), 7.34 – 7.23 (m, 4H), 7.23 – 7.06 (m, 5H), 7.03 – 6.68 (m, 3H), 6.46 (t, *J* = 2.5 Hz, 1H), 6.19 (dd, *J* = 8.6, 3.1 Hz, 1H), 4.96 – 4.78 (m, 2H), 4.47 – 4.32 (m, 1H), 4.19 (s, 1H), 3.85 (d, *J* = 1.7 Hz, 3H), 2.83 – 2.66 (m, 1H), 2.43 – 2.19 (m, 2H), 2.09 – 1.94 (m, 1H), 1.89 – 1.74 (m, 1H), 1.58 – 1.44 (m, 1H); ^{13}C NMR (75 MHz, $CDCl_3$) δ 161.2 (d, *J* = 245.5 Hz), 155.1, 148.4, 140.9, 140.1 (d, *J* = 3.3 Hz), 138.7, 137.0, 130.3 (d, *J* = 7.8 Hz), 128.7, 128.4, 128.2, 127.5, 127.1, 125.9, 114.8 (d, *J* = 20.9 Hz), 111.8, 98.2, 97.9, 71.0, 58.5, 56.9, 55.5, 38.0, 29.8, 20.0; ^{19}F NMR (565 MHz, $CDCl_3$) δ –116.4, –117.2; HRMS (ESI) m/z 468.2325 (M+H$^+$), calc. for $C_{34}H_{35}F_3NO_6S^+$ 468.2333.

The ee was determined by HPLC analysis: Lux i-Amylose-3 (4.6 mm i.d. x 250 mm) - CHIRALPAK IG (4.6 mm i.d. x 250 mm); Hexane/2-propanol = 97/3; flow rate: 1.0 mL/min; 25 °C; 210 nm; retention time: 19.2 min (minor) and 22.0 min (major).

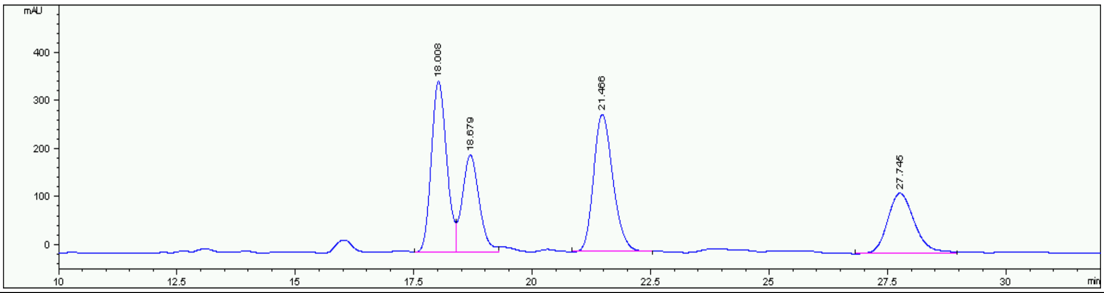

Entry	Retention Time	Area	Height	%Area
1	18.008	8040.7	356.8	30.643
2	18.679	5046.8	202.6	19.234
3	21.466	8127.4	286.2	30.974
4	27.745	5024.6	126.1	19.149

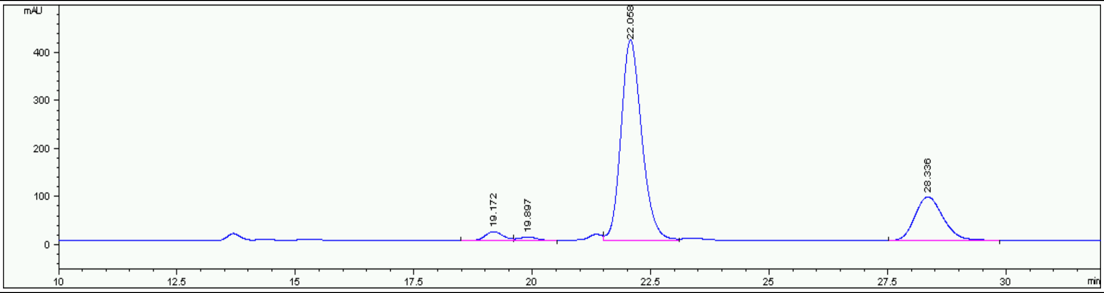

Entry	Retention Time	Area	Height	%Area
1	19.172	524.6	19.3	3.013
2	19.897	223.6	8	1.284
3	22.058	12862.7	419.4	73.877
4	28.336	3800.2	91.2	21.826

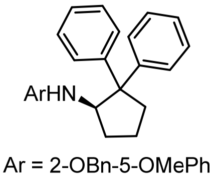

13y: white solid; Mp 154.6 - 156.2 °C; 16.2 mg, 36% yield; 88% ee; $[\alpha]_D^{22}$ -9.9 (*c* 1.0, $CHCl_3$); ^{1}H NMR (300 MHz, Chloroform-*d*) δ 7.36 – 7.27 (m, 5H), 7.19 – 7.02 (m, 7H), 6.95 (dd, *J* = 6.8, 3.0 Hz, 1H), 6.73 (d, *J* = 8.6 Hz, 1H), 6.41 (d, *J* = 2.8 Hz, 1H), 6.14 (dd, *J* = 8.6, 2.8 Hz, 1H), 4.95 – 4.74 (m, 2H), 4.27 (s, 1H), 4.15 – 4.02 (m, 1H), 3.82 (d, *J* = 4.9 Hz, 6H), 3.35 – 3.22 (m, 1H), 2.38 – 3.25 (m, 1H), 2.17 – 2.03 (m, 2H), 1.96 – 1.82 (m, 1H), 1.60 – 1.49 (m, 1H); ^{13}C NMR (75 MHz, $CDCl_3$) δ 155.2, 150.8, 143.4, 141.1, 140.8, 138.4, 138.3, 136.9, 128.4, 128.3, 128.1, 127.7, 127.7, 126.9, 120.3, 112.0, 111.3, 98.2, 97.8, 71.1, 59.1, 55.5, 55.5, 54.4, 36.2, 28.2, 19.7; HRMS (ESI) m/z 450.2426 (M+H$^+$), calc. for $C_{31}H_{32}NO_2^+$ 450.2428.

The ee was determined by HPLC analysis: CHIRALPAK IG (4.6 mm i.d. x 250 mm);

Hexane/2-propanol = 95/5; flow rate: 1.0 mL/min; 25 °C; 210 nm; retention time: 8.4 min (minor) and 10.8 min (major).

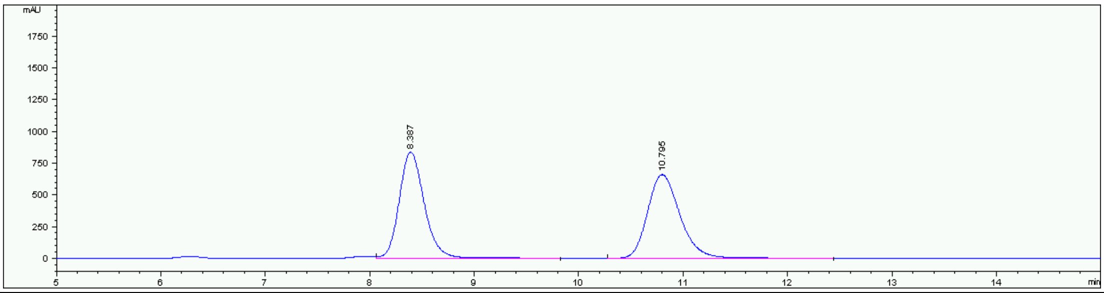

Entry	Retention Time	Area	Height	%Area
1	8.387	14428.3	840	49.598
2	10.795	14662.3	662.8	50.402

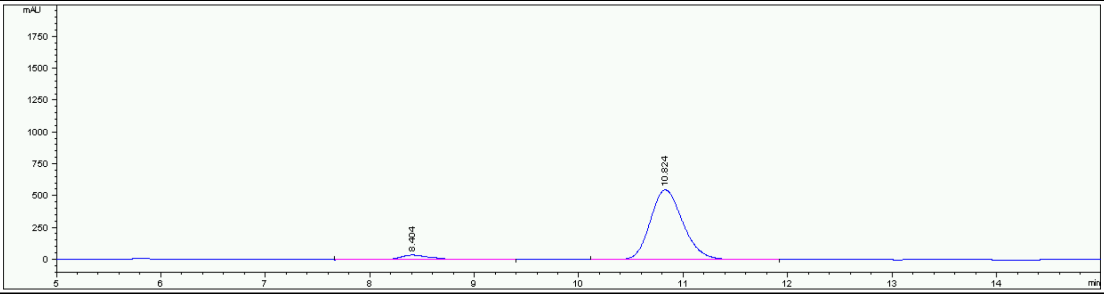

Entry	Retention Time	Area	Height	%Area
1	8.404	792.7	37.1	6.229
2	10.824	11933.1	549.1	93.771

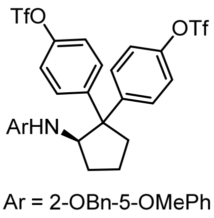

13z: white solid; Mp 53.5 - 54.7 °C; 59.6 mg, 80% yield; 95% ee; $[\alpha]_D^{22}$ –34.6 (*c* 1.0, $CHCl_3$); ^{1}H NMR (300 MHz, Chloroform-*d*) δ 7.39 – 7.29 (m, 4H), 7.28 (d, *J* = 2.3 Hz, 1H), 7.23 – 7.14 (m, 4H), 7.13 – 6.99 (m, 2H), 6.94 – 6.81 (m, 2H), 6.75 (d, *J* = 8.7 Hz, 1H), 6.38 (d, *J* = 2.8 Hz, 1H), 6.18 (dd, *J* = 8.6, 2.8 Hz, 1H), 4.93 – 4.75 (m, 2H), 4.31 (q, *J* = 9.0 Hz, 1H), 3.93 (d, *J* = 9.5 Hz, 1H), 3.80 (s, 3H), 2.82 – 2.63 (m 1H), 2.39 – 2.15 (m, 2H), 2.05 – 1.78 (m, 2H), 1.36 – 1.27 (m, 1H); ^{13}C NMR (75 MHz, $CDCl_3$) δ 155.1, 148.6, 148.0, 147.8, 143.7, 140.9, 138.0, 136.8, 130.5, 129.2, 128.5, 128.2 128.0, 121.2, 120.9, 118.7 (q, *J* = 320.8 Hz), 118.6 (q, *J* = 320.7 Hz), 111.7, 98.4, 98.3, 71.2, 58.3, 56.3, 55.5, 38.0, 29.4, 19.6; ^{19}F NMR (376 MHz, $CDCl_3$) δ –72.9, –73.0; HRMS (ESI) m/z 746.1298 (M+H$^+$), calc. for $C_{34}H_{30}F_6NO_8S^+$ 746.1312.

The ee was determined by HPLC analysis: CHIRALCEL OD-H (4.6 mm i.d. x 250 mm); Hexane/2-propanol = 90/10; flow rate: 1.0 mL/min; 25 °C; 210 nm; retention time: 6.6 min (minor) and 8.5 min (major).

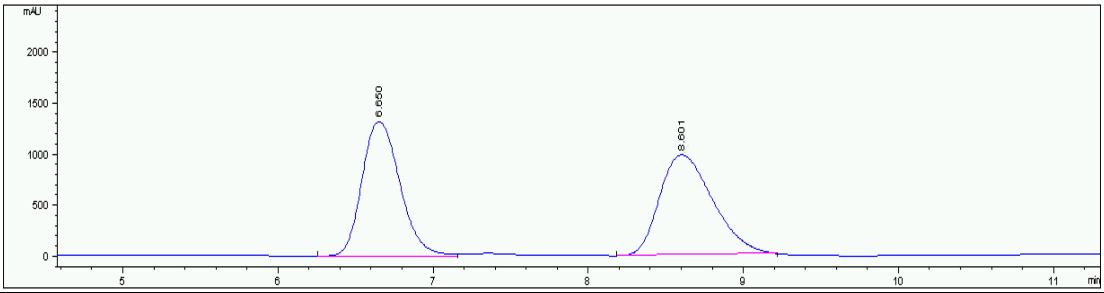

Entry	Retention Time	Area	Height	%Area
1	6.65	22759.2	1322.6	49.275
2	8.601	23429	982.9	50.725

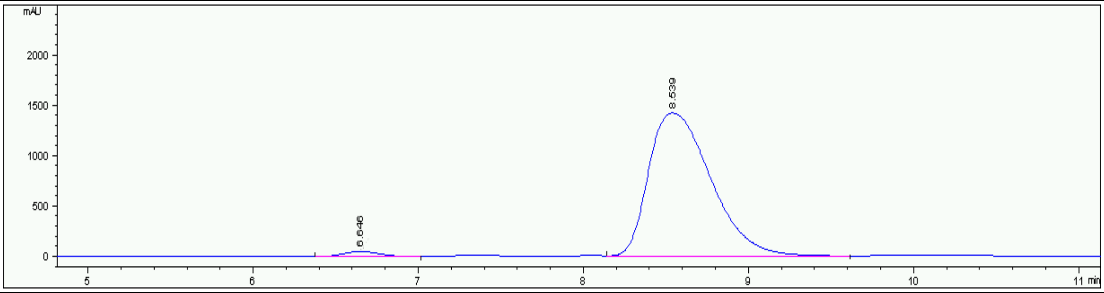

Entry	Retention Time	Area	Height	%Area
1	6.646	927.4	55	2.379
2	8.539	38063	1429.9	97.621

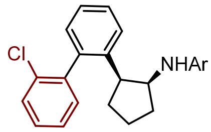

Ar = 2-OCH$_2$-(2,6-Me$_2$-Ph)-5-OMePh

15: yellow oil; 43.4 mg, 85% yield; 80% ee; dr = 6:1; $[\alpha]_D^{22}$ +15.6 (*c* 1.0, $CHCl_3$); ^{1}H NMR (300 MHz, $CDCl_3$) δ 7.41 (d, *J* = 7.4 Hz, 1H), 7.26 – 7.14 (m, 4H), 7.13 – 7.02 (m, 3H), 7.00 – 6.78 (m, 3H), 6.70 (d, *J* = 8.7 Hz, 1H), 6.18 – 5.92 (m, 2H), 4.83 (q, *J* = 10.1 Hz, 2H), 3.72 (d, *J* = 4.1 Hz, 3H), 3.37 – 3.24 (m, 1H), 3.16 – 3.00 (m, 1H), 2.23 (d, *J* = 5.8 Hz, 6H), 2.07 – 1.89 (m, 3H), 1.77 – 1.57 (m, 2H); ^{13}C NMR (75 MHz, $CDCl_3$) δ 154.7, 141.0, 140.4, 139.8, 138.4, 138.2, 133.3, 133.0, 131.7, 129.4, 128.9, 128.5, 128.2, 128.1, 128.1, 127.8, 127.0, 126.5, 126.2, 110.6, 97.8, 97.2, 65.2, 55.6, 55.4, 45.7, 33.3, 30.4, 23.2, 19.5; HRMS (ESI) m/z 512.2343 (M+H$^+$), calc. for $C_{33}H_{35}ClNO_2^+$ 512.2351.

The ee was determined by HPLC analysis: Lux Cellulose-1 (4.6 mm i.d. x 250 mm); Hexane/2-propanol = 98/2; flow rate: 1.0 mL/min; 25 ^{o}C; 210 nm; retention time: 6.6 min (minor) and 8.5 min (major).

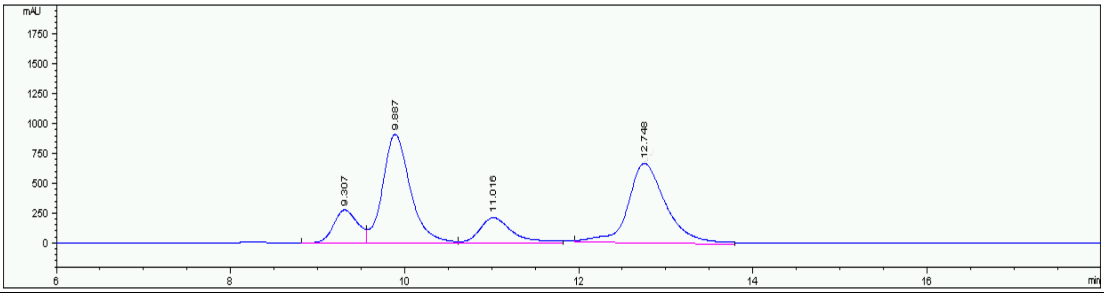

Entry	Retention Time	Area	Height	%Area
1	9.307	5770	284.8	10.793
2	9.887	20597.4	919.7	38.528
3	11.016	6197	220.3	11.592
4	12.748	20896.7	673.6	39.088

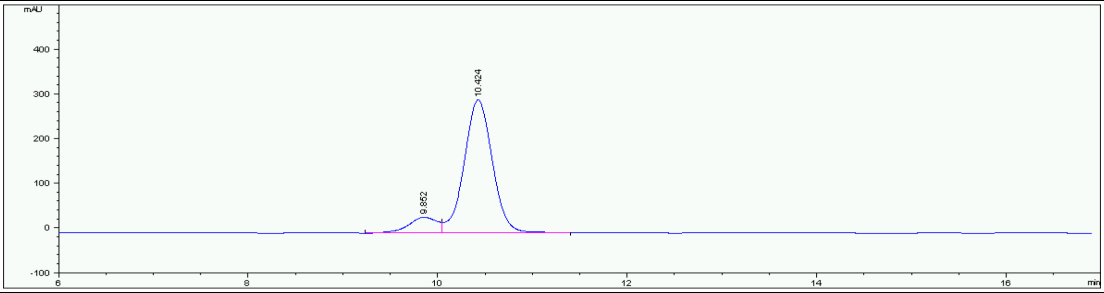

Entry	Retention Time	Area	Height	%Area
1	9.852	771.5	35.7	11.014
2	10.424	6233.2	298.7	88.986

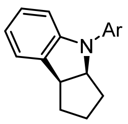

Ar = 2-OCH$_2$-(2,6-Me$_2$-Ph)-5-OMePh

16: yellow oil; 34.3 mg, 86% yield; 84% ee; $[\alpha]_D^{22}$ +21.4 (*c* 1.0, $CHCl_3$); ^{1}H NMR (300 MHz, $CDCl_3$) δ 7.28 – 7.06 (m, 7H), 6.87 – 6.61 (m, 3H), 5.17 – 4.90 (m, 3H), 3.87 (s, 3H), 3.85 – 3.74 (m, 1H), 2.43 (s, 6H), 3.16 – 1.78 (m, 3H), 1.74 – 1.50 (m, 3H); ^{13}C NMR (75 MHz, $CDCl_3$) δ 154.4, 149.4, 148.4, 138.0, 134.1, 133.6, 133.1, 128.2, 128.0, 127.0, 124.4, 117.5, 116.9, 111.6, 109.6, 107.3, 68.1, 66.6, 55.5, 45.7, 35.4, 32.9, 24.0, 19.4; HRMS (ESI) m/z 400.2263 (M+H$^+$), calc. for $C_{27}H_{30}NO_2^+$ 400.2271.

The ee was determined by HPLC analysis: Lux Cellulose-1 (4.6 mm i.d. x 250 mm); Hexane/2-propanol = 98/2; flow rate: 1.0 mL/min; 25 °C; 210 nm; retention time: 6.6 min (major) and 8.5 min (minor).

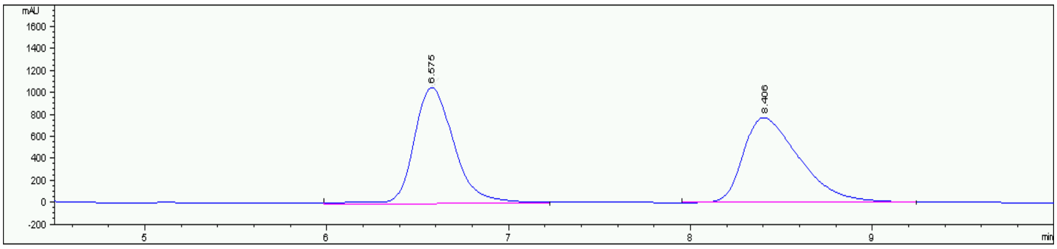

Entry	Retention Time	Area	Height	%Area

1	6.575	16909.9	1069.8	50.090
2	8.406	16849.3	774.1	49.910

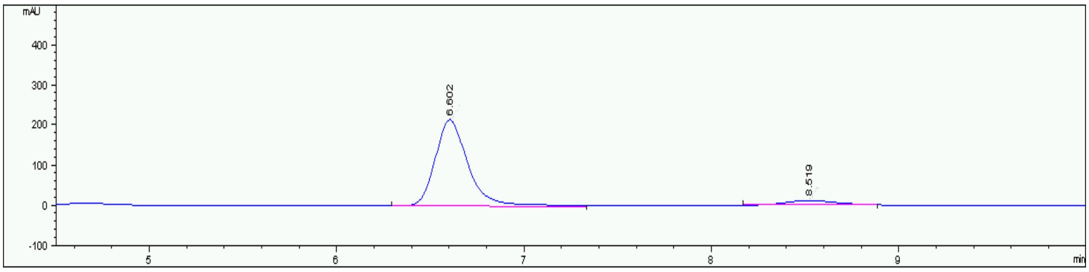

Entry	Retention Time	Area	Height	%Area
1	6.602	2743.6	216.4	91.898
2	8.519	241.9	11.8	8.102

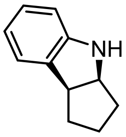

17: yellow oil; 40.7 mg, 64% yield; 84% ee; dr > 19:1; $[\alpha]_D^{22}$ +16.1 (*c* 1.0, $CHCl_3$); ^1H NMR (300 MHz, $CDCl_3$) δ 7.10 – 6.95 (m, 2H), 6.69 (t, *J* = 7.3 Hz, 1H), 6.55 (d, *J* = 7.8 Hz, 1H), 4.41 – 4.33 (m, 1H), 3.84 – 3.74 (m, 1H), 3.39 (s, 1H), 2.02 – 1.90 (m, 1H), 1.81 – 1.52 (m, 5H); ^{13}C NMR (75 MHz, $CDCl_3$) δ 151.1, 133.4, 127.3, 124.5, 118.4, 108.6, 63.3, 47.2, 36.8, 34.8, 24.4; HRMS (ESI) m/z 160.1119 ($M+H^+$), calc. for $C_{11}H_{14}N^+$ 160.1121.

The ee was determined by HPLC analysis: Lux Amylose-1 (4.6 mm i.d. x 250 mm); Hexane/2-propanol = 95/5; flow rate: 1.0 mL/min; 25 °C; 210 nm; retention time: 6.3 min (major) and 7.2 min (minor).

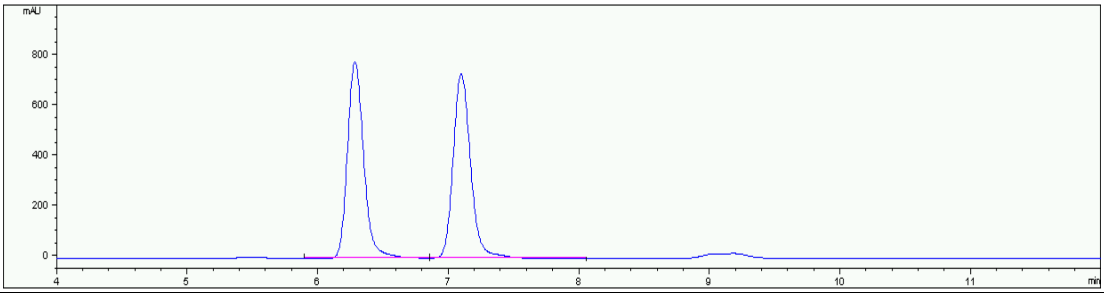

Entry	Retention Time	Area	Height	%Area
1	6.283	6809.8	784.1	49.705
2	7.097	6890.7	735.1	50.295

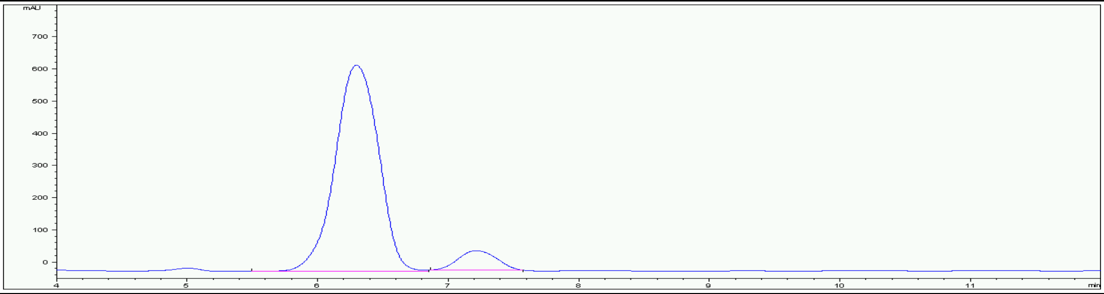

Entry	Retention Time	Area	Height	%Area

1	6.294	14932.2	641.9	92.025
2	7.213	1294.1	62.1	7.975

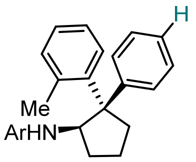

Ar = 2-OBn-5-OMePh

18: yellow oil; 36.3 mg, 78% yield; 94% ee; dr > 19:1; $[\alpha]_D^{22}$ −13.7 (*c* 1.0, $CHCl_3$); ^1H NMR (300 MHz, $CDCl_3$) δ 7.55 – 7.46 (m, 1H), 7.40 – 7.26 (m, 4H), 7.23 – 7.16 (m, 4H), 7.13 – 7.00 (m, 5H), 6.74 (d, *J* = 8.6 Hz, 1H), 6.48 (d, *J* = 2.8 Hz, 1H), 6.15 (dd, *J* = 8.6, 2.8 Hz, 1H), 4.84 (q, *J* = 11.2 Hz, 2H), 4.47 – 4.34 (m, 1H), 4.20 (s, 1H), 3.84 (s, 3H), 3.16 – 3.01 (m, 1H), 2.41 – 2.28 (m, 1H), 2.17 – 1.99 (m, 2H), 1.91 – 1.80 (m, 4H), 1.44 – 1.33 (m, 1H); ^{13}C NMR (75 MHz, $CDCl_3$) δ 155.1, 147.1, 143.4, 140.7, 138.7, 137.2, 137.0, 132.0, 128.3, 128.0, 127.7, 127.6, 126.4, 126.2, 126.2, 125.6, 111.8, 98.0, 97.4, 71.0, 58.5, 56.3, 55.4, 36.3, 28.2, 21.7, 19.7; HRMS (ESI) m/z 464.2578 (M+H$^+$), calc. for $C_{32}H_{34}NO_2^+$ 464.2584.

The ee was determined by HPLC analysis: Lux i-Amylose-3 (4.6 mm i.d. x 250 mm); Hexane/2-propanol = 98/2; flow rate: 1.0 mL/min; 25 °C; 210 nm; retention time: 9.6 min (minor) and 11.1 min (major).

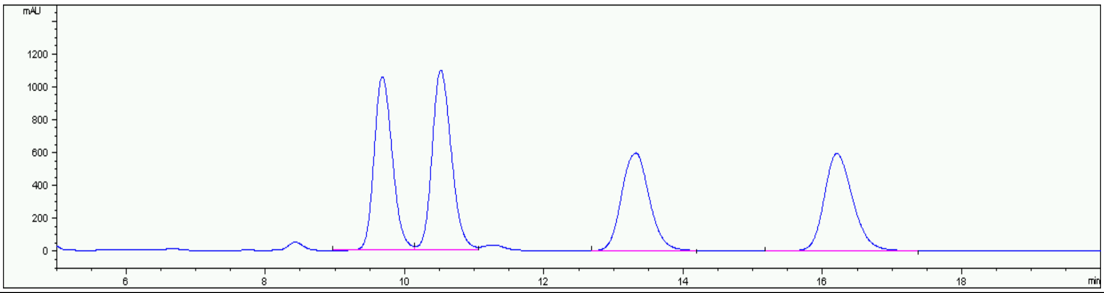

Entry	Retention Time	Area	Height	%Area
1	9.674	19401.8	1056.9	26.116
2	10.513	21339.5	1097.8	28.724
3	13.316	16570.6	595	22.305
4	16.201	16979.8	593.1	22.856

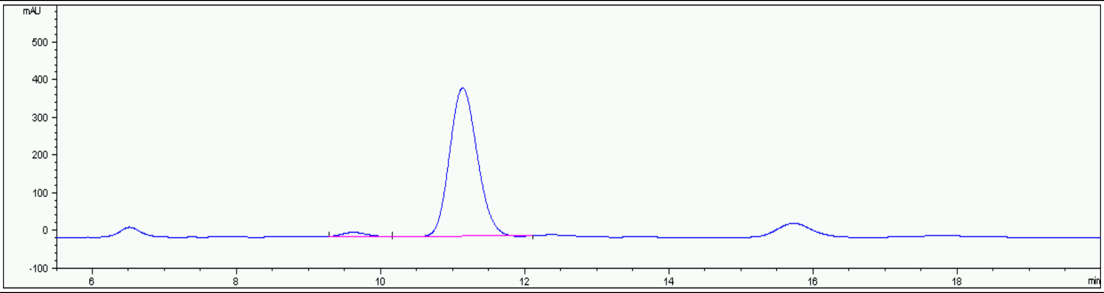

Entry	Retention Time	Area	Height	%Area
1	9.615	313.9	12.3	2.876
2	11.133	10599.1	395.6	97.124

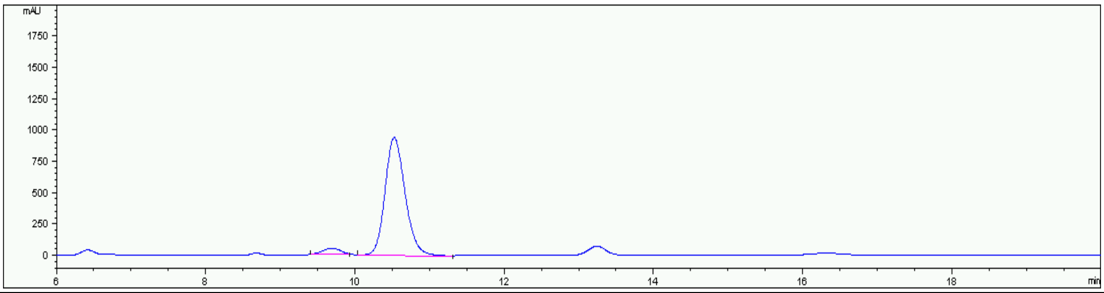

Entry	Retention Time	Area	Height	%Area
1	9.688	913.4	52.2	4.874
2	10.521	17828	942.8	95.126

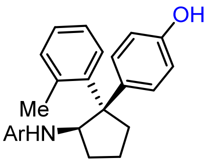

Ar = 2-OBn-5-OMePh

19: white solid; Mp 73.7 - 74.9 °C; 27.8 mg, 58% yield; 94% ee; dr > 19:1; $[\alpha]_D^{22}$ +18.4 (*c* 1.0, $CHCl_3$); ^1H NMR (300 MHz, CD_2Cl_2) δ 7.41 – 7.36 (m, 1H), 7.35 – 7.25 (m, 3H), 7.20 – 7.14 (m, 2H), 7.12 – 7.06 (m, 2H), 7.06 – 7.00 (m, 1H), 6.85 (d, *J* = 7.5 Hz, 2H), 6.69 (d, *J* = 8.6 Hz, 1H), 6.46 (d, *J* = 8.0 Hz, 2H), 6.41 (d, *J* = 2.8 Hz, 1H), 6.09 (dd, *J* = 8.6, 2.8 Hz, 1H), 4.94 – 4.72 (m, 3H), 4.38 – 4.23 (m, 1H), 4.09 (d, *J* = 9.6 Hz, 1H), 3.75 (s, 3H), 3.03 – 2.89 (m, 1H), 2.31 – 2.18 (m, 1H), 2.10 – 1.91 (m, 2H), 1.88 – 1.72 (m, 4H), 1.29 – 1.20 (m, 1H); ^{13}C NMR (75 MHz, CD_2Cl_2) δ 155.0, 153. 7, 147.0, 140.2, 138.4, 136.9, 135.2, 131.6, 129.0, 127.9, 127.4, 127.2, 125.8, 125.7, 125.0, 114.4, 111.7, 97.6, 97.5, 70.7, 58.1, 55.4, 55.0, 36.1, 28.04, 21.06, 19.2; HRMS (ESI) m/z 480.2525 (M+H$^+$), calc. for $C_{32}H_{34}NO_3^+$ 480.2533.

The ee was determined by HPLC analysis: Lux Amylose-1 (4.6 mm i.d. x 250 mm); Hexane/2-propanol = 85/15; flow rate: 1.0 mL/min; 25 °C; 210 nm; retention time: 6.6 min (minor) and 9.4 min (major).

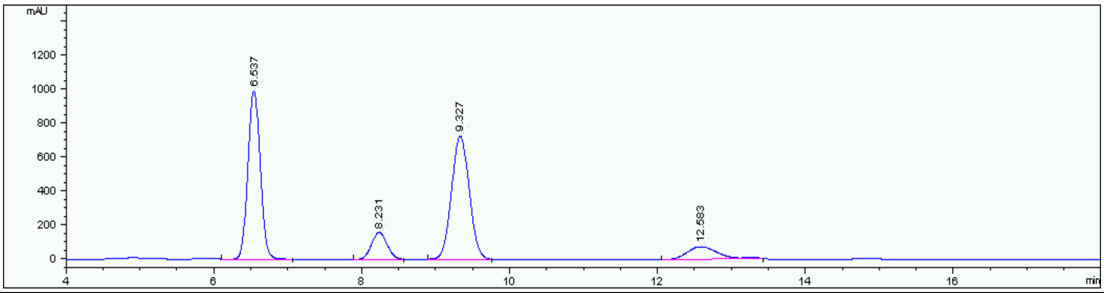

Entry	Retention Time	Area	Height	%Area
1	6.537	11733.2	996.7	40.951
2	8.231	2509.3	163.2	8.758
3	9.327	12263.5	729.4	42.802
4	12.583	2145.5	74.6	7.488

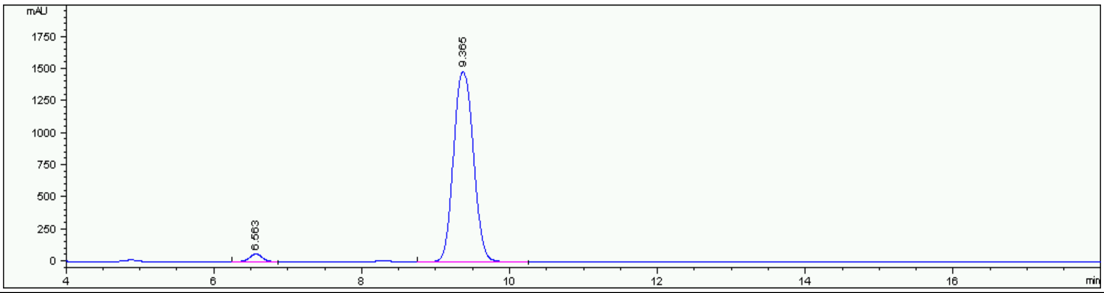

Entry	Retention Time	Area	Height	%Area
1	6.563	795.3	61.6	2.773
2	9.365	27881.3	1485.6	97.227

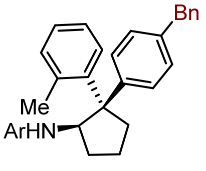

Ar = 2-OBn-5-OMePh

20: white solid; Mp 86.4 - 87.5 °C; 50.3 mg, 91% yield; 94% ee; dr > 19:1; $[\alpha]_D^{22}$ +31.4 (*c* 1.0, $CHCl_3$); ^1H NMR (300 MHz, $CDCl_3$) δ 7.53 – 7.46 (m, 1H), 7.39 – 7.27 (m, 6H), 7.25 – 7.15 (m, 6H), 7.13 – 7.07 (m, 1H), 7.06 – 6.96 (m, 2H), 6.95 – 6.86 (m, 2H), 6.76 (d, *J* = 8.6 Hz, 1H), 6.48 (d, *J* = 2.7 Hz, 1H), 6.17 (dd, *J* = 8.6, 2.7 Hz, 1H), 4.86 (q, *J* = 11.3 Hz, 2H), 4.44 – 4.25 (m, 2H), 3.90 (s, 2H), 3.86 (s, 3H), 3.12 – 3.00 (m, 1H), 2.43 – 2.27 (m, 1H), 2.16 – 1.98 (m, 2H), 1.92 – 1.82 (m, 4H), 1.47 – 1.37 (m, 1H); ^{13}C NMR (75 MHz, $CDCl_3$) δ 155.2, 147.1, 141.2, 141.0, 140.7, 138.8, 138.8, 137.2, 137.1, 132.0, 128.9, 128.6, 128.3, 128.3, 128.2, 127.8, 127.7, 126.4, 126.1, 126.0, 125.6, 112.2, 98.0, 97.4, 71.2, 58.5, 56.0, 55.4, 41.2, 36.2, 28.2, 21.8, 19.7; HRMS (ESI) m/z 554.3041 (M+H$^+$), calc. for $C_{39}H_{40}NO_2^+$ 554.3054.

The ee was determined by HPLC analysis: Lux Cellulose-1 (4.6 mm i.d. x 250 mm); Hexane/2-propanol = 95/5; flow rate: 1.0 mL/min; 25 °C; 210 nm; retention time: 11.7 min (minor) and 20.7 min (major).

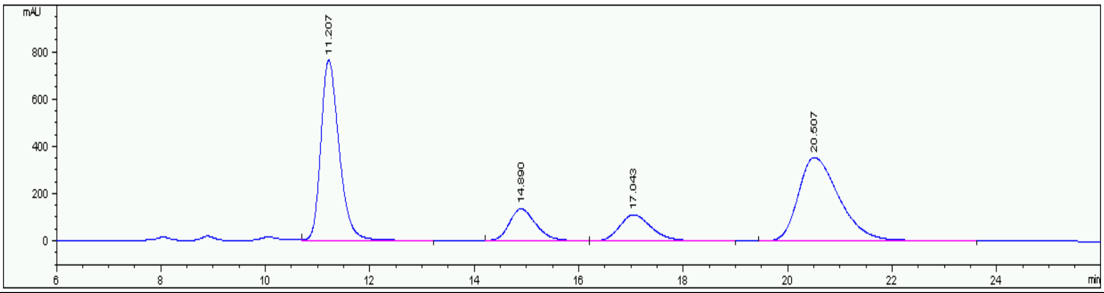

Entry	Retention Time	Area	Height	%Area
1	11.207	19160.8	766.3	39.167
2	14.89	5100.3	138.1	10.426
3	17.043	4984.5	113.2	10.189
4	20.507	19675	355.9	40.218

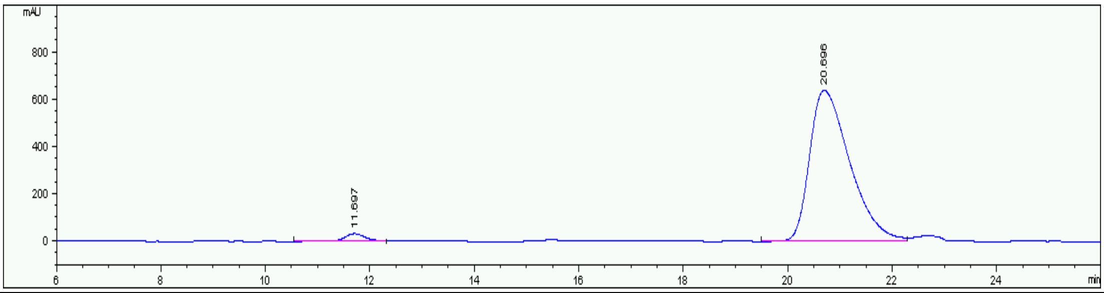

Entry	Retention Time	Area	Height	%Area
1	11.697	993.5	33.3	2.809
2	20.696	34367.5	640	97.191

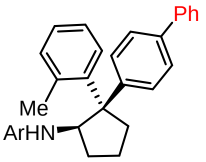

Ar = 2-OBn-5-OMePh

21: white solid; Mp 113.5 - 114.8 °C; 46.9 mg, 87% yield; 94% ee; dr > 19:1; $[\alpha]_D^{22}$ +13.9 (*c* 1.0, $CHCl_3$); ^{1}H NMR (300 MHz, $CDCl_3$) δ 7.53 – 7.33 (m, 7H), 7.25 – 7.21 (m, 1H), 7.19 – 7.04 (m, 10H), 6.73 (d, *J* = 8.6 Hz, 1H), 6.49 (d, *J* = 2.7 Hz, 1H), 6.14 (dd, *J* = 8.6, 2.7 Hz, 1H), 4.80 (q, *J* = 11.0 Hz, 2H), 4.42 – 4.35 (m, 1H), 3.82 (s, 3H), 3.16 – 3.03 (m, 1H), 2.39 – 2.30 (m, 1H), 2.15 – 2.01 (m, 2H), 1.89 – 1.80 (m, 4H), 1.42 – 1.34 (m, 1H).^{13}C NMR (75 MHz, $CDCl_3$) δ 155.2, 146.9, 142.5, 140.9, 140.5, 138.8, 138.5, 137.2, 136.8, 132.1, 128.6, 128.5, 128.2, 127.8, 127.8, 127.1, 127.0, 126.7, 126.4, 126.3, 125.6, 112.0, 98.3, 97.9, 71.2, 58.8, 56.2, 55.5, 36.1, 28.3, 21.8, 19.8; HRMS (ESI) m/z 540.2888 (M+H$^+$), calc. for $C_{38}H_{38}NO_2^+$ 540.2897.

The ee was determined by HPLC analysis: Lux Amylose-1 (4.6 mm i.d. x 250 mm); Hexane/2-propanol = 98/2; flow rate: 1.0 mL/min; 25 °C; 210 nm; retention time: 6.1 min (major) and 6.7 min (minor).

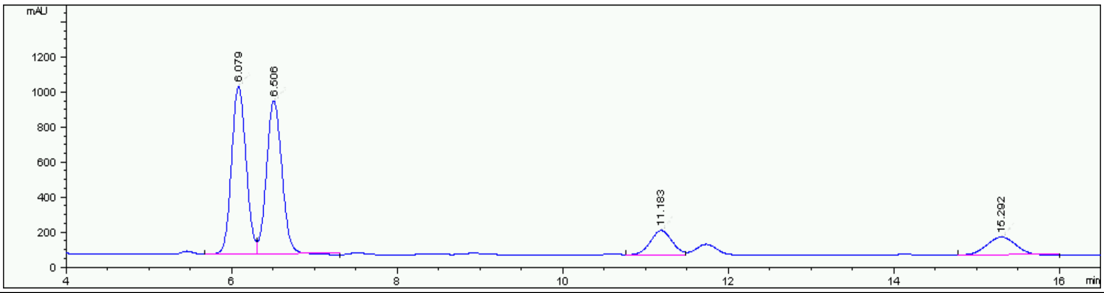

Entry	Retention Time	Area	Height	%Area
1	6.079	11733.7	959.4	41.334
2	6.506	11334.1	875	39.926
3	11.183	2731.6	143.3	9.623
4	15.292	2588.3	103.8	9.118

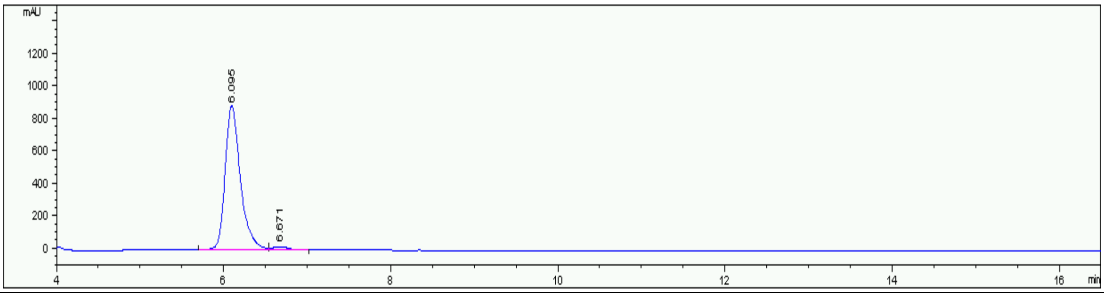

Entry	Retention Time	Area	Height	%Area
1	6.095	11509.3	884	96.840
2	6.671	375.6	25.5	3.160

Determination of the relationship between the ee of CPA C3 and the ee of product 3e

The ee of **C3** was determined by HPLC analysis: CHIRALPAK QN-AX (4.6 mm i.d. x 150 mm); methanol/acetonitrile/acetic acid/triethylamine = 50/50/2/1.6; flow rate 0.5 mL/min; 25 ^{o}C; 254 nm; retention time: 4.8 min, 7.2 min. (upper spectrum)

The ee of **3e** was determined by HPLC analysis: CHIRALPAK IE (4.6 mm i.d. x 250 mm); hexane/2-propanol = 60/40; flow rate 1.0 mL/min; 25 ^{o}C; 210 nm; retention time: 7.4 min, 12.8 min. (below spectrum)

A:

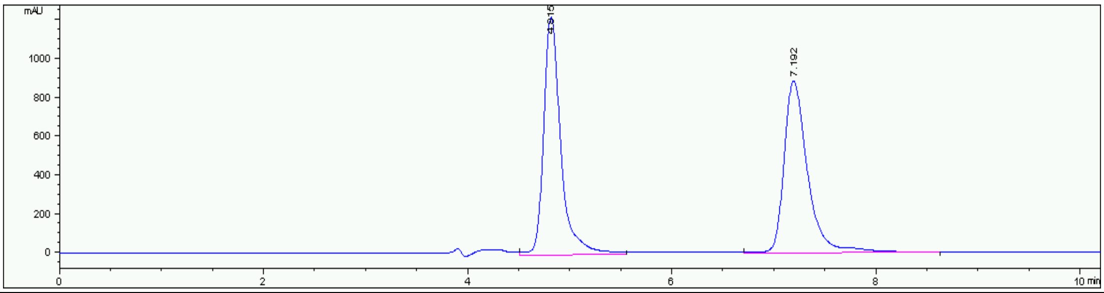

Entry	Retention Time	Area	Height	%Area
1	4.815	14663.9	1228.8	50.077
2	7.192	14619	891	49.923

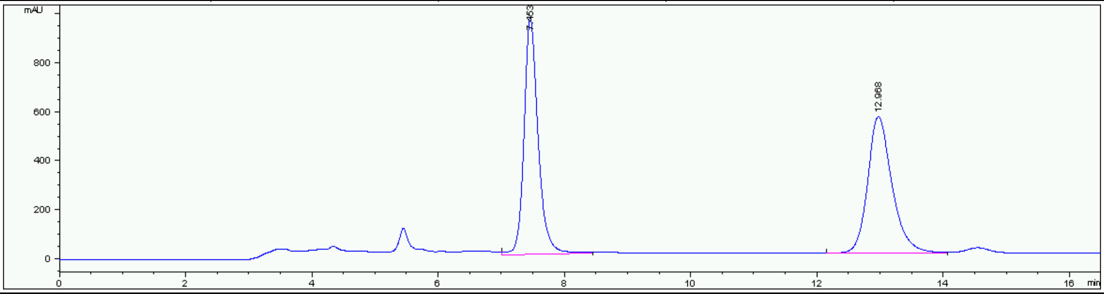

Entry	Retention Time	Area	Height	%Area
1	7.453	15246.5	969.7	50.396
2	12.968	15007	556.9	49.604

B:

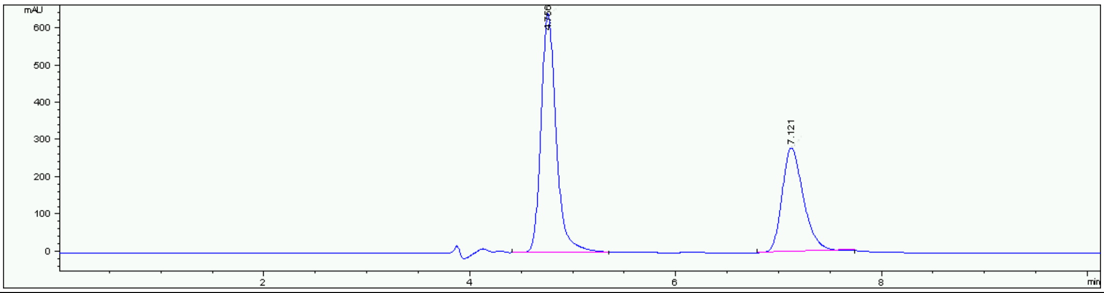

Entry	Retention Time	Area	Height	%Area
1	4.756	6541.2	640.6	62.241
2	7.121	3968.4	278	37.759

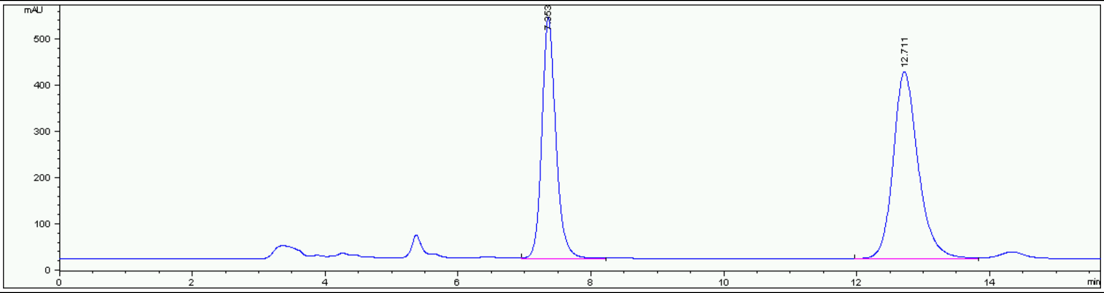

Entry	Retention Time	Area	Height	%Area
1	7.353	7681.1	523	41.964
2	12.711	10622.9	404.9	58.036

C:

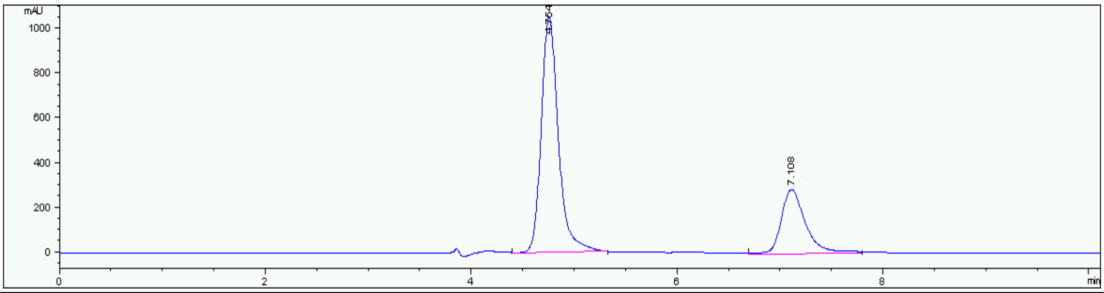

Entry	Retention Time	Area	Height	%Area
1	4.754	12259.8	1055.6	71.442
2	7.108	4900.6	290	28.558

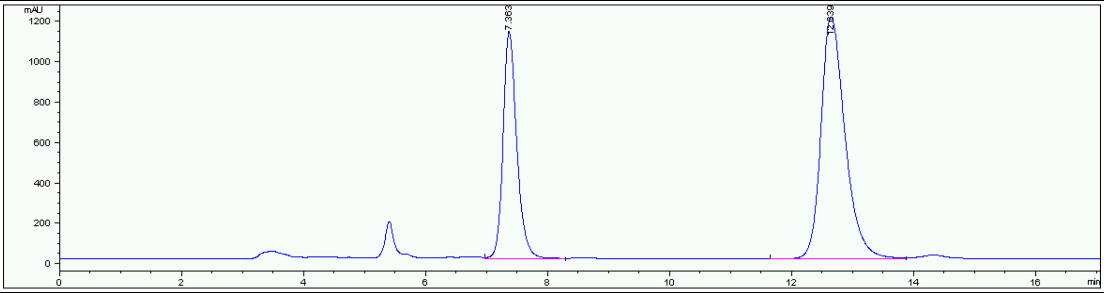

Entry	Retention Time	Area	Height	%Area
1	7.363	17728.4	1128	34.987
2	12.639	32943.3	1198.9	65.013

D:

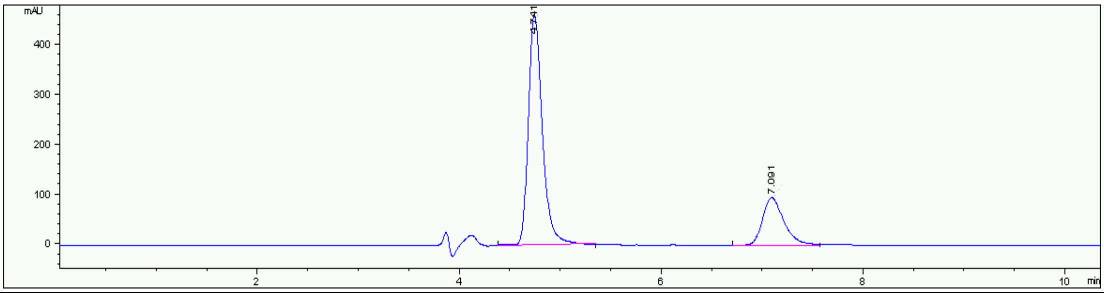

Entry	Retention Time	Area	Height	%Area
1	4.741	4479.2	464.3	75.583
2	7.091	1447	96.1	24.417

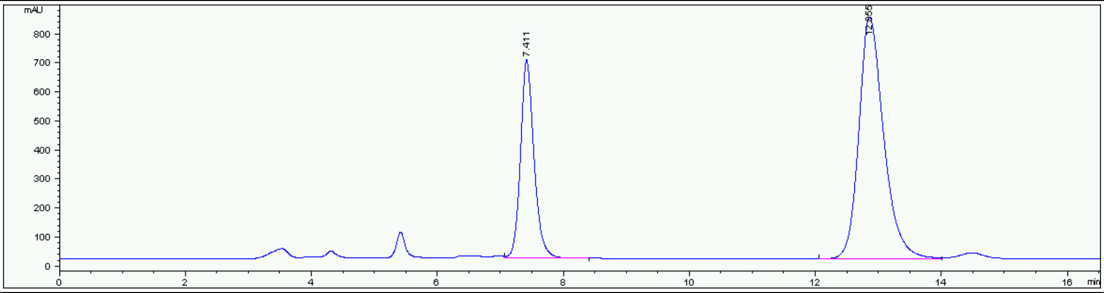

Entry	Retention Time	Area	Height	%Area
1	7.411	10353.8	685.6	31.169
2	12.855	22865	833.1	68.831

E:

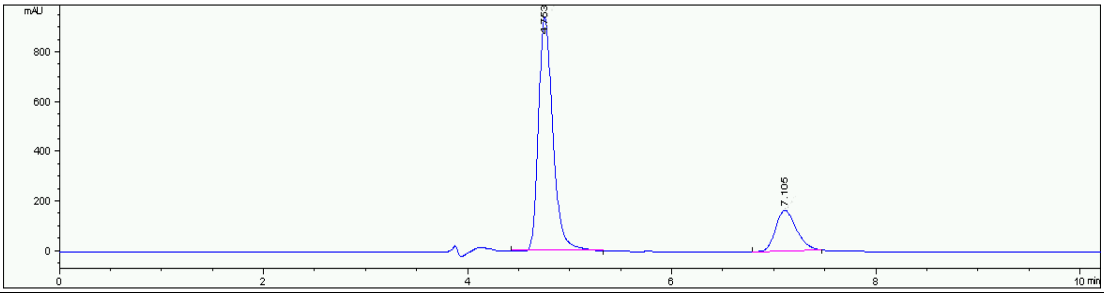

Entry	Retention Time	Area	Height	%Area
1	4.753	9231.8	942.5	79.260
2	7.105	2415.7	166.6	20.740

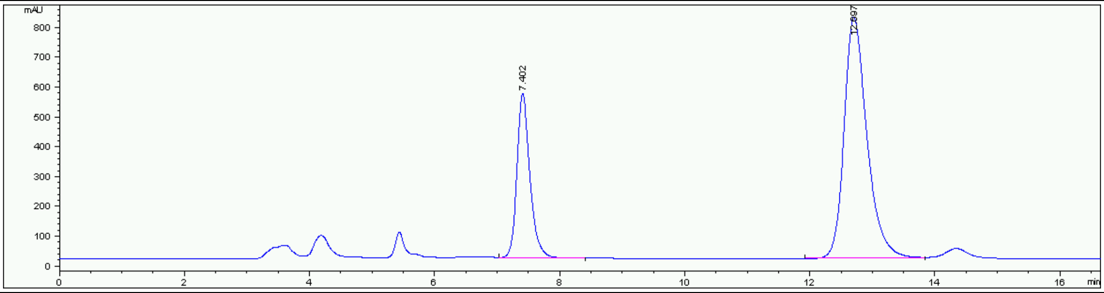

Entry	Retention Time	Area	Height	%Area
1	7.402	8087.2	552.6	28.053
2	12.697	20741.7	810.4	71.947

F:

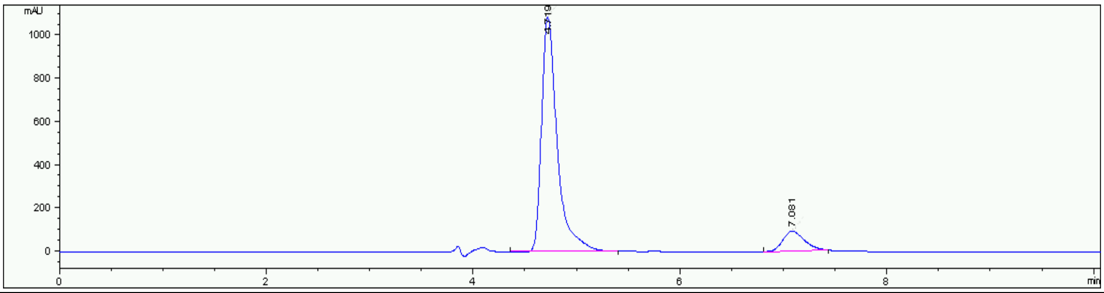

Entry	Retention Time	Area	Height	%Area
1	4.719	11547	1089.2	88.936
2	7.081	1436.4	95.3	11.064

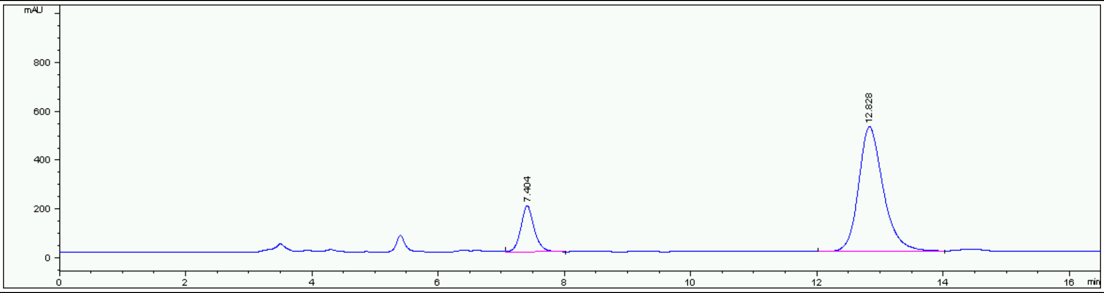

Entry	Retention Time	Area	Height	%Area
1	7.404	2873.6	189.6	16.997
2	12.828	14033.3	511.8	83.003

G:

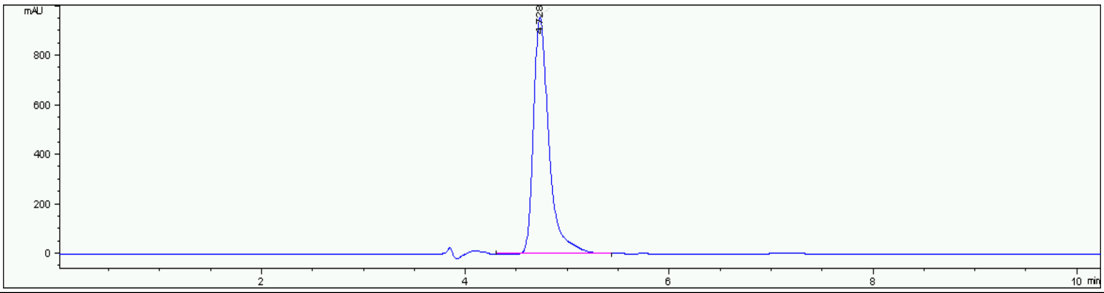

Entry	Retention Time	Area	Height	%Area
1	4.728	10128.9	957	100.000

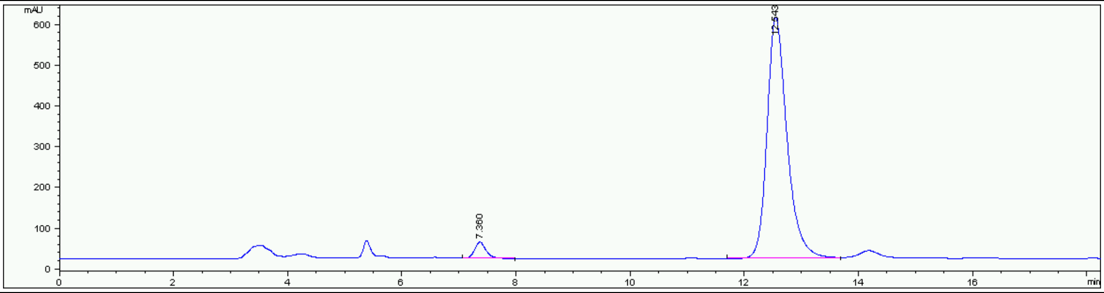

Entry	Retention Time	Area	Height	%Area
1	7.36	577.7	40.6	3.820
2	12.543	14543.9	593.4	96.180

8. Copies of NMR spectra

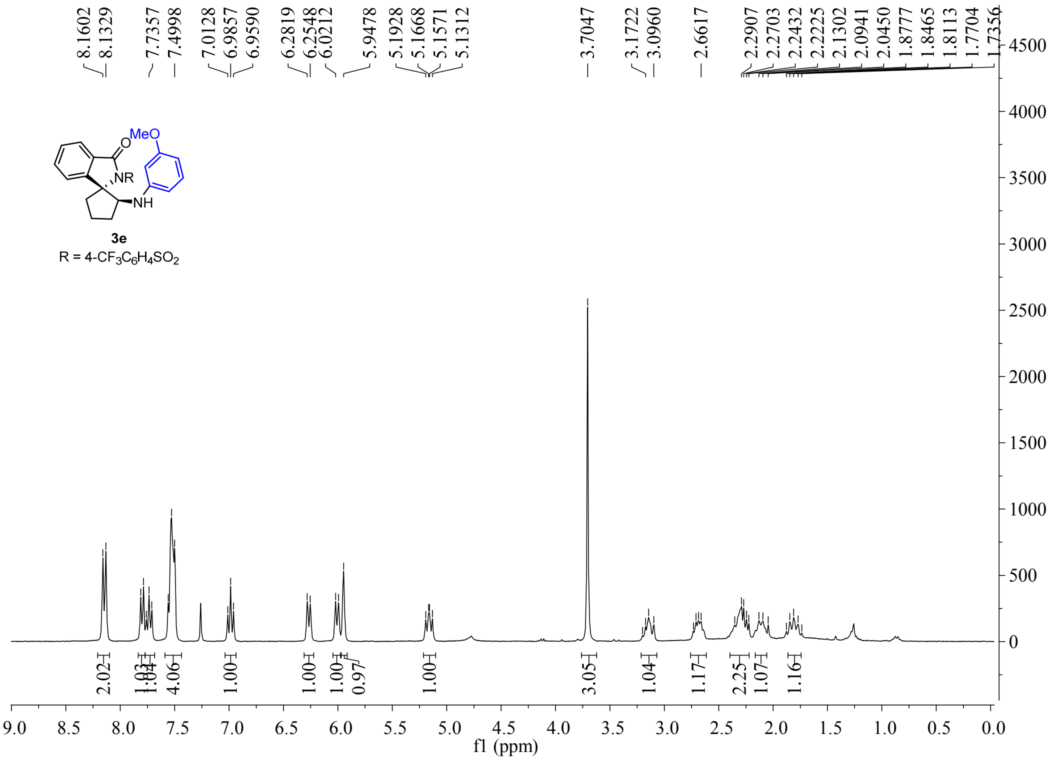

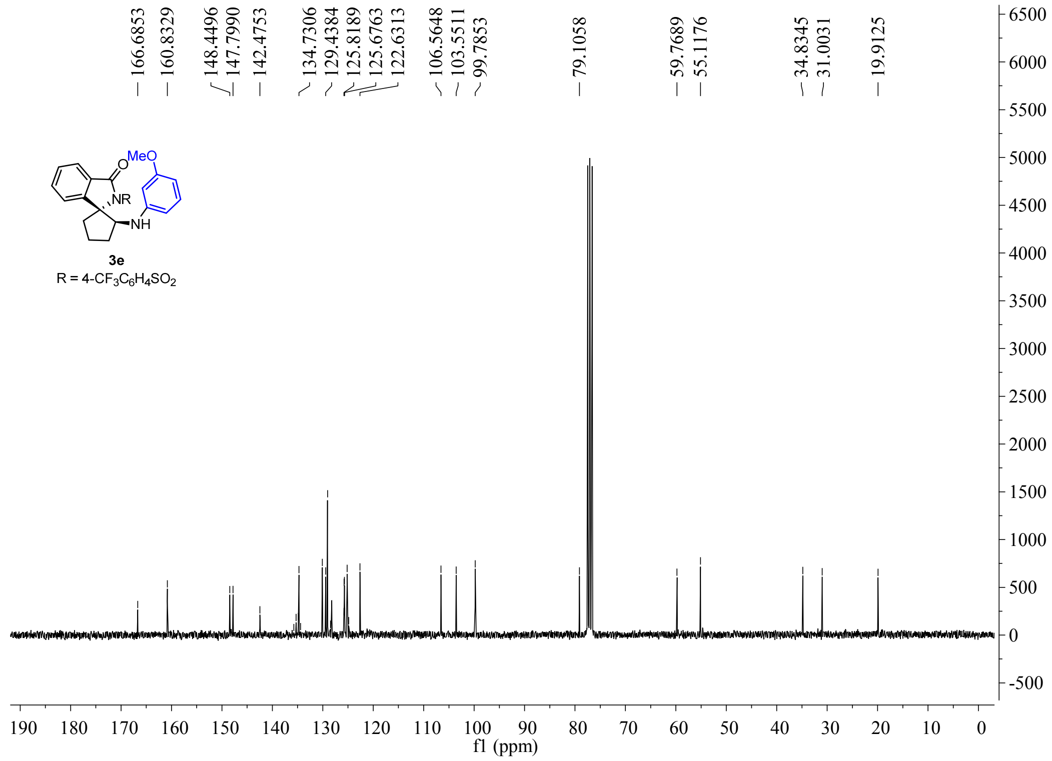

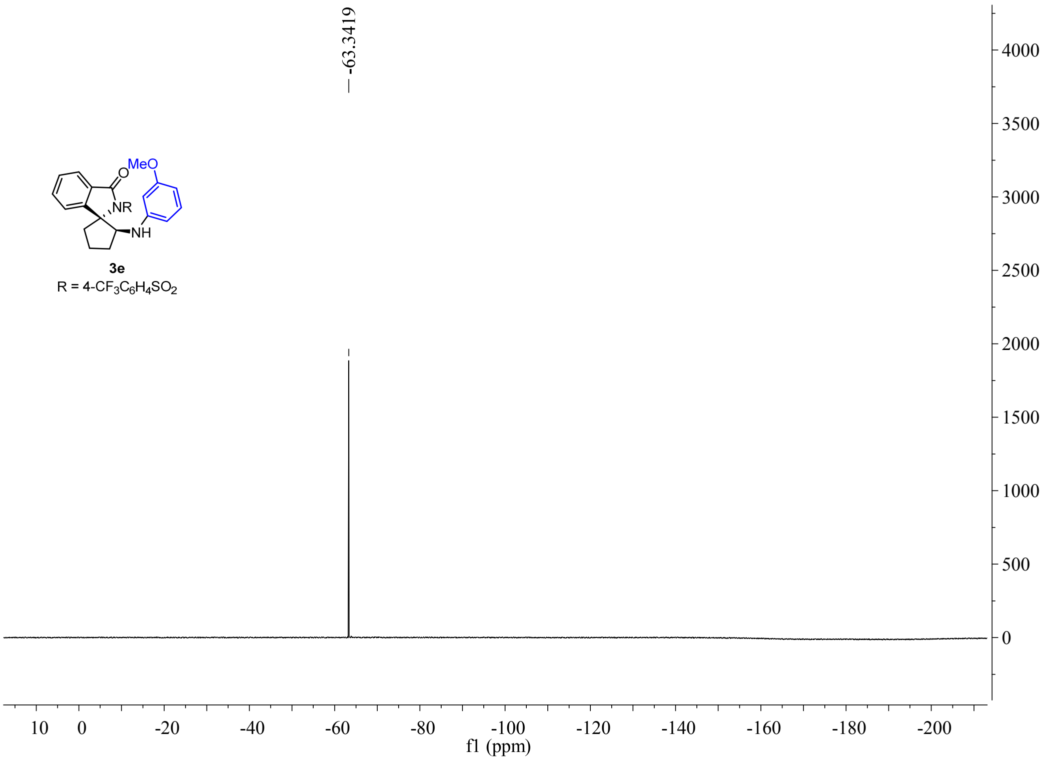
-63.3419
MeO
NR
NH
3e
R = 4-CF3C6H4SO2
f1 (ppm)

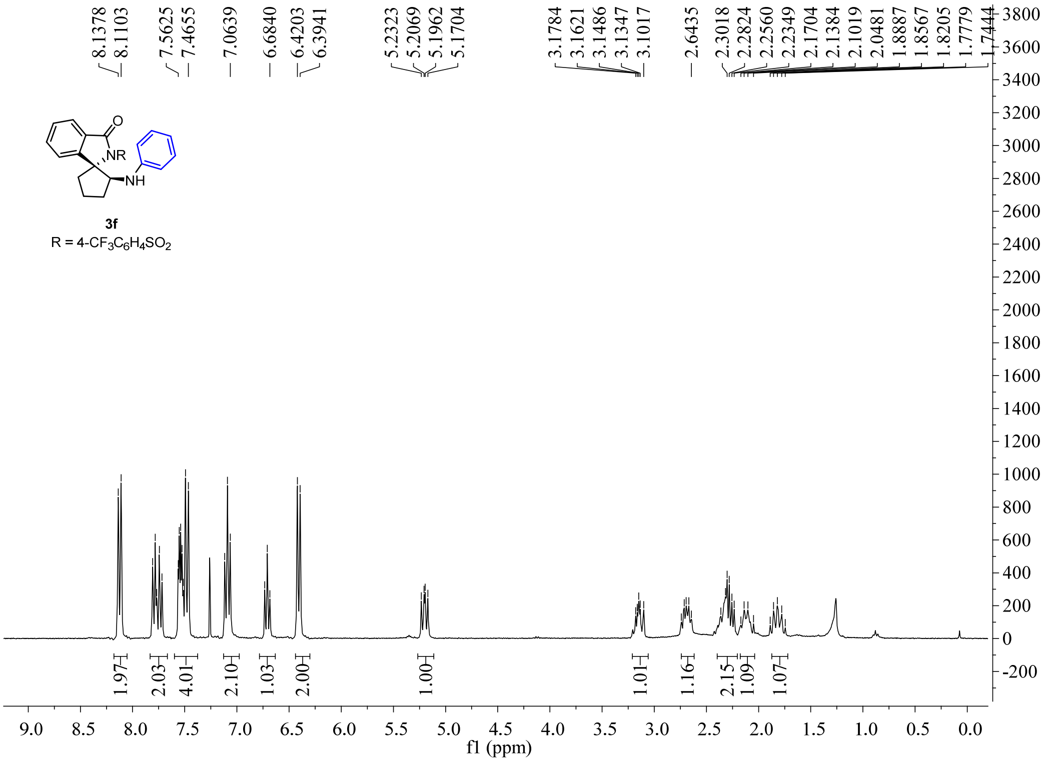
8.1378
8.1103
7.5625
7.4655
7.0639
6.6840
6.4203
6.3941
5.2323
5.2069
5.1962
5.1704
3.1784
3.1621
3.1486
3.1347
3.1017
2.6435
2.3018
2.2824
2.2560
2.2349
2.1704
2.1384
2.1019
2.0481
1.8887
1.8567
1.8205
1.7779
1.7444
NR
NH
3f
R = 4-CF3C6H4SO2
1.97
2.03
4.01
2.10
1.03
2.00
1.00
1.01
1.16
2.15
1.09
1.07
f1 (ppm)

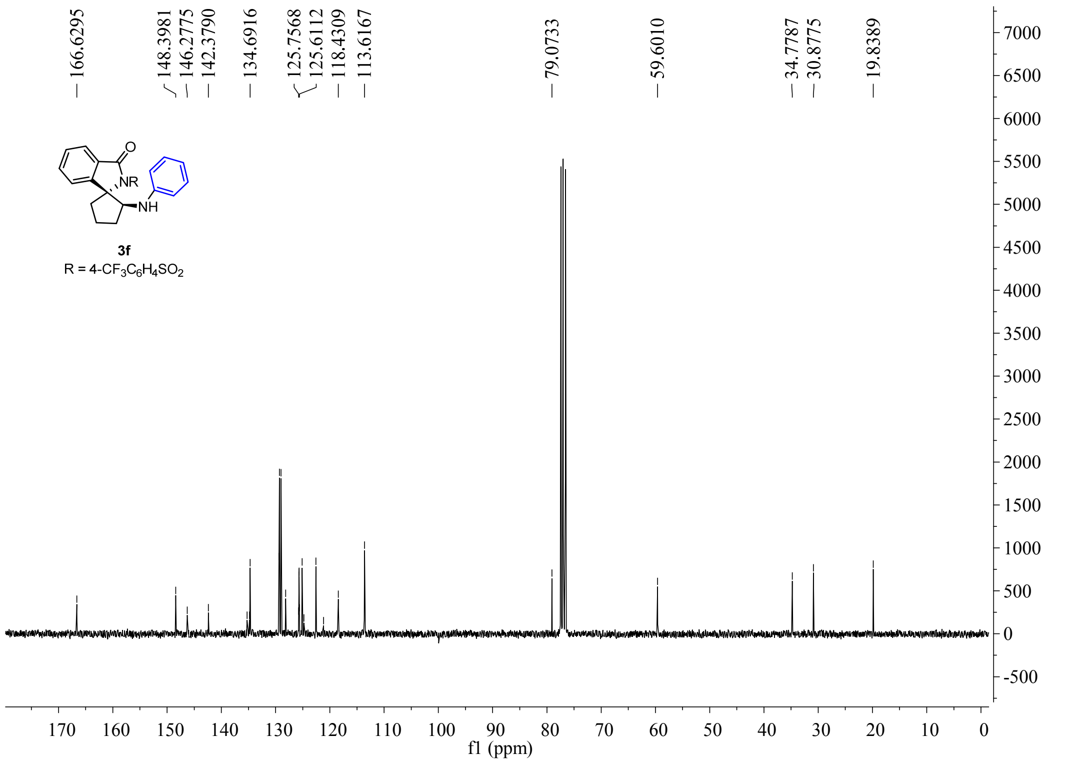
166.6295
148.3981
146.2775
142.3790
134.6916
125.7568
125.6112
118.4309
113.6167
79.0733
59.6010
34.7787
30.8775
19.8389
O
NR
NH
3f
R = 4-CF3C6H4SO2
f1 (ppm)

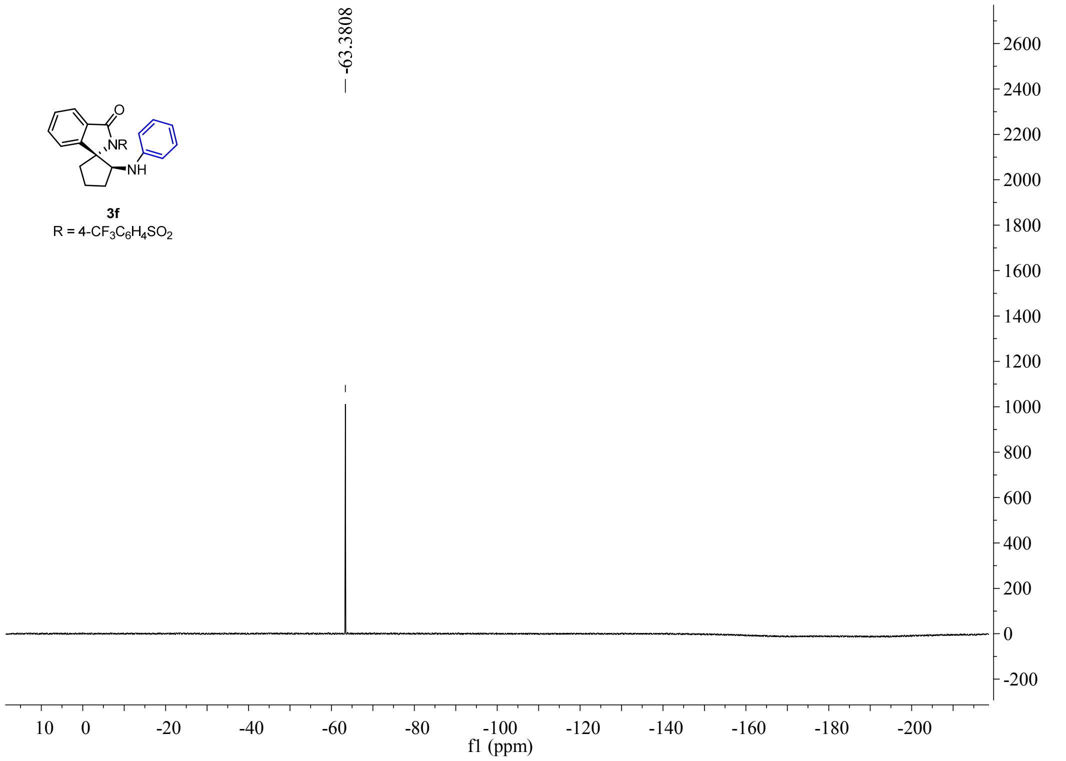
-63.3808
O
NR
NH
3f
R = 4-CF3C6H4SO2
f1 (ppm)

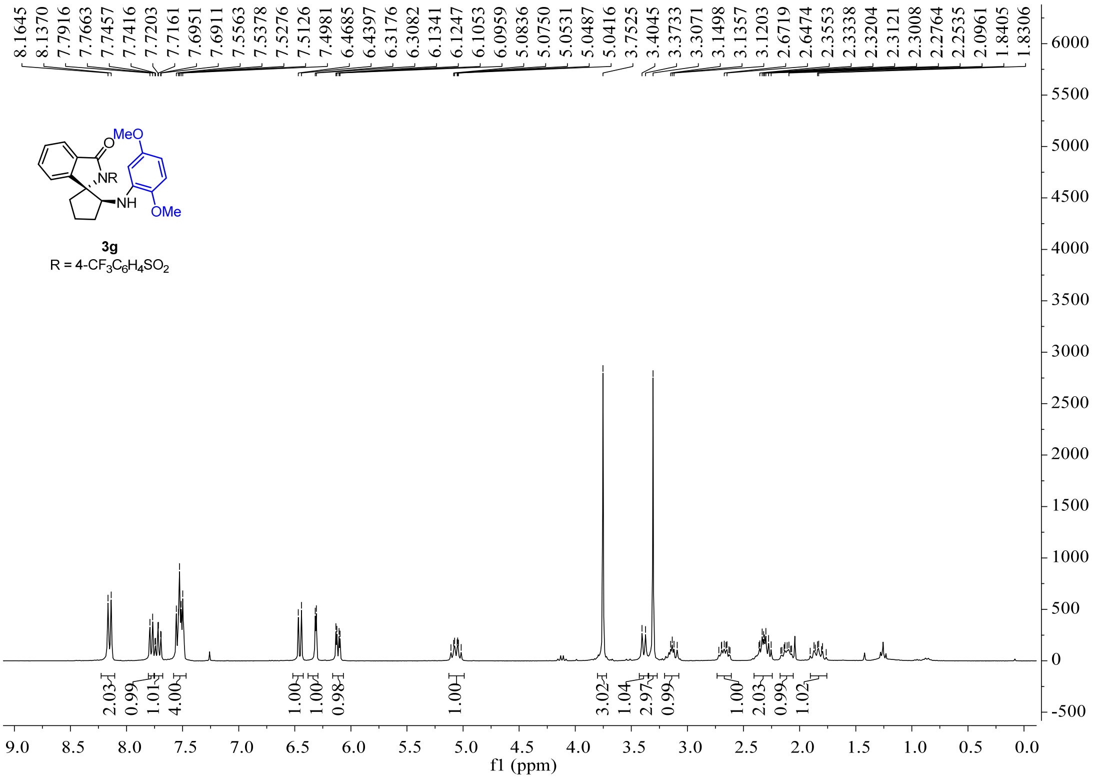
8.1645 8.1370 7.7916 7.7663 7.7457 7.7416 7.7203 7.7161 7.6951 7.6911 7.5563 7.5378 7.5276 7.5126 7.4981 6.4685 6.4397 6.3176 6.3082 6.1341 6.1247 6.1053 6.0959 5.0836 5.0750 5.0531 5.0487 5.0416 3.7525 3.4045 3.3733 3.3071 3.1498 3.1357 3.1203 2.6719 2.6474 2.3553 2.3338 2.3204 2.3121 2.3008 2.2764 2.2535 2.0961 1.8405 1.8306
MeO
O
NR
NH
OMe
3g
R = 4-CF3C6H4SO2
2.03 0.99 1.01 4.00 1.00 1.00 0.98 1.00 3.02 1.04 2.97 0.99 1.00 2.03 0.99 1.02
f1 (ppm)

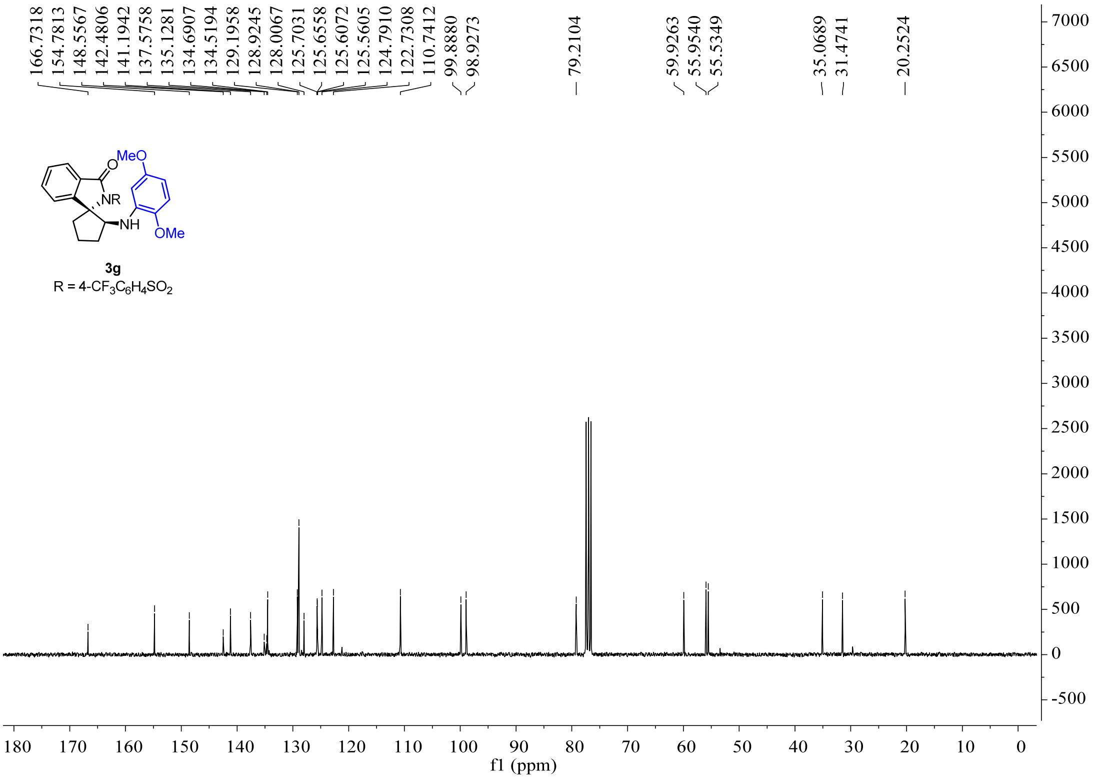
166.7318 154.7813 148.5567 142.4806 141.1942 137.5758 135.1281 134.6907 134.5194 129.1958 128.9245 128.0067 125.7031 125.6558 125.6072 125.5605 124.7910 122.7308 110.7412 99.8880 98.9273 79.2104 59.9263 55.9540 55.5349 35.0689 31.4741 20.2524
MeO
O
NR
NH
OMe
3g
R = 4-CF3C6H4SO2
f1 (ppm)

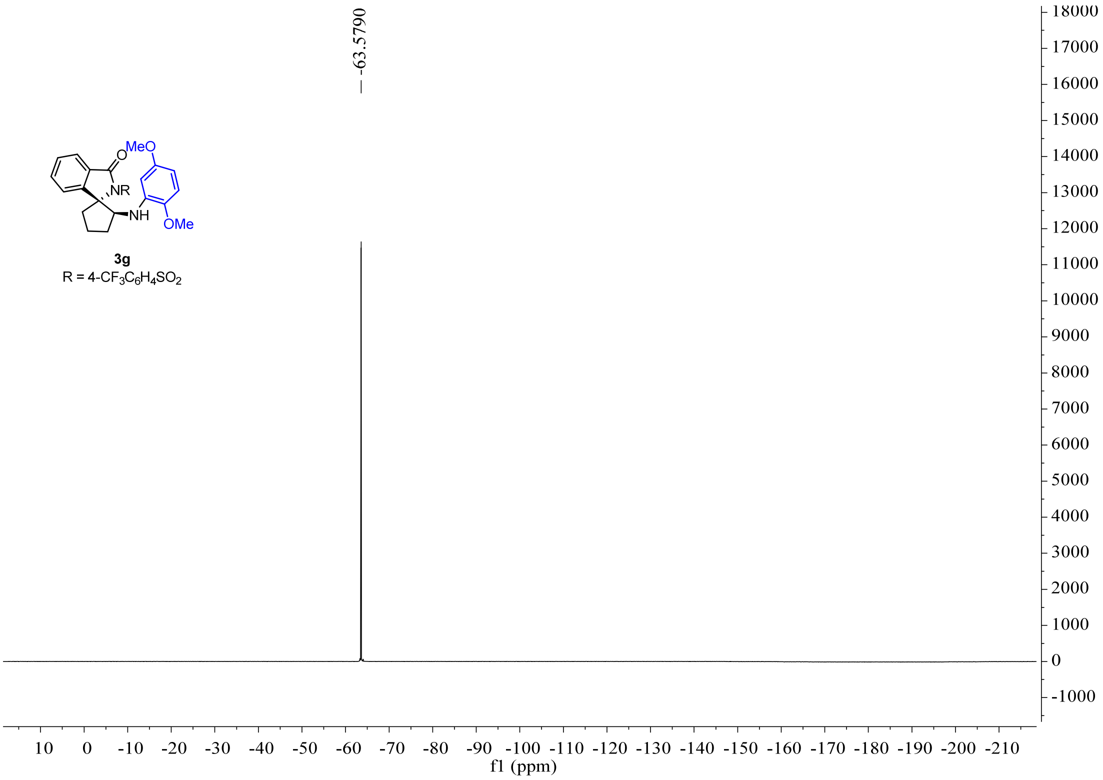
-63.5790
MeO
O
NR
NH
OMe
3g
R = 4-CF3C6H4SO2
f1 (ppm)

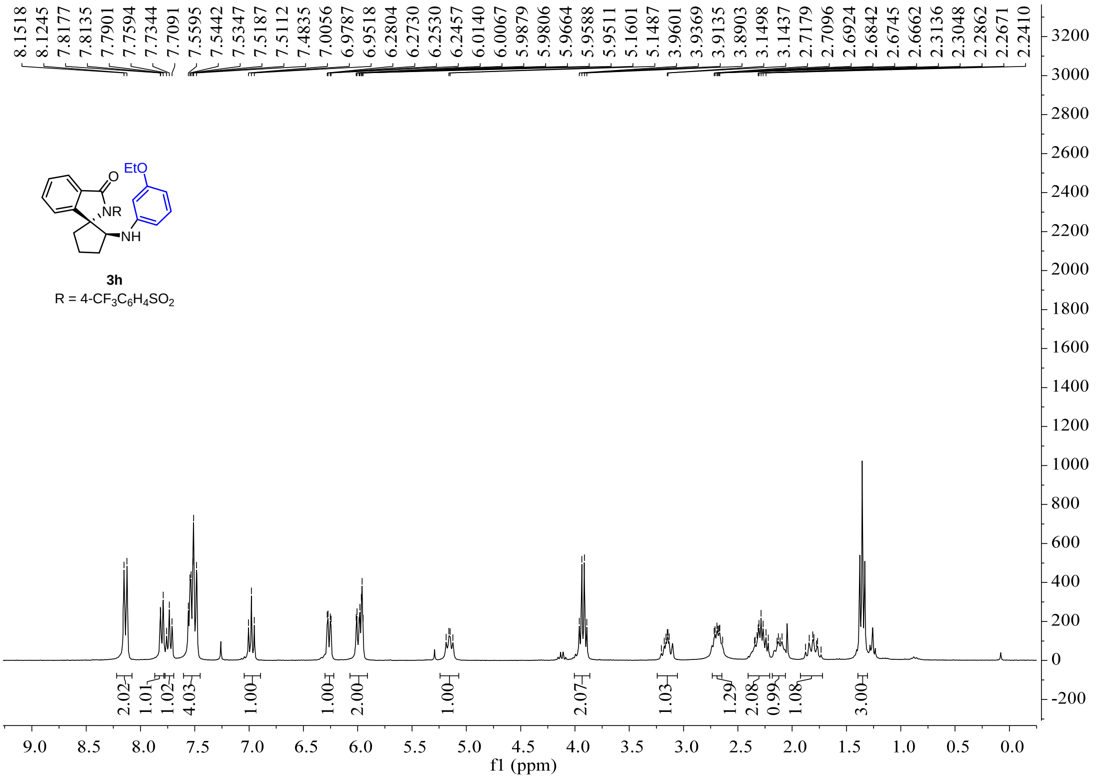
8.1518
8.1245
7.8177
7.8135
7.7901
7.7594
7.7344
7.7091
7.5595
7.5442
7.5347
7.5187
7.5112
7.4835
7.0056
6.9787
6.9518
6.2804
6.2730
6.2530
6.2457
6.0140
6.0067
5.9879
5.9806
5.9664
5.9588
5.9511
5.1601
5.1487
3.9601
3.9369
3.9135
3.8903
3.1498
3.1437
2.7179
2.7096
2.6924
2.6842
2.6745
2.6662
2.3136
2.3048
2.2862
2.2671
2.2410
EtO
O
NR
NH
3h
R = 4-CF3C6H4SO2
2.02
1.01
1.02
4.03
1.00
1.00
2.00
1.00
2.07
1.03
1.29
2.08
0.99
1.08
3.00
f1 (ppm)

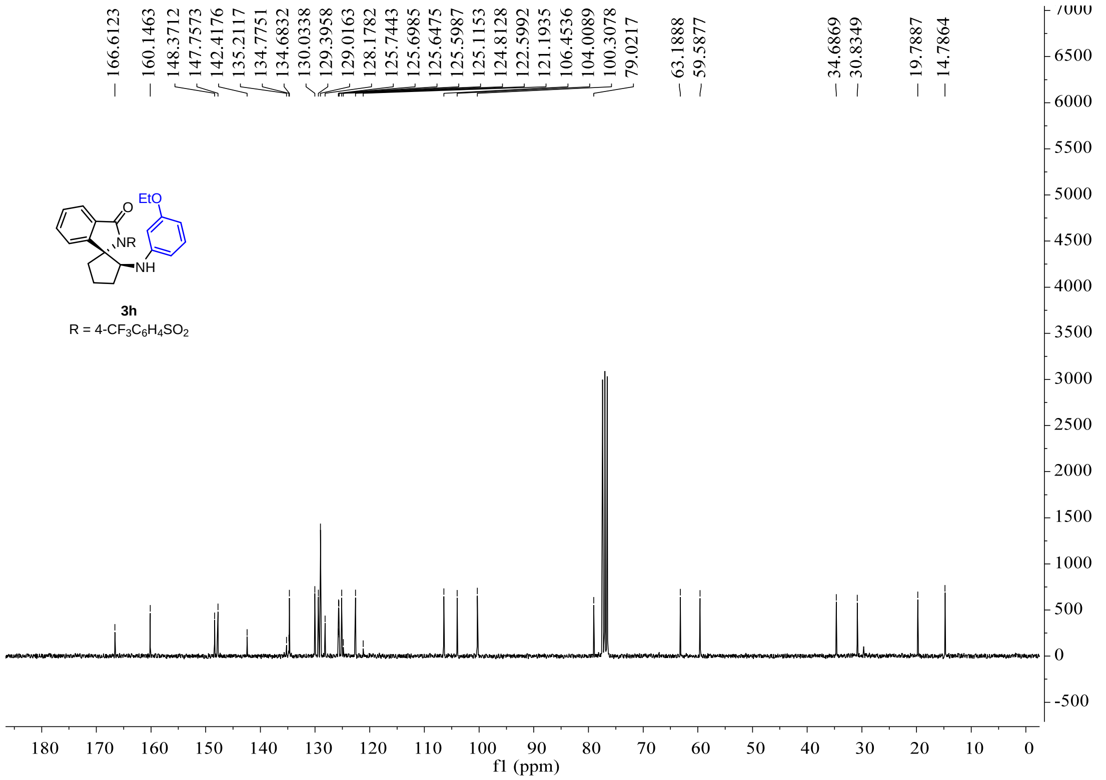
166.6123
160.1463
148.3712
147.7573
142.4176
135.2117
134.7751
134.6832
130.0338
129.3958
129.0163
128.1782
125.7443
125.6985
125.6475
125.5987
125.1153
124.8128
122.5992
121.1935
106.4536
104.0089
100.3078
79.0217
63.1888
59.5877
34.6869
30.8349
19.7887
14.7864
EtO
O
NR
NH
3h
R = 4-CF3C6H4SO2
f1 (ppm)

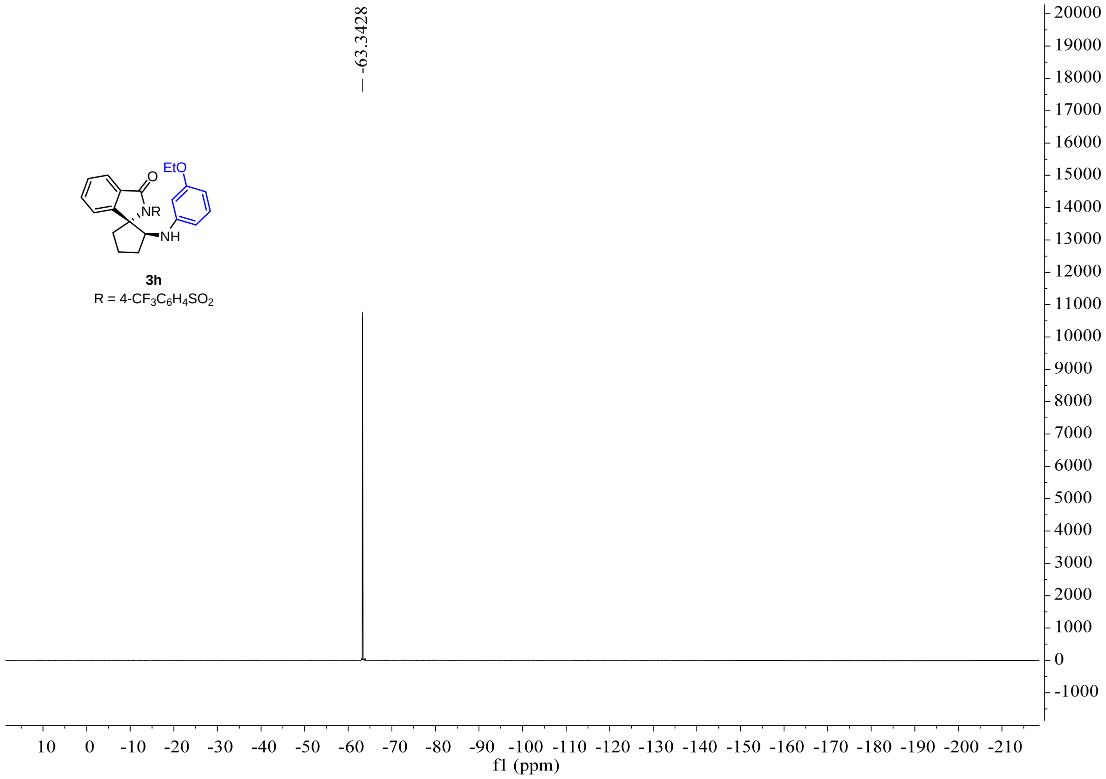
-63.3428
EtO
O
NR
NH
3h
R = 4-CF3C6H4SO2
f1 (ppm)

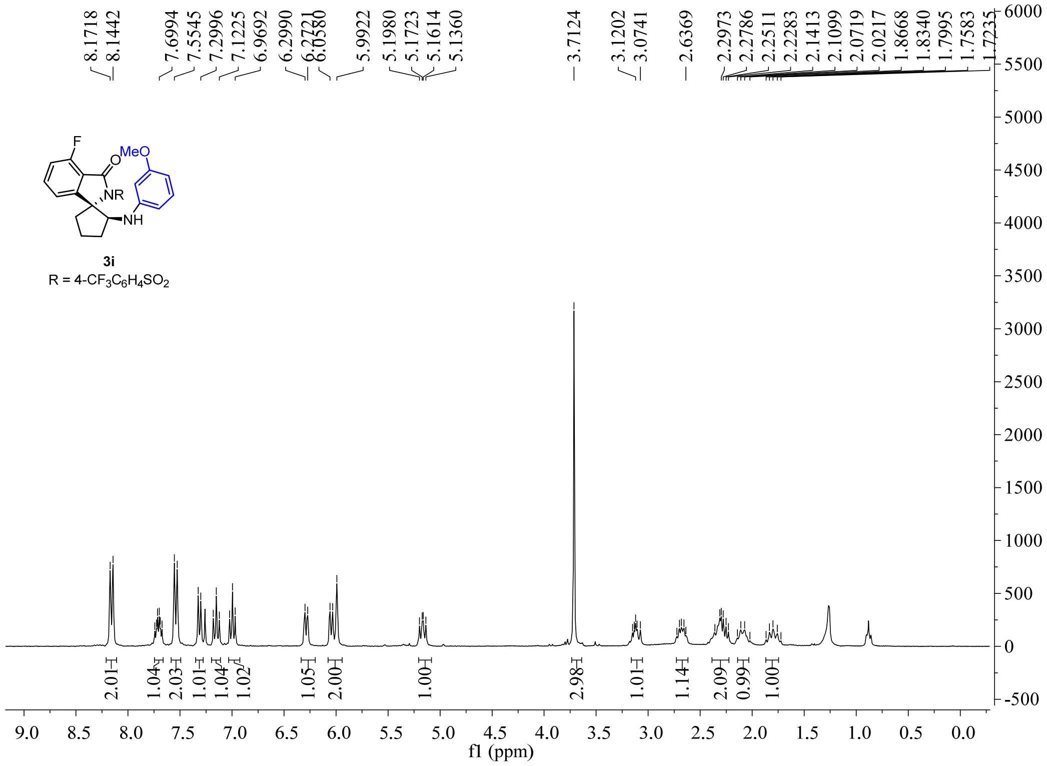

3i
R = 4-CF3C6H4SO2
f1 (ppm)

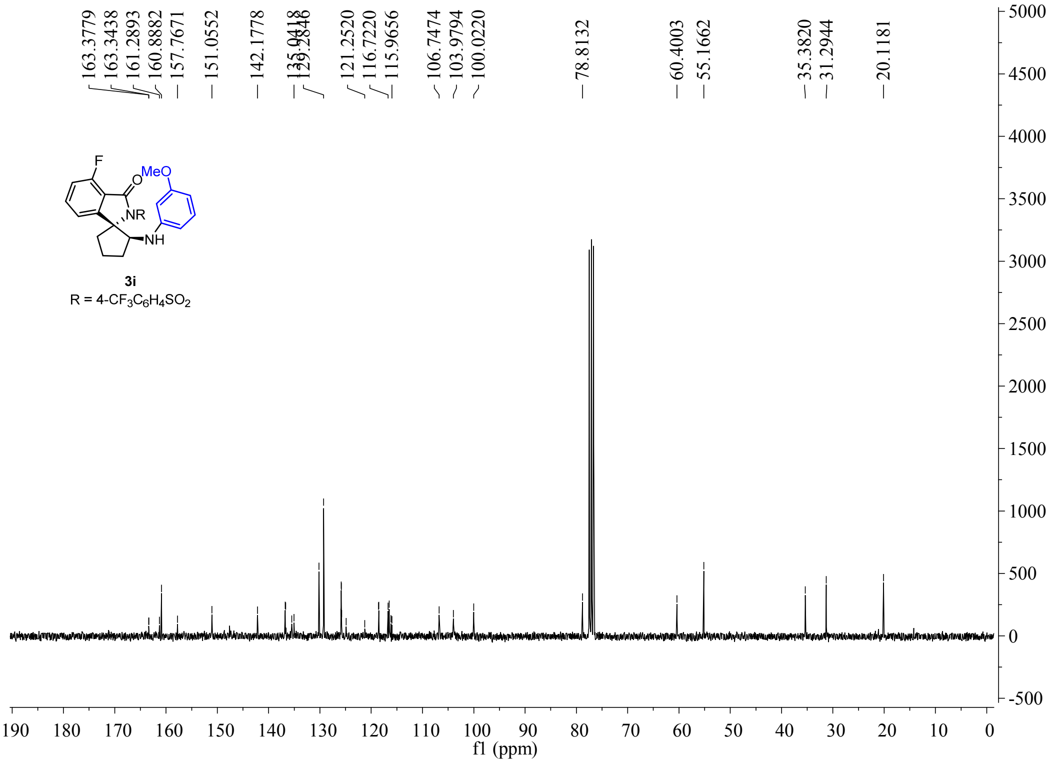

3i
R = 4-CF3C6H4SO2
f1 (ppm)

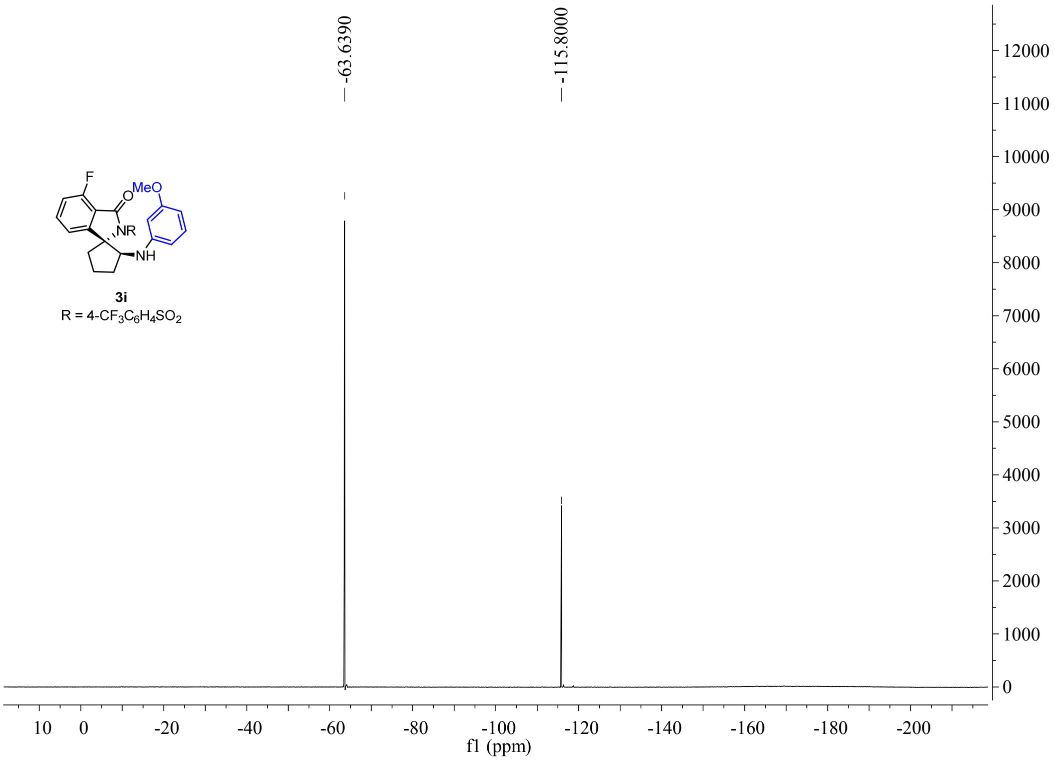
-63.6390
-115.8000
F
O
MeO
NR
NH
3i
R = 4-CF3C6H4SO2
f1 (ppm)

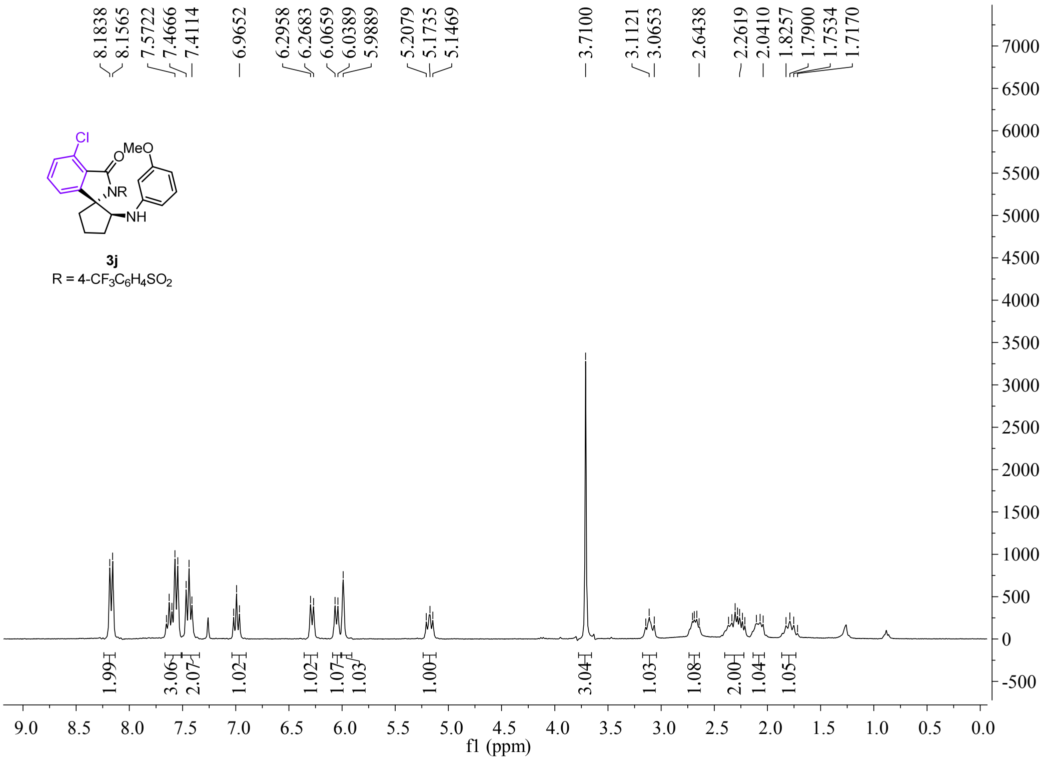
8.1838
8.1565
7.5722
7.4666
7.4114
6.9652
6.2958
6.2683
6.0659
6.0389
5.9889
5.2079
5.1735
5.1469
3.7100
3.1121
3.0653
2.6438
2.2619
2.0410
1.8257
1.7900
1.7534
1.7170
Cl
O
MeO
NR
NH
3j
R = 4-CF3C6H4SO2
1.99
3.06
2.07
1.02
1.02
1.07
1.03
1.00
3.04
1.03
1.08
2.00
1.04
1.05
f1 (ppm)

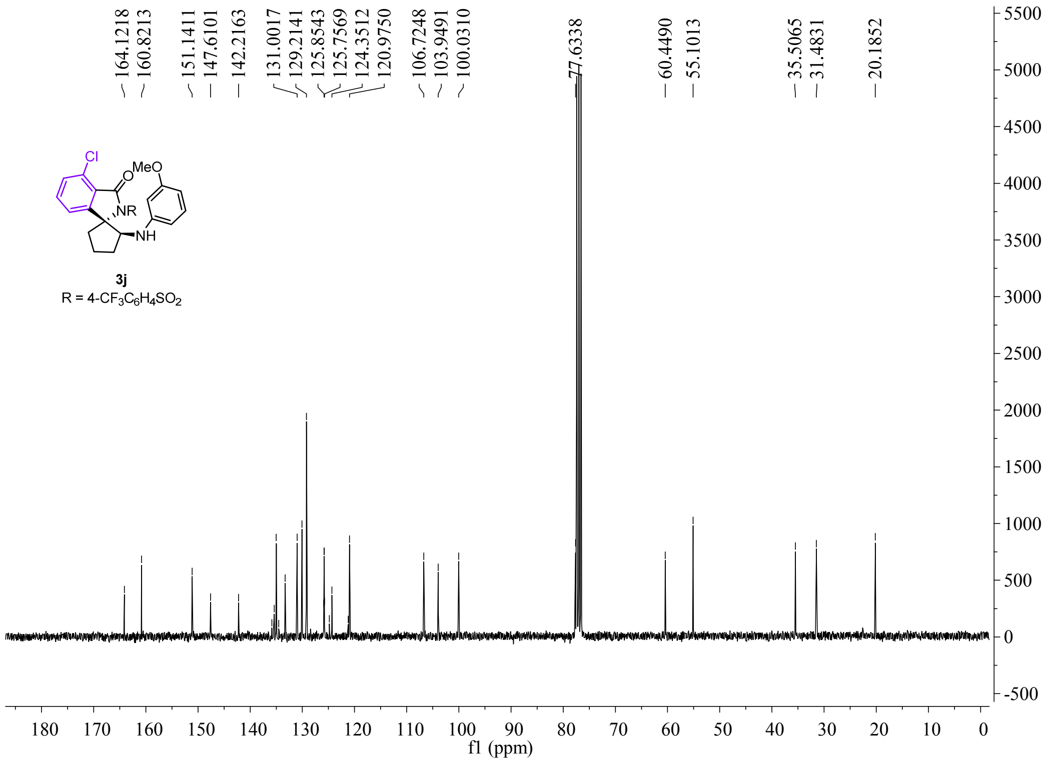
164.1218
160.8213
151.1411
147.6101
142.2163
131.0017
129.2141
125.8543
125.7569
124.3512
120.9750
106.7248
103.9491
100.0310
77.6338
60.4490
55.1013
35.5065
31.4831
20.1852
Cl
MeO
O
NR
NH
3j
R = 4-CF3C6H4SO2
f1 (ppm)

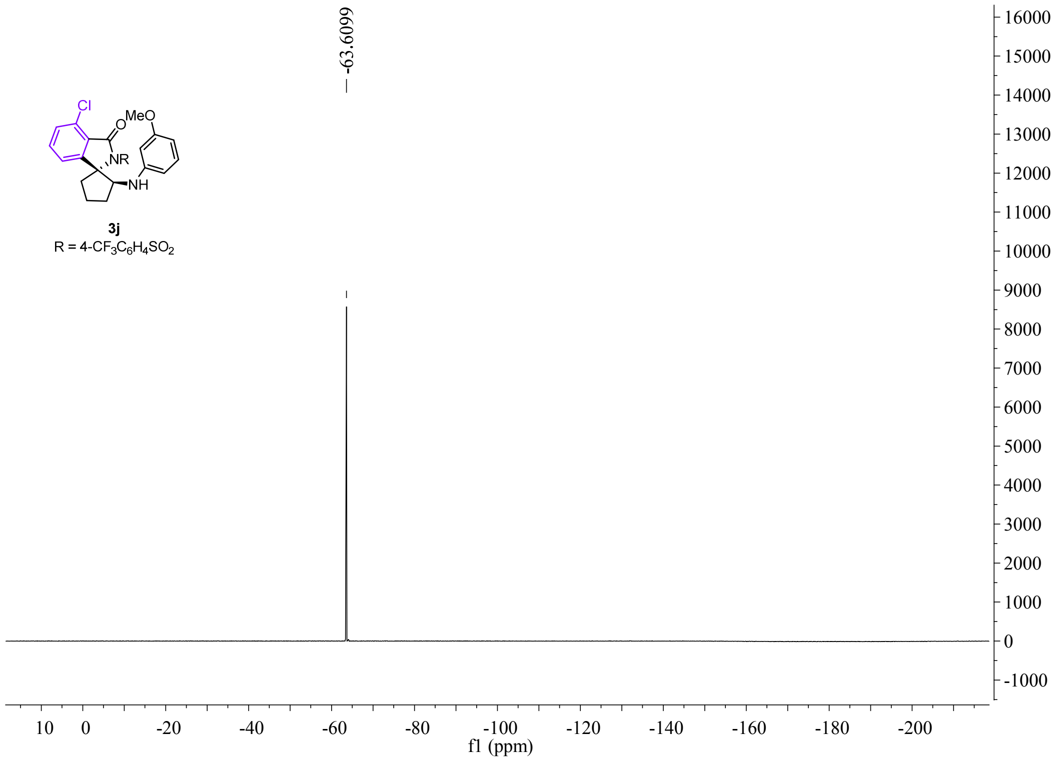
-63.6099
Cl
MeO
O
NR
NH
3j
R = 4-CF3C6H4SO2
f1 (ppm)

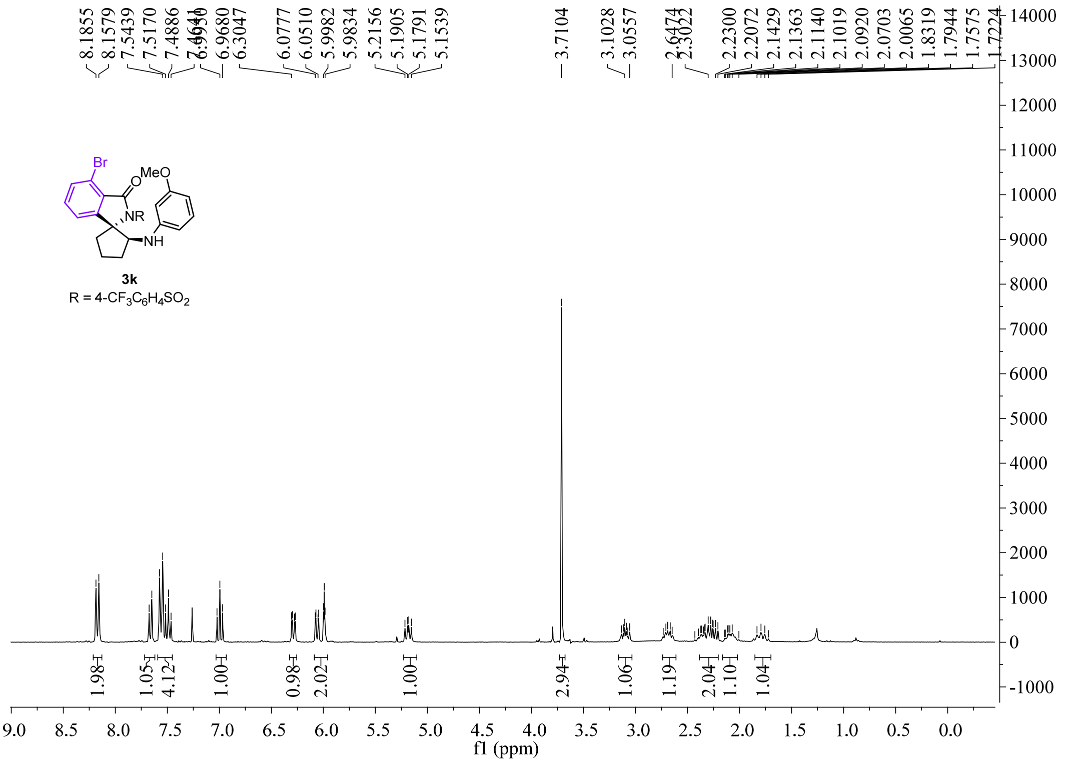
8.1855
8.1579
7.5439
7.5170
7.4886
7.4641
6.9950
6.9680
6.3047
6.0777
6.0510
5.9982
5.9834
5.2156
5.1905
5.1791
5.1539
3.7104
3.1028
3.0557
2.6474
2.3022
2.2300
2.2072
2.1429
2.1363
2.1140
2.1019
2.0920
2.0703
2.0065
1.8319
1.7944
1.7575
1.7224
Br
MeO
O
NR
NH
3k
R = 4-CF3C6H4SO2
1.98
1.05
4.12
1.00
0.98
2.02
1.00
2.94
1.06
1.19
2.04
1.10
1.04
f1 (ppm)

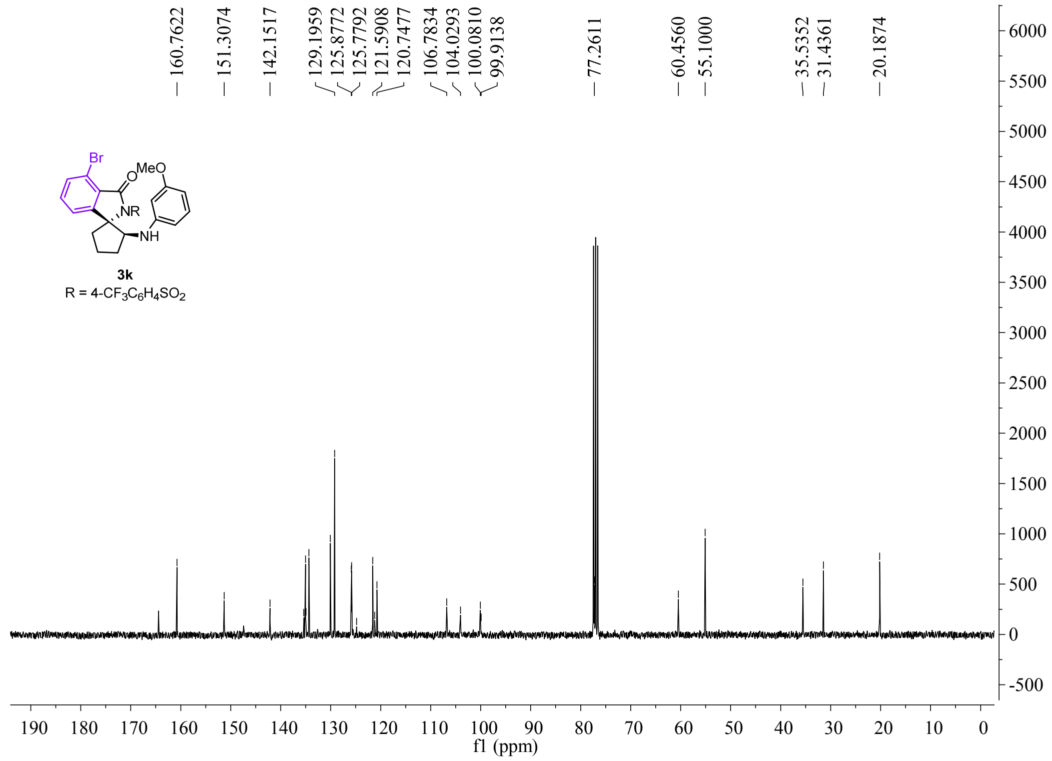
160.7622
151.3074
142.1517
129.1959
125.8772
125.7792
121.5908
120.7477
106.7834
104.0293
100.0810
99.9138
77.2611
60.4560
55.1000
35.5352
31.4361
20.1874
Br
MeO
O
NR
NH
3k
R = 4-CF3C6H4SO2
f1 (ppm)

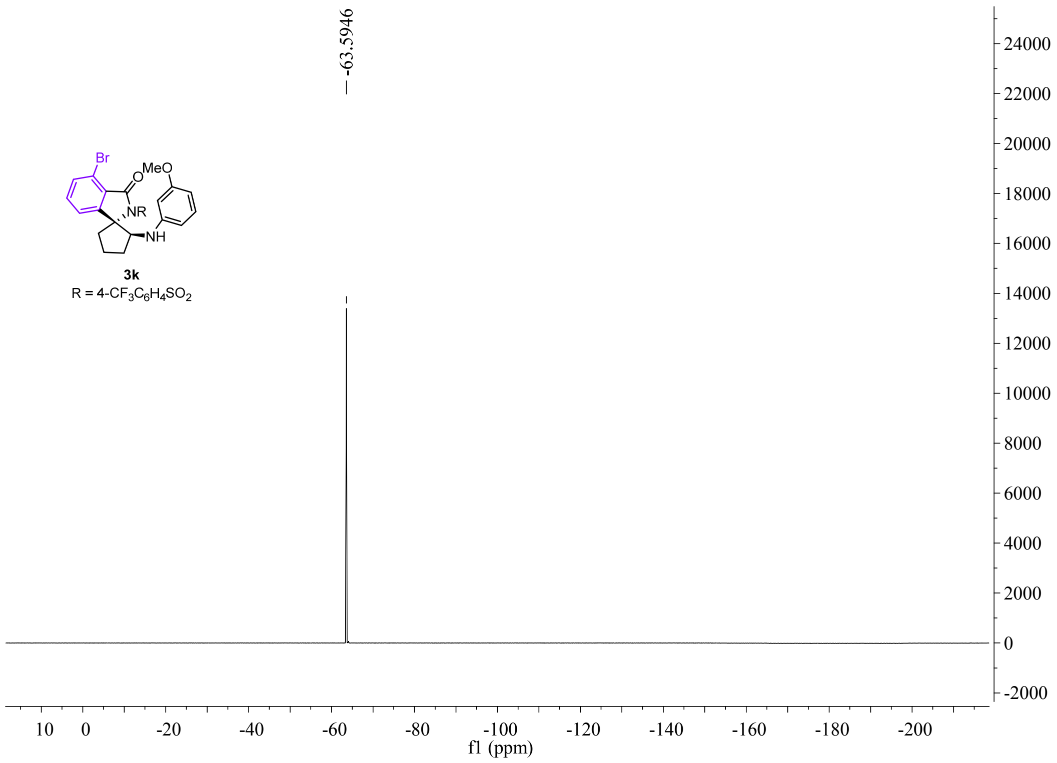
-63.5946
Br
MeO
O
NR
NH
3k
R = 4-CF$_3$C$_6$H$_4$SO$_2$
f1 (ppm)

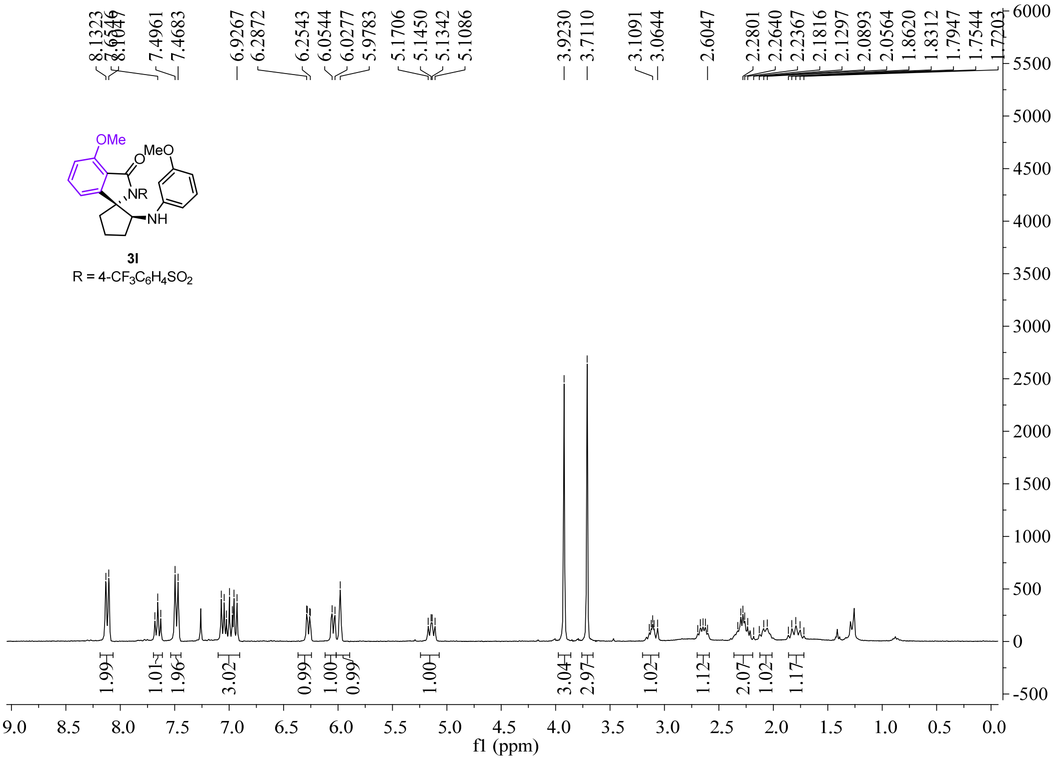
8.1323
7.6546
8.1047
7.4961
7.4683
6.9267
6.2872
6.2543
6.0544
6.0277
5.9783
5.1706
5.1450
5.1342
5.1086
3.9230
3.7110
3.1091
3.0644
2.6047
2.2801
2.2640
2.2367
2.1816
2.1297
2.0893
2.0564
1.8620
1.8312
1.7947
1.7544
1.7203
OMe
MeO
O
NR
NH
3l
R = 4-CF$_3$C$_6$H$_4$SO$_2$
1.99
1.01
1.96
3.02
0.99
1.00
0.99
1.00
3.04
2.97
1.02
1.12
2.07
1.02
1.17
f1 (ppm)

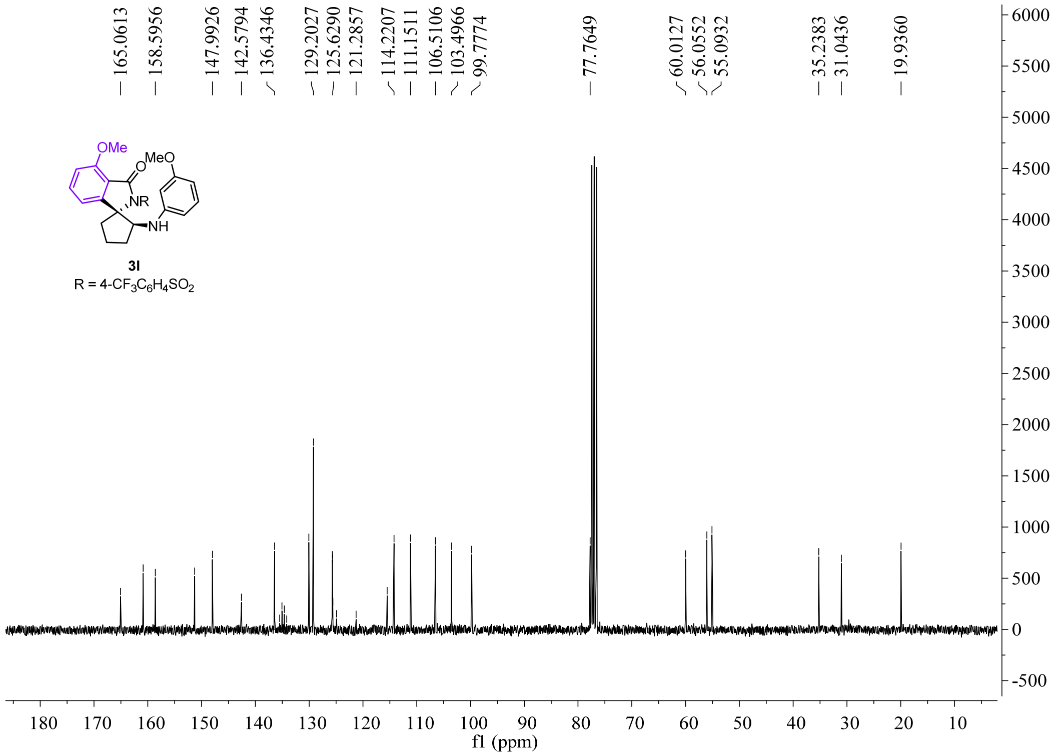
165.0613
158.5956
147.9926
142.5794
136.4346
129.2027
125.6290
121.2857
114.2207
111.1511
106.5106
103.4966
99.7774
77.7649
60.0127
56.0552
55.0932
35.2383
31.0436
19.9360
OMe
MeO
O
NR
NH
3l
R = 4-CF3C6H4SO2
f1 (ppm)

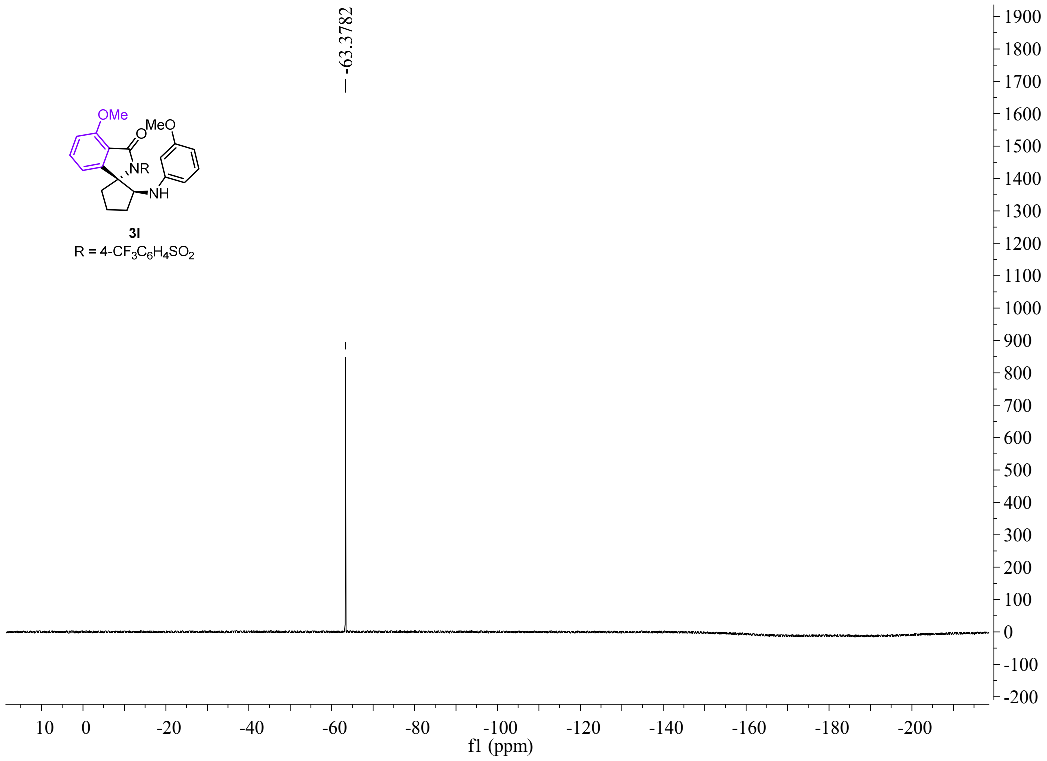
-63.3782
OMe
MeO
O
NR
NH
3l
R = 4-CF3C6H4SO2
f1 (ppm)

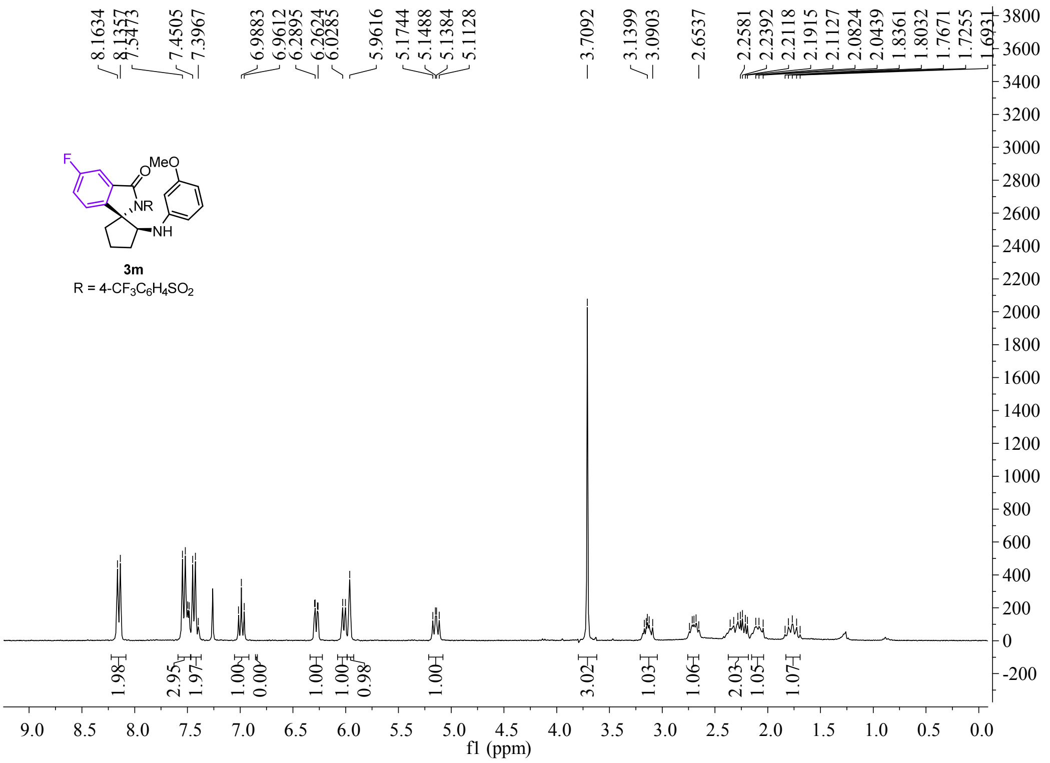

8.1634
8.1357
7.5473
7.4505
7.3967
6.9883
6.9612
6.2895
6.2624
6.0285
5.9616
5.1744
5.1488
5.1384
5.1128
3.7092
3.1399
3.0903
2.6537
2.2581
2.2392
2.2118
2.1915
2.1127
2.0824
2.0439
1.8361
1.8032
1.7671
1.7255
1.6931
3m
R = 4-CF3C6H4SO2
1.98
2.95
1.97
1.00
0.00
1.00
1.00
0.98
1.00
3.02
1.03
1.06
2.03
1.05
1.07
f1 (ppm)

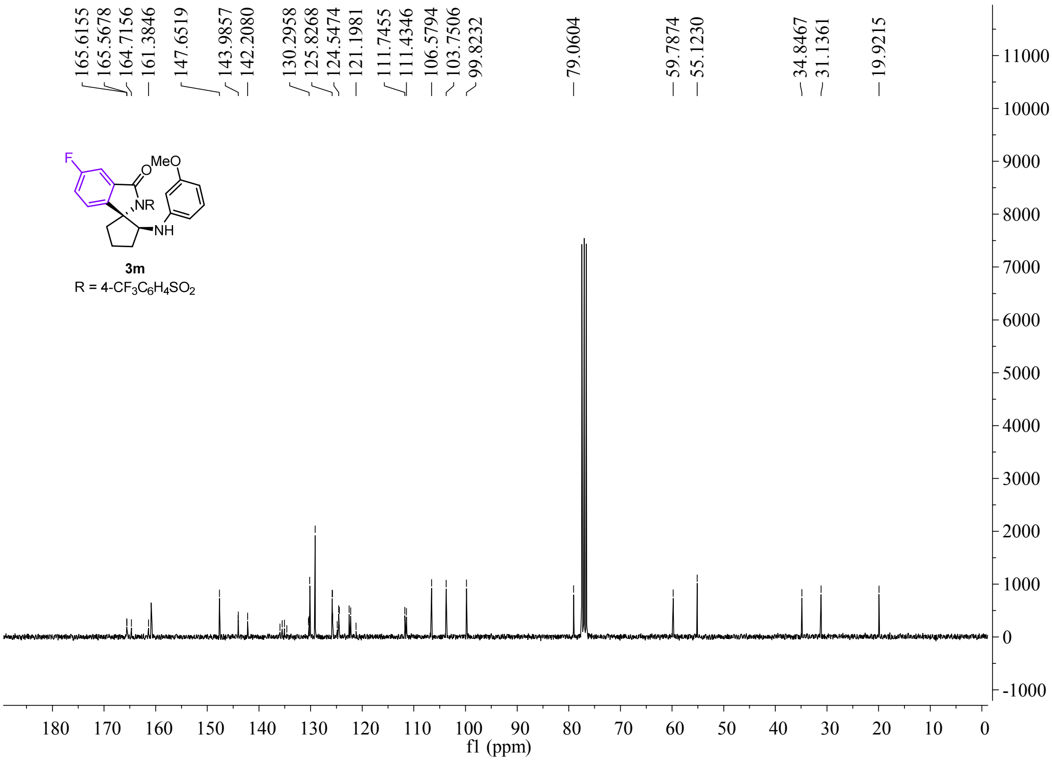

165.6155
165.5678
164.7156
161.3846
147.6519
143.9857
142.2080
130.2958
125.8268
124.5474
121.1981
111.7455
111.4346
106.5794
103.7506
99.8232
79.0604
59.7874
55.1230
34.8467
31.1361
19.9215
3m
R = 4-CF3C6H4SO2
f1 (ppm)

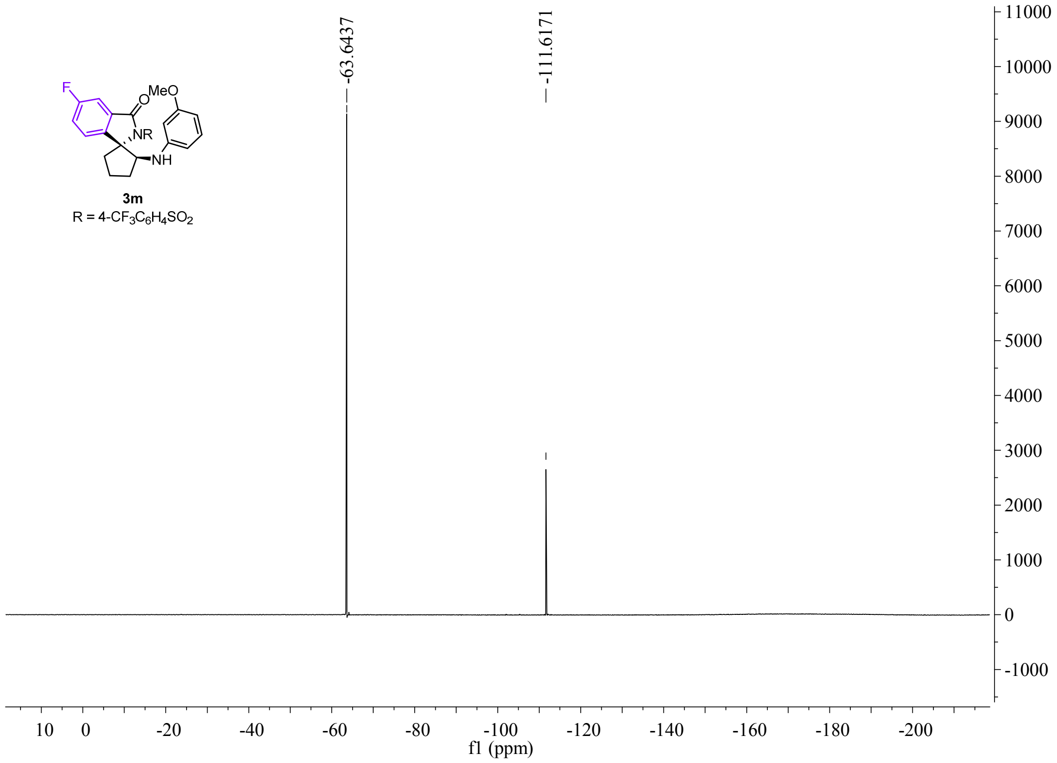
-63.6437
-111.6171
F
MeO
O
NR
NH
3m
R = 4-CF3C6H4SO2
f1 (ppm)

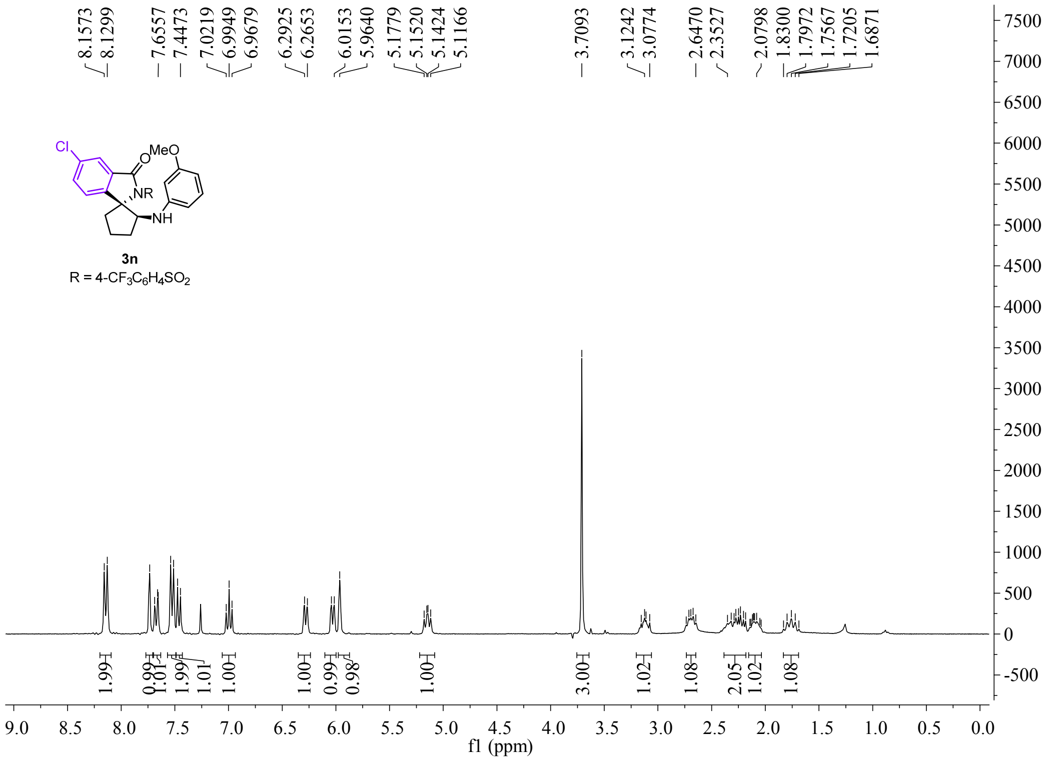
8.1573
8.1299
7.6557
7.4473
7.0219
6.9949
6.9679
6.2925
6.2653
6.0153
5.9640
5.1779
5.1520
5.1424
5.1166
3.7093
3.1242
3.0774
2.6470
2.3527
2.0798
1.8300
1.7972
1.7567
1.7205
1.6871
Cl
MeO
O
NR
NH
3n
R = 4-CF3C6H4SO2
1.99
0.99
1.01
1.99
1.01
1.00
1.00
0.99
0.98
1.00
3.00
1.02
1.08
2.05
1.02
1.08
f1 (ppm)

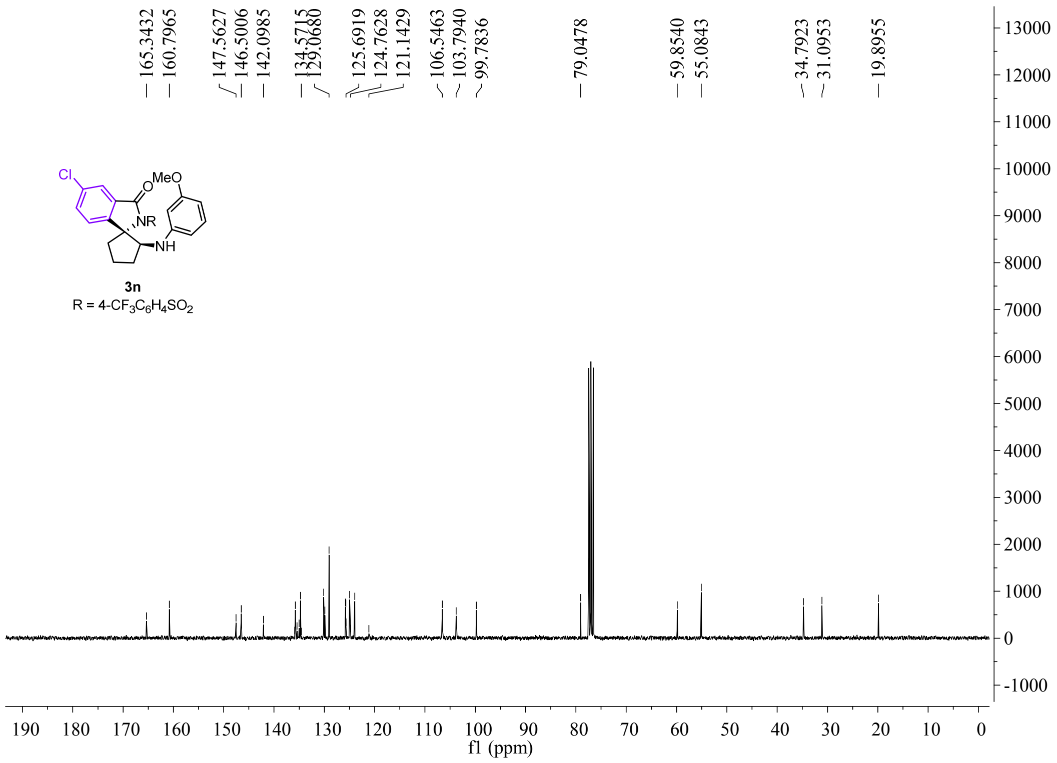

165.3432
160.7965
147.5627
146.5006
142.0985
134.5715
129.0680
125.6919
124.7628
121.1429
106.5463
103.7940
99.7836
79.0478
59.8540
55.0843
34.7923
31.0953
19.8955
Cl
MeO
O
NR
NH
3n
R = 4-CF3C6H4SO2
f1 (ppm)

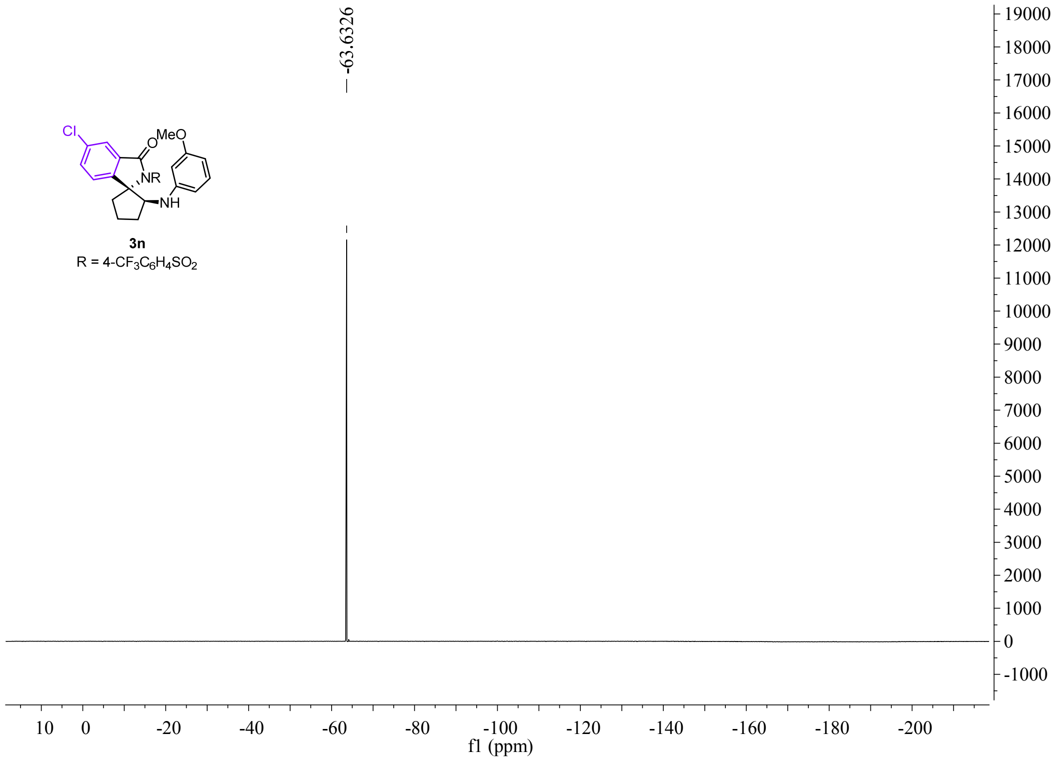

-63.6326
Cl
MeO
O
NR
NH
3n
R = 4-CF3C6H4SO2
f1 (ppm)

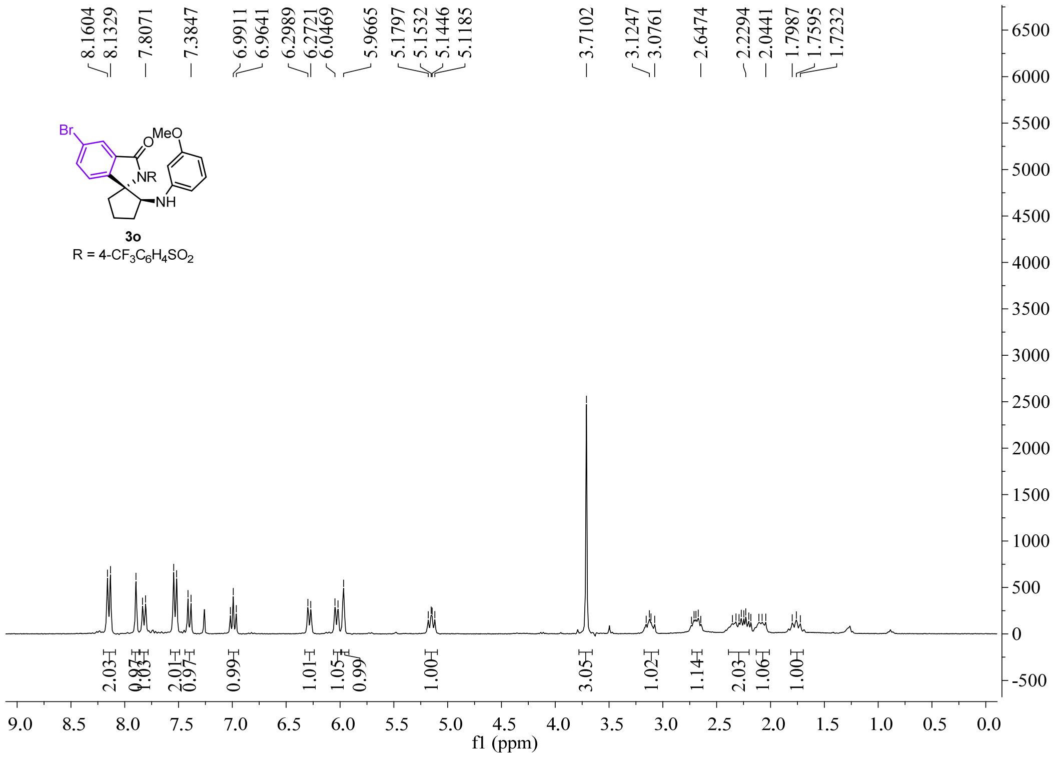
8.1604
8.1329
7.8071
7.3847
6.9911
6.9641
6.2989
6.2721
6.0469
5.9665
5.1797
5.1532
5.1446
5.1185
3.7102
3.1247
3.0761
2.6474
2.2294
2.0441
1.7987
1.7595
1.7232
Br
MeO
O
NR
NH
3o
R = 4-CF3C6H4SO2
2.03
0.97
1.03
2.01
0.97
0.99
1.01
1.05
0.99
1.00
3.05
1.02
1.14
2.03
1.06
1.00
f1 (ppm)

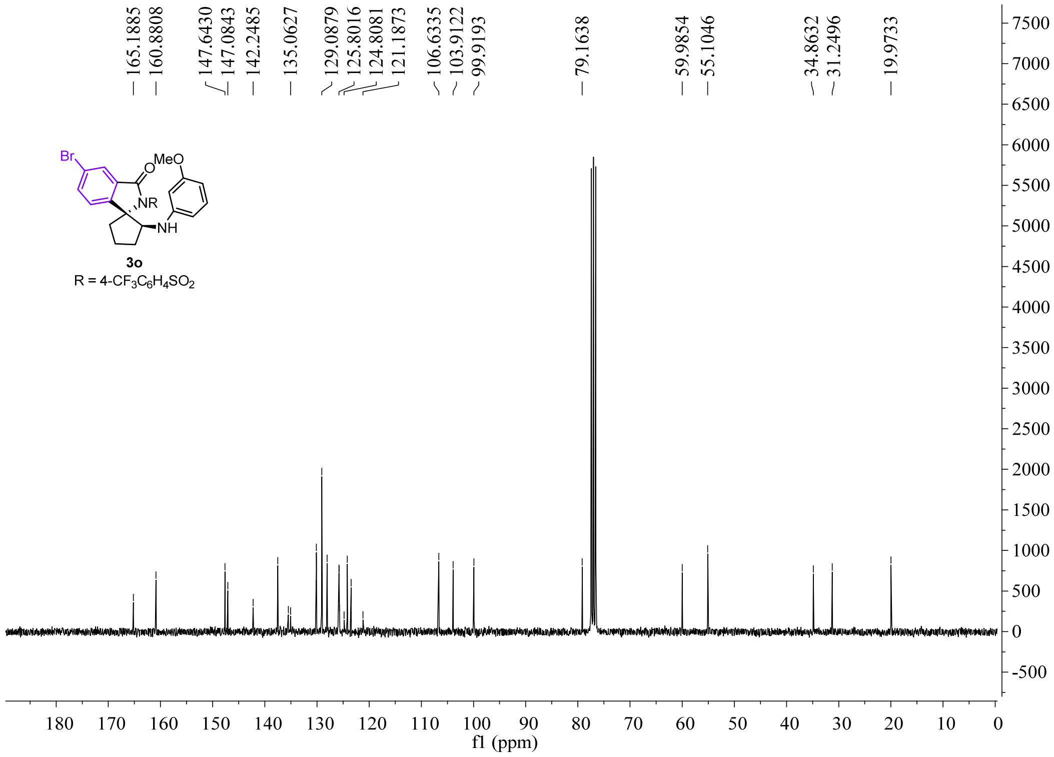
165.1885
160.8808
147.6430
147.0843
142.2485
135.0627
129.0879
125.8016
124.8081
121.1873
106.6335
103.9122
99.9193
79.1638
59.9854
55.1046
34.8632
31.2496
19.9733
Br
MeO
O
NR
NH
3o
R = 4-CF3C6H4SO2
f1 (ppm)

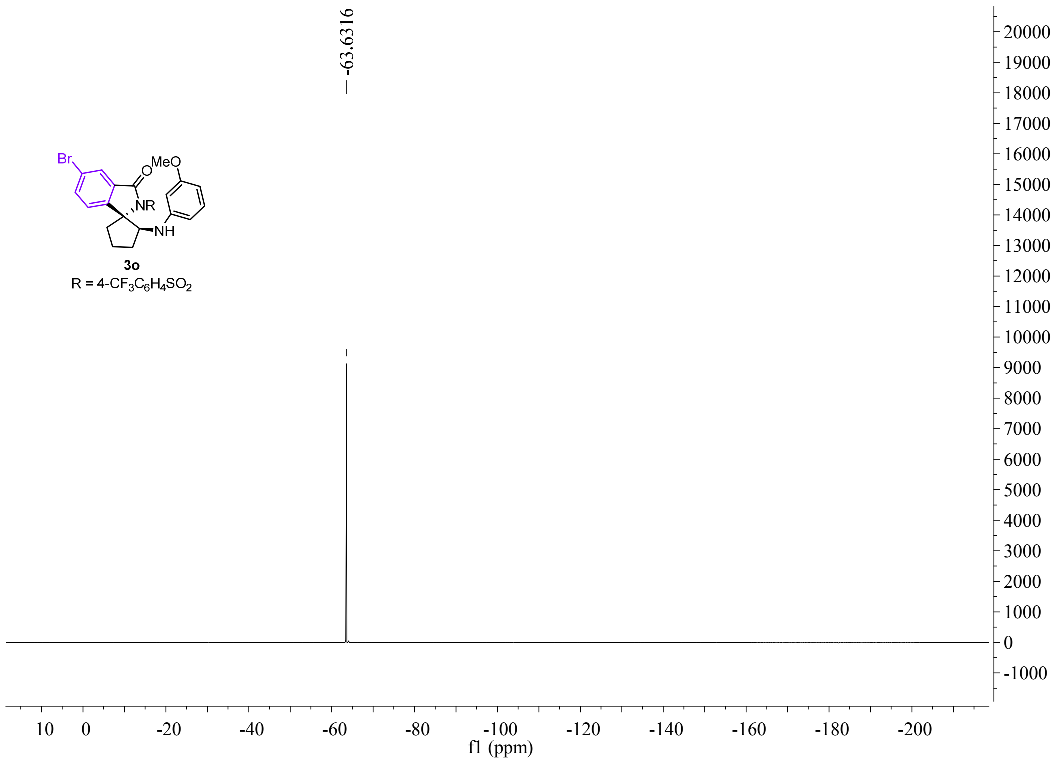

-63.6316
Br
MeO
O
NR
NH
3o
R = 4-CF3C6H4SO2
f1 (ppm)

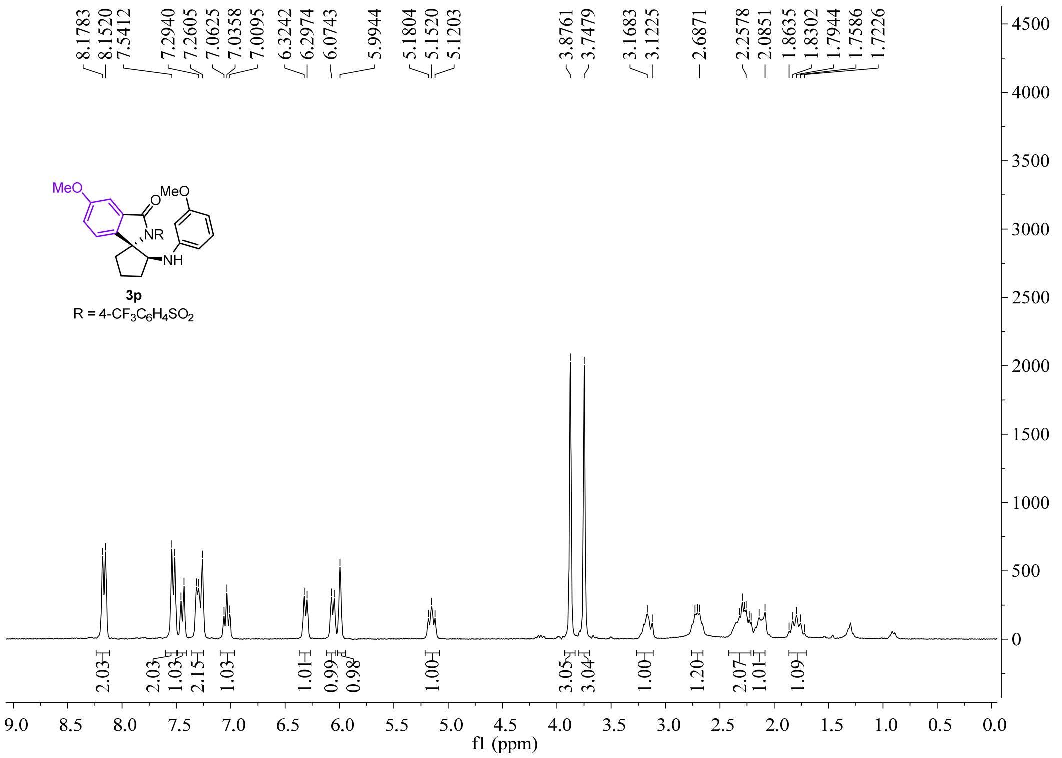

8.1783
8.1520
7.5412
7.2940
7.2605
7.0625
7.0358
7.0095
6.3242
6.2974
6.0743
5.9944
5.1804
5.1520
5.1203
3.8761
3.7479
3.1683
3.1225
2.6871
2.2578
2.0851
1.8635
1.8302
1.7944
1.7586
1.7226
MeO
MeO
O
NR
NH
3p
R = 4-CF3C6H4SO2
f1 (ppm)

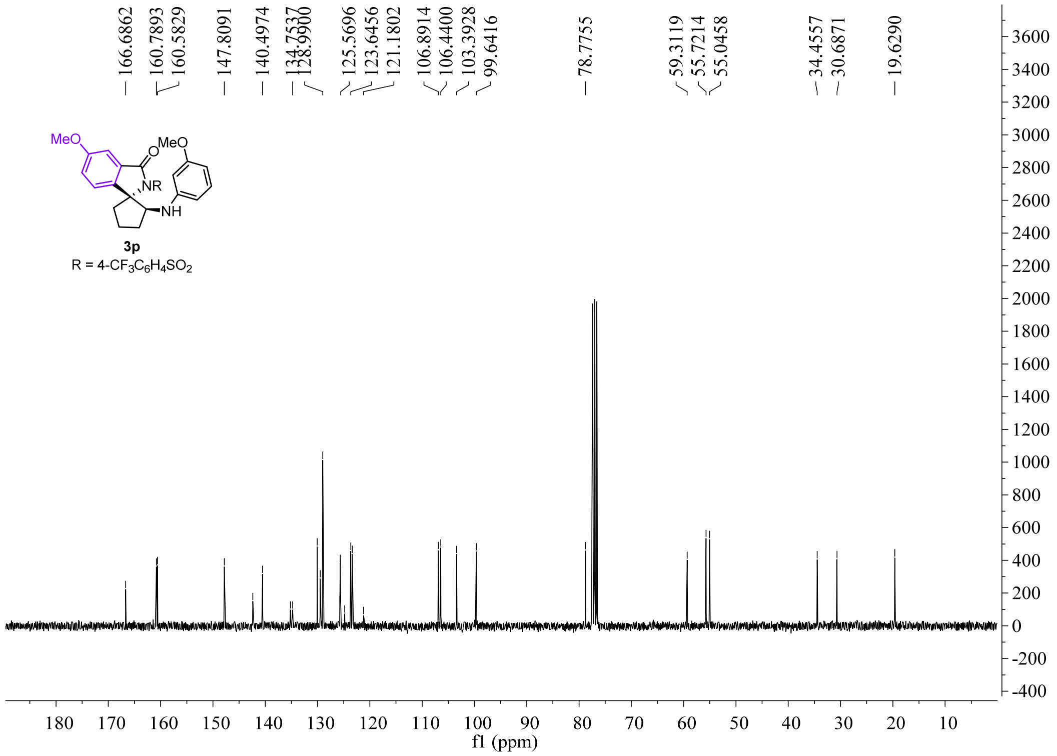

166.6862
160.7893
160.5829
147.8091
140.4974
128.9900
125.5696
123.6456
121.1802
106.8914
106.4400
103.3928
99.6416
78.7755
59.3119
55.7214
55.0458
34.4557
30.6871
19.6290
MeO
MeO
O
NR
NH
3p
R = 4-CF3C6H4SO2
f1 (ppm)

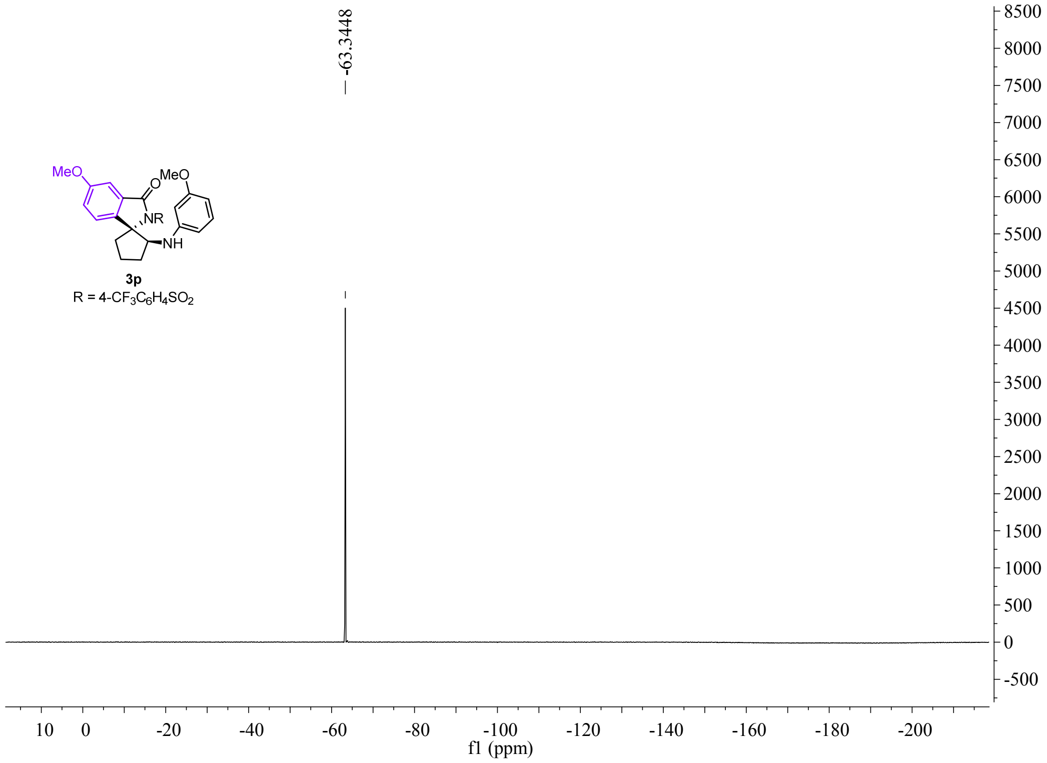

-63.3448
MeO
MeO
O
NR
NH
3p
R = 4-CF3C6H4SO2
f1 (ppm)

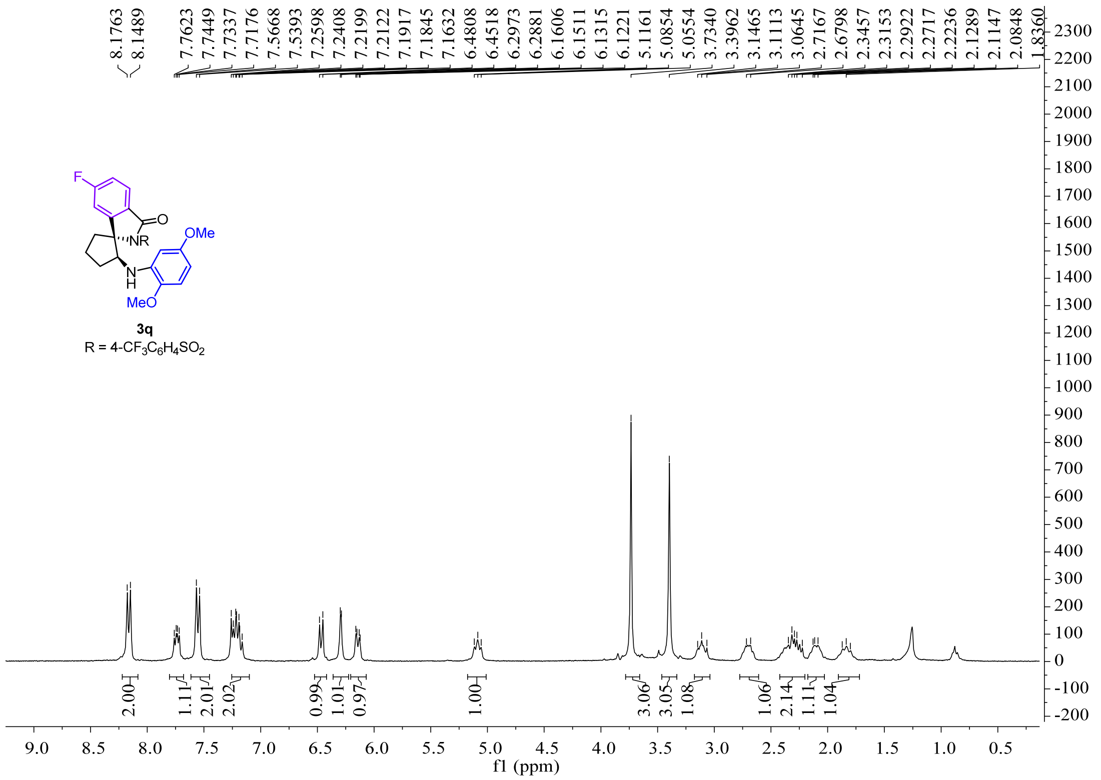
8.1763
8.1489
7.7623
7.7449
7.7337
7.7176
7.5668
7.5393
7.2598
7.2408
7.2199
7.2122
7.1917
7.1845
7.1632
6.4808
6.4518
6.2973
6.2881
6.1606
6.1511
6.1315
6.1221
5.1161
5.0854
5.0554
3.7340
3.3962
3.1465
3.1113
3.0645
2.7167
2.6798
2.3457
2.3153
2.2922
2.2717
2.2236
2.1289
2.1147
2.0848
1.8360
F
O
NR
OMe
N
H
MeO
3q
R = 4-CF3C6H4SO2
2.00
1.11
2.01
2.02
0.99
1.01
0.97
1.00
3.06
3.05
1.08
1.06
2.14
1.11
1.04
f1 (ppm)

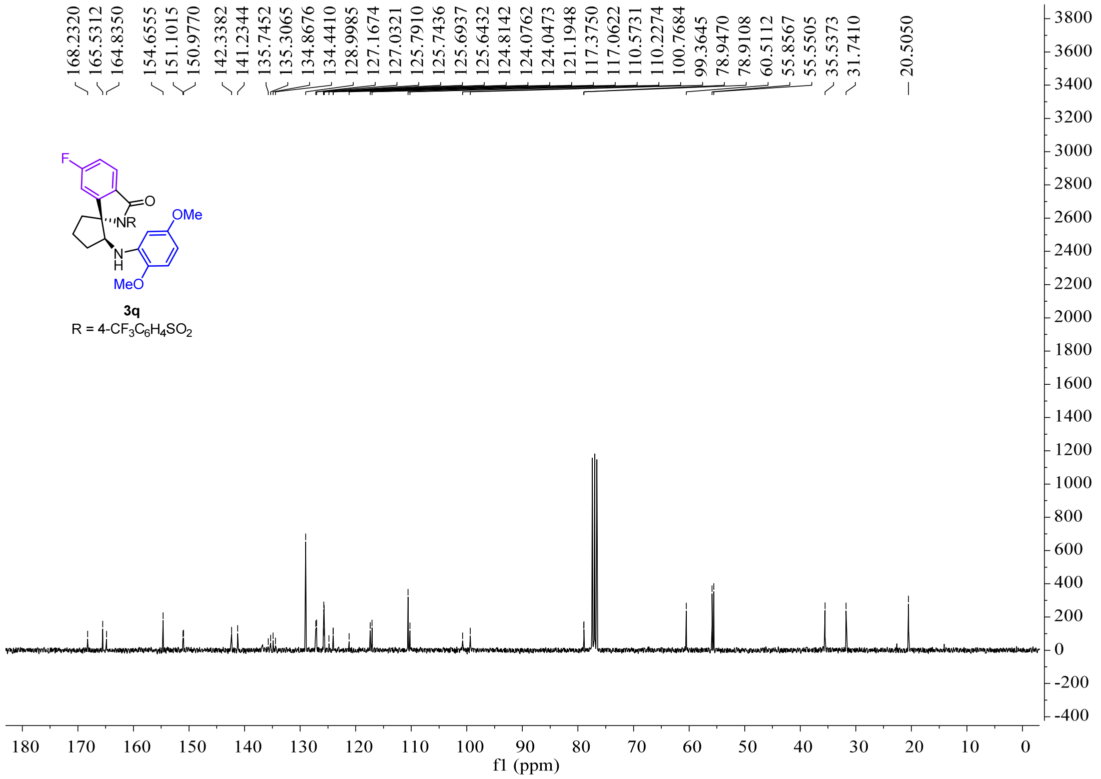
168.2320
165.5312
164.8350
154.6555
151.1015
150.9770
142.3382
141.2344
135.7452
135.3065
134.8676
134.4410
128.9985
127.1674
127.0321
125.7910
125.7436
125.6937
125.6432
124.8142
124.0762
124.0473
121.1948
117.3750
117.0622
110.5731
110.2274
100.7684
99.3645
78.9470
78.9108
60.5112
55.8567
55.5505
35.5373
31.7410
20.5050
F
O
NR
OMe
N
H
MeO
3q
R = 4-CF3C6H4SO2
f1 (ppm)

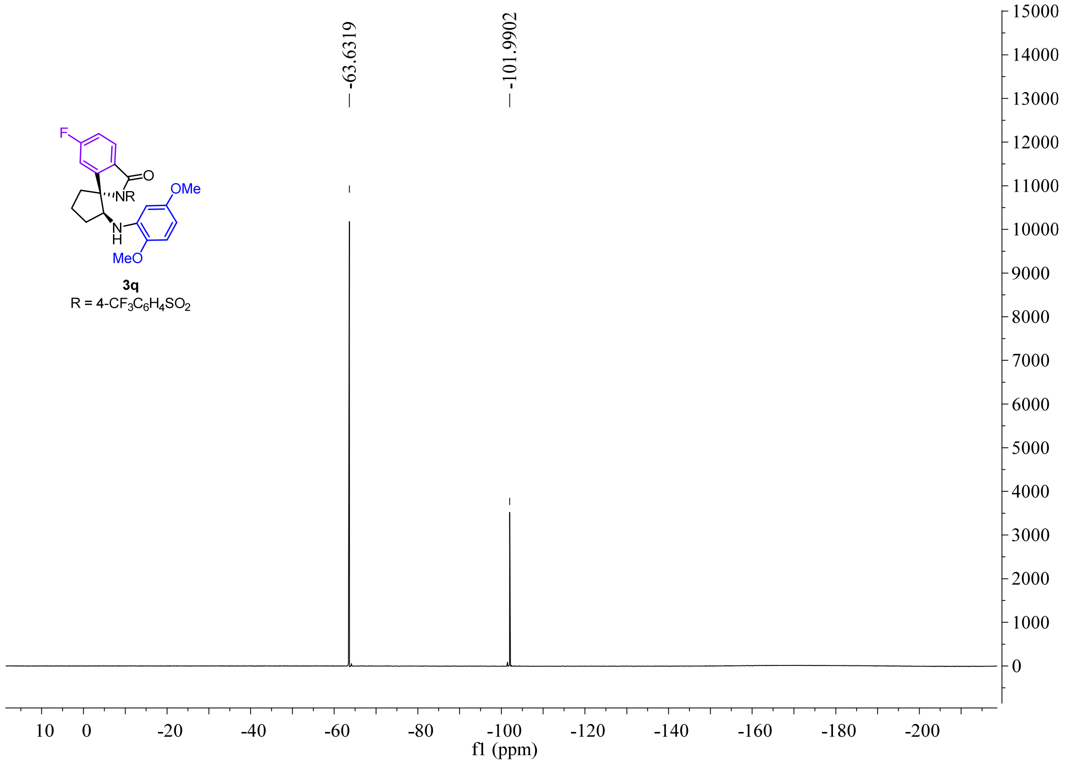
-63.6319
-101.9902
F
O
NR
OMe
H
N
MeO
3q
R = 4-CF$_3$C$_6$H$_4$SO$_2$
f1 (ppm)

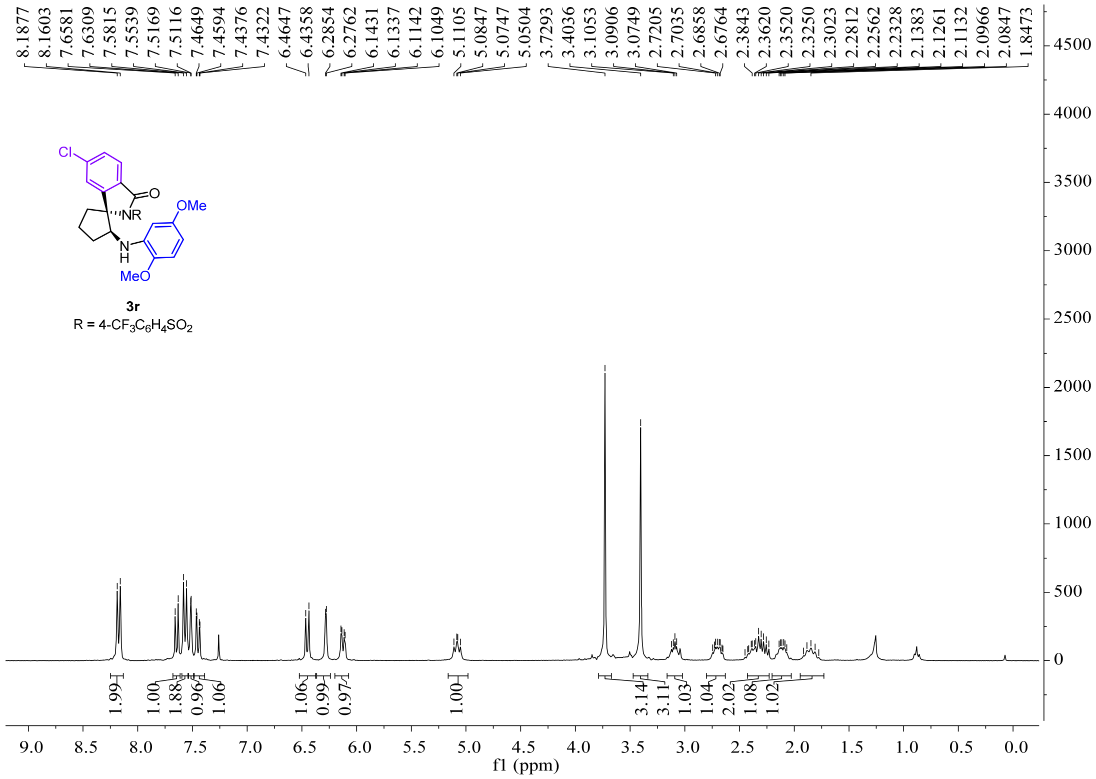
8.1877
8.1603
7.6581
7.6309
7.5815
7.5539
7.5169
7.5116
7.4649
7.4594
7.4376
7.4322
6.4647
6.4358
6.2854
6.2762
6.1431
6.1337
6.1142
6.1049
5.1105
5.0847
5.0747
5.0504
3.7293
3.4036
3.1053
3.0906
3.0749
2.7205
2.7035
2.6858
2.6764
2.3843
2.3620
2.3520
2.3250
2.3023
2.2812
2.2562
2.2328
2.1383
2.1261
2.1132
2.0966
2.0847
1.8473
Cl
O
NR
OMe
H
N
MeO
3r
R = 4-CF$_3$C$_6$H$_4$SO$_2$
1.99
1.00
1.88
0.96
1.06
1.06
0.99
0.97
1.00
3.14
3.11
1.03
1.04
2.02
1.08
1.02
f1 (ppm)

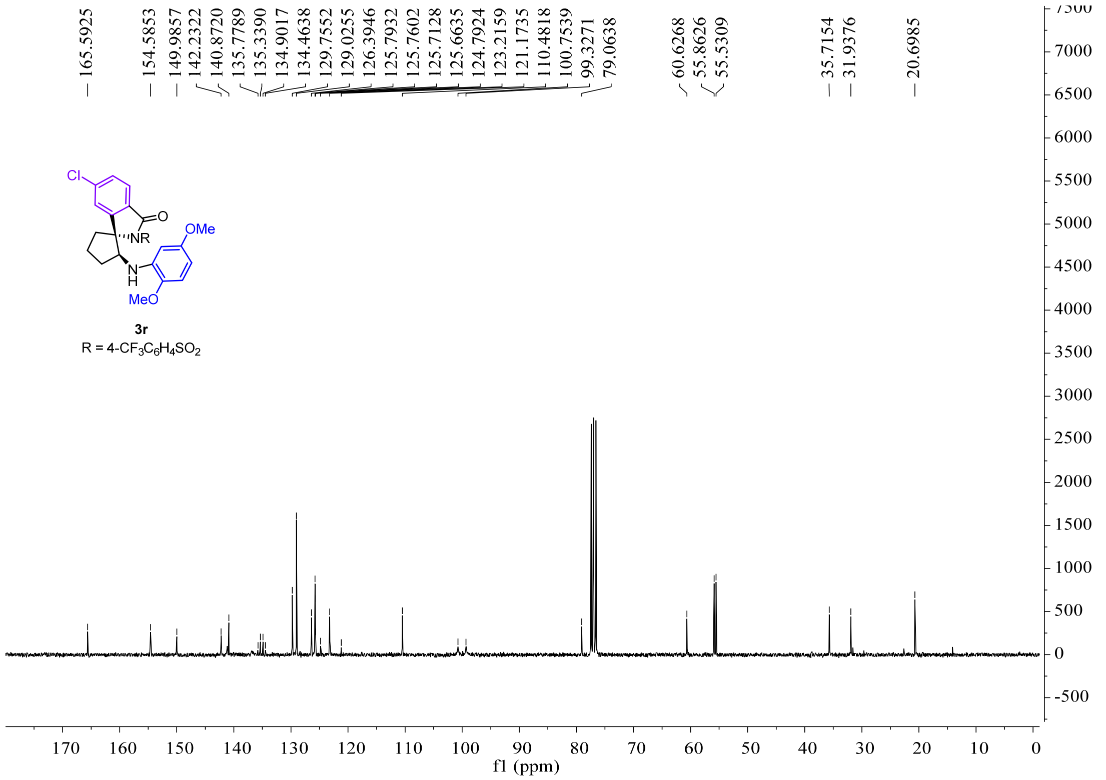
165.5925
154.5853
149.9857
142.2322
140.8720
135.7789
135.3390
134.9017
134.4638
129.7552
129.0255
126.3946
125.7932
125.7602
125.7128
125.6635
124.7924
123.2159
121.1735
110.4818
100.7539
99.3271
79.0638
60.6268
55.8626
55.5309
35.7154
31.9376
20.6985
Cl
O
NR
OMe
N
H
MeO
3r
R = 4-CF3C6H4SO2
f1 (ppm)

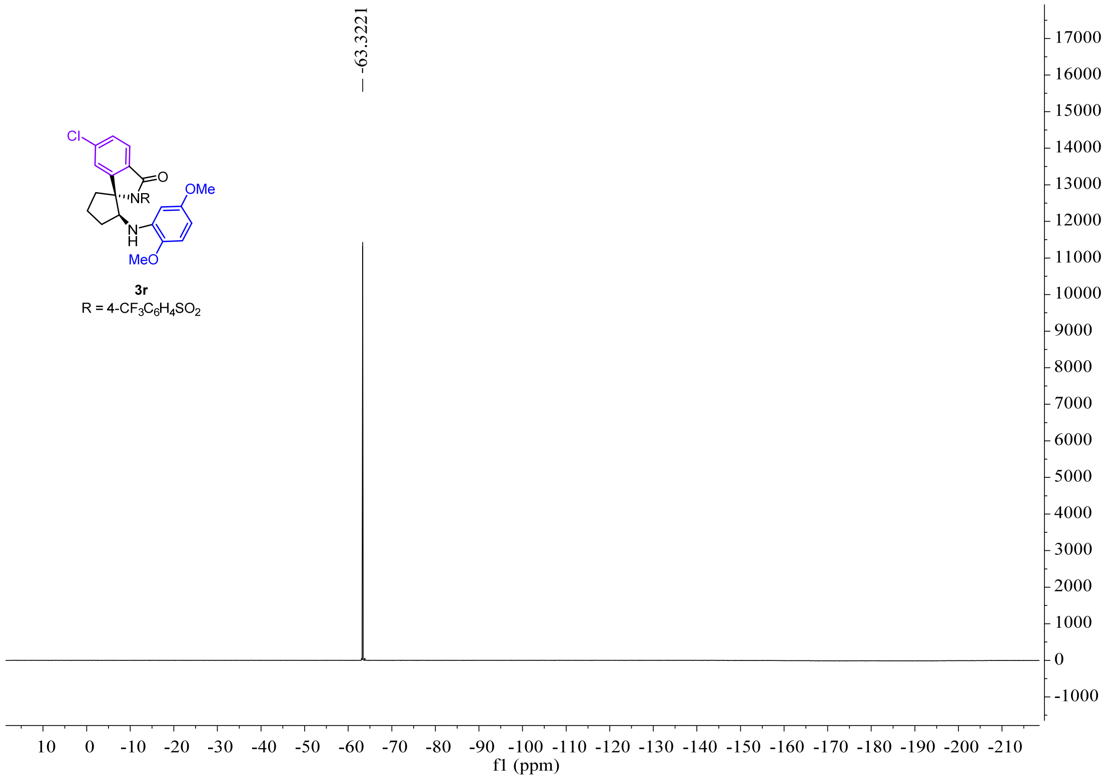
-63.3221
Cl
O
NR
OMe
N
H
MeO
3r
R = 4-CF3C6H4SO2
f1 (ppm)

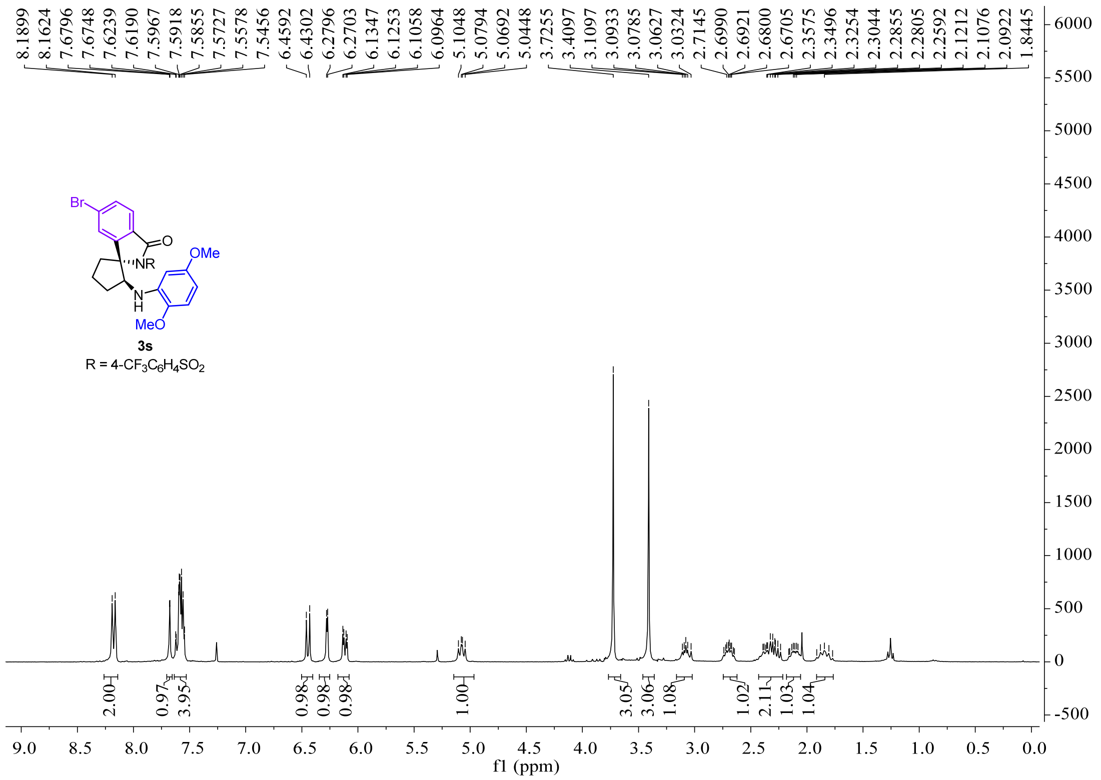
8.1899
8.1624
7.6796
7.6748
7.6239
7.6190
7.5967
7.5918
7.5855
7.5727
7.5578
7.5456
6.4592
6.4302
6.2796
6.2703
6.1347
6.1253
6.1058
6.0964
5.1048
5.0794
5.0692
5.0448
3.7255
3.4097
3.1097
3.0933
3.0785
3.0627
3.0324
2.7145
2.6990
2.6921
2.6800
2.6705
2.3575
2.3496
2.3254
2.3044
2.2855
2.2805
2.2592
2.1212
2.1076
2.0922
1.8445
Br
O
NR
OMe
N
H
MeO
3s
R = 4-CF3C6H4SO2
f1 (ppm)

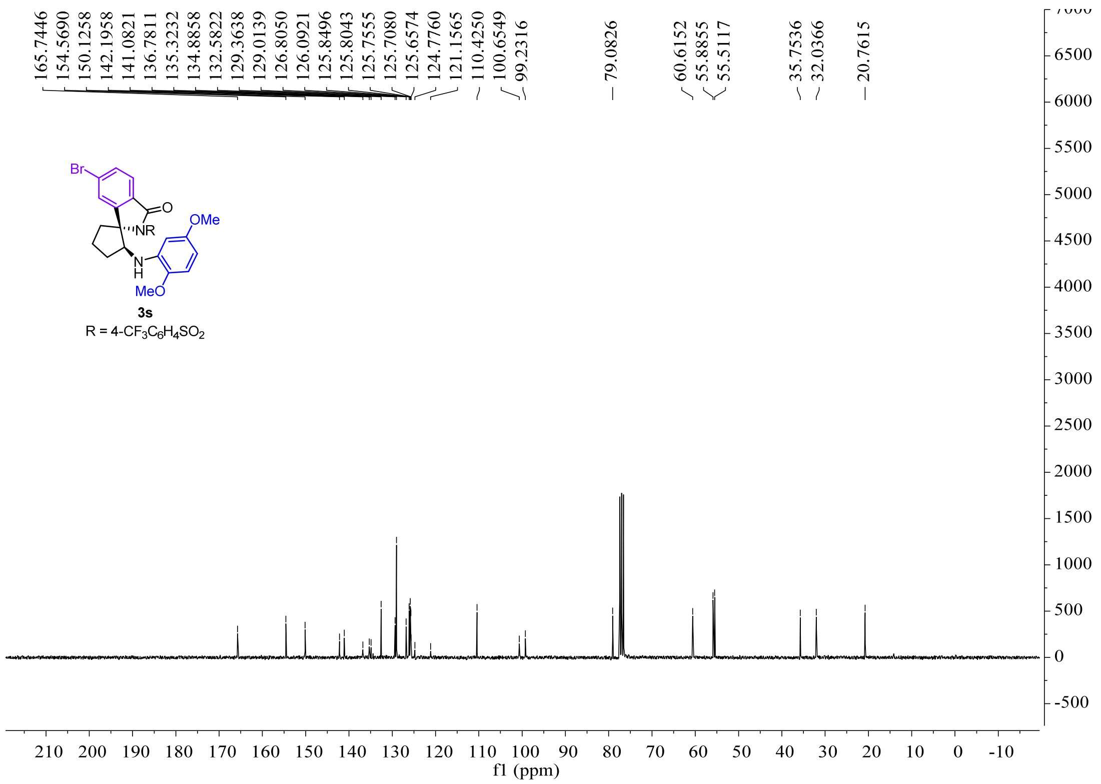
165.7446
154.5690
150.1258
142.1958
141.0821
136.7811
135.3232
134.8858
132.5822
129.3638
129.0139
126.8050
126.0921
125.8496
125.8043
125.7555
125.7080
125.6574
124.7760
121.1565
110.4250
100.6549
99.2316
79.0826
60.6152
55.8855
55.5117
35.7536
32.0366
20.7615
Br
O
NR
OMe
N
H
MeO
3s
R = 4-CF3C6H4SO2
f1 (ppm)

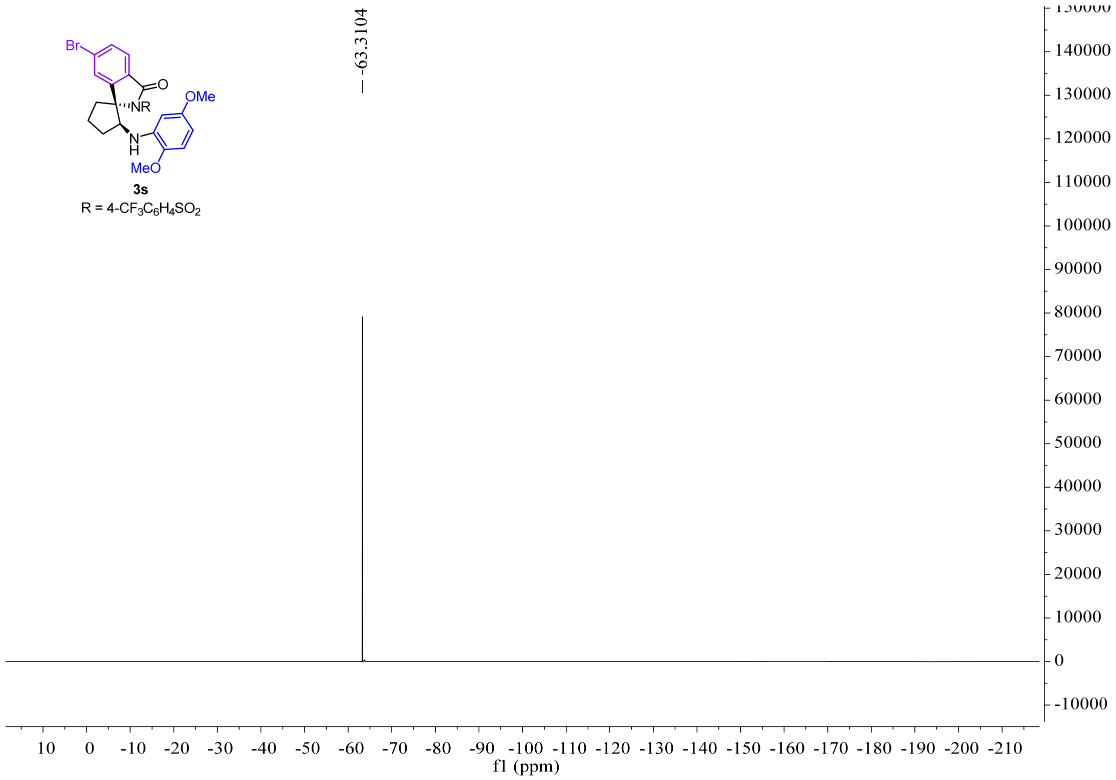
-63.3104
Br
O
NR
OMe
N
H
MeO
3s
R = 4-CF3C6H4SO2
f1 (ppm)

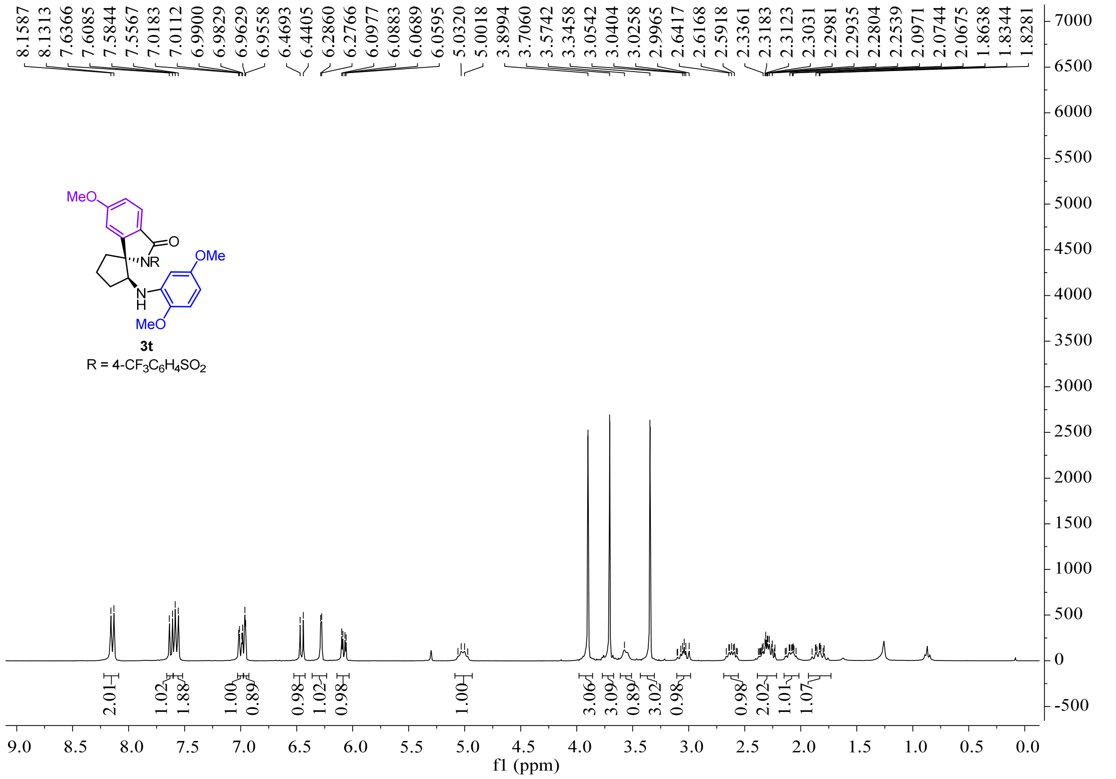
8.1587
8.1313
7.6366
7.6085
7.5844
7.5567
7.0183
7.0112
6.9900
6.9829
6.9629
6.9558
6.4693
6.4405
6.2860
6.2766
6.0977
6.0883
6.0689
6.0595
5.0320
5.0018
3.8994
3.7060
3.5742
3.3458
3.0542
3.0404
3.0258
2.9965
2.6417
2.6168
2.5918
2.3361
2.3183
2.3123
2.3031
2.2981
2.2935
2.2804
2.2539
2.0971
2.0744
2.0675
1.8638
1.8344
1.8281
MeO
O
NR
OMe
N
H
MeO
3t
R = 4-CF3C6H4SO2
2.01
1.02
1.88
1.00
0.89
0.98
1.02
0.98
1.00
3.06
3.09
0.89
3.02
0.98
0.98
2.02
1.01
1.07
f1 (ppm)

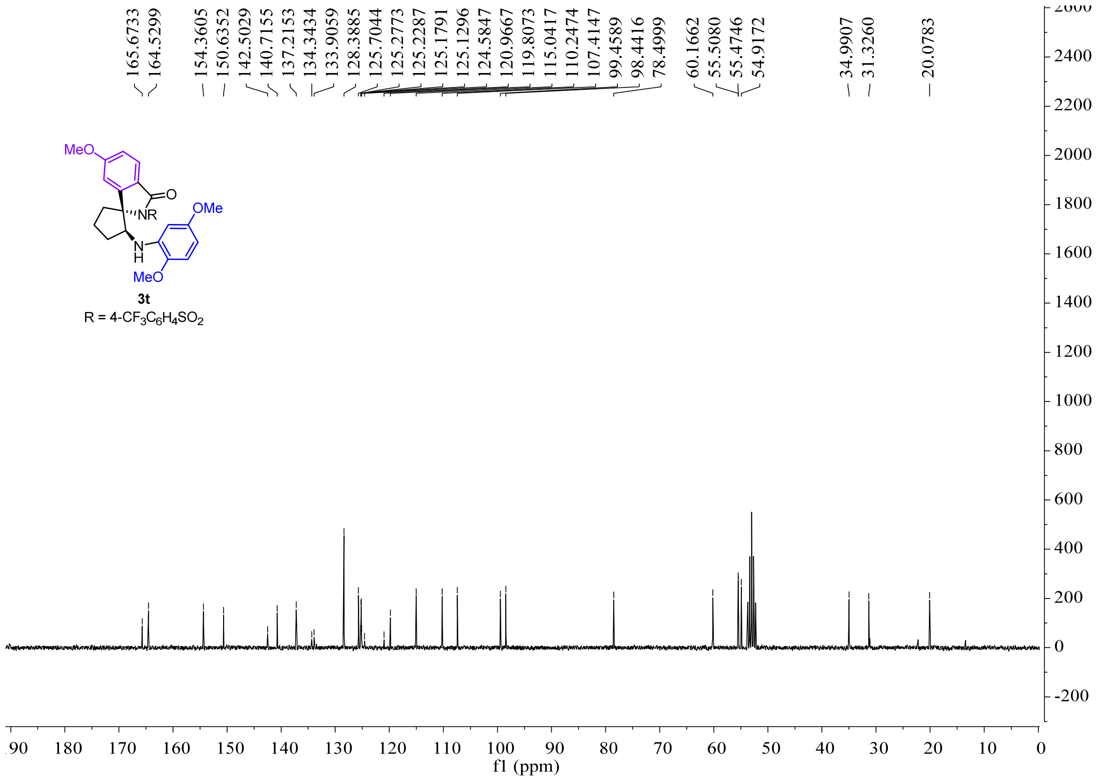
165.6733
164.5299
154.3605
150.6352
142.5029
140.7155
137.2153
134.3434
133.9059
128.3885
125.7044
125.2773
125.2287
125.1791
125.1296
124.5847
120.9667
119.8073
115.0417
110.2474
107.4147
99.4589
98.4416
78.4999
60.1662
55.5080
55.4746
54.9172
34.9907
31.3260
20.0783
MeO
O
NR
OMe
N
H
MeO
3t
R = 4-CF3C6H4SO2
f1 (ppm)

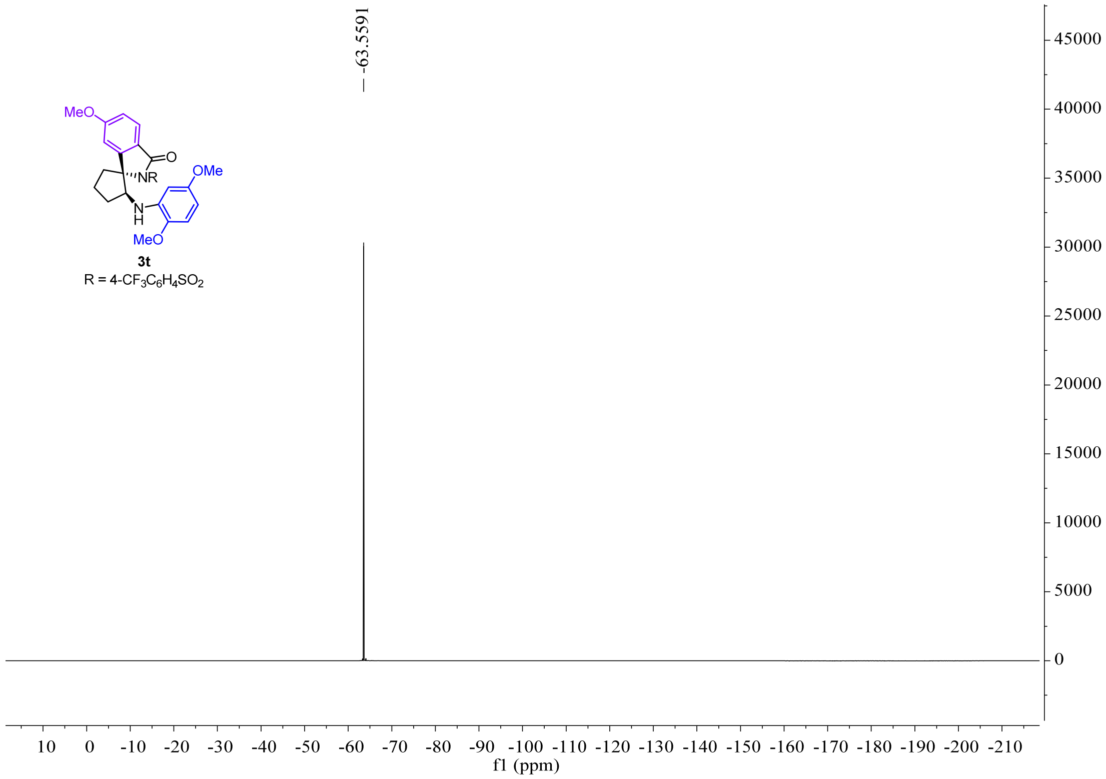
-63.5591
MeO
O
NR
OMe
N
H
MeO
3t
R = 4-CF3C6H4SO2
f1 (ppm)

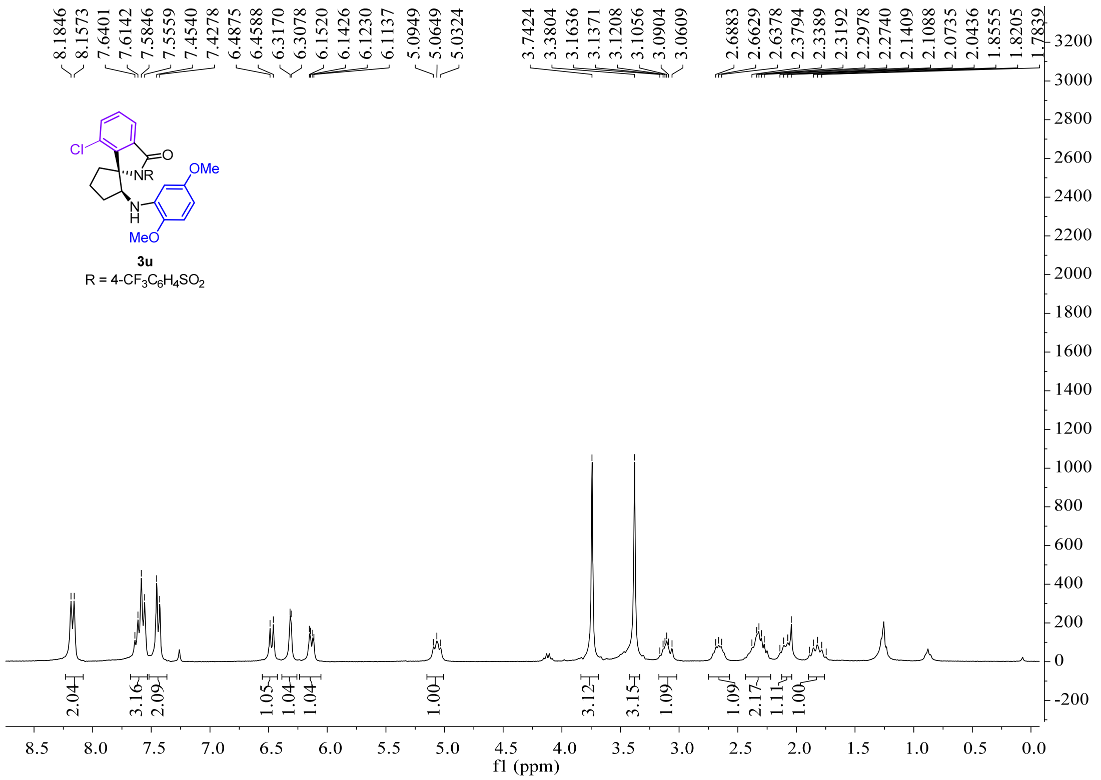
8.1846
8.1573
7.6401
7.6142
7.5846
7.5559
7.4540
7.4278
6.4875
6.4588
6.3170
6.3078
6.1520
6.1426
6.1230
6.1137
5.0949
5.0649
5.0324
3.7424
3.3804
3.1636
3.1371
3.1208
3.1056
3.0904
3.0609
2.6883
2.6629
2.6378
2.3794
2.3389
2.3192
2.2978
2.2740
2.1409
2.1088
2.0735
2.0436
1.8555
1.8205
1.7839
Cl
O
NR
OMe
N
H
MeO
3u
R = 4-CF3C6H4SO2
2.04
3.16
2.09
1.05
1.04
1.04
1.00
3.12
3.15
1.09
1.09
2.17
1.11
1.00
f1 (ppm)

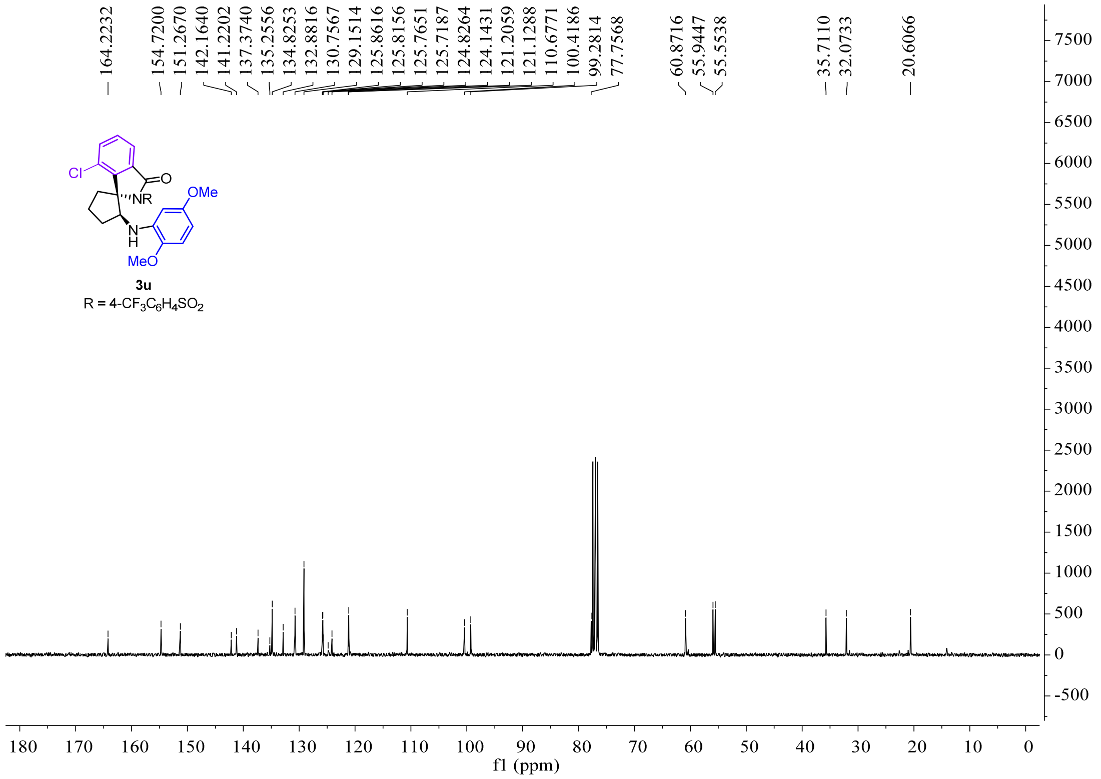
164.2232
154.7200
151.2670
142.1640
141.2202
137.3740
135.2556
134.8253
132.8816
130.7567
129.1514
125.8616
125.8156
125.7651
125.7187
124.8264
124.1431
121.2059
121.1288
110.6771
100.4186
99.2814
77.7568
60.8716
55.9447
55.5538
35.7110
32.0733
20.6066
Cl
O
NR
OMe
N
H
MeO
3u
R = 4-CF3C6H4SO2
f1 (ppm)

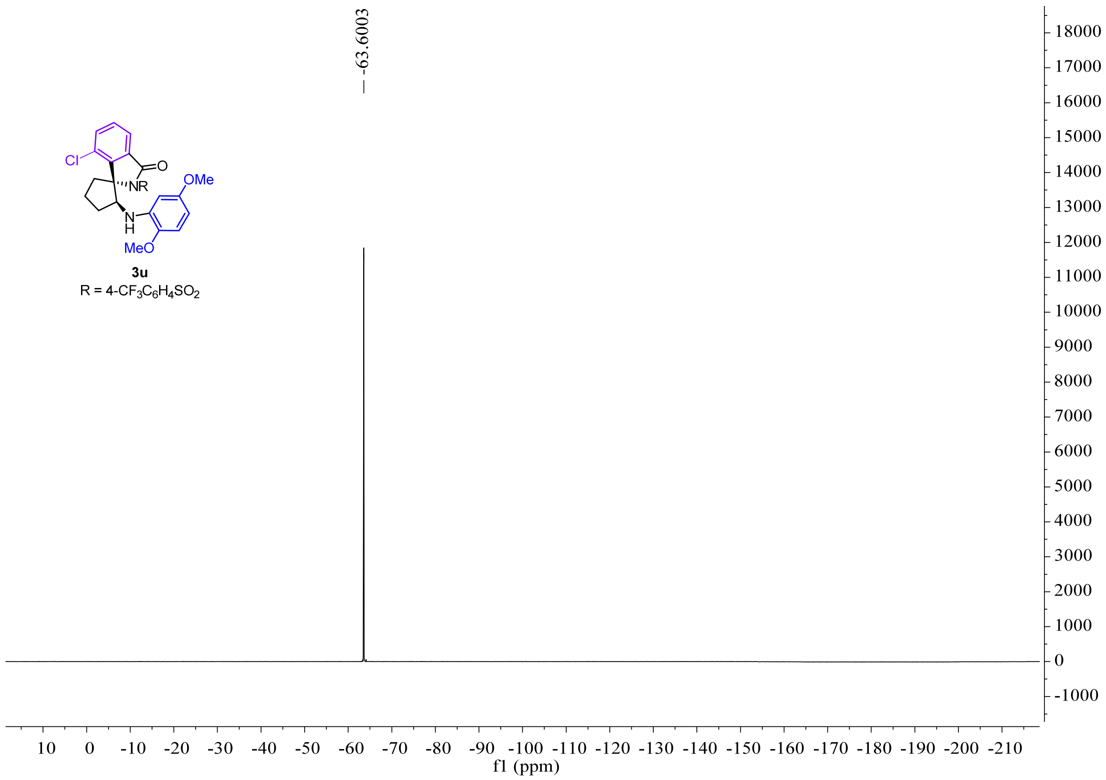
-63.6003
3u
R = 4-CF3C6H4SO2
OMe
MeO
Cl
NR
f1 (ppm)

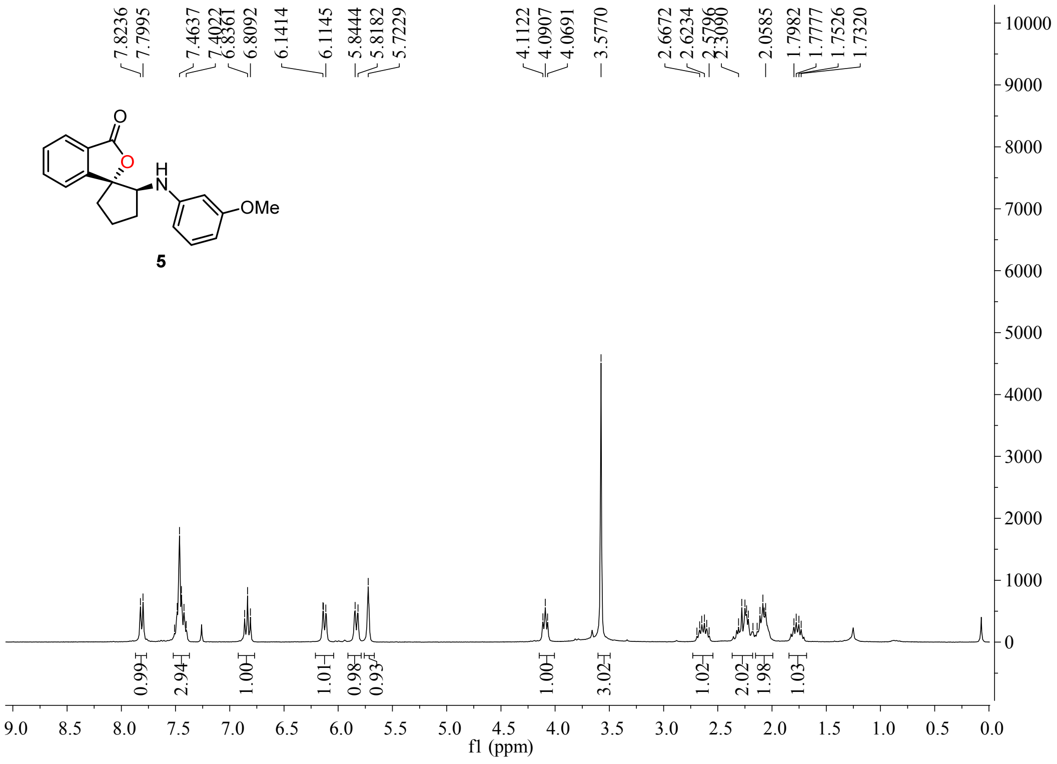
7.8236
7.7995
7.4637
7.4022
6.8361
6.8092
6.1414
6.1145
5.8444
5.8182
5.7229
4.1122
4.0907
4.0691
3.5770
2.6672
2.6234
2.5796
2.3090
2.0585
1.7982
1.7777
1.7526
1.7320
0.99
2.94
1.00
1.01
0.98
0.93
1.00
3.02
1.02
2.02
1.98
1.03
5
OMe
f1 (ppm)

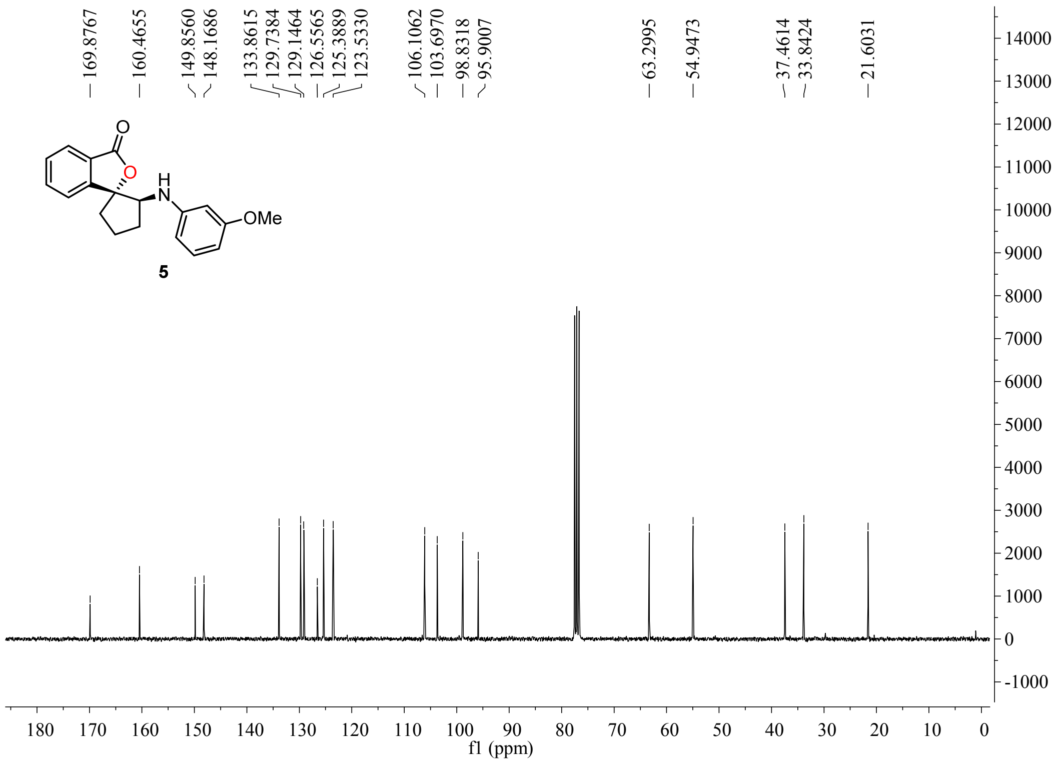

169.8767
160.4655
149.8560
148.1686
133.8615
129.7384
129.1464
126.5565
125.3889
123.5330
106.1062
103.6970
98.8318
95.9007
63.2995
54.9473
37.4614
33.8424
21.6031
OMe
5
f1 (ppm)

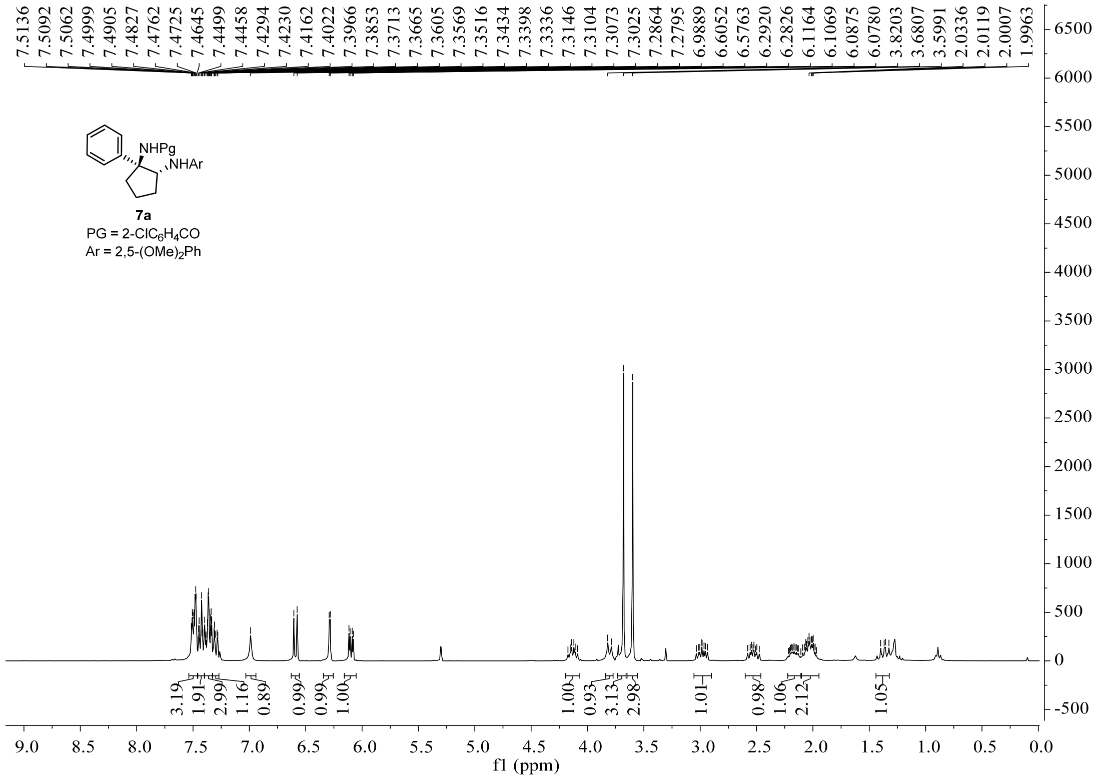

7.5136
7.5092
7.5062
7.4999
7.4905
7.4827
7.4762
7.4725
7.4645
7.4499
7.4458
7.4294
7.4230
7.4162
7.4022
7.3966
7.3853
7.3713
7.3665
7.3605
7.3569
7.3516
7.3434
7.3398
7.3336
7.3146
7.3104
7.3073
7.3025
7.2864
7.2795
6.9889
6.6052
6.5763
6.2920
6.2826
6.1164
6.1069
6.0875
6.0780
3.8203
3.6807
3.5991
2.0336
2.0119
2.0007
1.9963
NHPg
NHAr
7a
PG = 2-ClC6H4CO
Ar = 2,5-(OMe)2Ph
3.19
1.91
2.99
1.16
0.89
0.99
0.99
1.00
1.00
0.93
3.13
2.98
1.01
0.98
1.06
2.12
1.05
f1 (ppm)

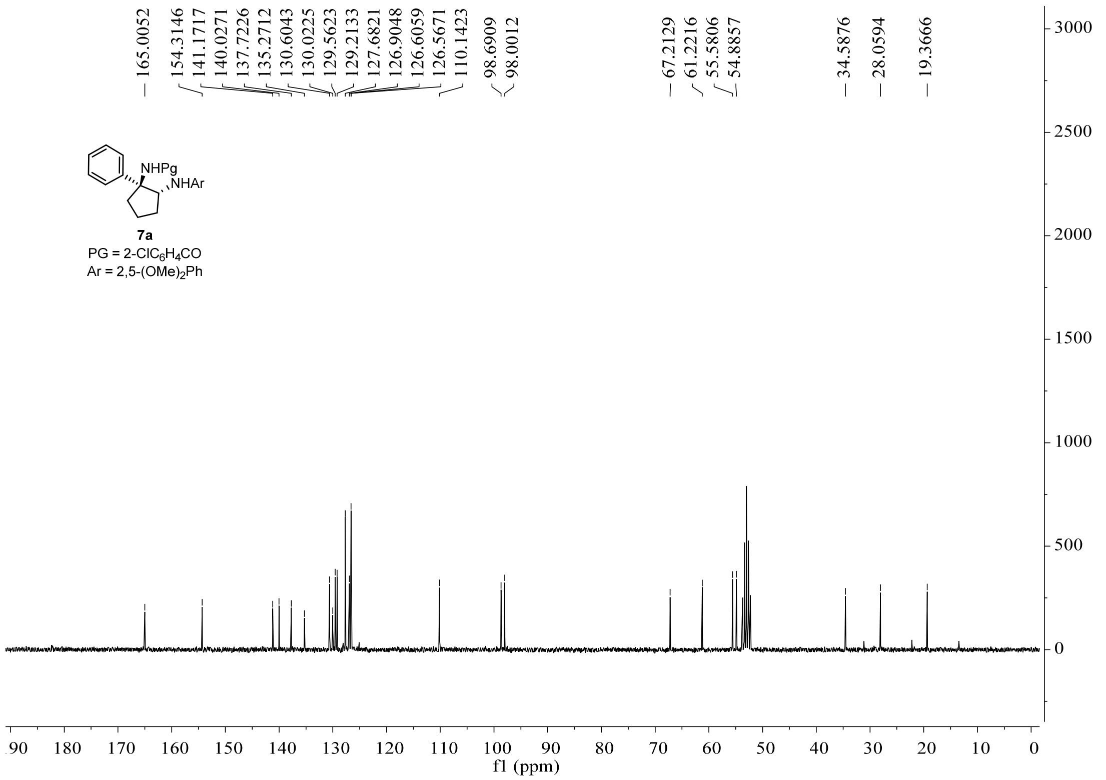
165.0052
154.3146
141.1717
140.0271
137.7226
135.2712
130.6043
130.0225
129.5623
129.2133
127.6821
126.9048
126.6059
126.5671
110.1423
98.6909
98.0012
67.2129
61.2216
55.5806
54.8857
34.5876
28.0594
19.3666
NHPg
NHAr
7a
PG = 2-ClC6H4CO
Ar = 2,5-(OMe)2Ph
f1 (ppm)

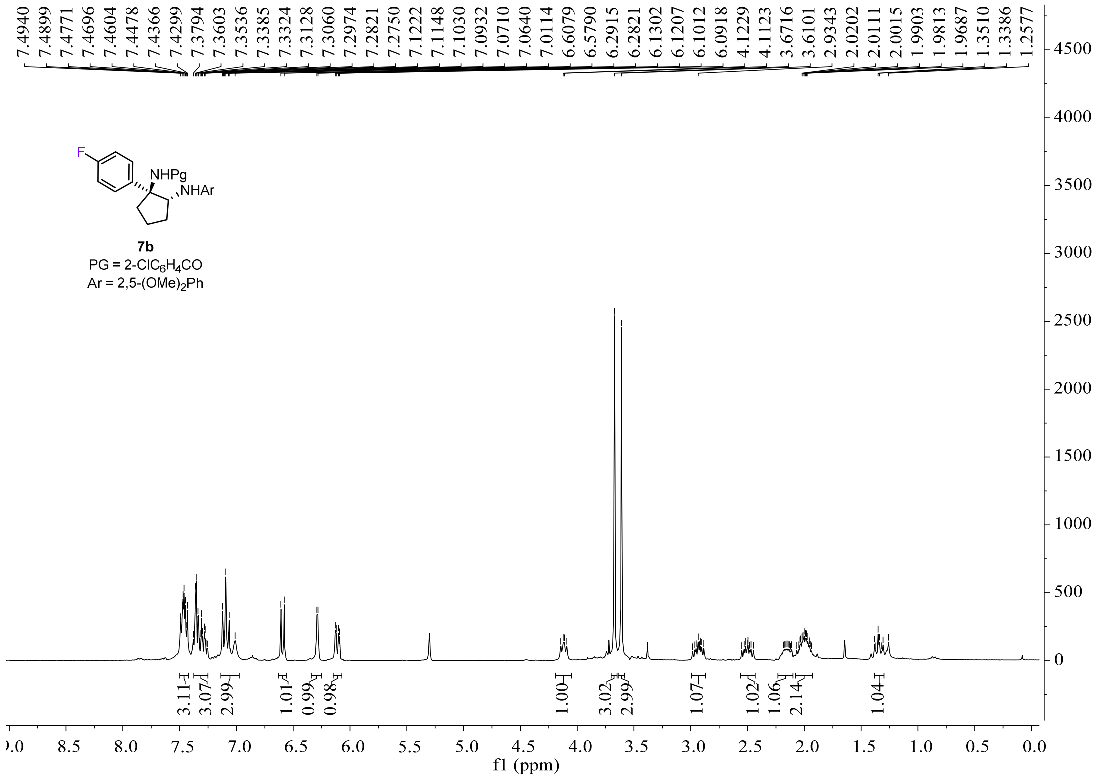
7.4940
7.4899
7.4771
7.4696
7.4604
7.4478
7.4366
7.4299
7.3794
7.3603
7.3536
7.3385
7.3324
7.3128
7.3060
7.2974
7.2821
7.2750
7.1222
7.1148
7.1030
7.0932
7.0710
7.0640
7.0114
6.6079
6.5790
6.2915
6.2821
6.1302
6.1207
6.1012
6.0918
4.1229
4.1123
3.6716
3.6101
2.9343
2.0202
2.0111
2.0015
1.9903
1.9813
1.9687
1.3510
1.3386
1.2577
F
NHPg
NHAr
7b
PG = 2-ClC6H4CO
Ar = 2,5-(OMe)2Ph
3.11
3.07
2.99
1.01
0.99
0.98
1.00
3.02
2.99
1.07
1.02
1.06
2.14
1.04
f1 (ppm)

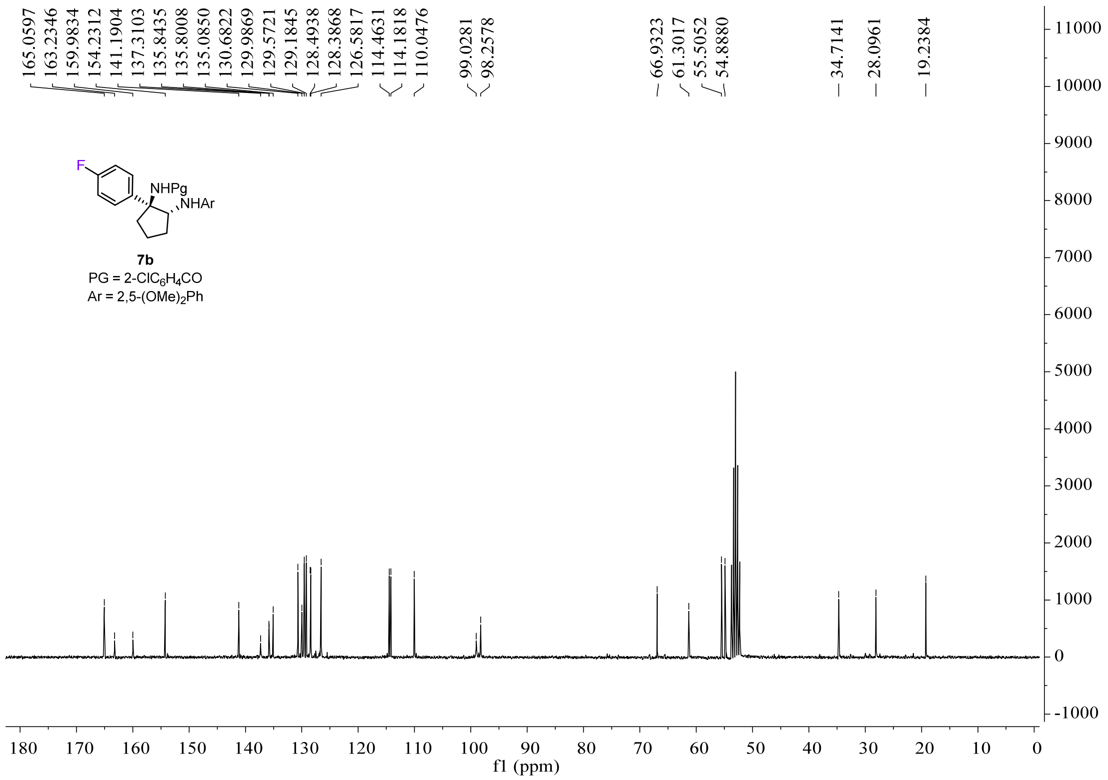
165.0597
163.2346
159.9834
154.2312
141.1904
137.3103
135.8435
135.8008
135.0850
130.6822
129.9869
129.5721
129.1845
128.4938
128.3868
126.5817
114.4631
114.1818
110.0476
99.0281
98.2578
66.9323
61.3017
55.5052
54.8880
34.7141
28.0961
19.2384
F
NHPg
NHAr
7b
PG = 2-ClC6H4CO
Ar = 2,5-(OMe)2Ph
f1 (ppm)

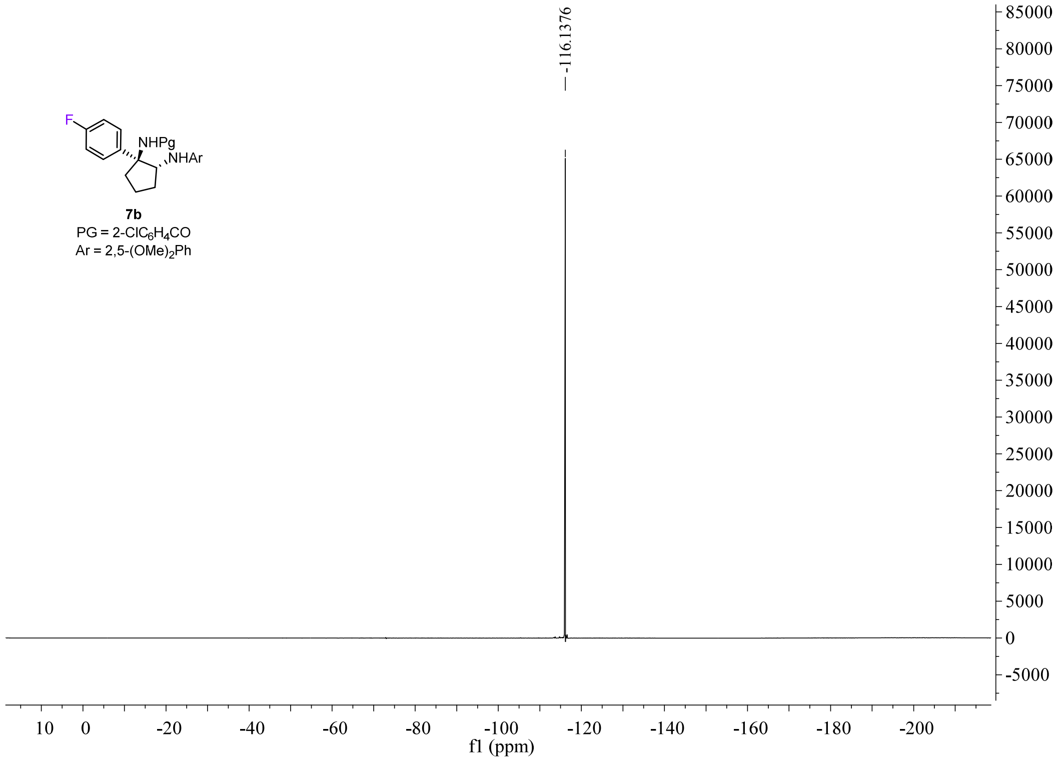
-116.1376
F
NHPg
NHAr
7b
PG = 2-ClC6H4CO
Ar = 2,5-(OMe)2Ph
f1 (ppm)

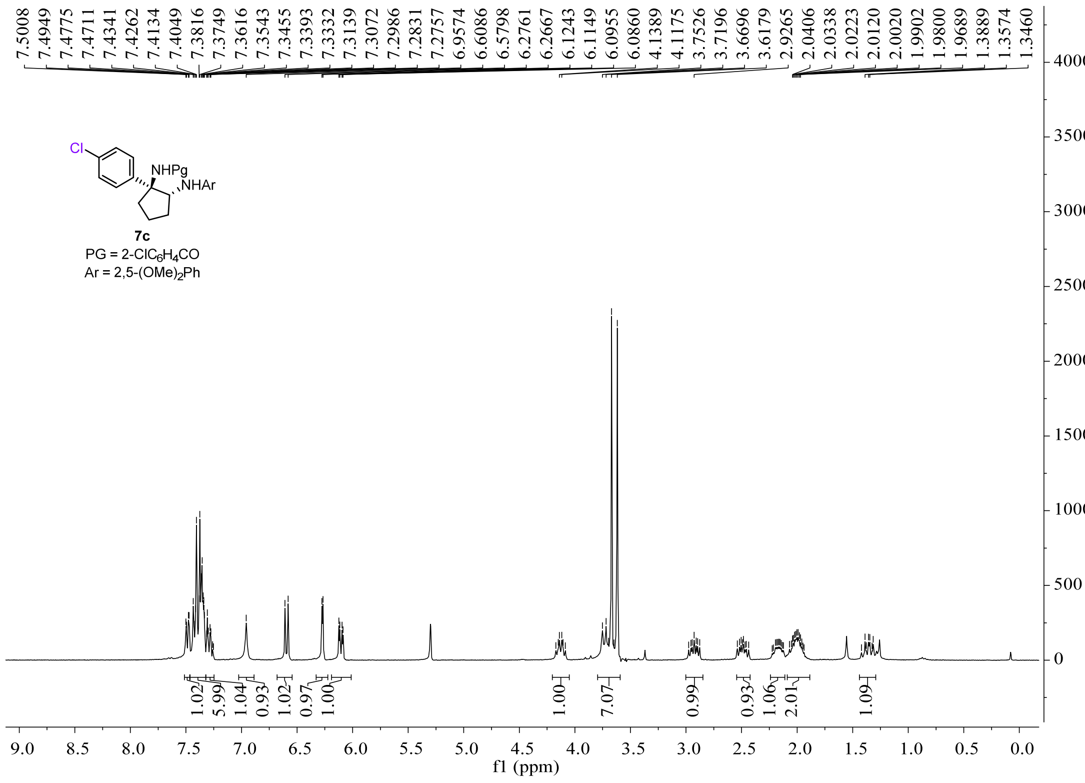

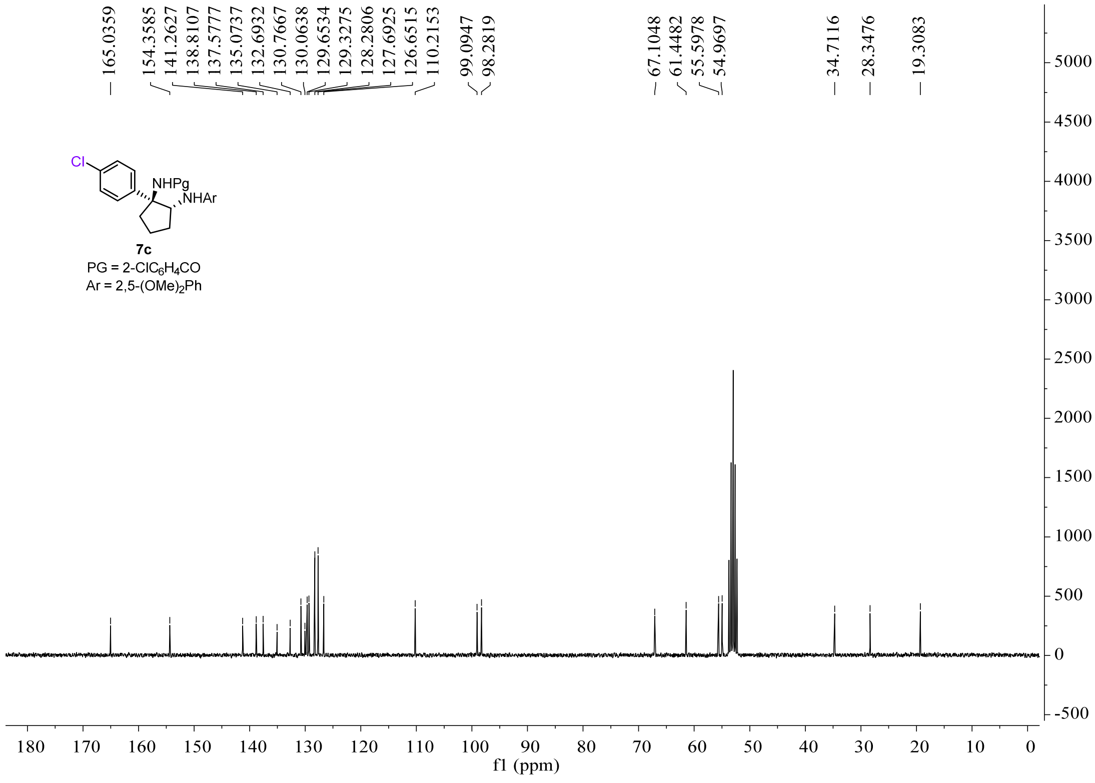

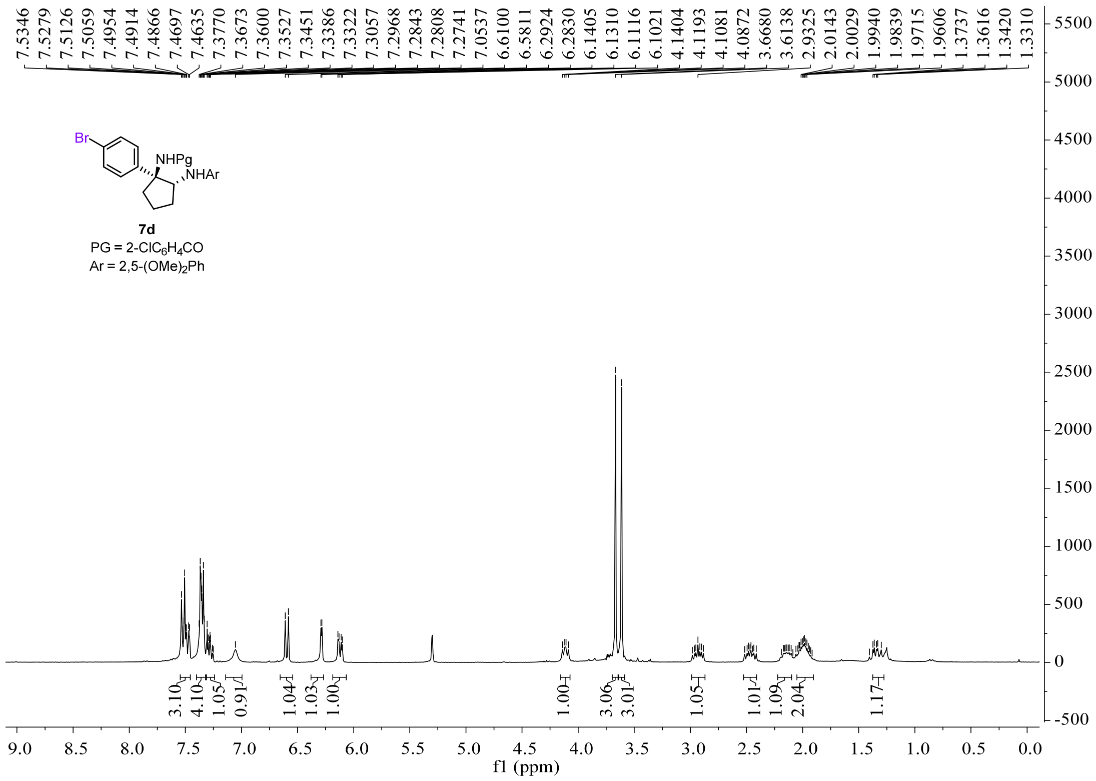
7.5346 7.5279 7.5126 7.5059 7.4954 7.4914 7.4866 7.4697 7.4635 7.3770 7.3673 7.3600 7.3527 7.3451 7.3386 7.3322 7.3057 7.2968 7.2843 7.2808 7.2741 7.0537 6.6100 6.5811 6.2924 6.2830 6.1405 6.1310 6.1116 6.1021 4.1404 4.1193 4.1081 4.0872 3.6680 3.6138 2.9325 2.0143 2.0029 1.9940 1.9839 1.9715 1.9606 1.3737 1.3616 1.3420 1.3310
Br
NHPg
NHAr
7d
PG = 2-ClC6H4CO
Ar = 2,5-(OMe)2Ph
3.10 4.10 1.05 0.91 1.04 1.03 1.00 1.00 3.06 3.01 1.05 1.01 1.09 2.04 1.17
f1 (ppm)

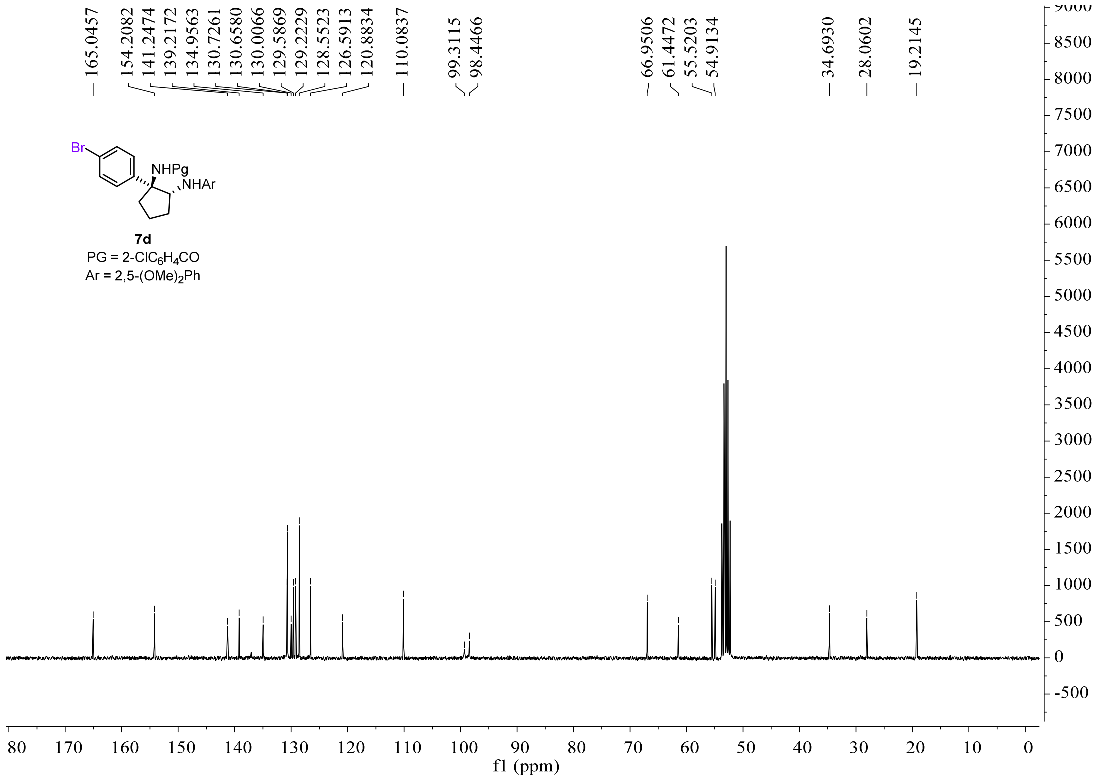
165.0457 154.2082 141.2474 139.2172 134.9563 130.7261 130.6580 130.0066 129.5869 129.2229 128.5523 126.5913 120.8834 110.0837 99.3115 98.4466 66.9506 61.4472 55.5203 54.9134 34.6930 28.0602 19.2145
Br
NHPg
NHAr
7d
PG = 2-ClC6H4CO
Ar = 2,5-(OMe)2Ph
f1 (ppm)

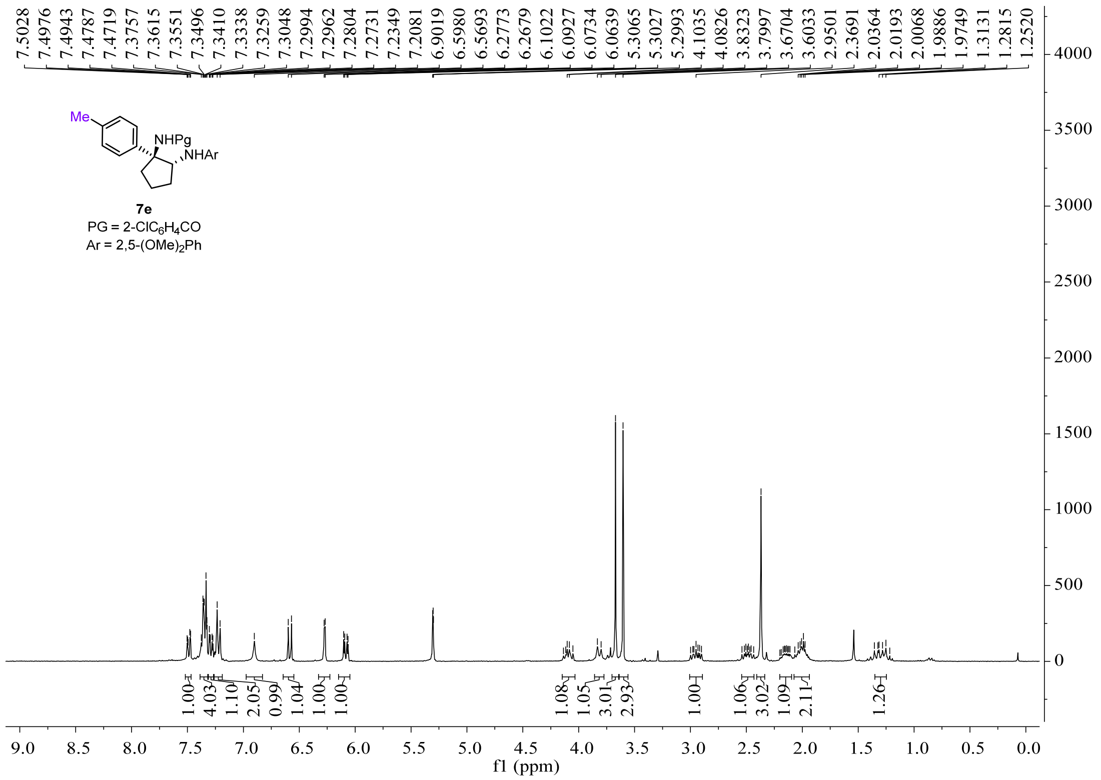
7.5028
7.4976
7.4943
7.4787
7.4719
7.3757
7.3615
7.3551
7.3496
7.3410
7.3338
7.3259
7.3048
7.2994
7.2962
7.2804
7.2731
7.2349
7.2081
6.9019
6.5980
6.5693
6.2773
6.2679
6.1022
6.0927
6.0734
6.0639
5.3065
5.3027
5.2993
4.1035
4.0826
3.8323
3.7997
3.6704
3.6033
2.9501
2.3691
2.0364
2.0193
2.0068
1.9886
1.9749
1.3131
1.2815
1.2520
Me
NHPg
NHAr
7e
PG = 2-ClC6H4CO
Ar = 2,5-(OMe)2Ph
1.00
4.03
1.10
2.05
0.99
1.04
1.00
1.00
1.08
1.05
3.01
2.93
1.00
1.06
3.02
1.09
2.11
1.26
f1 (ppm)

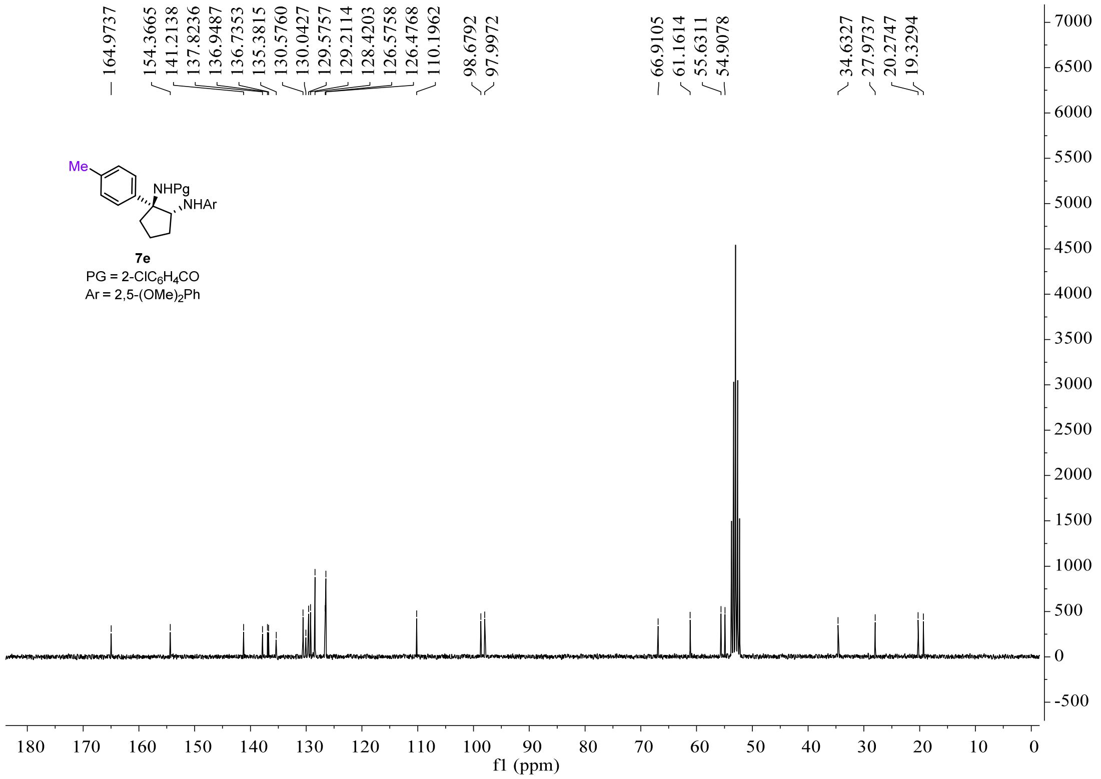
164.9737
154.3665
141.2138
137.8236
136.9487
136.7353
135.3815
130.5760
130.0427
129.5757
129.2114
128.4203
126.5758
126.4768
110.1962
98.6792
97.9972
66.9105
61.1614
55.6311
54.9078
34.6327
27.9737
20.2747
19.3294
Me
NHPg
NHAr
7e
PG = 2-ClC6H4CO
Ar = 2,5-(OMe)2Ph
f1 (ppm)

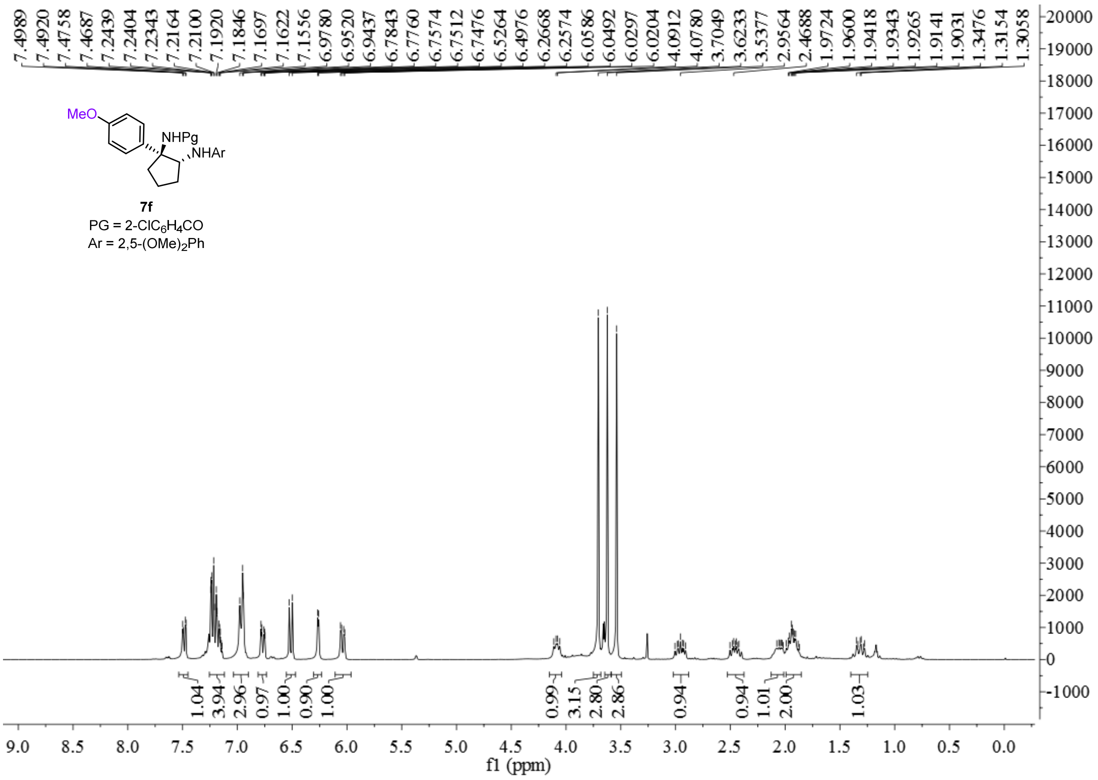

7.4989
7.4920
7.4758
7.4687
7.2439
7.2404
7.2343
7.2164
7.2100
7.1920
7.1846
7.1697
7.1622
7.1556
6.9780
6.9520
6.9437
6.7843
6.7760
6.7574
6.7512
6.7476
6.5264
6.4976
6.2668
6.2574
6.0586
6.0492
6.0297
6.0204
4.0912
4.0780
3.7049
3.6233
3.5377
2.9564
2.4688
1.9724
1.9600
1.9418
1.9343
1.9265
1.9141
1.9031
1.3476
1.3154
1.3058
MeO
NHPg
NHAr
7f
PG = 2-ClC6H4CO
Ar = 2,5-(OMe)2Ph
1.04
3.94
2.96
0.97
1.00
0.90
1.00
0.99
3.15
2.80
2.86
0.94
0.94
1.01
2.00
1.03
f1 (ppm)

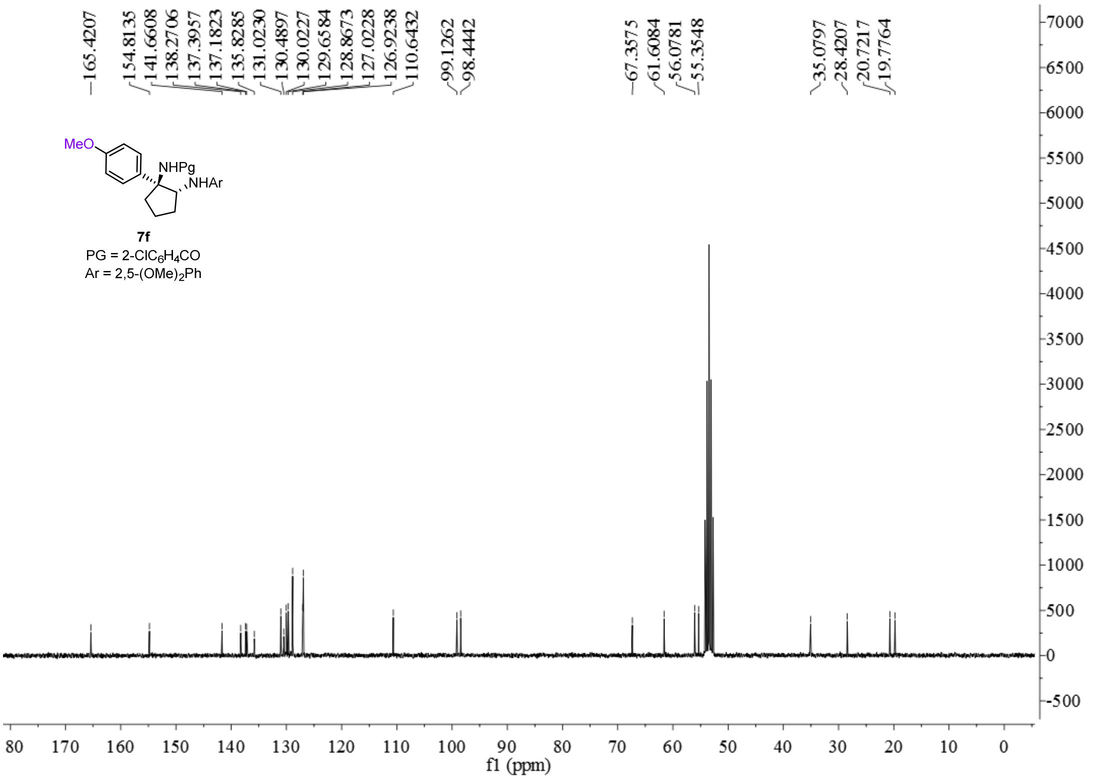

165.4207
154.8135
141.6608
138.2706
137.3957
137.1823
135.8285
131.0230
130.4897
130.0227
129.6584
128.8673
127.0228
126.9238
110.6432
99.1262
98.4442
67.3575
61.6084
56.0781
55.3548
35.0797
28.4207
20.7217
19.7764
MeO
NHPg
NHAr
7f
PG = 2-ClC6H4CO
Ar = 2,5-(OMe)2Ph
f1 (ppm)

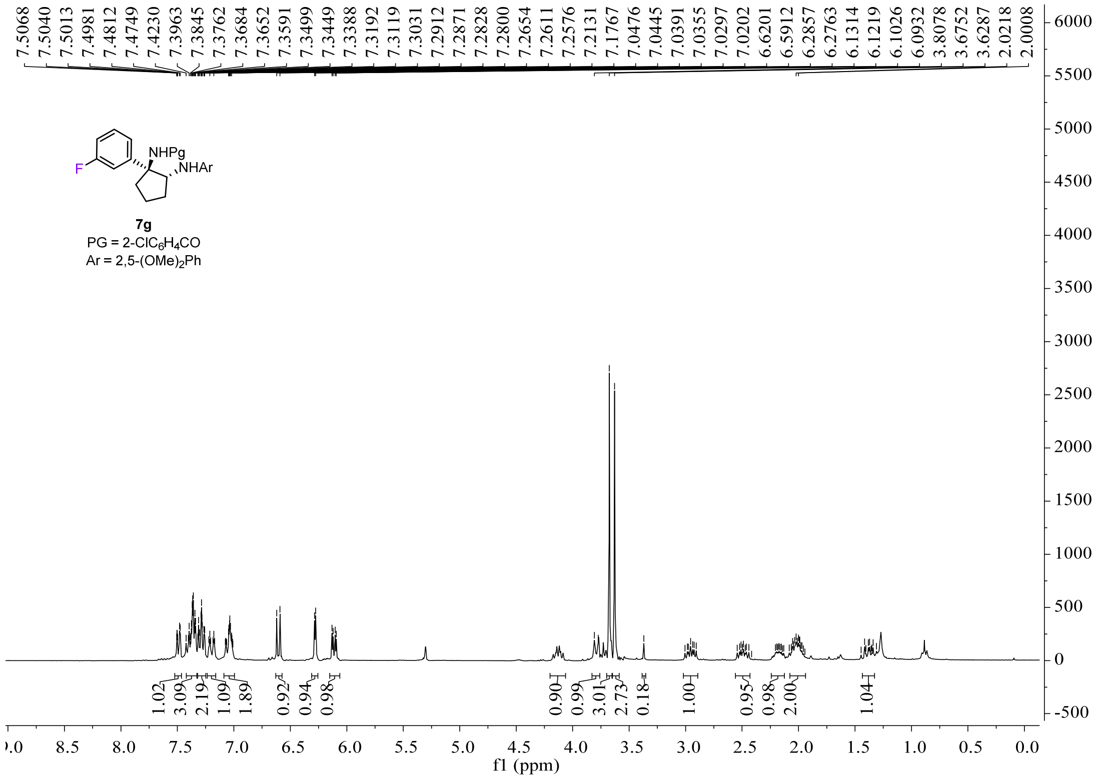

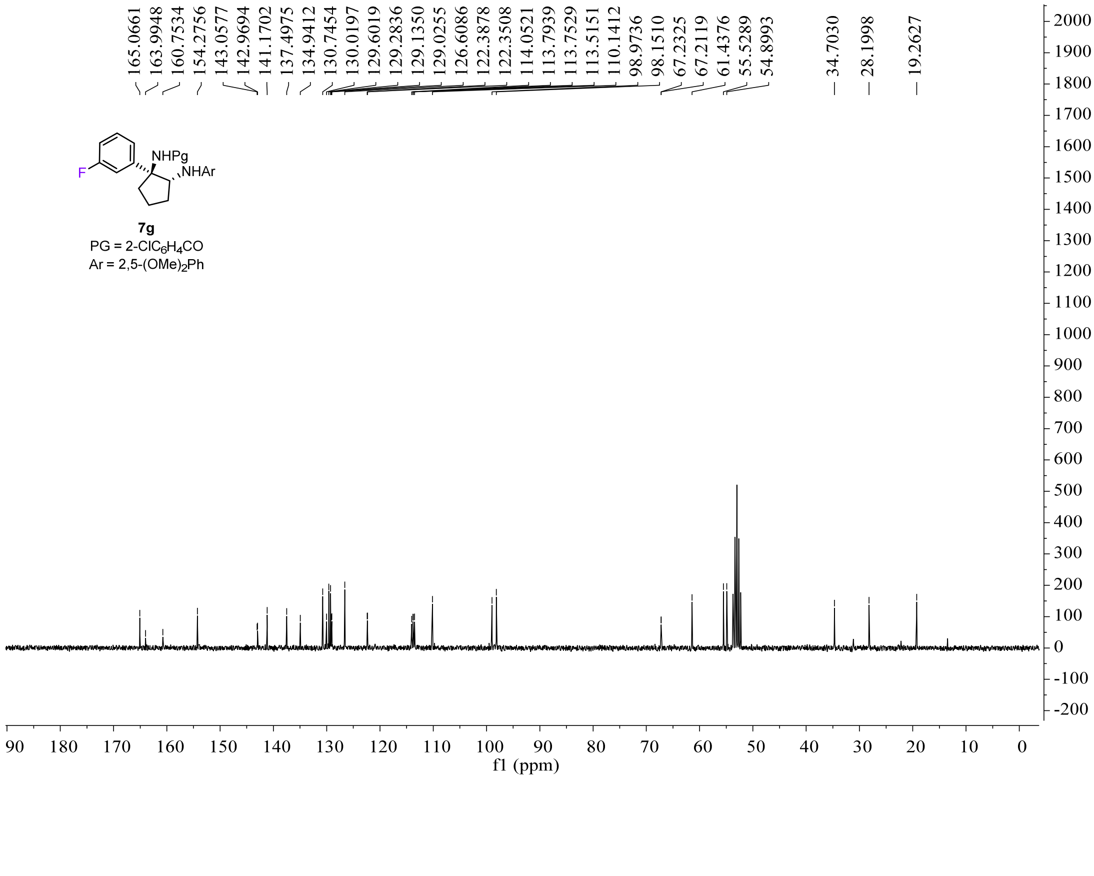

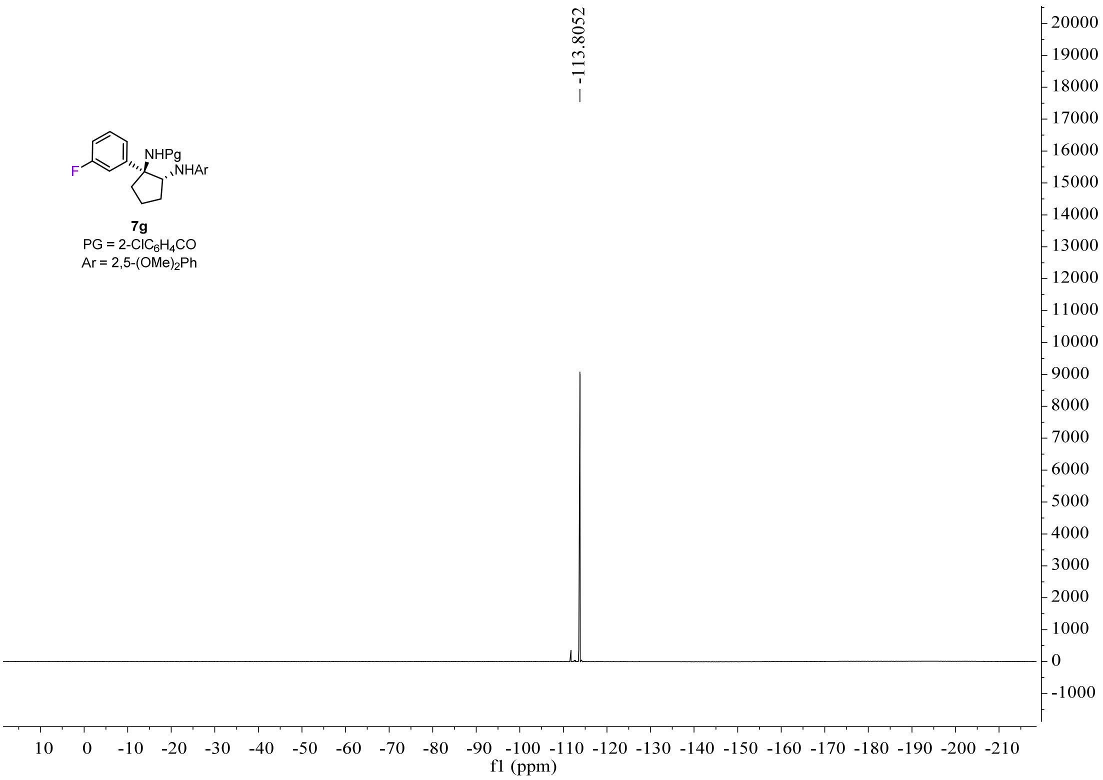

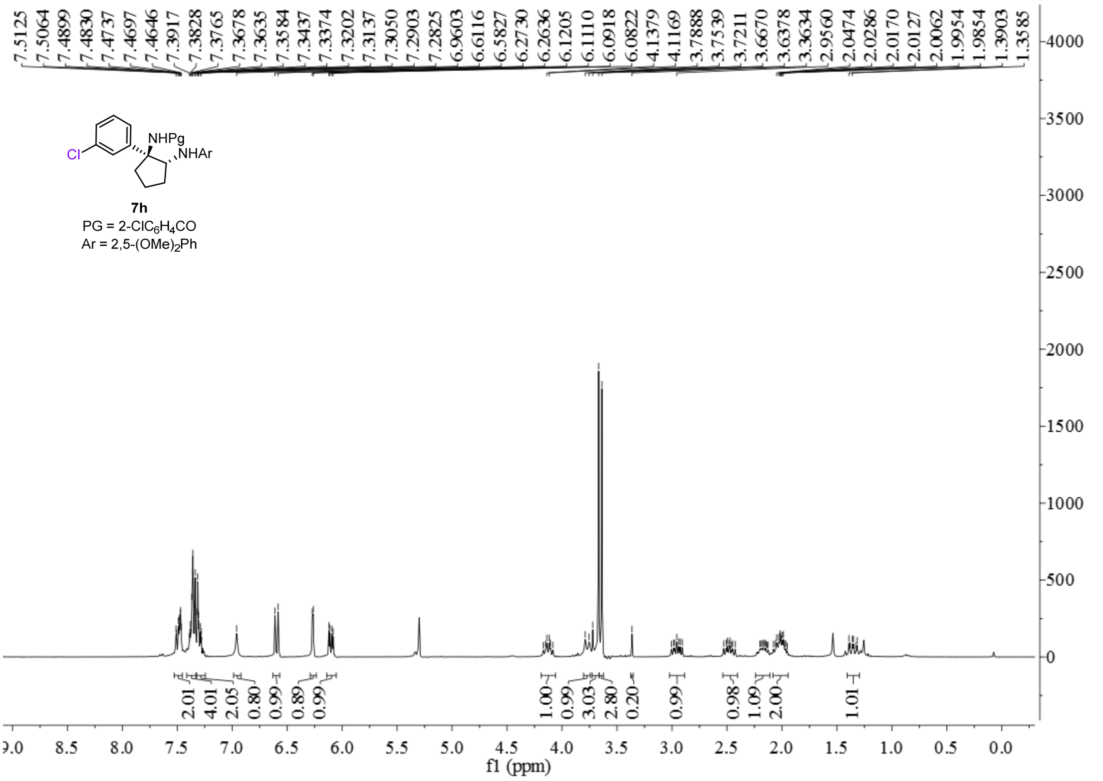

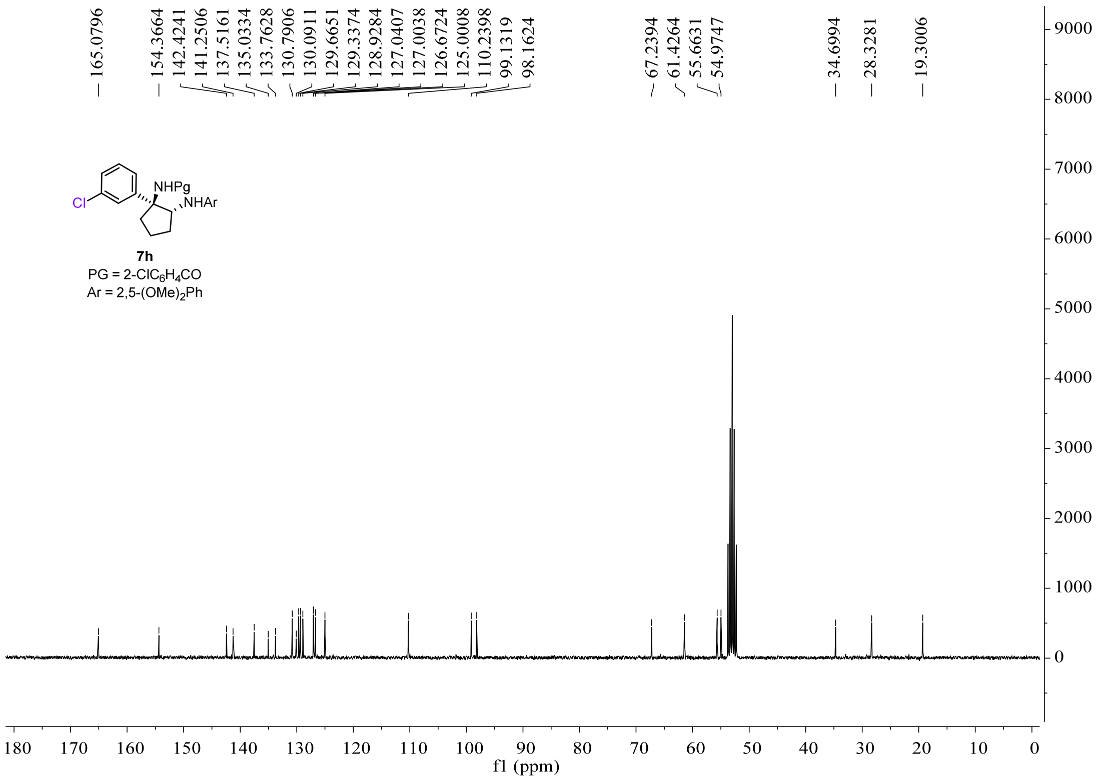
7h
PG = 2-ClC6H4CO
Ar = 2,5-(OMe)2Ph
f1 (ppm)

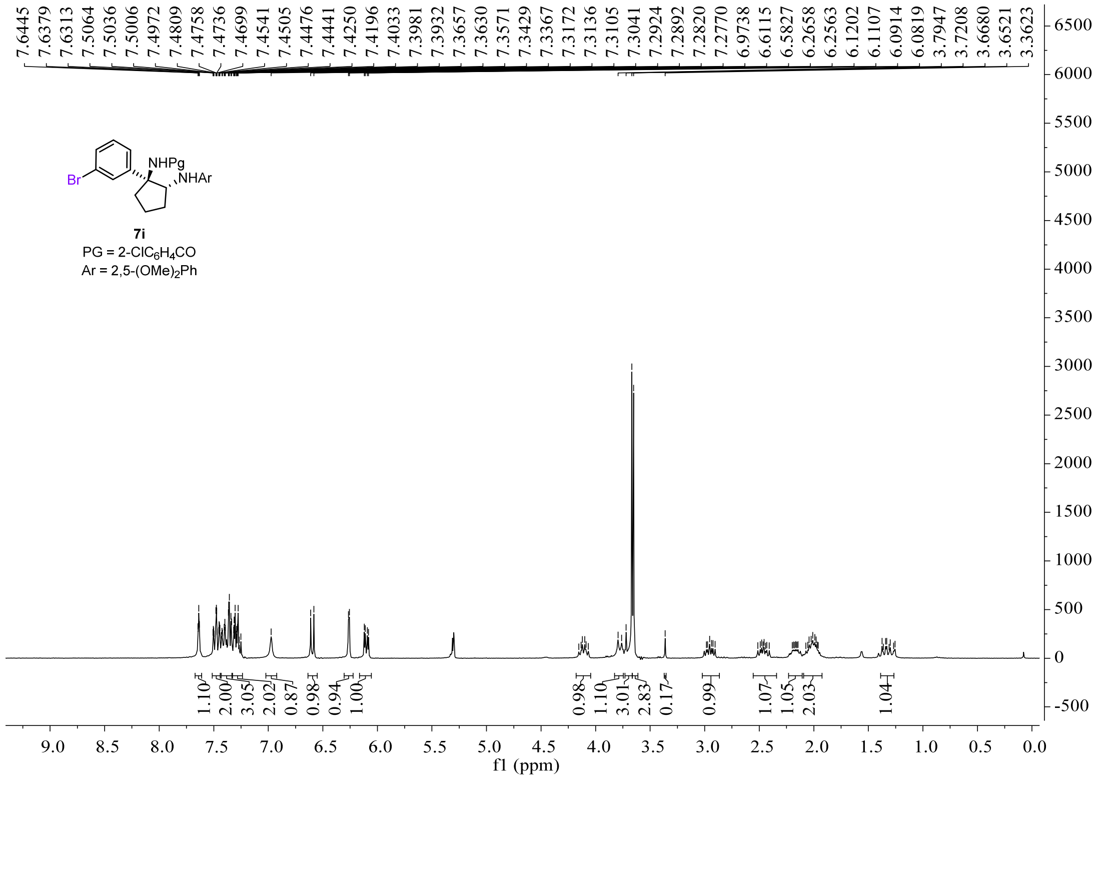
7i
PG = 2-ClC6H4CO
Ar = 2,5-(OMe)2Ph
f1 (ppm)

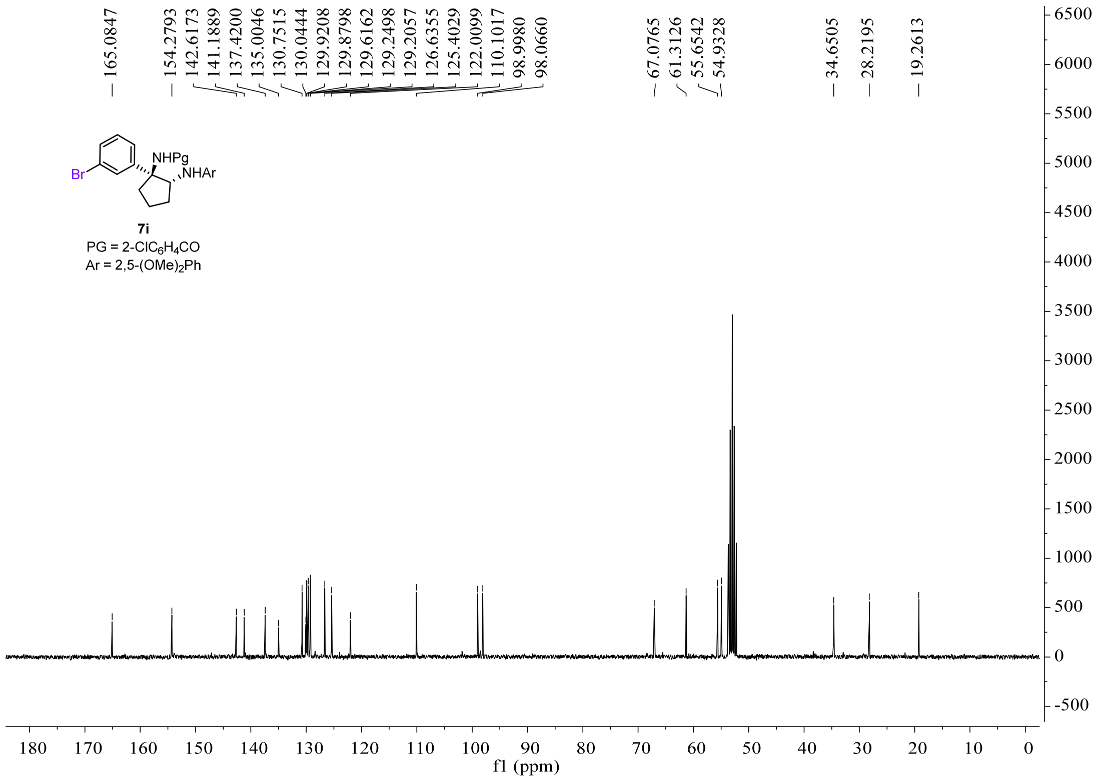

165.0847
154.2793
142.6173
141.1889
137.4200
135.0046
130.7515
130.0444
129.9208
129.8798
129.6162
129.2498
129.2057
126.6355
125.4029
122.0099
110.1017
98.9980
98.0660
67.0765
61.3126
55.6542
54.9328
34.6505
28.2195
19.2613
NHPg
NHAr
Br
7i
PG = 2-ClC6H4CO
Ar = 2,5-(OMe)2Ph
f1 (ppm)

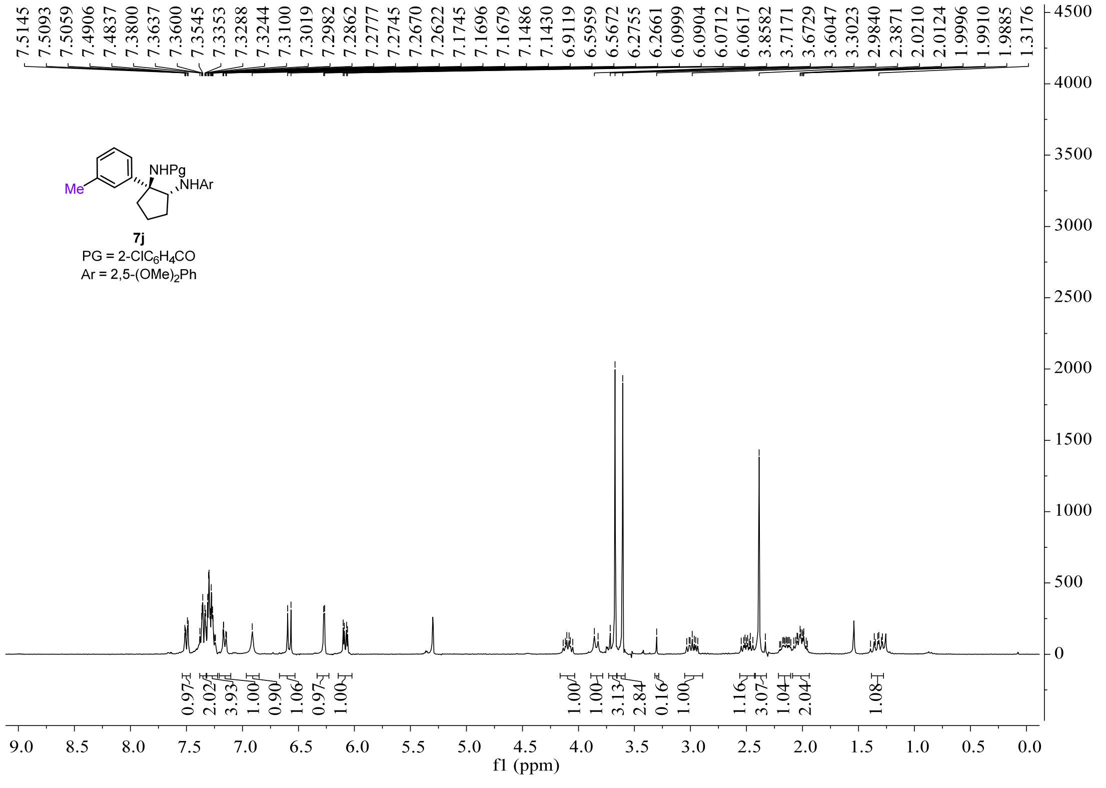

7.5145
7.5093
7.5059
7.4906
7.4837
7.3800
7.3637
7.3600
7.3545
7.3353
7.3288
7.3244
7.3100
7.3019
7.2982
7.2862
7.2777
7.2745
7.2670
7.2622
7.1745
7.1696
7.1679
7.1486
7.1430
6.9119
6.5959
6.5672
6.2755
6.2661
6.0999
6.0904
6.0712
6.0617
3.8582
3.7171
3.6729
3.6047
3.3023
2.9840
2.3871
2.0210
2.0124
1.9996
1.9910
1.9885
1.3176
NHPg
NHAr
Me
7j
PG = 2-ClC6H4CO
Ar = 2,5-(OMe)2Ph
0.97
2.02
3.93
1.00
0.90
1.06
0.97
1.00
1.00
1.00
3.13
2.84
0.16
1.00
1.16
3.07
1.04
2.04
1.08
f1 (ppm)

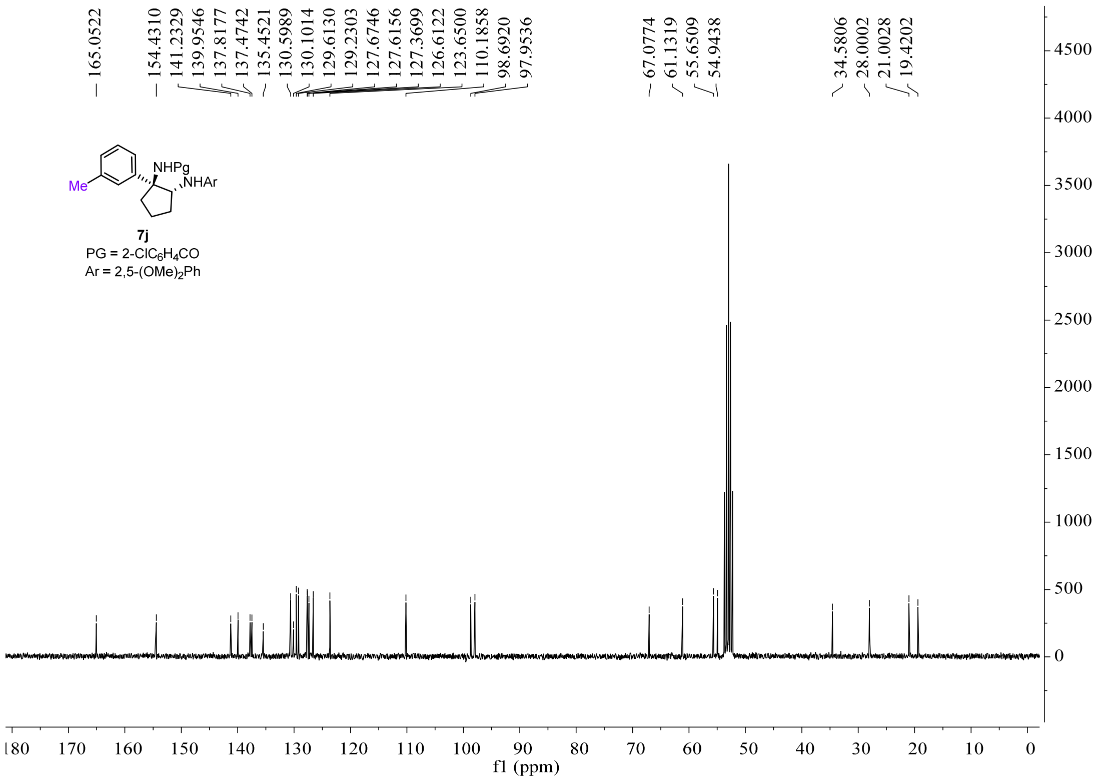
165.0522
154.4310
141.2329
139.9546
137.8177
137.4742
135.4521
130.5989
130.1014
129.6130
129.2303
127.6746
127.6156
127.3699
126.6122
123.6500
110.1858
98.6920
97.9536
67.0774
61.1319
55.6509
54.9438
34.5806
28.0002
21.0028
19.4202
7j
PG = 2-ClC6H4CO
Ar = 2,5-(OMe)2Ph
f1 (ppm)

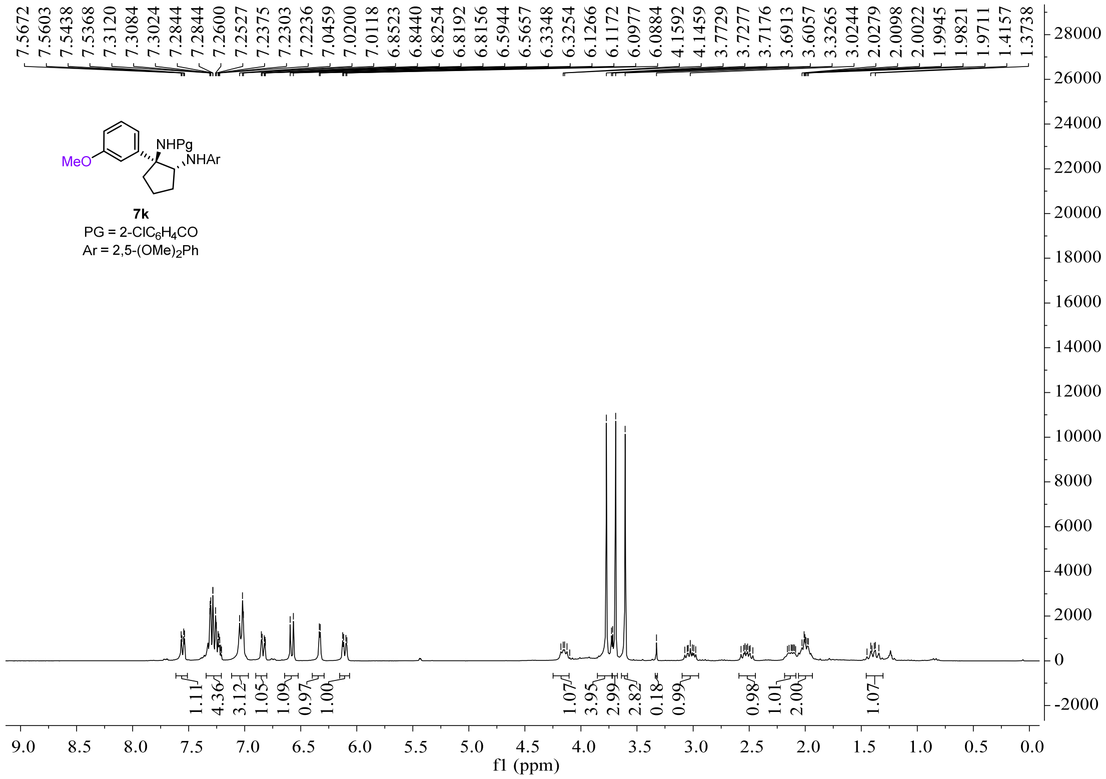
7k
PG = 2-ClC6H4CO
Ar = 2,5-(OMe)2Ph
f1 (ppm)

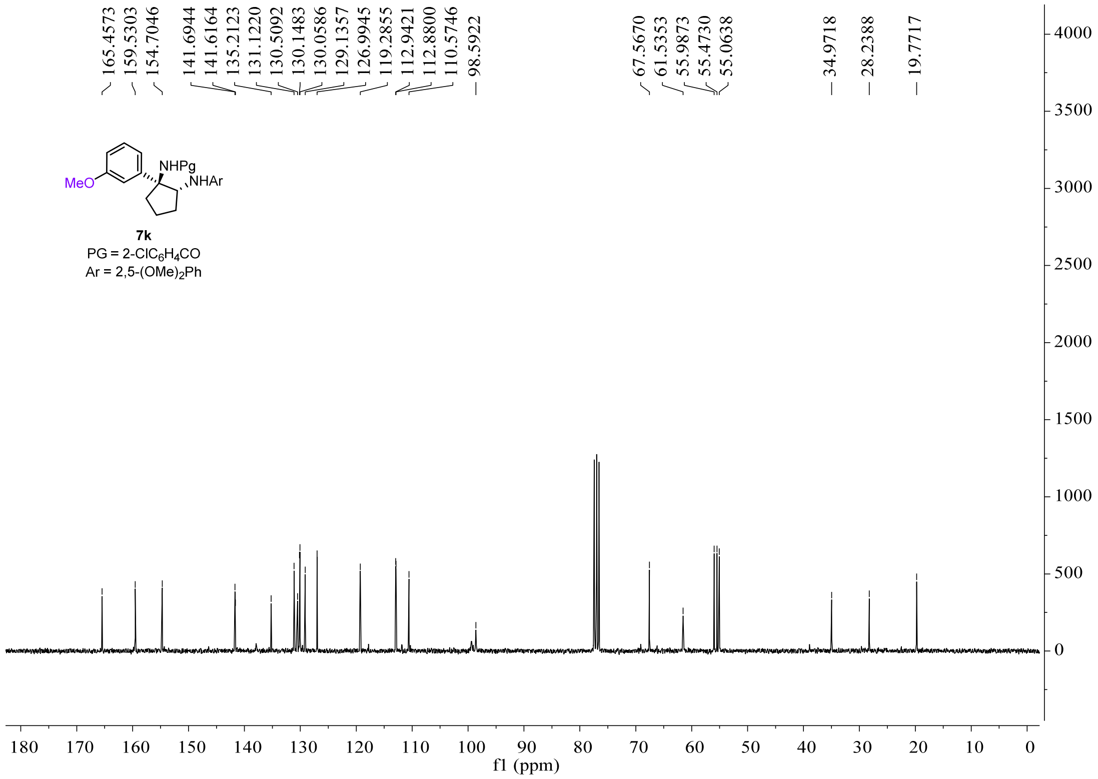
165.4573
159.5303
154.7046
141.6944
141.6164
135.2123
131.1220
130.5092
130.1483
130.0586
129.1357
126.9945
119.2855
112.9421
112.8800
110.5746
98.5922
67.5670
61.5353
55.9873
55.4730
55.0638
34.9718
28.2388
19.7717
MeO
NHPg
NHAr
7k
PG = 2-ClC6H4CO
Ar = 2,5-(OMe)2Ph
f1 (ppm)

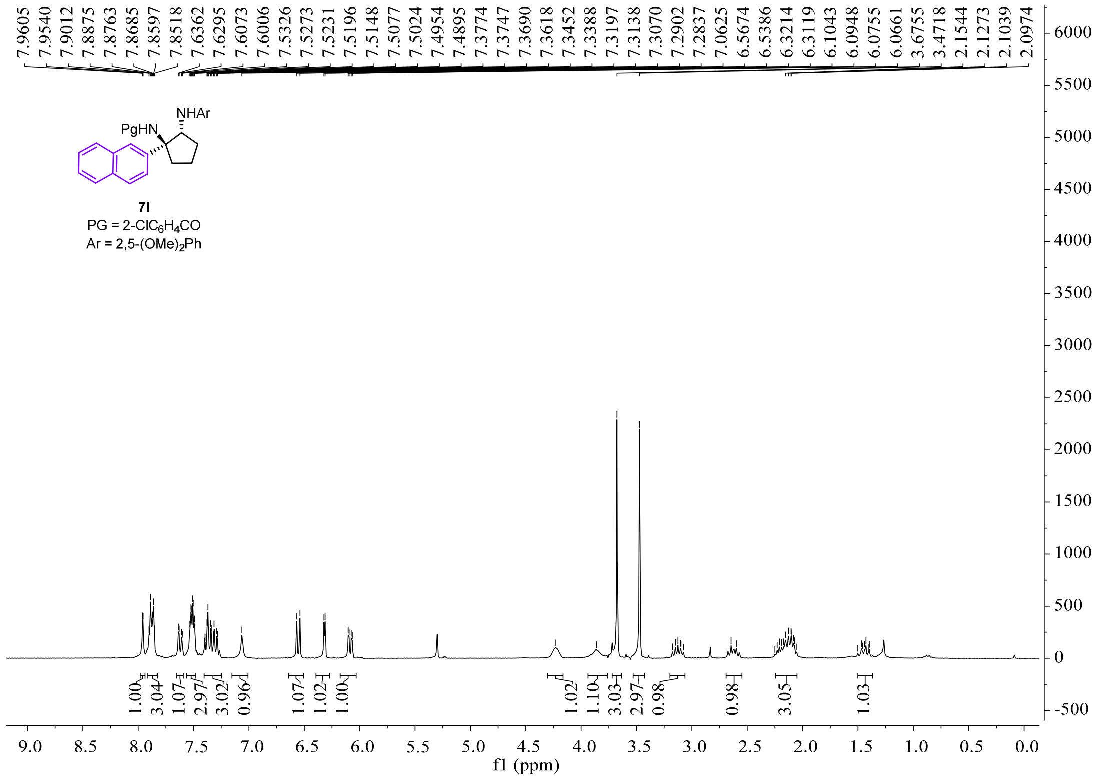
7.9605
7.9540
7.9012
7.8875
7.8763
7.8685
7.8597
7.8518
7.6362
7.6295
7.6073
7.6006
7.5326
7.5273
7.5231
7.5196
7.5148
7.5077
7.5024
7.4954
7.4895
7.3774
7.3747
7.3690
7.3618
7.3452
7.3388
7.3197
7.3138
7.3070
7.2902
7.2837
7.0625
6.5674
6.5386
6.3214
6.3119
6.1043
6.0948
6.0755
6.0661
3.6755
3.4718
2.1544
2.1273
2.1039
2.0974
PgHN
NHAr
7l
PG = 2-ClC6H4CO
Ar = 2,5-(OMe)2Ph
1.00
3.04
1.07
2.97
3.02
0.96
1.07
1.02
1.00
1.02
1.10
3.03
2.97
0.98
0.98
3.05
1.03
f1 (ppm)

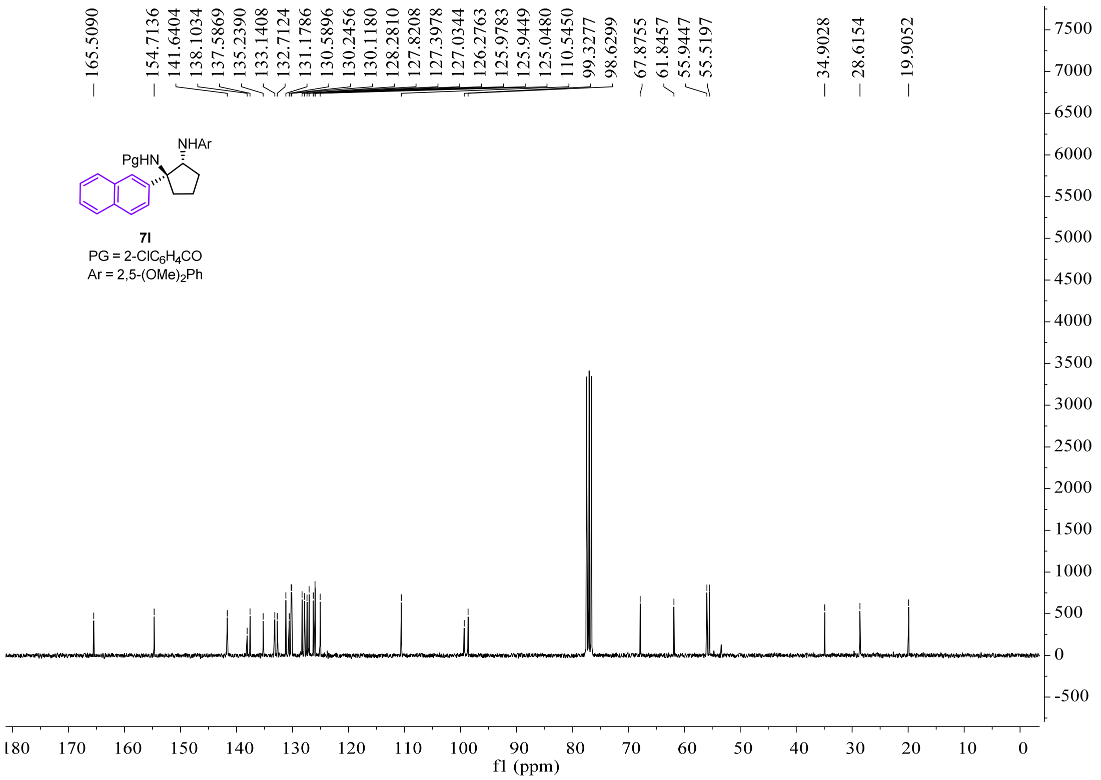
165.5090
154.7136
141.6404
138.1034
137.5869
135.2390
133.1408
132.7124
131.1786
130.5896
130.2456
130.1180
128.2810
127.8208
127.3978
127.0344
126.2763
125.9783
125.9449
125.0480
110.5450
99.3277
98.6299
67.8755
61.8457
55.9447
55.5197
34.9028
28.6154
19.9052
PgHN
NHAr
7l
PG = 2-ClC$_6$H$_4$CO
Ar = 2,5-(OMe)$_2$Ph
f1 (ppm)

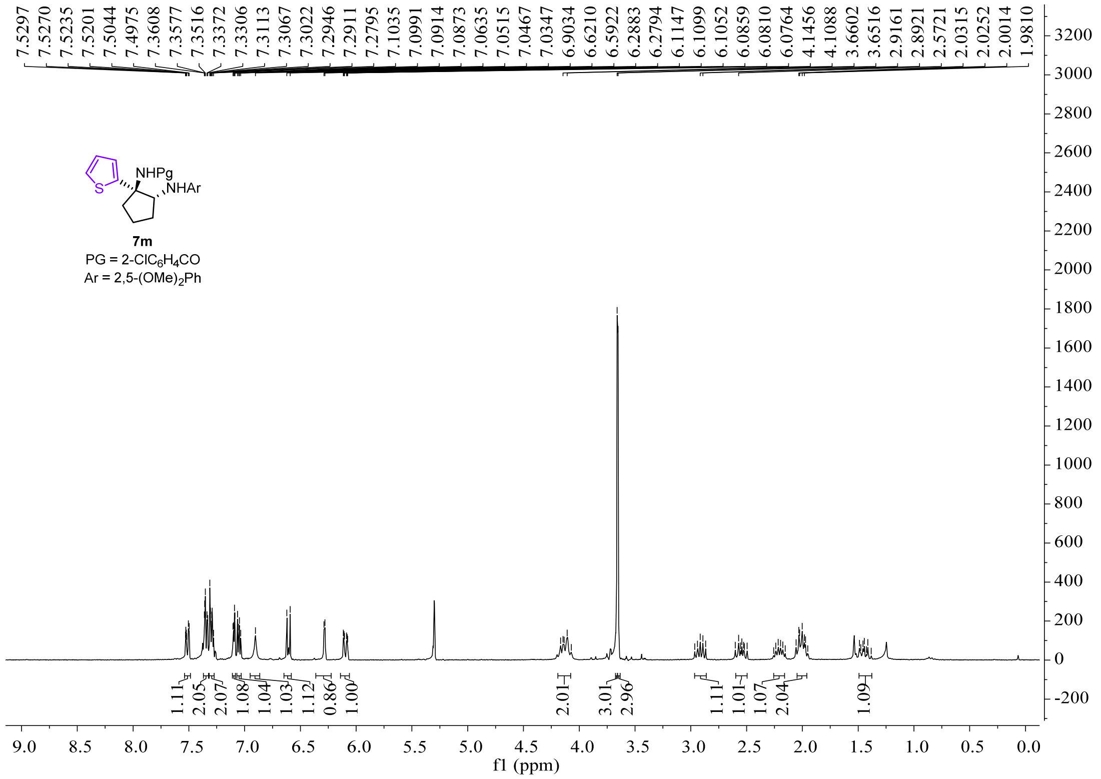
7.5297
7.5270
7.5235
7.5201
7.5044
7.4975
7.3608
7.3577
7.3516
7.3372
7.3306
7.3113
7.3067
7.3022
7.2946
7.2911
7.2795
7.1035
7.0991
7.0914
7.0873
7.0635
7.0515
7.0467
7.0347
6.9034
6.6210
6.5922
6.2883
6.2794
6.1147
6.1099
6.1052
6.0859
6.0810
6.0764
4.1456
4.1088
3.6602
3.6516
2.9161
2.8921
2.5721
2.0315
2.0252
2.0014
1.9810
NHPg
NHAr
S
7m
PG = 2-ClC$_6$H$_4$CO
Ar = 2,5-(OMe)$_2$Ph
1.11
2.05
2.07
1.08
1.04
1.03
1.12
0.86
1.00
2.01
3.01
2.96
1.11
1.01
1.07
2.04
1.09
f1 (ppm)

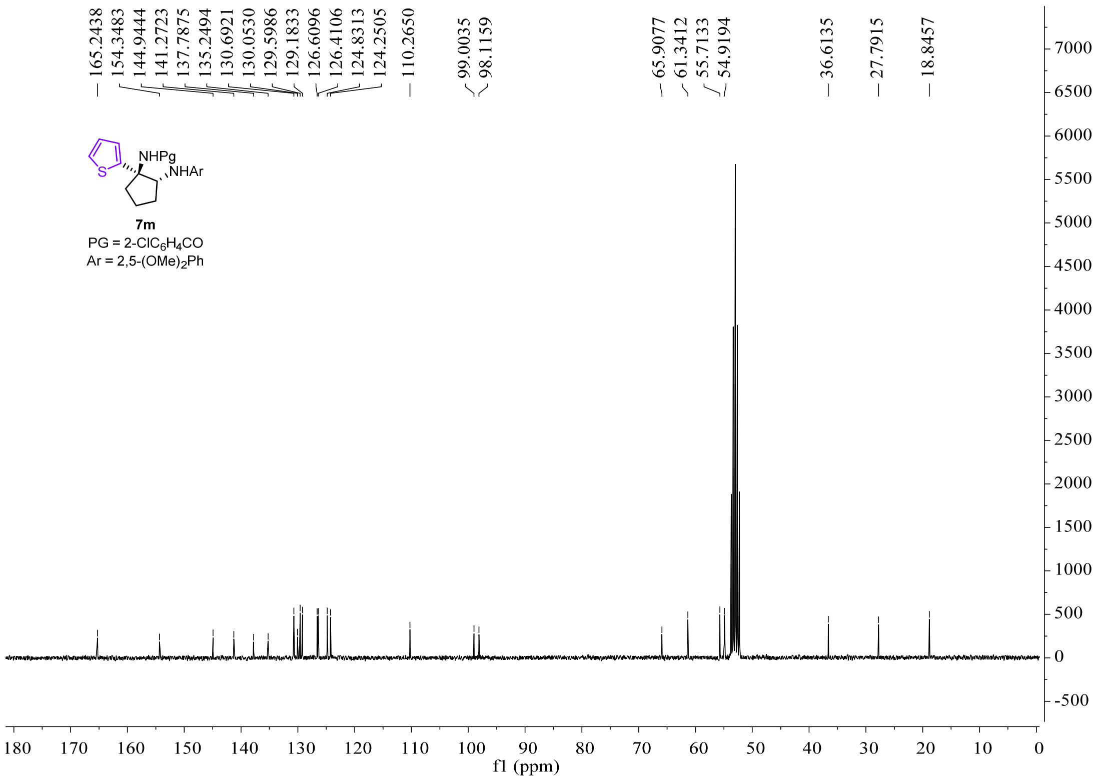

165.2438
154.3483
144.9444
141.2723
137.7875
135.2494
130.6921
130.0530
129.5986
129.1833
126.6096
126.4106
124.8313
124.2505
110.2650
99.0035
98.1159
65.9077
61.3412
55.7133
54.9194
36.6135
27.7915
18.8457
S
NHPg
NHAr
7m
PG = 2-ClC6H4CO
Ar = 2,5-(OMe)2Ph
f1 (ppm)

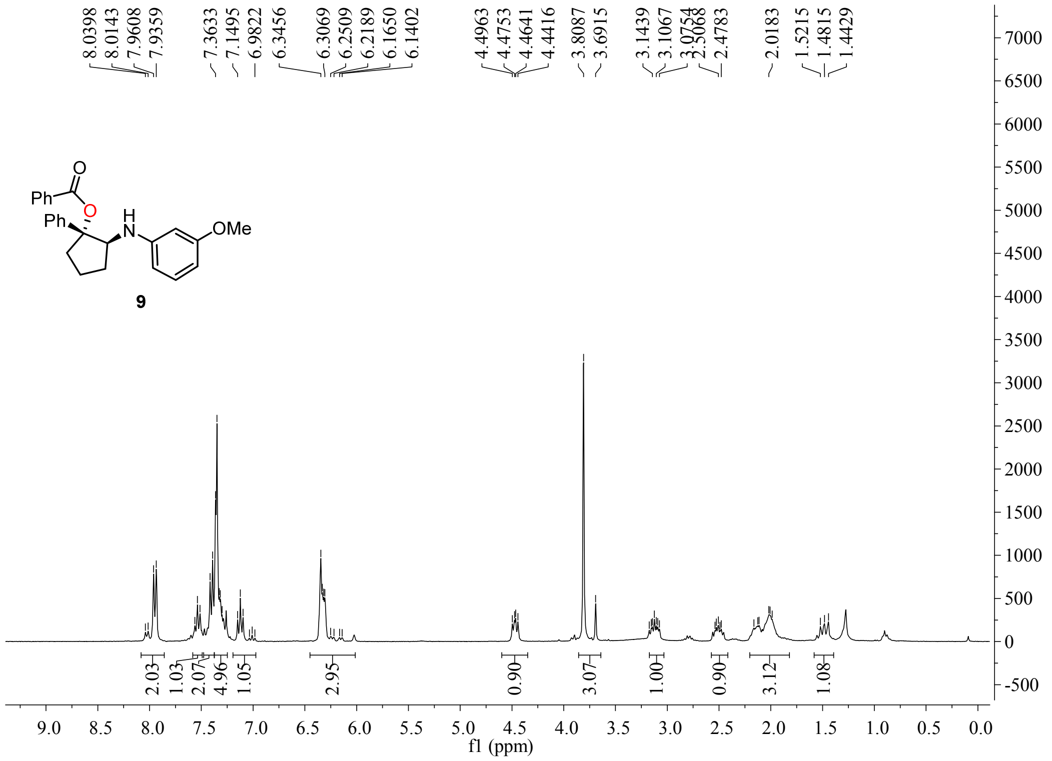

8.0398
8.0143
7.9608
7.9359
7.3633
7.1495
6.9822
6.3456
6.3069
6.2509
6.2189
6.1650
6.1402
4.4963
4.4753
4.4641
4.4416
3.8087
3.6915
3.1439
3.1067
2.4783
2.0183
1.5215
1.4815
1.4429
Ph
O
Ph
H
N
OMe
9
2.03
1.03
2.07
4.96
1.05
2.95
0.90
3.07
1.00
0.90
3.12
1.08
f1 (ppm)

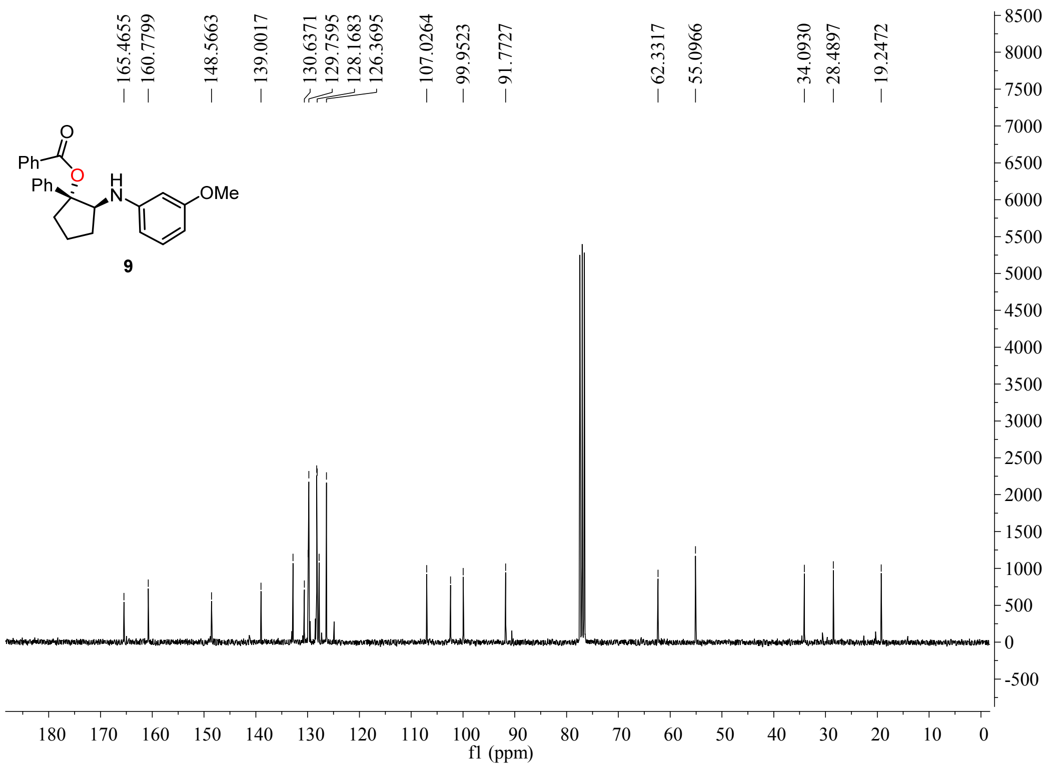
165.4655
160.7799
148.5663
139.0017
130.6371
129.7595
128.1683
126.3695
107.0264
99.9523
91.7727
62.3317
55.0966
34.0930
28.4897
19.2472
Ph
O
Ph
H
N
OMe
9
f1 (ppm)

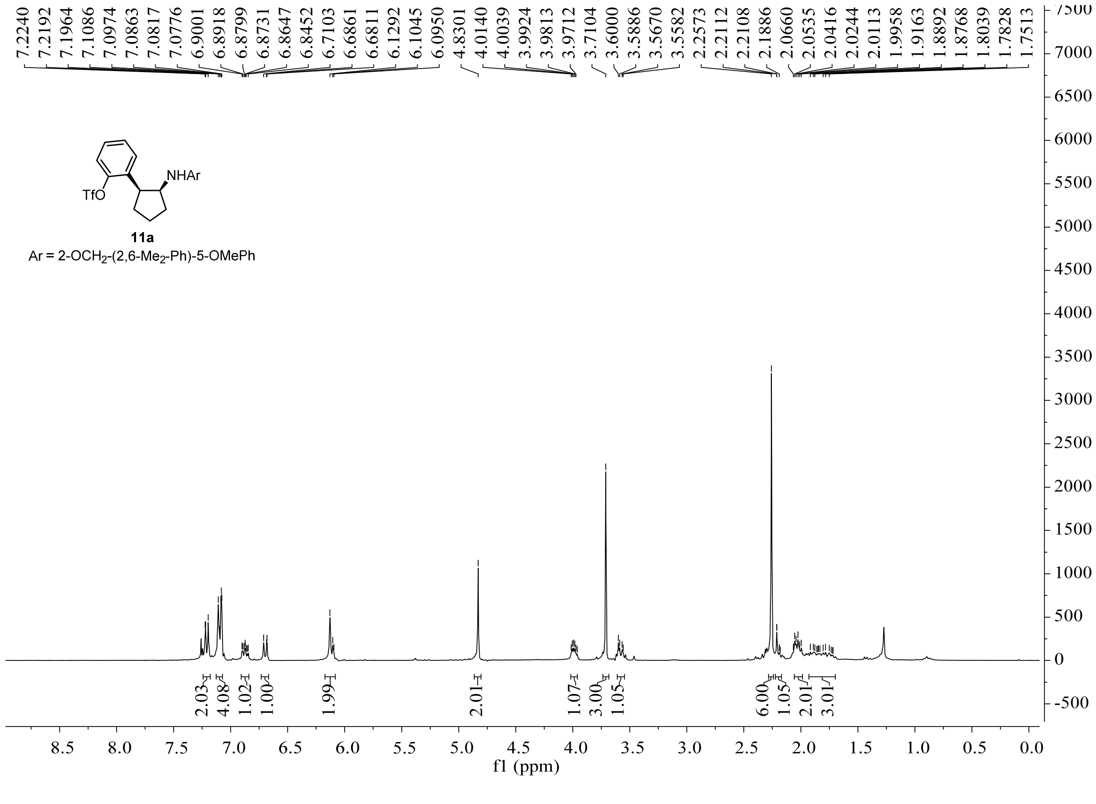
7.2240
7.2192
7.1964
7.1086
7.0974
7.0863
7.0817
7.0776
6.9001
6.8918
6.8799
6.8731
6.8647
6.8452
6.7103
6.6861
6.6811
6.1292
6.1045
6.0950
4.8301
4.0140
4.0039
3.9924
3.9813
3.9712
3.7104
3.6000
3.5886
3.5670
3.5582
2.2573
2.2112
2.2108
2.1886
2.0660
2.0535
2.0416
2.0244
2.0113
1.9958
1.9163
1.8892
1.8768
1.8039
1.7828
1.7513
NHAr
TfO
11a
Ar = 2-OCH2-(2,6-Me2-Ph)-5-OMePh
2.03
4.08
1.02
1.00
1.99
2.01
1.07
3.00
1.05
6.00
1.05
2.01
3.01
f1 (ppm)

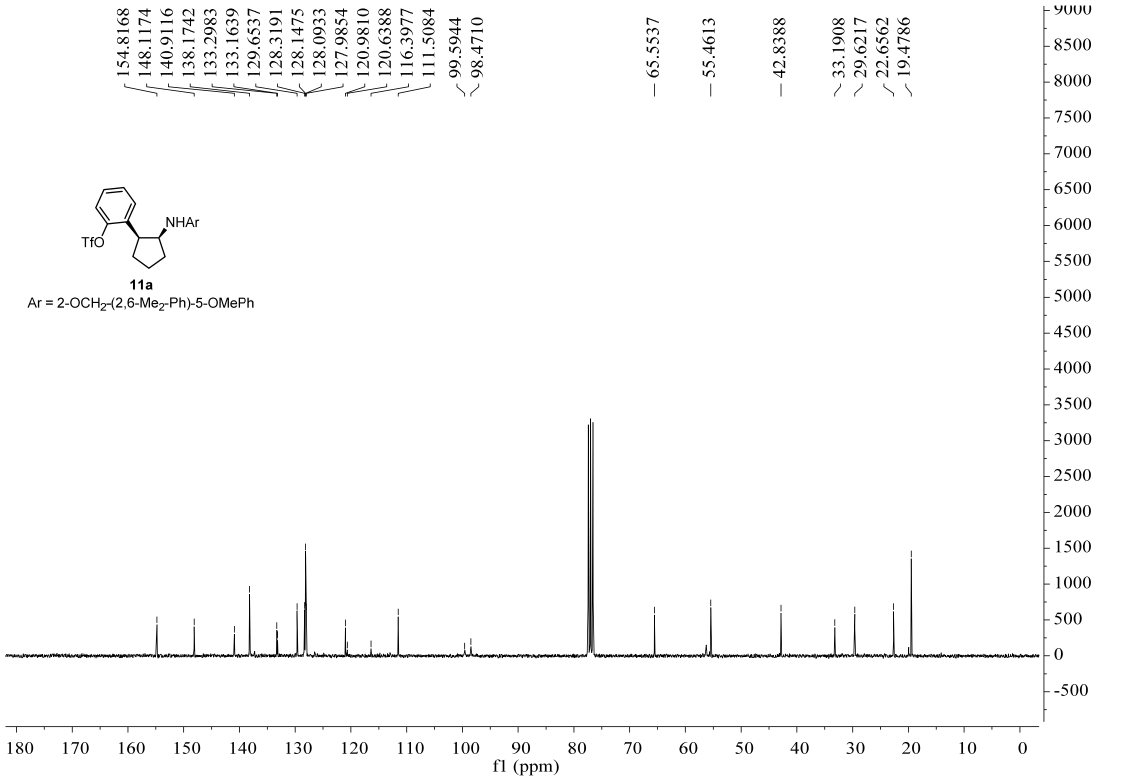
154.8168
148.1174
140.9116
138.1742
133.2983
133.1639
129.6537
128.3191
128.1475
128.0933
127.9854
120.9810
120.6388
116.3977
111.5084
99.5944
98.4710
65.5537
55.4613
42.8388
33.1908
29.6217
22.6562
19.4786
NHAr
TfO
11a
Ar = 2-OCH2-(2,6-Me2-Ph)-5-OMePh
f1 (ppm)

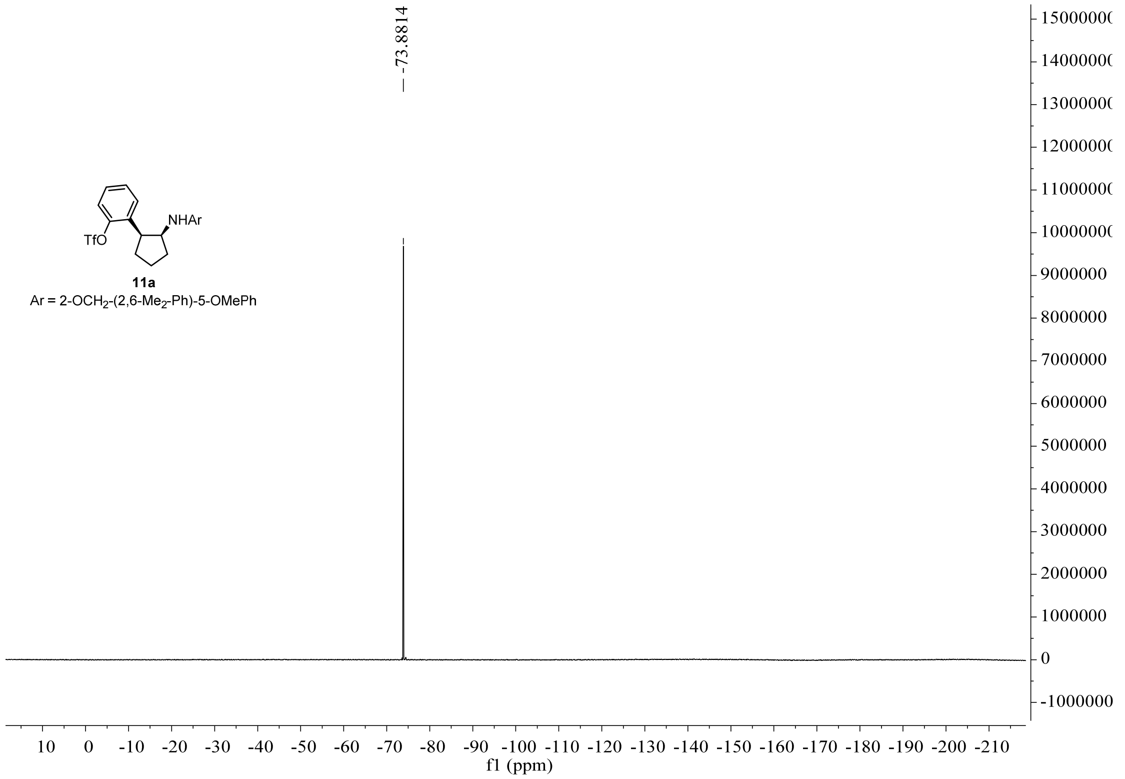
-73.8814
NHAr
TfO
11a
Ar = 2-OCH2-(2,6-Me2-Ph)-5-OMePh
f1 (ppm)

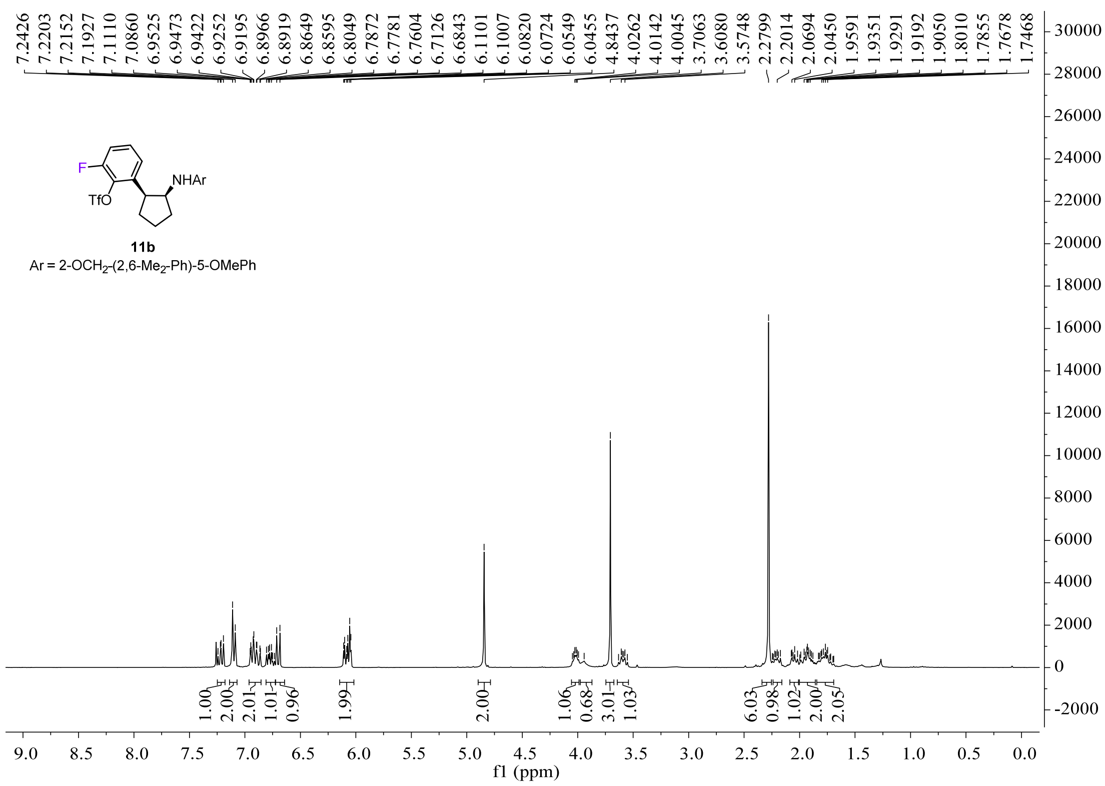
7.2426
7.2203
7.2152
7.1927
7.1110
7.0860
6.9525
6.9473
6.9422
6.9252
6.9195
6.8966
6.8919
6.8649
6.8595
6.8049
6.7872
6.7781
6.7604
6.7126
6.6843
6.1101
6.1007
6.0820
6.0724
6.0549
6.0455
4.8437
4.0262
4.0142
4.0045
3.7063
3.6080
3.5748
2.2799
2.2014
2.0694
2.0450
1.9591
1.9351
1.9291
1.9192
1.9050
1.8010
1.7855
1.7678
1.7468
F
TfO
NHAr
11b
Ar = 2-OCH2-(2,6-Me2-Ph)-5-OMePh
1.00
2.00
2.01
1.01
0.96
1.99
2.00
1.06
0.68
3.01
1.03
6.03
0.98
1.02
2.00
2.05
f1 (ppm)

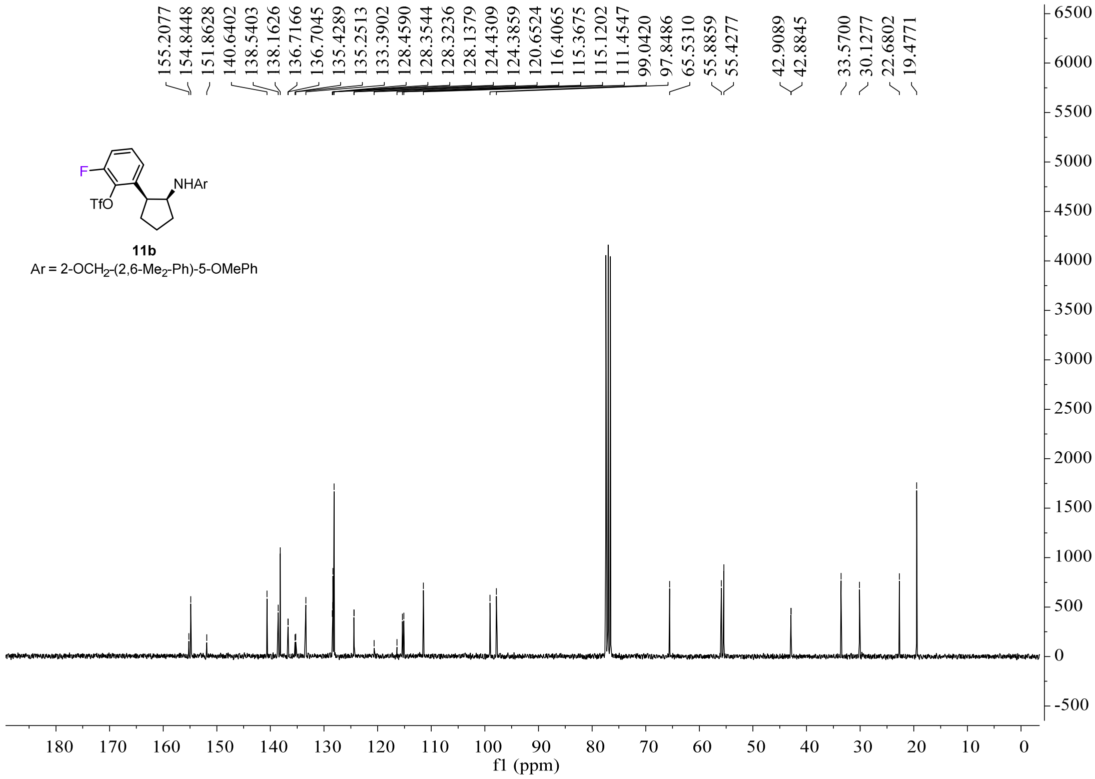
155.2077
154.8448
151.8628
140.6402
138.5403
138.1626
136.7166
136.7045
135.4289
135.2513
133.3902
128.4590
128.3544
128.3236
128.1379
124.4309
124.3859
120.6524
116.4065
115.3675
115.1202
111.4547
99.0420
97.8486
65.5310
55.8859
55.4277
42.9089
42.8845
33.5700
30.1277
22.6802
19.4771
F
TfO
NHAr
11b
Ar = 2-OCH2-(2,6-Me2-Ph)-5-OMePh
f1 (ppm)

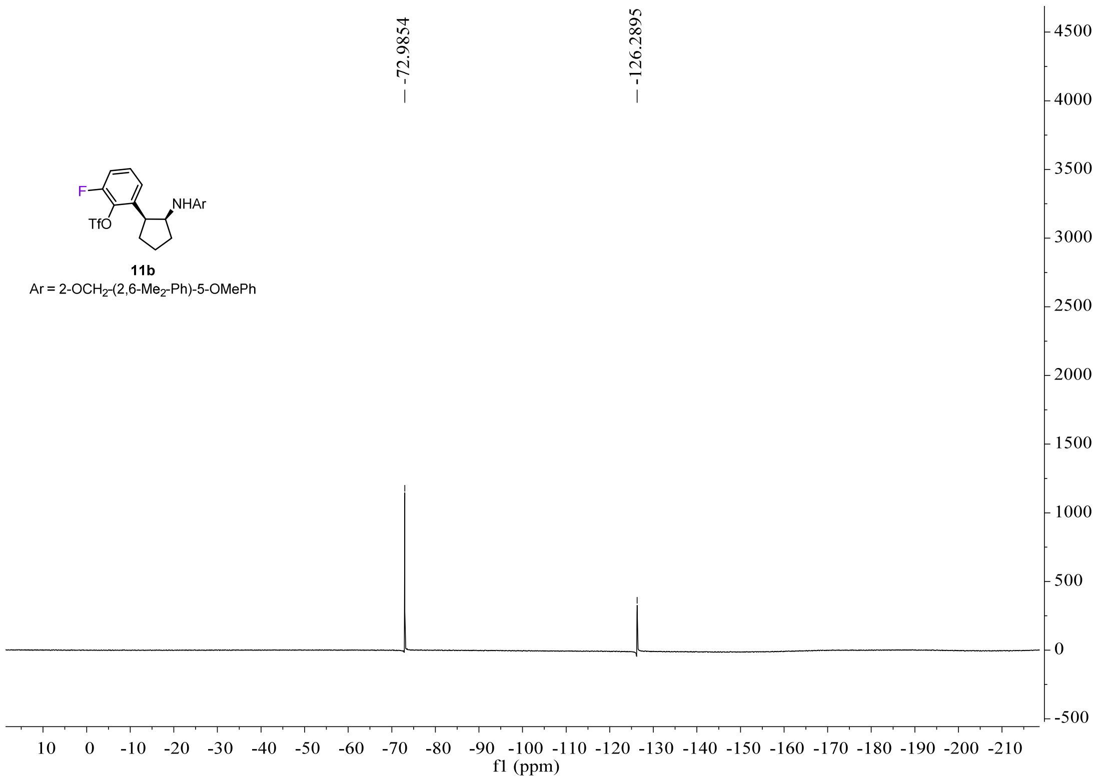
-72.9854
-126.2895
F
TfO
NHAr
11b
Ar = 2-OCH2-(2,6-Me2-Ph)-5-OMePh
f1 (ppm)

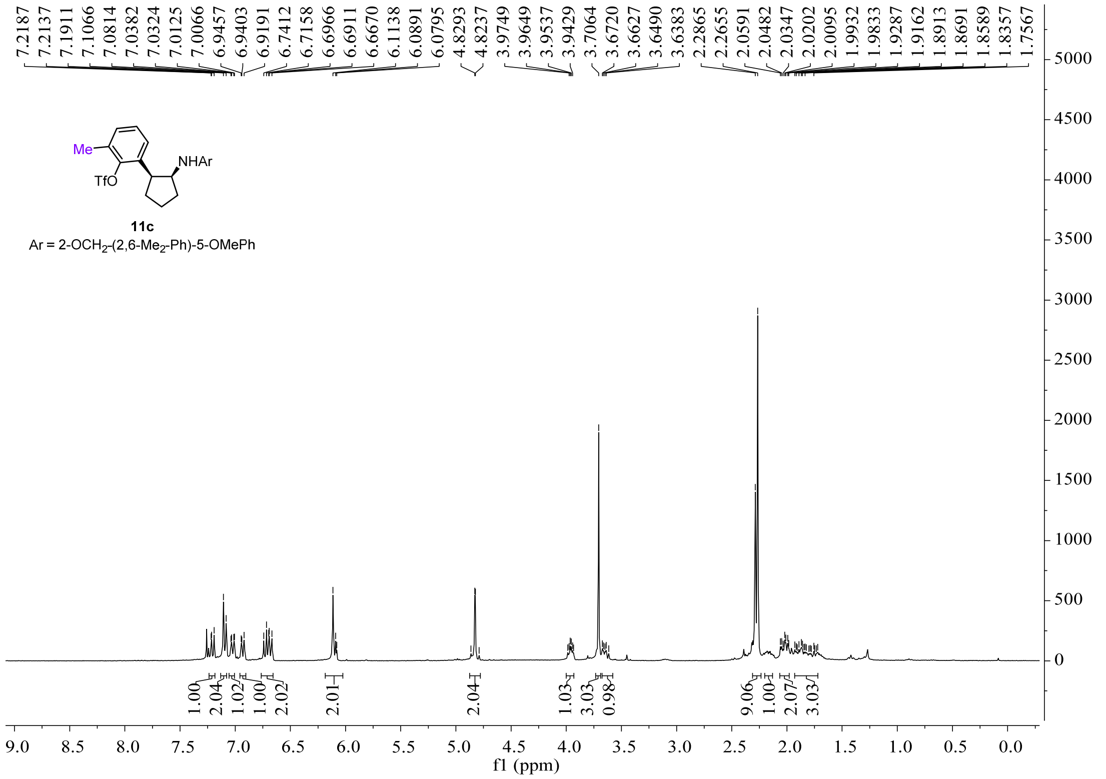
7.2187
7.2137
7.1911
7.1066
7.0814
7.0382
7.0324
7.0125
7.0066
6.9457
6.9403
6.9191
6.7412
6.7158
6.6966
6.6911
6.6670
6.1138
6.0891
6.0795
4.8293
4.8237
3.9749
3.9649
3.9537
3.9429
3.7064
3.6720
3.6627
3.6490
3.6383
2.2865
2.2655
2.0591
2.0482
2.0347
2.0202
2.0095
1.9932
1.9833
1.9287
1.9162
1.8913
1.8691
1.8589
1.8357
1.7567
Me
TfO
NHAr
11c
Ar = 2-OCH2-(2,6-Me2-Ph)-5-OMePh
1.00
2.04
1.02
1.00
2.02
2.01
2.04
1.03
3.03
0.98
9.06
1.00
2.07
3.03
f1 (ppm)

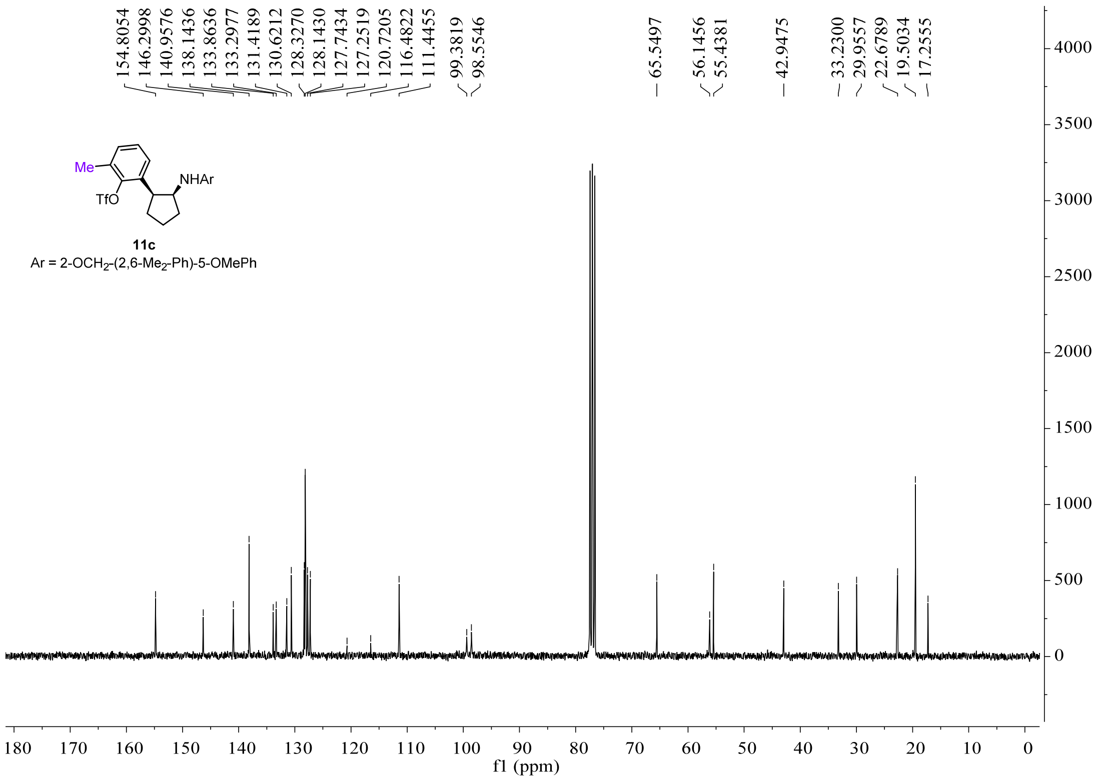
154.8054
146.2998
140.9576
138.1436
133.8636
133.2977
131.4189
130.6212
128.3270
128.1430
127.7434
127.2519
120.7205
116.4822
111.4455
99.3819
98.5546
65.5497
56.1456
55.4381
42.9475
33.2300
29.9557
22.6789
19.5034
17.2555
Me
NHAr
TfO
11c
Ar = 2-OCH2-(2,6-Me2-Ph)-5-OMePh
f1 (ppm)

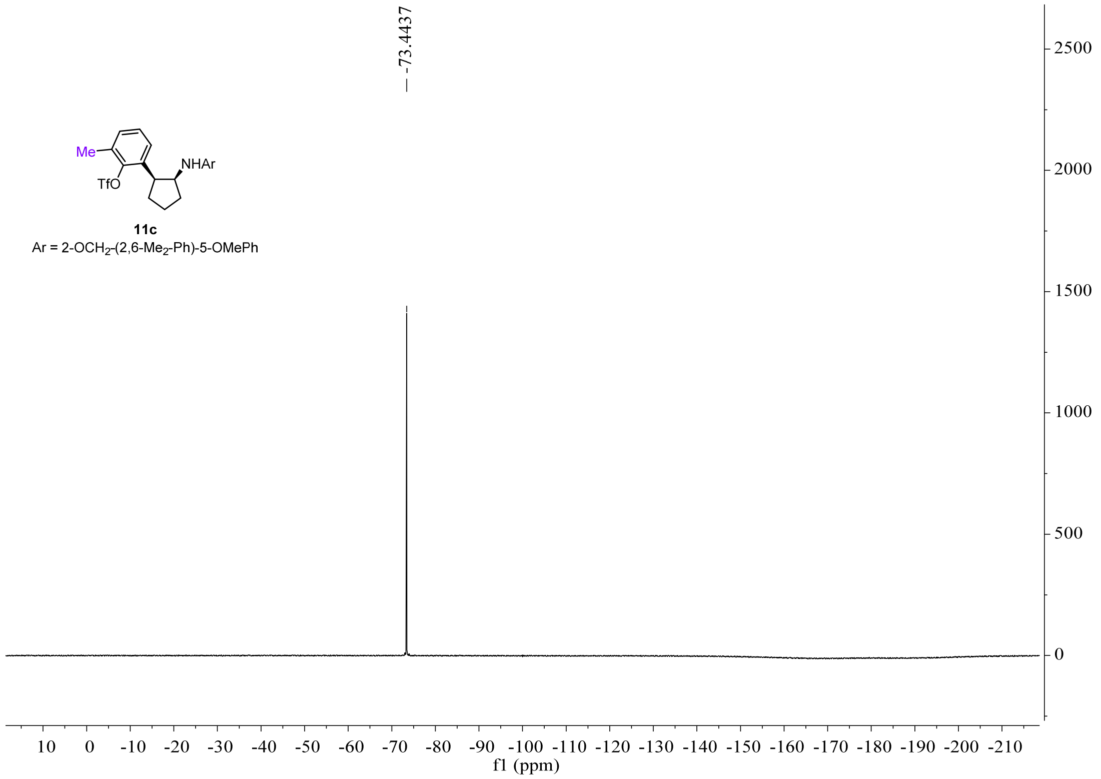
-73.4437
Me
NHAr
TfO
11c
Ar = 2-OCH2-(2,6-Me2-Ph)-5-OMePh
f1 (ppm)

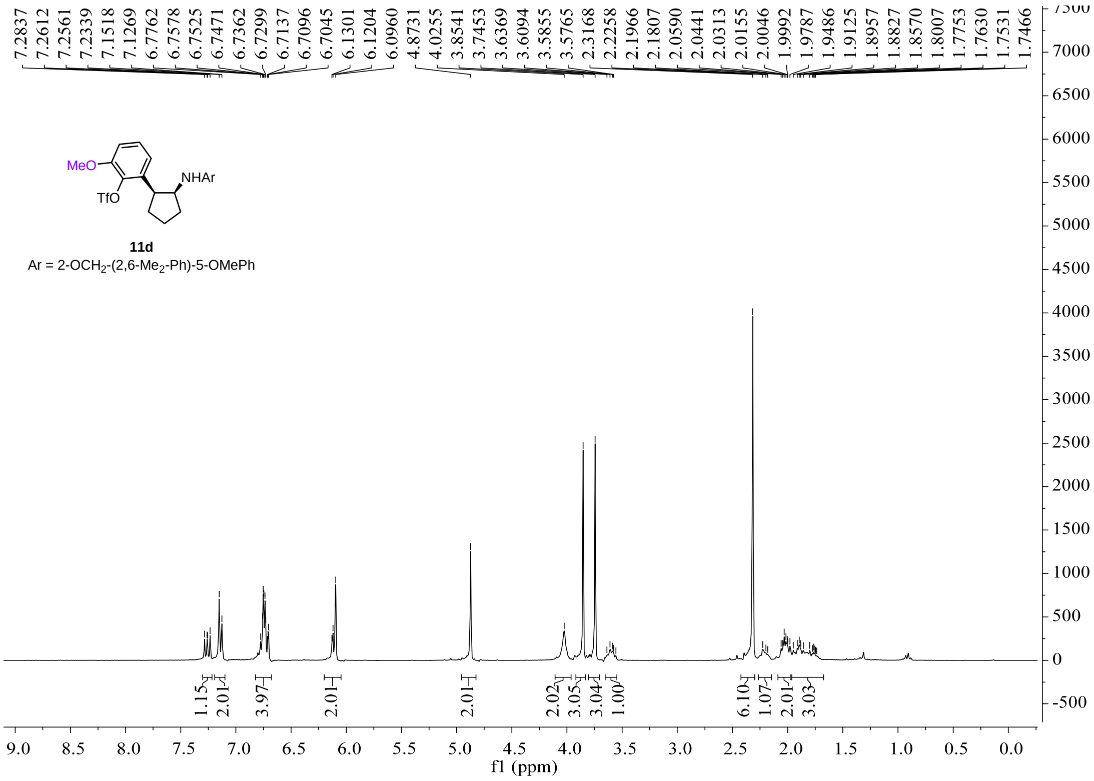
7.2837
7.2612
7.2561
7.2339
7.1518
7.1269
6.7762
6.7578
6.7525
6.7471
6.7362
6.7299
6.7137
6.7096
6.7045
6.1301
6.1204
6.0960
4.8731
4.0255
3.8541
3.7453
3.6369
3.6094
3.5855
3.5765
2.3168
2.2258
2.1966
2.1807
2.0590
2.0441
2.0313
2.0155
2.0046
1.9992
1.9787
1.9486
1.9125
1.8957
1.8827
1.8570
1.8007
1.7753
1.7630
1.7531
1.7466
MeO
NHAr
TfO
11d
Ar = 2-OCH2-(2,6-Me2-Ph)-5-OMePh
1.15
2.01
3.97
2.01
2.01
2.02
3.05
3.04
1.00
6.10
1.07
2.01
3.03
f1 (ppm)

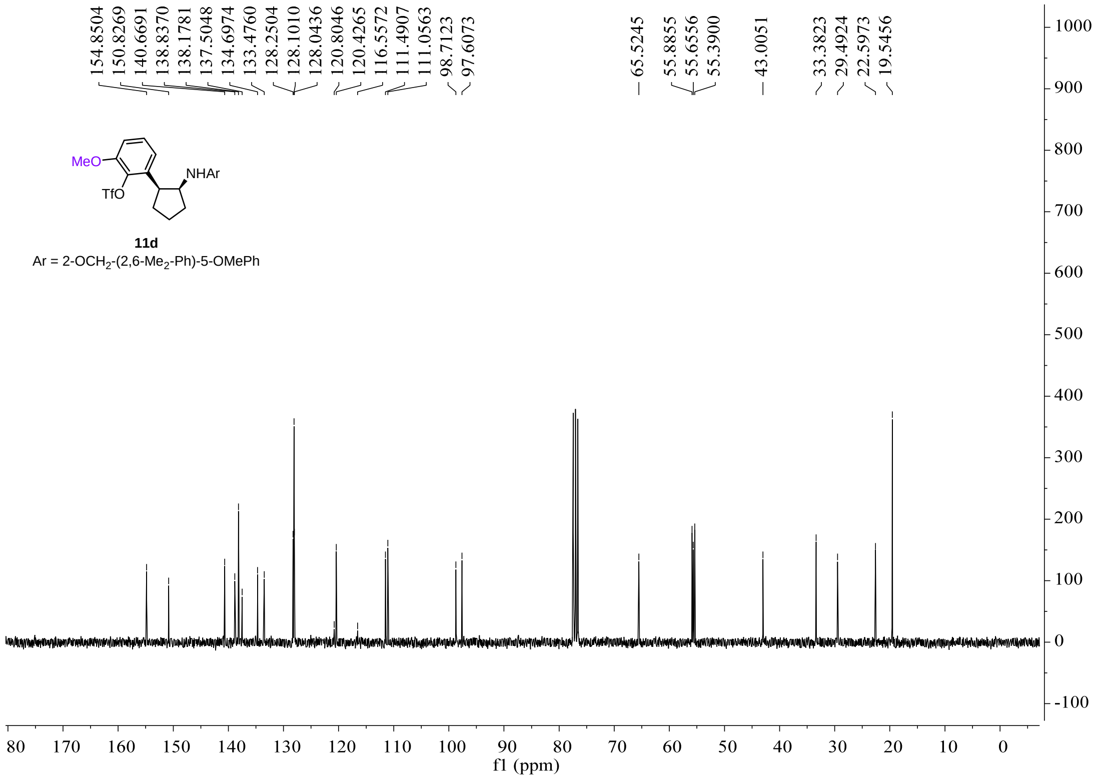
154.8504
150.8269
140.6691
138.8370
138.1781
137.5048
134.6974
133.4760
128.2504
128.1010
128.0436
120.8046
120.4265
116.5572
111.4907
111.0563
98.7123
97.6073
65.5245
55.8855
55.6556
55.3900
43.0051
33.3823
29.4924
22.5973
19.5456
MeO
NHAr
TfO
11d
Ar = 2-OCH2-(2,6-Me2-Ph)-5-OMePh
f1 (ppm)

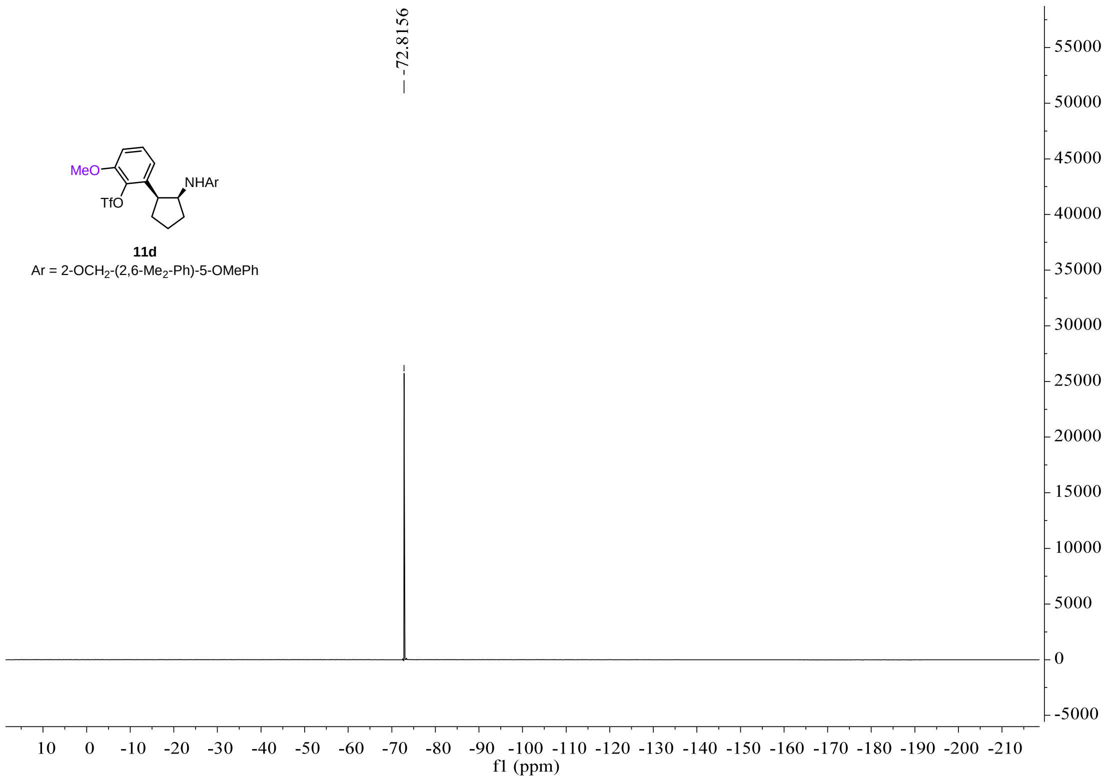
-72.8156
MeO
TfO
NHAr
11d
Ar = 2-OCH2-(2,6-Me2-Ph)-5-OMePh
f1 (ppm)

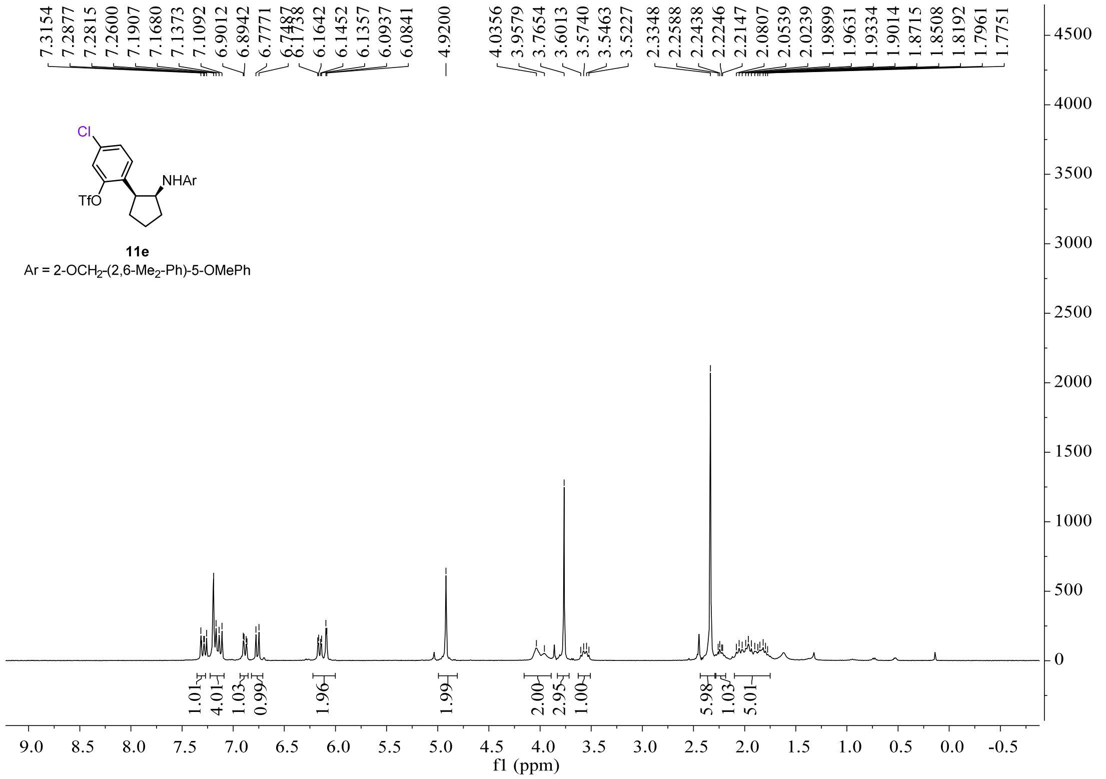
7.3154
7.2877
7.2815
7.2600
7.1907
7.1680
7.1373
7.1092
6.9012
6.8942
6.7771
6.7487
6.1758
6.1642
6.1452
6.1357
6.0937
6.0841
4.9200
4.0356
3.9579
3.7654
3.6013
3.5740
3.5463
3.5227
2.3348
2.2588
2.2438
2.2246
2.2147
2.0807
2.0539
2.0239
1.9899
1.9631
1.9334
1.9014
1.8715
1.8508
1.8192
1.7961
1.7751
Cl
TfO
NHAr
11e
Ar = 2-OCH2-(2,6-Me2-Ph)-5-OMePh
1.01
4.01
1.03
0.99
1.96
1.99
2.00
2.95
1.00
5.98
1.03
5.01
f1 (ppm)

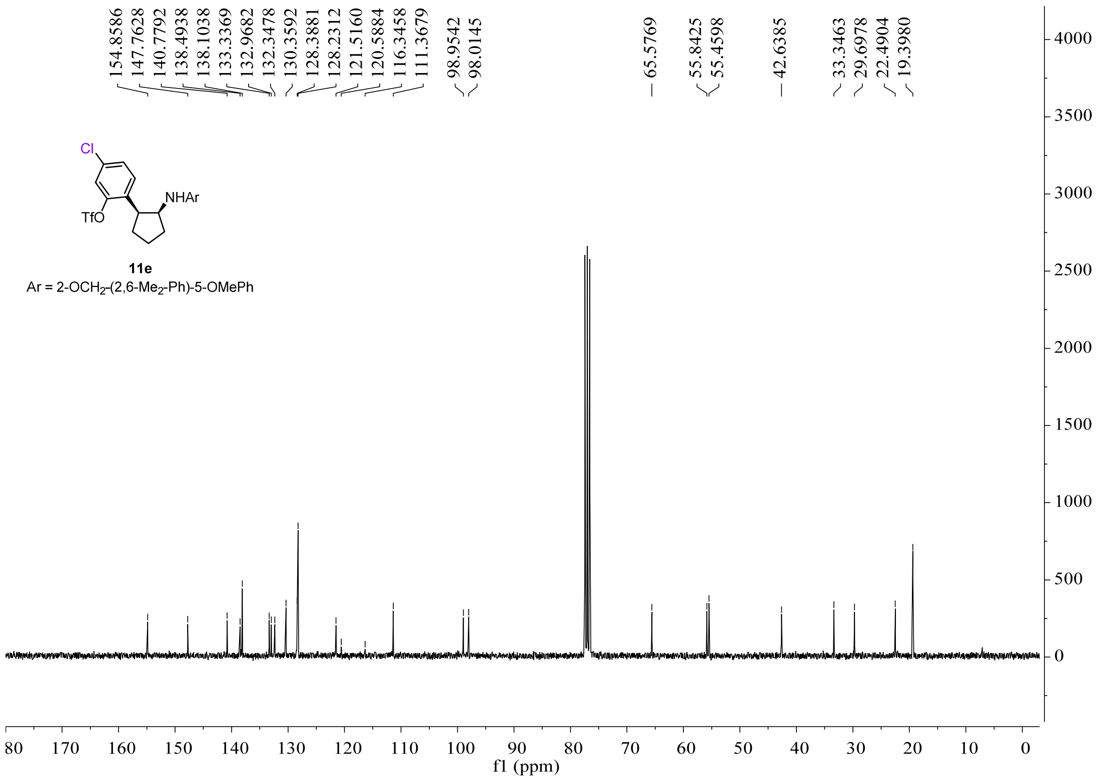
154.8586
147.7628
140.7792
138.4938
138.1038
133.3369
132.9682
132.3478
130.3592
128.3881
128.2312
121.5160
120.5884
116.3458
111.3679
98.9542
98.0145
65.5769
55.8425
55.4598
42.6385
33.3463
29.6978
22.4904
19.3980
Cl
NHAr
TfO
11e
Ar = 2-OCH2-(2,6-Me2-Ph)-5-OMePh
f1 (ppm)

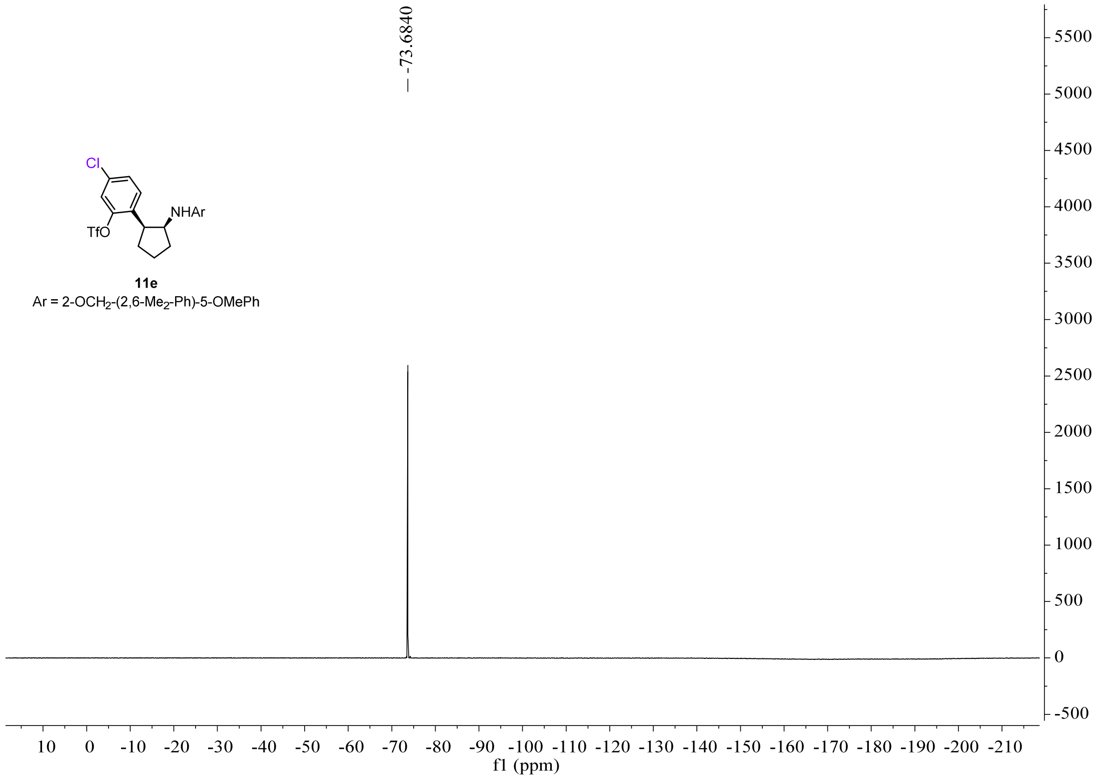
-73.6840
Cl
NHAr
TfO
11e
Ar = 2-OCH2-(2,6-Me2-Ph)-5-OMePh
f1 (ppm)

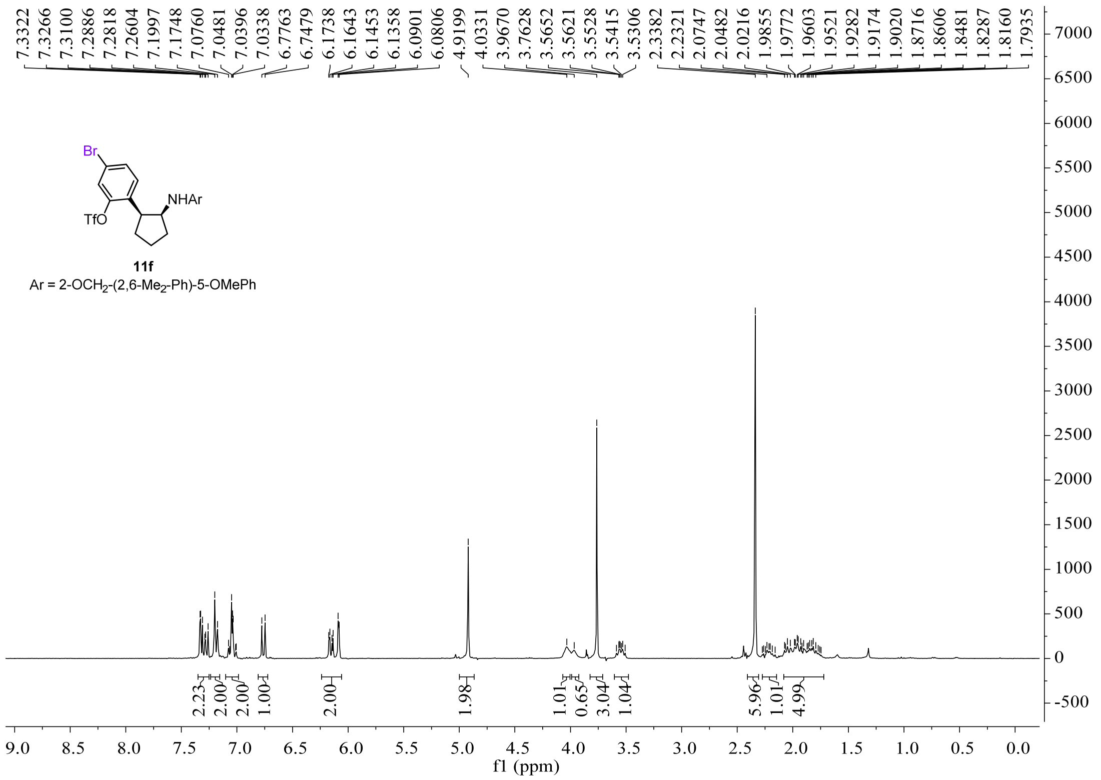
7.3322
7.3266
7.3100
7.2886
7.2818
7.2604
7.1997
7.1748
7.0760
7.0481
7.0396
7.0338
6.7763
6.7479
6.1738
6.1643
6.1453
6.1358
6.0901
6.0806
4.9199
4.0331
3.9670
3.7628
3.5652
3.5621
3.5528
3.5415
3.5306
2.3382
2.2321
2.0747
2.0482
2.0216
1.9855
1.9772
1.9603
1.9521
1.9282
1.9174
1.9020
1.8716
1.8606
1.8481
1.8287
1.8160
1.7935
Br
TfO
NHAr
11f
Ar = 2-OCH2-(2,6-Me2-Ph)-5-OMePh
2.23
2.00
2.00
1.00
2.00
1.98
1.01
0.65
3.04
1.04
5.96
1.01
4.99
f1 (ppm)

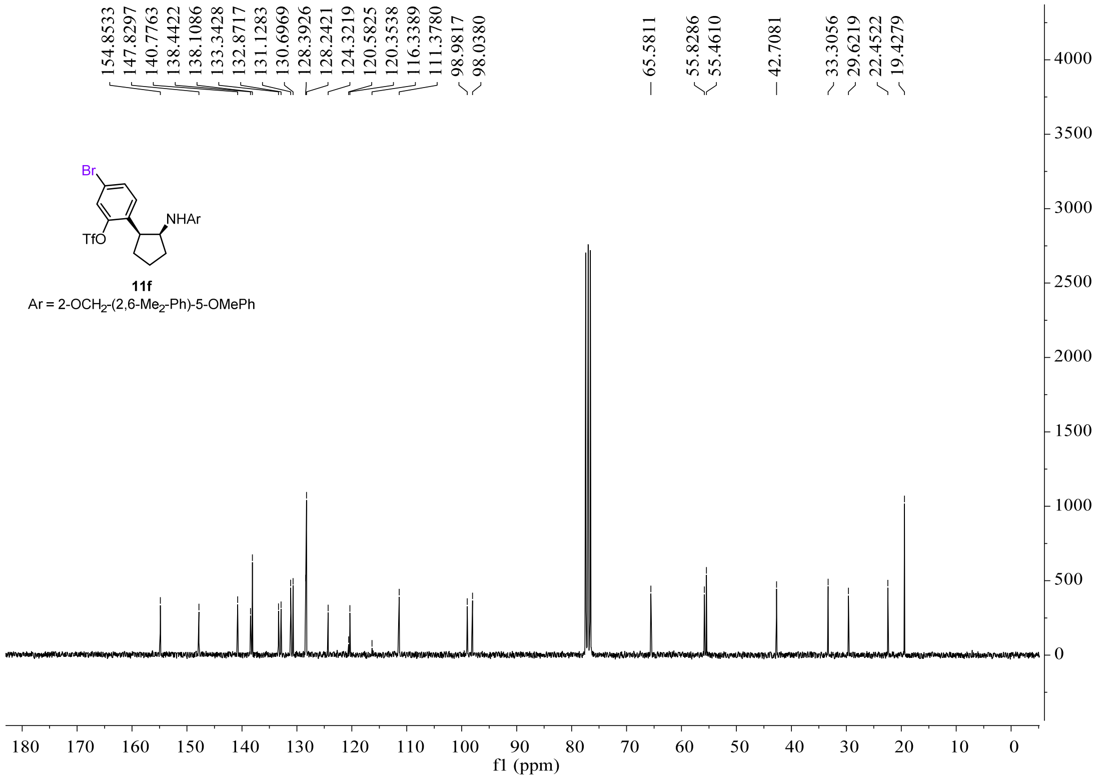
154.8533
147.8297
140.7763
138.4422
138.1086
133.3428
132.8717
131.1283
130.6969
128.3926
128.2421
124.3219
120.5825
120.3538
116.3389
111.3780
98.9817
98.0380
65.5811
55.8286
55.4610
42.7081
33.3056
29.6219
22.4522
19.4279
Br
TfO
NHAr
11f
Ar = 2-OCH2-(2,6-Me2-Ph)-5-OMePh
f1 (ppm)

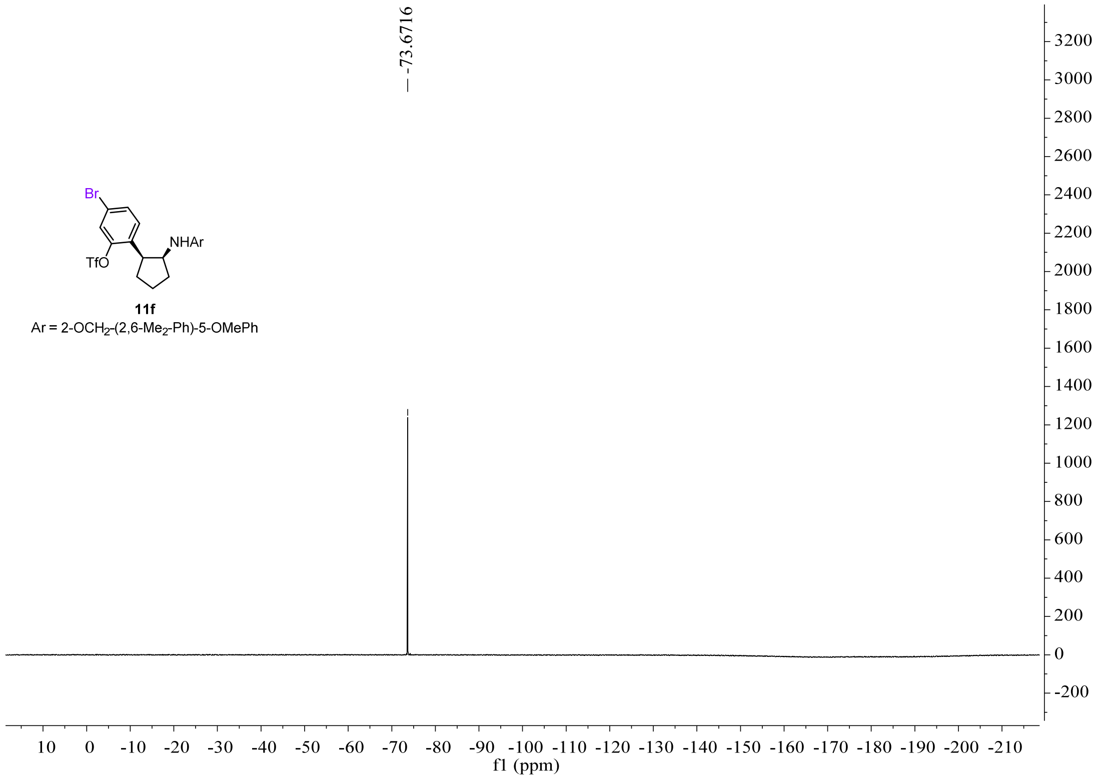

11f
Ar = 2-OCH$_2$-(2,6-Me$_2$-Ph)-5-OMePh

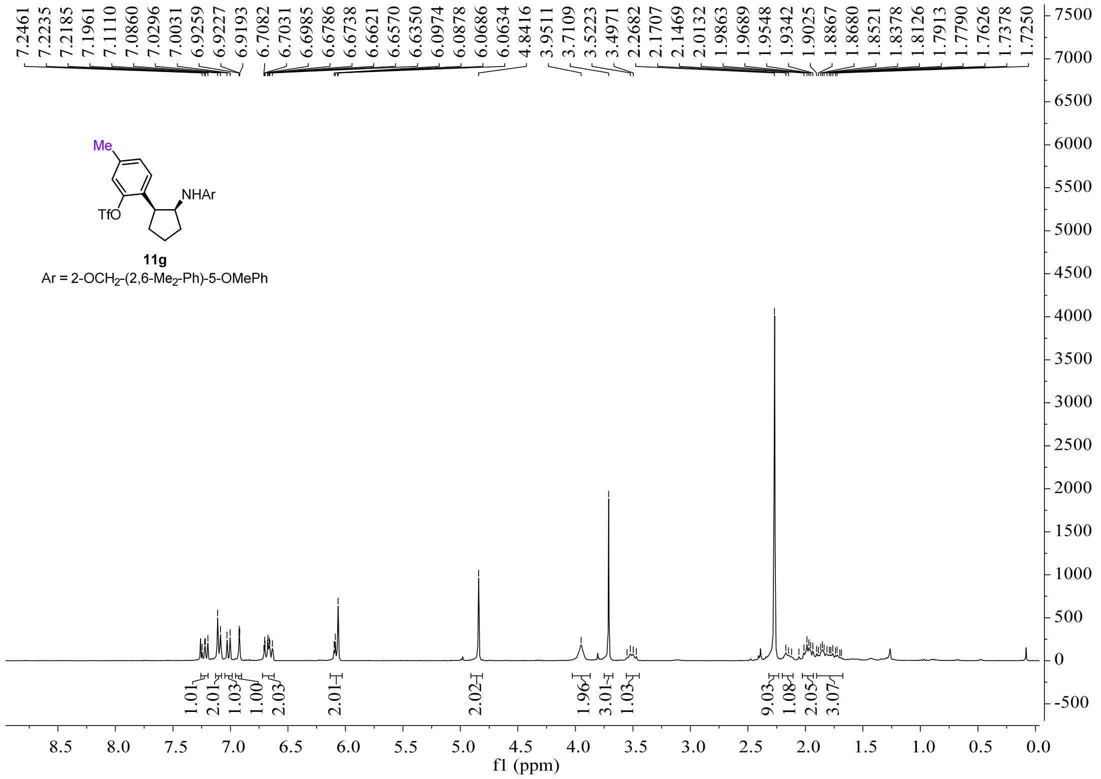

11g
Ar = 2-OCH$_2$-(2,6-Me$_2$-Ph)-5-OMePh

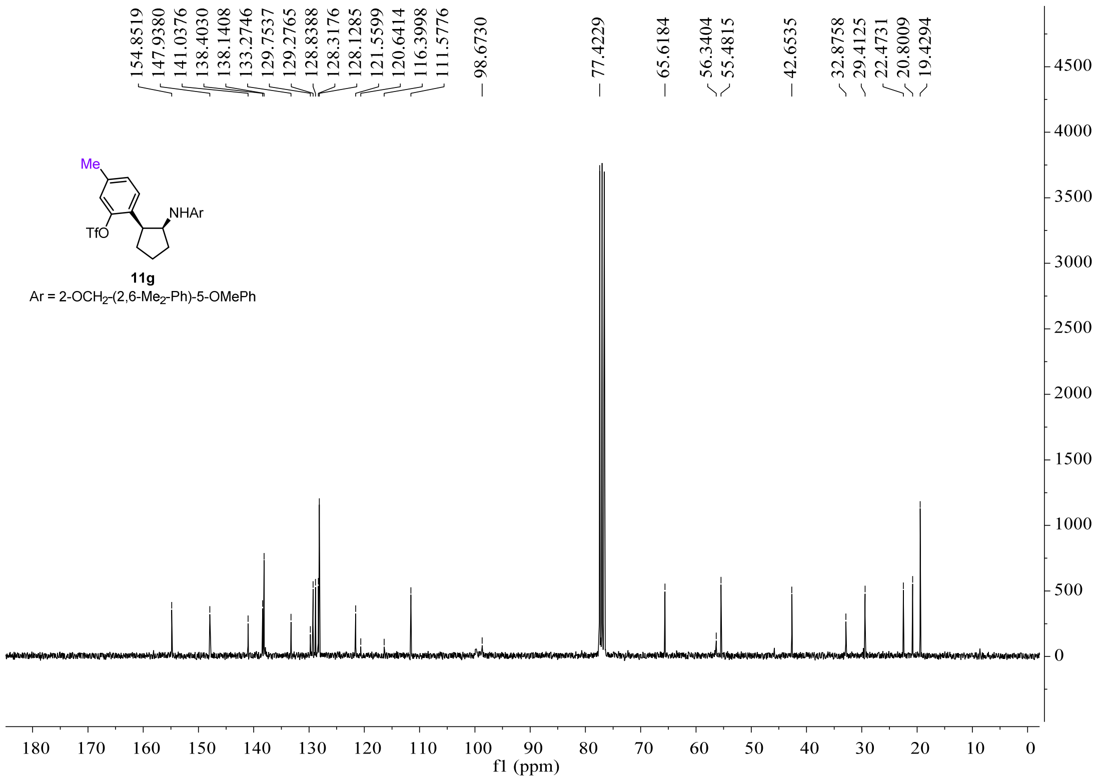
154.8519
147.9380
141.0376
138.4030
138.1408
133.2746
129.7537
129.2765
128.8388
128.3176
128.1285
121.5599
120.6414
116.3998
111.5776
98.6730
77.4229
65.6184
56.3404
55.4815
42.6535
32.8758
29.4125
22.4731
20.8009
19.4294
Me
NHAr
TfO
11g
Ar = 2-OCH2-(2,6-Me2-Ph)-5-OMePh
f1 (ppm)

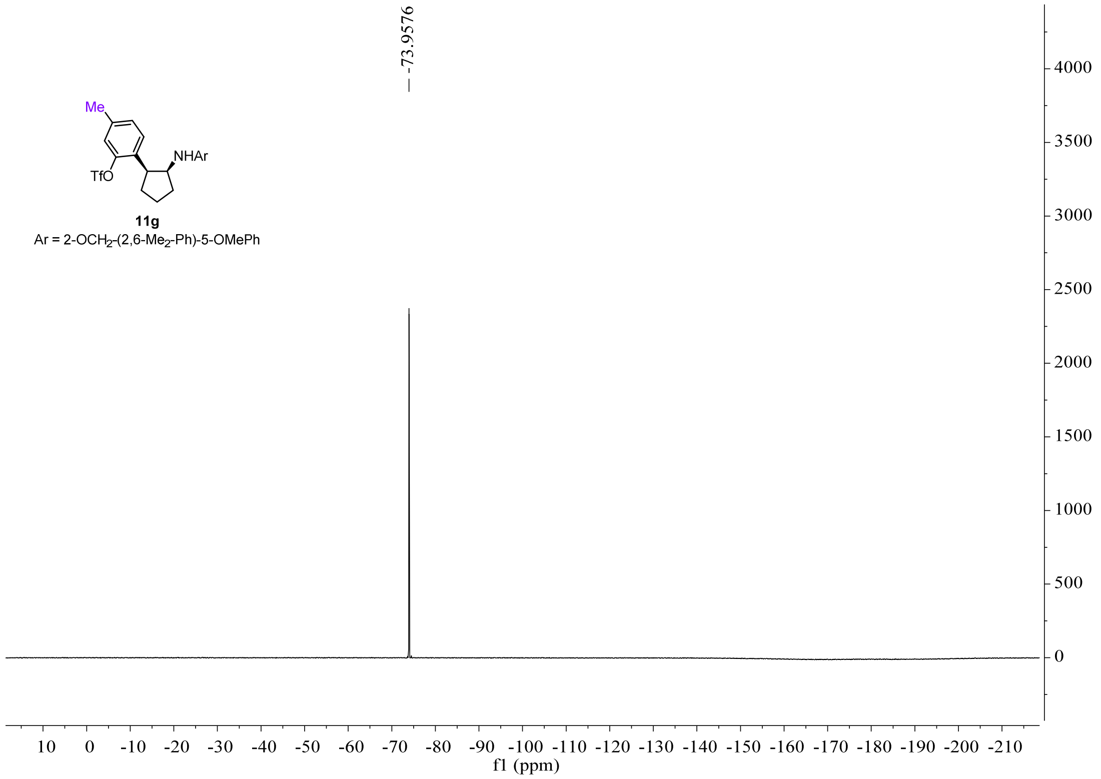
-73.9576
Me
NHAr
TfO
11g
Ar = 2-OCH2-(2,6-Me2-Ph)-5-OMePh
f1 (ppm)

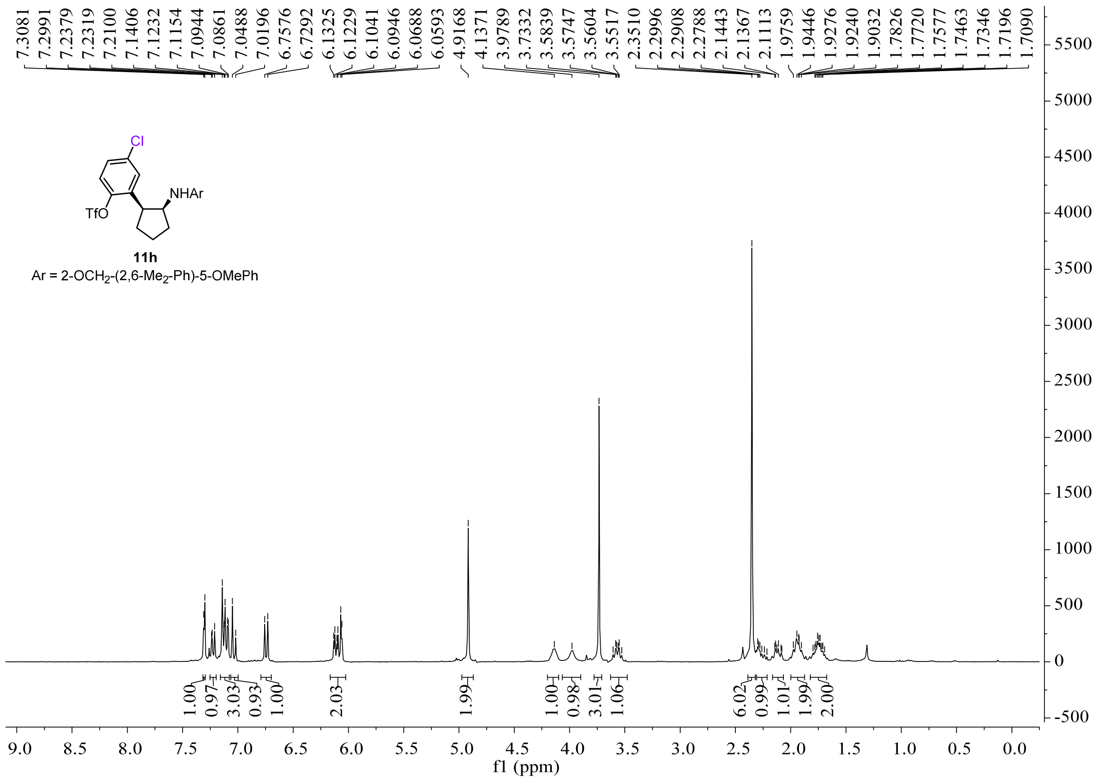
7.3081
7.2991
7.2379
7.2319
7.2100
7.1406
7.1232
7.1154
7.0944
7.0861
7.0488
7.0196
6.7576
6.7292
6.1325
6.1229
6.1041
6.0946
6.0688
6.0593
4.9168
4.1371
3.9789
3.7332
3.5839
3.5747
3.5604
3.5517
2.3510
2.2996
2.2908
2.2788
2.1443
2.1367
2.1113
1.9759
1.9446
1.9276
1.9240
1.9032
1.7826
1.7720
1.7577
1.7463
1.7346
1.7196
1.7090
Cl
NHAr
TfO
11h
Ar = 2-OCH2-(2,6-Me2-Ph)-5-OMePh
1.00
0.97
3.03
0.93
1.00
2.03
1.99
1.00
0.98
3.01
1.06
6.02
0.99
1.01
1.99
2.00
f1 (ppm)

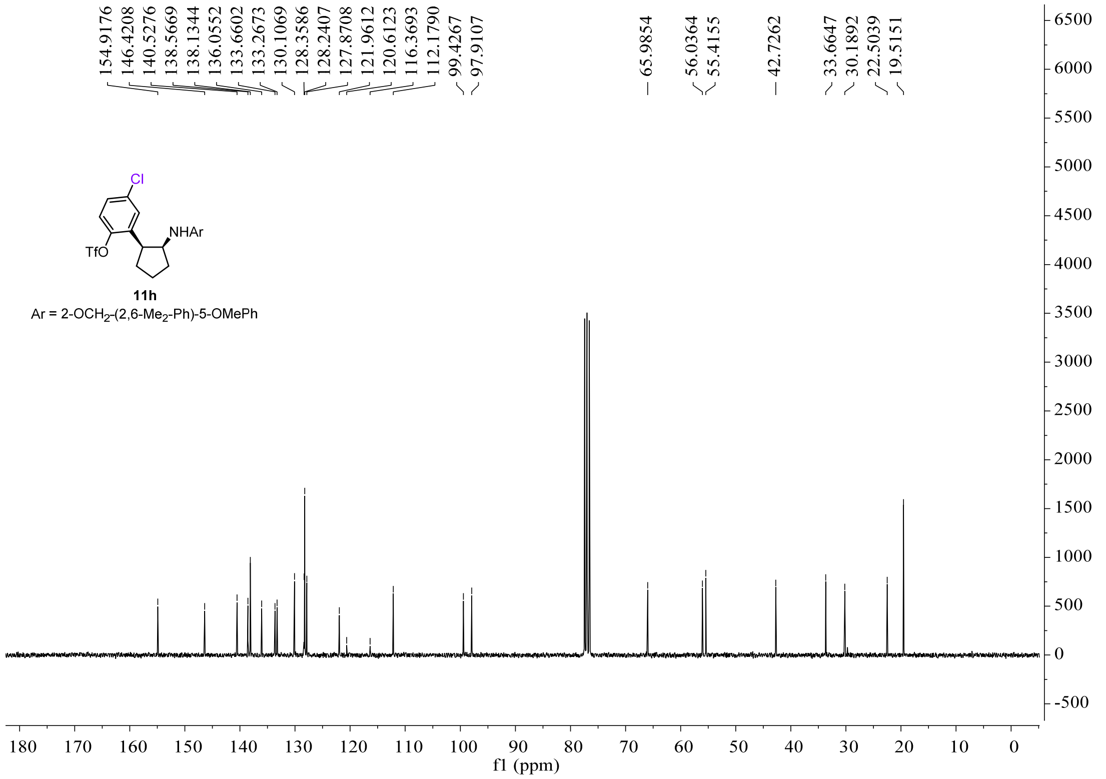
154.9176
146.4208
140.5276
138.5669
138.1344
136.0552
133.6602
133.2673
130.1069
128.3586
128.2407
127.8708
121.9612
120.6123
116.3693
112.1790
99.4267
97.9107
65.9854
56.0364
55.4155
42.7262
33.6647
30.1892
22.5039
19.5151
Cl
NHAr
TfO
11h
Ar = 2-OCH2-(2,6-Me2-Ph)-5-OMePh
f1 (ppm)

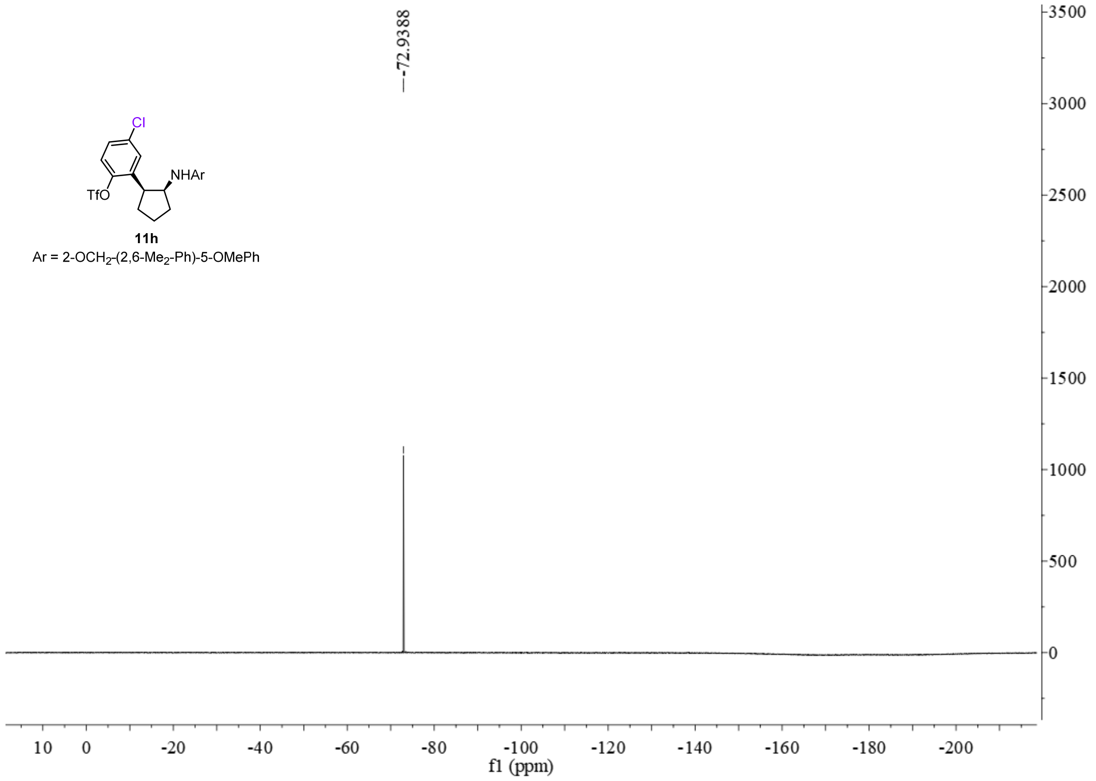

11h

Ar = 2-OCH$_2$-(2,6-Me$_2$-Ph)-5-OMePh

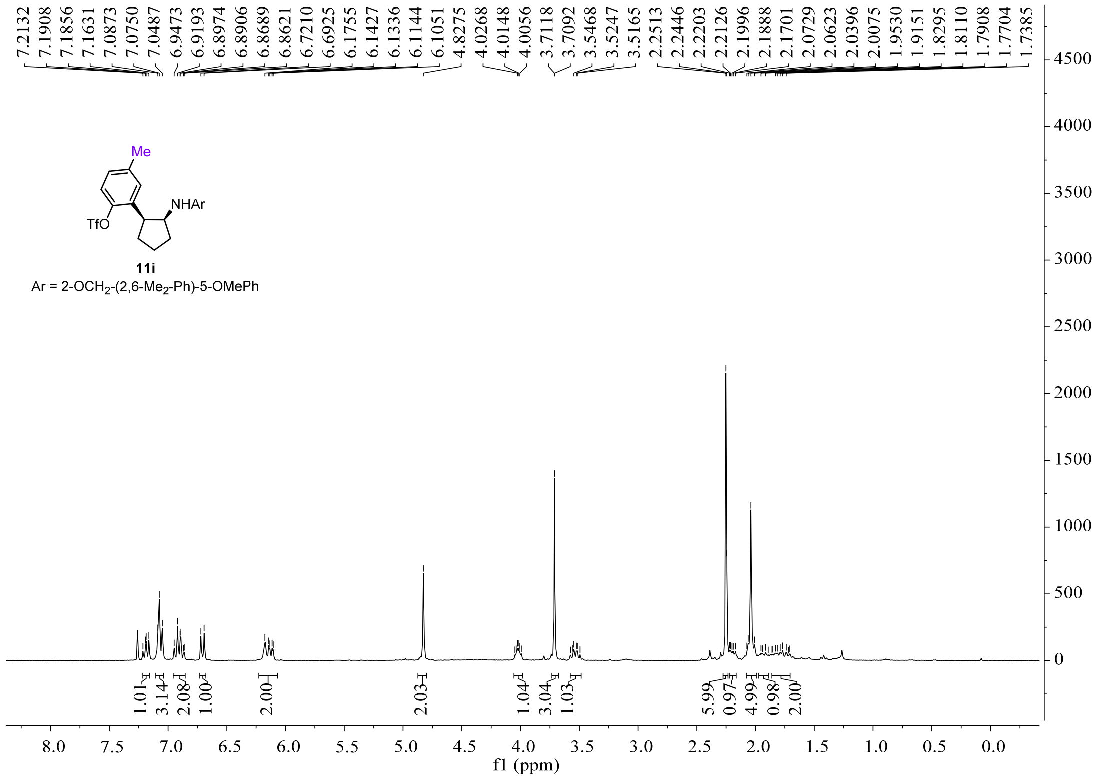

11i

Ar = 2-OCH$_2$-(2,6-Me$_2$-Ph)-5-OMePh

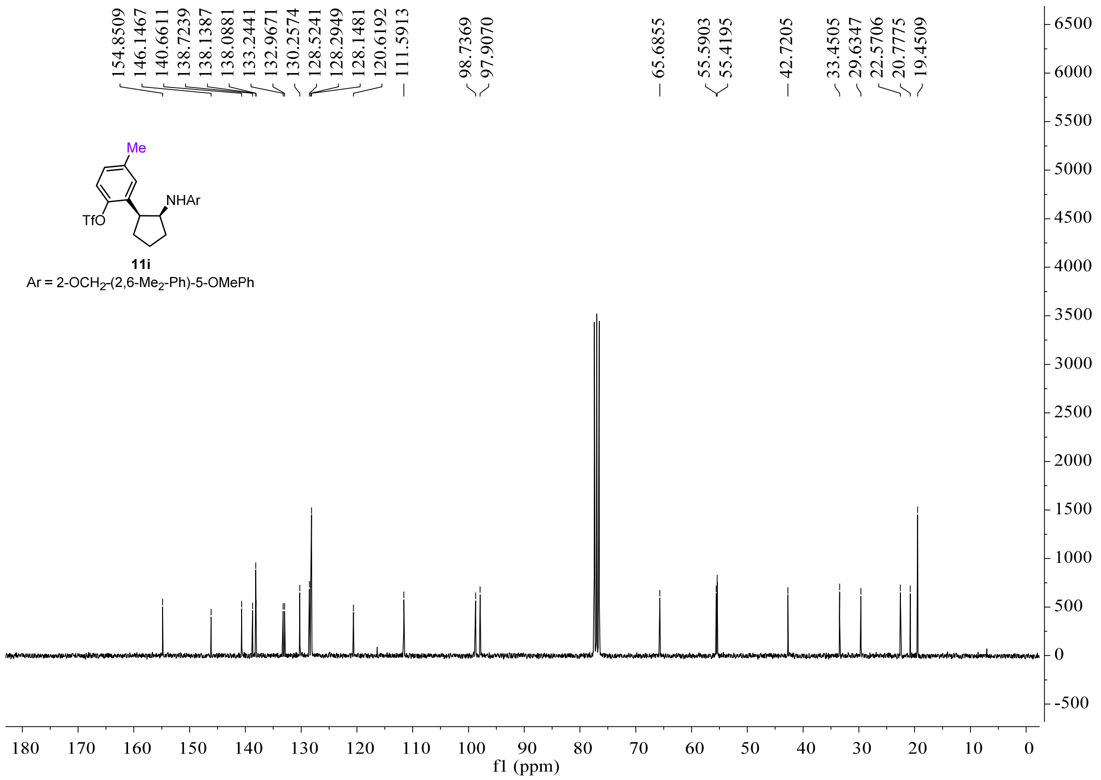

11i
Ar = 2-OCH$_2$-(2,6-Me$_2$-Ph)-5-OMePh

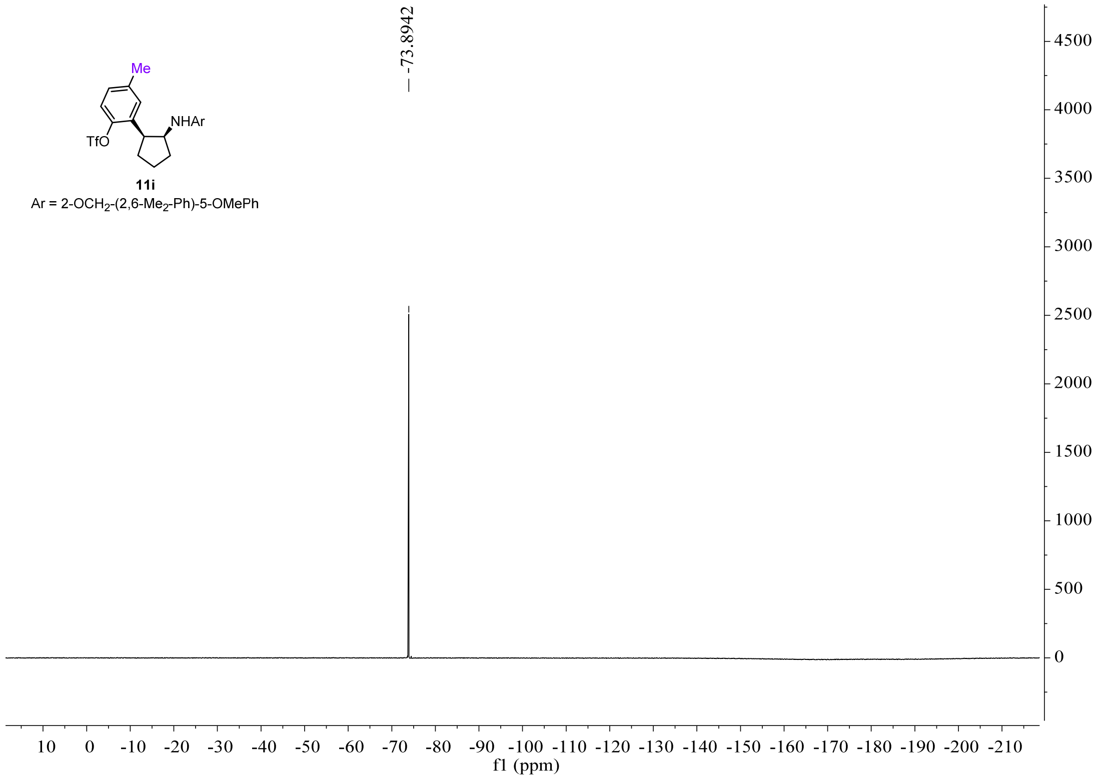

11i
Ar = 2-OCH$_2$-(2,6-Me$_2$-Ph)-5-OMePh

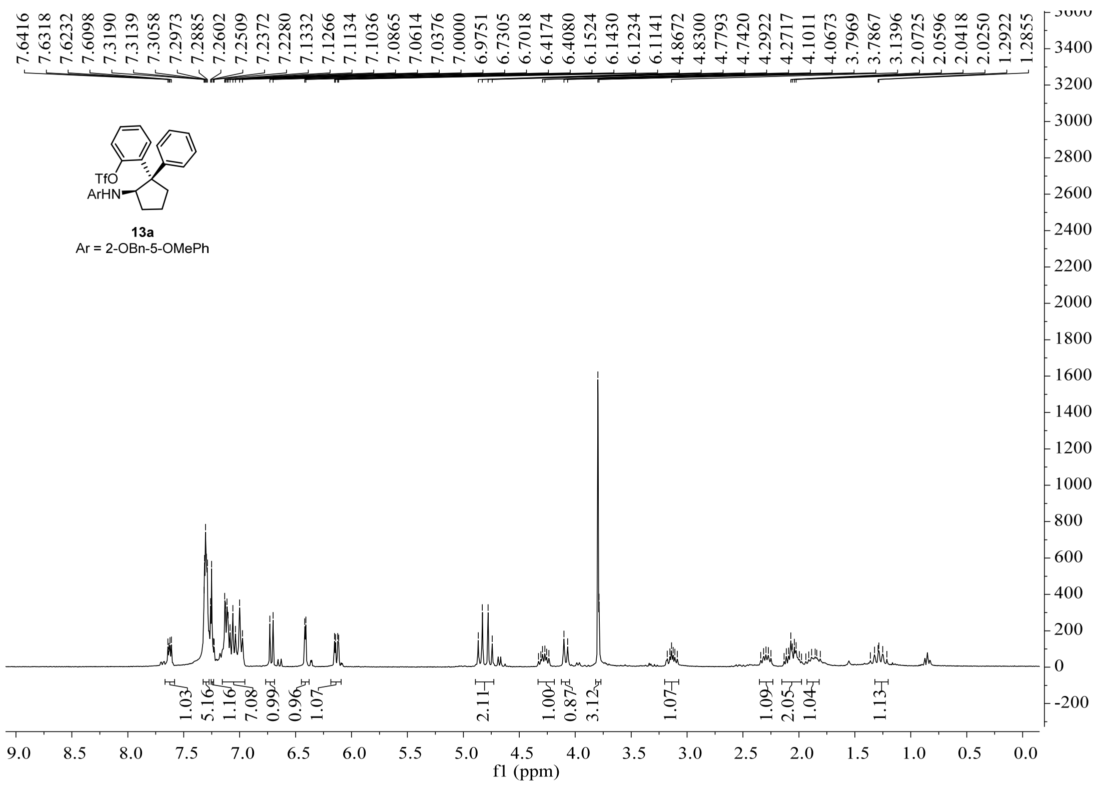
7.6416 7.6318 7.6232 7.6098 7.3190 7.3139 7.3058 7.2973 7.2885 7.2602 7.2509 7.2372 7.2280 7.1332 7.1266 7.1134 7.1036 7.0865 7.0614 7.0376 7.0000 6.9751 6.7305 6.7018 6.4174 6.4080 6.1524 6.1430 6.1234 6.1141 4.8672 4.8300 4.7793 4.7420 4.2922 4.2717 4.1011 4.0673 3.7969 3.7867 3.1396 2.0725 2.0596 2.0418 2.0250 1.2922 1.2855
TfO
ArHN
13a
Ar = 2-OBn-5-OMePh
1.03 5.16 1.16 7.08 0.99 0.96 1.07 2.11 1.00 0.87 3.12 1.07 1.09 2.05 1.04 1.13
f1 (ppm)

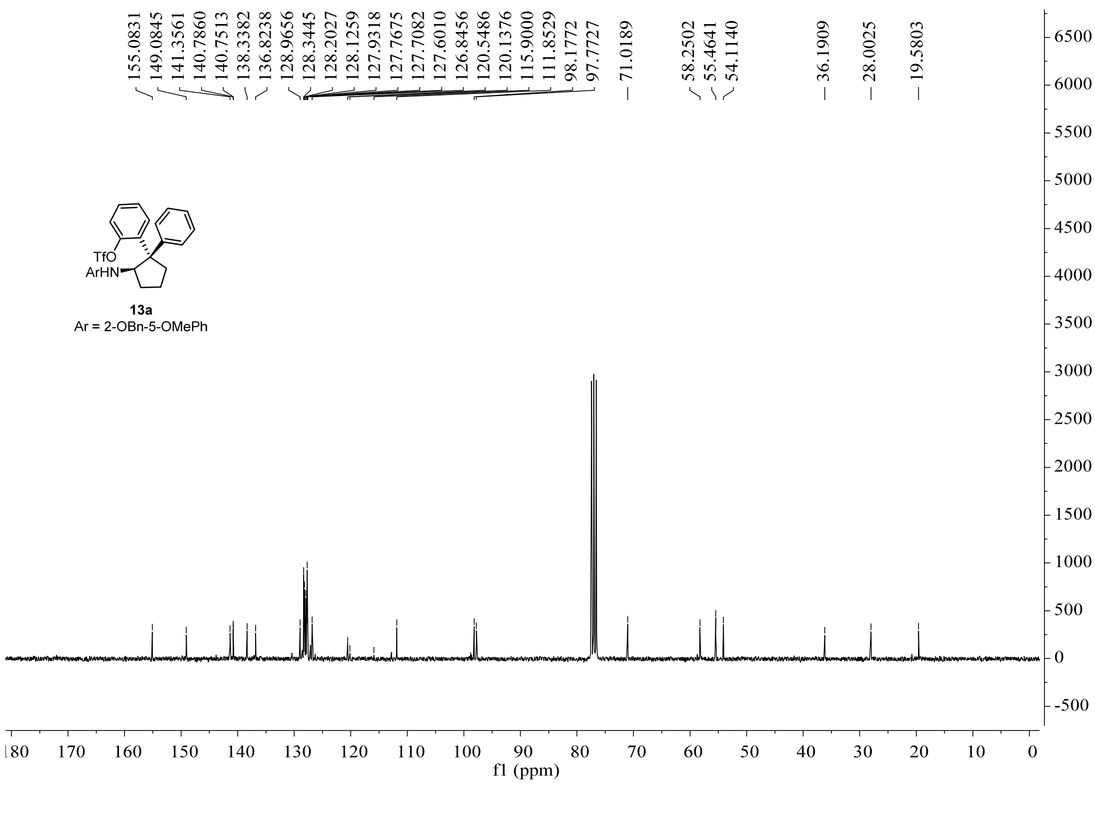
155.0831 149.0845 141.3561 140.7860 140.7513 138.3382 136.8238 128.9656 128.3445 128.2027 128.1259 127.9318 127.7675 127.7082 127.6010 126.8456 120.5486 120.1376 115.9000 111.8529 98.1772 97.7727 71.0189 58.2502 55.4641 54.1140 36.1909 28.0025 19.5803
TfO
ArHN
13a
Ar = 2-OBn-5-OMePh
f1 (ppm)

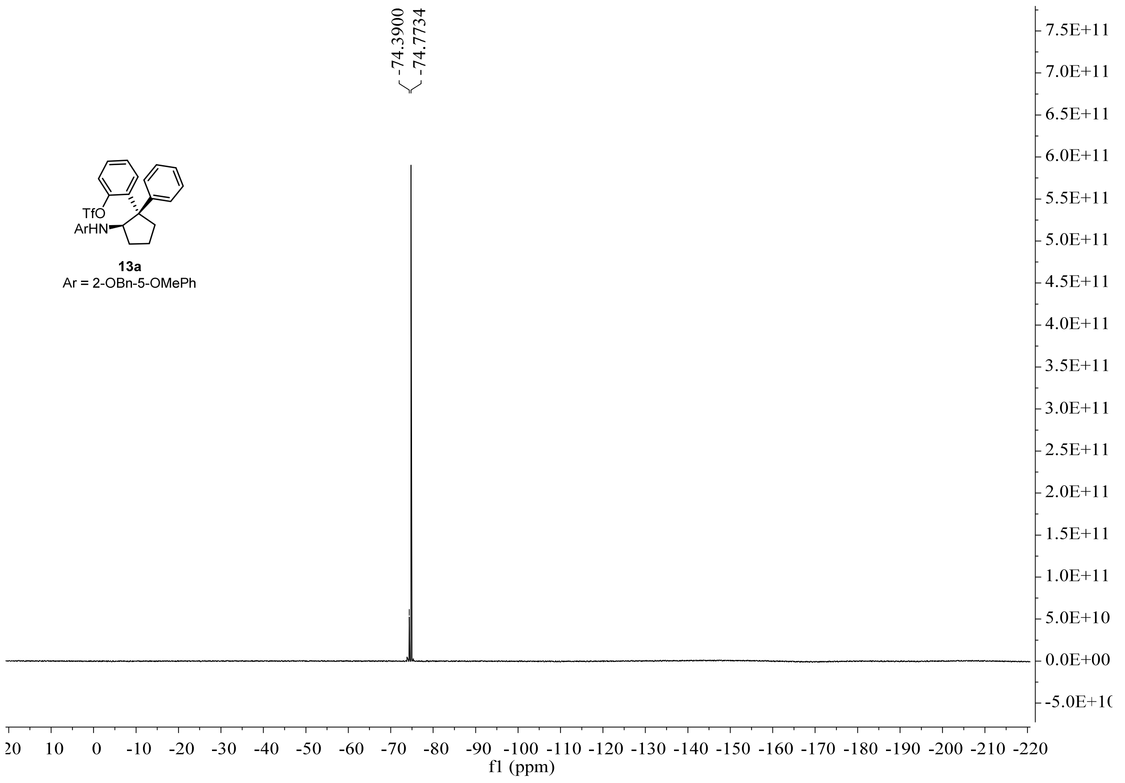
-74.3900
-74.7734
TfO
ArHN
13a
Ar = 2-OBn-5-OMePh
f1 (ppm)

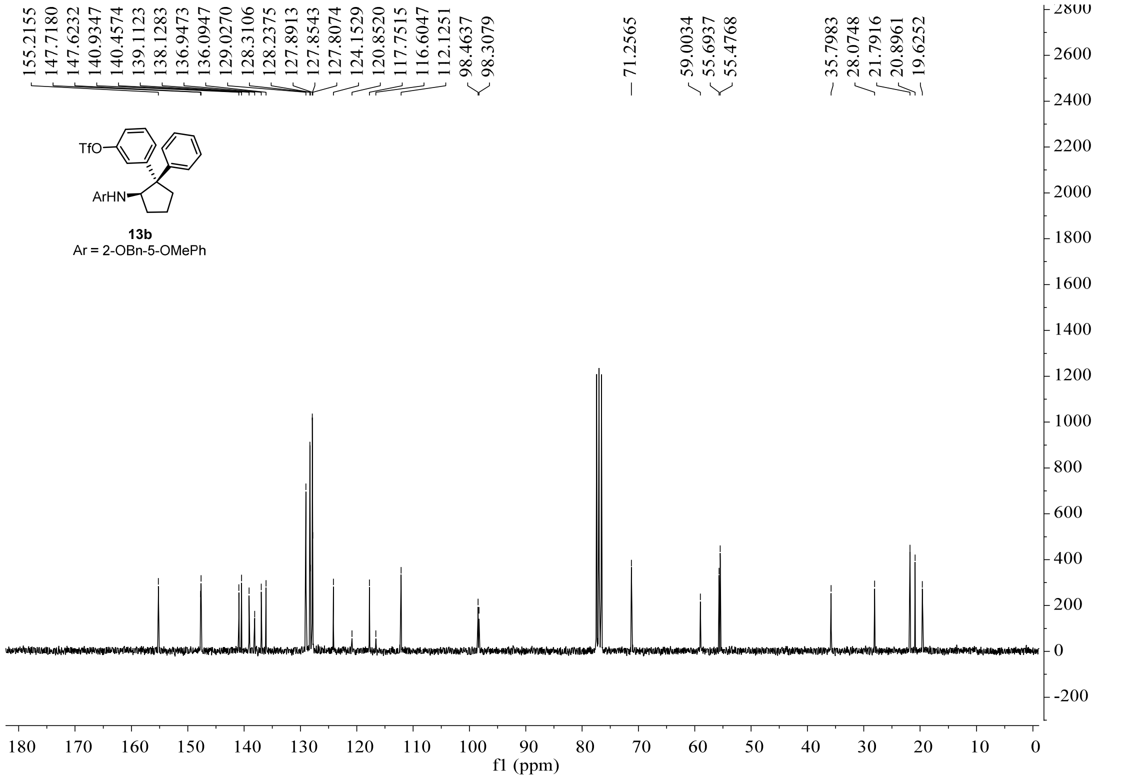
155.2155
147.7180
147.6232
140.9347
140.4574
139.1123
138.1283
136.9473
136.0947
129.0270
128.3106
128.2375
127.8913
127.8543
127.8074
124.1529
120.8520
117.7515
116.6047
112.1251
98.4637
98.3079
71.2565
59.0034
55.6937
55.4768
35.7983
28.0748
21.7916
20.8961
19.6252
TfO
ArHN
13b
Ar = 2-OBn-5-OMePh
f1 (ppm)

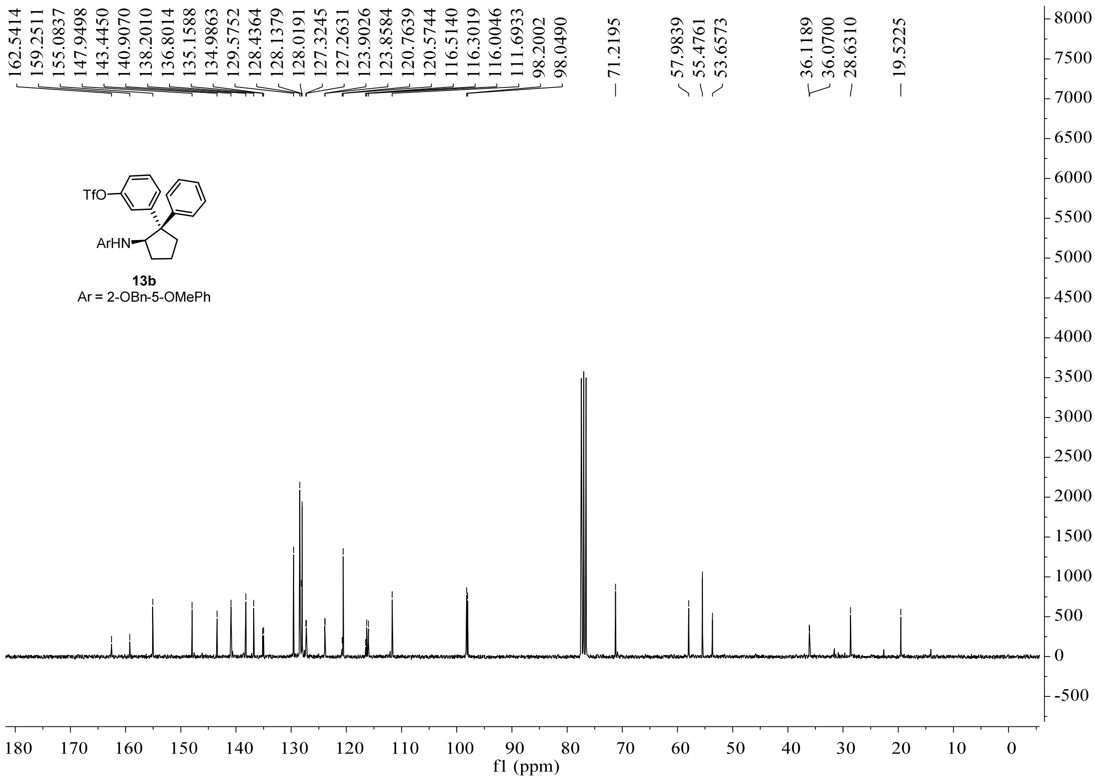
162.5414
159.2511
155.0837
147.9498
143.4450
140.9070
138.2010
136.8014
135.1588
134.9863
129.5752
128.4364
128.1379
128.0191
127.3245
127.2631
123.9026
123.8584
120.7639
120.5744
116.5140
116.3019
116.0046
111.6933
98.2002
98.0490
71.2195
57.9839
55.4761
53.6573
36.1189
36.0700
28.6310
19.5225
TfO
ArHN
13b
Ar = 2-OBn-5-OMePh
f1 (ppm)

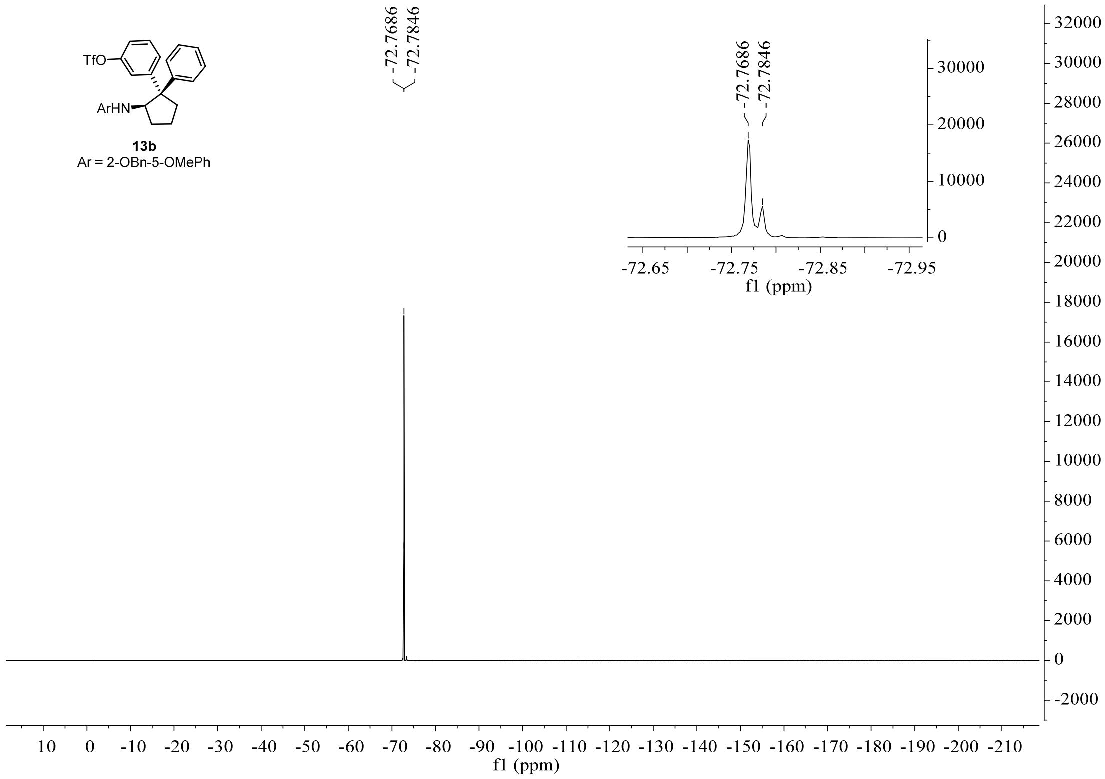
-72.7686
-72.7846
TfO
ArHN
13b
Ar = 2-OBn-5-OMePh
f1 (ppm)

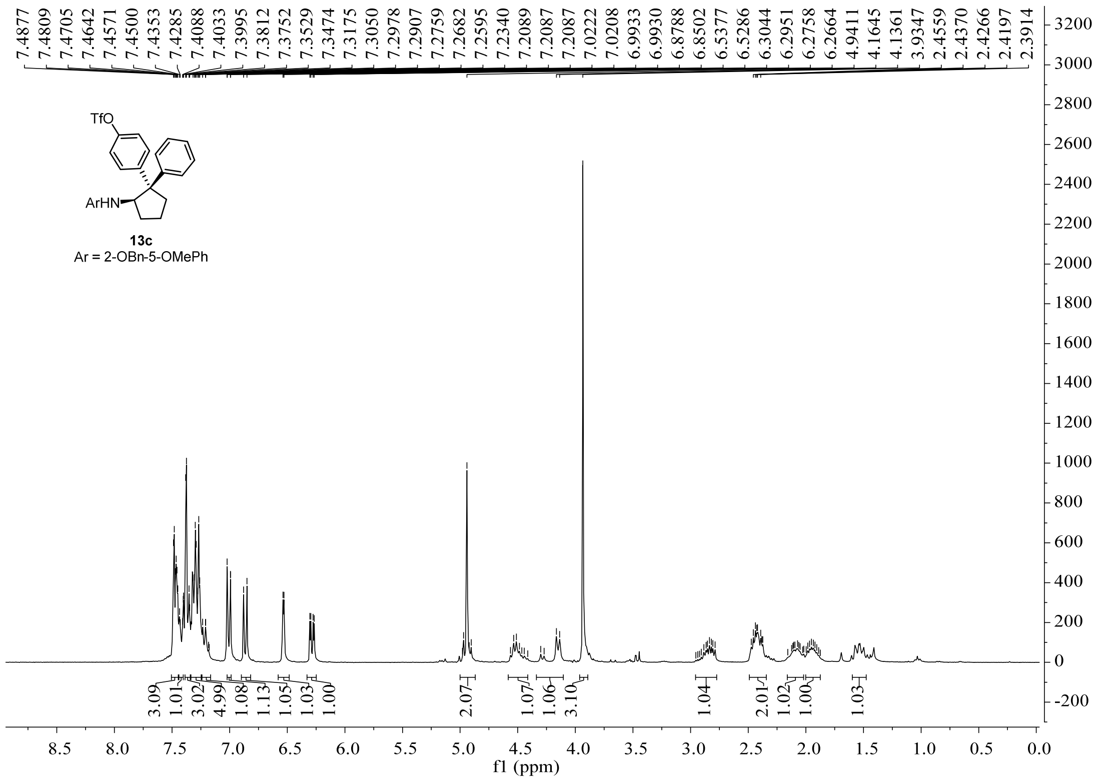
7.4877
7.4809
7.4705
7.4642
7.4571
7.4500
7.4353
7.4285
7.4088
7.4033
7.3995
7.3812
7.3752
7.3529
7.3474
7.3175
7.3050
7.2978
7.2907
7.2759
7.2682
7.2595
7.2340
7.2089
7.2087
7.2087
7.0222
7.0208
6.9933
6.9930
6.8788
6.8502
6.5377
6.5286
6.3044
6.2951
6.2758
6.2664
4.9411
4.1645
4.1361
3.9347
2.4559
2.4370
2.4266
2.4197
2.3914
TfO
ArHN
13c
Ar = 2-OBn-5-OMePh
3.09
1.01
3.02
4.99
1.08
1.13
1.05
1.03
1.00
2.07
1.07
1.06
3.10
1.04
2.01
1.02
1.00
1.03
f1 (ppm)

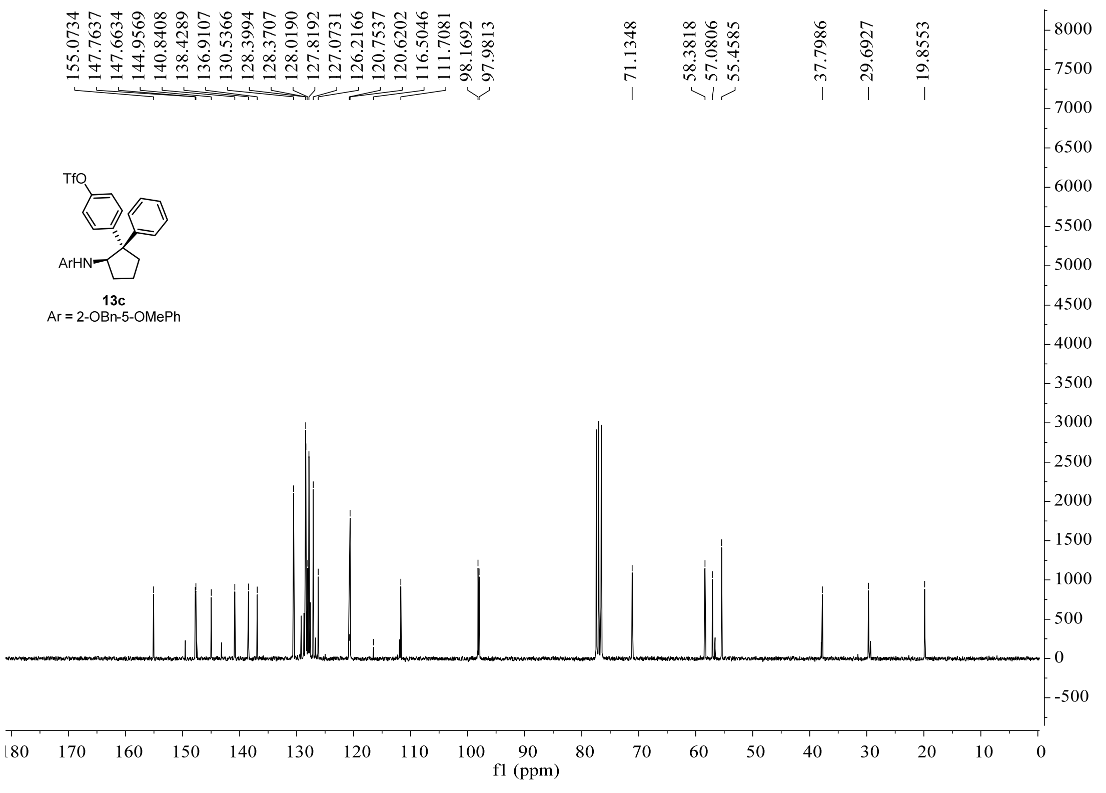
155.0734
147.7637
147.6634
144.9569
140.8408
138.4289
136.9107
130.5366
128.3994
128.3707
128.0190
127.8192
127.0731
126.2166
120.7537
120.6202
116.5046
111.7081
98.1692
97.9813
71.1348
58.3818
57.0806
55.4585
37.7986
29.6927
19.8553
TfO
ArHN
13c
Ar = 2-OBn-5-OMePh
f1 (ppm)

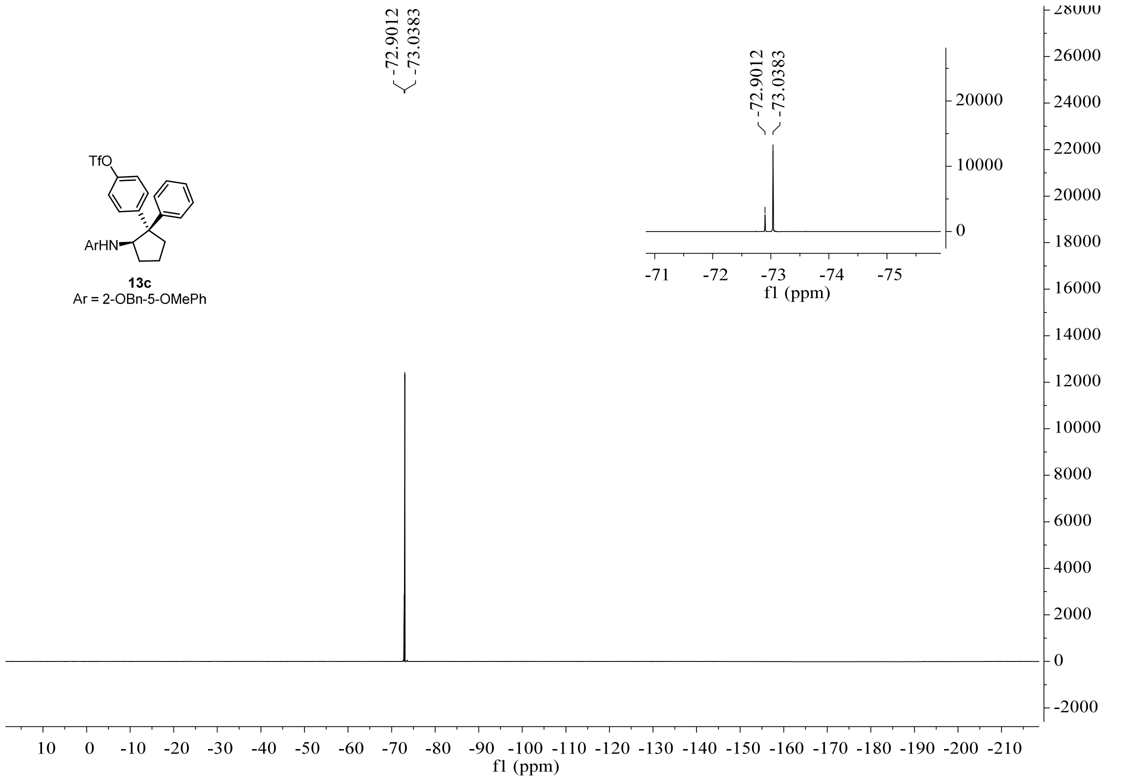
TfO
ArHN
13c
Ar = 2-OBn-5-OMePh
-72.9012
-73.0383
f1 (ppm)

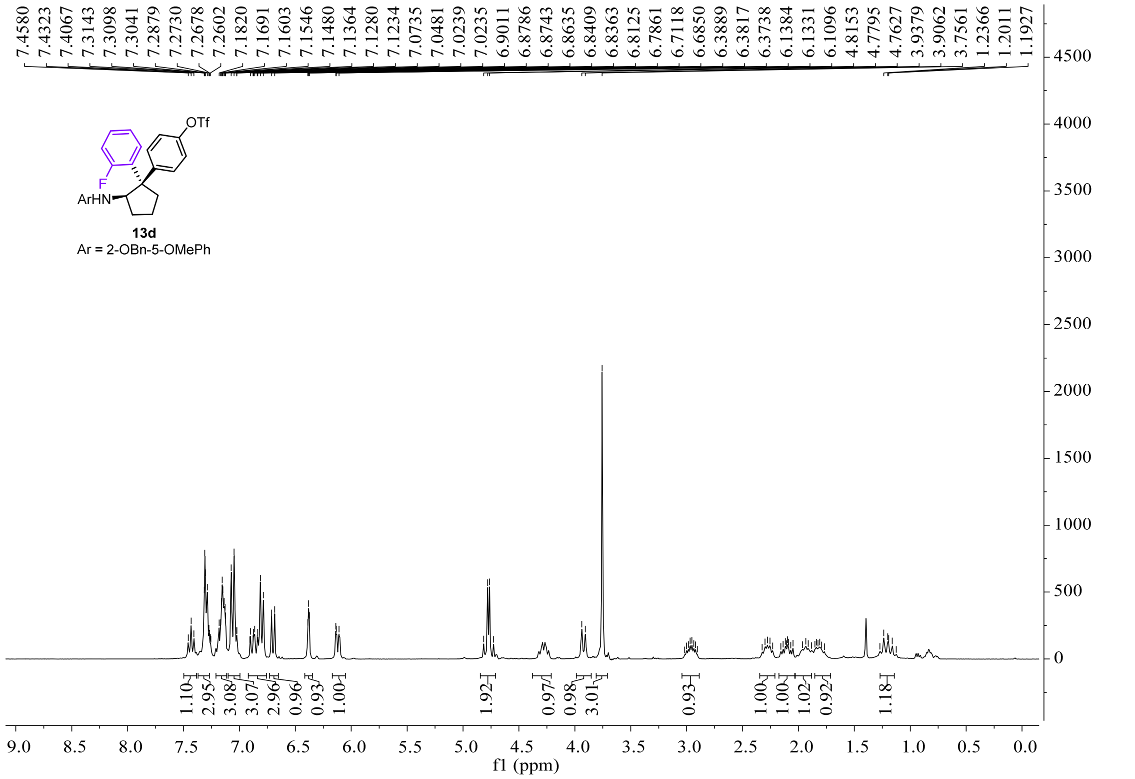
OTf
F
ArHN
13d
Ar = 2-OBn-5-OMePh
f1 (ppm)

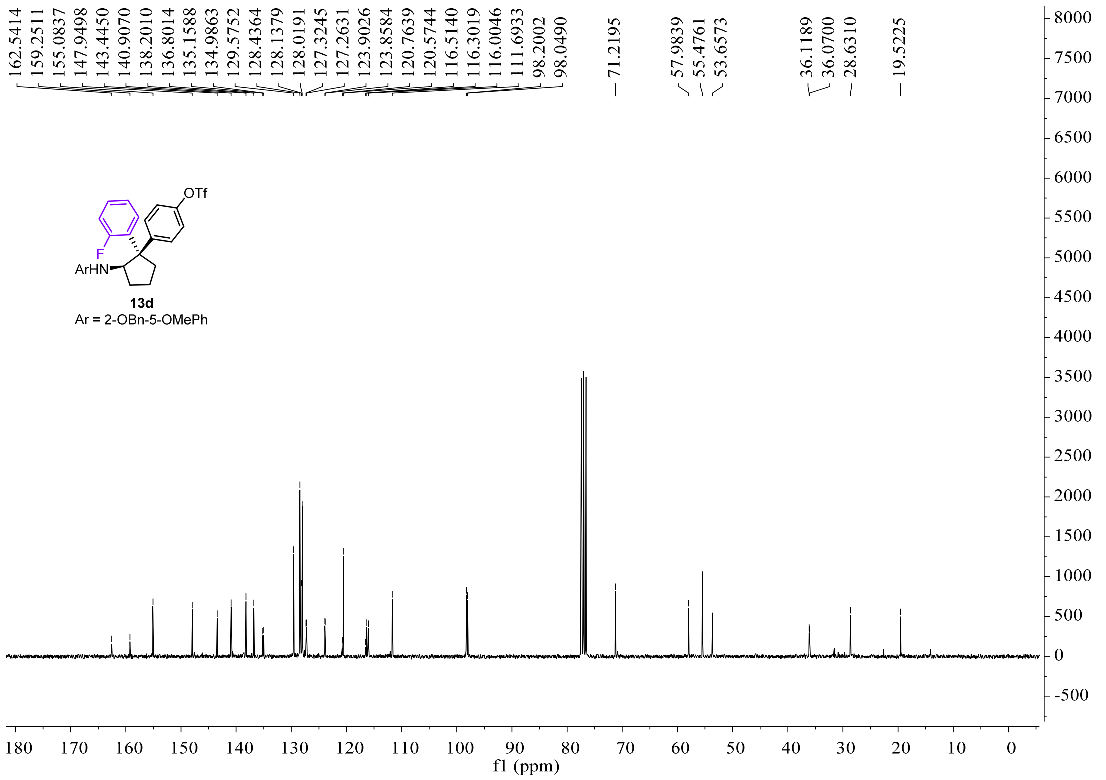
162.5414
159.2511
155.0837
147.9498
143.4450
140.9070
138.2010
136.8014
135.1588
134.9863
129.5752
128.4364
128.1379
128.0191
127.3245
127.2631
123.9026
123.8584
120.7639
120.5744
116.5140
116.3019
116.0046
111.6933
98.2002
98.0490
71.2195
57.9839
55.4761
53.6573
36.1189
36.0700
28.6310
19.5225
OTf
F
ArHN
13d
Ar = 2-OBn-5-OMePh
f1 (ppm)

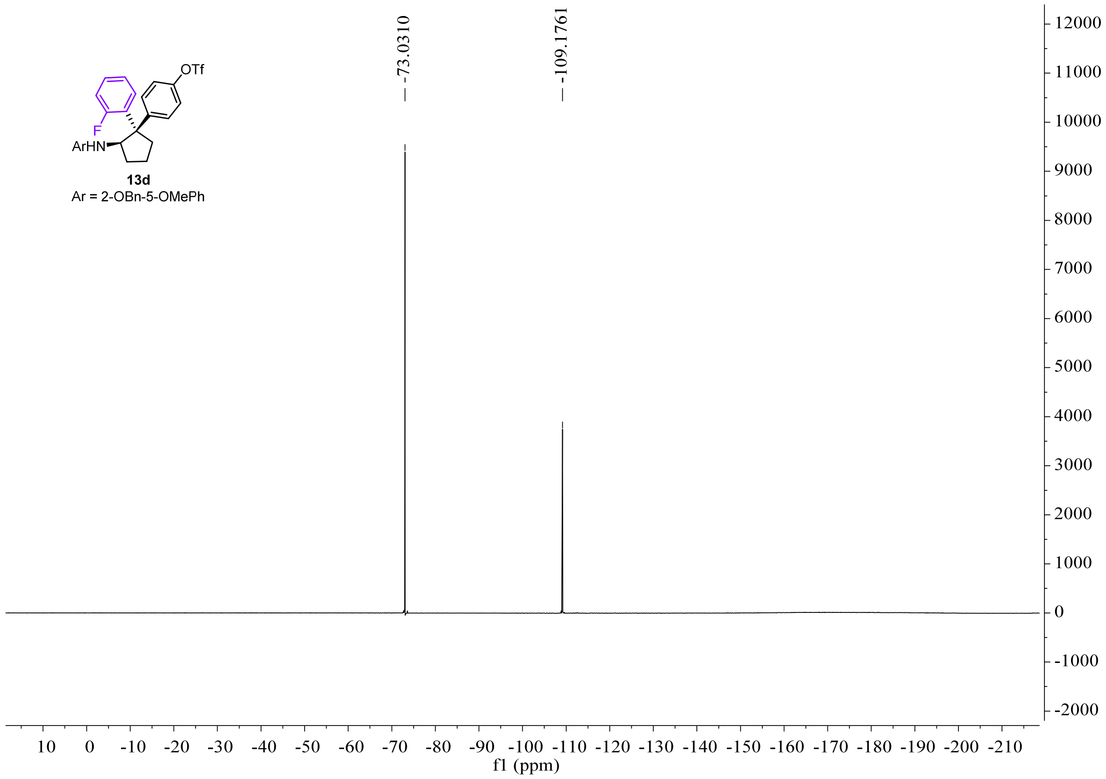
-73.0310
-109.1761
OTf
F
ArHN
13d
Ar = 2-OBn-5-OMePh
f1 (ppm)

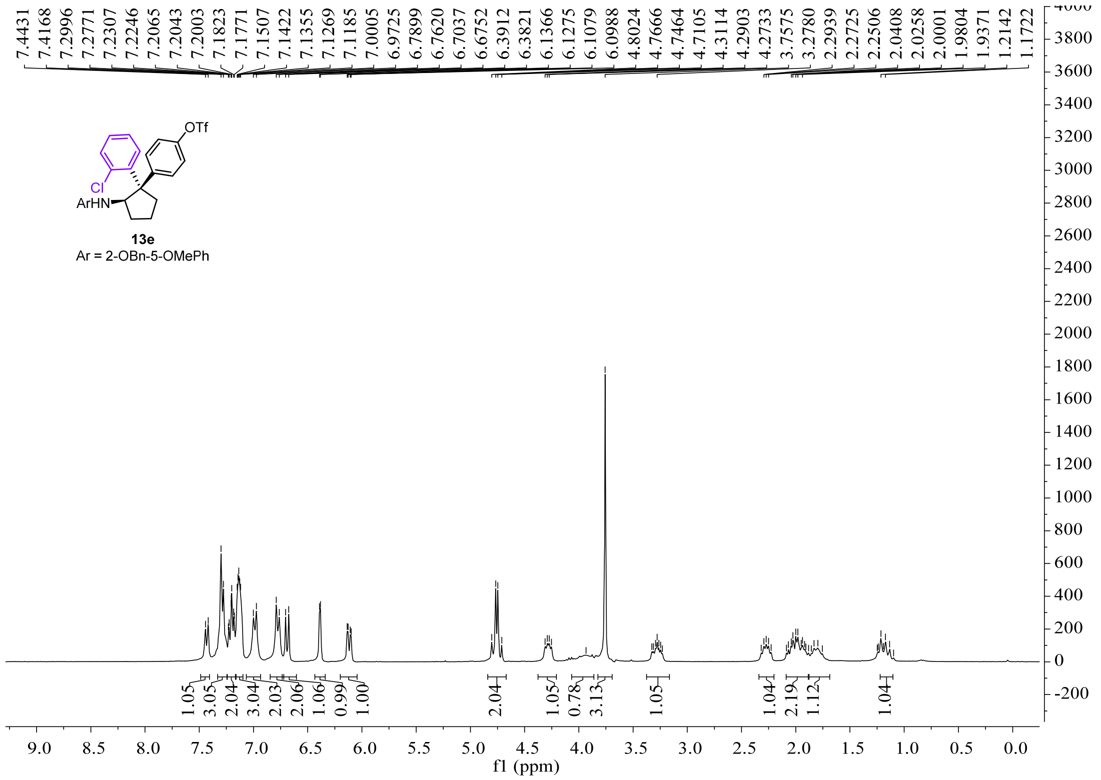
OTf
Cl
ArHN
13e
Ar = 2-OBn-5-OMePh
f1 (ppm)

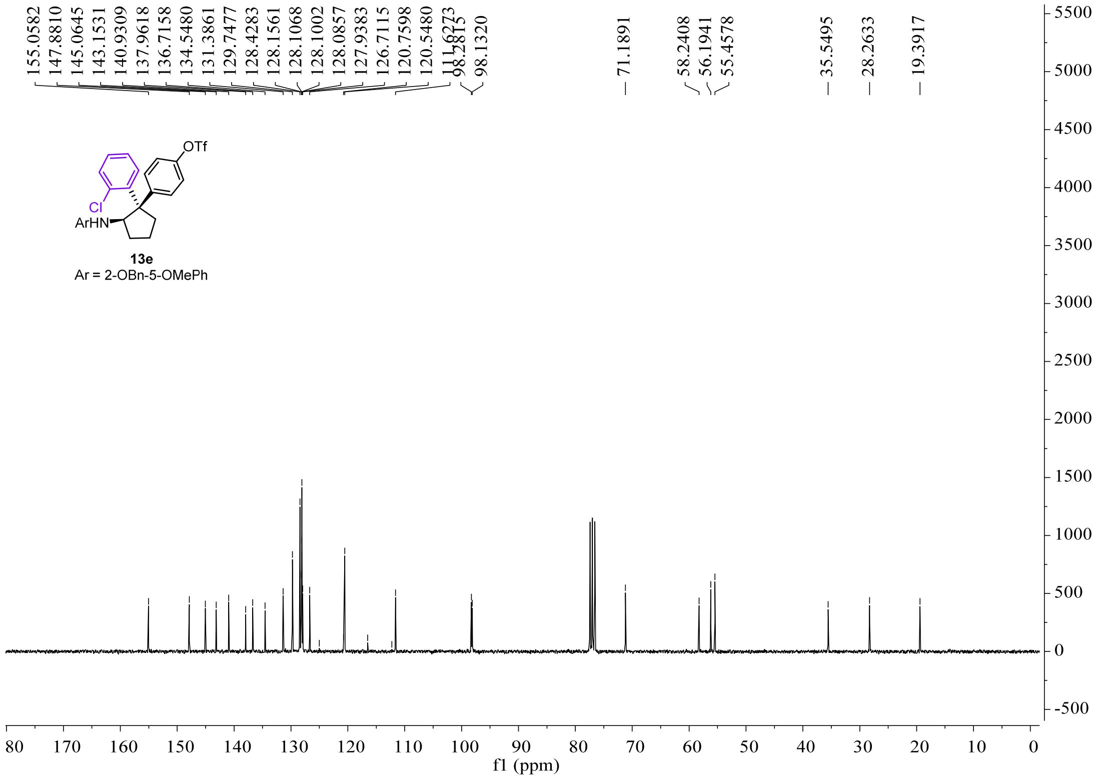
OTf
Cl
ArHN
13e
Ar = 2-OBn-5-OMePh
f1 (ppm)

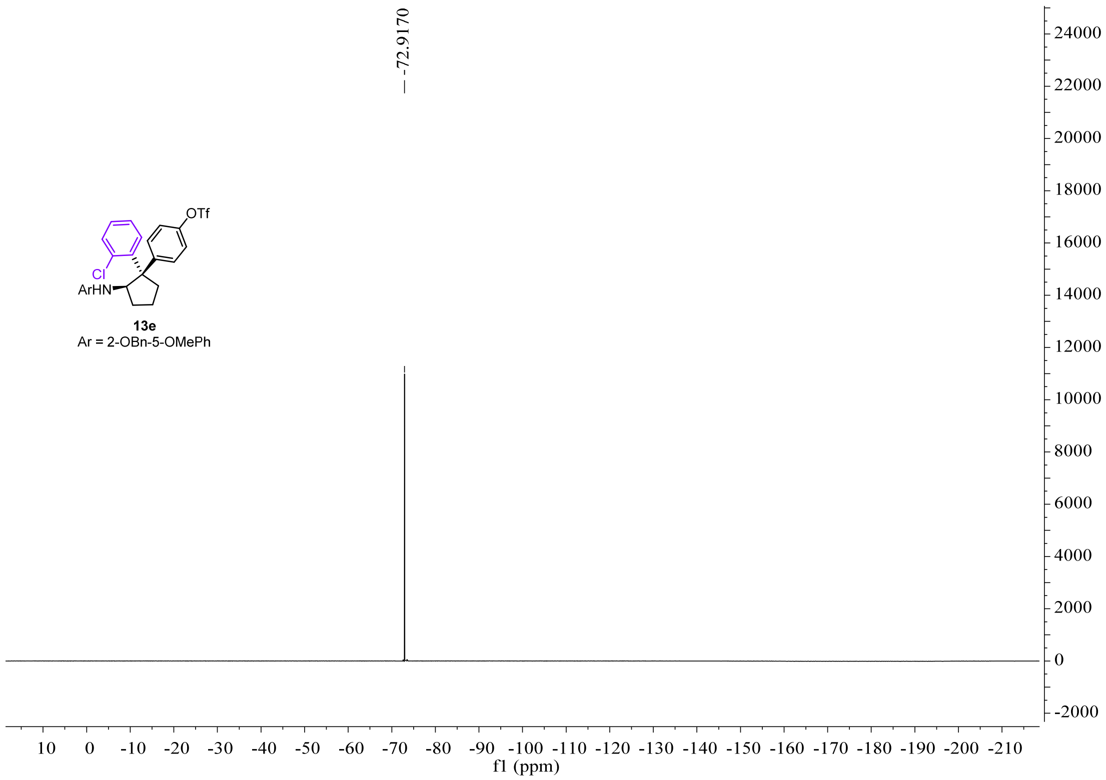

-72.9170
OTf
Cl
ArHN
13e
Ar = 2-OBn-5-OMePh
f1 (ppm)

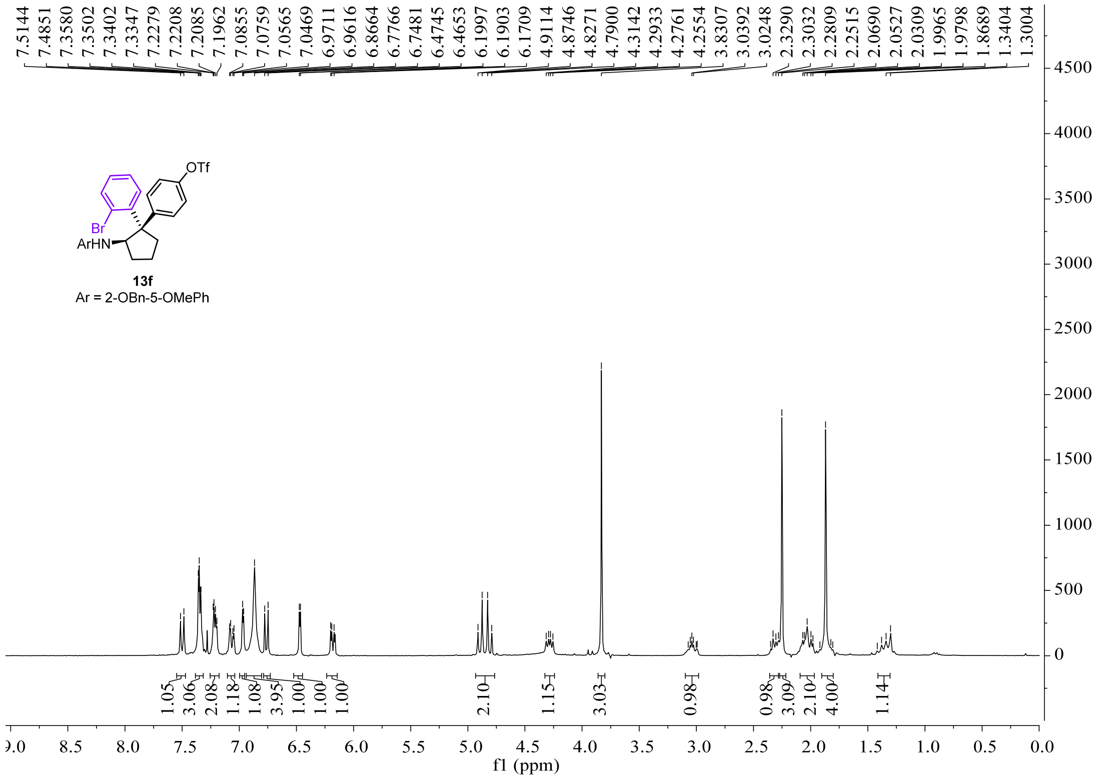

7.5144
7.4851
7.3580
7.3502
7.3402
7.3347
7.2279
7.2208
7.2085
7.1962
7.0855
7.0759
7.0565
7.0469
6.9711
6.9616
6.8664
6.7766
6.7481
6.4745
6.4653
6.1997
6.1903
6.1709
4.9114
4.8746
4.8271
4.7900
4.3142
4.2933
4.2761
4.2554
3.8307
3.0392
3.0248
2.3290
2.3032
2.2809
2.2515
2.0690
2.0527
2.0309
1.9965
1.9798
1.8689
1.3404
1.3004
OTf
Br
ArHN
13f
Ar = 2-OBn-5-OMePh
1.05
3.06
2.08
1.18
1.08
3.95
1.00
1.00
1.00
2.10
1.15
3.03
0.98
0.98
3.09
2.10
4.00
1.14
f1 (ppm)

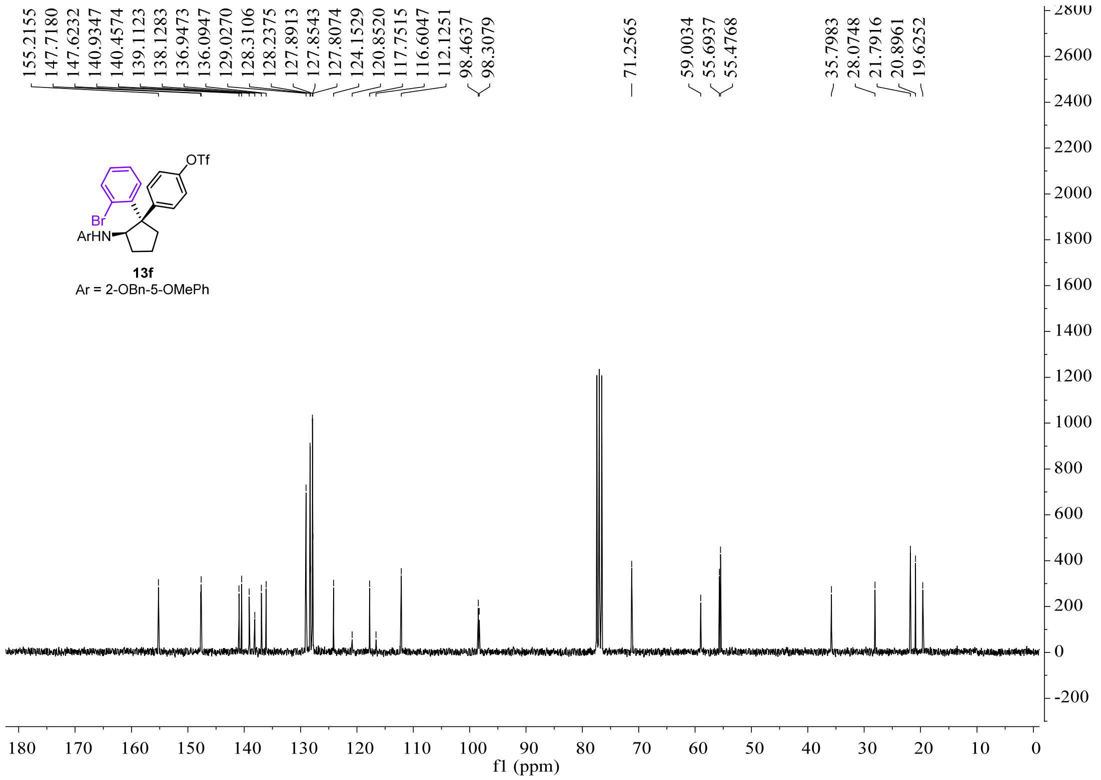

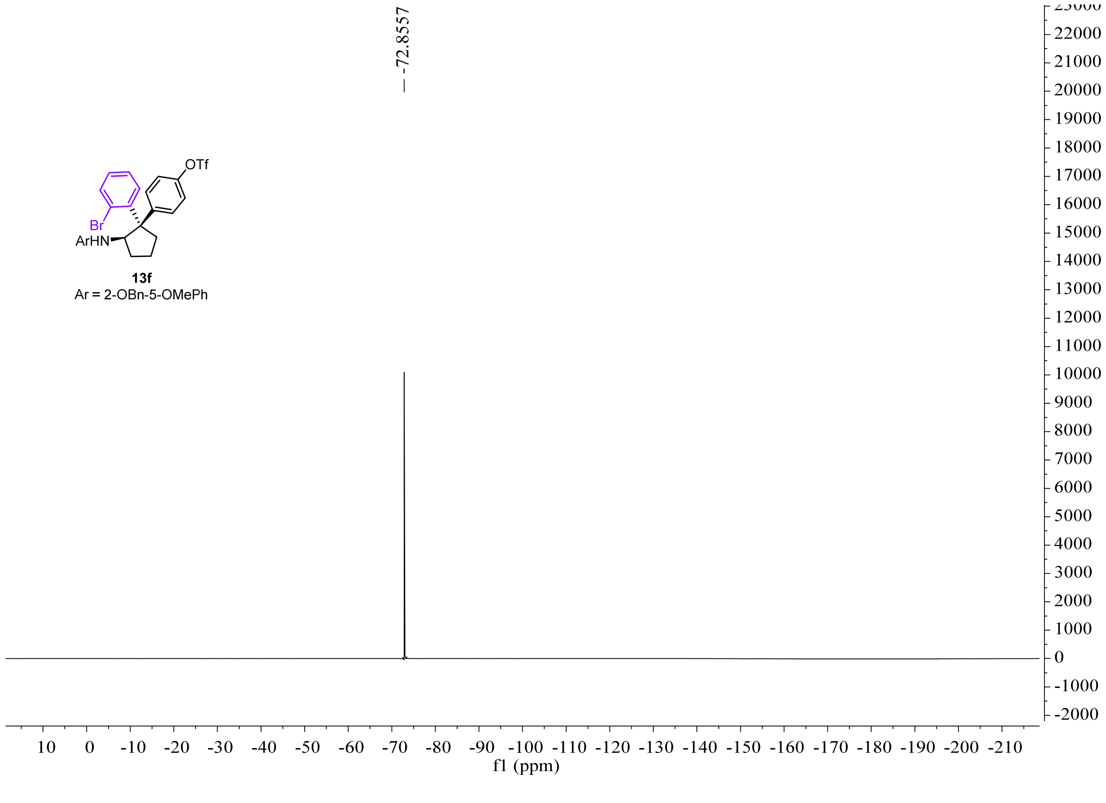

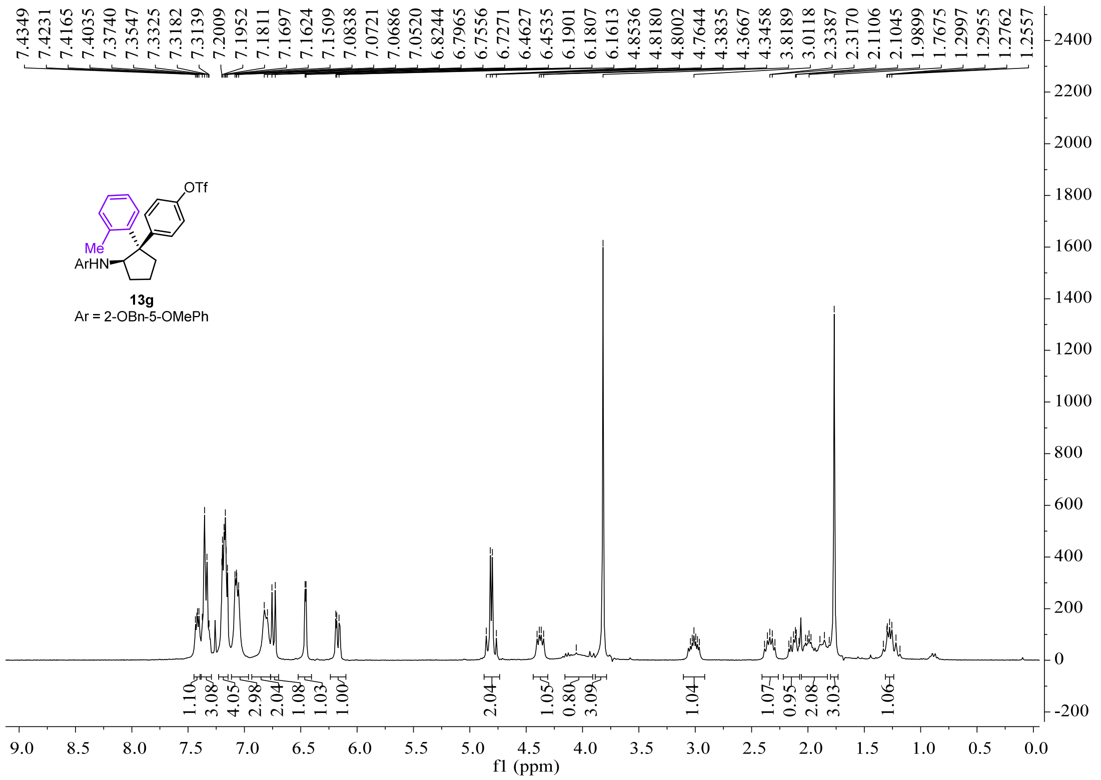
7.4349
7.4231
7.4165
7.4035
7.3740
7.3547
7.3325
7.3182
7.3139
7.2009
7.1952
7.1811
7.1697
7.1624
7.1509
7.0838
7.0721
7.0686
7.0520
6.8244
6.7965
6.7556
6.7271
6.4627
6.4535
6.1901
6.1807
6.1613
4.8536
4.8180
4.8002
4.7644
4.3835
4.3667
4.3458
3.8189
3.0118
2.3387
2.3170
2.1106
2.1045
1.9899
1.7675
1.2997
1.2955
1.2762
1.2557
OTf
Me
ArHN
13g
Ar = 2-OBn-5-OMePh
1.10
3.08
4.05
2.98
2.04
1.08
1.03
1.00
2.04
1.05
0.80
3.09
1.04
1.07
0.95
2.08
3.03
1.06
f1 (ppm)

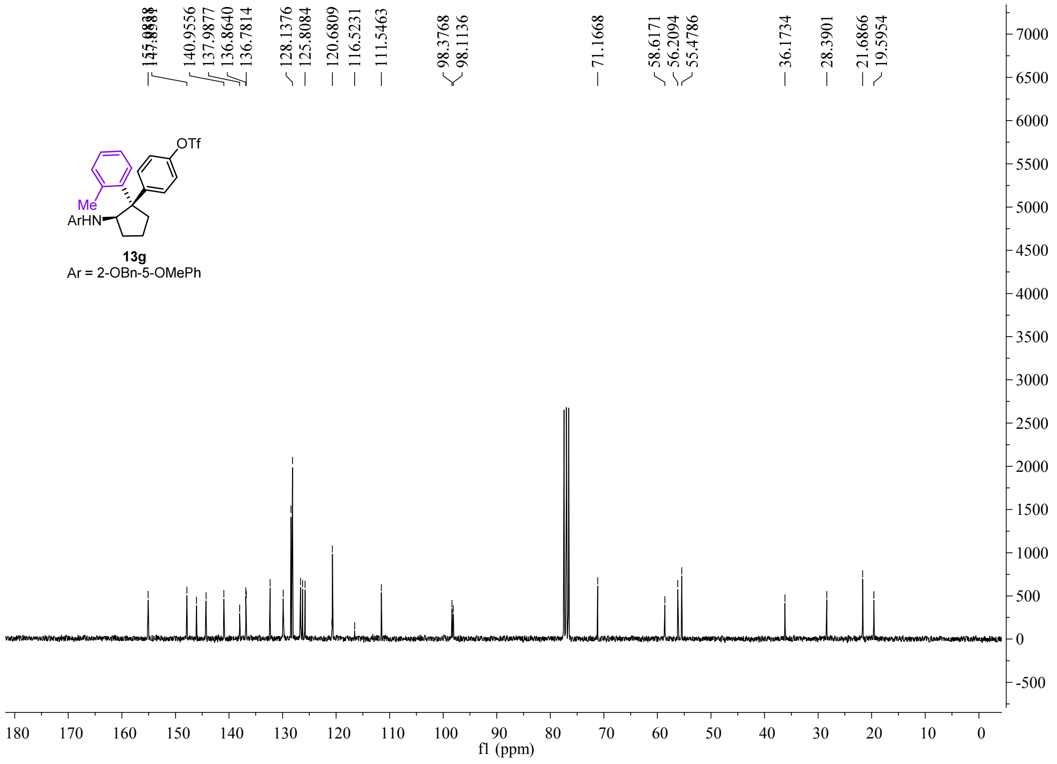
140.9556
137.9877
136.8640
136.7814
128.1376
125.8084
120.6809
116.5231
111.5463
98.3768
98.1136
71.1668
58.6171
56.2094
55.4786
36.1734
28.3901
21.6866
19.5954
OTf
Me
ArHN
13g
Ar = 2-OBn-5-OMePh
f1 (ppm)

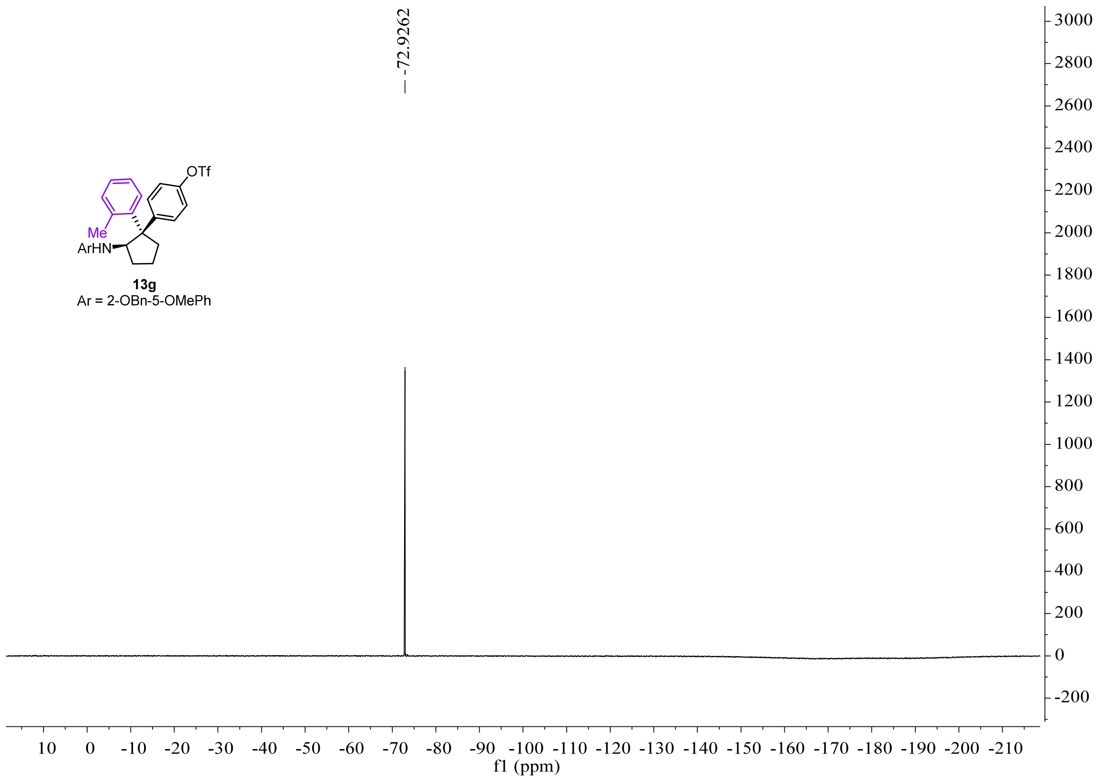
-72.9262
OTf
Me
ArHN
13g
Ar = 2-OBn-5-OMePh
f1 (ppm)

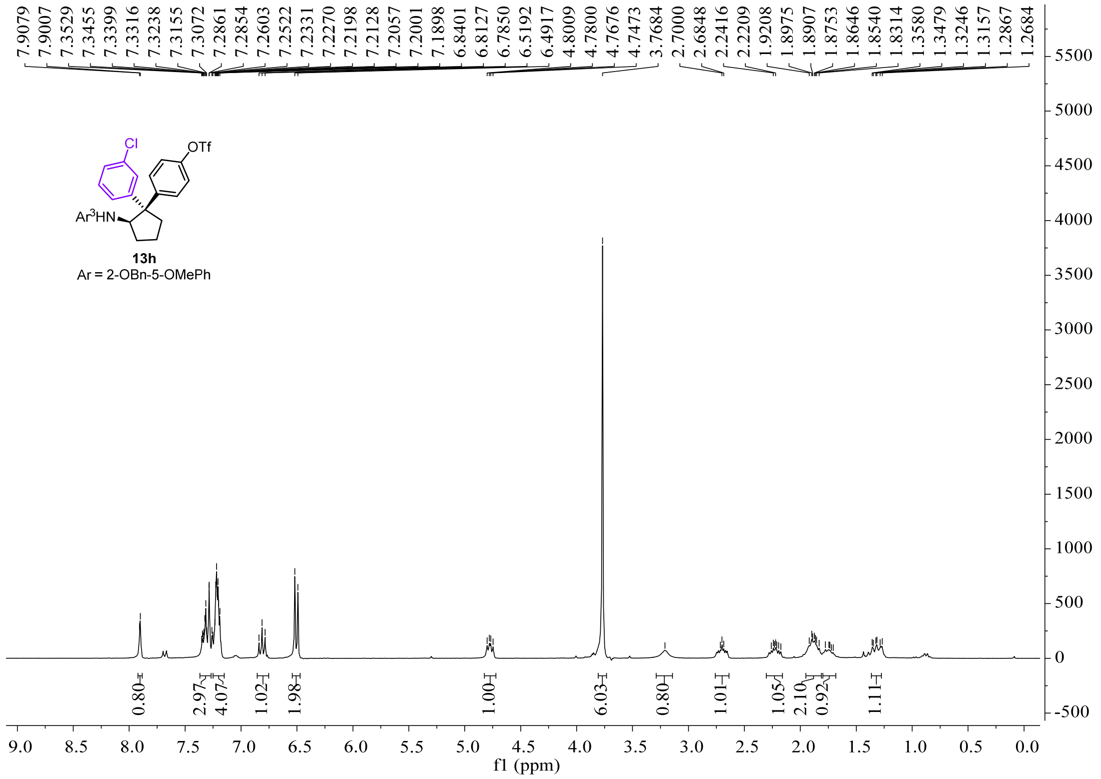
Cl
OTf
Ar3HN
13h
Ar = 2-OBn-5-OMePh
f1 (ppm)

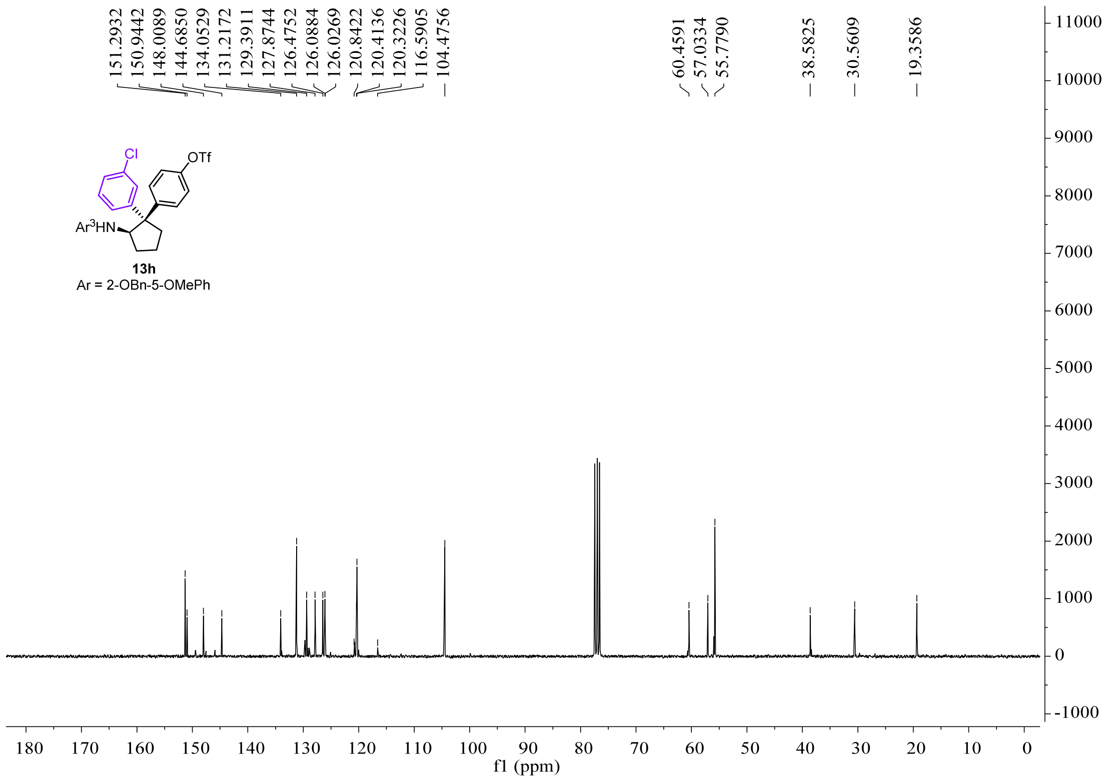
151.2932
150.9442
148.0089
144.6850
134.0529
131.2172
129.3911
127.8744
126.4752
126.0884
126.0269
120.8422
120.4136
120.3226
116.5905
104.4756
60.4591
57.0334
55.7790
38.5825
30.5609
19.3586
Cl
OTf
Ar3HN
13h
Ar = 2-OBn-5-OMePh
f1 (ppm)

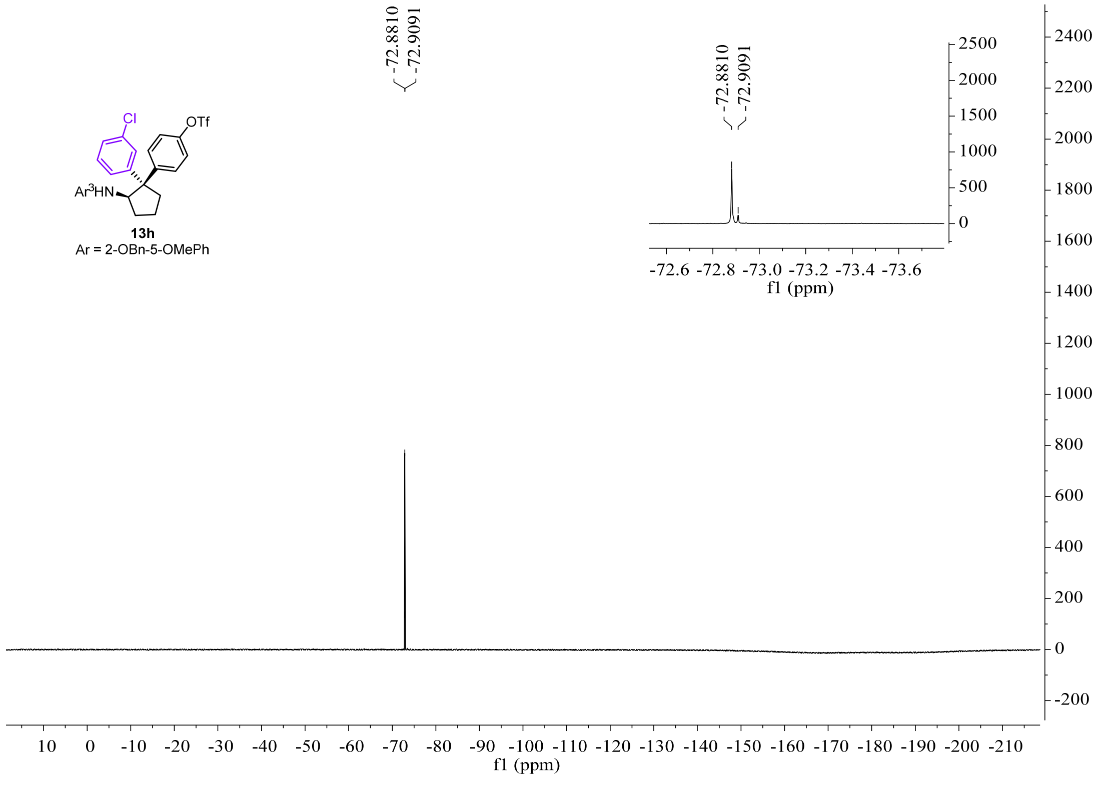
-72.8810
-72.9091
Cl
OTf
Ar3HN
13h
Ar = 2-OBn-5-OMePh
f1 (ppm)

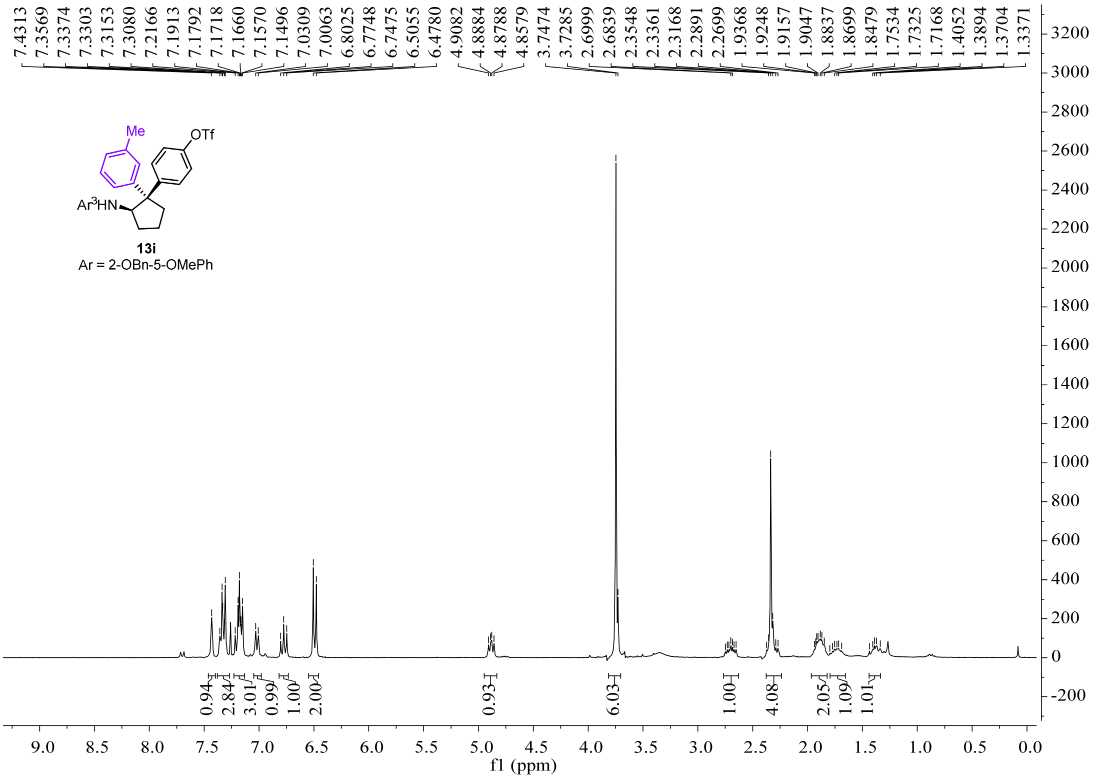

7.4313
7.3569
7.3374
7.3303
7.3153
7.3080
7.2166
7.1913
7.1792
7.1718
7.1660
7.1570
7.1496
7.0309
7.0063
6.8025
6.7748
6.7475
6.5055
6.4780
4.9082
4.8884
4.8788
4.8579
3.7474
3.7285
2.6999
2.6839
2.3548
2.3361
2.3168
2.2891
2.2699
1.9368
1.9248
1.9157
1.9047
1.8837
1.8699
1.8479
1.7534
1.7325
1.7168
1.4052
1.3894
1.3704
1.3371
Me
OTf
Ar3HN
13i
Ar = 2-OBn-5-OMePh
0.94
2.84
3.01
0.99
1.00
2.00
0.93
6.03
1.00
4.08
2.05
1.09
1.01
f1 (ppm)

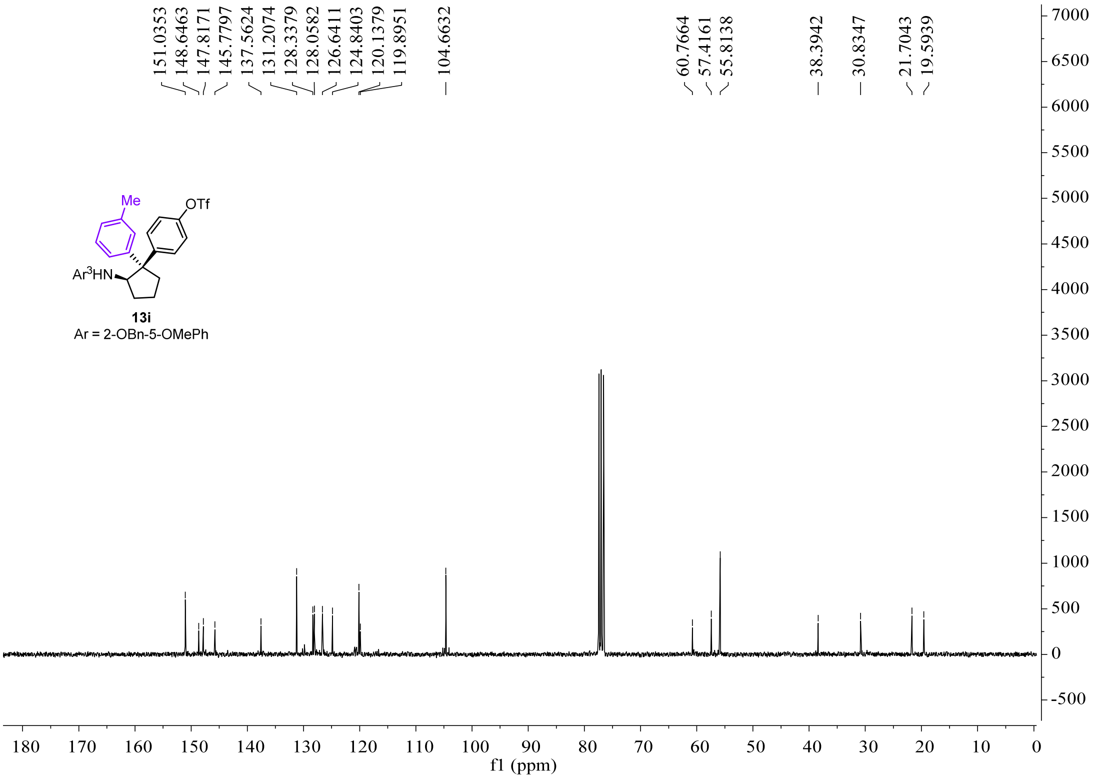

151.0353
148.6463
147.8171
145.7797
137.5624
131.2074
128.3379
128.0582
126.6411
124.8403
120.1379
119.8951
104.6632
60.7664
57.4161
55.8138
38.3942
30.8347
21.7043
19.5939
Me
OTf
Ar3HN
13i
Ar = 2-OBn-5-OMePh
f1 (ppm)

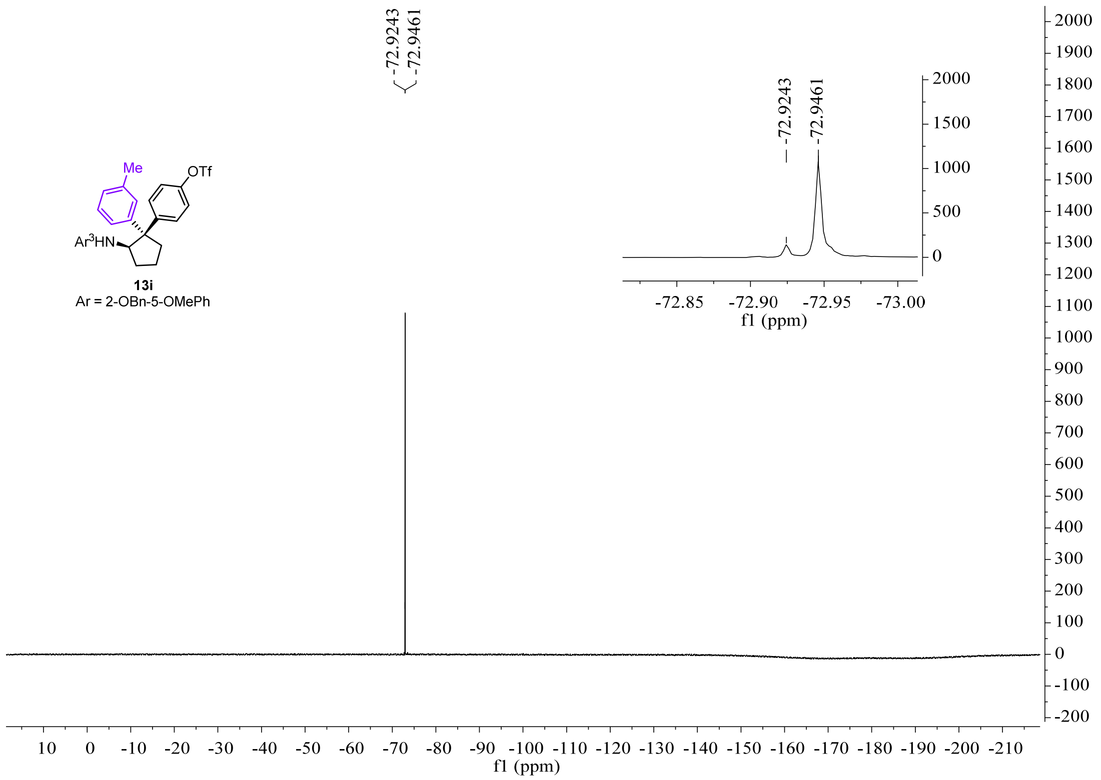

-72.9243
-72.9461
Me
OTf
Ar3HN
13i
Ar = 2-OBn-5-OMePh
-72.85 -72.90 -72.95 -73.00
f1 (ppm)
f1 (ppm)

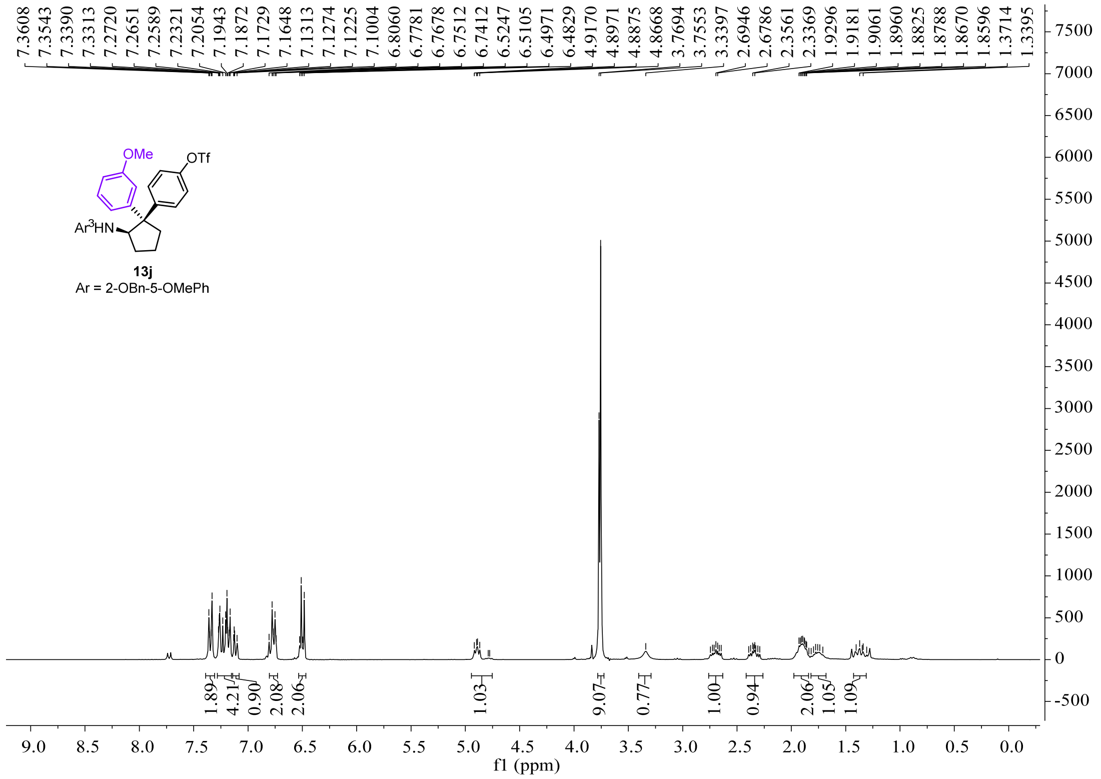

OMe
OTf
Ar3HN
13j
Ar = 2-OBn-5-OMePh
1.89
4.21
0.90
2.08
2.06
1.03
9.07
0.77
1.00
0.94
2.06
1.05
1.09
f1 (ppm)

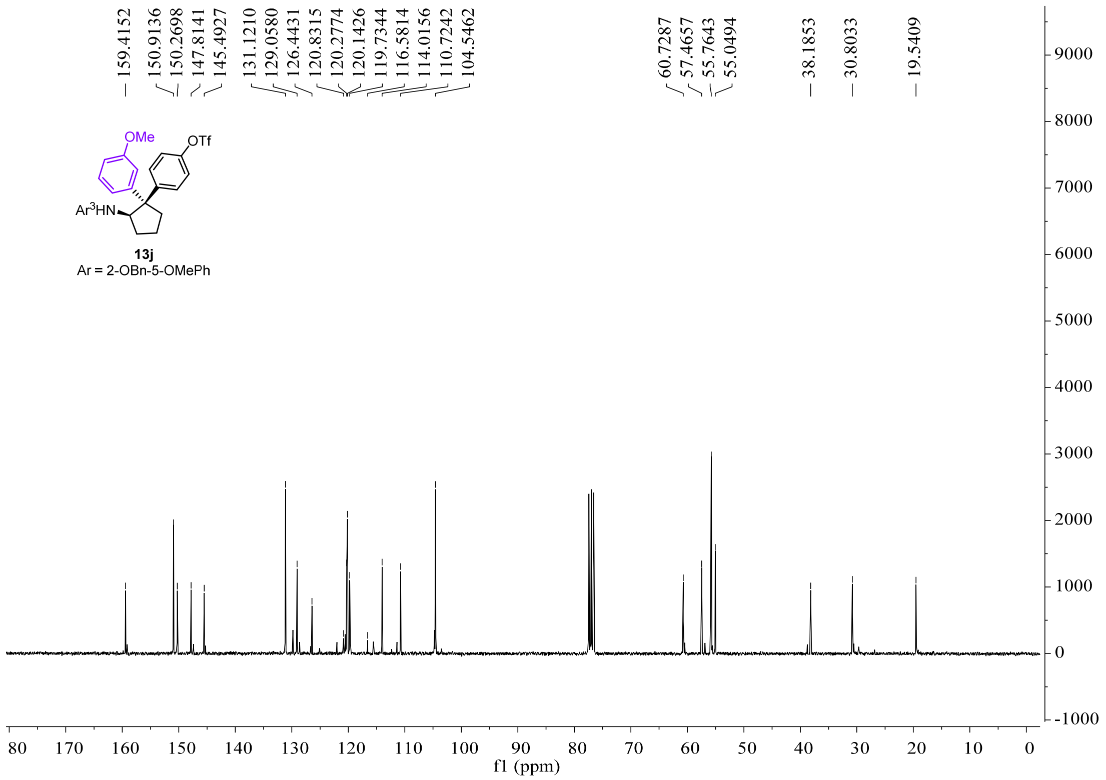
159.4152
150.9136
150.2698
147.8141
145.4927
131.1210
129.0580
126.4431
120.8315
120.2774
120.1426
119.7344
116.5814
114.0156
110.7242
104.5462
60.7287
57.4657
55.7643
55.0494
38.1853
30.8033
19.5409
OMe
OTf
Ar^{3}HN
13j
Ar = 2-OBn-5-OMePh
f1 (ppm)

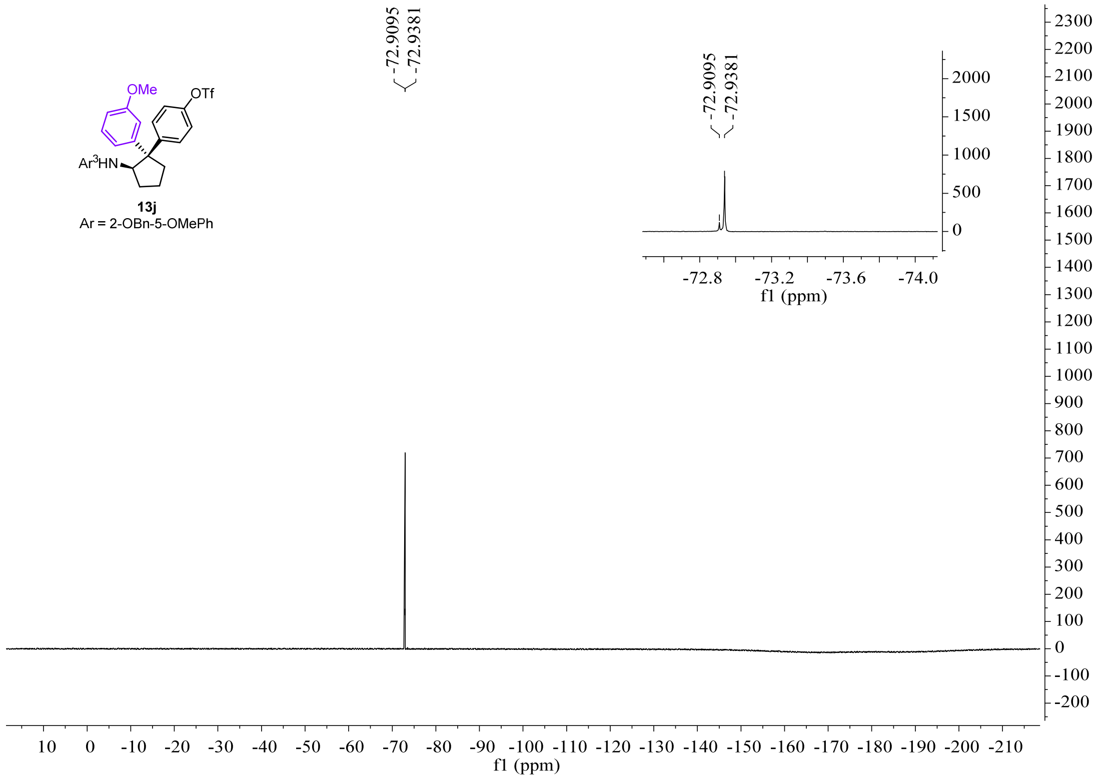
-72.9095
-72.9381
OMe
OTf
Ar^{3}HN
13j
Ar = 2-OBn-5-OMePh
f1 (ppm)

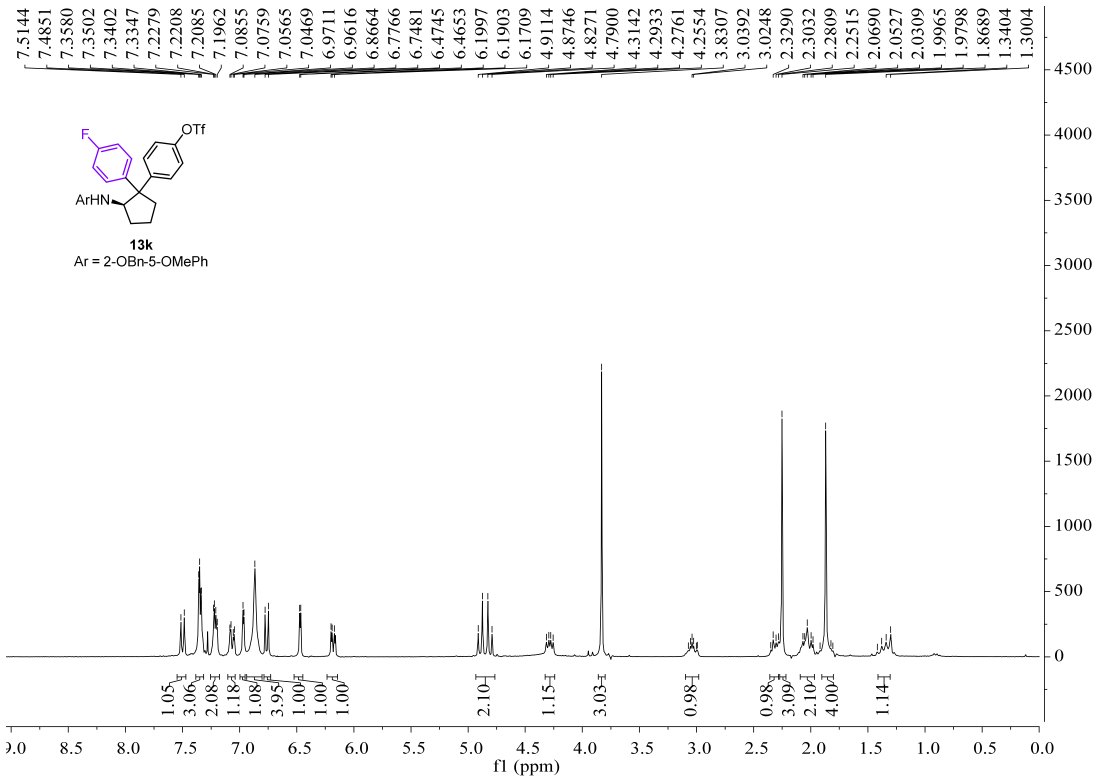
13k
Ar = 2-OBn-5-OMePh
f1 (ppm)

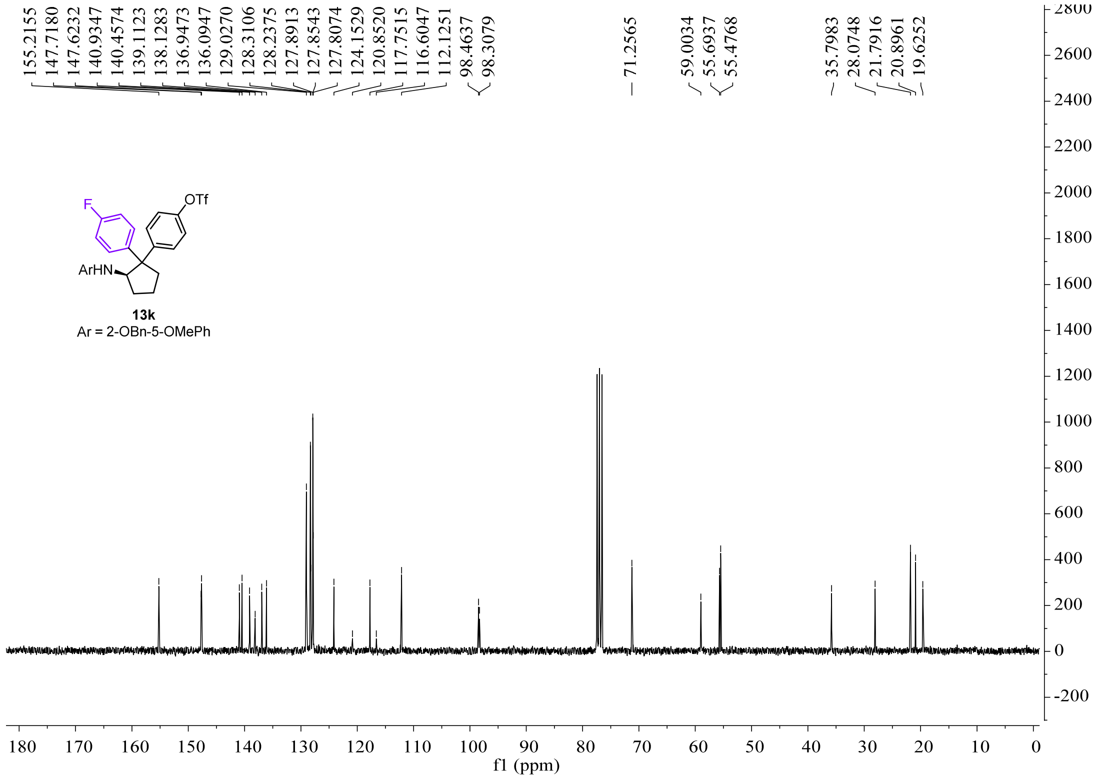
13k
Ar = 2-OBn-5-OMePh
f1 (ppm)

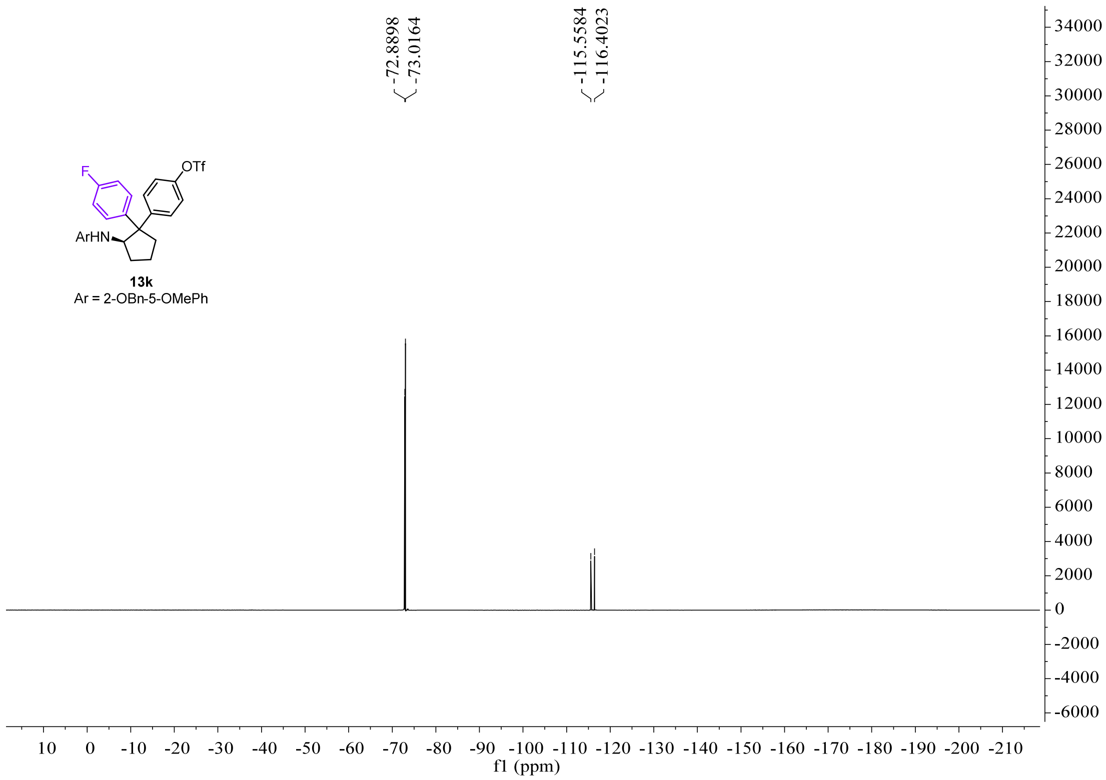
-72.8898
-73.0164
-115.5584
-116.4023
F
OTf
ArHN
13k
Ar = 2-OBn-5-OMePh
f1 (ppm)

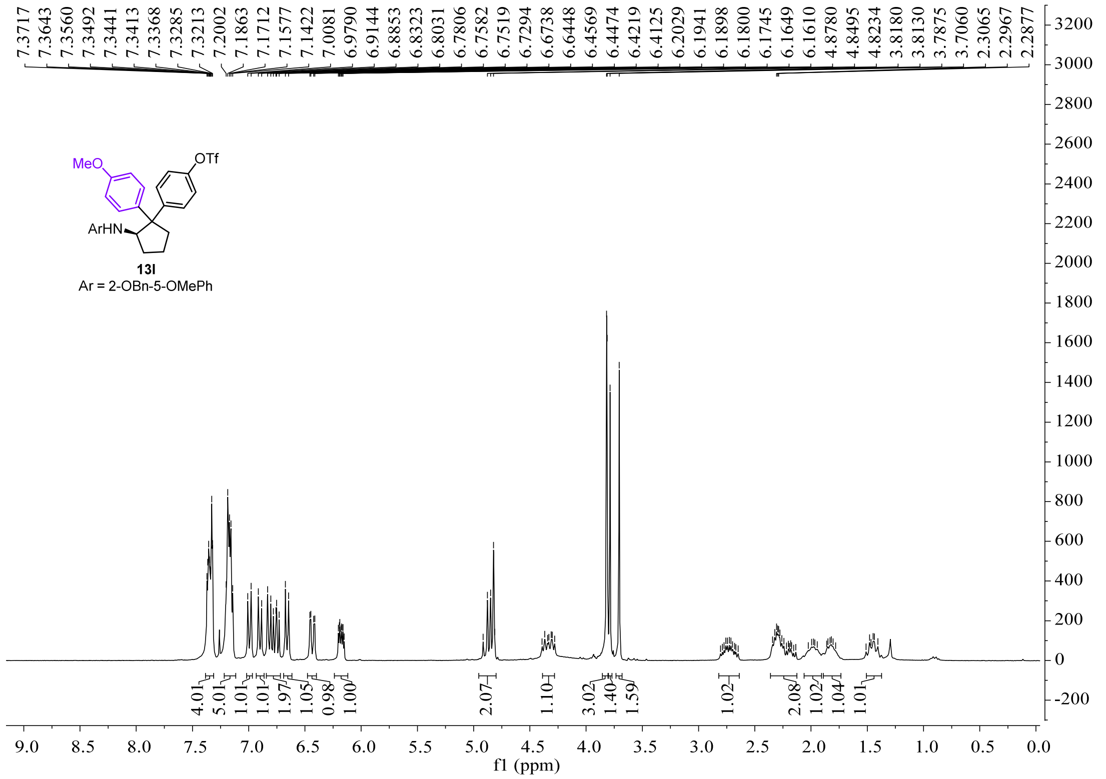
7.3717
7.3643
7.3560
7.3492
7.3441
7.3413
7.3368
7.3285
7.3213
7.2002
7.1863
7.1712
7.1577
7.1422
7.0081
6.9790
6.9144
6.8853
6.8323
6.8031
6.7806
6.7582
6.7519
6.7294
6.6738
6.6448
6.4569
6.4474
6.4219
6.4125
6.2029
6.1941
6.1898
6.1800
6.1745
6.1649
6.1610
4.8780
4.8495
4.8234
3.8180
3.8130
3.7875
3.7060
2.3065
2.2967
2.2877
MeO
OTf
ArHN
13l
Ar = 2-OBn-5-OMePh
4.01
5.01
1.01
1.01
1.97
1.05
0.98
1.00
2.07
1.10
3.02
1.40
1.59
1.02
2.08
1.02
1.04
1.01
f1 (ppm)

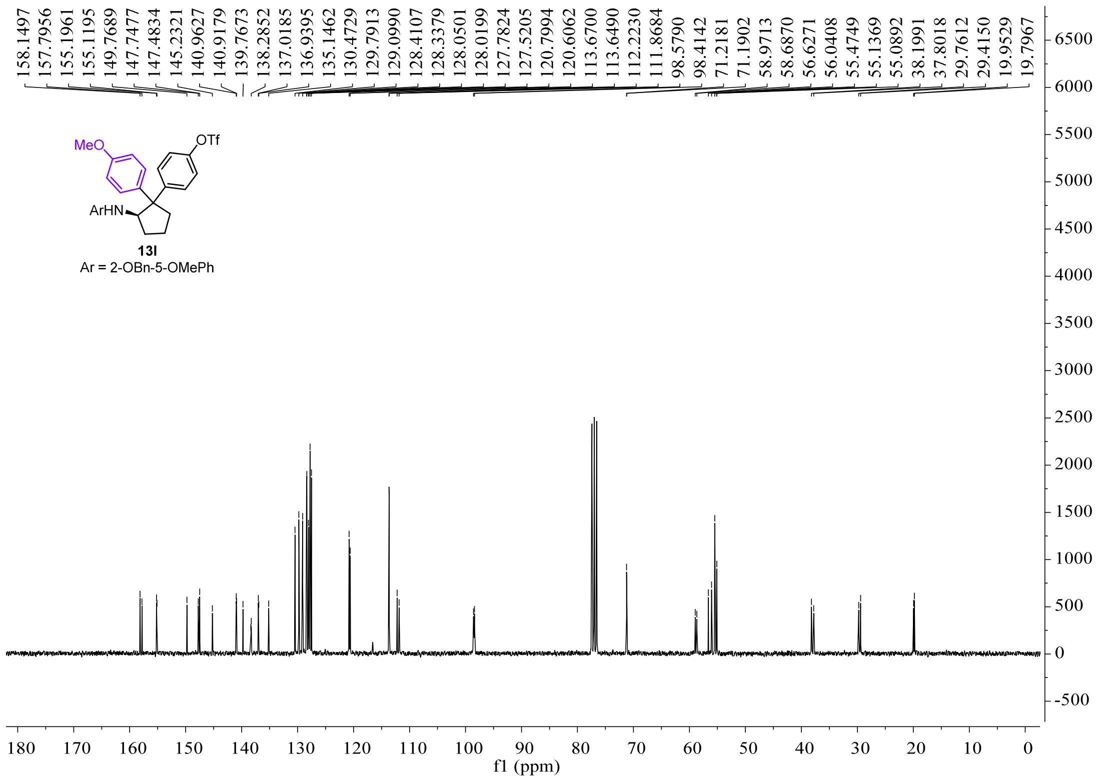
158.1497
157.7956
155.1961
155.1195
149.7689
147.7477
147.4834
145.2321
140.9627
140.9179
139.7673
138.2852
137.0185
136.9395
135.1462
130.4729
129.7913
129.0990
128.4107
128.3379
128.0501
128.0199
127.7824
127.5205
120.7994
120.6062
113.6700
113.6490
112.2230
111.8684
98.5790
98.4142
71.2181
71.1902
58.9713
58.6870
56.6271
56.0408
55.4749
55.1369
55.0892
38.1991
37.8018
29.7612
29.4150
19.9529
19.7967
MeO
OTf
ArHN
13l
Ar = 2-OBn-5-OMePh
f1 (ppm)

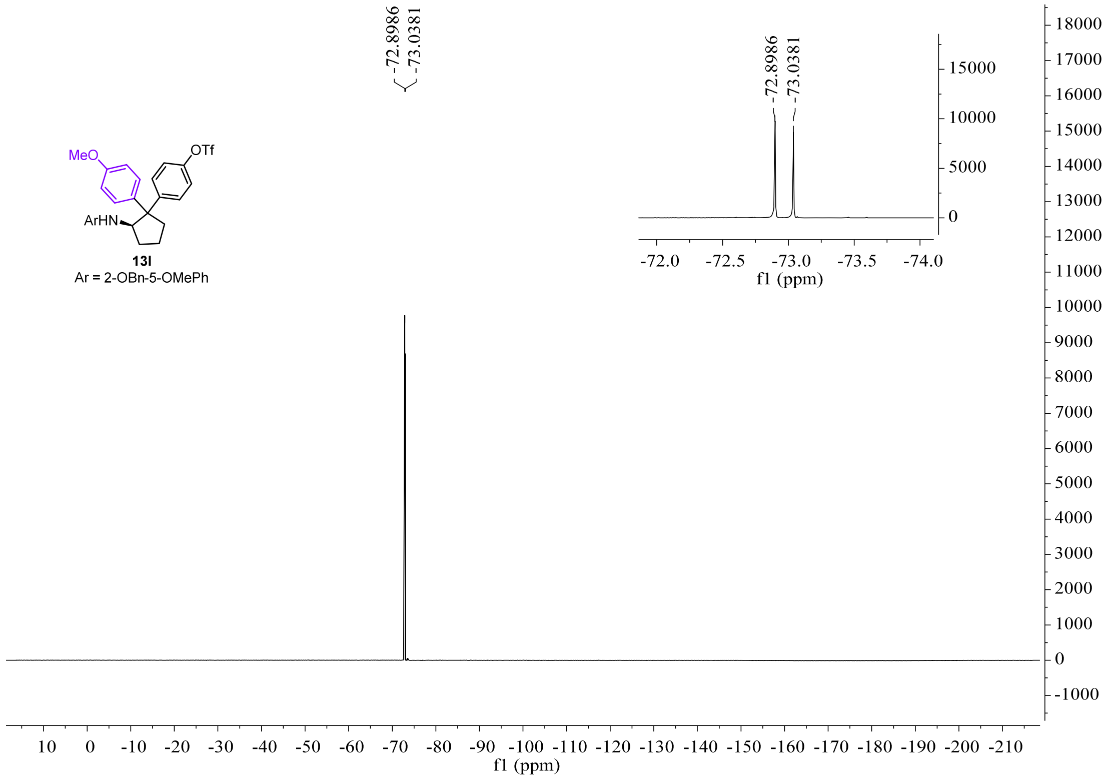
-72.8986
-73.0381
MeO
OTf
ArHN
13l
Ar = 2-OBn-5-OMePh
f1 (ppm)

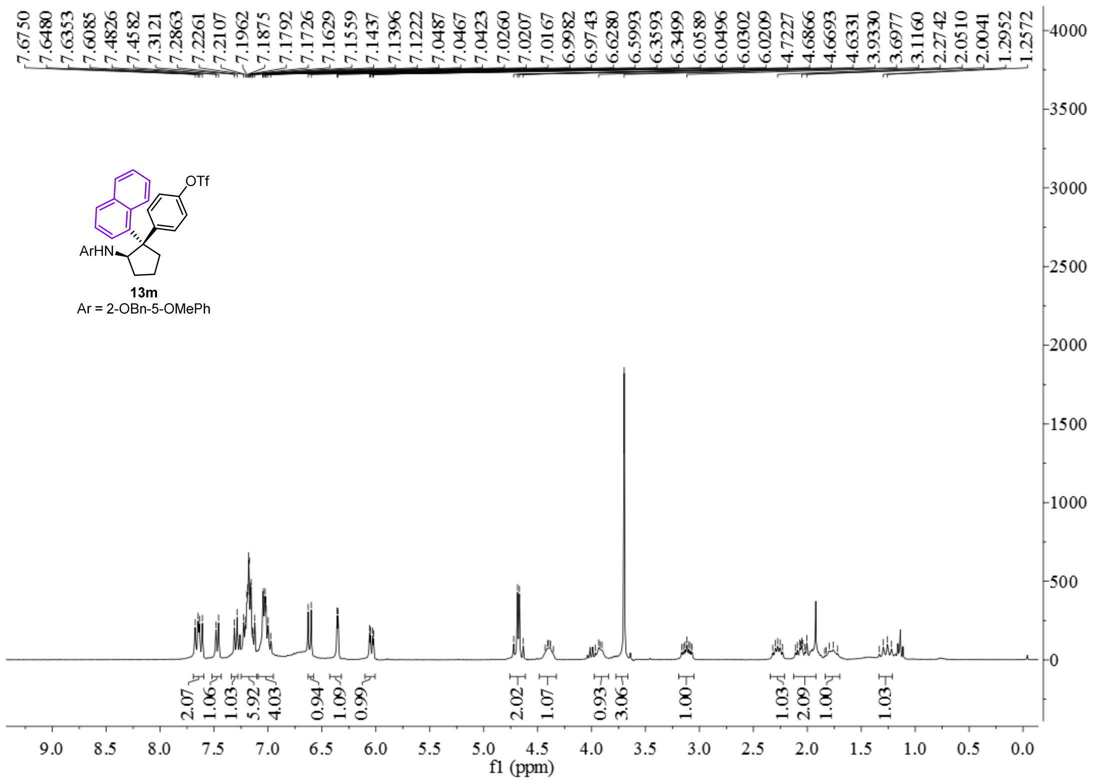

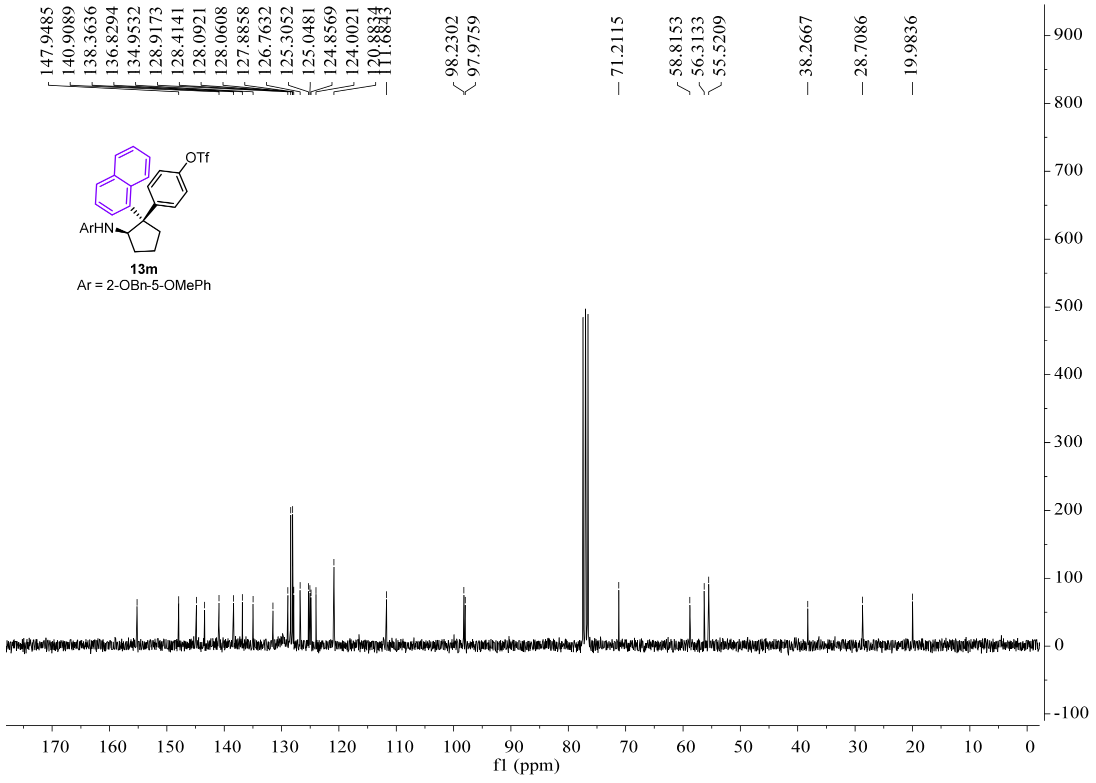

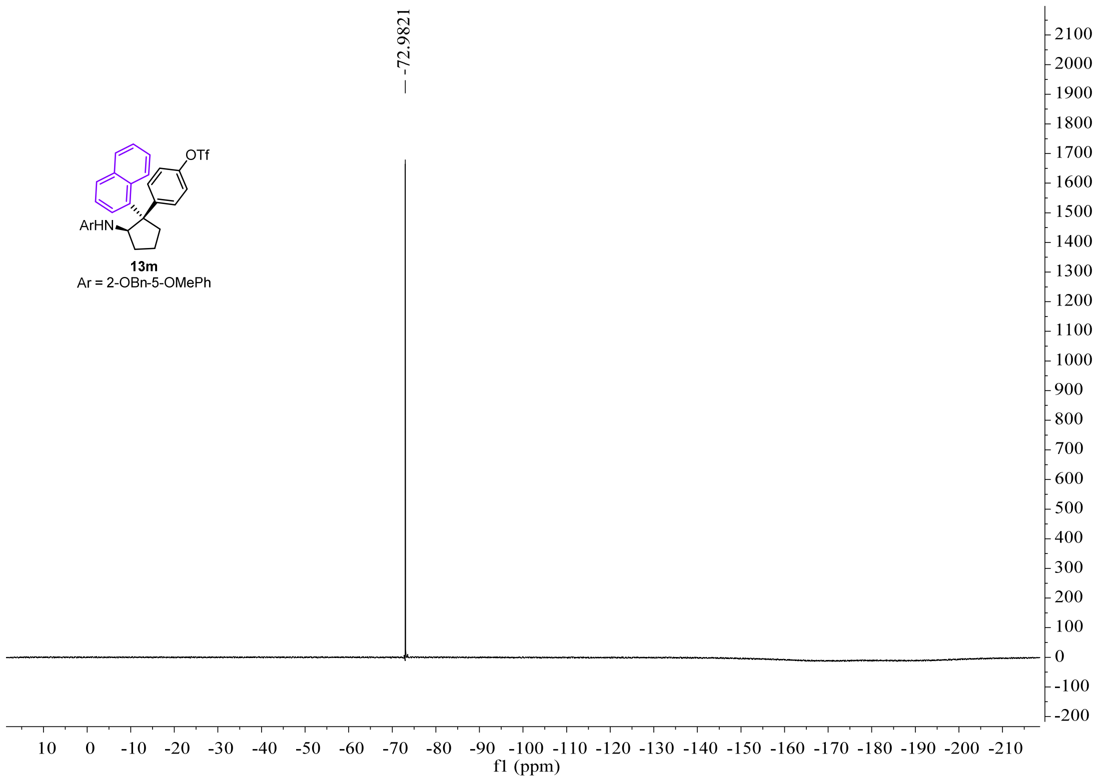
-72.9821
OTf
ArHN
13m
Ar = 2-OBn-5-OMePh
f1 (ppm)

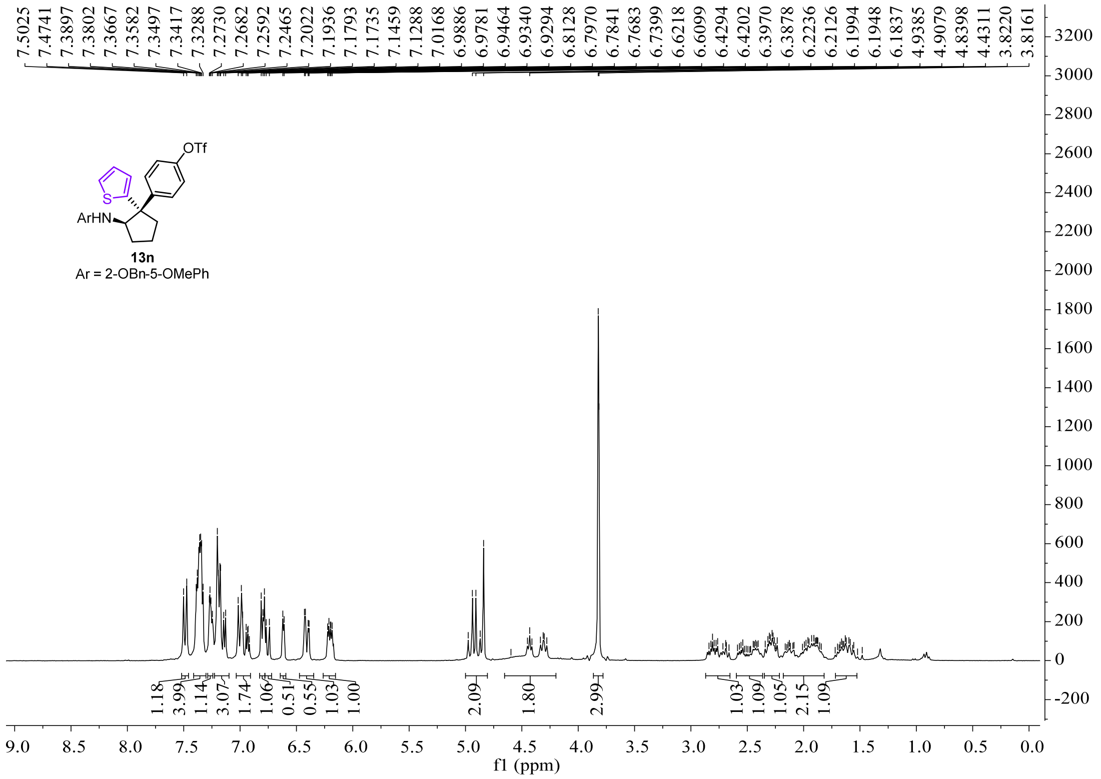
OTf
S
ArHN
13n
Ar = 2-OBn-5-OMePh
f1 (ppm)

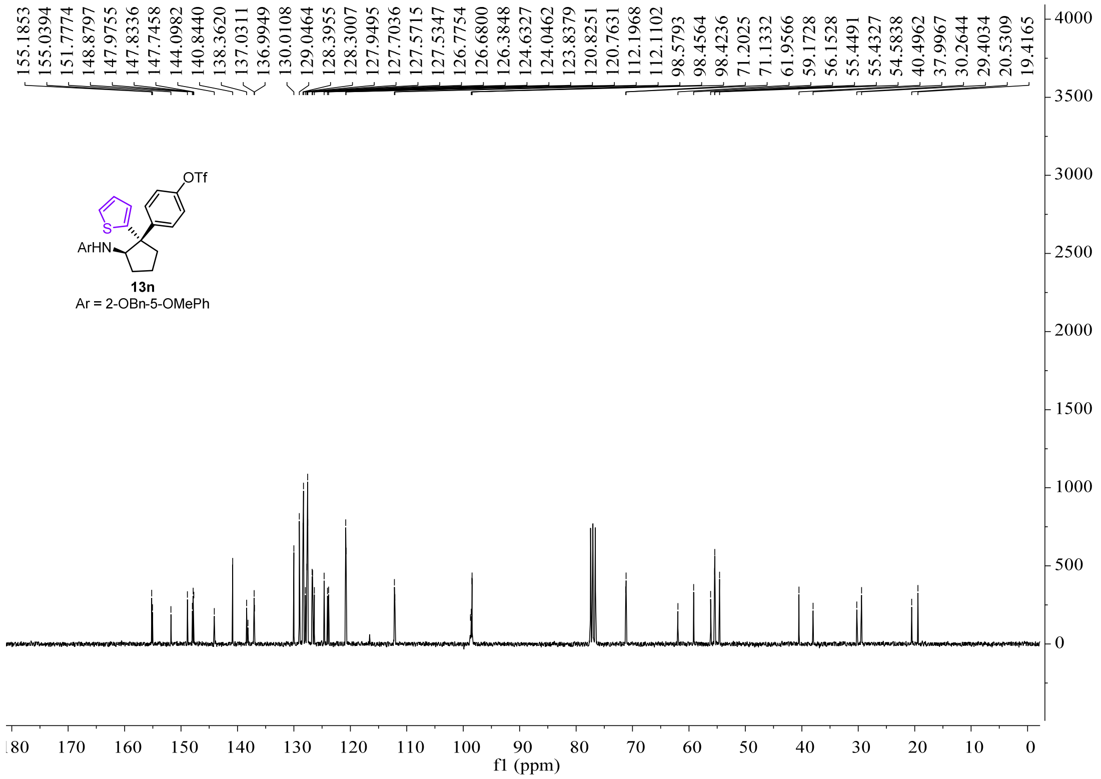
155.1853
155.0394
151.7774
148.8797
147.9755
147.8336
147.7458
144.0982
140.8440
138.3620
137.0311
136.9949
130.0108
129.0464
128.3955
128.3007
127.9495
127.7036
127.5715
127.5347
126.7754
126.6800
126.3848
124.6327
124.0462
123.8379
120.8251
120.7631
112.1968
112.1102
98.5793
98.4564
98.4236
71.2025
71.1332
61.9566
59.1728
56.1528
55.4491
55.4327
54.5838
40.4962
37.9967
30.2644
29.4034
20.5309
19.4165
OTf
S
ArHN
13n
Ar = 2-OBn-5-OMePh
f1 (ppm)

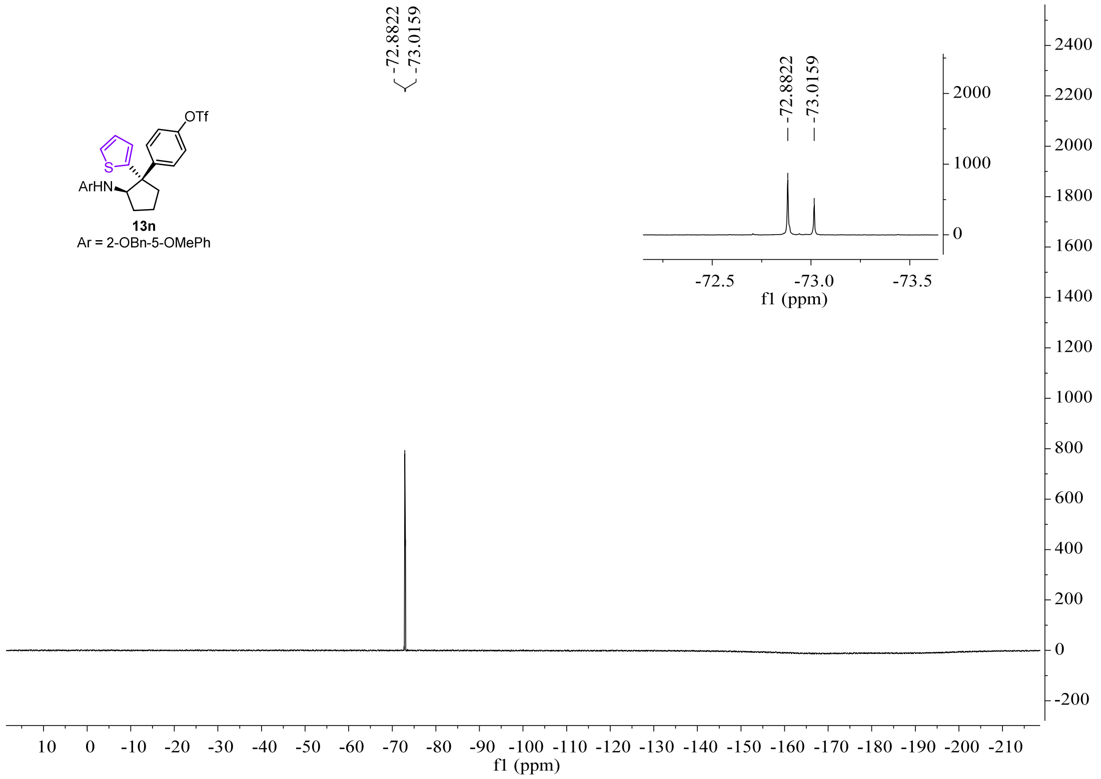
-72.8822
-73.0159
OTf
S
ArHN
13n
Ar = 2-OBn-5-OMePh
f1 (ppm)

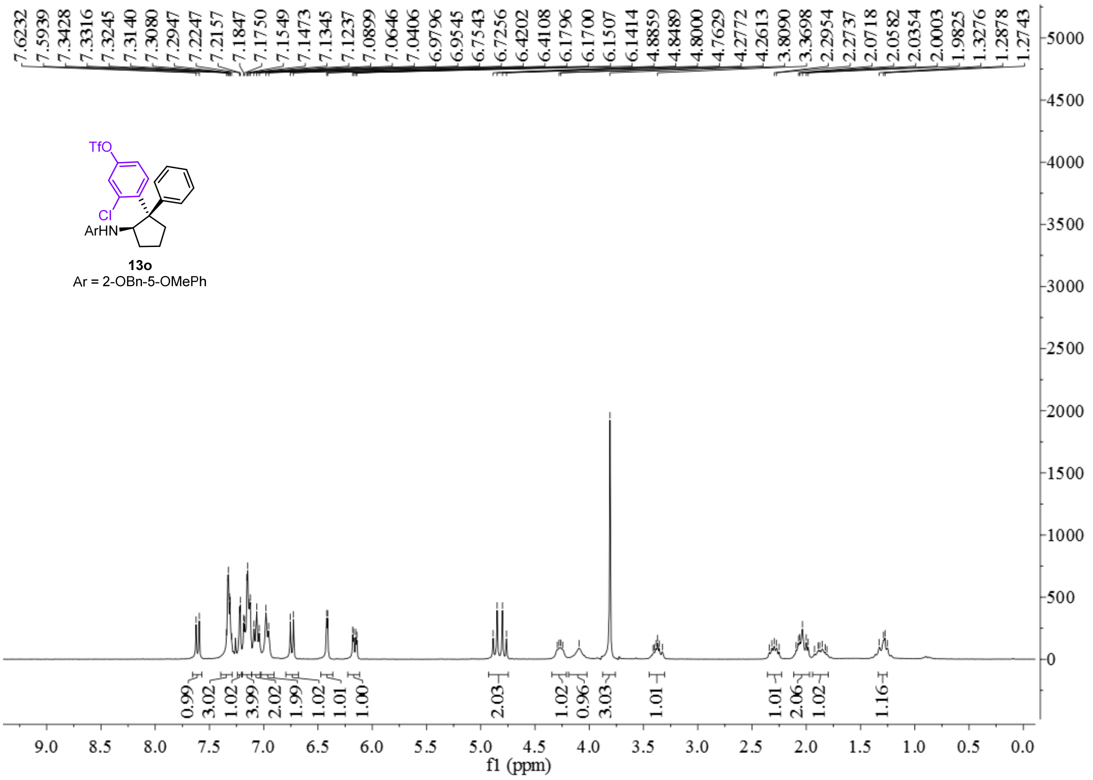
7.6232
7.5939
7.3428
7.3316
7.3245
7.3140
7.3080
7.2947
7.2247
7.2157
7.1847
7.1750
7.1549
7.1473
7.1345
7.1237
7.0899
7.0646
7.0406
6.9796
6.9545
6.7543
6.7256
6.4202
6.4108
6.1796
6.1700
6.1507
6.1414
4.8859
4.8489
4.8000
4.7629
4.2772
4.2613
3.8090
3.3698
2.2954
2.2737
2.0718
2.0582
2.0354
2.0003
1.9825
1.3276
1.2878
1.2743
TfO
Cl
ArHN
13o
Ar = 2-OBn-5-OMePh
0.99
3.02
1.02
3.99
2.02
1.99
1.02
1.01
1.00
2.03
1.02
0.96
3.03
1.01
1.01
2.06
1.02
1.16
f1 (ppm)

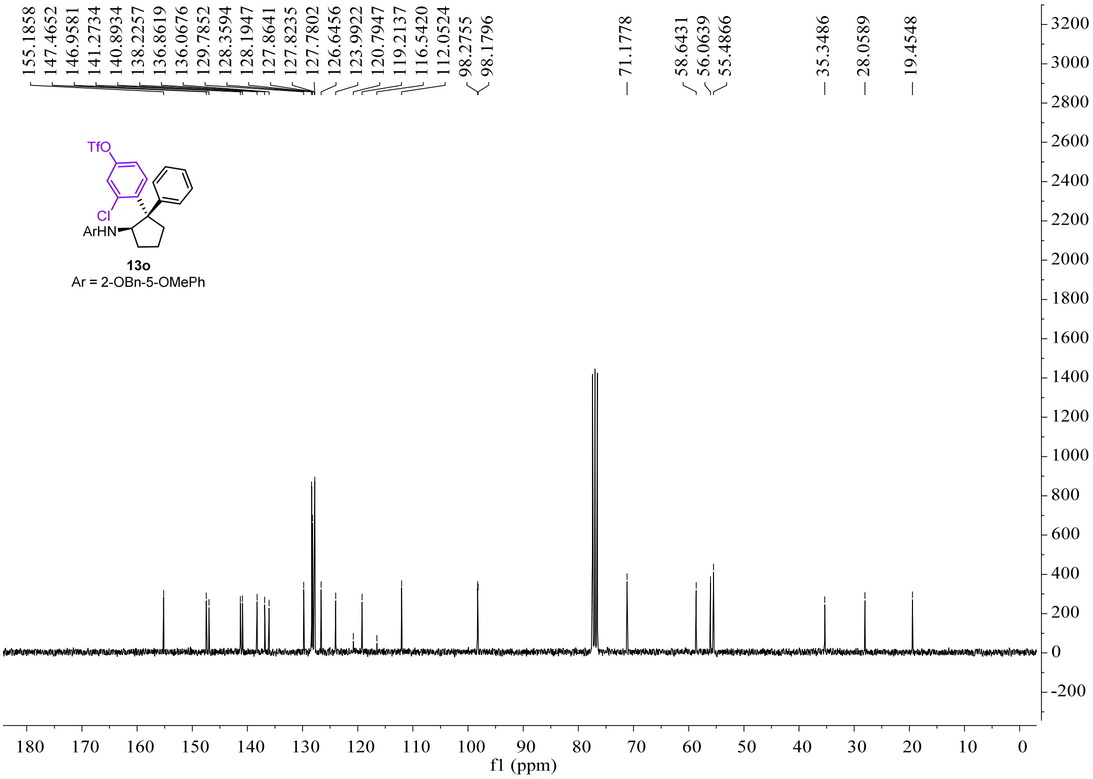
155.1858
147.4652
146.9581
141.2734
140.8934
138.2257
136.8619
136.0676
129.7852
128.3594
128.1947
127.8641
127.8235
127.7802
126.6456
123.9922
120.7947
119.2137
116.5420
112.0524
98.2755
98.1796
71.1778
58.6431
56.0639
55.4866
35.3486
28.0589
19.4548
TfO
Cl
ArHN
13o
Ar = 2-OBn-5-OMePh
f1 (ppm)

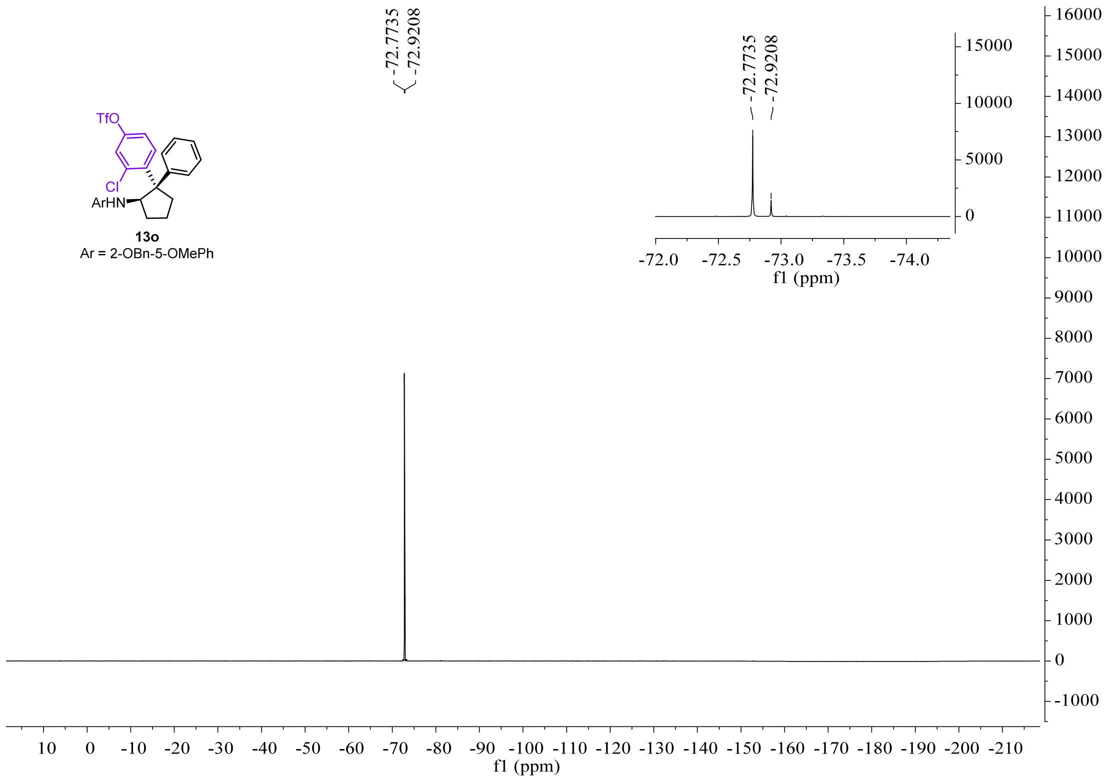
TfO
Cl
ArHN
13o
Ar = 2-OBn-5-OMePh
-72.7735
-72.9208
f1 (ppm)

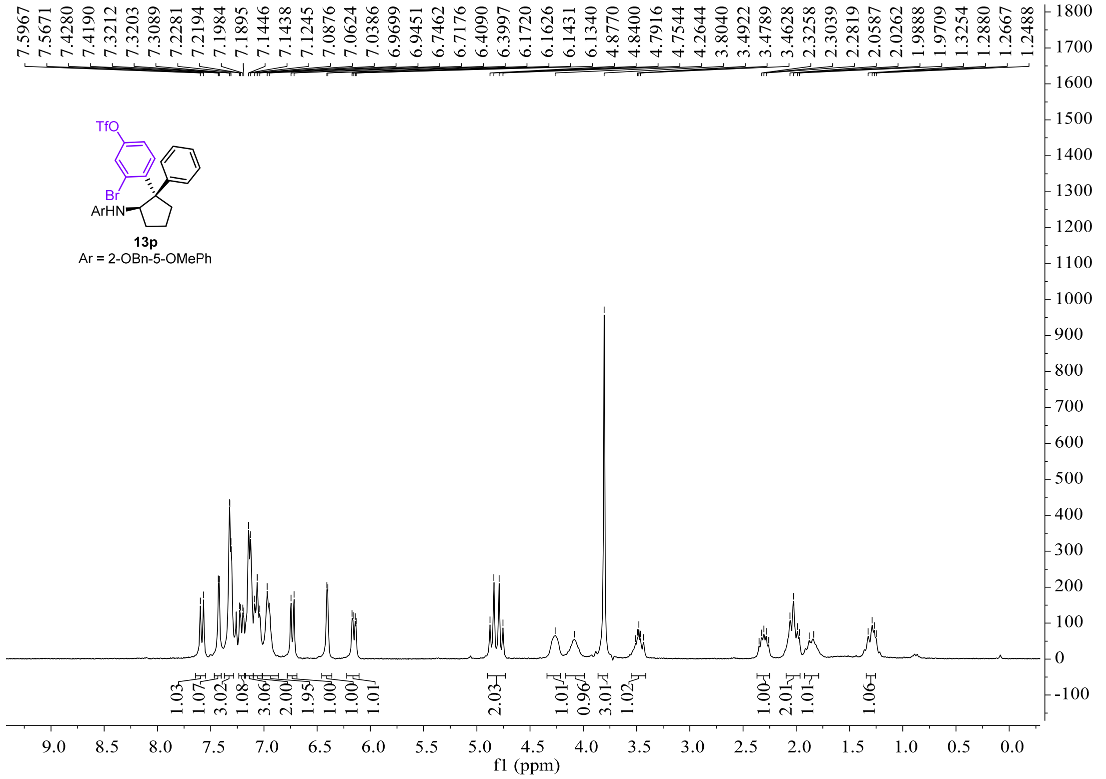
TfO
Br
ArHN
13p
Ar = 2-OBn-5-OMePh
f1 (ppm)

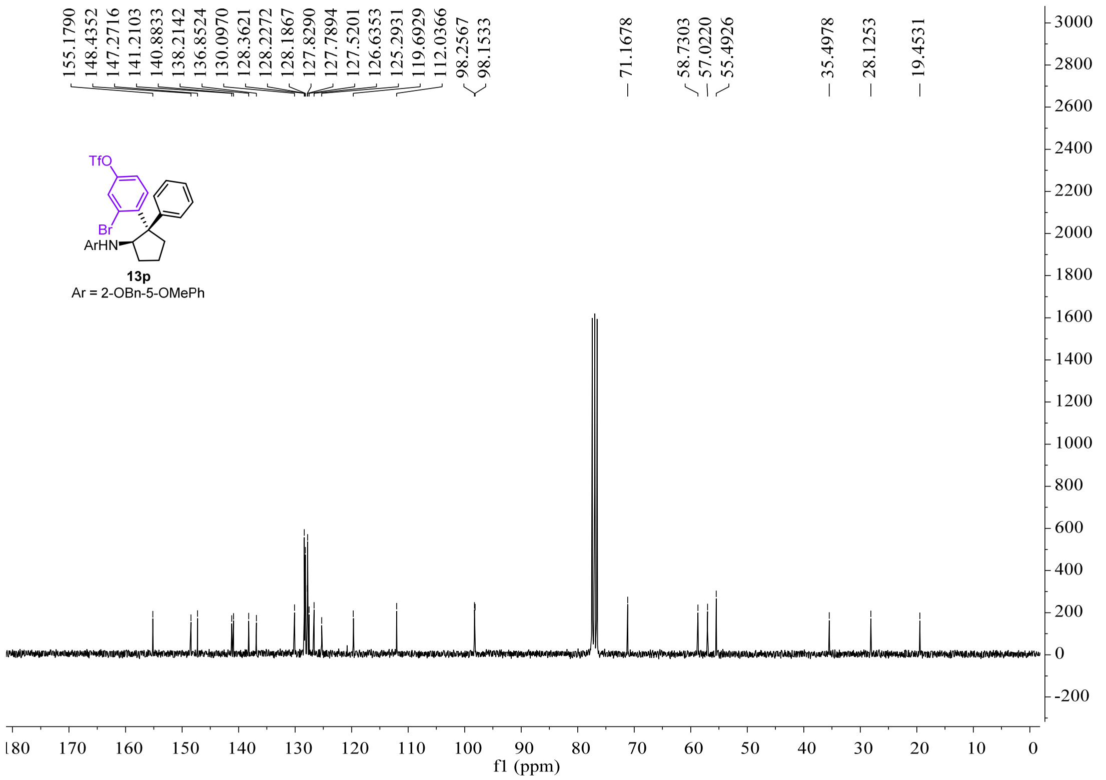
155.1790
148.4352
147.2716
141.2103
140.8833
138.2142
136.8524
130.0970
128.3621
128.2272
128.1867
127.8290
127.7894
127.5201
126.6353
125.2931
119.6929
112.0366
98.2567
98.1533
71.1678
58.7303
57.0220
55.4926
35.4978
28.1253
19.4531
TfO
Br
ArHN
13p
Ar = 2-OBn-5-OMePh
f1 (ppm)

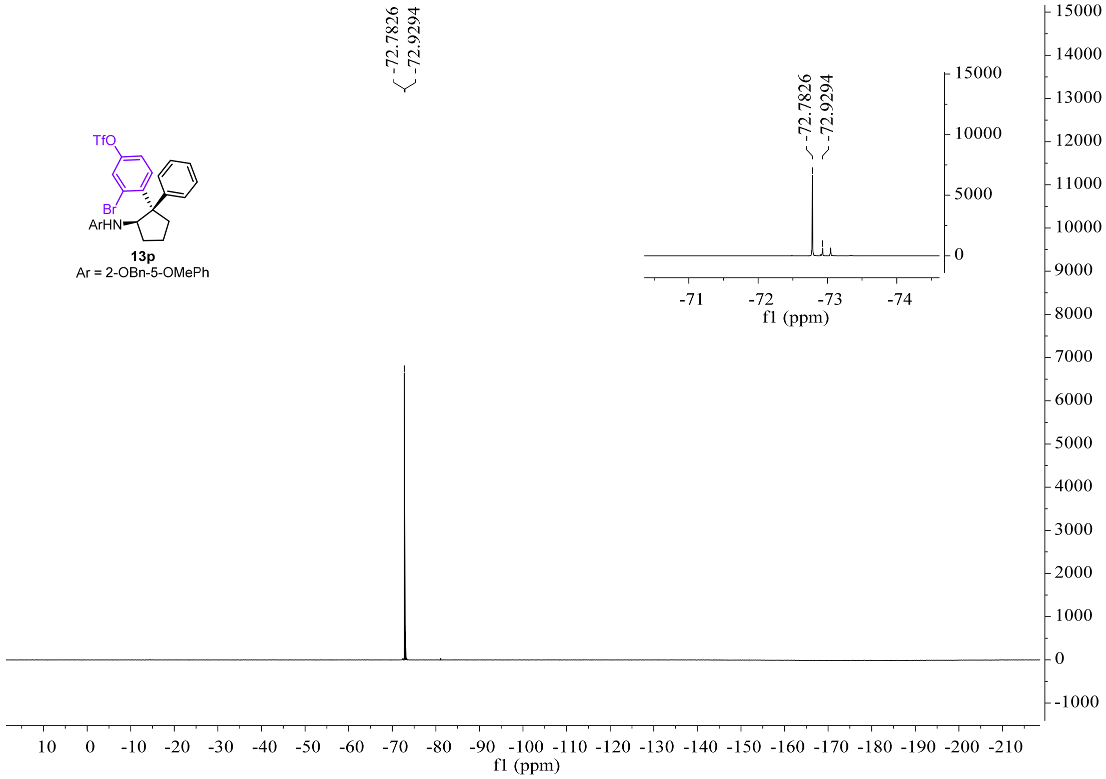
-72.7826
-72.9294
TfO
Br
ArHN
13p
Ar = 2-OBn-5-OMePh
f1 (ppm)

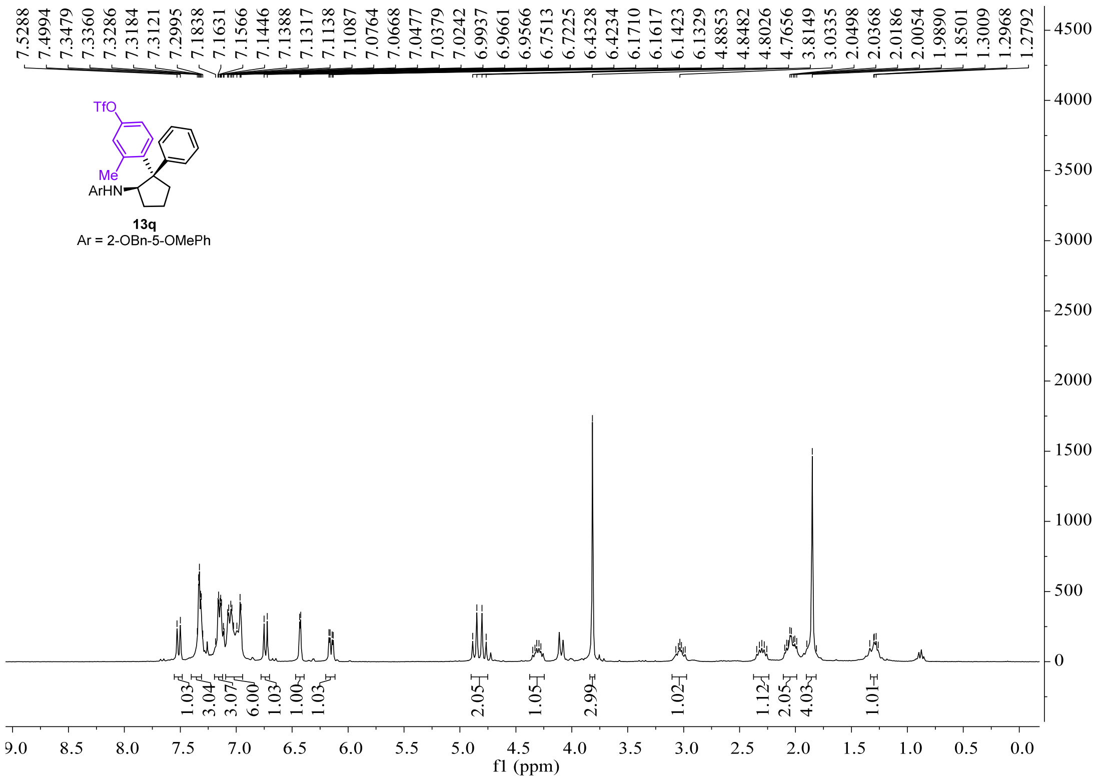
7.5288
7.4994
7.3479
7.3360
7.3286
7.3184
7.3121
7.2995
7.1838
7.1631
7.1566
7.1446
7.1388
7.1317
7.1138
7.1087
7.0764
7.0668
7.0477
7.0379
7.0242
6.9937
6.9661
6.9566
6.7513
6.7225
6.4328
6.4234
6.1710
6.1617
6.1423
6.1329
4.8853
4.8482
4.8026
4.7656
3.8149
3.0335
2.0498
2.0368
2.0186
2.0054
1.9890
1.8501
1.3009
1.2968
1.2792
TfO
Me
ArHN
13q
Ar = 2-OBn-5-OMePh
1.03
3.04
3.07
6.00
1.03
1.00
1.03
2.05
1.05
2.99
1.02
1.12
2.05
4.03
1.01
f1 (ppm)

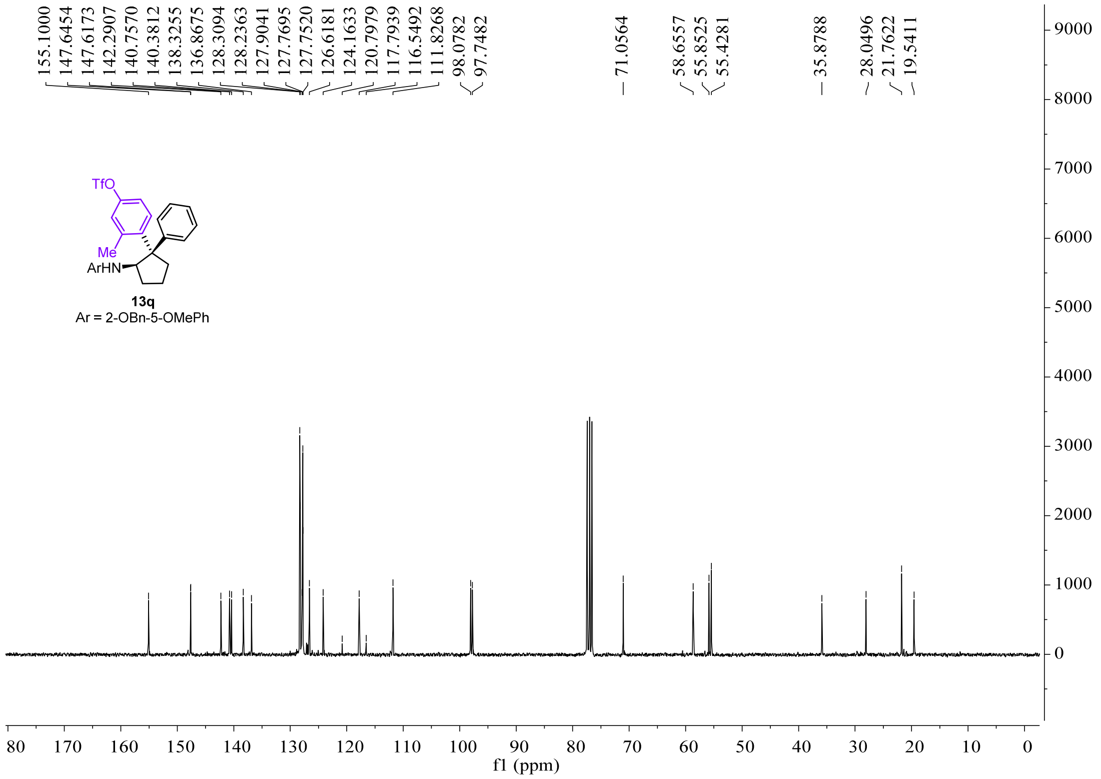
155.1000
147.6454
147.6173
142.2907
140.7570
140.3812
138.3255
136.8675
128.3094
128.2363
127.9041
127.7695
127.7520
126.6181
124.1633
120.7979
117.7939
116.5492
111.8268
98.0782
97.7482
71.0564
58.6557
55.8525
55.4281
35.8788
28.0496
21.7622
19.5411
TfO
Me
ArHN
13q
Ar = 2-OBn-5-OMePh
f1 (ppm)

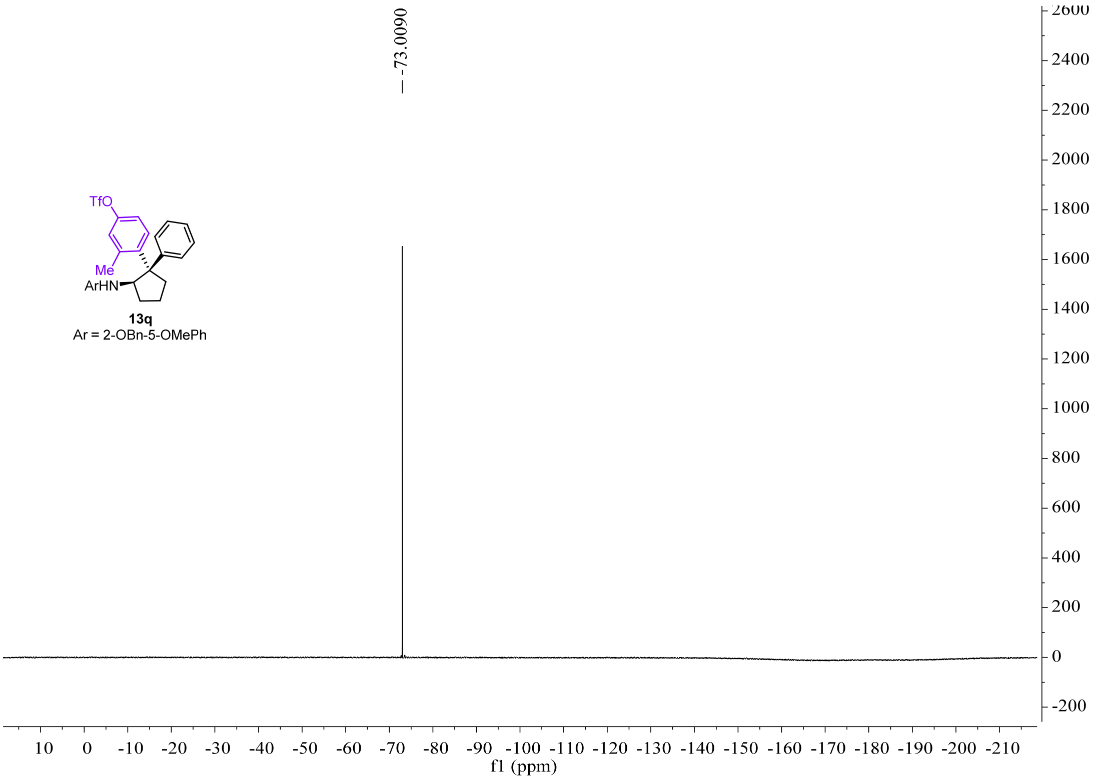
-73.0090
TfO
Me
ArHN
13q
Ar = 2-OBn-5-OMePh
f1 (ppm)

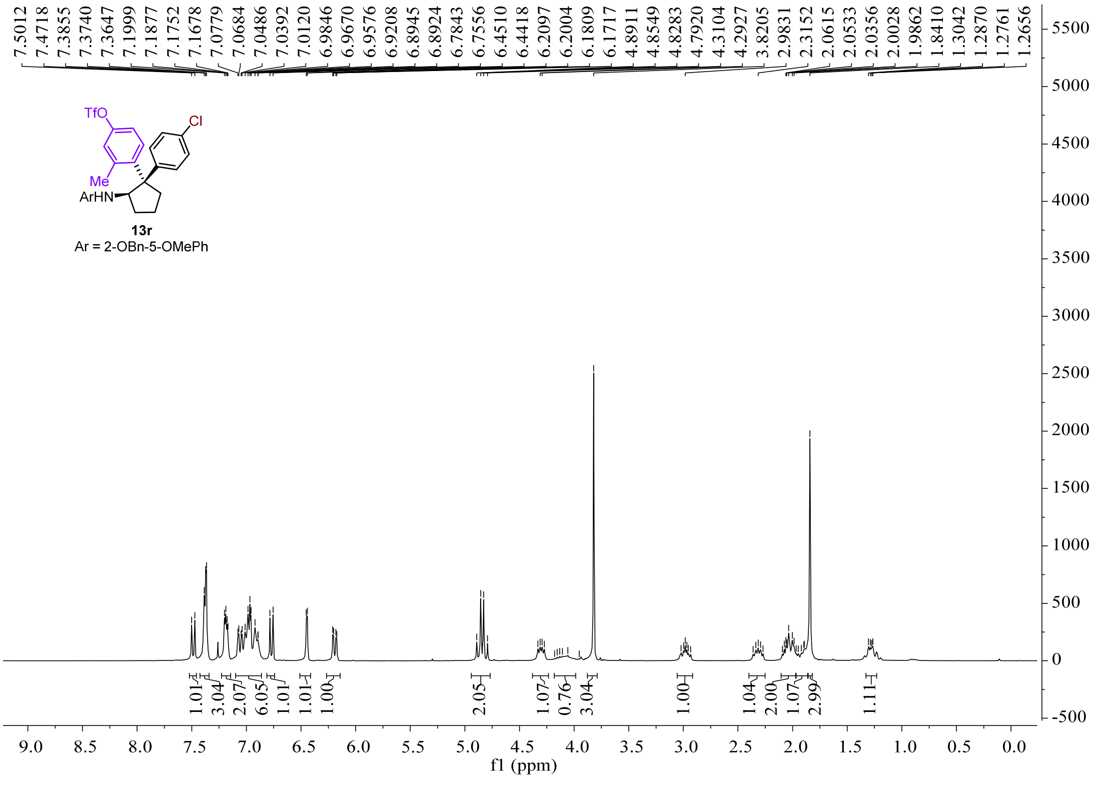
7.5012
7.4718
7.3855
7.3740
7.3647
7.1999
7.1877
7.1752
7.1678
7.0779
7.0684
7.0486
7.0392
7.0120
6.9846
6.9670
6.9576
6.9208
6.8945
6.8924
6.7843
6.7556
6.4510
6.4418
6.2097
6.2004
6.1809
6.1717
4.8911
4.8549
4.8283
4.7920
4.3104
4.2927
3.8205
2.9831
2.3152
2.0615
2.0533
2.0356
2.0028
1.9862
1.8410
1.3042
1.2870
1.2761
1.2656
TfO
Cl
Me
ArHN
13r
Ar = 2-OBn-5-OMePh
1.01
3.04
2.07
6.05
1.01
1.01
1.00
2.05
1.07
0.76
3.04
1.00
1.04
2.00
1.07
2.99
1.11
f1 (ppm)

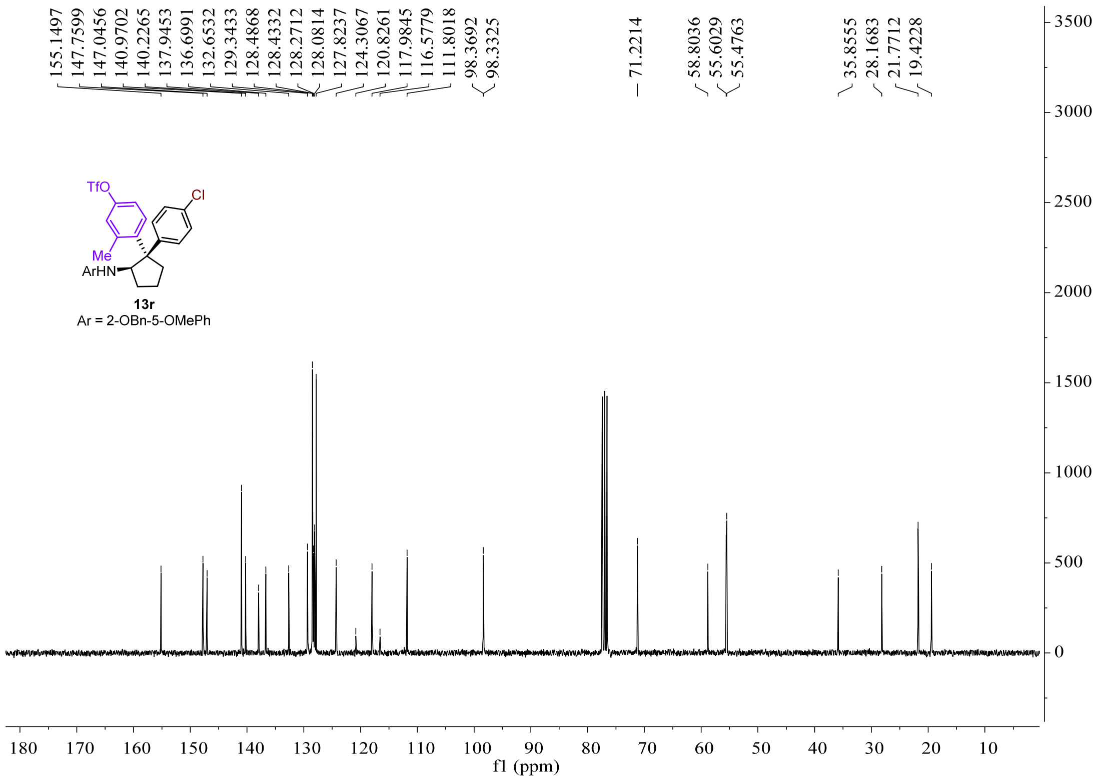
155.1497
147.7599
147.0456
140.9702
140.2265
137.9453
136.6991
132.6532
129.3433
128.4868
128.4332
128.2712
128.0814
127.8237
124.3067
120.8261
117.9845
116.5779
111.8018
98.3692
98.3325
71.2214
58.8036
55.6029
55.4763
35.8555
28.1683
21.7712
19.4228
TfO
Cl
Me
ArHN
13r
Ar = 2-OBn-5-OMePh
f1 (ppm)

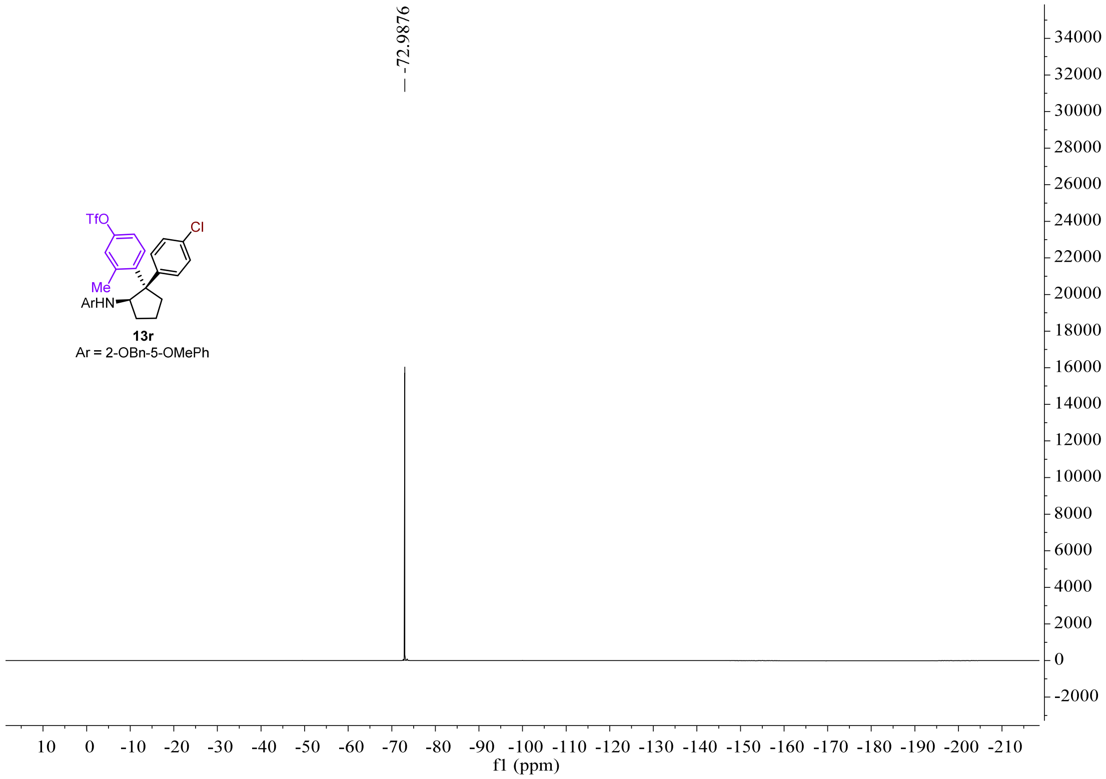
-72.9876
TfO
Cl
Me
ArHN
13r
Ar = 2-OBn-5-OMePh
f1 (ppm)

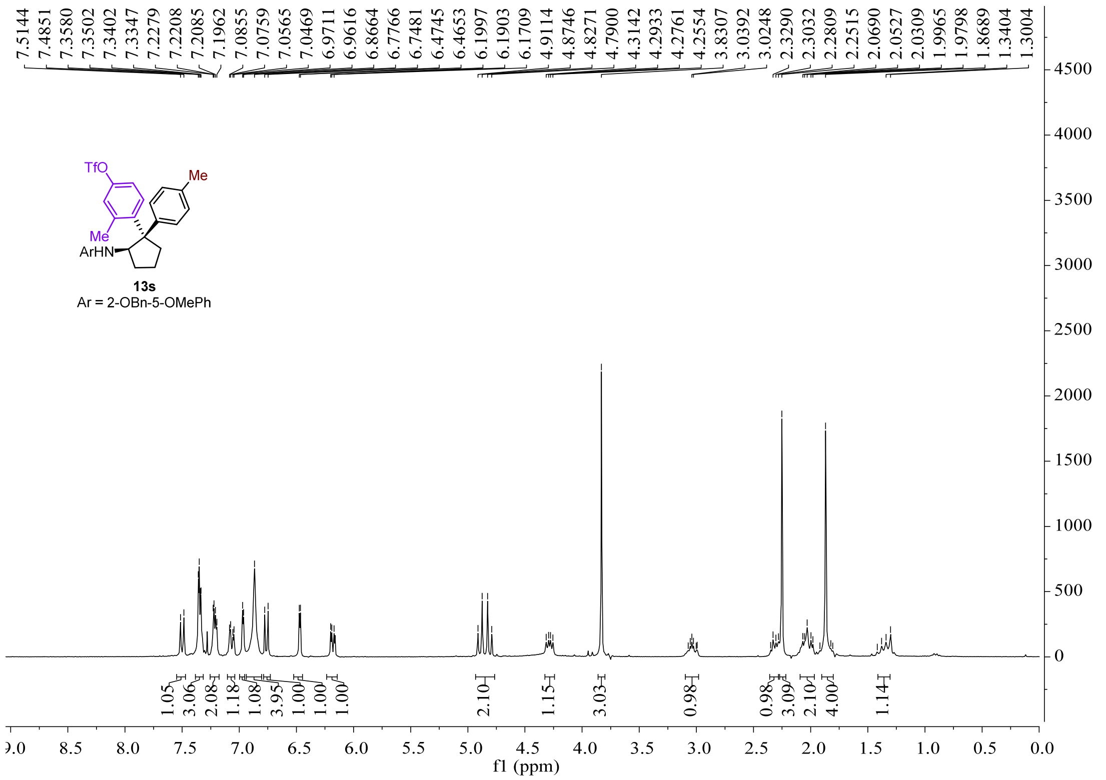
7.5144
7.4851
7.3580
7.3502
7.3402
7.3347
7.2279
7.2208
7.2085
7.1962
7.0855
7.0759
7.0565
7.0469
6.9711
6.9616
6.8664
6.7766
6.7481
6.4745
6.4653
6.1997
6.1903
6.1709
4.9114
4.8746
4.8271
4.7900
4.3142
4.2933
4.2761
4.2554
3.8307
3.0392
3.0248
2.3290
2.3032
2.2809
2.2515
2.0690
2.0527
2.0309
1.9965
1.9798
1.8689
1.3404
1.3004
TfO
Me
Me
ArHN
13s
Ar = 2-OBn-5-OMePh
1.05
3.06
2.08
1.18
1.08
3.95
1.00
1.00
1.00
2.10
1.15
3.03
0.98
0.98
3.09
2.10
4.00
1.14
f1 (ppm)
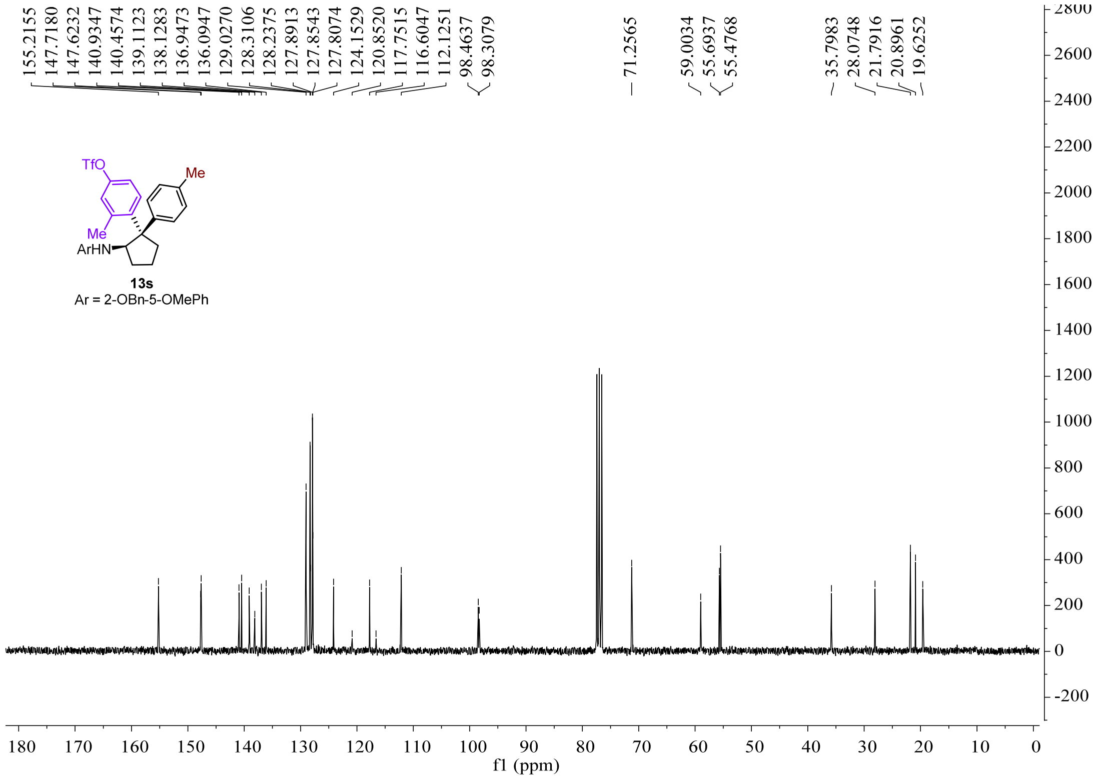
155.2155
147.7180
147.6232
140.9347
140.4574
139.1123
138.1283
136.9473
136.0947
129.0270
128.3106
128.2375
127.8913
127.8543
127.8074
124.1529
120.8520
117.7515
116.6047
112.1251
98.4637
98.3079
71.2565
59.0034
55.6937
55.4768
35.7983
28.0748
21.7916
20.8961
19.6252
TfO
Me
Me
ArHN
13s
Ar = 2-OBn-5-OMePh
f1 (ppm)

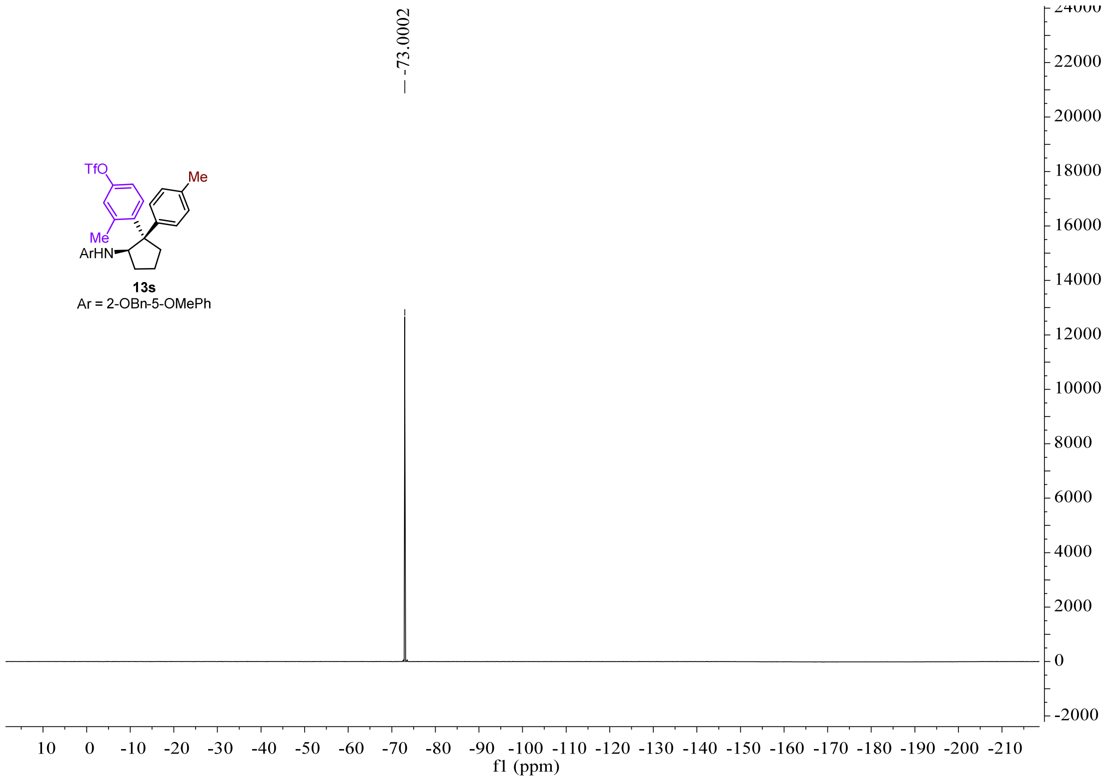
-73.0002
TfO
Me
Me
ArHN
13s
Ar = 2-OBn-5-OMePh
f1 (ppm)

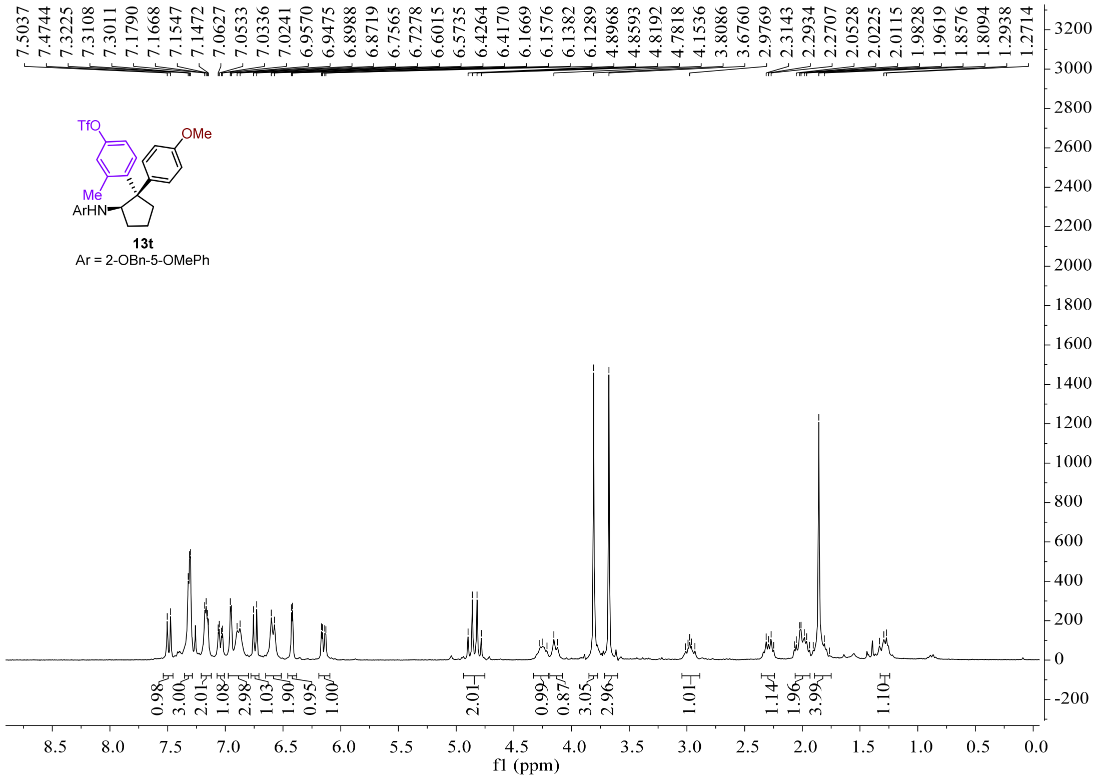
7.5037
7.4744
7.3225
7.3108
7.3011
7.1790
7.1668
7.1547
7.1472
7.0627
7.0533
7.0336
7.0241
6.9570
6.9475
6.8988
6.8719
6.7565
6.7278
6.6015
6.5735
6.4264
6.4170
6.1669
6.1576
6.1382
6.1289
4.8968
4.8593
4.8192
4.7818
4.1536
3.8086
3.6760
2.9769
2.3143
2.2934
2.2707
2.0528
2.0225
2.0115
1.9828
1.9619
1.8576
1.8094
1.2938
1.2714
TfO
OMe
Me
ArHN
13t
Ar = 2-OBn-5-OMePh
0.98
3.00
2.01
1.08
2.98
1.03
1.90
0.95
1.00
2.01
0.99
0.87
3.05
2.96
1.01
1.14
1.96
3.99
1.10
f1 (ppm)

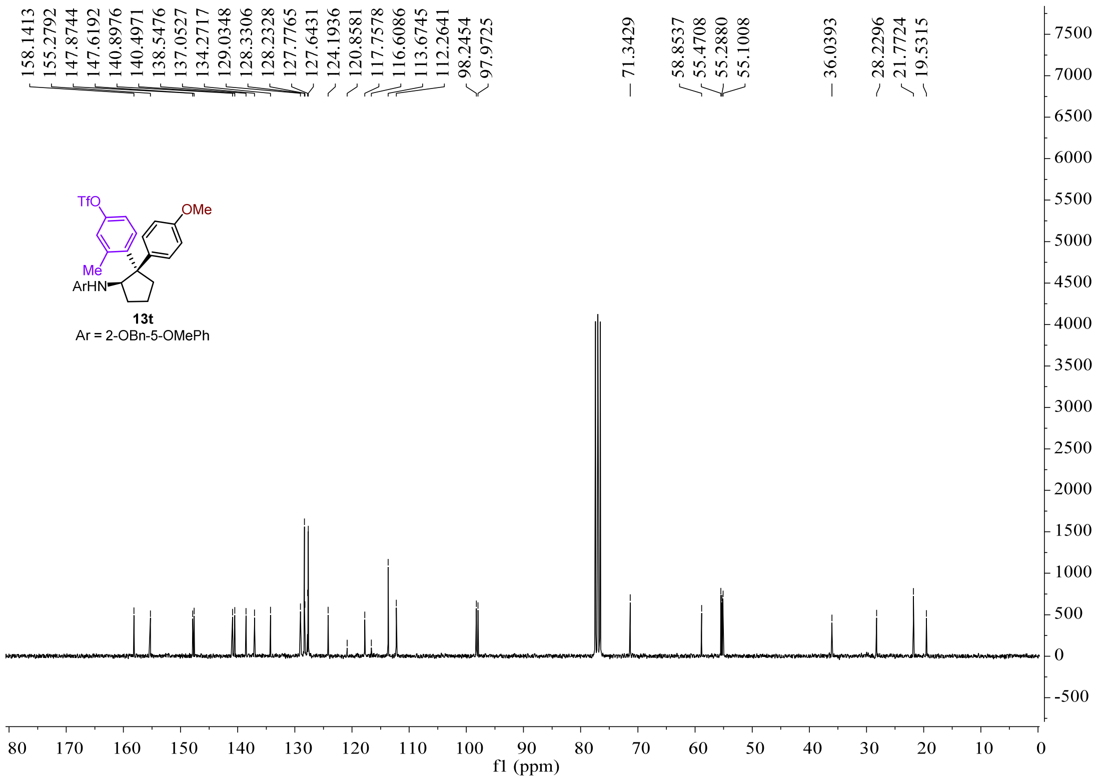
158.1413
155.2792
147.8744
147.6192
140.8976
140.4971
138.5476
137.0527
134.2717
129.0348
128.3306
128.2328
127.7765
127.6431
124.1936
120.8581
117.7578
116.6086
113.6745
112.2641
98.2454
97.9725
71.3429
58.8537
55.4708
55.2880
55.1008
36.0393
28.2296
21.7724
19.5315
TfO
OMe
Me
ArHN
13t
Ar = 2-OBn-5-OMePh
f1 (ppm)

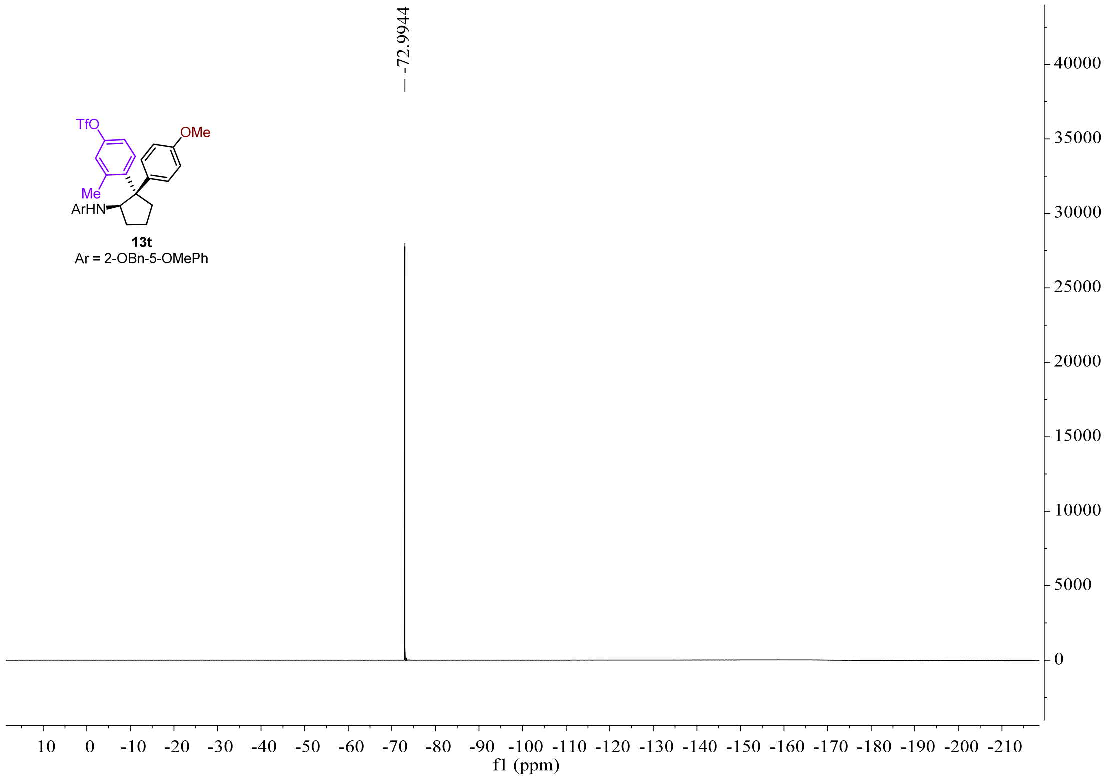
-72.9944
TfO
OMe
Me
ArHN
13t
Ar = 2-OBn-5-OMePh
f1 (ppm)

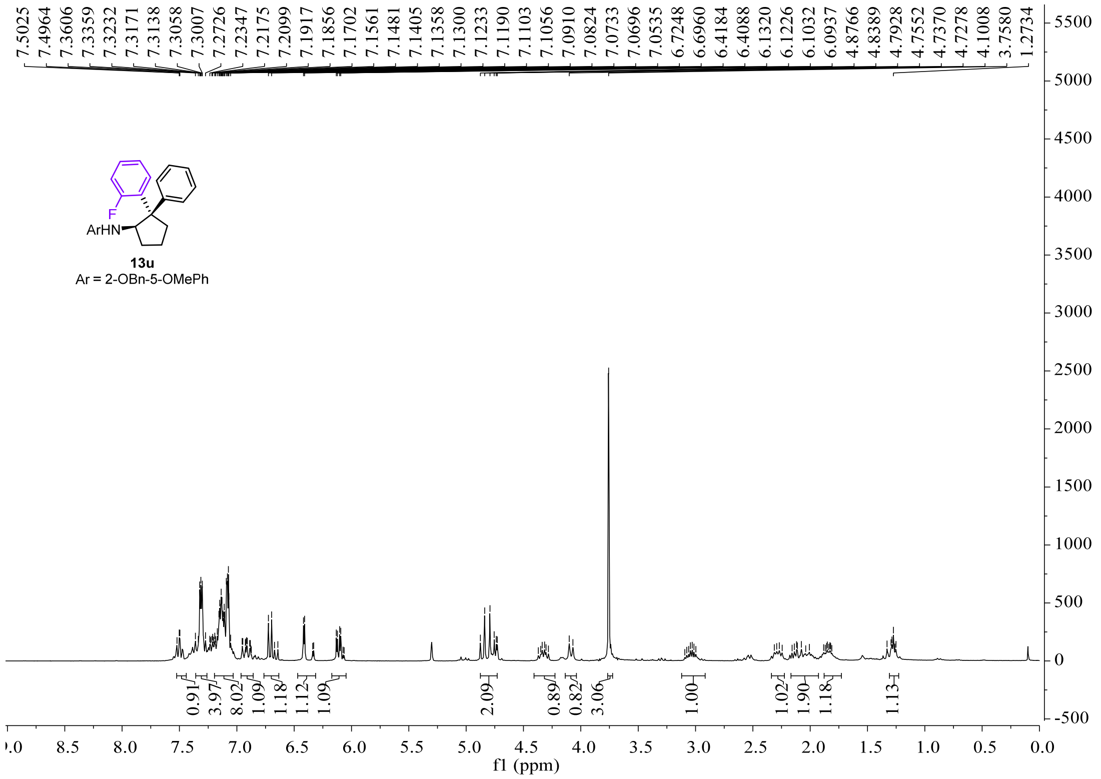
7.5025
7.4964
7.3606
7.3359
7.3232
7.3171
7.3138
7.3058
7.3007
7.2726
7.2347
7.2175
7.2099
7.1917
7.1856
7.1702
7.1561
7.1481
7.1405
7.1358
7.1300
7.1233
7.1190
7.1103
7.1056
7.0910
7.0824
7.0733
7.0696
7.0535
6.7248
6.6960
6.4184
6.4088
6.1320
6.1226
6.1032
6.0937
4.8766
4.8389
4.7928
4.7552
4.7370
4.7278
4.1008
3.7580
1.2734
F
ArHN
13u
Ar = 2-OBn-5-OMePh
0.91
3.97
8.02
1.09
1.18
1.12
1.09
2.09
0.89
0.82
3.06
1.00
1.02
1.90
1.18
1.13
f1 (ppm)

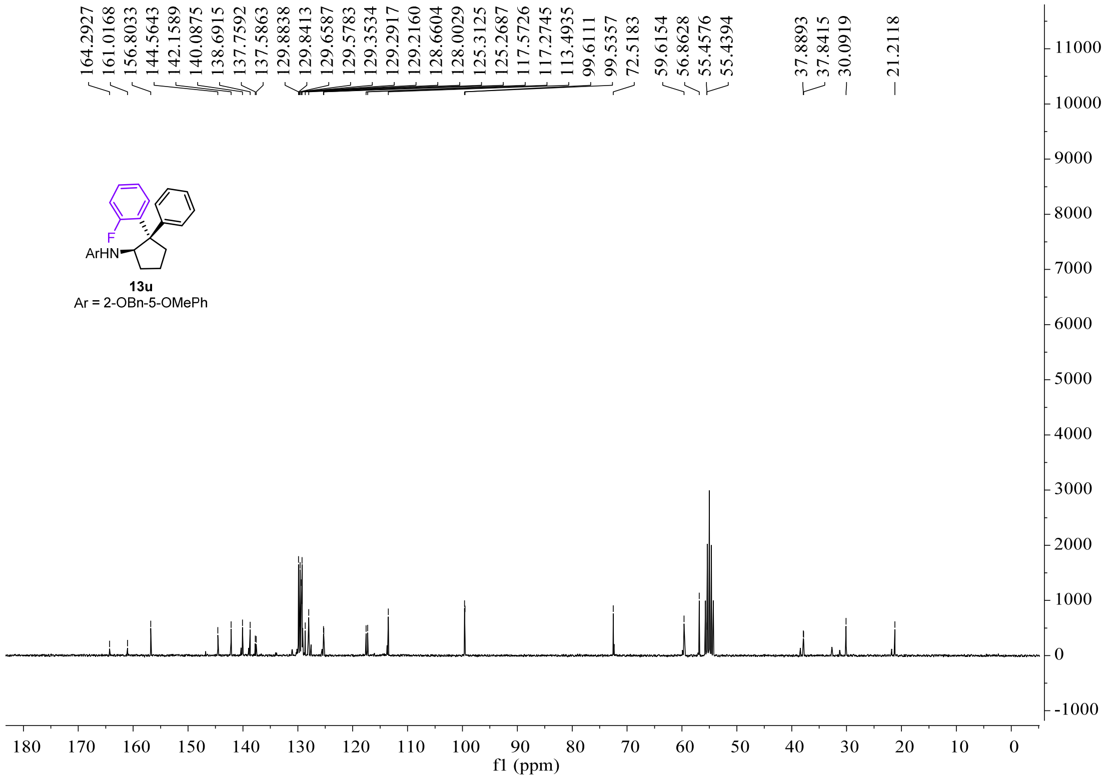
164.2927
161.0168
156.8033
144.5643
142.1589
140.0875
138.6915
137.7592
137.5863
129.8838
129.8413
129.6587
129.5783
129.3534
129.2917
129.2160
128.6604
128.0029
125.3125
125.2687
117.5726
117.2745
113.4935
99.6111
99.5357
72.5183
59.6154
56.8628
55.4576
55.4394
37.8893
37.8415
30.0919
21.2118
F
ArHN
13u
Ar = 2-OBn-5-OMePh
f1 (ppm)

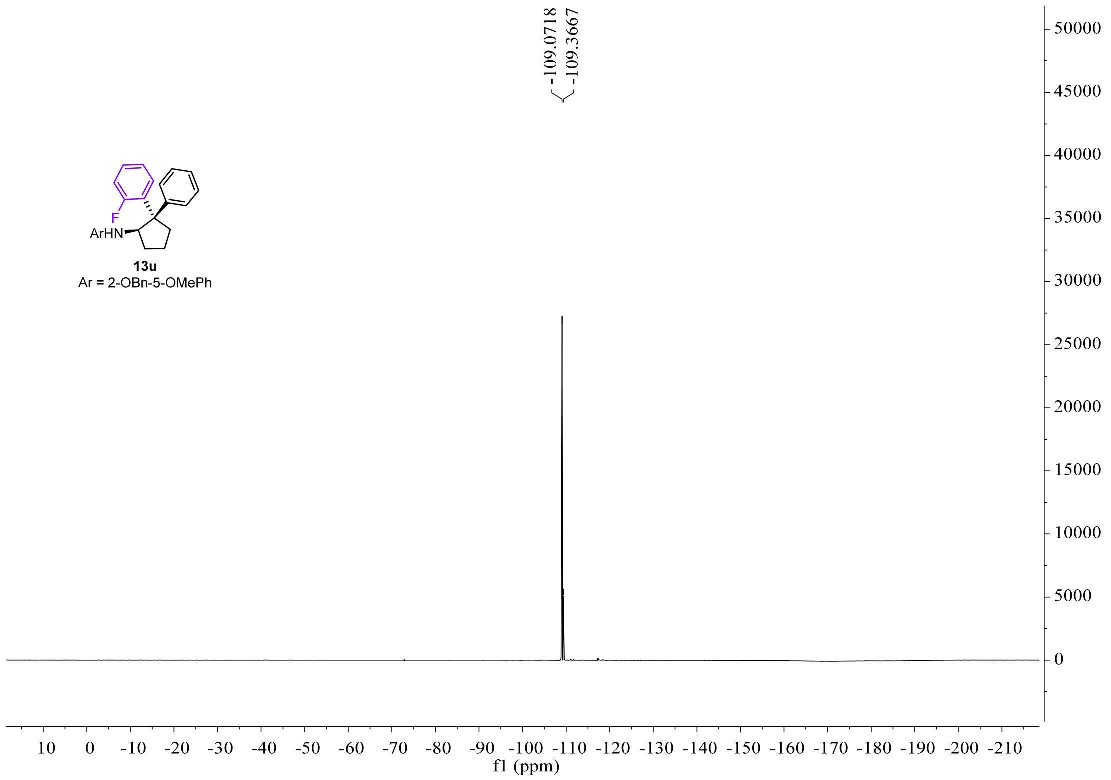
-109.0718
-109.3667
F
ArHN
13u
Ar = 2-OBn-5-OMePh
f1 (ppm)

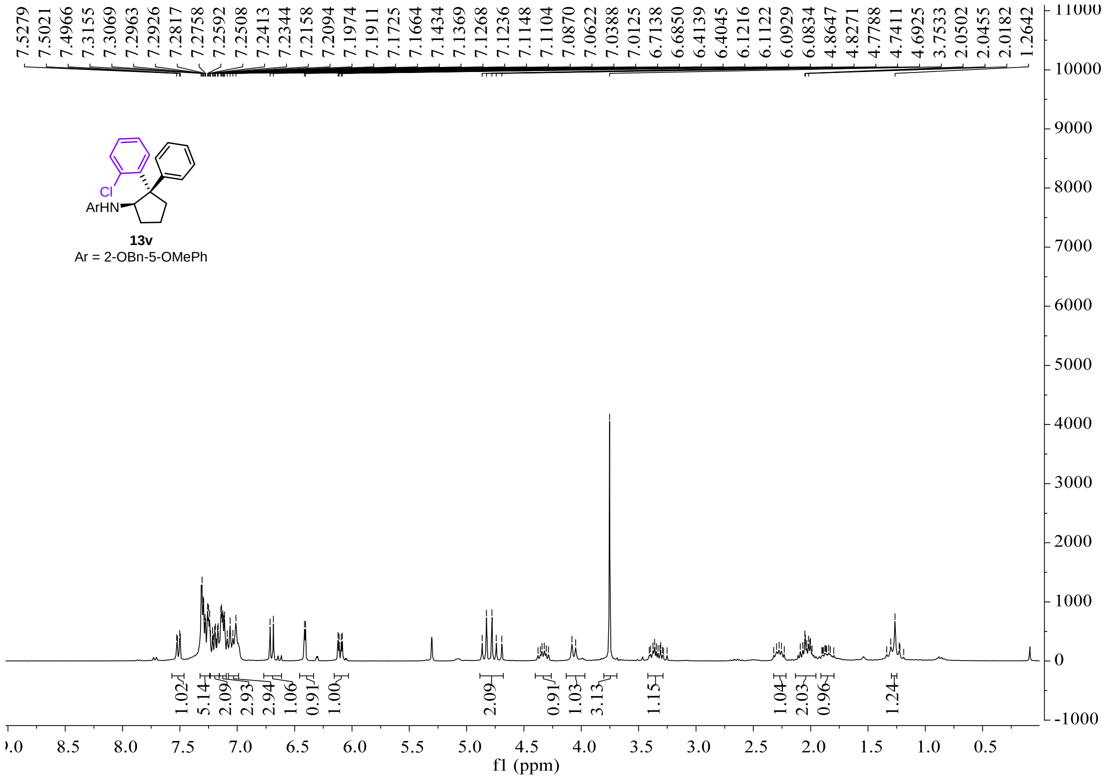
7.5279
7.5021
7.4966
7.3155
7.3069
7.2963
7.2926
7.2817
7.2758
7.2592
7.2508
7.2413
7.2344
7.2158
7.2094
7.1974
7.1911
7.1725
7.1664
7.1434
7.1369
7.1268
7.1236
7.1148
7.1104
7.0870
7.0622
7.0388
7.0125
6.7138
6.6850
6.4139
6.4045
6.1216
6.1122
6.0929
6.0834
4.8647
4.8271
4.7788
4.7411
4.6925
3.7533
2.0502
2.0455
2.0182
1.2642
Cl
ArHN
13v
Ar = 2-OBn-5-OMePh
1.02
5.14
2.09
2.93
2.94
1.06
0.91
1.00
2.09
0.91
1.03
3.13
1.15
1.04
2.03
0.96
1.24
f1 (ppm)

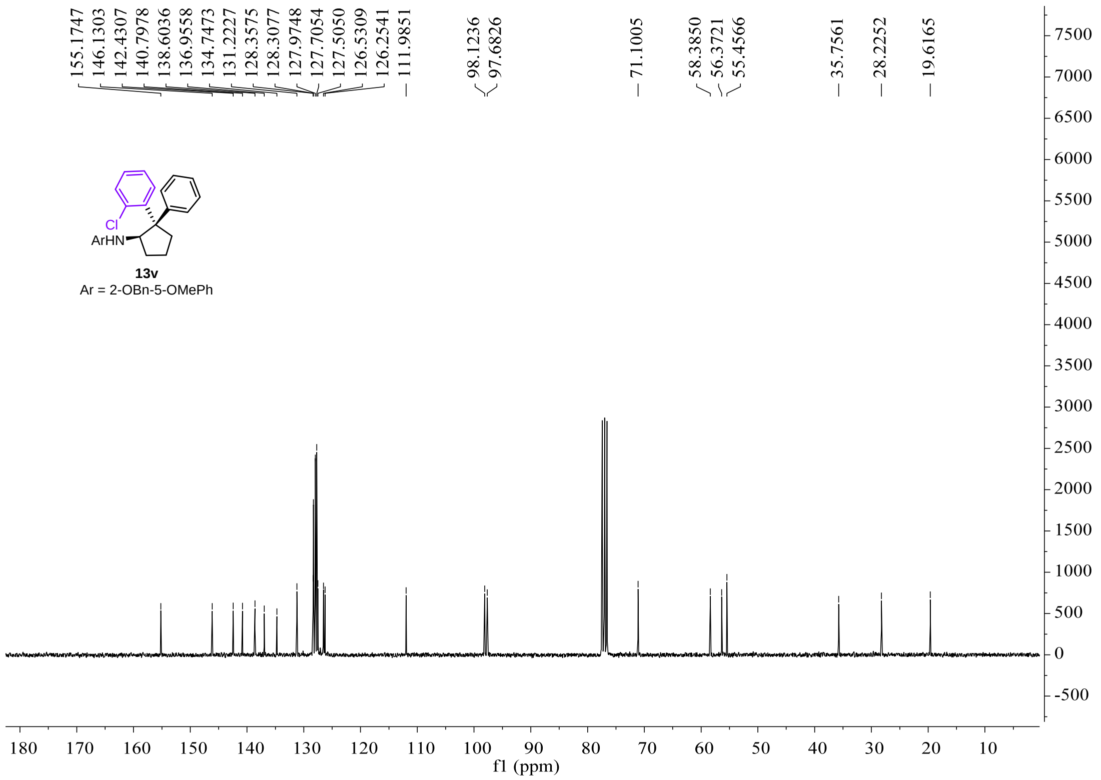
155.1747
146.1303
142.4307
140.7978
138.6036
136.9558
134.7473
131.2227
128.3575
128.3077
127.9748
127.7054
127.5050
126.5309
126.2541
111.9851
98.1236
97.6826
71.1005
58.3850
56.3721
55.4566
35.7561
28.2252
19.6165
Cl
ArHN
13v
Ar = 2-OBn-5-OMePh
f1 (ppm)

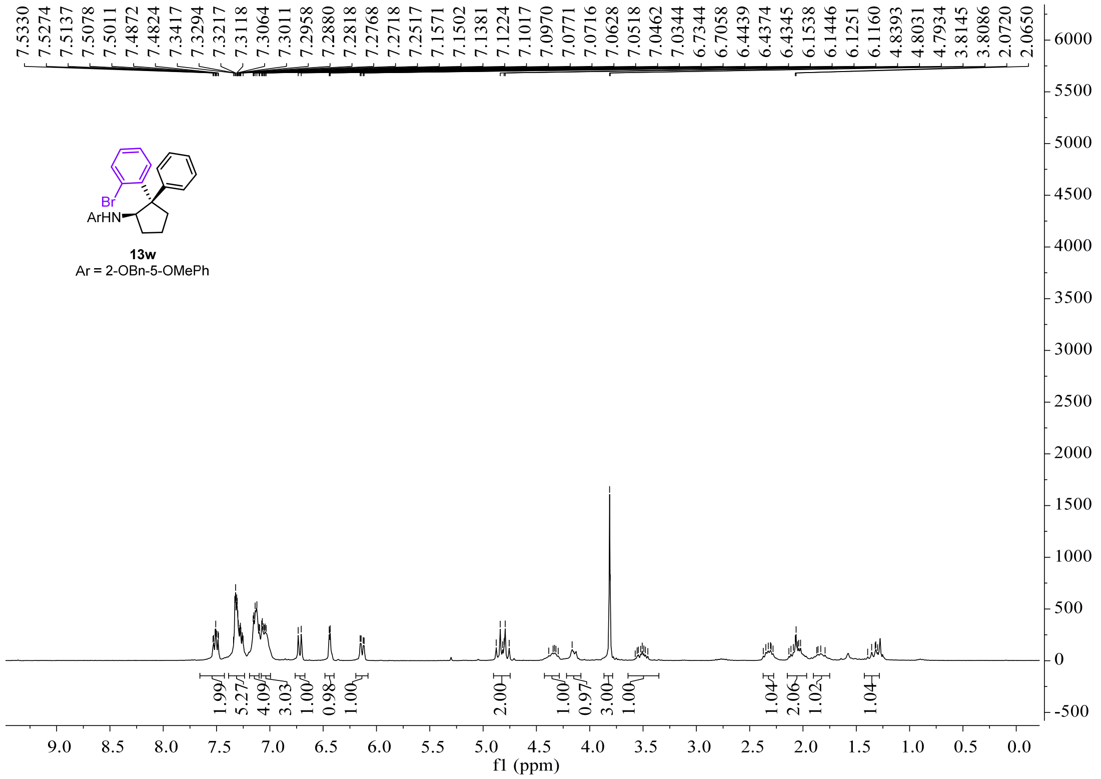
7.5330
7.5274
7.5137
7.5078
7.5011
7.4872
7.4824
7.3417
7.3294
7.3217
7.3118
7.3064
7.3011
7.2958
7.2880
7.2818
7.2768
7.2718
7.2517
7.1571
7.1502
7.1381
7.1224
7.1017
7.0970
7.0771
7.0716
7.0628
7.0518
7.0462
7.0344
6.7344
6.7058
6.4439
6.4374
6.4345
6.1538
6.1446
6.1251
6.1160
4.8393
4.8031
4.7934
3.8145
3.8086
2.0720
2.0650
Br
ArHN
13w
Ar = 2-OBn-5-OMePh
1.99
5.27
4.09
3.03
1.00
0.98
1.00
2.00
1.00
0.97
3.00
1.00
1.04
2.06
1.02
1.04
f1 (ppm)

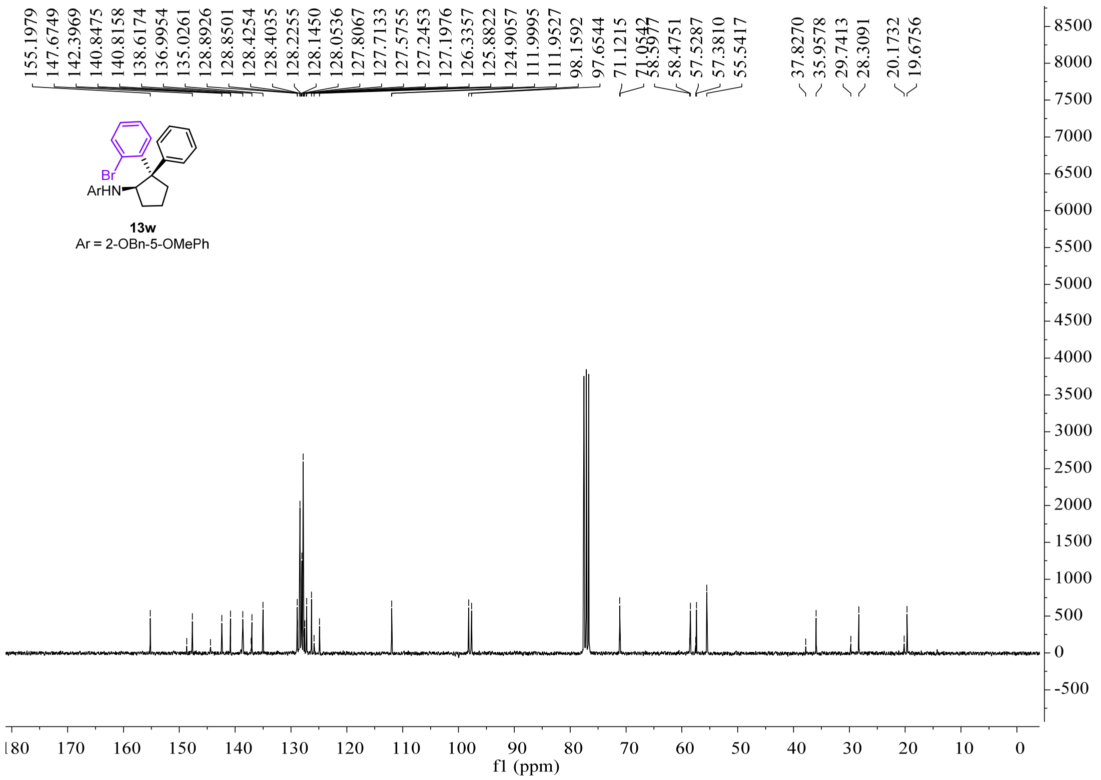
155.1979
147.6749
142.3969
140.8475
140.8158
138.6174
136.9954
135.0261
128.8926
128.8501
128.4254
128.4035
128.2255
128.1450
128.0536
127.8067
127.7133
127.5755
127.2453
127.1976
126.3357
125.8822
124.9057
111.9995
111.9527
98.1592
97.6544
71.1215
58.4751
57.5287
57.3810
55.5417
37.8270
35.9578
29.7413
28.3091
20.1732
19.6756
Br
ArHN
13w
Ar = 2-OBn-5-OMePh
f1 (ppm)

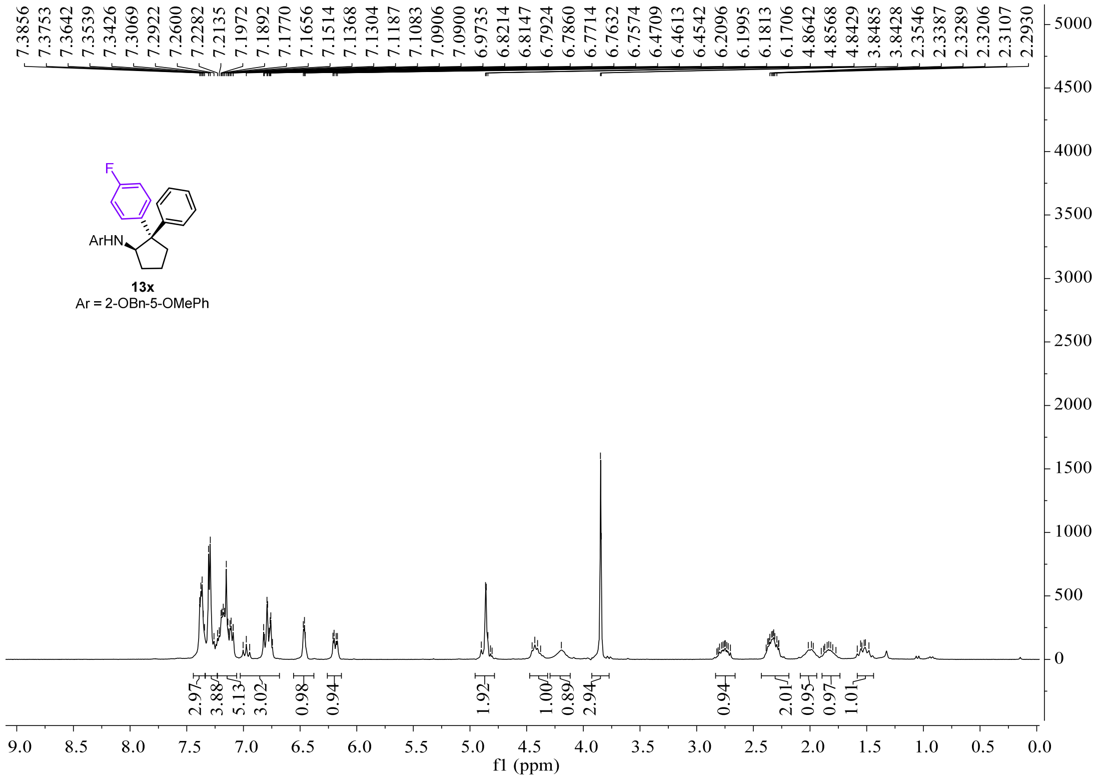
7.3856
7.3753
7.3642
7.3539
7.3426
7.3069
7.2922
7.2600
7.2282
7.2135
7.1972
7.1892
7.1770
7.1656
7.1514
7.1368
7.1304
7.1187
7.1083
7.0906
7.0900
6.9735
6.8214
6.8147
6.7924
6.7860
6.7714
6.7632
6.7574
6.4709
6.4613
6.4542
6.2096
6.1995
6.1813
6.1706
4.8642
4.8568
4.8429
3.8485
3.8428
2.3546
2.3387
2.3289
2.3206
2.3107
2.2930
F
ArHN
13x
Ar = 2-OBn-5-OMePh
f1 (ppm)

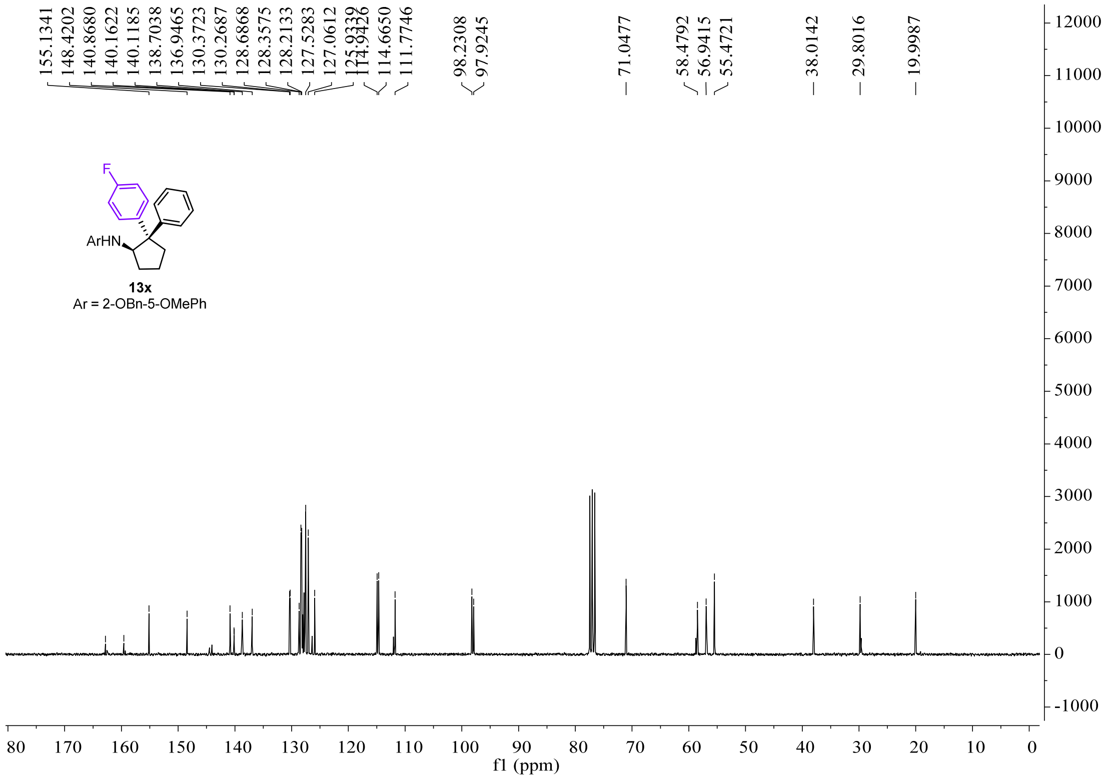
155.1341
148.4202
140.8680
140.1622
140.1185
138.7038
136.9465
130.3723
130.2687
128.6868
128.3575
128.2133
127.5283
127.0612
125.9339
114.9426
114.6650
111.7746
98.2308
97.9245
71.0477
58.4792
56.9415
55.4721
38.0142
29.8016
19.9987
F
ArHN
13x
Ar = 2-OBn-5-OMePh
12000
11000
10000
9000
8000
7000
6000
5000
4000
3000
2000
1000
0
-1000
80 170 160 150 140 130 120 110 100 90 80 70 60 50 40 30 20 10 0
f1 (ppm)

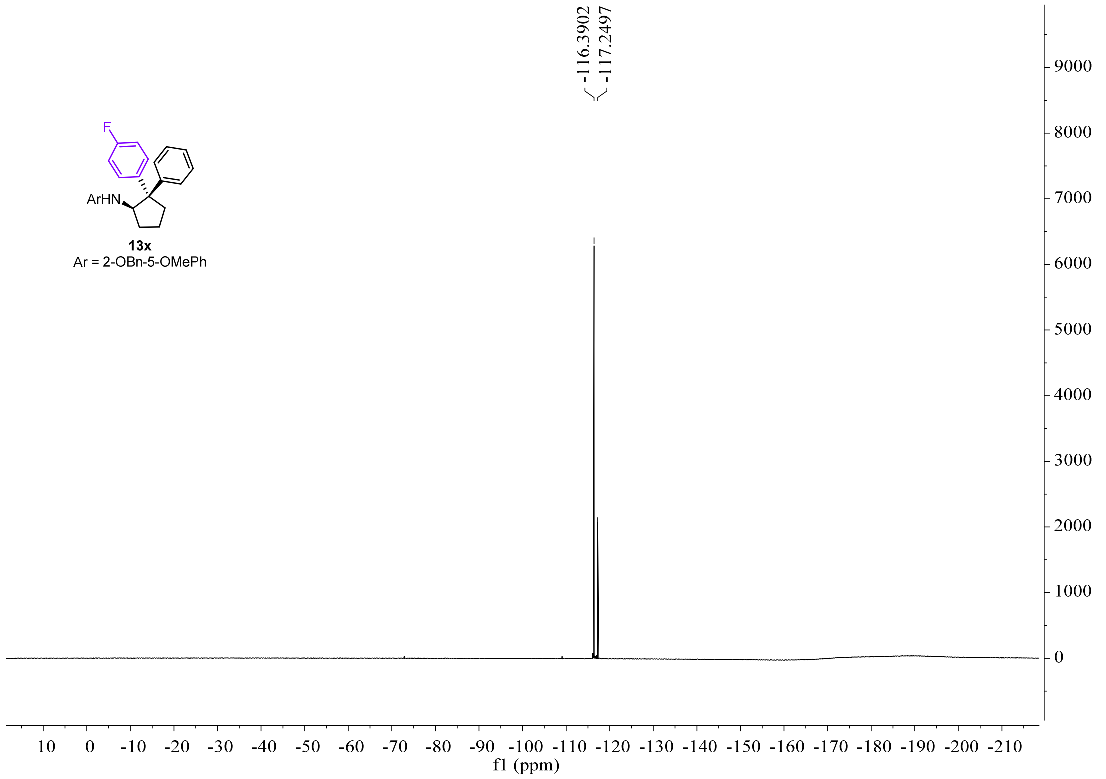
-116.3902
-117.2497
F
ArHN
13x
Ar = 2-OBn-5-OMePh
9000
8000
7000
6000
5000
4000
3000
2000
1000
0
10 0 -10 -20 -30 -40 -50 -60 -70 -80 -90 -100 -110 -120 -130 -140 -150 -160 -170 -180 -190 -200 -210
f1 (ppm)

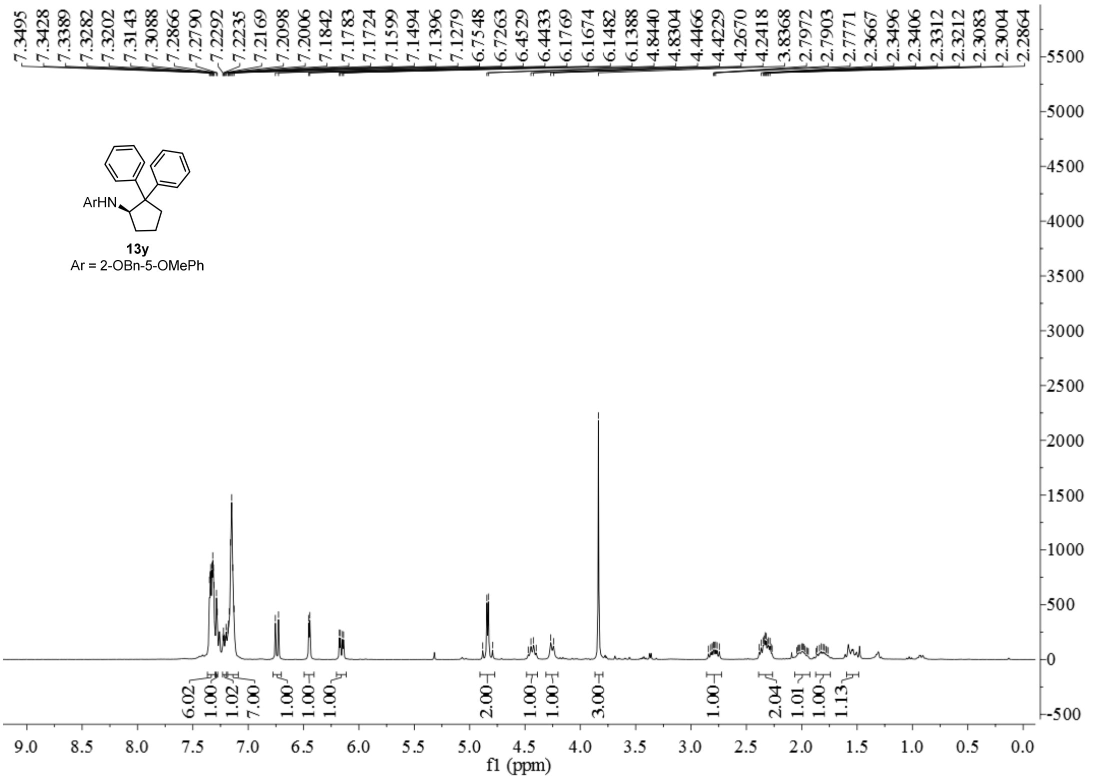
7.3495
7.3428
7.3389
7.3282
7.3202
7.3143
7.3088
7.2866
7.2790
7.2292
7.2235
7.2169
7.2098
7.2006
7.1842
7.1783
7.1724
7.1599
7.1494
7.1396
7.1279
6.7548
6.7263
6.4529
6.4433
6.1769
6.1674
6.1482
6.1388
4.8440
4.8304
4.4466
4.4229
4.2670
4.2418
3.8368
2.7972
2.7903
2.7771
2.3667
2.3496
2.3406
2.3312
2.3212
2.3083
2.3004
2.2864
ArHN
13y
Ar = 2-OBn-5-OMePh
6.02
1.00
1.02
7.00
1.00
1.00
1.00
2.00
1.00
1.00
3.00
1.00
2.04
1.01
1.00
1.13
f1 (ppm)

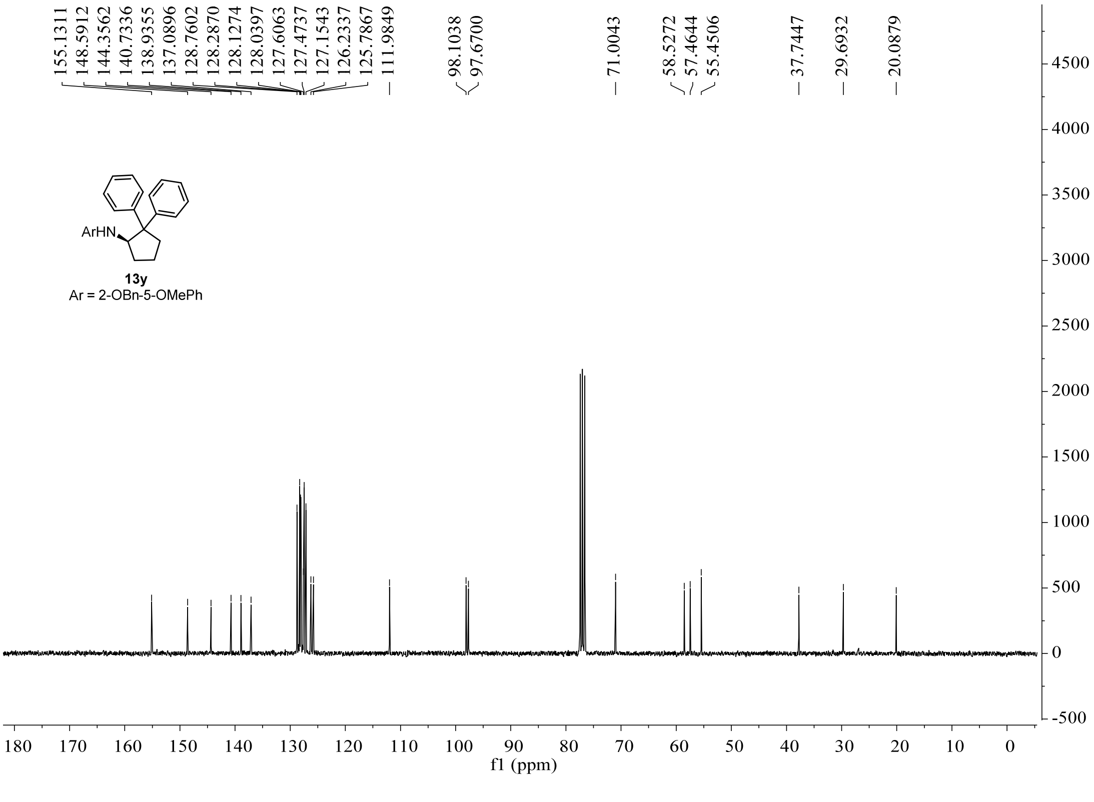
155.1311
148.5912
144.3562
140.7336
138.9355
137.0896
128.7602
128.2870
128.1274
128.0397
127.6063
127.4737
127.1543
126.2337
125.7867
111.9849
98.1038
97.6700
71.0043
58.5272
57.4644
55.4506
37.7447
29.6932
20.0879
ArHN
13y
Ar = 2-OBn-5-OMePh
f1 (ppm)

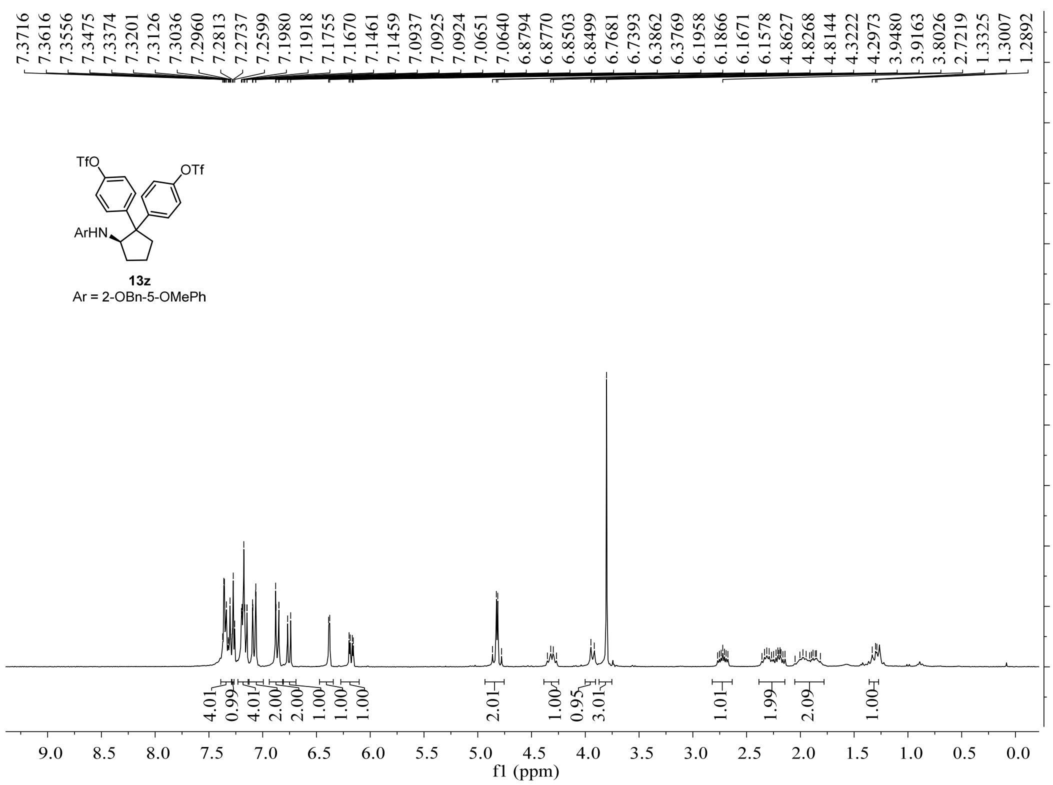
7.3716
7.3616
7.3556
7.3475
7.3374
7.3201
7.3126
7.3036
7.2960
7.2813
7.2737
7.2599
7.1980
7.1918
7.1755
7.1670
7.1461
7.1459
7.0937
7.0925
7.0924
7.0651
7.0640
6.8794
6.8770
6.8503
6.8499
6.7681
6.7393
6.3862
6.3769
6.1958
6.1866
6.1671
6.1578
4.8627
4.8268
4.8144
4.3222
4.2973
3.9480
3.9163
3.8026
2.7219
1.3325
1.3007
1.2892
TfO
OTf
ArHN
13z
Ar = 2-OBn-5-OMePh
4.01
0.99
4.01
2.00
2.00
1.00
1.00
1.00
2.01
1.00
0.95
3.01
1.01
1.99
2.09
1.00
f1 (ppm)

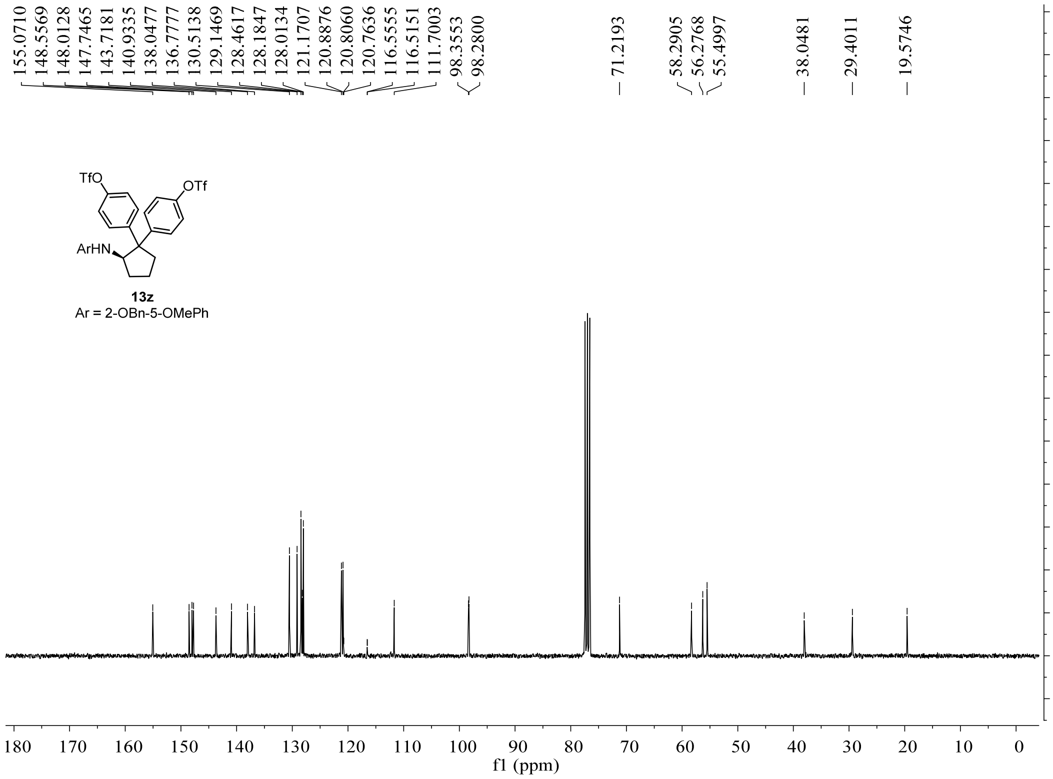
155.0710
148.5569
148.0128
147.7465
143.7181
140.9335
138.0477
136.7777
130.5138
129.1469
128.4617
128.1847
128.0134
121.1707
120.8876
120.8060
120.7636
116.5555
116.5151
111.7003
98.3553
98.2800
71.2193
58.2905
56.2768
55.4997
38.0481
29.4011
19.5746
TfO
OTf
ArHN
13z
Ar = 2-OBn-5-OMePh
f1 (ppm)

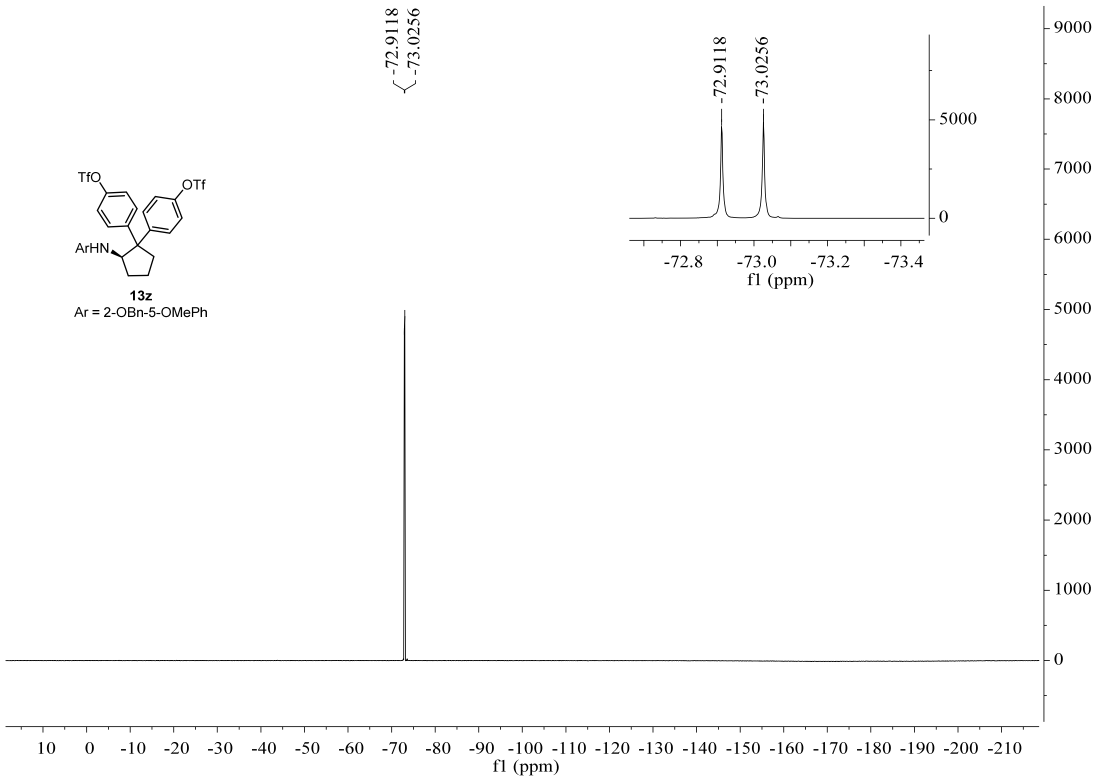
-72.9118
-73.0256
TfO
OTf
ArHN
13z
Ar = 2-OBn-5-OMePh
f1 (ppm)

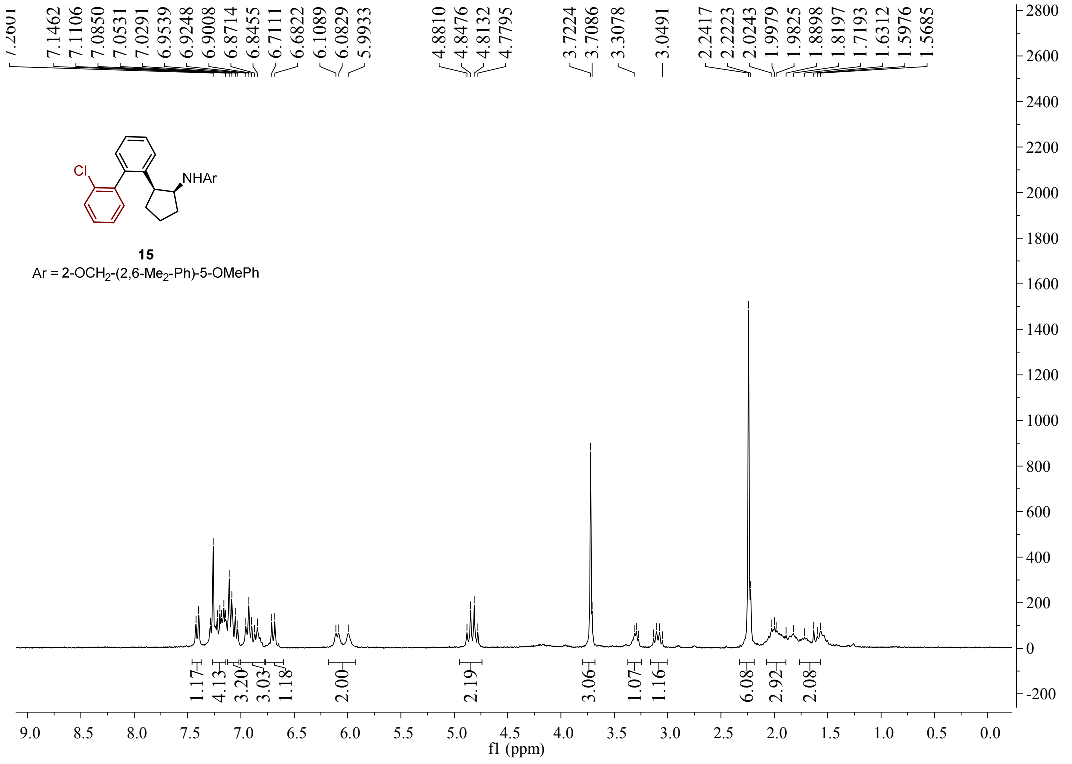
7.1462
7.1106
7.0850
7.0531
7.0291
6.9539
6.9248
6.9008
6.8714
6.8455
6.7111
6.6822
6.1089
6.0829
5.9933
4.8810
4.8476
4.8132
4.7795
3.7224
3.7086
3.3078
3.0491
2.2417
2.2223
2.0243
1.9979
1.9825
1.8898
1.8197
1.7193
1.6312
1.5976
1.5685
Cl
NHAr
15
Ar = 2-OCH2-(2,6-Me2-Ph)-5-OMePh
1.17
4.13
3.20
3.03
1.18
2.00
2.19
3.06
1.07
1.16
6.08
2.92
2.08
f1 (ppm)

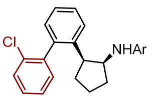

15
Ar = 2-OCH$_2$-(2,6-Me$_2$-Ph)-5-OMePh

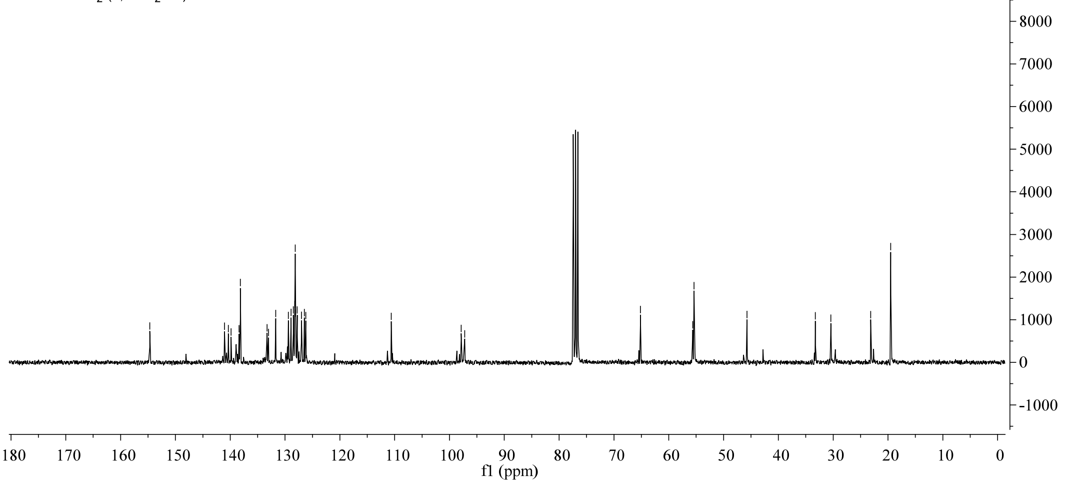

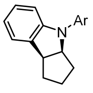

16
Ar = 2-OCH$_2$-(2,6-Me$_2$-Ph)-5-OMePh

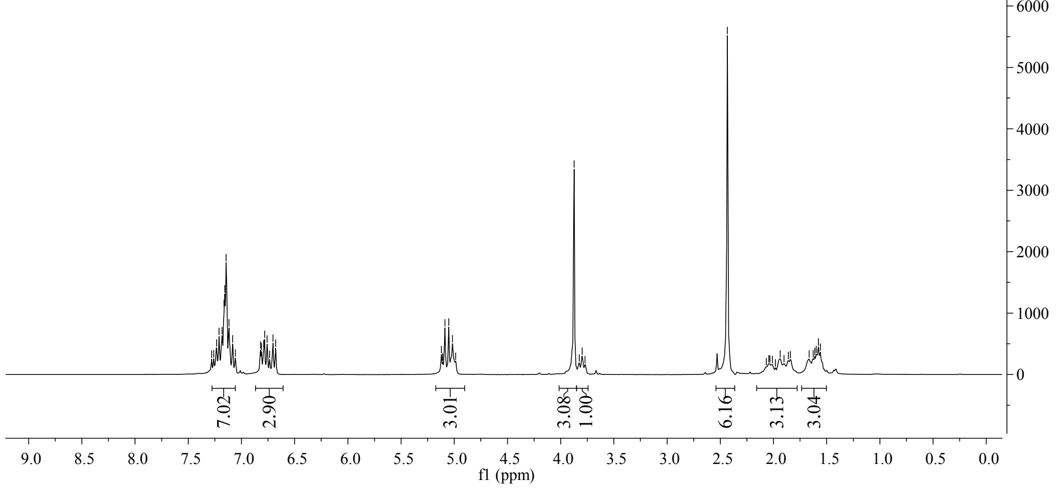

16

Ar = 2-OCH$_2$-(2,6-Me$_2$-Ph)-5-OMePh

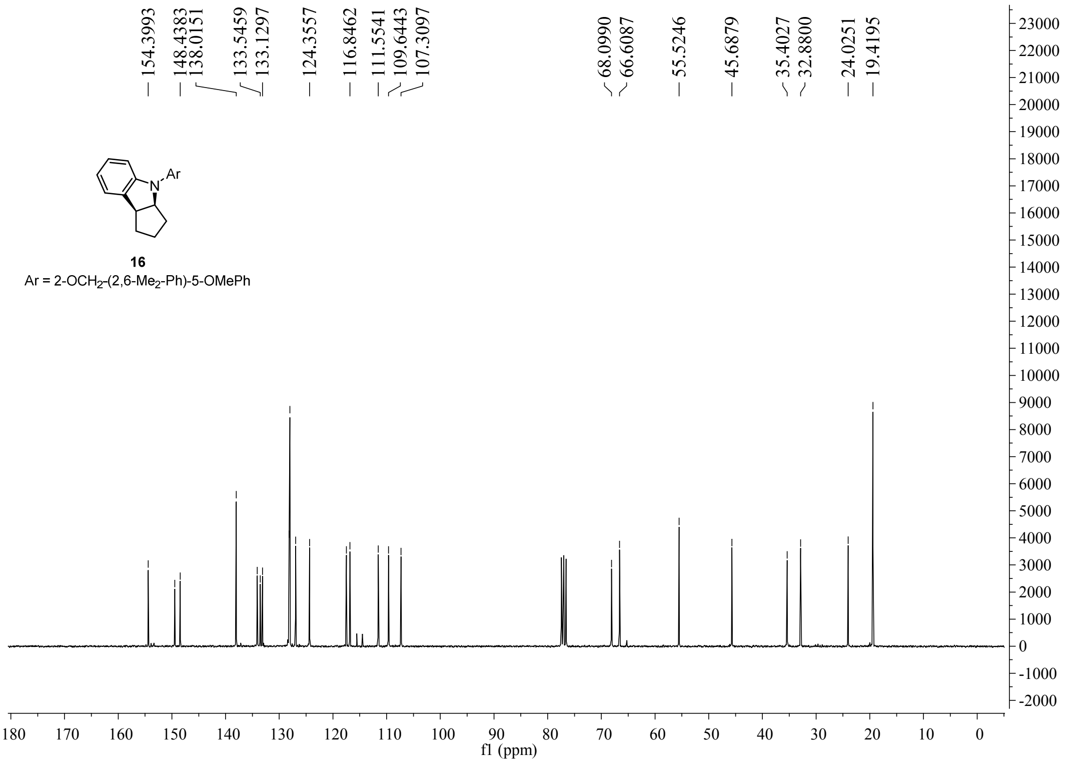

17

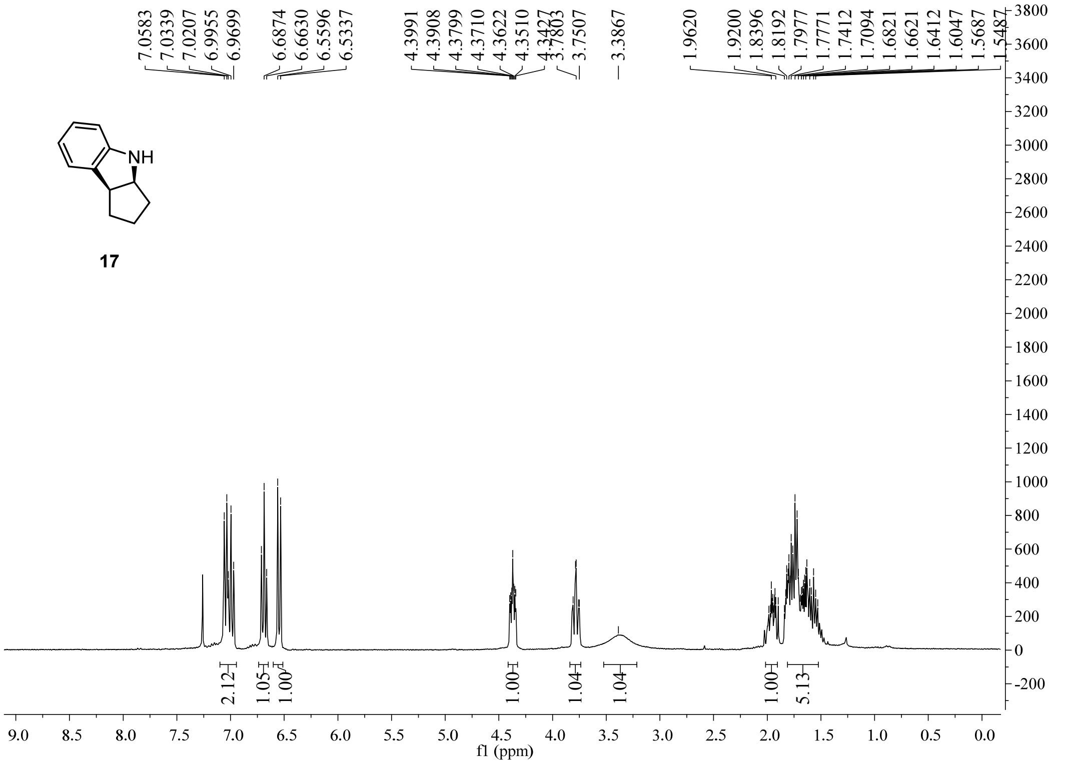

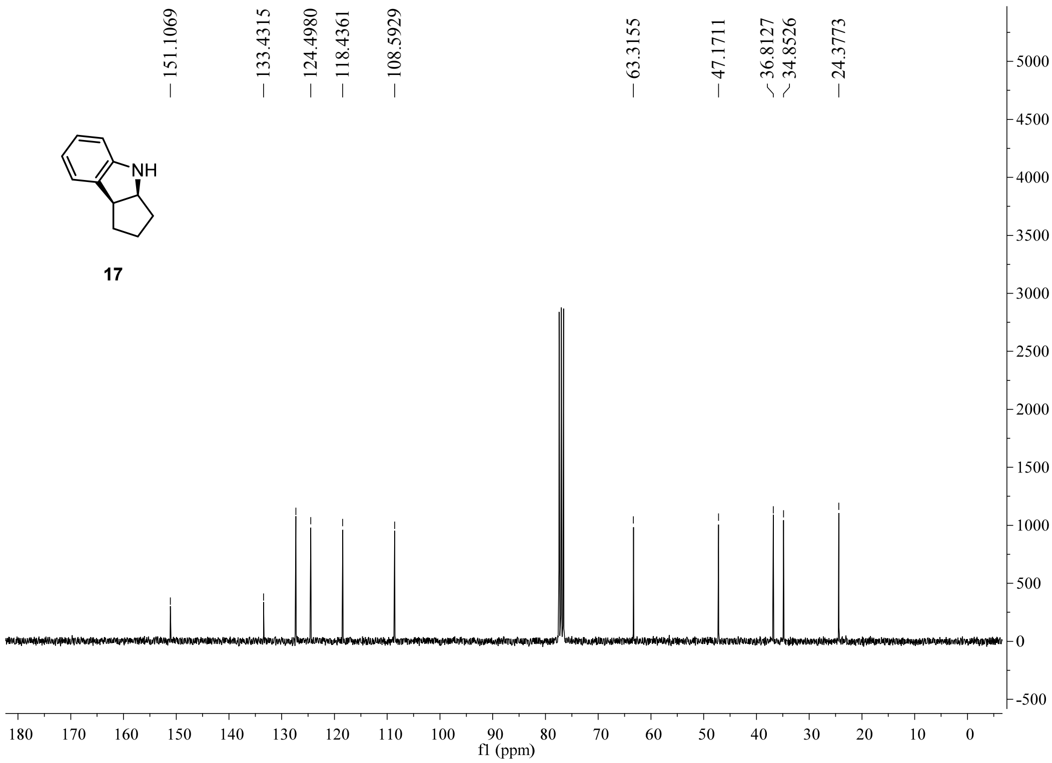
151.1069
133.4315
124.4980
118.4361
108.5929
63.3155
47.1711
36.8127
34.8526
24.3773
NH
17
fl (ppm)

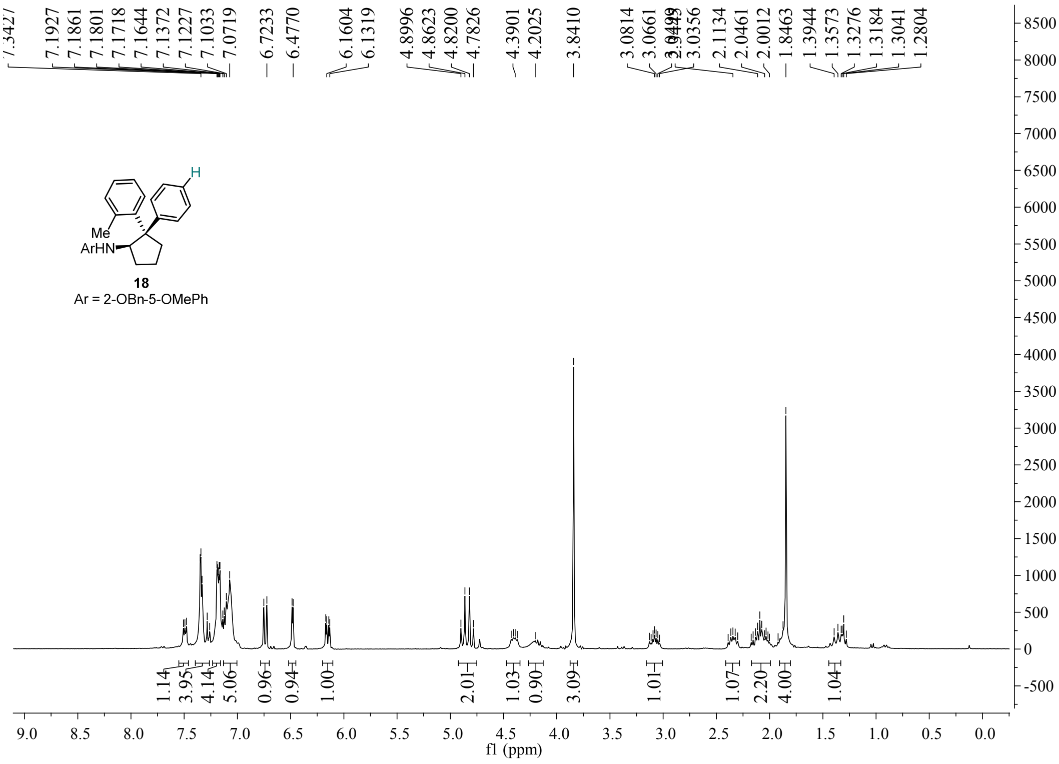
7.1927
7.1861
7.1801
7.1718
7.1644
7.1372
7.1227
7.1033
7.0719
6.7233
6.4770
6.1604
6.1319
4.8996
4.8623
4.8200
4.7826
4.3901
4.2025
3.8410
3.0814
3.0661
3.0356
2.1134
2.0461
2.0012
1.8463
1.3944
1.3573
1.3276
1.3184
1.3041
1.2804
H
Me
ArHN
18
Ar = 2-OBn-5-OMePh
1.14
3.95
4.14
5.06
0.96
0.94
1.00
2.01
1.03
0.90
3.09
1.01
1.07
2.20
4.00
1.04
fl (ppm)

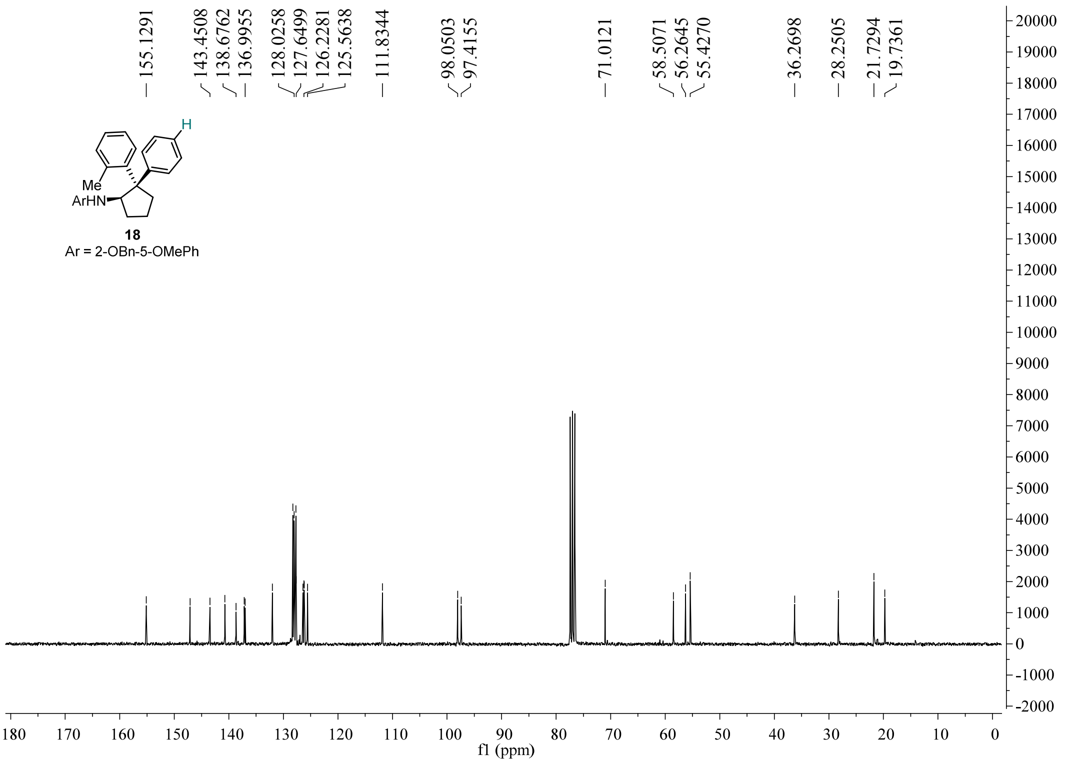
155.1291
143.4508
138.6762
136.9955
128.0258
127.6499
126.2281
125.5638
111.8344
98.0503
97.4155
71.0121
58.5071
56.2645
55.4270
36.2698
28.2505
21.7294
19.7361
H
Me
ArHN
18
Ar = 2-OBn-5-OMePh
fl (ppm)

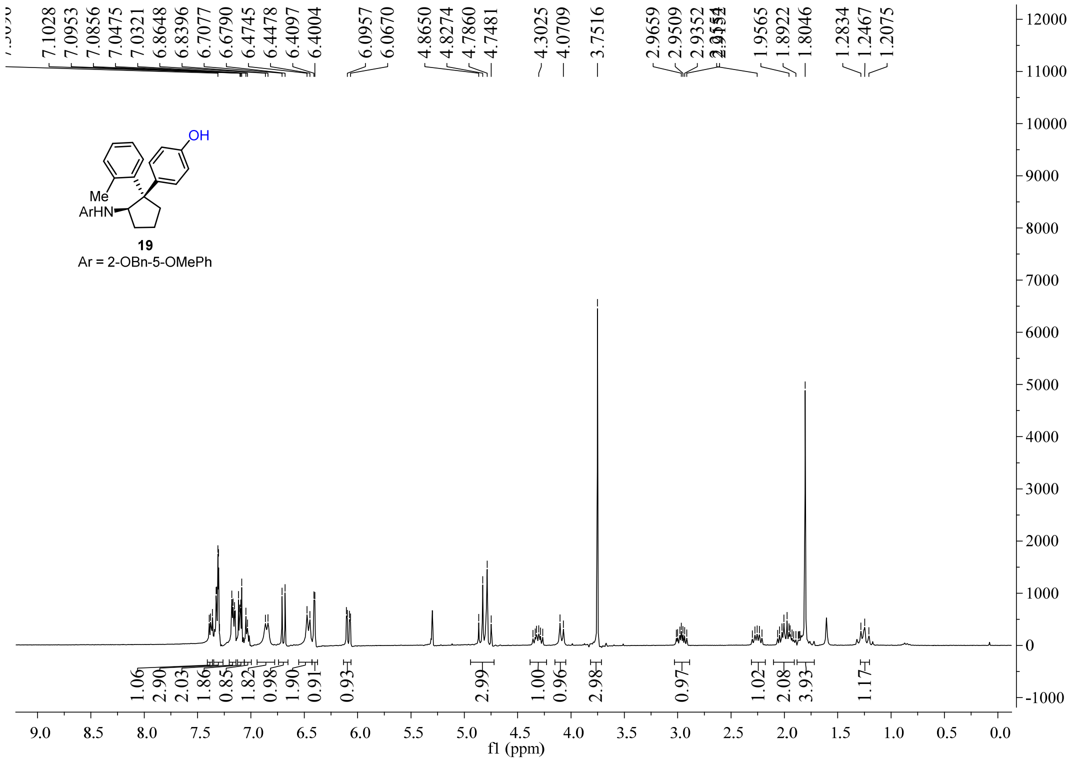
7.1028
7.0953
7.0856
7.0475
7.0321
6.8648
6.8396
6.7077
6.6790
6.4745
6.4478
6.4097
6.4004
6.0957
6.0670
4.8650
4.8274
4.7860
4.7481
4.3025
4.0709
3.7516
2.9659
2.9509
2.9352
1.9565
1.8922
1.8046
1.2834
1.2467
1.2075
OH
Me
ArHN
19
Ar = 2-OBn-5-OMePh
1.06
2.90
2.03
1.86
0.85
1.82
0.98
1.90
0.91
0.93
2.99
1.00
0.96
2.98
0.97
1.02
2.08
3.93
1.17
fl (ppm)

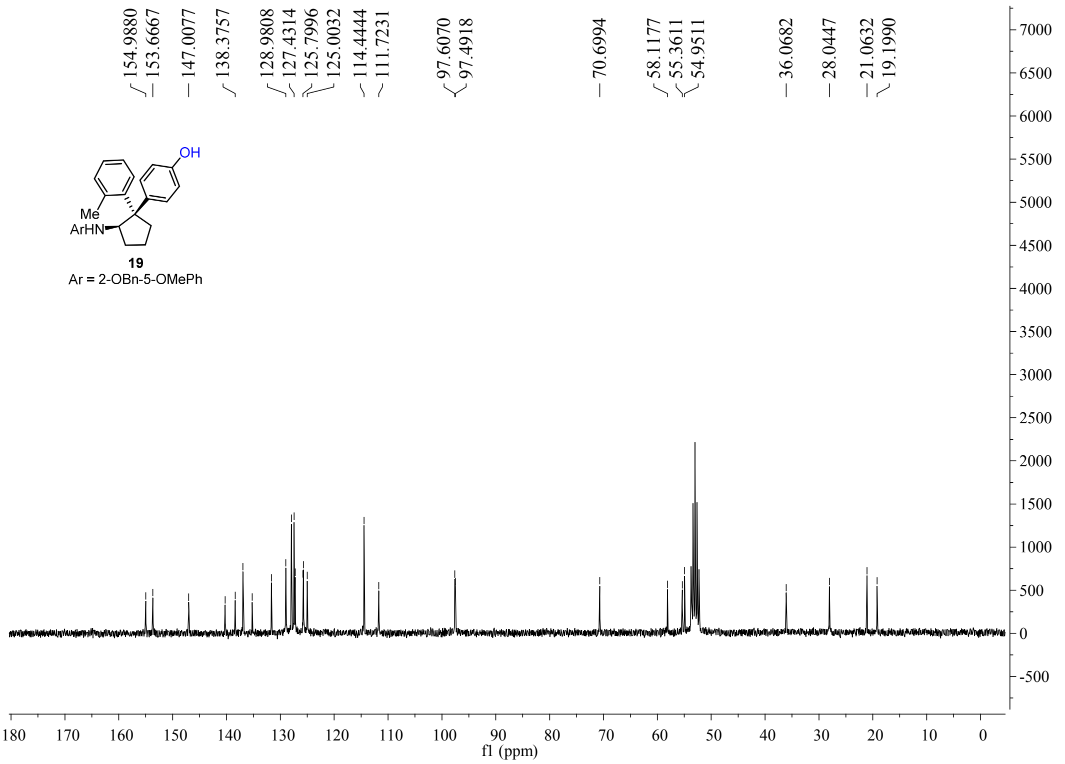
154.9880
153.6667
147.0077
138.3757
128.9808
127.4314
125.7996
125.0032
114.4444
111.7231
97.6070
97.4918
70.6994
58.1177
55.3611
54.9511
36.0682
28.0447
21.0632
19.1990
OH
Me
ArHN
19
Ar = 2-OBn-5-OMePh
fl (ppm)

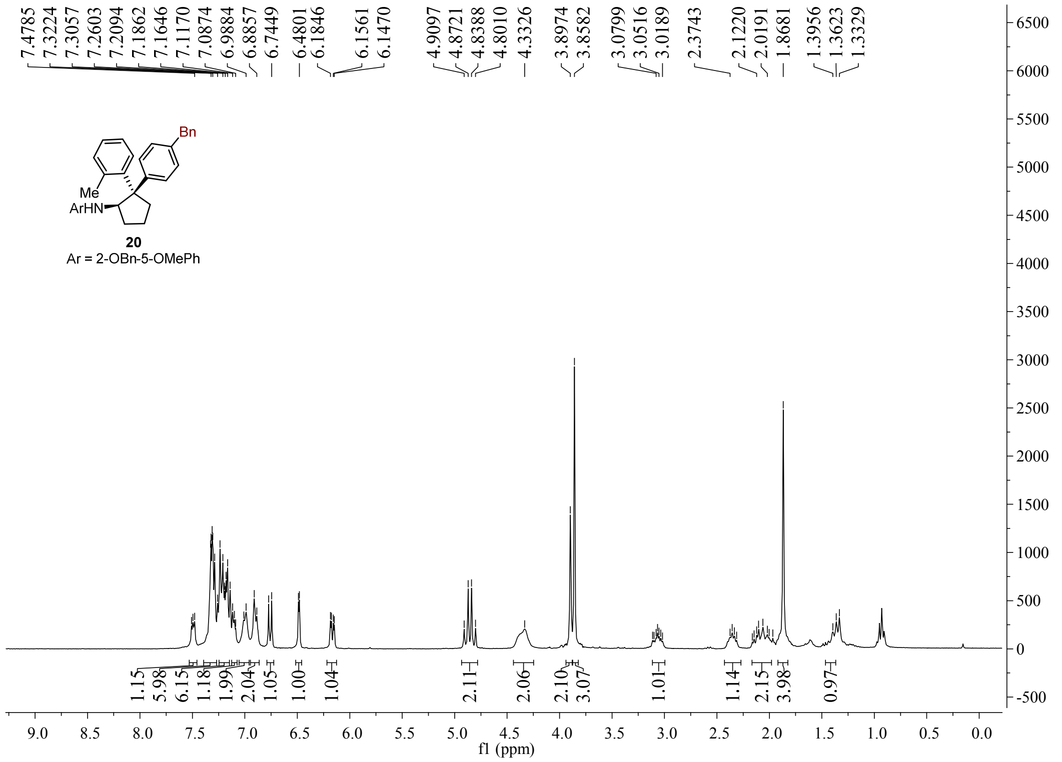
7.4785
7.3224
7.3057
7.2603
7.2094
7.1862
7.1646
7.1170
7.0874
6.9884
6.8857
6.7449
6.4801
6.1846
6.1561
6.1470
4.9097
4.8721
4.8388
4.8010
4.3326
3.8974
3.8582
3.0799
3.0516
3.0189
2.3743
2.1220
2.0191
1.8681
1.3956
1.3623
1.3329
Bn
Me
ArHN
20
Ar = 2-OBn-5-OMePh
1.15
5.98
6.15
1.18
1.99
2.04
1.05
1.00
1.04
2.11
2.06
2.10
3.07
1.01
1.14
2.15
3.98
0.97
fl (ppm)

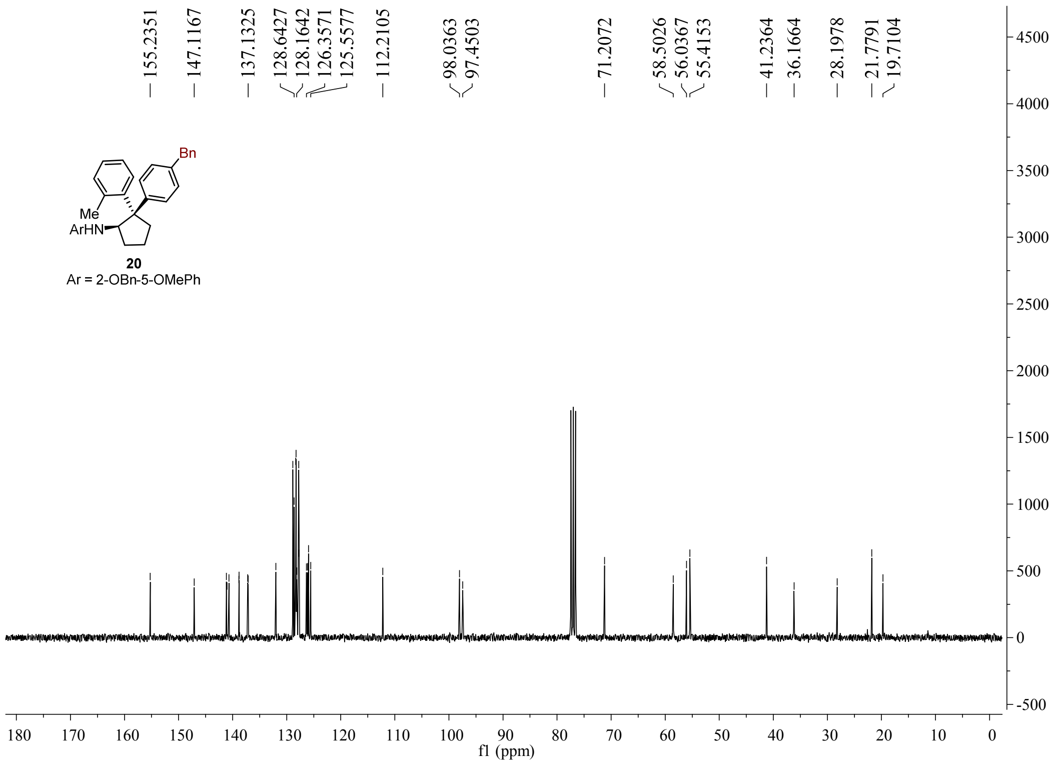
155.2351
147.1167
137.1325
128.6427
128.1642
126.3571
125.5577
112.2105
98.0363
97.4503
71.2072
58.5026
56.0367
55.4153
41.2364
36.1664
28.1978
21.7791
19.7104
Bn
Me
ArHN
20
Ar = 2-OBn-5-OMePh
f1 (ppm)

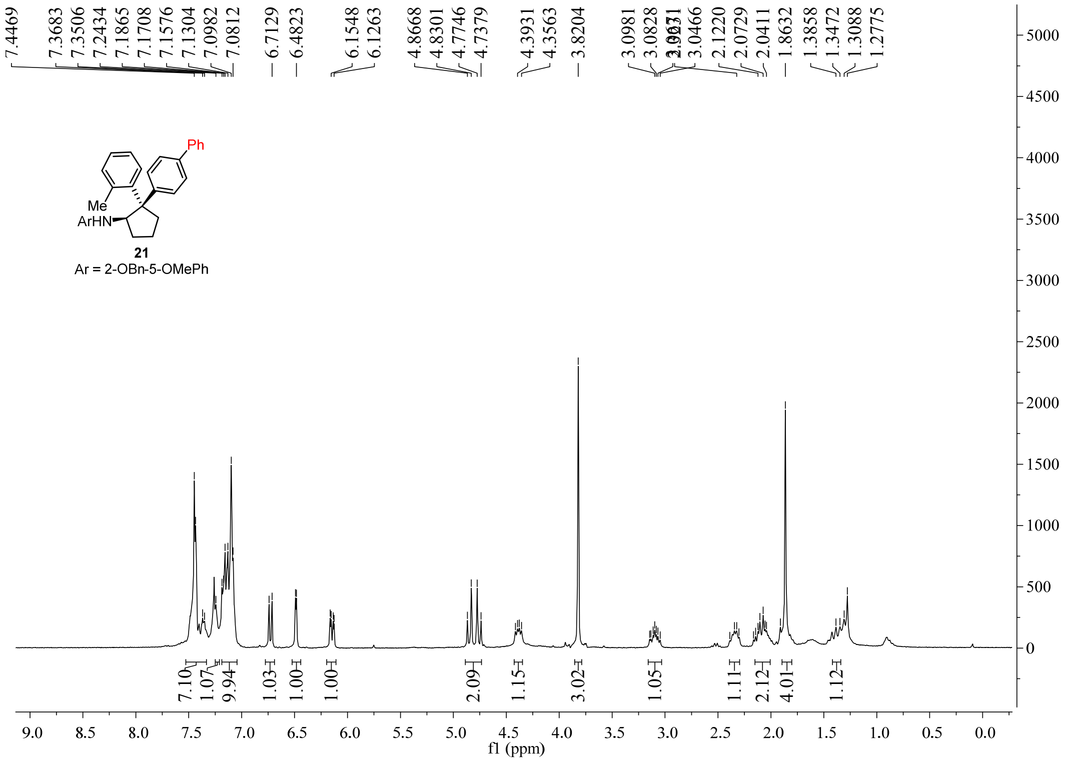
7.4469
7.3683
7.3506
7.2434
7.1865
7.1708
7.1576
7.1304
7.0982
7.0812
6.7129
6.4823
6.1548
6.1263
4.8668
4.8301
4.7746
4.7379
4.3931
4.3563
3.8204
3.0981
3.0828
3.0466
2.1220
2.0729
2.0411
1.8632
1.3858
1.3472
1.3088
1.2775
Ph
Me
ArHN
21
Ar = 2-OBn-5-OMePh
7.10
1.07
9.94
1.03
1.00
1.00
2.09
1.15
3.02
1.05
1.11
2.12
4.01
1.12
f1 (ppm)

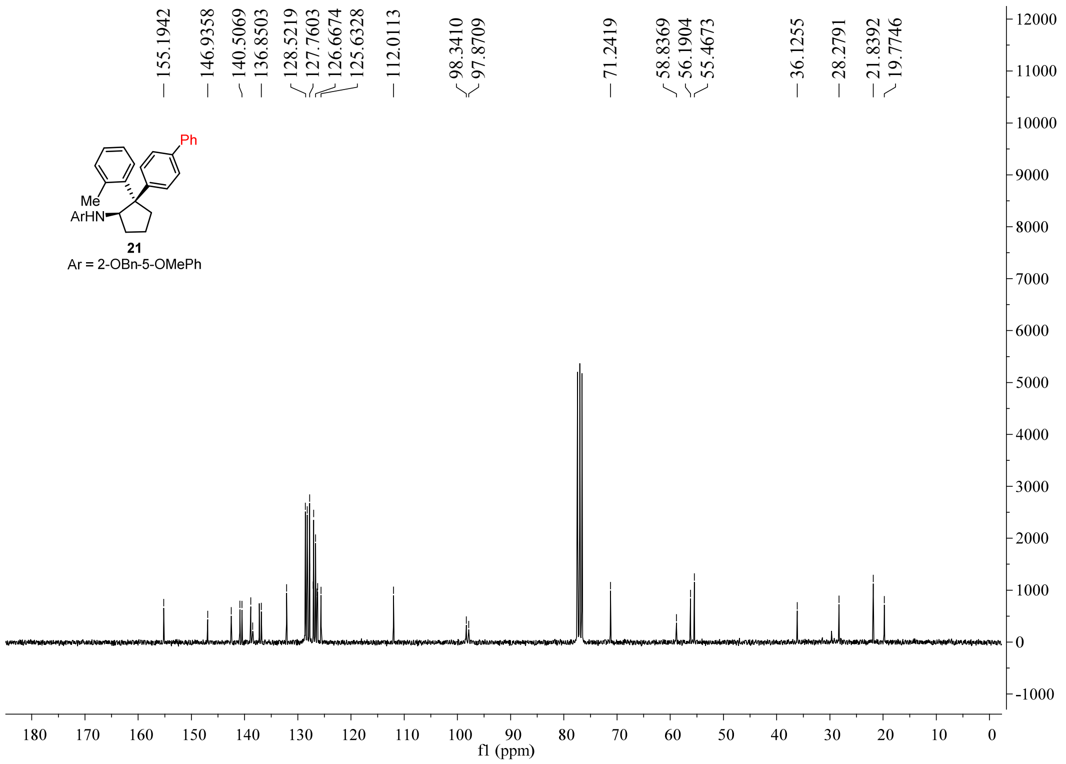
155.1942
146.9358
140.5069
136.8503
128.5219
127.7603
126.6674
125.6328
112.0113
98.3410
97.8709
71.2419
58.8369
56.1904
55.4673
36.1255
28.2791
21.8392
19.7746
Ph
Me
ArHN
21
Ar = 2-OBn-5-OMePh
f1 (ppm)

9. Other attempted substrates

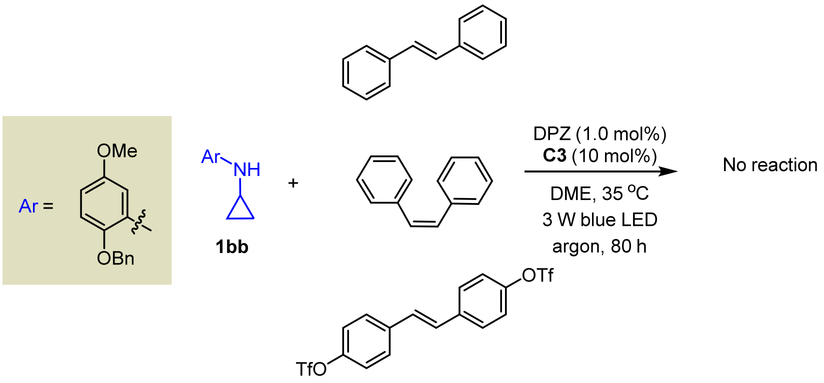

Comments: We carried out a series of transformations between **1bb** with *trans*-stilbene, *cis*-stilbene, and *trans*-stilbene that features OTf group on the aromatic rings under the established reaction conditions. However, no reaction was observed (please read the below figure). We suggest that the higher steric hindrance of such nonterminal olefins leads to the failure of the transformations.
